# Genome-wide DNA methylation profiling predicts relapse in childhood B-cell acute lymphoblastic leukaemia

**DOI:** 10.1111/bjh.12113

**Published:** 2012-10-30

**Authors:** Juan Sandoval, Holger Heyn, Jesús Méndez-González, Antonio Gomez, Sebastian Moran, Montserrat Baiget, Montserrat Melo, Isabel Badell, Josep F Nomdedéu, Manel Esteller

**Affiliations:** 1 Cancer Epigenetics and Biology Programme (PEBC), Bellvitge Biomedical Research Institute (IDIBELL), L Hospitalet de LlobregatBarcelona, Catalonia, Spain; 2 Department of Genetics, Hospital de la Santa Creu i Sant PauBarcelona, Catalonia, Spain; 3 Department of Paediatrics, Hospital Parc Taulí de SabadellBarcelona, Catalonia, Spain; 4 Department of Paediatric Haematology, Hospital de la Santa Creu i Sant PauBarcelona, Catalonia, Spain; 5 Department of Haematology, Hospital de la Santa Creu i Sant PauBarcelona, Catalonia, Spain; 6 Department of Physiological Sciences II, School of Medicine, University of BarcelonaBarcelona, Catalonia, Spain; 7 Institució Catalana de Recerca i Estudis Avançats (ICREA)Barcelona, Catalonia, Spain

**Keywords:** childhood B-ALL, relapse, survival, DNA methylation, Infinium 450K assay

Although the five-year survival of childhood acute lymphoblastic leukaemia (ALL) exceeds 80%, a group of patients presents poor prognosis due to early relapse (van den Berg *et al*, 2011). To date, treatment strategies have been defined by cytogenetically-based subtype categorization. However, ALL patients without chromosomal translocations associated with poor prognosis lack diagnostic markers that would allow specific therapies to be developed. DNA methylation alteration is a frequent event in cancer and is potentially very useful in the diagnosis, prognosis and prediction of drug response (Rodríguez-Paredes & Esteller, 2011). Hence, we attempted to characterize childhood B-ALLs without Philadelphia (*BCR-ABL1*) and *MLL* translocations on the basis of the DNA methylation profile of more than 450 000 CpG sites with the aim of providing a means to improve the accuracy of prognosis and treatment strategies. All the obtained DNA methylation data have been deposited in the Gene Expression Omnibus (GEO) database in the following link: http://www.ncbi.nlm.nih.gov/geo/query/acc.cgi?token=bfsbfcigsakcuty&acc=GSE39141

We derived genome-wide DNA methylation profiles of 29 childhood B-ALL patients and four normal B-cell samples (NBC) using the Infinium 450 K DNA methylation Bead assay (450 K) (Deneberg *et al*, 2011). Twenty-five patient samples were obtained at the time of diagnosis (including 10 cytogenetically normal, eight hyperdiploid, five pseudodiploid and two unclassified samples) and four samples at disease relapse (Table 1). Profiling varying 11 112 CpG sites (SD>0·25) within all samples analysed clearly distinguished healthy B-cell specimens from B-ALL patient samples (Fig 1A).

**Figure 1:**
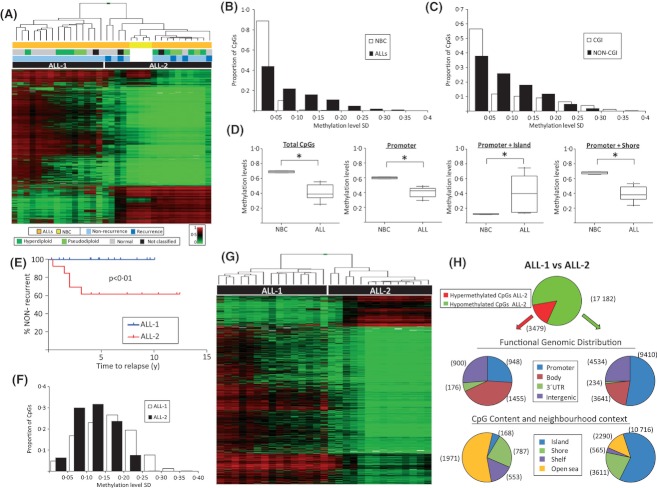
Genome-wide DNA methylation profile of B-cell ALL patients. (A) Unsupervised hierarchical clustering of four normal B-cell donors (yellow) and 29 ALL patients (orange) using CpGs with standard deviation>0.25. The cytogenetic subtypes and disease recurrence is indicated. (B) Comparison of variability at 436,346 CpG sites across normal B-cell (NBC) and B-ALL (ALL) samples. (C) Variability of methylation levels across CpG sites within CpG islands (CGI) and outside CpG islands (non-CGI). (D) Box plot displaying the distribution of β-values of total, promoter, islands in promoters and shores in promoters associated with differentially methylated CpG sites of B-ALL *versus* NBC samples. Significance is indicated by an asterisk. (E) Kaplan-Meier curve showing time to relapse of patients in B-ALL group 1 (ALL-1) and group 2 (ALL-2). Biopsies from four of the five patients that recurred were taken at the time of relapse. (F) Variability of DNA methylation levels in differentially methylated CpG sites in groups ALL-1 and ALL-2. (G) Hierarchical cluster of 20 661 differentially methylated CpG sites between B-ALL groups ALL-1 and ALL-2. (H) Genomic distribution of the 17 182 hypomethylated and 3 479 hypermethylated CpGs sites in ALL-2 compared with group ALL-1 with respect to functional genomic distribution (promoter, gene body, 3′UTR and intergenic) and CpG content (CpG island, shore, shelf and open sea).

**Table 1:** Clinical characteristics of the B-ALL samples

Number of patients	29
Age, years; median (range)	4·0 (1·2–17)
<1 year (%)	0 (0)
1-10 years (%)	21 (72)
>10 years (%)	8 (28)
Sex	
Male (%)	15 (52)
Female (%)	14 (48)
White blood cell count	
<50 x 10^9^/l (%)	23 (79)
>50 x 10^9^/l (%)	4 (14)
Unknown (%)	2 (7)
Cytogenetic abnormality	
High Hyperdiploidy (51–81 chromosomes) (%)	8 (28)
Pseudodiploidy (%)	6 (21)
Normal (%)	12 (41)
No result (%)	3 (10)
Treatment protocol	
SHOP/LAL 99 (%)	14 (48)
SHOP/LAL 2005 (%)	11 (38)
Not specified (%)	4 (14)
Median follow-up (years)	6·5
Relapse	
Yes (%)	5 (17)*
No (%)	24 (83)

* Four out of five samples taken at relapse.

SHOP/LAL, Sociedad Española de Hematología Pediátrica /Leucemia Aguda Linfoblástica.

Overall, B-ALL samples had a significantly greater variance in DNA methylation level than NBC samples (Wilcoxon test, *P* < 0·01) with 11% and 56% showing a standard deviation greater than 0·05% in healthy and ALL samples, respectively (Fig 1B). We further noted that most of the variation in ALL samples was a loss of DNA methylation located outside of the CpG rich (CpG island; CGI) context (62%, SD>0·05). However, the highest variance (SD≥0·25) was DNA hypermethylation found within the CGI context (Fig 1C, Fig S1), consistent with previous results obtained with lower coverage platforms (Milani *et al*, 2010).

To obtain closer insight into the nature of variant sites, we determined differentially methylated CpG sites (dmCpGs) between healthy and cancer specimens (Table S1). We identified 3,414 dmCpGs, 88·2% (3,014) and 11·8% (400) of which respectively lost and gained DNA methylation in cancer samples (Fig 1D; Fig S2). Despite the predominantly hypomethylated dmCpGs, CGIs in gene promoters significantly gained DNA methylation at dmCpGs (Wilcoxon test; *P* < 0·01). Interestingly, the CGIs flanking CpG-poor regions (CpG island shores) were hypomethylated in ALL samples (Wilcoxon test; *P* < 0·01), consistent with previous studies identifying both regions as being highly variable in different cancer types, including leukaemia (Milani *et al*, 2010; Irizarry *et al*, 2009).

Analysing variant CpG sites in an unsupervised manner, we identified two clearly distinct DNA methylome profiles in B-ALL patients (Fig 1A). While 14 samples (ALL-1) displayed highly aberrant methylation levels compared with the control, 15 samples (ALL-2) showed close similarities to healthy B-cells. Most strikingly, the five with disease-relapse-associated samples were all present in ALL-2 (5/15; X^2^ test, *P* < 0·01), presenting a signature with a significant association between DNA methylation and disease-free survival (log-rank Mantel-Cox test, *P* < 0·01; Fig 1E) and suggesting a possible application in future therapy strategies by taking into account epigenetically defined groups as previously suggested (Milani *et al*, 2010).

Considering the presence of distinct DNA methylation subtypes in childhood B-ALLs and their potential application in clinical practice, we extracted a DNA methylation profile represented by 20 661 dmCpGs that distinguished the two groups (Table S2). In total, we detected 17 182 hypo- and 3 479 hypermethylated CpG sites in ALL-2 compared with ALL-1, respectively; differing in variance and associated with unique genomic features (Fig 1F-H). Confirming the signature in a 10-fold cross-validation model (area under the curve: 89·5), we concluded that the signature reliably detected both B-ALL subtypes and is thus an important tool for future disease diagnosis.

In order to determine the affected biological and disease-associated pathways, we analysed the gene ontology (GO) of hyper- and hypomethylated CpG sites located in gene promoters. We noticed an enrichment (GO, level 5) of genes related to lymphocyte (including B-cell) differentiation in 672 gene promoters presenting higher methylation level in ALL-2 (false discovery rate [FDR]<0·05, Table S3). These genes include *FOXP1*, *TCF3*, *BLNK*, *CD79A*, *RAG1* and *RAG2*, also associated with chromosomal translocations in ALLs and mutation in B-cell maturation-defective syndromes. We suggest that defects in the B-cell differentiation process display a unique property of samples present in ALL-2.

The 2 608 genes associated with promoters showing less methylation in ALL-2 were highly enriched in developmental genes (FDR<0·05), including 97 *HOX* genes (Table S4). From the epigenetic point of view, developmental genes are associated with the Polycomb (Pc) complex and are marked by the histone modification H3K27me3. Interestingly, 43% (281 out of 654) of Polycomb target genes (PcTG) displayed lower methylation in ALL-2 gene promoters and were significantly enriched compared with promoters gaining methylation (X^2^ test, *P* < 0·01) (Lee *et al*, 2006). Accordingly, we found 24% (2181 out of 9052) of hypomethylated CpG sites associated with and significantly enriched in the Polycomb histone mark H3K27me3 (X^2^ test, *P* < 0·01, Fig S3) (Ernst *et al*, 2011). Taken together, the GO and PcTG analyses suggest that hypomethylation of genes involved in developmental processes are a unique feature of patient samples present in ALL-2. Hypermethylation of PcTG (Deneberg *et al*, 2011) has been previously identified as good prognostic markers in acute myeloid leukaemia patients, supporting the predictive potential of DNA methylation signatures in leukaemia. In addition, overexpression of *HOX* genes has been related to oncogenic transformation (Bach *et al*, 2010) and an increase in stem cell self-renewal (Ross *et al*, 2012) in leukaemia cells.

Overall, genome-wide screening of DNA methylation in normal B-cells and primary B-ALL samples revealed distinct profiles, but most importantly defined two previously unknown B-ALL subtypes. Furthermore, we hypothesize that epigenetic changes mediate an undifferentiated stem cell-like phenotype of a newly identified B-ALL subtype that is possibly associated with drug resistance, resulting in disease relapse and presenting a signature of potential clinical use in the future.

## Supporting Information

**Fig S1**DNA methylation level in different genomic features. **Fig S2**Differentially methylated CpG site (dmCpG) between B-cell (NBC) and B-ALL (ALL) samples. **Fig S3**Relative number of hyper- and hypomethylated differentially methylated sites between ALL-1 and ALL-2 considering their overlap with loci occupied by different histone marks, H2A.Z and CTCF. **Table S1**3,414 differentially methylated CpG sites comparing healthy B-cells and B-ALL samples. **Table S2**20,661 differentially methylated CpG sites comparing B-cell group ALL-1 and ALL-2. **Table S3**Gene ontology analysis of hypomethylated gene promoters in group ALL-2.
