# Supplementary material for: Genome-wide DNA methylation profiling predicts relapse in childhood B-cell acute lymphoblastic leukaemia
Source: Br J Haematol. 2012 Oct 30;160(3):406–9. doi: 10.1111/bjh.12113 (PMC3568176; doi:10.1111/bjh.12113)
Supplement: Supplementary file 1 — Fig S1DNA methylation level in different genomic features. Fig S2Differentially methylated CpG site (dmCpG) between B-cell (NBC) and B-ALL (ALL) samples. Fig S3Relative number of hyper- and hypomethylated differentially methylated sites between ALL-1 and ALL-2 considering their overlap with loci occupied by different histone marks, H2A.Z and CTCF. Table S13,414 differentially methylated CpG sites comparing healthy B-cells and B-ALL samples. Table S220,661 differentially methylated CpG sites comparing B-cell group ALL-1 and ALL-2. Table S3Gene ontology analysis of hypomethylated gene promoters in group ALL-2. [file bjh0160-0406-sd1.pdf]

Supplementary information

1. **Patients and Methods**

2. **Supplementary Figures**

3. **Supplementary Tables**

1. Patients and Methods

Patient samples

Ficoll-enriched, cryopreserved bone marrow samples from 29 children diagnosed with B-ALL were obtained from the Department of Hematology of the Hospital de Sant Pau (Barcelona, Spain). Twenty-five of the samples were taken at the time of diagnosis, and four were obtained at relapse. All patients (or parents) provided informed consent to use these samples for research studies in accordance with the Declaration of Helsinki. The study was approved by the institutional review board of all participating institutions. Peripheral blood mononuclear cells (PBMCs) from healthy donors were extracted using a Ficoll gradient. To separate CD19-positive cells CD19 MicroBeads (Miltenyi Biotec) were applied following the manufacturer's instructions. DNA was extracted using phenol:chloroform:isoamylalcohol (Sigma).

Infinium HumanMethylation450 BeadChip

All DNA samples were assessed for integrity, quantity and purity by electrophoresis in a 1.3% agarose gel, picogreen quantification and nanodrop measurement. All samples were randomly distributed into 96 well plates. Bisulfite conversion of 500 ng of genomic DNA was performed using an EZ DNA methylation kit (Zymo Research) following the manufacturer's instructions. 200 ng of bisulfite-converted DNA were used for hybridization on the HumanMethylation450 BeadChip (Illumina). Briefly, samples were whole genome amplified, and then subjected to an enzymatic end-point fragmentation, precipitation and resuspension. The resuspended samples were hybridized onto the BeadChip for 16 hours at 48ºC and washed. A single nucleotide extension with labeled dideoxy-nucleotides was performed and repeated rounds of staining were applied with a combination of labeled antibodies differentiating between biotin and DNP. Data were normalized using GenomeStudio V2010.3 (Illumina). The DNA methylation level is displayed as β-values ranging from 0 to 1. We filtered for high signal quality ($p<0.01$), array probes not containing SNPs (dbSNP) and those located on autosomes. In total, we analyzed 436,346 CpG sites. All the obtained DNA methylation data have been deposited in the Gene Expression Omnibus (GEO) database in the followink link:
http://www.ncbi.nlm.nih.gov/geo/query/acc.cgi?token=bfsbfcigsakcuty&acc=GSE39141

Statistical analysis

Gene ontology (GO) analysis was performed using DAVID 6.7 (Huang *et al*, 2009) (National Institute of Allergy and Infectious Diseases (NIAID), NIH). Enriched GO terms

were considered significant for values of FDR below a threshold of 0.05. Differentially methylated CpG sites were selected using a threshold of a 0.3 difference (δ) in average β-values between the groups and a FDR<0.01 (Wilcoxon rank test). All clusters were created within GenomeStudio V2010.3 using the Manhattan method.

B-ALL subgroup specific signatures were confirmed by performing a ten-fold cross-validation, randomly splitting our original sample group into training and test subsets. The model cluster was built on the training subset, using a hierarchical clustering method with a complete link type, and calculating the Euclidean distances between samples. This cluster model was successfully applied to the test subset (AUC: 89.5).

Histone marks and regions occupied by CTCF and H2A.Z were identified using chromatin immunoprecipitation sequencing (ChIP-seq) experiments, enrolled in the ENCODE project processing a lymphoblastoid cell line of a healthy donor (GM12878) (Ernst *et al*, 2011).

Ernst, J., Kheradpour, P., Mikkelsen, T.S., Shoresh, N., Ward, L.D., Epstein, C.B., Zhang, X., Wang, L., Issner, R., Coyne, M., Ku, M., Durham, T., Kellis, M. & Bernstein, B.E. (2011) Mapping and analysis of chromatin state dynamics in nine human cell types. *Nature*, **473,** 43–49.

Huang, D.W., Sherman, B.T. & Lempicki, R.A. (2009) Systematic and integrative analysis of large gene lists using DAVID bioinformatics resources. *Nature Protocols*, **4,** 44–57.

2. Supplementary Figures

Supplementary figure 1. DNA methylation level in different genomic features. (**a**) Box plot displaying the distribution of total β-values of NBC and B-ALL samples regarding their functional genomic distribution: promoter, gene body, 3´UTR and intergenic. Significance is indicated. (**b**) Box plot displaying the distribution of total β-values of NBC and B-ALL samples regarding their CpG content: CpG island, shore, shelf and open sea. Significance is indicated.

Supplementary figure 2. Differentially methylated CpG site (dmCpG) between B-cell (NBC) and B-ALL (ALL) samples. (**a**) Hierarchical clustering of 4 healthy NBC samples (yellow) and 29 ALL patients (orange) using dmCpGs. (**b**) Genomic distribution of 3,414 differentially methylated CpGs sites, including 3,014 hypomethylated and 400 hypermethylated CpGs sites in B-ALLs compared to NBC regarding functional genomic distribution (promoter, gene body, 3´UTR and intergenic) and CpG content (CpG island, shore, shelf and open sea). (**c**) Up, hierarchical clustering using promoter associated dmCpG sites located in CpG islands and or shores of 4 healthy NBC samples (yellow), 29 ALL patients (orange). Below, average β-values of promoter associated dmCpG sites located in CpG islands or shores of NBC and B-ALL samples. (**d**) Genomic distribution of the 1,068 promoter associated dmCpGs, including 828 hypomethylated and 240 hypermethylated sites in B-ALLs compared to NBC regarding their CpG content (CpG island, shore, shelf and open sea).

Supplementary figure 3. Relative number of hyper- and hypomethylated differentially methylated sites between ALL-1 and ALL-2 considering their overlap with loci occupied by different histone marks, H2A.Z and CTCF.

Supplementary Figure 1

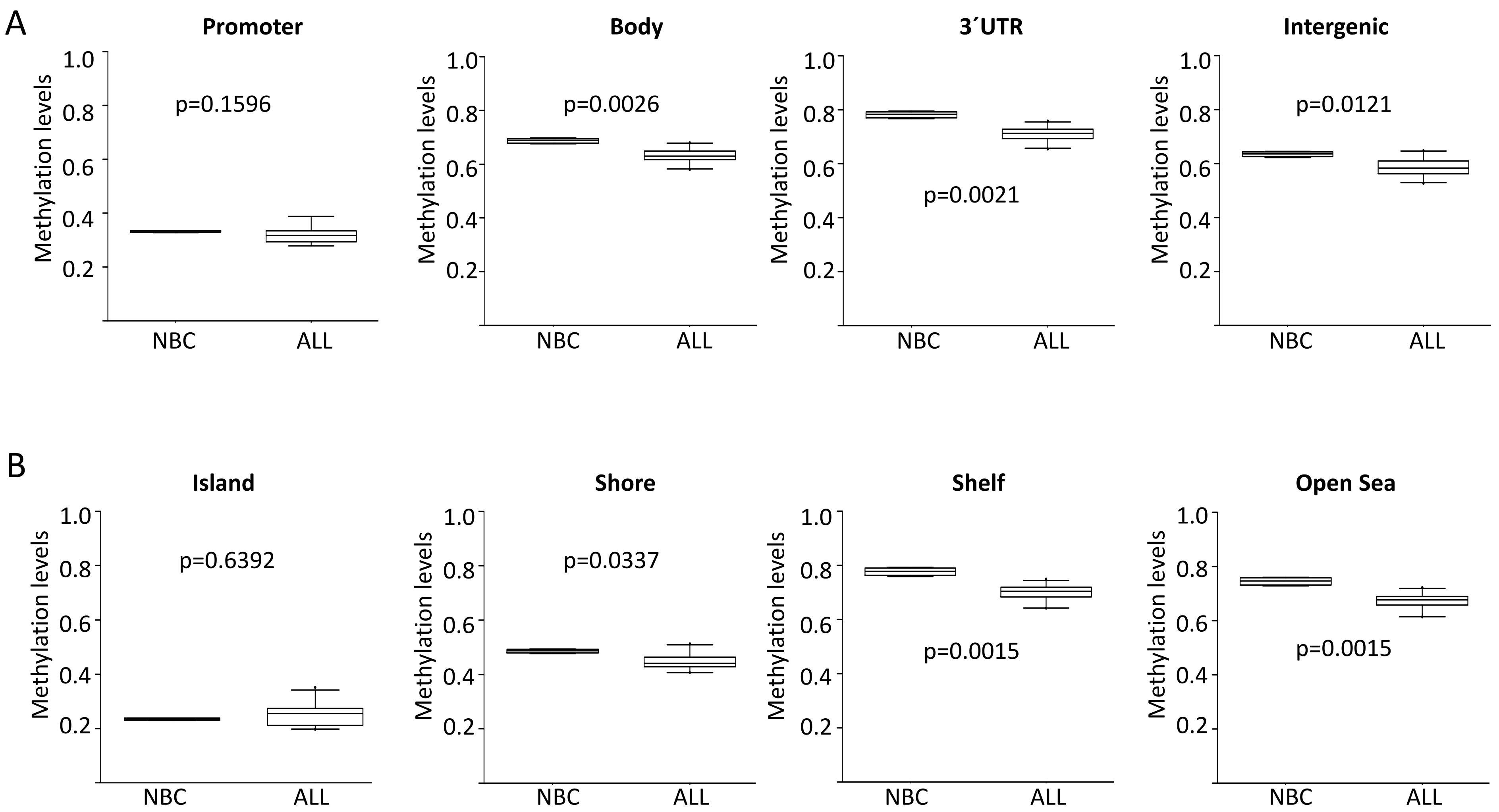

Supplementary Figure 2

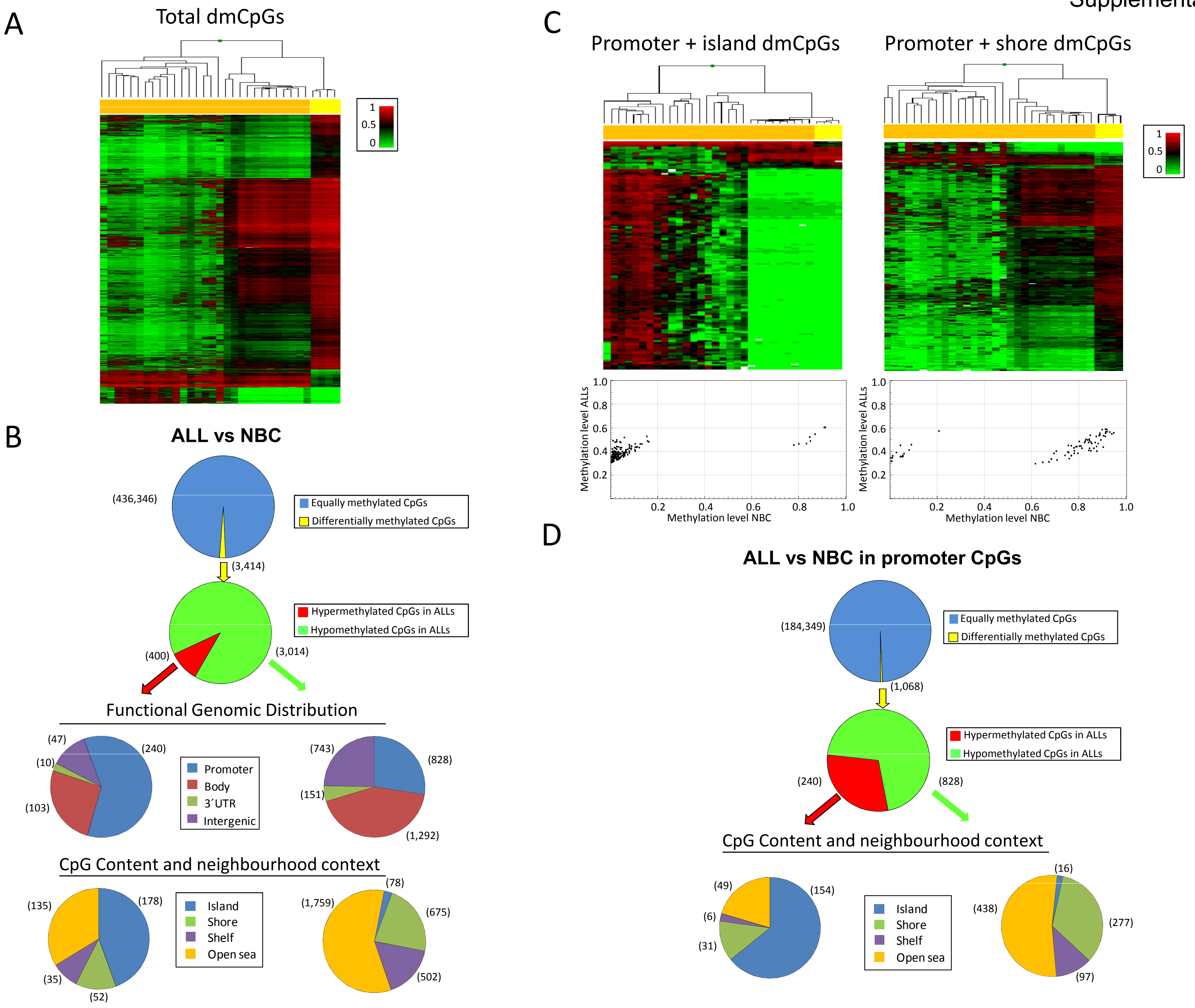

Supplementary Figure 3

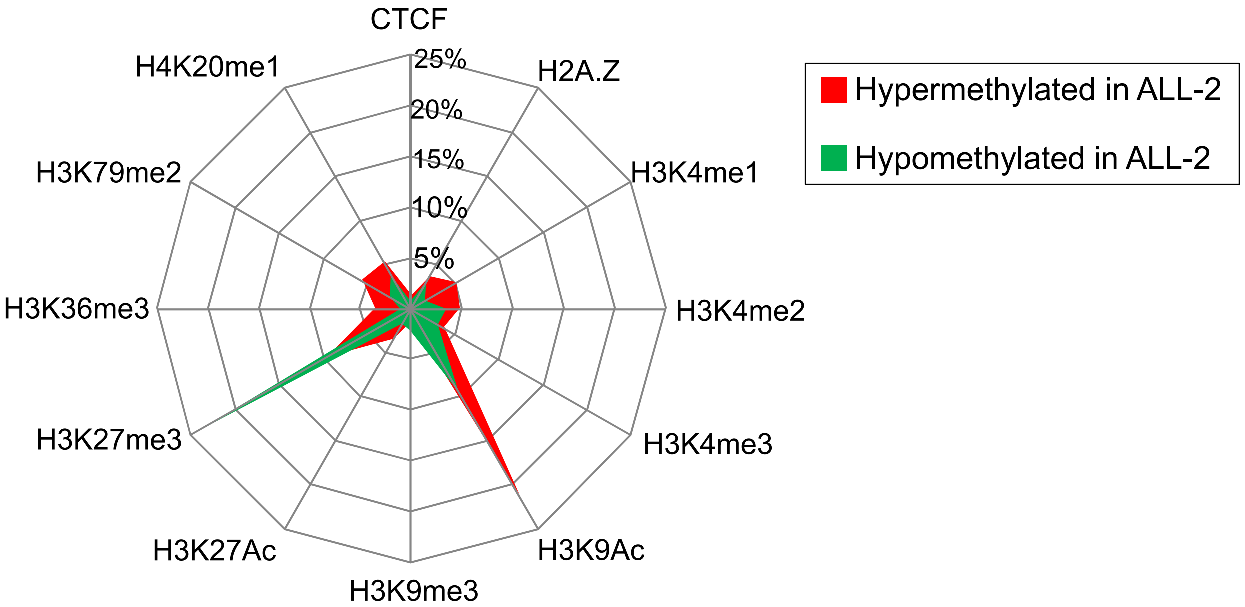

Supplementary table 1:

3,414 differentially methylated CpG sites comparing healthy B-cells and B-ALL samples.

TargetID	Gene name	FDR	δ β-value
cg13618969	FAM125B	0.000420813	0.587643713
cg16260349	TSPAN14	0.000420813	0.581527076
cg25305879		0.000420813	0.580689528
cg22062741	ARHGEF10	0.000420813	0.580321719
cg17164954	ARID1B	0.000420813	0.569513727
cg15012027		0.000420813	0.567779348
cg01752594	DLEU2	0.000420813	0.566497211
cg26574777	PCCA;PCCA	0.000420813	0.560272571
cg01606027	LIMK2	0.000420813	0.557643037
cg27660627	ANKRD11	0.000420813	0.556003573
cg21892720	LAIR1;LAIR1	0.000420813	0.554996865
cg10531355	SERINC5	0.000420813	0.55446299
cg19023589	TMCO3	0.000420813	0.553312538
cg11572080	TRAF3IP2	0.000420813	0.550520591
cg07186962		0.000420813	0.548628033
cg18830993	PELI2	0.000420813	0.548303734
cg08140558	NCRNA00171	0.000420813	0.544951783
cg08539965	EIF4G3	0.002321262	0.543304193
cg00554993	CEBPE	0.000420813	0.535818579
cg04855678	OSTalpha	0.000420813	0.535814386
cg16995791		0.000420813	0.53414575
cg21473786		0.000420813	0.533449657
cg12147994	SH3YL1	0.000420813	0.532864393
cg24663338		0.000420813	0.531343315
cg00211174	MCM2	0.000420813	0.531321989
cg09273683	PIP4K2A	0.000420813	0.527628801
cg19869035	IQCE;IQCE	0.000420813	0.525398857
cg11356375	CDYL	0.000420813	0.525274452
cg17444090		0.000420813	0.525210326
cg27260684	KIAA0513	0.000420813	0.524977049
cg21346154	TGFA;TGFA	0.000420813	0.52231356
cg17036458	SSH2	0.003004124	0.521700367
cg12237948	STK35	0.000420813	0.520230588
cg14602393		0.000420813	0.520051378
cg06512263	CDKAL1	0.000420813	0.519638365
cg08945443	ZMYND17	0.000420813	0.518456567
cg12039595		0.000420813	0.518106581
cg04739200	MYB	0.001569481	0.51786605
cg12940181		0.000420813	0.5170386
cg13814351	FAM163B	0.000420813	0.514970422
cg16353350	C10orf55	0.000420813	0.514766935
cg24833027	ARHGEF10	0.000420813	0.513894871
cg00382999	NCK1	0.002321262	0.513860757
cg24576960	FAM114A1	0.000420813	0.513591024
cg02187259	FAM49A	0.000420813	0.51177517
cg14043104	KLF12	0.000420813	0.511110647
cg10426076	PRG2	0.000420813	0.510503736
cg08130265	C15orf5;SGK269	0.000420813	0.509788284
cg16522993		0.000420813	0.508234922
cg02329430	NPTN	0.000420813	0.508179232
cg17253709	B3GNT2	0.000420813	0.507514427
cg03997145		0.000420813	0.505999591
cg04875041	VPS13D;VPS13D	0.000420813	0.505896761
cg19676553	BRF1	0.000420813	0.504450884

cg02061820	MBNL1	0.005790932	0.503144639
cg25137372	HAL;HAL	0.000420813	0.503033543
cg13381110	PHLPP1	0.001569481	0.501241353
cg15921911		0.000420813	0.500625997
cg07714276	RREB1	0.000420813	0.499622514
cg21221540		0.000420813	0.499238034
cg13599613	SH2D3C	0.001569481	0.49779937
cg01357222		0.000420813	0.497179134
cg18895476	TPST2;TPST2	0.000420813	0.495829873
cg02259723	ADPGK	0.000420813	0.495343396
cg25939853		0.000420813	0.494842134
cg14799927	TRIM26	0.000420813	0.494452283
cg09583374	ITGA5	0.000420813	0.493912038
cg01948978	ATP2B4	0.000420813	0.49366022
cg22074114	KY	0.000420813	0.493032955
cg27644327	BACH2	0.000420813	0.492259192
cg18891604		0.000420813	0.491835555
cg05859076	RAB43	0.000532358	0.491276577
cg25983531	AOAH	0.001569481	0.490251975
cg25791430	MRPL16	0.000420813	0.489449428
cg13375463	AHDC1	0.000420813	0.489251746
cg26924822	RBPJ	0.000420813	0.489181147
cg26117521	EBF1	0.000420813	0.488371318
cg07804470	STK24	0.000420813	0.486971299
cg23648239		0.000420813	0.485998797
cg11123644	TTLL4	0.000420813	0.485917165
cg14150115		0.000420813	0.48437091
cg25194194	SLC35E3	0.000420813	0.484115163
cg08670658	ADRBK1	0.000420813	0.483918083
cg07690127	MAD1L1	0.000420813	0.483411452
cg14210119		0.000420813	0.483261202
cg17494897	TSSC1	0.002321262	0.482028095
cg17291166		0.000420813	0.481507484
cg02990302	C16orf80	0.000420813	0.48125955
cg25084760	PCCA;PCCA	0.000420813	0.481161058
cg19252328	DOCK9;DOCK9;DOCK9;DOCK9	0.001569481	0.480845227
cg16903174	ASAP2;ASAP2	0.000420813	0.479279184
cg16061354	SLC43A2	0.000420813	0.479059085
cg18693141		0.000420813	0.478615681
cg02226192	ANKRD11	0.000420813	0.47850741
cg22090404	RNASEH2B;RNASEH2B	0.000420813	0.477480079
cg06496803	TBC1D14;TBC1D14	0.000420813	0.477119262
cg02484352	PDLIM4;PDLIM4	0.000420813	0.477094634
cg05760765		0.001569481	0.476793688
cg23924737	MRPS23	0.000420813	0.476417631
cg16481332	IQCE;IQCE	0.000420813	0.475469317
cg19370689	LRRC17;FBXL13;LRRC17;FBXL13	0.000420813	0.475079222
cg05949397		0.002321262	0.474486215
cg14199261	CDK6;CDK6	0.000420813	0.474479273
cg10220544	FGGY;FGGY	0.003185744	0.474296206
cg23299218	DIAPH3	0.000420813	0.473851466
cg22729539	MLXIP	0.000420813	0.473465699
cg20338754	RNF138;RNF138	0.000420813	0.473094897
cg07921759	SAMD4A;SAMD4A	0.000420813	0.472993844
cg14129169	ZNF788	0.000420813	0.472578474
cg08096381	IQSEC1	0.002321262	0.472527459
cg00828556		0.002321262	0.472138842
cg20794855	ADHFE1	0.001569481	0.471876586
cg12041266	ARRB1;ARRB1	0.000420813	0.471224904

cg04704634	IL3	0.000420813	0.471182871
cg00819078	FAM134B	0.000636165	0.471116067
cg07920381		0.000420813	0.47110648
cg04431143	CTDSPL;CTDSPL	0.000420813	0.470745818
cg17789193		0.000420813	0.470594393
cg23100428	SNAI1	0.000420813	0.470577605
cg13390284		0.000420813	0.470518053
cg26514793	CHST3	0.000420813	0.470102404
cg21671607	RAG2;C11orf74	0.002321262	0.46997861
cg02662417	ARID1B;ARID1B;ARID1B	0.000636165	0.46940284
cg15961455		0.002321262	0.469033339
cg07205203		0.002321262	0.468879898
cg22094071	FAM82B	0.000420813	0.468875656
cg06418238	RPTOR;RPTOR	0.000420813	0.468404946
cg12893030	CHST3	0.000420813	0.467884777
cg02471848		0.000420813	0.467719069
cg06380725	TRAF3IP2;TRAF3IP2;TRAF3IP2;TRAF3IP2	0.000420813	0.467701736
cg25154959	RAG1	0.005790932	0.46749922
cg00824381		0.000466745	0.467097454
cg20723412	TBC1D14;TBC1D14	0.000420813	0.466540606
cg10981439	TREM1;TREM1	0.002321262	0.466413541
cg00291213	RUNX1	0.000420813	0.466268615
cg16348358	LCK	0.000420813	0.46624192
cg18728780		0.002321262	0.466023223
cg20995304	HDAC7;HDAC7	0.000420813	0.465725272
cg17128308	STX18	0.000420813	0.46569802
cg01766943	SPTBN1;SPTBN1	0.000420813	0.465530642
cg23807570	ATP2A2;ATP2A2;ATP2A2	0.000420813	0.465525493
cg09009070	AUTS2;AUTS2	0.000420813	0.465336394
cg08796391	MAD1L1;MAD1L1;MAD1L1	0.000636165	0.465098576
cg04804052	SMARCA4;SMARCA4;SMARCA4;SMARCA4;SMAR	0.000420813	0.464980642
cg25037335		0.004997918	0.464589062
cg14154784	TLE1	0.000420813	0.464286049
cg03138446	SLC27A3	0.000420813	0.464095853
cg07197493	SBF2	0.000636165	0.463650933
cg23882164		0.000420813	0.463029993
cg09485853	PKM2;PKM2;PKM2	0.003185744	0.462839032
cg12592387		0.000420813	0.462736693
cg06538549	ITGB1;ITGB1	0.000420813	0.462286537
cg11018338	CCDC109A	0.000420813	0.462272187
cg01942816	MIR589;FBXL18	0.000420813	0.462224632
cg17237804		0.004405484	0.462046128
cg10585941	SMYD2	0.002321262	0.46201058
cg07824483	MAFG;MAFG	0.002321262	0.46176207
cg23673974	TBKBP1	0.000420813	0.46155973
cg13883633	MGA;MGA	0.000420813	0.461416714
cg20594765	GALNT2	0.000420813	0.461387668
cg16389345		0.000420813	0.461291478
cg07708453	PRDM2;PRDM2;PRDM2;PRDM2;PRDM2	0.000420813	0.461037877
cg00874877	C6orf10	0.000420813	0.460871843
cg21683390	C18orf1;C18orf1	0.000420813	0.460757498
cg19683780		0.000420813	0.460753958
cg09859659	DENND3	0.000420813	0.460575838
cg01267797	SERINC5	0.000420813	0.459986683
cg03308839	NDE1;NDE1;MYH11;MYH11;MYH11;MYH11	0.007567702	0.459862525
cg10594837	DENND3	0.000420813	0.459861662
cg05303899		0.000420813	0.458989373
cg19701828		0.000420813	0.458648183
cg23169588	TMCO3	0.000420813	0.458615174

cg01516792	RPTOR;RPTOR	0.000420813	0.458120655
cg16393899	LYRM4;LYRM4	0.000420813	0.458072424
cg15377871	CEBPE;CEBPE	0.000420813	0.457949488
cg26162326	YWHAG	0.007567702	0.457730107
cg10336193		0.000420813	0.457696781
cg01481646	TSPAN14;TSPAN14	0.000420813	0.457661055
cg09481537		0.000420813	0.457328788
cg24765748	GPRC5A	0.000636165	0.457068856
cg16846069	SCNN1A;SCNN1A;SCNN1A	0.001173833	0.456915865
cg14284211	FKBP5;FKBP5;FKBP5;FKBP5	0.002321262	0.456158954
cg15429134	LOH12CR1	0.000420813	0.45614663
cg06178179	CA7;CA7	0.000420813	0.456052117
cg02033302		0.000420813	0.45594902
cg01275661		0.000420813	0.455440289
cg12870750	CHD3;CHD3;CHD3	0.000420813	0.455020835
cg16548961		0.000420813	0.454802879
cg03801691	VGLL4;VGLL4;VGLL4;VGLL4	0.000420813	0.454617682
cg13521170	BCL2	0.000420813	0.454480623
cg05113898	RPTOR;RPTOR	0.000420813	0.454148742
cg16646003	POLS	0.000420813	0.453845874
cg13777984	ENOX1;ENOX1	0.001038267	0.453759586
cg07554408		0.002321262	0.453745857
cg16677191	GLRX;GLRX	0.000420813	0.453459127
cg17411792		0.000420813	0.453400591
cg15087459		0.003185744	0.452509222
cg03686088		0.000420813	0.452313186
cg20555462	UBASH3B	0.000420813	0.452312838
cg08210507	MAD1L1;MAD1L1;MAD1L1	0.000420813	0.451728052
cg08244301	SLC27A1	0.000420813	0.451572472
cg11190658	FAM167B	0.000420813	0.451237737
cg03846111	ATF7IP	0.000420813	0.451222099
cg13765778	DAPK1	0.000420813	0.450589722
cg05814100	RPTOR;RPTOR	0.000636165	0.450432204
cg23463186	TSPAN14;TSPAN14	0.005790932	0.450092918
cg10722267		0.001569481	0.449953805
cg24864097	GRK5	0.000420813	0.449655989
cg10374402	SH2D4B;SH2D4B;SH2D4B	0.00961166	0.449468274
cg02779913	TTC25	0.007567702	0.449233625
cg22830091	YWHAG	0.002321262	0.449222392
cg12352399	TOP2B	0.000420813	0.449108036
cg06399735	PSD3	0.000420813	0.448733082
cg06419601		0.000420813	0.448710926
cg24710671	C21orf2	0.000420813	0.448687806
cg26868097	MBTPS1	0.000420813	0.448548286
cg00329411		0.002321262	0.44823206
cg05753799	C20orf27	0.000420813	0.448218757
cg06840743		0.000420813	0.448212078
cg23200357		0.000420813	0.447909052
cg01179256	CHST11	0.000420813	0.447416005
cg06155414	MBNL2;MBNL2	0.000420813	0.447365423
cg10834480	TRAF3IP2;TRAF3IP2;TRAF3IP2	0.000420813	0.447171841
cg09169633	MAN2A2	0.000420813	0.447061174
cg11939496	CD244;CD244;CD244	0.000420813	0.446937152
cg09302474	PIK3R5	0.001038267	0.446785954
cg17433678	PFKP	0.002321262	0.446785212
cg27159096	CMTM2	0.000420813	0.446726187
cg06933796		0.001038267	0.446702135
cg08134671	GNG7	0.001569481	0.446403804
cg06684088		0.007567702	0.446222538

cg16385865		0.002321262	0.446129323
cg09232805	GALNTL6	0.000420813	0.446077954
cg14519777	CUX1;CUX1;CUX1	0.000420813	0.446024522
cg03006077	TGFBR3	0.000420813	0.445763623
cg24527636		0.000420813	0.445741667
cg13323091		0.000420813	0.445740516
cg25706447	PIGV	0.000420813	0.445275201
cg13071068	AGPAT1;AGPAT1	0.001569481	0.445071543
cg07436694	METTL9;METTL9	0.007567702	0.444968893
cg15489693	CLNK	0.000420813	0.444887161
cg09841898	MAP4K5;MAP4K5	0.000420813	0.444807153
cg05413628	CLCN7;CLCN7	0.002321262	0.444787065
cg10227830	GAB1;GAB1	0.000420813	0.444766771
cg04569233	CX3CR1;CX3CR1;CX3CR1;CX3CR1;CX3CR1	0.000420813	0.444673612
cg13365436	AP2A2	0.003185744	0.444542614
cg15288326	KIF21B	0.001038267	0.443640547
cg22893605		0.000420813	0.44360617
cg11495377		0.000420813	0.443427649
cg16675872		0.001038267	0.443207879
cg19445044		0.000466745	0.443134768
cg11198596	LRIG1	0.002321262	0.443130223
cg11555873		0.000420813	0.443060028
cg20110257	NRM	0.000420813	0.442981954
cg14919455	RREB1;RREB1;RREB1;RREB1	0.002321262	0.442874996
cg09755397		0.004405484	0.44283725
cg10951380	KIRREL	0.004405484	0.442810403
cg20275211	PPP2R2C;PPP2R2C	0.001038267	0.442493135
cg13271206	RPS3	0.001038267	0.442202737
cg09504873	DYNC1LI2	0.004405484	0.442024151
cg00901982		0.000420813	0.441757222
cg03371275	MAML2	0.000420813	0.441209308
cg25201372	TBC1D4	0.002321262	0.441144477
cg07958192	TIGD2;TIGD2	0.000420813	0.441117368
cg25020666	YWHAG	0.00961166	0.440849753
cg01182455		0.000420813	0.440796224
cg17118262	CCL1	0.000420813	0.440577517
cg23221723	TNFRSF10C	0.002321262	0.440319894
cg03689092	LIMS2;LIMS2	0.001038267	0.440060899
cg00785482		0.000420813	0.440016334
cg18098769	DNAH17	0.000420813	0.439901689
cg13374432	AKAP13;AKAP13	0.005790932	0.439884471
cg06812844	TRPM2	0.000420813	0.439572796
cg08091439	AHCYL1	0.007567702	0.439566781
cg06575692	DUS2L	0.000420813	0.439476022
cg04932082		0.002321262	0.439395702
cg20159193	NUDT3	0.000420813	0.439372883
cg10938221	KIAA0564	0.000420813	0.439287294
cg23991413	PDE4DIP	0.000420813	0.439179003
cg08970648	CISH;CISH	0.000420813	0.43899703
cg15709065	AMOTL2	0.000420813	0.438830292
cg09588284	ACRV1;ACRV1;ACRV1;ACRV1;ACRV1;ACRV1;AC	0.000420813	0.438724347
cg25958098	SLC12A6;SLC12A6;SLC12A6;SLC12A6;SLC12A6;S	0.000636165	0.438372616
cg04396303	PDE11A;PDE11A;PDE11A;PDE11A	0.000420813	0.438109363
cg10914815	STK24;STK24	0.000420813	0.437930409
cg08619712		0.001569481	0.437511961
cg16702362	MGAT5	0.000420813	0.437431366
cg14600368		0.000420813	0.437374972
cg17814814		0.000420813	0.437255364
cg00834988	AUTS2;AUTS2	0.000420813	0.436790155

cg05878073	ABCD4;ABCD4	0.001038267	0.436633297
cg06153925	RPTOR;RPTOR	0.002321262	0.436590832
cg00352106	MACF1;MACF1	0.001038267	0.436574428
cg08654262	DDAH1;DDAH1	0.000636165	0.436459131
cg00340349	CDK9	0.000420813	0.436385971
cg06937549	HNRNPH1	0.000420813	0.436162962
cg17397870	TRIM39;TRIM39	0.000420813	0.436008869
cg15823954	HMHB1	0.005790932	0.435783993
cg22489510		0.000420813	0.435774499
cg21440776	IFFO1;IFFO1;IFFO1	0.000636165	0.435720751
cg00035347	NT5C2;NT5C2	0.000420813	0.435634259
cg08837215	UBE2O	0.000420813	0.435442112
cg14329026	SLA2;SLA2	0.000420813	0.435305229
cg24211388	AIF1;AIF1;AIF1	0.000420813	0.435288339
cg06069407	TMCO3	0.000420813	0.435116019
cg10357682	C20orf27	0.002321262	0.435045897
cg08888553	C16orf91	0.000420813	0.434999178
cg23535322		0.001777515	0.434838564
cg02928840	PLXNA2	0.001038267	0.433915719
cg16596052	RGS10;RGS10	0.003185744	0.433835908
cg15030712	CHN2	0.004405484	0.433772739
cg04898039	MAD1L1;MAD1L1;MAD1L1	0.000420813	0.43326085
cg18568335	PACS2;PACS2	0.002321262	0.43322124
cg03278564		0.004405484	0.433158504
cg07391141		0.000420813	0.432994864
cg06963709		0.001038267	0.432758912
cg24397007	FOSL2	0.000420813	0.432475608
cg21210041	MYO18A;MYO18A	0.000420813	0.432220853
cg09238666	TK2	0.000420813	0.43219353
cg10903916	ASAP2;ASAP2	0.000420813	0.432034311
cg25865542	CMTM7;CMTM7	0.000420813	0.432012434
cg15793258	TNFAIP8L1	0.000420813	0.431985672
cg09354692		0.001038267	0.431906122
cg09406856	LRIG1	0.000420813	0.431714391
cg12110801		0.000420813	0.431713087
cg09039672	ACVR1B;ACVR1B;ACVR1B	0.004405484	0.431433112
cg09417547		0.000420813	0.43130757
cg11845168	XPO4	0.000420813	0.431140671
cg02615735	LY6G5C	0.000420813	0.431116642
cg14919082		0.001038267	0.431114212
cg02763540	UHRF1;UHRF1	0.000420813	0.431037176
cg07669182	FYB;FYB	0.000420813	0.431009571
cg26214026	ATXN2	0.004405484	0.430948556
cg05512561		0.000420813	0.430855706
cg21875980	EGLN1	0.007567702	0.43055075
cg08560387	TSPAN14;TSPAN14	0.000420813	0.430225341
cg23839180	FAM49A	0.002321262	0.430024948
cg08237971		0.002321262	0.430021771
cg04522498	TRAPPC9;TRAPPC9	0.000420813	0.429828815
cg10054332	PVT1	0.000420813	0.429722597
cg00707814	KDM2B;KDM2B	0.001038267	0.42970657
cg24448870	CHCHD6	0.000420813	0.429693109
cg06079564	HIPK2;HIPK2	0.001038267	0.429654504
cg11118440		0.001569481	0.42962297
cg07266910	ZMAT3;ZMAT3	0.001038267	0.429504454
cg08550517		0.000420813	0.42943135
cg16386293	ECM1;ECM1	0.000420813	0.429411614
cg12449049	CMAH	0.000420813	0.429314887
cg15889594		0.000420813	0.429241001

cg24092939	ADCY6	0.000420813	0.429094982
cg18242288		0.000420813	0.429084307
cg14277677	HIP1R	0.000420813	0.428619682
cg00449738		0.000420813	0.42856917
cg23590660	FOXJ2	0.005790932	0.428473547
cg02125365		0.002321262	0.4282509
cg17980999	PNLDC1	0.000420813	0.428183934
cg27449572	LOXL4	0.002321262	0.4281737
cg05897963	PLEKHF1	0.000420813	0.428092449
cg06573096		0.000466745	0.427845843
cg22006208	SEZ6;SEZ6	0.004405484	0.427779914
cg02135427		0.000636165	0.427636518
cg16306078		0.002321262	0.427632387
cg13746813		0.001038267	0.427490861
cg16229875	CEBPE	0.002321262	0.427414637
cg17571266	ZNF385A;ZNF385A;ZNF385A	0.000420813	0.427369818
cg14989714		0.003185744	0.42736796
cg24742520	UBR4	0.00961166	0.427341565
cg14541011	RALGDS;RALGDS	0.000420813	0.427336233
cg00556100		0.002321262	0.42718181
cg04100124		0.000636165	0.427166836
cg09017001		0.000420813	0.427072174
cg03072035		0.000420813	0.426824885
cg00287012	TTC39C;TTC39C;TTC39C	0.000420813	0.426635084
cg10864794	ARSB;ARSB	0.000420813	0.426560462
cg23342367	TPD52;TPD52	0.002321262	0.426422059
cg25681902		0.000420813	0.426363022
cg00211115		0.000420813	0.426229277
cg17200850	HRH2;HRH2	0.000420813	0.426083165
cg08285589	RAG1	0.000420813	0.425733492
cg21054703	ALOX5AP	0.000420813	0.425662085
cg11898646	EBF1	0.000420813	0.425531877
cg02311864	TNFRSF21	0.000420813	0.425518626
cg02482003		0.000636165	0.425511671
cg10691395		0.003185744	0.425464418
cg26633373	MYADM;MYADM;MYADM;MYADM;MYADM	0.001569481	0.425345162
cg10123514	HLA-DMB	0.002321262	0.425263083
cg13683301		0.000420813	0.425180352
cg07795968	JPH2;JPH2	0.002321262	0.425093848
cg01113988		0.001038267	0.424885771
cg02734505	ZNF385A;ZNF385A;ZNF385A	0.000420813	0.424839491
cg15659527	TTC7A	0.000420813	0.424833738
cg23558116		0.000636165	0.424817682
cg00323915	GIMAP4	0.000636165	0.424651754
cg18445438		0.000420813	0.424414524
cg09849688	ARID1A;ARID1A	0.003185744	0.42439067
cg08024038	RAI1	0.000420813	0.424252373
cg22123711	TNFRSF8;TNFRSF8	0.000420813	0.424250518
cg15826519	CAPZB	0.002321262	0.424051717
cg03003394	KLF7	0.000420813	0.423842733
cg18305855	C7orf60	0.001038267	0.423617228
cg22891595		0.000420813	0.423584675
cg24982541	CAST;CAST;CAST;CAST;CAST;CAST;CAST;CAST	0.005790932	0.423504967
cg05705140		0.000420813	0.42340749
cg03134661	UHRF1;UHRF1	0.001038267	0.423356471
cg24879415	CYGB;PRCD	0.000420813	0.423123999
cg24760467	LZTS2	0.000420813	0.423091949
cg18790741	NUP210	0.005790932	0.423070408
cg10296867	GAS7	0.000420813	0.422999769

cg12781678		0.000420813	0.422855178
cg04496824	MTF1	0.000420813	0.422833525
cg17852326	HLX	0.000466745	0.42274208
cg04191427	RPTOR;RPTOR	0.000636165	0.422741926
cg12122241	SIRPA;SIRPA;SIRPA	0.000420813	0.422728124
cg15361215	FAM45B;FAM45A	0.000420813	0.422408402
cg18797923	AMT;AMT;AMT;NICN1;AMT;AMT	0.000420813	0.422191436
cg20318272	PODXL;PODXL	0.000420813	0.422097592
cg16545743	IL3	0.000420813	0.422091581
cg16565031		0.002321262	0.422063622
cg22612590	TMCO3	0.001038267	0.42141117
cg00543769		0.000420813	0.421243134
cg10330187	ZC3H18	0.000420813	0.421177338
cg07570498	PHC2;PHC2	0.000420813	0.421132739
cg05163330	ADPRHL1;ADPRHL1	0.001038267	0.421038645
cg09142405		0.000420813	0.420917803
cg10996816		0.000420813	0.420809027
cg06677021	SERINC5	0.001569481	0.420783339
cg23101885	RAD51AP2	0.000420813	0.420590028
cg26904702	SLC27A3	0.000420813	0.419941863
cg22690339	BTBD9;BTBD9;BTBD9	0.000420813	0.419853074
cg11973132	ADRBK1	0.000420813	0.419791755
cg22222999		0.000420813	0.419757665
cg08498833	TG	0.000636165	0.419717227
cg02299020		0.000420813	0.41967975
cg07078269	PLXND1	0.000420813	0.41961536
cg08721026		0.000420813	0.419399896
cg04407063	ADRBK1	0.002321262	0.419265494
cg03075156	PRKCE	0.000420813	0.419261216
cg06806158	RPS6KA2;RPS6KA2	0.000420813	0.419237933
cg24135923		0.000420813	0.419178695
cg25600951		0.000420813	0.419035382
cg08994763	C3orf21	0.000420813	0.418888348
cg19863456	GPR157	0.000636165	0.418879778
cg10927461		0.000420813	0.418516307
cg14200572	KIF13A;KIF13A;KIF13A;KIF13A	0.003185744	0.4183017
cg14434109	BRD3	0.000420813	0.418177022
cg04398180	ADPRHL1;ADPRHL1;ADPRHL1	0.000420813	0.418098606
cg02889001	CLCN7;CLCN7	0.002321262	0.418007355
cg20951650	IRS1	0.000420813	0.417976197
cg04930596	NCOR2;NCOR2	0.000420813	0.417935645
cg15602677	SNTG2	0.000420813	0.417875357
cg24007886	OBFC2B;SLC39A5	0.001569481	0.41784941
cg24163263		0.004405484	0.417637836
cg16279861		0.007567702	0.417497947
cg04217515	ITGB2;ITGB2	0.001569481	0.417293459
cg04316353		0.001569481	0.417177125
cg24630825	GPM6A;GPM6A	0.000420813	0.417005451
cg12385032	BCAS3;BCAS3	0.000636165	0.41697438
cg03554394		0.004405484	0.416586081
cg19759478	C1orf183	0.000420813	0.416558217
cg13074203	SNTB1	0.005790932	0.416519303
cg14114297		0.002321262	0.416506108
cg09139509	RPTOR;RPTOR	0.000636165	0.416280905
cg26394055	FCER1G	0.002321262	0.416158613
cg20893838	RREB1;RREB1;RREB1;RREB1	0.000420813	0.416124725
cg27025738	SIPA1L1	0.000420813	0.416047834
cg01142676	FXYD2;FXYD2;FXYD2	0.001569481	0.415818482
cg15818109	COL11A2;COL11A2;COL11A2	0.000420813	0.41572791

cg20382047	C6orf25;C6orf25;C6orf25;C6orf25;C6orf25;C6orf25;	0.000420813	0.415707017
cg24405716	MTMR10	0.000420813	0.415466032
cg00644351	RGS10;RGS10	0.004405484	0.415465659
cg16770832	PELI2	0.000420813	0.415436173
cg07993367	ATP6V0E2;LOC401431;ATP6V0E2	0.000420813	0.415118313
cg18185648	ABCA4	0.000420813	0.41505417
cg17717972	ARHGEF10	0.000420813	0.415016229
cg00416882	PIAS2;PIAS2	0.000420813	0.415006903
cg19948549	D4S234E;D4S234E	0.000420813	0.414960252
cg09274091	LTB4R;LTB4R;CIDEB	0.000466745	0.414896948
cg22147446	SLC23A2;SLC23A2	0.000420813	0.414806128
cg03029090	LIPC	0.000420813	0.414804948
cg13492133	SMG7;SMG7;SMG7	0.000420813	0.414697677
cg21109666	DISC1;DISC1;DISC1;DISC1;DISC1;TSNAX-DISC1;	0.005790932	0.414629372
cg03463948	UBASH3B	0.003185744	0.414548834
cg03625953	SMG6;SMG6	0.000420813	0.414504549
cg16584662	PML;PML;PML;PML;PML;PML;PML;PML;PML	0.001569481	0.414374241
cg21515305	ELFN2	0.001038267	0.414299042
cg04008438		0.000420813	0.414292021
cg21899461	STK39	0.000636165	0.414201129
cg17434577	RPTOR;RPTOR	0.000420813	0.413982058
cg27226125	ALG1L2	0.000420813	0.41367683
cg08454507	RPTOR;RPTOR	0.000420813	0.413555607
cg27457201	RPTOR;RPTOR	0.000420813	0.413548603
cg05124117		0.001569481	0.413507298
cg15891546	DENND3	0.000420813	0.413418081
cg26407841		0.002321262	0.413365351
cg01213231	ITGA5	0.000420813	0.41327048
cg21606953	NRM	0.004405484	0.413183223
cg15686615		0.000420813	0.413091283
cg20176098		0.001038267	0.412799423
cg07197230	CECR2	0.005790932	0.412764907
cg12338690	POM121	0.000420813	0.412735459
cg07620928		0.000636165	0.412610386
cg19714347	TRIM26	0.000636165	0.412591638
cg01256139	RBMS1;RBMS1	0.00961166	0.412565459
cg26567012	GLRA1;GLRA1	0.002321262	0.412564051
cg13623749	JARID2	0.000636165	0.412503196
cg24519108	ANGPT2;ANGPT2;ANGPT2;MCPH1	0.002321262	0.412363768
cg26207766		0.001569481	0.412313411
cg02332073	TSGA13	0.000420813	0.412300222
cg25709789	TAOK1	0.000636165	0.412267604
cg14943796	BAHCC1	0.001569481	0.412256086
cg12970757	SLC2A14	0.000420813	0.41195433
cg15906007	DDX17;DDX17	0.004405484	0.411918324
cg05591126		0.000420813	0.411817793
cg06622199	SEPT9;SEPT9;SEPT9;SEPT9;SEPT9	0.000420813	0.411706976
cg10658307		0.001569481	0.411685422
cg27566842	ADARB2	0.000420813	0.411597334
cg00271311	CNTF;ZFP91-CNTF	0.000420813	0.411559226
cg20748242	RNF44	0.000420813	0.411484462
cg02873783		0.001569481	0.411400516
cg09183124		0.000636165	0.411273732
cg00638210	PLXNA1	0.002321262	0.411223356
cg13499923		0.000420813	0.411193356
cg19513987	MAD1L1;MAD1L1;MAD1L1	0.000420813	0.411142541
cg07091529	FAM49A	0.000420813	0.411007262
cg13519902		0.000420813	0.410957212
cg25124300	XDH	0.007567702	0.410888504

cg13242871	PREX1	0.000420813	0.410800013
cg16564828	MCF2L;MCF2L	0.002321262	0.410696488
cg00520380	LIMK2;LIMK2;LIMK2	0.000466745	0.410580561
cg00391031	KIAA1274	0.000420813	0.41056809
cg07880727	C7orf69;PKD1L1	0.000420813	0.410448228
cg22091236	RPTOR;RPTOR	0.000420813	0.41041799
cg14495953	LOC728606	0.000420813	0.410290241
cg15225325	PDLIM7;PDLIM7;PDLIM7	0.000420813	0.410285426
cg20628942	TMCO3	0.001038267	0.410257044
cg15091747	RUNX1	0.000420813	0.410241102
cg06595162	NCRNA00114;NCRNA00114	0.000420813	0.410230768
cg19478079		0.000420813	0.410106681
cg02043791	FGFR1;FGFR1;FGFR1;FGFR1;FGFR1;FGFR1;FGF	0.001569481	0.410052648
cg18308031		0.000420813	0.409797047
cg05626616	SEPT9;SEPT9;SEPT9;SEPT9;SEPT9;SEPT9;SEPT	0.000420813	0.409666837
cg22386492	KIAA1026;KIAA1026	0.000636165	0.409614926
cg06448249	CD79A;CD79A	0.000420813	0.409386568
cg15354065	DGKA;DGKA;DGKA;DGKA	0.005790932	0.409361523
cg25153741	COL23A1	0.004405484	0.409353415
cg08051604	MEG3;MEG3;MEG3	0.002321262	0.409035137
cg03990819		0.002321262	0.408985097
cg26443127		0.007567702	0.408932374
cg13104185	RERE;RERE	0.002321262	0.408928295
cg13536447		0.002321262	0.408894302
cg08203192	PRKCE	0.000420813	0.408828895
cg09152136		0.003185744	0.40881568
cg00255699	TRIT1	0.005790932	0.408644247
cg24897320	CYB561D1;CYB561D1;CYB561D1;CYB561D1;CYB	0.001038267	0.40861638
cg13745692	FHIT;FHIT	0.001038267	0.408575243
cg02855207	RELT;RELT	0.000420813	0.408371036
cg14294646	FAM125B	0.000420813	0.408143384
cg05226180	KCNMA1;KCNMA1;KCNMA1;KCNMA1	0.000636165	0.407964006
cg01012242	SLC37A3;SLC37A3	0.000420813	0.407920482
cg03437540	PRR5;PRR5-ARHGAP8;PRR5;PRR5;PRR5;PRR5	0.000420813	0.407904798
cg03475687	TBCD	0.003185744	0.407783842
cg10451425	ARMC5;ARMC5	0.000420813	0.407639933
cg18173184		0.000420813	0.407617286
cg21680729	PEMT;PEMT;PEMT	0.000719053	0.407550632
cg03325407	BCL2L15	0.000420813	0.407520891
cg23714707	PEMT;PEMT;PEMT	0.000420813	0.407408847
cg23465650	EBF1	0.000420813	0.407246504
cg27441486	NAV1;NAV1	0.000420813	0.407095446
cg03471346	ABL2;ABL2;ABL2;ABL2;ABL2;ABL2;ABL2;ABL2;AB	0.001038267	0.407061297
cg18165707	DENND3	0.000420813	0.406952964
cg24561419		0.007567702	0.406884323
cg23925650		0.000420813	0.406815783
cg14907310		0.001569481	0.406723965
cg07537699	LY6G5C	0.000420813	0.406565157
cg08655953	PLCH2	0.000420813	0.406462152
cg16661761	LOXL2	0.000420813	0.406449852
cg14594044	TBC1D14;TBC1D14	0.000420813	0.406397968
cg12182708	MMS19	0.000420813	0.406308579
cg23189692	EIF4G1;EIF4G1;EIF4G1;EIF4G1;EIF4G1	0.00961166	0.406142364
cg06285909	TMCO7	0.000420813	0.405961119
cg18741439	CSGALNACT1;CSGALNACT1;CSGALNACT1	0.000420813	0.405871488
cg25510823		0.001038267	0.405865177
cg10111352	ABCC1;ABCC1;ABCC1;ABCC1;ABCC1	0.000420813	0.405818733
cg20072792	RECQL5;LOC643008;LOC643008	0.004405484	0.405805996
cg02561343	UST	0.000420813	0.405582599

cg04006133	HSDL1;HSDL1	0.000420813	0.405563064
cg01578442		0.000420813	0.405543844
cg06380320	SEPT9;SEPT9;SEPT9;SEPT9;SEPT9	0.000420813	0.405454292
cg00629382	MSRA;MSRA;MSRA	0.000420813	0.405403523
cg17192381	BCL2	0.000420813	0.405403062
cg14267222		0.000420813	0.405403054
cg22946147	ZNF804B;MGC26647	0.000420813	0.405320492
cg01513063	SEPT9;SEPT9;SEPT9;SEPT9;SEPT9;SEPT9;SEPT	0.000420813	0.405117436
cg05379597	CAMK2G;CAMK2G;CAMK2G;CAMK2G;CAMK2G	0.000420813	0.405042906
cg02420027		0.000420813	0.404863876
cg26062560	C3orf21	0.000420813	0.404844669
cg05894462	PTPRN2;PTPRN2;PTPRN2	0.000420813	0.404716764
cg02729344		0.000420813	0.404697251
cg27579771	ARHGAP26;ARHGAP26	0.00961166	0.404621601
cg11937033		0.000420813	0.404620426
cg25839744	FAM65C	0.000420813	0.404601872
cg21328082	TREM1	0.007567702	0.404580812
cg25888881	ZNF438;ZNF438;ZNF438;ZNF438;ZNF438;ZNF438	0.000420813	0.404480659
cg11846618	BRF1	0.000420813	0.404417526
cg19997662	CHSY1	0.000420813	0.404301891
cg01883989	KLF7	0.000420813	0.404081152
cg18447740	OSCAR;OSCAR;OSCAR;OSCAR	0.000420813	0.404016012
cg20923885	SEPT9;SEPT9;SEPT9;SEPT9;SEPT9;SEPT9;SEPT	0.000420813	0.403941813
cg25223634	C10orf26;C10orf26	0.000420813	0.403666183
cg05488778	ARHGEF10	0.000420813	0.403592002
cg24864663	TGM5;TGM5	0.000420813	0.403420972
cg20539175	TMCO3	0.000420813	0.403409936
cg03255417		0.002321262	0.403377083
cg26421805		0.001569481	0.403372826
cg05050341	ENG;ENG	0.000420813	0.40335188
cg23140706	NFE2;NFE2	0.001569481	0.403276228
cg14039779	DUSP3;C17orf105	0.002321262	0.403240954
cg05850884	TRIM26	0.007567702	0.403181146
cg03965138	SIGLEC5	0.000420813	0.403151271
cg02027561	NCOA7;NCOA7	0.000420813	0.403123119
cg00329157	GCET2;GCET2	0.000420813	0.40292056
cg16725974	SYNE2;SYNE2	0.004405484	0.402743293
cg06439889		0.000420813	0.402488845
cg06411447	ANKRD11	0.000420813	0.402325174
cg18464903	IGF2BP2;IGF2BP2	0.001038267	0.402247074
cg09742346	CMIP;CMIP	0.000420813	0.402063778
cg01937932	ITGAX	0.000420813	0.401980428
cg17353680	KCNK12	0.002321262	0.40176163
cg05347794	FOXN3;FOXN3	0.000420813	0.401687302
cg21617903	VGLL4;VGLL4;VGLL4;VGLL4	0.000420813	0.401625069
cg16131053		0.000420813	0.401562809
cg22450342	MR1	0.000420813	0.401551262
cg18393175		0.003185744	0.401536417
cg07464408		0.004405484	0.401414297
cg04406620	CYB5B	0.007567702	0.401412022
cg05727149	CFDP1	0.002321262	0.401349037
cg21679402	THAP4	0.004405484	0.401326733
cg16569650	GNA12	0.000420813	0.401277372
cg07267067	SOAT2	0.000420813	0.401228738
cg06547715	CXCR2;CXCR2;CXCR2	0.002321262	0.401083738
cg22020227	OLFML3	0.000420813	0.401006094
cg20145598	SGTA	0.002321262	0.400853298
cg08810397	CLCN7;CLCN7	0.001569481	0.400759109
cg20580833	CRTAC1	0.000420813	0.400709449

cg26797124		0.001038267	0.400694679
cg08876518	CMIP;CMIP	0.004405484	0.400636454
cg19226017	FKBP5;LOC285847	0.000420813	0.40062724
cg10351692	POM121C	0.001777515	0.400468786
cg01981181	LMBR1L	0.000420813	0.400330203
cg09526022	NRXN2;NRXN2	0.000420813	0.400233247
cg25963412	ACOX3;ACOX3	0.002321262	0.400175484
cg23007087	AP3S2;AP3S2	0.000420813	0.400117503
cg15440661		0.000420813	0.399926144
cg18847118		0.000420813	0.399650947
cg01882471		0.005790932	0.399622321
cg21027282	RAD51L1;RAD51L1;RAD51L1	0.001038267	0.399569112
cg19605623	SCHIP1	0.000420813	0.399510554
cg08444833	EIF2C2;EIF2C2	0.001569481	0.399383766
cg19095143	TEX264;TEX264;TEX264	0.000420813	0.399330086
cg17648076		0.000420813	0.399282745
cg06083200	PPP1R11	0.000420813	0.399257344
cg10370574	REXO1	0.004405484	0.399235881
cg26475911	KCTD2	0.000420813	0.399200408
cg13702949		0.000636165	0.399194604
cg20636248		0.000420813	0.399191076
cg17758673	RGS12;RGS12	0.001569481	0.399147657
cg15416352	MNT	0.000420813	0.39903744
cg21323630	PCBP4;PCBP4;PCBP4	0.000420813	0.39886007
cg11360546	C7orf50;C7orf50;C7orf50	0.000420813	0.398848827
cg22586996	EBF1	0.001038267	0.398733975
cg25597952	MAD1L1;MAD1L1;MAD1L1	0.000420813	0.398670033
cg00735329	MBP;MBP	0.000420813	0.398655684
cg02845870		0.000420813	0.398651715
cg04581293		0.000420813	0.398607051
cg04781916	MSH6	0.000636165	0.398569549
cg22866998	DIP2C	0.000420813	0.398452871
cg04854098	TSPAN5	0.000420813	0.398375144
cg01433914	ADD3;ADD3;ADD3	0.000420813	0.398354544
cg06747087	MIR595;PTPRN2;PTPRN2;PTPRN2	0.000420813	0.398099581
cg09634212	HDAC4	0.001569481	0.398030125
cg14207267	IQCD	0.00961166	0.397905313
cg14071925	LILRA1	0.000420813	0.397879174
cg23751110		0.001569481	0.397793185
cg14724265	PPEF2;PPEF2	0.000420813	0.397665031
cg18641463	MAD1L1;MAD1L1;MAD1L1	0.004405484	0.397620976
cg09802818	LOC283404	0.005790932	0.39756268
cg15123742	CSGALNACT1;CSGALNACT1;CSGALNACT1	0.000420813	0.397371853
cg15888569	CPEB2;CPEB2	0.000420813	0.397300491
cg19668724		0.000420813	0.3972923
cg23804842	ZNF705A;ZNF705A	0.000420813	0.397255212
cg22698744	RUNX1	0.000420813	0.397248237
cg20426042	PGCP	0.002321262	0.397143182
cg13810695		0.000420813	0.39706181
cg14773365	HRH2;HRH2	0.000420813	0.396972297
cg19020666		0.001038267	0.396940932
cg08832745	FBXL13;FBXL13	0.001038267	0.396887232
cg08529825	HIPK1;HIPK1	0.001569481	0.396835124
cg15673994	TNRC4	0.000420813	0.396818004
cg01116477		0.001038267	0.396607336
cg23312431	TRPM2	0.000420813	0.396554581
cg23715732	RPTOR;RPTOR	0.000420813	0.396503828
cg15572396	PXK	0.001038267	0.396490586
cg17855390		0.000636165	0.396312387

cg06372223	SLC7A5	0.000420813	0.396151237
cg24228231		0.000420813	0.396131339
cg05590818	TLK1;TLK1	0.000420813	0.396053906
cg24674269		0.004405484	0.396044355
cg03845363	TOM1L2;TOM1L2	0.000420813	0.396001324
cg21571160	C4BPB;C4BPB;C4BPB;C4BPB;C4BPB	0.002321262	0.395795193
cg13497089	SCD5;SCD5	0.002321262	0.39578015
cg15128555	IGFBP2	0.000420813	0.39555505
cg19742736	TBC1D20	0.000420813	0.395497966
cg21582611		0.000420813	0.39545175
cg05006384	C14orf49	0.000420813	0.395288444
cg05951776	PEX14	0.000420813	0.39521085
cg14205216		0.000420813	0.395082146
cg25389087	MBP;MBP	0.000420813	0.394948886
cg20387392	ZBTB20;ZBTB20;ZBTB20;ZBTB20	0.003185744	0.394915937
cg17195635		0.001569481	0.394909066
cg03425860		0.001569481	0.394881182
cg05583921	ZBTB47	0.001569481	0.394864714
cg04384112	CFDP1	0.000636165	0.394845257
cg26419676		0.000636165	0.394810376
cg25422678	BRE;BRE;BRE;BRE;BRE	0.000420813	0.394721284
cg12125772		0.001038267	0.394712208
cg00899659	C10orf25;ZNF22	0.000420813	0.394600112
cg25256141	CUX1;CUX1;CUX1	0.003185744	0.394562432
cg20150640	C1orf213;C1orf213;C1orf213;ZNF436	0.001038267	0.394472392
cg01976921		0.000420813	0.394448785
cg13601957		0.000420813	0.394402326
cg08772206	SH2D4B;SH2D4B	0.000420813	0.394377112
cg27585822	FAM65C	0.000420813	0.394345615
cg17823346	ZMIZ1	0.000420813	0.394292783
cg23079727	VGLL4;ATG7;VGLL4;ATG7;VGLL4;ATG7;VGLL4	0.000420813	0.394275436
cg17065712	ZMIZ1	0.001038267	0.394264092
cg07202353	EHD4	0.000420813	0.394092822
cg14083146	RALGAPA2	0.005790932	0.393987318
cg27301488		0.001569481	0.393965359
cg18151635		0.000636165	0.393793895
cg24554439	TMCO3	0.000420813	0.393624588
cg24444059	LOC158376	0.000420813	0.393563647
cg27531842		0.000420813	0.393541028
cg02999224	SLC7A7;SLC7A7;SLC7A7;SLC7A7	0.007567702	0.393473227
cg05261759	TOR2A;TOR2A;TOR2A;TOR2A	0.000420813	0.39341069
cg24903893	EBF1	0.002321262	0.39339906
cg07428959	PHF17;PHF17	0.000636165	0.393344417
cg05845831		0.000636165	0.393249129
cg12150931	ZNF385A;ZNF385A	0.000420813	0.393175149
cg17341174	BAIAP2L1	0.001569481	0.393036
cg20216752	ALOX12	0.000636165	0.392998828
cg24143611	SNORD23;GLTSCR2	0.000636165	0.392969621
cg12201988	RIN2	0.007567702	0.392899964
cg15034267	GTPBP1	0.000420813	0.392805298
cg14223485	C9orf25	0.000420813	0.392594753
cg08684511	COL23A1	0.000636165	0.392582693
cg05606799	KISS1	0.000420813	0.392565696
cg25083732	S100P	0.004405484	0.392391416
cg07127883		0.001569481	0.392366116
cg10422067	TP53I11;TP53I11	0.000420813	0.392360883
cg16471612		0.000420813	0.392147891
cg02713162	SPRY4;SPRY4	0.000636165	0.392107016
cg13853953	THAP4;THAP4	0.000420813	0.392013383

cg25364972		0.003185744	0.391960786
cg00456343		0.000420813	0.391943904
cg00378158	LARGE;LARGE	0.000420813	0.391924906
cg00395296	NDUFA6	0.000420813	0.391828107
cg00810971		0.004405484	0.391697668
cg03417473	MOV10;MOV10	0.000420813	0.391687003
cg27162435	KCNAB3	0.005790932	0.391649694
cg23351327	PRKAG2;PRKAG2	0.000420813	0.391468213
cg25202370	HIVEP3;HIVEP3	0.000420813	0.391460924
cg04731810	HLTF;HLTF	0.000420813	0.391423309
cg23679982	SMYD4	0.000420813	0.391355026
cg08999812	TBCD	0.001569481	0.39128358
cg27047494		0.000420813	0.391233162
cg16657152		0.002321262	0.391006813
cg08252384	FAM153B	0.003185744	0.390910289
cg21271945	PRKCZ;PRKCZ;PRKCZ	0.002321262	0.390839284
cg25073093	NRXN2;NRXN2	0.000420813	0.390713202
cg05132782		0.000420813	0.390566729
cg15648345	MKS1;MKS1	0.002321262	0.390527161
cg07053162	PPL	0.000420813	0.390509382
cg08166362	C3orf37;C3orf37	0.004405484	0.39049309
cg14170787	AKAP2;PALM2-AKAP2;PALM2-AKAP2;AKAP2	0.000420813	0.390482416
cg26780819		0.000420813	0.39032714
cg11414202	NLK	0.007567702	0.390299962
cg01340991	SYNGAP1	0.000420813	0.390267703
cg07910529	MAD1L1;MAD1L1;MAD1L1	0.000420813	0.390237636
cg22799757	SCRN1;SCRN1;SCRN1;SCRN1	0.000420813	0.390197847
cg24607603	KIAA2018	0.000466745	0.389929271
cg26391080	SH2D4B	0.000420813	0.389928722
cg17519749	MYT1L	0.000420813	0.389881566
cg02771673	PCCA;PCCA	0.002321262	0.389851673
cg25649826	USP22	0.000420813	0.389797766
cg24694018	POLR3GL	0.000420813	0.389695648
cg16241062		0.001038267	0.389562809
cg13868473	MAD1L1;MAD1L1;MAD1L1	0.000420813	0.38949512
cg04870120		0.004405484	0.389424294
cg08305575		0.003185744	0.389352318
cg23048001	MAD1L1;MAD1L1;MAD1L1	0.001777515	0.389336182
cg20414082		0.001038267	0.389250647
cg21126943	CEACAM6	0.002321262	0.389188418
cg12067421		0.002321262	0.389184217
cg16785938	SLC2A9;SLC2A9	0.001569481	0.389023141
cg20892919		0.001038267	0.388917178
cg24733614	CHST11	0.000420813	0.388809664
cg10087651	FAM109A	0.000420813	0.388596729
cg05163268		0.000420813	0.388538241
cg08390994	APOL3;APOL3	0.000420813	0.38836535
cg00970435	ARSG	0.001038267	0.388308808
cg00110545		0.000420813	0.388301151
cg12886494	ARPP-21;ARPP-21;ARPP-21	0.007567702	0.388300955
cg02451443		0.001569481	0.388282239
cg26075639		0.000719053	0.38827696
cg19225688	CXCR2;CXCR2;CXCR2	0.004405484	0.388248224
cg16081096	IQSEC1;IQSEC1	0.000420813	0.388202874
cg04005793	OBFC2B;SLC39A5	0.005790932	0.38818689
cg18352516		0.000420813	0.388182784
cg25738350		0.000420813	0.388146678
cg04502852		0.000420813	0.388113648
cg07651540	LOC728606	0.002321262	0.388104574

cg10108888		0.000420813	0.388092172
cg13985446		0.001038267	0.387663628
cg19796955	TBK1	0.001038267	0.387649635
cg17588094		0.001569481	0.387631001
cg13354414	ZNF804B;MGC26647	0.000420813	0.387624586
cg10395101	ANGPT2;ANGPT2;ANGPT2;MCPH1	0.001038267	0.387567271
cg08925046	PDLIM1	0.004229594	0.387534584
cg00008671	FAM190B	0.003185744	0.387489648
cg04159006		0.000420813	0.387480353
cg10601159	THAP4;THAP4	0.001569481	0.387417459
cg08115618		0.000420813	0.387401287
cg14608550	SEPT9;SEPT9;SEPT9;SEPT9;SEPT9	0.000420813	0.387365821
cg25863735	ADAL	0.001038267	0.387353568
cg15833565	ERI3	0.002321262	0.387304041
cg05955436	RRBP1;RRBP1	0.000420813	0.387278919
cg08351131	HEXIM2	0.000420813	0.387241347
cg03818307	ECM1;ECM1;ECM1;ECM1	0.000420813	0.38710728
cg20486551		0.000420813	0.387090416
cg21836905		0.000420813	0.386995062
cg07187268	C22orf15	0.000420813	0.386944707
cg16462433		0.000636165	0.386883078
cg00110171	NFATC1;NFATC1;NFATC1;NFATC1;NFATC1	0.000636165	0.386693942
cg02547426	RGS12;RGS12;RGS12	0.000420813	0.386604108
cg00748432	SLC25A18	0.000420813	0.386566116
cg12551813	CAMSAP1L1	0.000420813	0.386475015
cg26306329		0.000420813	0.386282082
cg23716690	KIF1B;KIF1B	0.000636165	0.386250307
cg07340007	STK24;STK24	0.002321262	0.38623244
cg27507284	NFE2L2;NFE2L2;NFE2L2	0.002321262	0.386229213
cg05035470		0.000636165	0.386208674
cg17169037	KDM2B;KDM2B	0.000420813	0.386155124
cg03554335	C17orf72	0.000420813	0.38614984
cg09172244	EBF1	0.000636165	0.386098259
cg27405731	CUX1;CUX1;CUX1	0.000636165	0.386037596
cg01824284	BAHCC1	0.000420813	0.386025878
cg00223136		0.000636165	0.385928028
cg07598021	GRPEL2	0.000420813	0.385887699
cg15636295	HBEGF	0.000420813	0.385811186
cg03602500	KIR3DX1	0.000636165	0.38579259
cg00566158	PTPRN2;PTPRN2;PTPRN2	0.000420813	0.385721474
cg15448894		0.007567702	0.385720813
cg07496207	ERGIC1	0.000420813	0.385717245
cg17250929	S100A5	0.000420813	0.385663502
cg08961793	SULT1A1	0.002321262	0.385663207
cg02329670	MIR497;MIR195	0.000420813	0.385613181
cg00343063	ZNF213;ZNF213	0.000420813	0.385595418
cg19334176	DIP2C	0.002321262	0.385569235
cg24307601		0.000420813	0.385391286
cg08840913	ATAD2B	0.000420813	0.385383804
cg11809958	FGD4	0.002321262	0.385375591
cg00967073		0.000636165	0.385359452
cg12656896		0.000420813	0.385351029
cg18477569	BACH2;BACH2	0.002321262	0.385305454
cg02532853	NCRNA00114;NCRNA00114	0.003185744	0.385266178
cg04363470	PPFIBP2	0.000420813	0.385261572
cg05338167	CALML4;CALML4;CALML4;CALML4	0.002321262	0.385182497
cg15207662	HRH2;HRH2	0.000420813	0.385176347
cg06205746	ADPRHL1;ADPRHL1	0.000420813	0.385125221
cg09926649	COMT;COMT;COMT	0.000466745	0.385123514

cg24339704	GNG7	0.001569481	0.385109158
cg04714478		0.000420813	0.385089016
cg08788930	DENND3	0.000636165	0.384966663
cg10673192	TRAPPC9;TRAPPC9	0.002321262	0.38495848
cg00686823	TPRA1	0.000420813	0.384928332
cg05027594	TAPT1	0.000636165	0.384832385
cg01883208	PCNXL2	0.000420813	0.384809802
cg09977718	RPTOR;RPTOR	0.002321262	0.384779062
cg27168573	PXN;PXN	0.000420813	0.384710088
cg07125981	CUL2	0.001777515	0.384618492
cg12559197	PDE8B;PDE8B;PDE8B;PDE8B;PDE8B	0.000420813	0.384587141
cg20738719	SEMA5B	0.000420813	0.384579189
cg05367967		0.002321262	0.384522603
cg01565508	PIK3R5	0.000420813	0.38428931
cg13679772	FOXN3	0.004405484	0.384281378
cg06601266	C10orf119	0.000420813	0.384273375
cg24162781		0.004405484	0.384270628
cg01192077	EBF1	0.007567702	0.384264068
cg04822495	PLXNC1	0.001569481	0.384184396
cg07052737		0.002321262	0.384057377
cg23554917	UBASH3B	0.000420813	0.384015397
cg10445988	MAD1L1;MAD1L1;MAD1L1	0.000420813	0.384009817
cg03585084	LHPP;LHPP	0.000420813	0.38392763
cg05991009	RFX8	0.002321262	0.383893231
cg06573088	CAP2	0.000420813	0.383842637
cg20097219	TBC1D16	0.001038267	0.383795688
cg07831553		0.000420813	0.383753051
cg03408354	SLC7A5	0.000420813	0.383748987
cg16664617	SLC27A1	0.000420813	0.383722263
cg16201957	MAML3	0.000420813	0.383661449
cg22912818	TMCO3	0.000420813	0.383617828
cg07164388	LTB4R2;LTB4R2;LTB4R2;LTB4R2;LTB4R;CIDEB;L	0.000420813	0.383613601
cg17803713	SH3RF3	0.000420813	0.383590552
cg12045443		0.000636165	0.383403204
cg19384448		0.002321262	0.383333806
cg07815238		0.001038267	0.383326868
cg10211062		0.000420813	0.383215088
cg07924575	HPS4;SRRD	0.005790932	0.383126716
cg22898924	ARRDC5	0.000420813	0.3830458
cg04681879	RPTOR;RPTOR	0.000420813	0.383010961
cg00920938	BMP1;BMP1;BMP1	0.001038267	0.38300216
cg24171555	AAK1	0.007567702	0.382936784
cg00920254	SLC29A1;SLC29A1;SLC29A1;SLC29A1;SLC29A1	0.000420813	0.382850478
cg14374994	PDCD1LG2	0.000636165	0.382783148
cg18642369	DOCK9;DOCK9	0.000420813	0.382762138
cg16560824	DENND3	0.000420813	0.382639276
cg03228516	CSNK1E;CSNK1E	0.000420813	0.382575122
cg17713161	C17orf28	0.000420813	0.382533125
cg06267718	ELFN2	0.000420813	0.382435521
cg16182375	ZMIZ1	0.000420813	0.382426717
cg18366956	SDK2	0.000420813	0.382332485
cg27470433	CACNA1C;CACNA1C;CACNA1C;CACNA1C;CACN	0.000420813	0.382274177
cg10920329		0.000420813	0.382186149
cg14364926	ZER1	0.000420813	0.382143479
cg22255288	RPTOR;RPTOR	0.000420813	0.382139102
cg08559364	VGLL4;VGLL4	0.000420813	0.382106728
cg14786790	FAM13A	0.000466745	0.382003721
cg10104487		0.000420813	0.381946782
cg05481243	BID;BID	0.000420813	0.381916641

cg13071069	IQSEC1;IQSEC1	0.000420813	0.381858414
cg15862380	DIP2C	0.001569481	0.381617685
cg19991046	DENND3	0.001038267	0.381524988
cg14276125	ADRBK1	0.002321262	0.381366213
cg05597945	BANP;BANP	0.000420813	0.381231621
cg04751089	IRS1	0.000420813	0.381185178
cg26103719	FBLN5	0.001569481	0.381163497
cg03400731	C17orf85;C17orf85	0.001569481	0.381095379
cg03556480	IGFBP2	0.000466745	0.381082308
cg15584954	DIP2C	0.000636165	0.381045293
cg03331229	MMP13	0.000420813	0.381040161
cg08219486	RPTOR;RPTOR	0.000420813	0.380917402
cg19212779	FRYL	0.000420813	0.380841179
cg15522953	ZMIZ1	0.000420813	0.380770907
cg02052797		0.000636165	0.380751514
cg14503564	LDB2;LDB2	0.007567702	0.380590446
cg22124221		0.000420813	0.380570195
cg03604774		0.000420813	0.380547075
cg19348484	FURIN	0.000420813	0.380538504
cg24517501	GFI1;GFI1	0.000420813	0.380531934
cg16580197		0.002321262	0.380530564
cg25644380	BCL3	0.000420813	0.380476741
cg23036852		0.001569481	0.380386697
cg02345886	GALNT3	0.007567702	0.380203412
cg24834394		0.002321262	0.380181496
cg00872683		0.000420813	0.379978987
cg01432609	RPTOR;RPTOR	0.000420813	0.379794894
cg15447017	LMNA;LMNA;LMNA	0.000420813	0.379785272
cg23260484	EFCAB2;EFCAB2;EFCAB2;EFCAB2;EFCAB2	0.000636165	0.379765323
cg14109456	NEK6;NEK6;NEK6;NEK6	0.000420813	0.379687784
cg14689122	PDIA5;PDIA5	0.000420813	0.379687493
cg13408655	COL15A1	0.000636165	0.379521052
cg02482730	SEPT9;SEPT9;SEPT9;SEPT9;SEPT9;SEPT9;SEPT	0.000420813	0.379451994
cg04406229		0.000420813	0.379430628
cg11601967	MAD1L1;MAD1L1;MAD1L1	0.000420813	0.379423774
cg03295274		0.005790932	0.379377357
cg08070491	SP2	0.001569481	0.379329535
cg26686009	C10orf25;ZNF22	0.000420813	0.379181389
cg15029935	CRTC3;CRTC3	0.003185744	0.37907813
cg22437221	FYB;FYB	0.001569481	0.379048249
cg26275848		0.000420813	0.379042221
cg19821988	ATP2A3;ATP2A3;ATP2A3;ATP2A3;ATP2A3;ATP2A	0.000420813	0.378658903
cg06576340	PILRB;PILRB;PILRB	0.000420813	0.378646676
cg20615117	SORL1	0.005790932	0.378566123
cg17491300	ARHGEF10	0.000420813	0.378564426
cg13549638	RPTOR;RPTOR	0.000420813	0.37848574
cg02410870	TRABD	0.000420813	0.378407821
cg10540679		0.000420813	0.378404026
cg12051956	C12orf53	0.000420813	0.378396726
cg07176064	HMGXB4;HMGXB4	0.001569481	0.378312901
cg26628751	B3GNTL1	0.001038267	0.378290206
cg01391548		0.000420813	0.378281177
cg11014810	SH3PXD2A	0.000420813	0.378224131
cg16977751	AP1B1;AP1B1;SNORD125;AP1B1	0.002321262	0.378145591
cg04855961		0.001569481	0.378084868
cg16230060	RNU5E;RNU5D;CKMT2;CKMT2;CKMT2	0.000420813	0.378029453
cg01364674		0.000420813	0.377989704
cg10950251		0.002321262	0.377950237
cg06825512	APCDD1	0.004405484	0.377883791

cg06366374	LOC285780	0.000420813	0.377877251
cg18761976	SV2B;SV2B	0.003185744	0.377875719
cg18176723	PPCDC	0.000420813	0.377729463
cg10717869	SLC41A1	0.000420813	0.377646909
cg19862242	TNFAIP3	0.002321262	0.37764115
cg21518332		0.000420813	0.377609513
cg21031917	KHDRBS2	0.002321262	0.377597811
cg01950511	TRAF3IP1;TRAF3IP1	0.001038267	0.3775944
cg07778180	CCL4L1;CCL4L2	0.002321262	0.377533476
cg10541534	SETD7	0.000420813	0.377529727
cg09686308	CIB3	0.000420813	0.377524451
cg16283183	LIMD1	0.000636165	0.377495937
cg19306970	HLX	0.000420813	0.377488829
cg19078067	SDK2	0.000420813	0.377440973
cg07337598	ANXA9	0.000636165	0.377427778
cg08324090	ACAP1	0.002321262	0.377300807
cg00925339	NT5E	0.000420813	0.377293454
cg12355794	SLC22A13	0.000636165	0.377254747
cg18303397	IFT122;IFT122;MBD4;IFT122;IFT122	0.001569481	0.37725076
cg22855764	TANC1;TANC1	0.000420813	0.377222612
cg22566142	LNPEP;LNPEP	0.000420813	0.377094534
cg17855595	ZNF692;ZNF692	0.000636165	0.376975598
cg17974145	CUX1;CUX1;CUX1	0.000420813	0.3769099
cg14873515	NDST1	0.000420813	0.376897916
cg12308346	MAML3	0.000420813	0.376877646
cg14231959	ARHGEF10	0.005790932	0.376840807
cg06821871	CBFA2T3;CBFA2T3	0.000420813	0.376824731
cg27196880	PTRF	0.002321262	0.376748455
cg13553662		0.000636165	0.376691286
cg04257752	C21orf29;KRTAP12-3	0.001569481	0.376673154
cg13474692	MGMT	0.002321262	0.376658776
cg00668519	VGLL4;ATG7;VGLL4;ATG7;VGLL4;ATG7;VGLL4	0.002321262	0.376529498
cg08511772	CDC42EP1	0.000420813	0.376428875
cg14827643	RGS12;RGS12	0.000420813	0.37636471
cg16215084	DDR1;DDR1	0.000636165	0.376221489
cg15765353		0.000420813	0.376159124
cg04044203	ESAM	0.000420813	0.376068519
cg13849419	TJP2;TJP2;TJP2;TJP2	0.000420813	0.376011096
cg06789500	MAD1L1;MAD1L1;MAD1L1	0.001038267	0.375947406
cg02322400	MAML2	0.000420813	0.375945086
cg12313149		0.003185744	0.375850161
cg01008894	RGS12;RGS12	0.000420813	0.37567717
cg10520790	PROM1;PROM1;PROM1;PROM1;PROM1;PROM1;	0.000420813	0.375645046
cg14918082	KCNAB3	0.007567702	0.375590203
cg10117599	PRKAR1B;PRKAR1B;PRKAR1B;PRKAR1B;PRKAR	0.005790932	0.375314033
cg08400424	ARHGAP26;ARHGAP26	0.000420813	0.375261466
cg23630423	RTN4RL1	0.000420813	0.375261353
cg17228105	ERG;ERG;ERG;ERG	0.000420813	0.375240867
cg18103836		0.000420813	0.37520498
cg11194613	CTTN;CTTN	0.004405484	0.375178428
cg01944288	NTNG2	0.000420813	0.375140818
cg04134235	FAM49A	0.001569481	0.375123138
cg13300580	SLC9A1	0.000420813	0.375084578
cg02057681	CHL1	0.000420813	0.375026159
cg02142900		0.000420813	0.37501572
cg18601596	KCNK16;KCNK16;KCNK17;KCNK16;KCNK17	0.001038267	0.375000685
cg26675785	ABCC1;ABCC1;ABCC1;ABCC1;ABCC1	0.000420813	0.374996779
cg08384322	C16orf46;C16orf46	0.002321262	0.374852106
cg08915438	FKBP5;LOC285847	0.001038267	0.374814406

cg23736297	RPTOR;RPTOR	0.000420813	0.374805692
cg27628849	PHF17;PHF17	0.000420813	0.374671582
cg13816466	LOC158376	0.000420813	0.374656129
cg10873171	AEBP1	0.000420813	0.37455963
cg26955132		0.001038267	0.374453737
cg11313780		0.000636165	0.374390222
cg10738119	YWHAG	0.002321262	0.374372089
cg07061368	FKBP5;FKBP5;FKBP5;FKBP5	0.007567702	0.37428597
cg00435173	RAB5C;RAB5C	0.002321262	0.374266563
cg09341580	GALR3	0.000636165	0.374248494
cg18747197		0.002321262	0.374209145
cg16596317		0.000636165	0.374205534
cg18181904	PCTP;PCTP	0.000420813	0.374179273
cg12076102	ICAM2;ICAM2;ICAM2;ICAM2;ICAM2	0.001569481	0.374153828
cg18045102	RFX8	0.000420813	0.374147669
cg22280671	EFHA1	0.003185744	0.374131251
cg20300911		0.000420813	0.373995698
cg17850088		0.002321262	0.373911018
cg02918093		0.000420813	0.373898153
cg03994959	LIG4;LIG4;LIG4;LIG4	0.005790932	0.373846446
cg19317600	ACOX3;ACOX3	0.000636165	0.373837912
cg03919657	ACSF2	0.001038267	0.373826559
cg03123320		0.001038267	0.373825752
cg19009305	ISG20	0.000420813	0.373787713
cg01466011		0.002321262	0.373750357
cg23606775	CLSTN1;CLSTN1	0.000420813	0.373706414
cg07698793		0.001038267	0.373600173
cg02692177		0.000420813	0.37353294
cg14707053	TAF8	0.004405484	0.373345329
cg05436845	MAP3K11	0.002321262	0.373289385
cg11706849	MGAT5	0.002321262	0.373270636
cg21078654	TMOD3	0.000420813	0.373145946
cg11095743		0.000420813	0.373074277
cg08763351	SPRR4	0.001569481	0.373061837
cg06312985	RP9P	0.005790932	0.373028523
cg16951074	BCL11A;BCL11A;BCL11A	0.005790932	0.372976475
cg18702935		0.000420813	0.372943153
cg13526642	AKAP2;PALM2-AKAP2;PALM2-AKAP2	0.000420813	0.372867557
cg18465945	PEMT;PEMT;PEMT	0.000636165	0.372735466
cg26334358		0.001569481	0.372520386
cg20595271	SH2B3	0.000420813	0.372509101
cg17404289	MAD1L1;MAD1L1;MAD1L1	0.007567702	0.372398186
cg09029902	ZNF217;ZNF217	0.000420813	0.372366605
cg23112464	TMCO3	0.000420813	0.372356747
cg04438064	HDAC4	0.000420813	0.372234416
cg19384997	CRYL1	0.005790932	0.372195562
cg26106921		0.000420813	0.372173135
cg06679089	TNRC6B	0.000420813	0.37216624
cg05141988	SCML4	0.000420813	0.372154756
cg22579224	KIAA1199	0.000420813	0.372036536
cg18101488		0.000420813	0.372023676
cg01981433	TNFAIP3	0.000420813	0.371997407
cg17238319	RFTN1	0.004405484	0.371995083
cg12221864	WDR43	0.004405484	0.371859472
cg17022488		0.002321262	0.371853455
cg02286857	TTC7A	0.000420813	0.371849788
cg26747517	ANKRD11	0.000420813	0.371849696
cg18564971	SMURF1;SMURF1	0.001038267	0.371819794
cg20029153	MYO18A;MYO18A	0.001038267	0.371801359

cg22521263		0.000420813	0.371705759
cg15137213	VPS53;VPS53	0.000420813	0.371641232
cg21157904	TRIM14;NANS	0.001038267	0.371627482
cg19835040	RTP3	0.000420813	0.37160289
cg22250556		0.002321262	0.371594001
cg22032626	NCKAP5L	0.002321262	0.371456218
cg15549075	PRKCE	0.000420813	0.371436872
cg01296705	HRH2;HRH2	0.000420813	0.371390802
cg04398156		0.002321262	0.371388011
cg11704201		0.00961166	0.371304133
cg15919833	CAST;CAST;CAST;CAST;CAST;CAST;CAST;CAST	0.000466745	0.371297561
cg24838825		0.002321262	0.371283251
cg07986378	ETV6	0.001569481	0.371259577
cg05180258	NUMB;NUMB;NUMB;NUMB	0.004405484	0.37122621
cg19445588		0.000636165	0.37114772
cg23223330		0.000420813	0.371052862
cg03796003	KCTD5	0.00961166	0.370963896
cg21130221	KCNQ1;KCNQ1	0.000420813	0.370959294
cg03004102	CD34;CD34	0.001569481	0.37083842
cg02854313	CSGALNACT1;CSGALNACT1;CSGALNACT1	0.000636165	0.370741527
cg00385956	NPHP4	0.000420813	0.370646337
cg14254562	TLE1	0.000420813	0.370621298
cg20341251	WNT5B;WNT5B	0.000420813	0.370553045
cg09595245	SLC12A5	0.000420813	0.370497694
cg15461663		0.001038267	0.370275639
cg10330057		0.000420813	0.37025611
cg13702370		0.000420813	0.370229657
cg16303353	CACNA1C;CACNA1C;CACNA1C;CACNA1C;CACN	0.000420813	0.370228646
cg07922513	KCNAB2;KCNAB2	0.001569481	0.370222858
cg08698943		0.005790932	0.370009121
cg00870269	LIPC	0.000420813	0.369799929
cg13049432	NCRNA00114;NCRNA00114	0.000420813	0.369776543
cg19678959	TUBA8	0.000420813	0.369762546
cg00569500	TNFRSF8	0.000636165	0.369754513
cg09408143	RAB11FIP3;RAB11FIP3	0.005790932	0.369675034
cg08484560	ATP11A;ATP11A	0.005790932	0.369665398
cg19408207	ZMAT3;ZMAT3	0.007567702	0.369576554
cg02458141	HS1BP3	0.000420813	0.369547974
cg03606511	BCL11A;BCL11A;BCL11A	0.000420813	0.369504241
cg21133433		0.000420813	0.369471416
cg15431659	C3orf21	0.001569481	0.369410868
cg12224388		0.001569481	0.369358164
cg22115465	MAD1L1;MAD1L1;MAD1L1	0.000636165	0.369316983
cg13308743		0.00961166	0.369288369
cg16586406	TPM3	0.000420813	0.369256418
cg02413938	ESPNL	0.000420813	0.36918991
cg21268658	PTPRF;PTPRF	0.002321262	0.369100012
cg14565014	PLEKHG6	0.000420813	0.369048859
cg17980404	IGFBP4	0.000420813	0.369039353
cg06634140		0.000420813	0.368918804
cg19755813	GEMIN4	0.000420813	0.368813996
cg25578476		0.000420813	0.368792225
cg08675117	RFX2;RFX2	0.000636165	0.368767339
cg10963218	S100A13;S100A13;S100A13;S100A1;S100A13;S10	0.001038267	0.368747285
cg27466999	LMF1	0.001569481	0.368720535
cg05575639	CHD7	0.004405484	0.368575811
cg20966270		0.001038267	0.368390262
cg03153115	GNG7	0.000420813	0.368272827
cg20702417	DNMT3A;DNMT3A;DNMT3A	0.000420813	0.368170672

cg18404811		0.002321262	0.368159692
cg11419575		0.000420813	0.368078202
cg20415945	HDAC4	0.002321262	0.368061789
cg15763670	C10orf105;C10orf105;CDH23	0.000420813	0.368021873
cg25343661	VEGFA;VEGFA;VEGFA;VEGFA;VEGFA;VEGFA;VE	0.000420813	0.367967652
cg05707116	EIF2AK3	0.000420813	0.367920131
cg20043649		0.007567702	0.367912864
cg11505026	PRKCE	0.000420813	0.367909166
cg02319094	P2RY6;P2RY6;P2RY6	0.000420813	0.367906285
cg07137429		0.000420813	0.367883947
cg05758467	HDAC4	0.002321262	0.367787863
cg07491702	CDAN1	0.007567702	0.367785549
cg01155092	TMEM100	0.002321262	0.367745763
cg16163535	SLC12A7	0.003185744	0.367677264
cg22222461	PGCP	0.000420813	0.367665086
cg02830936	MTMR9L	0.001038267	0.367608468
cg22117819		0.000420813	0.367608432
cg24917065	SLC25A37	0.000636165	0.367587807
cg23472153	MPRIP;MPRIP	0.000420813	0.367557491
cg18636739	CSDA;CSDA	0.002321262	0.367350343
cg05774614	RPTOR;RPTOR	0.000420813	0.367335897
cg15889847	PLEKHG3	0.001569481	0.36732444
cg02010760		0.000420813	0.367307218
cg05568549	CCND3;CCND3;CCND3;CCND3	0.000420813	0.367302597
cg13641778	TMEM110	0.000420813	0.367193065
cg24402667	ARHGAP23	0.002321262	0.367152358
cg14124646	HPS6	0.002321262	0.367145376
cg03162506		0.000420813	0.367140444
cg23893629	LAX1;LAX1	0.004405484	0.367113166
cg03291548		0.000420813	0.367087801
cg05248470	LILRB2;LILRB2	0.000420813	0.367078473
cg09086087	SLC12A4;SLC12A4;SLC12A4;SLC12A4;SLC12A4	0.000420813	0.367044409
cg03892714	SH3PXD2B	0.000420813	0.36702964
cg10186254	NRXN3;NRXN3;NRXN3	0.002629558	0.367026474
cg05788080	ITPRIP	0.005790932	0.367025948
cg21244580		0.000636165	0.366975809
cg21422361	MAD1L1;MAD1L1;MAD1L1	0.002321262	0.366889167
cg02167757	SIAH1;SIAH1	0.000420813	0.366856591
cg13524037	HLA-DMB	0.000420813	0.36665887
cg05903720		0.002321262	0.366654804
cg02087985	SNX27	0.001038267	0.366646309
cg13880868	GLE1;GLE1	0.000420813	0.366500338
cg21089050	GAS7	0.000420813	0.366295076
cg00153693		0.000420813	0.366276038
cg02981163		0.000420813	0.366272448
cg02744046	LIPC	0.000636165	0.366252563
cg00669330	C2orf85	0.001038267	0.366007161
cg20320472	KDM2A;KDM2A	0.000420813	0.365970579
cg17511604	ZHX2	0.000420813	0.365939556
cg02652361	MALAT1	0.000636165	0.365884662
cg11346718		0.000420813	0.365850571
cg11307565	PXN;PXN	0.002321262	0.365792891
cg11661914	ADCY6	0.001038267	0.365753212
cg02007534		0.002321262	0.365743681
cg04409030	SOCS2	0.000719053	0.365698848
cg08324801		0.000420813	0.3656802
cg10323962	CA5A	0.001038267	0.365589448
cg05874806	MAP4K4;MAP4K4;MAP4K4	0.000420813	0.365547417
cg03121834	PILRA;PILRA;PILRA	0.000420813	0.365494609

cg26914296	OIT3	0.000420813	0.365467809
cg08002791	GPR133	0.000420813	0.365378565
cg07349815		0.000420813	0.365376283
cg08628233		0.000420813	0.365323177
cg25379762	GAS7	0.000420813	0.36529301
cg22806934	UBASH3B	0.000420813	0.365287759
cg08859278	ZAP70	0.000420813	0.365273992
cg08371951	SYNGAP1	0.000420813	0.365215461
cg10614445	DNMT3A;DNMT3A;DNMT3A	0.000420813	0.365124577
cg11463380	RGS12;RGS12;RGS12	0.000420813	0.365116861
cg23695222		0.000420813	0.365069818
cg10196532	RCBTB1	0.000636165	0.364949032
cg23705979	CDK6;CDK6	0.000420813	0.364893385
cg15157945	NBEAL2	0.000420813	0.364820698
cg18741277	ZGLP1	0.000420813	0.364724261
cg14037250	BRD3	0.000636165	0.364720094
cg08061598	FYN;FYN	0.004405484	0.364715826
cg21487856	SPTBN1;SPTBN1	0.000420813	0.364707818
cg26027052		0.000420813	0.364634491
cg16704703		0.001569481	0.364573234
cg08937107		0.000420813	0.36455776
cg08553274	KIAA1530	0.001038267	0.364531264
cg16671238	CRYL1	0.000420813	0.364413515
cg15775674	CAPN3;CAPN3;CAPN3;CAPN3;CAPN3;CAPN3;CA	0.002321262	0.364403284
cg02217713	PRKAR1B;PRKAR1B;PRKAR1B;PRKAR1B;PRKAR	0.004405484	0.364244049
cg20909017	ITGA5	0.000420813	0.36421113
cg07366300	B3GNT8	0.002629558	0.364119091
cg06330289		0.000636165	0.36405211
cg02023973	CLCN7;CLCN7	0.002321262	0.364023941
cg22688566	MYO18A;MYO18A	0.000420813	0.364021747
cg10687006	FAM188B	0.000636165	0.363962545
cg06950683	CBFA2T3	0.000420813	0.36395665
cg10547908	FAM174B	0.000420813	0.363855006
cg18326562	MAP2K6	0.000420813	0.363715908
cg15731920	LASP1	0.000420813	0.363710645
cg05206789	AFF1	0.000420813	0.363623785
cg13525639	SMURF1;SMURF1	0.000420813	0.363543222
cg12807187	EIF4E3;EIF4E3;EIF4E3	0.002321262	0.363528054
cg09249101		0.000420813	0.363442684
cg17884698		0.000420813	0.363434358
cg01074955	NPHP4	0.005790932	0.363406321
cg10239022	GPR133	0.000420813	0.363331448
cg13577072	KCNQ1;KCNQ1OT1;KCNQ1	0.000420813	0.36332687
cg17679427	CREM;CREM;CREM;CREM;CREM;CREM;CREM;C	0.000420813	0.363319652
cg21122199	C17orf64	0.000420813	0.363274595
cg09060789	LSP1	0.001569481	0.363126498
cg00697095	GRB7;GRB7	0.007567702	0.363103034
cg07264329		0.000420813	0.363102503
cg09840122	TMPO;TMPO;TMPO	0.000420813	0.363054476
cg07307994		0.003185744	0.362997156
cg04900877		0.000420813	0.362986274
cg04193065		0.003185744	0.362898982
cg16902863	SSPO	0.000420813	0.36287518
cg24749385		0.000636165	0.362852848
cg22040631		0.00961166	0.362770684
cg18013792	MAD1L1;MAD1L1;MAD1L1	0.000420813	0.362754472
cg04525441	ADARB2	0.000420813	0.362718866
cg00855461	SIPA1L2	0.000420813	0.362700848
cg18166144	PTPRE;PTPRE	0.000420813	0.362637542

cg21842959	TSSC1	0.001836098	0.362598913
cg13714797	KDM4C;KDM4C	0.000420813	0.36254549
cg04964845	PPM1A;PPM1A	0.002321262	0.362516079
cg12973294	SYNPO2;SYNPO2;SYNPO2	0.000420813	0.362512228
cg04413853	C17orf64	0.001569481	0.362479703
cg27296341	IPCEF1;OPRM1;IPCEF1;IPCEF1	0.003185744	0.362454304
cg12069540	C10orf58;C10orf58	0.000420813	0.362417741
cg14009440	TMEM53	0.000420813	0.362400434
cg22992279	RBPJ;RBPJ;RBPJ;RBPJ	0.000420813	0.362386754
cg02448743	KAZALD1	0.000420813	0.362319028
cg09358725	LMO2	0.002321262	0.362271358
cg09075743	MAD1L1;MAD1L1;MAD1L1	0.000636165	0.362222567
cg17339440	RAB11FIP1;RAB11FIP1	0.000420813	0.362220676
cg24425727	CDKN1A;CDKN1A	0.000636165	0.36219728
cg25940202		0.000420813	0.362193204
cg07272042		0.000420813	0.362181043
cg13345558	PRKAA1;PRKAA1	0.000420813	0.362160201
cg06716730	DUSP14	0.001173833	0.362075718
cg26130864	SLC4A11	0.000636165	0.362030382
cg10505658	CCDC57	0.001038267	0.362014824
cg08044454	ELMO1;ELMO1;ELMO1;ELMO1	0.000420813	0.36199625
cg23453353		0.000420813	0.361914935
cg13260278	RGS10;RGS10	0.000636165	0.361818833
cg17389988	C7orf10	0.001038267	0.361729761
cg23173586	PNP	0.000420813	0.361712857
cg17547295	CLINT1	0.00961166	0.361637722
cg08873568	ARRDC5	0.001569481	0.361617269
cg16014060	RAD51L1	0.000420813	0.361602546
cg26464796	DHX29	0.00961166	0.361598829
cg07011110	BTN1A1	0.001569481	0.361476467
cg03663984	LMOD3	0.001038267	0.361398598
cg08355863		0.000636165	0.361361054
cg08808812	CMIP;CMIP	0.000420813	0.361358944
cg17593384	AFF1;AFF1	0.001569481	0.361254924
cg20257866	WISP1;WISP1	0.000420813	0.361200062
cg03126694	LPAR6;LPAR6;RB1	0.000420813	0.361103993
cg02464665	MIR1182;FAM89A	0.000420813	0.361091013
cg21697512	CD34;CD34	0.000420813	0.361086429
cg17805199	H6PD	0.000420813	0.361071434
cg18278694	CEP350	0.00961166	0.361001255
cg15468423	GNG7	0.000420813	0.360825141
cg10097651		0.000420813	0.36079247
cg08822136		0.003185744	0.360720823
cg11580897		0.000420813	0.360719284
cg00145118	GNPDA1	0.000420813	0.360604485
cg23022999	RINL	0.000636165	0.360556663
cg00848594	C10orf25;ZNF22	0.000420813	0.360545023
cg01859228	DAPK2	0.000420813	0.36049881
cg11494437	BTD	0.000420813	0.360387883
cg01866518	PXN;PXN;PXN	0.000420813	0.360327336
cg15436606	AFF1	0.000420813	0.360279628
cg00258779		0.002321262	0.360256728
cg08410533	DIP2C	0.000420813	0.360126169
cg15033511		0.007567702	0.359977853
cg19588519		0.002321262	0.359952596
cg06447341		0.000420813	0.359928694
cg20715953		0.000420813	0.359855877
cg10359807	USP7	0.000420813	0.359848818
cg01028142	CMPK2	0.000420813	0.359810687

cg19477205	MIR154	0.007567702	0.359800777
cg25165199	RCBTB2	0.000420813	0.359754311
cg17442852	SLC43A2	0.000420813	0.359735114
cg12883617	UBASH3B	0.000420813	0.359698145
cg13879523	PDE9A;PDE9A;PDE9A;PDE9A;PDE9A;PDE9A;PDE	0.000420813	0.359655678
cg11070818		0.000420813	0.359618737
cg20504474		0.001569481	0.359569812
cg16469223	THPO	0.000420813	0.359449001
cg17692230		0.003613402	0.359443018
cg22257056	CMTM7;CMTM7	0.000420813	0.359412782
cg22029476	LILRA1	0.000420813	0.359350305
cg11937508	GPR1;GPR1	0.001569481	0.359333566
cg13958199	NEK6;NEK6;NEK6	0.000420813	0.359279015
cg09380069	PCTP;PCTP	0.001038267	0.359171608
cg27172287		0.002321262	0.359134292
cg09315264	STK40	0.000420813	0.359112293
cg11082447	PHF7;PHF7	0.000420813	0.359034264
cg06380691	RASA3	0.005790932	0.358941551
cg23100365		0.000420813	0.358889075
cg16125214	PIK3CD	0.000420813	0.358881766
cg22575127	MGMT	0.000420813	0.358827909
cg19858280	CCDC127	0.000420813	0.35879665
cg08930843	ZNF438;ZNF438;ZNF438;ZNF438;ZNF438;ZNF438	0.004997918	0.358743382
cg23840027	EPB41;EPB41;EPB41;EPB41;EPB41	0.001038267	0.358738279
cg09115473	TRIO	0.000636165	0.358695678
cg15490880	PKLR;PKLR	0.000466745	0.358630248
cg03841638		0.000636165	0.358593918
cg19602452	SPRR4	0.000719053	0.358543554
cg18057306	MIPEP	0.000420813	0.3585092
cg07456815	XKR4	0.001569481	0.358358508
cg12564151	DENND3	0.000420813	0.358334587
cg15252509	MALT1;MALT1	0.003185744	0.35830199
cg22443940		0.000636165	0.358295125
cg18842187	ITPR3	0.000420813	0.358277954
cg01922613	ABCA4	0.000420813	0.358197993
cg19584649	ESYT2	0.005790932	0.358188565
cg07223106	PCNXL2	0.002321262	0.358165377
cg01328473	PXDN	0.000420813	0.358145509
cg24570346		0.004405484	0.358137054
cg21121258		0.000420813	0.358119812
cg00130947	LAMA2;LAMA2	0.003185744	0.358118335
cg10558740	FLJ22536	0.000420813	0.35804491
cg09773897	NFIC	0.000420813	0.357972316
cg16460860		0.003185744	0.357947625
cg06151145	SLAIN2	0.000420813	0.357921619
cg06017212	SLC43A2	0.000420813	0.357891454
cg25633383	EHMT2;EHMT2	0.000420813	0.357846241
cg19597449	CCL25	0.002321262	0.357770952
cg05057352	XYLT1	0.001569481	0.357757349
cg21213853	GRM2;GRM2	0.00961166	0.357740699
cg00861207	ABLIM2;ABLIM2;ABLIM2;ABLIM2;ABLIM2;ABLIM2;A	0.000636165	0.357645806
cg21044968	TRAF3IP2;TRAF3IP2;TRAF3IP2;TRAF3IP2	0.000636165	0.357590043
cg06678005		0.000420813	0.357566297
cg17331950		0.000420813	0.35743718
cg04767507	ZMIZ1	0.000420813	0.35742483
cg06469955	RPTOR;RPTOR	0.002321262	0.357346428
cg05924882		0.001569481	0.357295347
cg25727952	CRYL1	0.000420813	0.357256503
cg19273668		0.000420813	0.357118622

cg21008363	POM121C	0.001569481	0.357100918
cg06061966	DGKZ;DGKZ	0.002321262	0.357062005
cg13630239	RRP12;RRP12	0.000636165	0.357034281
cg07912766	SMAD2;SMAD2	0.001038267	0.356911892
cg26065247		0.000420813	0.356869375
cg27175112		0.004405484	0.356843921
cg17578539	HRH2	0.008237556	0.356761389
cg20748132		0.001038267	0.356736122
cg23924526	PRKCE	0.001569481	0.356721842
cg09802610	NPHP4	0.000420813	0.356713862
cg09631880	CRADD	0.000420813	0.356651663
cg01618697		0.00961166	0.356626414
cg02002247	SLC27A1	0.000636165	0.356596364
cg08043998	KDM2B;KDM2B	0.000420813	0.356593777
cg08844900	PLXND1	0.000420813	0.356588791
cg07438246	SLC6A6;SLC6A6;SLC6A6	0.000420813	0.356537997
cg20731819	ADARB2	0.000532358	0.356515101
cg04880657		0.000420813	0.356510218
cg26244225	APOLD1;APOLD1	0.000420813	0.35631091
cg00565090	SNORD23;GLTSCR2	0.001569481	0.356181737
cg08371223	SMPD3	0.001038267	0.356172831
cg11466449		0.000420813	0.356150699
cg18721397	SUB1	0.001038267	0.356112094
cg16173529	MSRA;MSRA;MSRA	0.000420813	0.356053508
cg21197336		0.000420813	0.356046389
cg05292082	TIAM1	0.000420813	0.356010677
cg23490396	LASS6	0.000636165	0.355950622
cg13758310	LETM1	0.001038267	0.355935343
cg24131359	CPM	0.000420813	0.355928797
cg26463496		0.000636165	0.355821428
cg27333269	EFHD2	0.000420813	0.355737084
cg20732160	PFKFB4	0.000420813	0.355715158
cg02960418	PDGFA;PDGFA	0.000636165	0.355699316
cg02676052	LCP2	0.005790932	0.35563745
cg18447299	DIAPH1;DIAPH1	0.001038267	0.355623699
cg17820022	PLCH2	0.001569481	0.3554996
cg02345399		0.002321262	0.355482883
cg08082507	GTF2IRD2P	0.000420813	0.355467016
cg26405097	JARID2	0.002321262	0.355460086
cg07512993	TK2	0.001038267	0.35544878
cg26974444	SNX29	0.002321262	0.355394793
cg12877853	PTPRN2;PTPRN2;MIR595;PTPRN2	0.000420813	0.355384235
cg22614400	GIGYF1	0.000636165	0.355316728
cg21593835	MYLK;MYLK;MYLK;MYLK	0.001569481	0.355300051
cg20944379	IL24;IL24	0.000420813	0.355228316
cg22164891	ZNF217	0.000420813	0.355226152
cg22945666		0.000420813	0.355219593
cg23926598		0.001569481	0.355190178
cg15246989	PLXDC1	0.000636165	0.355153686
cg15664905	PHLDB3	0.000420813	0.355144699
cg04936619	C17orf75	0.000420813	0.355129238
cg22014983	MAP4K3	0.000420813	0.355087712
cg09946870	DENND3	0.000420813	0.355063921
cg00204976		0.000420813	0.354998003
cg25775449	LTB4R;LTB4R;CIDEB	0.000420813	0.354838551
cg05217983	RUNX2;RUNX2;RUNX2	0.005790932	0.35478676
cg10247711	WDR25;WDR25	0.002321262	0.354770116
cg00077838		0.00961166	0.354761637
cg24914483		0.007567702	0.3547468

cg09126279		0.000420813	0.354662033
cg15445722	FAM71D	0.000636165	0.35461088
cg01514033		0.002321262	0.354556034
cg06527166	HIVEP3;HIVEP3	0.000420813	0.354551289
cg27480819	SOX5;SOX5;SOX5	0.003185744	0.354485153
cg00522231	ITGB1BP1;ITGB1BP1	0.001038267	0.354345936
cg04207746		0.00961166	0.354288158
cg09140232		0.000420813	0.35428132
cg22689323	GML	0.000420813	0.354271073
cg18693985	CPEB4	0.00961166	0.354227916
cg09256413	NEGR1	0.000636165	0.354221293
cg16691158		0.000420813	0.354219164
cg13201297	LINGO4;LINGO4	0.000636165	0.354192697
cg02690117		0.000636165	0.354052101
cg24877842	TRAK1;TRAK1	0.000420813	0.354047633
cg07426634		0.002321262	0.353957944
cg19282952		0.005790932	0.353942847
cg11525409		0.001038267	0.353921739
cg19699682		0.002321262	0.353877297
cg12279175		0.000420813	0.353871504
cg08850675	DAGLB;DAGLB	0.000420813	0.353870494
cg00432953		0.004405484	0.353826118
cg04203702	MIR572	0.000420813	0.353750628
cg03771580	OR5A2	0.000420813	0.353722568
cg16706546	IGF2BP2;IGF2BP2	0.003613402	0.353699171
cg03652336	MAML3	0.000420813	0.353663744
cg00981060		0.00657282	0.353634686
cg12614529	MND1	0.000420813	0.353589829
cg22546748		0.000420813	0.353548645
cg04740898	BZRAP1;BZRAP1	0.000420813	0.353411759
cg07720334	DNMT3A;DNMT3A;DNMT3A	0.000420813	0.35336601
cg11552868	ETS1	0.000420813	0.353359711
cg09863136	SH2B2	0.000636165	0.353294122
cg17023203	ADARB2	0.000420813	0.353272092
cg08220149	NDE1;NDE1	0.000420813	0.353243566
cg23072629	PER2	0.000420813	0.353239331
cg26585899	ATXN1L;ZNF821	0.005790932	0.353203835
cg18651347	AUTS2;AUTS2	0.002321262	0.353121362
cg10730398		0.000420813	0.353073819
cg18338142		0.000636165	0.352937783
cg02403929	RPTOR;RPTOR	0.004405484	0.352920332
cg13633625		0.00961166	0.352826282
cg17150663	KCP;KCP	0.002321262	0.352726941
cg06396119		0.002321262	0.352715016
cg07184423	REPS1;REPS1	0.001038267	0.352691007
cg17499212	KCNAB2;KCNAB2	0.000636165	0.352614349
cg24897506		0.000420813	0.352544562
cg26662512	MAD1L1;MAD1L1;MAD1L1	0.000420813	0.352504203
cg24486958		0.000636165	0.35248144
cg13836518	CORO2A;CORO2A	0.000420813	0.352458992
cg00797286		0.000420813	0.352423357
cg24606533	PPAP2B;PPAP2B	0.004405484	0.352400856
cg22065514	COL7A1;UCN2	0.00961166	0.352374468
cg02765962	HRH2;HRH2	0.001038267	0.35229225
cg22141235		0.000420813	0.352291589
cg00771722	IL3	0.000420813	0.352254363
cg03071793	LITAF	0.000420813	0.352239949
cg02516957		0.007567702	0.352223886
cg17351376	CD248;CD248	0.000466745	0.352208418

cg10991454	LCLAT1;LCLAT1	0.000420813	0.352065022
cg17438457		0.000420813	0.35204879
cg01471006	FAM190B	0.000420813	0.352019779
cg11505048	RGL1;APOBEC4	0.002321262	0.35187407
cg21227325	PCSK5	0.003185744	0.351829472
cg06363243	IFT140	0.007567702	0.351795261
cg15245051	APOL3;APOL3	0.000466745	0.351762408
cg17587986	DIP2C	0.000420813	0.351751576
cg09206294	MAPKBP1;MAPKBP1	0.004405484	0.351730746
cg25962657		0.001038267	0.351725659
cg01598007	DUSP27	0.000420813	0.351703924
cg24144574	PRKAR1B;PRKAR1B;PRKAR1B;PRKAR1B;PRKAR	0.004405484	0.351568015
cg16012294	MAD1L1;MAD1L1;MAD1L1	0.000420813	0.351566847
cg08273360		0.000420813	0.351554173
cg25278786	GUK1;GUK1;GUK1	0.00961166	0.351429297
cg20681184	PIK3CD	0.000420813	0.351379373
cg09364733	PDS5B	0.000420813	0.351379024
cg27229664	KIAA0513	0.000636165	0.351307657
cg09286367	MIR589;FBXL18	0.005790932	0.351291759
cg22396119		0.007567702	0.351196082
cg00580752	RNU5E;RNU5D;ACOT12	0.000420813	0.351116001
cg24027780	LOC728743	0.001038267	0.351088682
cg25937598	CALHM3	0.000420813	0.351074691
cg17990365	IFITM3	0.004405484	0.351052689
cg13051249		0.000420813	0.351040593
cg11015768	ST3GAL5	0.000420813	0.350880267
cg15704521	GNA12	0.002321262	0.350852944
cg09267188	PTDSS1	0.002321262	0.350792717
cg25213720	MXD3;MXD3	0.000636165	0.350728896
cg12774454	ABLIM2;ABLIM2;ABLIM2;ABLIM2;ABLIM2;ABLIM2;A	0.000420813	0.350696877
cg19040266	SLC22A5	0.000420813	0.350683223
cg26692202		0.000420813	0.350662571
cg26873754	FUCA1	0.000420813	0.350516857
cg03068346	PFKP	0.002321262	0.350463376
cg05928448		0.000420813	0.35039651
cg01283625		0.001038267	0.35038004
cg00555420	TAPT1	0.002321262	0.350355991
cg05785753	NADSYN1	0.000420813	0.35027744
cg25758828	PAX8;PAX8;PAX8;PAX8;PAX8;LOC440839	0.007567702	0.350223023
cg08784966	TBC1D22B	0.003185744	0.350117537
cg27587780	IRF1	0.004405484	0.350105103
cg08421051	LOC338799	0.000420813	0.3500135
cg23089445		0.002321262	0.349984304
cg02549595	IRF8	0.001038267	0.349937597
cg24770256	FOXK2	0.000636165	0.349919984
cg01186212	ANK3	0.000420813	0.349871635
cg07875068		0.00961166	0.349839213
cg16145324	MAPK14;MAPK14;MAPK14;MAPK14	0.002629558	0.349722907
cg03095773	RFX8	0.000420813	0.349700341
cg26328150	TRAPPC9;TRAPPC9	0.001038267	0.349672484
cg00061520	ADCY9	0.000636165	0.349658112
cg22720761		0.003185744	0.349646609
cg17301987	FARS2	0.000420813	0.349620611
cg18802332	WISP1;WISP1	0.000636165	0.349575134
cg14714222	PREX1	0.001038267	0.349555231
cg18052528	TNIK;TNIK;TNIK;TNIK;TNIK;TNIK;TNIK;TNIK	0.000420813	0.349488284
cg19193595	SMAD3	0.000636165	0.349219849
cg03980250		0.001569481	0.349182366
cg15855924	ANKRD10	0.002321262	0.349148293

cg20806296		0.000420813	0.349113384
cg16633925	PALM;PALM	0.001038267	0.349068516
cg00478326		0.000420813	0.348993544
cg04868238	FLJ14107;BIN3	0.002321262	0.348956589
cg16814786	NCOA4;NCOA4;NCOA4;NCOA4;NCOA4	0.000420813	0.348946866
cg04041639	TBCD	0.000420813	0.348889622
cg01353670		0.00961166	0.348880877
cg11383802	DHCR7;DHCR7	0.003185744	0.348746404
cg03680873		0.005790932	0.348731685
cg13870520	SCARB2	0.001038267	0.3486699
cg20128099	PROSC	0.000636165	0.348647402
cg01938023	TMEM173	0.007567702	0.348604838
cg11036656		0.005790932	0.348598084
cg09570855		0.000420813	0.348591657
cg25148705		0.000636165	0.348470659
cg10973762	DUT;DUT;DUT	0.001173833	0.348458955
cg07411165	ZNF827	0.000420813	0.348451117
cg24175188	PXK	0.001569481	0.348193404
cg10354512	WISP1;WISP1	0.000532358	0.348192544
cg08878567		0.007567702	0.348152284
cg01980810	SPTBN2	0.004405484	0.348147267
cg14595786	SIGLEC9	0.002321262	0.348071758
cg27244242	LY6G5C	0.001038267	0.348016844
cg14999931	PFKFB3	0.002321262	0.347984401
cg07810733	HMGA2	0.002321262	0.347966777
cg06606207	C1orf201	0.000420813	0.347923209
cg14575356	ARHGAP18	0.004405484	0.347836811
cg01577029		0.002321262	0.347809719
cg06022607		0.000636165	0.347788535
cg17173423	MS4A3;MS4A3;MS4A3	0.002321262	0.347737755
cg16410524	RCSD1	0.000420813	0.347714492
cg11169848	LOC100130987;CLCF1;CLCF1	0.001569481	0.347670612
cg10054641	TMEM71;TMEM71	0.00961166	0.34757882
cg21877355	DYNC1H1	0.000636165	0.347577231
cg03249011		0.000719053	0.34750574
cg01527562		0.001038267	0.347505725
cg03059247	C8orf12;FAM167A	0.000420813	0.347499965
cg02578470	MCM2	0.002321262	0.347478914
cg01486260	ITGAX	0.002321262	0.34744419
cg17359975	CTGF	0.000420813	0.347378729
cg03879613	GSTK1;GSTK1;GSTK1;GSTK1	0.000420813	0.347368233
cg14704780	C6orf10	0.000420813	0.34730544
cg11010561		0.003185744	0.347296774
cg04884313	DYNC1H1	0.000420813	0.347236489
cg02989448	SLC45A1	0.000420813	0.347190268
cg26620147	AVIL	0.007567702	0.347188202
cg25518868	DIAPH1;DIAPH1	0.002321262	0.347179429
cg01113811	MAML2	0.000420813	0.347108503
cg09806432	SGSM2;SGSM2	0.000420813	0.347106591
cg27193519	MGRN1;MGRN1;MGRN1;MGRN1	0.000636165	0.347062323
cg02921257	XIRP1	0.001038267	0.346990974
cg12587087		0.000420813	0.346968579
cg08586913	ARHGEF10	0.000420813	0.346947037
cg09676171		0.000420813	0.346843915
cg04428662	MFSD10;MFSD10	0.000420813	0.346841517
cg02719634	SLC22A18AS;SLC22A18;SLC22A18AS;SLC22A18	0.000420813	0.346807856
cg09363068	COX19	0.007567702	0.346792207
cg27570256	LOC100270710	0.002629558	0.346640246
cg20242066	LCT	0.005790932	0.346621566

cg16788865		0.007567702	0.346586896
cg07535628	EFCAB4A	0.000420813	0.346576962
cg01309569	NPHP4	0.000420813	0.346491877
cg13618880		0.000420813	0.346488051
cg03593471		0.000636165	0.346443371
cg04564003		0.000420813	0.34640306
cg02640147	KCNIP1;KCNIP1;KCNIP1	0.00961166	0.346389015
cg04051927		0.004405484	0.346374788
cg16505891	CCDC88C	0.004405484	0.346267004
cg03737424		0.002321262	0.346243716
cg18885162	FOXP4;FOXP4;FOXP4	0.000420813	0.346204827
cg23316191		0.000420813	0.346196234
cg27052418		0.00961166	0.346162823
cg06819373	PTPRN2;PTPRN2;PTPRN2	0.000420813	0.346161837
cg12032027	ZNF217	0.007567702	0.346127837
cg01473602	CSRP2	0.001038267	0.346111138
cg04887078	ADARB2	0.000420813	0.346089954
cg22121647	TMEM140	0.004405484	0.346060995
cg24995503		0.000420813	0.345918938
cg20305578		0.000420813	0.345907158
cg09418321	DYRK4	0.001038267	0.345853072
cg23009067	GNA12	0.000466745	0.345843593
cg10389644	MAFK	0.000420813	0.345839119
cg08331754		0.000420813	0.345824084
cg19038246		0.002321262	0.345775081
cg13780428		0.001038267	0.345734071
cg17628249	C17orf64	0.000420813	0.345694026
cg05079191	ZNF804B;MGC26647	0.000636165	0.345651495
cg25601446	SCN4A	0.000420813	0.345616295
cg25375916	SLC33A1	0.002321262	0.345615513
cg17594256		0.001038267	0.345597625
cg02006142	NSMCE2	0.007567702	0.345540509
cg00963171	MAD1L1;MAD1L1;MAD1L1	0.000420813	0.345489684
cg24154631		0.001569481	0.34545223
cg16692439	ASPHD2	0.003185744	0.345410785
cg02806322	CMTM7;CMTM7	0.000636165	0.345391748
cg01487409	FAM125B	0.000420813	0.34536344
cg10760299	GATM	0.005790932	0.345360585
cg08823240	MAP3K14	0.003185744	0.345296477
cg07568430	CSF1;CSF1;CSF1;CSF1	0.000420813	0.345294212
cg19861623	TTC7B	0.000420813	0.345290273
cg14833933	CAPN3;CAPN3;CAPN3;CAPN3;CAPN3;CAPN3	0.000420813	0.345275299
cg14378259	ELFN2	0.000420813	0.345266483
cg24655669	MAD1L1;MAD1L1;MAD1L1	0.007567702	0.345254291
cg22143352	JDP2;JDP2;JDP2;JDP2	0.000636165	0.345252471
cg12102181	STAG1	0.000420813	0.345250272
cg25003697		0.000420813	0.345229927
cg25343388	MIR589;FBXL18	0.000420813	0.345226068
cg26910870	PRKCZ;PRKCZ;PRKCZ	0.001038267	0.345182519
cg07346187	ZC3H12D	0.002321262	0.345181053
cg12259379	MCCD1	0.000636165	0.345117629
cg08476843	STARD13;STARD13;STARD13;STARD13	0.000466745	0.345106646
cg07935357	PEMT;PEMT;PEMT	0.004405484	0.345056397
cg25444339	HIP1	0.000420813	0.345030339
cg22900075	CUEDC1	0.001038267	0.344991052
cg00318322	GFOD2;GFOD2	0.000420813	0.344950425
cg27470486	ACLY;ACLY	0.001569481	0.344794192
cg08250081		0.000420813	0.344773529
cg09376583		0.000420813	0.344632903

cg09087961	IGF2BP3	0.002321262	0.344627585
cg08492568		0.002321262	0.344563608
cg07585928	DUSP15;TTLL9;DUSP15;DUSP15	0.000420813	0.344559181
cg02253978	CMTM2	0.002321262	0.344525556
cg08370173		0.001038267	0.3443619
cg26550214		0.002321262	0.344294219
cg04450606	PGLYRP3	0.001038267	0.344240809
cg25251478	DDR1;DDR1	0.000420813	0.344203357
cg10365563	SP4	0.000420813	0.344084007
cg25221625	CRYBB3	0.004405484	0.344083388
cg23647554	ZBTB47	0.004405484	0.344073191
cg22542139	PEMT;PEMT;PEMT	0.000636165	0.344063451
cg00393373	ZNF518B	0.000420813	0.344037666
cg08106792	S100A6	0.000420813	0.344019178
cg10840361	COG5;HBP1;COG5	0.000420813	0.344014894
cg07647771	USP10	0.000420813	0.344004017
cg05083852	CMIP	0.002321262	0.343977169
cg19883066	EXT1	0.000466745	0.343967614
cg05659486		0.001038267	0.343814132
cg20808613	AEN	0.001038267	0.343782526
cg16180552	IP6K2;IP6K2	0.000636165	0.343758295
cg08456420	MACF1	0.001569481	0.343732753
cg26227523	PSTPIP1	0.000420813	0.343716058
cg04234923		0.000420813	0.343712661
cg09924669	IFT140;TMEM204	0.000420813	0.343702004
cg03032595	PDLIM7;PDLIM7;PDLIM7	0.001038267	0.343652178
cg24090911	AHRR	0.002321262	0.34363939
cg06650246	MAPKAPK2;MAPKAPK2	0.000420813	0.343551337
cg26462457		0.001038267	0.343442563
cg05474761		0.002321262	0.343412024
cg02400565	CEACAM6	0.000636165	0.343373955
cg08316831	HMG20A	0.000420813	0.343339586
cg23095729	C22orf25;MIR185	0.002321262	0.343325437
cg23167606		0.005790932	0.34330712
cg13578652	UBASH3A;UBASH3A	0.001569481	0.343256593
cg27606341	FYB;FYB;FYB;FYB	0.007567702	0.343248504
cg05863755	TSPAN9;TSPAN9	0.000420813	0.343193883
cg14304236		0.003185744	0.343120128
cg15553397	ST6GALNAC3;ST6GALNAC3	0.000420813	0.342978115
cg13999433	AKNA	0.00961166	0.342898082
cg13135241		0.000420813	0.342607374
cg23531734	LOC100130691	0.000636165	0.342593559
cg18184935	STAC2	0.002321262	0.342427488
cg19161559	CUX1;CUX1;CUX1	0.003185744	0.342411814
cg11421182	BLCAP;BLCAP;BLCAP;BLCAP;BLCAP	0.003185744	0.342389665
cg16776035		0.000420813	0.342384697
cg25729060	MPRIP;MPRIP	0.000420813	0.342335016
cg23732024	LY96	0.007567702	0.342252957
cg26764198	ZCCHC14	0.000420813	0.342251825
cg26562462	TBC1D14;TBC1D14	0.002321262	0.34222684
cg07470570	AUTS2;AUTS2	0.000420813	0.342131921
cg17795540	TRAPPC9;TRAPPC9	0.000636165	0.342023257
cg22152446	FRYL	0.000420813	0.342001308
cg20435097	FAM53B	0.001038267	0.341998972
cg21674927	IL1R2;IL1R2	0.00961166	0.341980812
cg00777315	DCT;DCT	0.001038267	0.341972437
cg04922063	CCNY;CCNY	0.002321262	0.341969098
cg19152255		0.00961166	0.341866277
cg03157862		0.004405484	0.341787258

cg25368647	MXD3;MXD3	0.000420813	0.341771868
cg14596589	SLC12A7	0.004405484	0.341763668
cg26479022		0.002321262	0.341703272
cg22555517	RAP1GAP2;RAP1GAP2	0.000420813	0.341584962
cg16018154	RPTOR;RPTOR	0.000636165	0.341544196
cg27592424	C16orf58	0.001038267	0.341531569
cg22245858	SCNN1A	0.000420813	0.341520237
cg04228709	TIAM2;TIAM2	0.007567702	0.341485657
cg13660174	SURF4	0.007567702	0.341471793
cg13823415	TBXAS1;TBXAS1	0.000636165	0.341220136
cg25936902		0.002321262	0.341220095
cg21769093	VWC2	0.001569481	0.341174671
cg14014879	ABCA4	0.000420813	0.341144336
cg18951390	RPTOR;RPTOR	0.002321262	0.341133694
cg17276535	RGS12;RGS12;RGS12	0.000420813	0.341115194
cg04204452	SLC43A2	0.007567702	0.34109808
cg24753998	C21orf7;C21orf7	0.000420813	0.341032333
cg20686125	AKT3;AKT3	0.005790932	0.340924018
cg07052063	MMS19	0.000420813	0.34091807
cg12374579	ASPSCR1	0.000420813	0.340825659
cg20636526	C6orf10	0.000420813	0.34079523
cg26814335	AGPAT5	0.00961166	0.340744328
cg06532212	FKRP;FKRP	0.007567702	0.340723973
cg22888023	RPTOR;RPTOR	0.000420813	0.340709198
cg00290355	MED12L;P2RY14	0.003185744	0.340662997
cg14930864	YWHAQ	0.001569481	0.340587563
cg17922695	SEPT9;SEPT9;SEPT9;SEPT9;SEPT9;SEPT9	0.003185744	0.340504112
cg11884704	SLC25A25;SLC25A25	0.000420813	0.340415936
cg19620086	SLC38A2	0.002321262	0.340377738
cg27434890	MYB;MYB;MYB;MYB;MYB;MYB;MYB;MYB	0.00961166	0.340300884
cg21757633	SAPS2	0.000420813	0.340285534
cg18919478	CTHRC1	0.003185744	0.340132583
cg04730930	LILRB1;LILRB1;LILRB1;LILRB1	0.000420813	0.340068323
cg02704331	IQCE;IQCE	0.000420813	0.340031696
cg17469471	UXS1	0.000420813	0.340030225
cg26373351	ETV6	0.000420813	0.340008906
cg19982684		0.000420813	0.339881565
cg00607755	PKDCC	0.000420813	0.339828033
cg25333602	LOC146880;LOC146880	0.000420813	0.339764305
cg04023226	CYGB;PRCD	0.000420813	0.339746636
cg18546384	MAD1L1;MAD1L1;MAD1L1	0.000420813	0.339646517
cg04381049		0.002321262	0.339611235
cg27423445	APOLD1	0.007567702	0.339598391
cg26828842		0.000420813	0.339584488
cg09271709	NRXN1;NRXN1	0.003185744	0.339579556
cg21111416	HLTF;HLTF	0.000420813	0.339516558
cg02965295	ANKRD44	0.000420813	0.339496279
cg03879320	SLC7A5	0.000420813	0.339414458
cg14485514	NCRNA00171	0.001038267	0.339404703
cg16167809	LST1;LST1;LST1;LST1;LST1;LST1;LST1;LST1	0.000636165	0.339361036
cg01829163	SLC7A5	0.002321262	0.339356504
cg16572224	SH3PXD2B	0.003185744	0.339331152
cg08730778	NDE1;NDE1	0.000420813	0.33928449
cg03787282		0.00961166	0.339276324
cg21040069	GPT	0.000420813	0.339244072
cg00326902	ROS1	0.000420813	0.339205928
cg19570558	MSH6	0.000636165	0.339177097
cg01526553	MYL6;MYL6	0.001569481	0.339146564
cg16578549	CTNNBIP1;CTNNBIP1	0.001038267	0.339113525

cg17125585	GNG12	0.001038267	0.339078995
cg05765647	IQCE;IQCE	0.000420813	0.339051969
cg11036672	C4orf23	0.000420813	0.338972231
cg04433322	SLC4A1	0.000420813	0.338891664
cg10036075		0.000420813	0.338880223
cg01437221	MAD1L1;MAD1L1;MAD1L1	0.002321262	0.338879728
cg21578050	SEMA6A	0.000420813	0.338791914
cg01113530		0.005790932	0.338733654
cg23859051	SLC43A2	0.000420813	0.338714249
cg00858400	SLC7A5	0.004405484	0.338635346
cg05808246		0.005790932	0.338613033
cg02800100	AUTS2;AUTS2;AUTS2	0.000420813	0.338602212
cg01862311		0.007567702	0.338529702
cg11045746	FAM26F	0.003185744	0.338489023
cg00599809	POM121	0.000420813	0.338467763
cg05819930	GPRC5A	0.001038267	0.338437503
cg07857444		0.000420813	0.338437098
cg18208602	APCDD1	0.000636165	0.338433865
cg16834011	COMT	0.000636165	0.338375298
cg15393585		0.000636165	0.338320092
cg09275693	LOC100133612	0.007567702	0.338267721
cg13298209	GRID2IP	0.000420813	0.338253557
cg18328168		0.000420813	0.338202955
cg14615927	HDAC4	0.003185744	0.338202331
cg01454395		0.005790932	0.338173784
cg20495365	RGS12;RGS12;RGS12	0.000420813	0.33810528
cg10825068		0.005790932	0.338104028
cg17715243	WDR37	0.002321262	0.337937978
cg09039163	ESPNL;ESPNL	0.000420813	0.337925277
cg04353769	MS4A6A;MS4A6A;MS4A6A	0.000420813	0.337881709
cg06568490	HDAC11;HDAC11	0.000420813	0.337866047
cg18470038	ANO6;ANO6;ANO6;ANO6	0.002321262	0.337841244
cg23449544		0.000420813	0.337828974
cg13356324	GUK1;GUK1;GUK1	0.000636165	0.337821878
cg27262821	SEC1	0.004405484	0.337811336
cg02400308	ATP10B	0.000636165	0.337704477
cg26643870		0.004405484	0.33763985
cg06241812	FOXP1;FOXP1	0.003185744	0.337516559
cg03670238	WISP1;WISP1	0.000636165	0.337504073
cg26416981	NINJ2	0.000420813	0.337461925
cg12200164	TNFAIP3	0.000636165	0.337428686
cg12253071		0.00961166	0.337338363
cg09288885		0.001038267	0.337230942
cg00007221	PTPRF;PTPRF	0.000420813	0.337217837
cg18674487		0.002321262	0.337216245
cg01149940	TRABD	0.000420813	0.337206768
cg04790755	CYHR1;CYHR1;CYHR1	0.000420813	0.337003857
cg10030250	MMP2;MMP2	0.001569481	0.336887778
cg22304262	SLC1A5;SLC1A5;SLC1A5	0.000636165	0.336878019
cg27208536	HTRA3	0.003185744	0.336825849
cg07240689	PDCD11	0.000420813	0.336821339
cg17121205	PHLDB3	0.005790932	0.336753032
cg20543183	LY6G5C	0.000420813	0.336710702
cg01076495	ECM1;ECM1;ECM1;ECM1	0.000420813	0.336682037
cg09734418	ITGB1BP1;ITGB1BP1	0.001569481	0.336616529
cg15102179		0.000420813	0.33655203
cg20932822	CMTM8	0.004405484	0.336455835
cg06796885		0.001569481	0.336434166
cg01363734	TESC;TESC;TESC	0.007567702	0.336417241

cg08766256	CMIP;CMIP	0.000420813	0.336349778
cg09918634		0.000420813	0.33634359
cg01364581	GIMAP8	0.000420813	0.336321178
cg19422205	SEPT9;SEPT9;SEPT9;SEPT9;SEPT9	0.001569481	0.336318465
cg26969933	C6orf47;BAT4	0.004405484	0.336306126
cg00305585	IFT140	0.000420813	0.336305838
cg05965490	RBPMS;RBPMS;RBPMS;RBPMS	0.000532358	0.336280736
cg15700582	ARHGAP25;ARHGAP25	0.002321262	0.336240274
cg21711214	HPS4;HPS4	0.003185744	0.336227801
cg15173586	FAM91A1	0.003185744	0.33604102
cg02724472	LRRC17;FBXL13;LRRC17;FBXL13	0.000420813	0.3359987
cg00942920	LAX1;LAX1;LAX1	0.000420813	0.335969962
cg05246110	SAPS3;SAPS3;SAPS3;SAPS3;SAPS3;SAPS3	0.000420813	0.335950106
cg15323253		0.000420813	0.33586178
cg14283710	GTF3C5;GTF3C5	0.001569481	0.33584572
cg26478401	FAS;FAS;FAS;FAS;FAS;FAS;FAS;ACTA2	0.000636165	0.335840531
cg03787988	MRVI1;MRVI1;MRVI1;MRVI1	0.000420813	0.335809686
cg11849692	LDB1;LDB1	0.000420813	0.335790191
cg04493247	NSUN5;NSUN5;NSUN5;NSUN5	0.002321262	0.335781096
cg17018896	MAD1L1;MAD1L1;MAD1L1	0.000420813	0.335767422
cg19722698	TXNRD1;TXNRD1;TXNRD1;TXNRD1;TXNRD1	0.002321262	0.335678126
cg26517376	FAM53B	0.000420813	0.335647062
cg23108232		0.005790932	0.335619043
cg06855485	GAB1;GAB1	0.000636165	0.335545168
cg08672140	LHFPL2	0.002629558	0.335532224
cg04313565	XYLT1	0.000420813	0.335500047
cg20656868	S1PR4	0.000420813	0.335467806
cg15746187	FBXO2;FBXO44	0.000420813	0.335455359
cg16527877		0.000420813	0.335439584
cg25370567		0.001569481	0.335415337
cg26853057		0.000420813	0.335412661
cg03259887		0.000420813	0.33538971
cg23522915	HDAC7;HDAC7	0.000420813	0.335387603
cg10481072	CDK6;CDK6	0.003185744	0.335371294
cg15478483		0.003185744	0.33536788
cg14716686	C21orf7	0.000420813	0.335315399
cg16602500	ZCCHC11;ZCCHC11;ZCCHC11	0.003185744	0.335243523
cg25566714	MUM1;MUM1	0.000420813	0.335237116
cg08705382	GALNT2	0.001569481	0.335232432
cg16223546		0.005790932	0.335188422
cg24231513	RASGRF1;RASGRF1;RASGRF1	0.000420813	0.335165705
cg14272049		0.000636165	0.335163272
cg21784940	REPIN1;REPIN1;REPIN1;REPIN1	0.000636165	0.335162552
cg15611037	PPARD;PPARD	0.000420813	0.335137965
cg05918327	ZFHX3;ZFHX3	0.001569481	0.335131219
cg11231958		0.001777515	0.335100452
cg09756109	IFT140;TMEM204	0.002321262	0.335082937
cg15213650	KDM4B	0.000420813	0.335049059
cg19264571	APCDD1	0.008237556	0.335023017
cg24116385		0.003185744	0.335013571
cg03988107	HIC2	0.000420813	0.334924418
cg05044994	LPP;FLJ42393	0.000420813	0.334912339
cg12049875	RIN2	0.007567702	0.334903804
cg10483525	ZFHX3;ZFHX3	0.000420813	0.334877129
cg07739927	LOC146880;LOC146880	0.000420813	0.334873507
cg09976051	AGA	0.000636165	0.334819196
cg26226408	LOC646982;LOC646982;LOC646982	0.001569481	0.334780174
cg16747164		0.000420813	0.334584621
cg03631656	ANGPT2;ANGPT2;ANGPT2;ANGPT2;ANGPT2;AN(	0.007567702	0.334560918

cg26426774	C8orf41;MAK16;C8orf41	0.000636165	0.334547674
cg22370920		0.002321262	0.334433207
cg21851956	GRID2IP	0.000420813	0.334396268
cg03408904	CECR2	0.007567702	0.334394219
cg26068180	NASP;NASP;NASP	0.002321262	0.334370362
cg14016418		0.000420813	0.334322783
cg07376029	GC	0.002321262	0.334276429
cg20203400	TMEM182	0.002321262	0.334163487
cg09151131		0.000420813	0.334140557
cg03725404	SLC2A9;SLC2A9	0.002321262	0.334110537
cg01330815	LRRN2;LRRN2	0.000420813	0.334106865
cg06586713	PCLO;PCLO	0.000636165	0.334080779
cg03502979	ADPRHL1;ADPRHL1	0.000420813	0.33400702
cg19668951	SLC39A13;SLC39A13	0.000420813	0.33400123
cg06848060	GUCY2D	0.001038267	0.333938986
cg01619533	ANKRD11	0.000420813	0.333898127
cg00294534	PSMG3;PSMG3	0.005790932	0.333853096
cg13537156	C17orf28	0.000420813	0.333814204
cg17194270	SYNGR1;SYNGR1;SYNGR1	0.007567702	0.333767839
cg04157865	TBC1D16	0.000420813	0.333763107
cg03209332		0.005790932	0.333662098
cg07155381	RPH3AL	0.000420813	0.333650191
cg19379721	LPAR2	0.000636165	0.333630178
cg16771104	HESX1;HESX1	0.000420813	0.333592397
cg23344241	SPTBN2	0.003185744	0.33354874
cg09701062		0.000420813	0.333537987
cg05638589	TNS3	0.007567702	0.333535572
cg15948504	PI4KB	0.000420813	0.333453959
cg02010852	CRB2	0.000420813	0.333442366
cg18954479	CDKAL1	0.001173833	0.333423896
cg20382675	FOXP1;MIR1284;FOXP1	0.001038267	0.333420646
cg08780218		0.000420813	0.333417363
cg04049033	RILP	0.000420813	0.333304903
cg16518115	SERINC5	0.000420813	0.333260226
cg10818676	DUSP27	0.000466745	0.333256871
cg24785473		0.007567702	0.333222086
cg03900378		0.002321262	0.333218346
cg25472296	DOCK1	0.00961166	0.333214294
cg25817639	ODZ3	0.000420813	0.333144809
cg02409722	SETD7	0.001569481	0.333099727
cg15559456	S100A13;S100A13;S100A13;S100A1;S100A13;S10	0.000420813	0.333045302
cg06957310	ADAP2	0.003185744	0.333042453
cg05031424	SLC16A11	0.000420813	0.333023161
cg19662895		0.004405484	0.332977842
cg09682727		0.000420813	0.332929941
cg16049597		0.004405484	0.332928646
cg15863827	KCNMA1;KCNMA1;KCNMA1;KCNMA1	0.000420813	0.332892043
cg01092108		0.005790932	0.33283441
cg21502048	PTGER2	0.000420813	0.332772337
cg12128262	IER3IP1	0.003185744	0.332769346
cg10044470		0.000420813	0.332742921
cg26138846	SLC44A4	0.000420813	0.332728087
cg04677846	C21orf33;C21orf33	0.000420813	0.332694059
cg20768358	TP73	0.003185744	0.332691329
cg26875131	VASH2;VASH2;VASH2	0.000420813	0.332552003
cg08741688	RGS12;RGS12;RGS12	0.000420813	0.332539457
cg04585669	VGLL4;VGLL4;VGLL4;VGLL4	0.000420813	0.332466944
cg07636761	USP7	0.004405484	0.332375233
cg13720710	ADARB2	0.001569481	0.332311514

cg13601739	SLCO2B1;SLCO2B1;SLCO2B1	0.000420813	0.332301673
cg00313685	CUX1;CUX1;CUX1	0.002321262	0.332265545
cg17394304	BCL6;BCL6;BCL6;BCL6	0.000420813	0.332184981
cg08126118	ENOX1;ENOX1	0.000420813	0.332127247
cg05948872	PFKFB2	0.000420813	0.332089647
cg17320378	FAM129C	0.000420813	0.332078837
cg11134246	CCDC24	0.004405484	0.33204575
cg00547480	LDB1;LDB1	0.004405484	0.332009248
cg11633461	C10orf54;CDH23	0.000420813	0.332006772
cg10256468	GYG1	0.000420813	0.332006753
cg25507845	CAMK2A;CAMK2A	0.000420813	0.332000548
cg13720395	YWHAG	0.000420813	0.331946644
cg06407843		0.00961166	0.331940851
cg26254667	TNFRSF1A	0.000420813	0.331939925
cg18039797	BUB1B;PAK6;PAK6	0.000420813	0.331936698
cg05202300		0.000420813	0.33186923
cg05517572	STAP2;STAP2;STAP2;STAP2	0.001038267	0.331855546
cg04329347	ALDOA	0.001038267	0.331854447
cg10346364	LOC100130987;CLCF1;CLCF1	0.000420813	0.33185297
cg14642395	SLC22A11	0.000420813	0.331834741
cg19595244	AHCYL1	0.00961166	0.331828212
cg10859151	SH2D4B;SH2D4B	0.000420813	0.331773716
cg00584686	SNTG2	0.000420813	0.331747881
cg20277740	NPHP4	0.000420813	0.331747642
cg02067584	C17orf59	0.007567702	0.331609905
cg08632701		0.000636165	0.331600948
cg13783238	SMC4;MIR15B;SMC4;MIR16-2	0.001038267	0.331561835
cg15511120		0.00961166	0.331543206
cg27051260	C1QTNF4	0.000420813	0.331534477
cg25064352		0.000420813	0.331210409
cg08661112	PANK1	0.000420813	0.331167254
cg18796027	KIFC1	0.000420813	0.331122529
cg16067536		0.000420813	0.331110893
cg14694744	XIRP1	0.001038267	0.331092379
cg08390901	LOC100287216;SH3RF3	0.000636165	0.331068349
cg06098215	NCOA4;NCOA4;NCOA4;NCOA4;NCOA4	0.001038267	0.331013926
cg26721382	MATN1	0.000420813	0.330983398
cg03330678	SEPT9;SEPT9;SEPT9;SEPT9	0.003185744	0.330742089
cg22149555		0.005790932	0.330620371
cg26925644	PHLDB1	0.000420813	0.33061337
cg17701942	STX5	0.001038267	0.330583956
cg20442697	LRRC17;FBXL13;LRRC17;LRRC17;LRRC17;FBXL1	0.000420813	0.330445713
cg00483992		0.000420813	0.330428716
cg14587065	P2RX1	0.002321262	0.330362631
cg02839351	HDGF2;HDGF2	0.000420813	0.330359547
cg08461760		0.003185744	0.330351575
cg16857113	CNTN6	0.003185744	0.330344503
cg16103203	TINAGL1	0.000420813	0.330319653
cg24714606	MBNL2;MBNL2	0.000420813	0.330310431
cg09020213	RASA3	0.001569481	0.330307476
cg03646329	LPAR6;LPAR6;LPAR6;RB1;LPAR6	0.003185744	0.33029753
cg08130175	UBXN10	0.000420813	0.330263478
cg27462475	DOCK9;DOCK9	0.005790932	0.330235378
cg09923593	CEP68	0.001569481	0.330179242
cg11869828	APOC3	0.000420813	0.330174971
cg08383160		0.000420813	0.330173941
cg01301660		0.002321262	0.330139748
cg01366670	CBFA2T3;CBFA2T3	0.000420813	0.330071666
cg06610259	MLXIP	0.000420813	0.330019566

cg12724086	MKLN1	0.000420813	0.32999434
cg10857441	POLS	0.00961166	0.329967221
cg21484631	UHRF1;UHRF1	0.000420813	0.329912469
cg17298005	ELK3	0.000420813	0.329901147
cg15701612		0.001038267	0.329857195
cg00113315	SLC2A5;SLC2A5	0.000420813	0.329809055
cg12689752	IQGAP3	0.000636165	0.329785682
cg04468671		0.007567702	0.329691495
cg13361023	ATP10A	0.000420813	0.329659007
cg02462661	STOML1	0.000420813	0.329658721
cg20117260	ATP5SL;ATP5SL;ATP5SL;ATP5SL;ATP5SL;ATP5S	0.004405484	0.329604794
cg06654628	SYNPO;SYNPO;SYNPO;SYNPO	0.000420813	0.329569645
cg20687098	WIPF1;WIPF1	0.007567702	0.329520477
cg18411043	LAPTM5	0.000420813	0.32951473
cg13948857	C5orf56	0.000420813	0.329513933
cg09653641	CDK6;CDK6	0.000420813	0.329497034
cg04623297	RFX8	0.001038267	0.329477183
cg07827420	SEPT9;SEPT9;SEPT9;SEPT9;SEPT9	0.002321262	0.329477138
cg14457610	LMOD3;LMOD3	0.000420813	0.329450796
cg20232503	PAK1;PAK1	0.001569481	0.329444691
cg07523043	TRIM26	0.007567702	0.329417689
cg15492982		0.000420813	0.329222047
cg06513139	UBXN6;UBXN6	0.000420813	0.329193151
cg14742809	ZSWIM6	0.000420813	0.329143292
cg05522498	DCPS	0.000420813	0.32911211
cg02997962	C20orf26;CRNKL1;C20orf26	0.007567702	0.329095484
cg17301053	RPS6KA2;RPS6KA2	0.001038267	0.329070509
cg00975876	PRICKLE4	0.000420813	0.329003163
cg02847674	NPPA	0.000420813	0.328954846
cg22787468	SEZ6;SEZ6	0.004405484	0.328899813
cg06784232	CSGALNACT1;CSGALNACT1	0.000420813	0.328876123
cg15571705	BARX2	0.004405484	0.328803334
cg01147055	RASA3	0.000420813	0.328577919
cg08489478	ZFYVE27;ZFYVE27;ZFYVE27	0.000420813	0.328564547
cg11075561		0.002321262	0.328551958
cg17220749	GALNT2	0.002321262	0.328534639
cg23829395		0.000420813	0.328470089
cg08314457		0.002321262	0.328326057
cg21149811	SNTG2	0.000420813	0.328319033
cg02645135		0.003185744	0.328270079
cg23524191	MAD1L1;MAD1L1;MAD1L1	0.001569481	0.328237309
cg00999858	MAN1C1	0.000420813	0.328123043
cg16596859		0.000420813	0.32810104
cg08380391	AATK	0.000420813	0.328074392
cg11948905		0.000420813	0.328041277
cg07862930	KCNJ16;KCNJ16;KCNJ16	0.007567702	0.327993953
cg17910478		0.000420813	0.327969994
cg27353085	PLXND1	0.000420813	0.327952051
cg02761866	ASB8	0.000420813	0.327901594
cg12385021		0.000420813	0.327843986
cg05657709	ILDR1	0.000420813	0.327820445
cg12422704	ABR;ABR	0.000420813	0.327766487
cg19027424	SH3PXD2B	0.000420813	0.327744989
cg09141635	RBM47	0.00961166	0.327585983
cg26103369	KIAA0317	0.000420813	0.327565476
cg16125874	CLEC14A;CLEC14A	0.000420813	0.327463065
cg08906309	LOC100128288	0.003185744	0.327453139
cg08783317		0.000636165	0.327429601
cg27496296	BAHCC1	0.003185744	0.327412807

cg03290748	PIAS1	0.000420813	0.327381563
cg25214914	RXFP4	0.000420813	0.327374442
cg14963928		0.005790932	0.327348742
cg04535236	GATA2;GATA2;GATA2	0.001038267	0.327335612
cg14185922	ENG;ENG	0.000420813	0.327312567
cg22137236		0.000636165	0.327309938
cg16111448	DHDPSL;DHDPSL	0.000420813	0.327278234
cg06524846		0.002321262	0.327277763
cg18493214	KIF13A;KIF13A;KIF13A;KIF13A	0.000420813	0.327158164
cg13470063	TIE1	0.000420813	0.327141898
cg19341945		0.000420813	0.32710131
cg14527110	P4HA2;P4HA2;P4HA2;P4HA2;P4HA2	0.000420813	0.327097466
cg04189320		0.000420813	0.327031951
cg19561297	LRRC17;FBXL13;LRRC17;LRRC17;LRRC17;FBXL1	0.000420813	0.326962869
cg22341788	FLT1;FLT1;FLT1;FLT1	0.000420813	0.326922251
cg16981685		0.003185744	0.326908443
cg21220520	C14orf105;C14orf105	0.002321262	0.326889498
cg09530438		0.000420813	0.326723042
cg05246645		0.000420813	0.326673787
cg02291020	ANXA11;ANXA11;ANXA11	0.003004124	0.326668142
cg02332537	RTP3	0.000420813	0.326664378
cg25542745		0.003613402	0.326644118
cg15779785	CACNA1C;CACNA1C;CACNA1C;CACNA1C;CACN	0.007567702	0.326614186
cg14149774	FAM69B	0.002321262	0.326606254
cg21171115	AKAP2;PALM2-AKAP2;PALM2-AKAP2;AKAP2	0.00961166	0.326522987
cg06714981	SND1	0.000420813	0.326473972
cg20752878	ASGR1	0.007567702	0.326452471
cg18041814	CPNE5	0.000420813	0.326342919
cg12131208	C17orf64	0.005790932	0.326311297
cg08497772		0.000420813	0.326287536
cg23779478	SLA2;SLA2	0.003185744	0.326282066
cg15930380	MIER1;MIER1;MIER1;MIER1;MIER1;MIER1;MIER1	0.000420813	0.326256087
cg24212377		0.000420813	0.326209177
cg19445335	ADAP1	0.000420813	0.326182292
cg12271487	ARMC2	0.001038267	0.326151732
cg26544722		0.004405484	0.326136799
cg06412669	RPTOR;RPTOR	0.000420813	0.326092432
cg16289618	LOC285830;LOC285830	0.005790932	0.32597739
cg02414045	SMAD3	0.000420813	0.325894545
cg17839611	ABI3;ABI3;GNGT2	0.000636165	0.325853079
cg04087571	SIK3	0.001569481	0.325766578
cg18964319		0.007567702	0.325732719
cg02108623	PRDM1	0.000420813	0.325730284
cg16665442		0.003185744	0.325642641
cg10270895	PARD3	0.005790932	0.325589248
cg13226839	LYRM4;LYRM4	0.002321262	0.325544684
cg14043316	PLXNB2	0.000420813	0.325535051
cg03763518	C1orf54	0.000420813	0.325523861
cg01546472	ODZ3	0.000636165	0.325500985
cg24531534	LOXL2	0.001038267	0.325500133
cg22978003	UGT3A2;UGT3A2;UGT3A2	0.000420813	0.325440616
cg04061506		0.003185744	0.325438387
cg27613013	MED9	0.000420813	0.325430532
cg08830576	C16orf91	0.000420813	0.325384123
cg03348100		0.005790932	0.325367328
cg23685994		0.000420813	0.325363997
cg11415156		0.000420813	0.325321902
cg07840332		0.000420813	0.325287699
cg00864684		0.000420813	0.325262821

cg24442766	INADL	0.003185744	0.325211791
cg27428104	TNXB	0.000420813	0.325189291
cg24055836	FAM190A;FAM190A	0.001569481	0.32518567
cg00825967	CBFA2T3;CBFA2T3	0.000420813	0.325169827
cg12649175	DPP4	0.000420813	0.325149016
cg24074477	CECR2	0.007567702	0.3251398
cg14115597	SSBP2	0.001038267	0.325131527
cg01929340	GAS7	0.002321262	0.325124281
cg00469897		0.002321262	0.325113104
cg03226872	BLCAP;BLCAP;BLCAP;BLCAP;BLCAP	0.000420813	0.325113036
cg21535931	PLEC1;PLEC1	0.003185744	0.32509926
cg14519515	ADRBK1	0.000420813	0.324950055
cg14743210		0.000420813	0.324893264
cg04701716	CKLF;CKLF;CKLF;CKLF;CKLF	0.000636165	0.324892629
cg14531665	SPIN1	0.000420813	0.324889702
cg17806069		0.000420813	0.324879097
cg06333233	CREG2	0.002321262	0.32486539
cg15481493		0.007567702	0.324819619
cg15068180		0.007567702	0.324805481
cg05486924	C3orf21	0.000420813	0.3248025
cg05382012	AGRP;AGRP	0.005790932	0.32474348
cg27534833		0.000420813	0.324726814
cg17749384	MPP7	0.000420813	0.324725562
cg05729480	9-Sep	0.000420813	0.324691553
cg02288964	AGRN	0.001038267	0.324677446
cg02505015		0.000420813	0.324652323
cg20869310		0.000420813	0.324636854
cg13178170	TGFBR3	0.000420813	0.324626806
cg10709350	LAPTM5	0.000420813	0.324535422
cg27637303		0.002321262	0.324519669
cg13167431		0.000420813	0.324490271
cg27292264	MIR1280;EEFSEC	0.000420813	0.324432508
cg06458106		0.00961166	0.324415006
cg18109874	RFX1	0.002321262	0.324366517
cg00864618		0.000636165	0.324365064
cg20674658	RNPEP	0.000420813	0.324355237
cg13109911	PEA15	0.000420813	0.324333836
cg04921244		0.000420813	0.324260585
cg03775372	FRMD4A	0.000420813	0.324257495
cg17465423	ZNF385A;ZNF385A	0.000420813	0.324230541
cg03455424		0.000636165	0.324220673
cg08461451	LINGO3	0.00961166	0.324214446
cg06624267		0.000420813	0.324165028
cg22129323	VAMP1;VAMP1;VAMP1	0.000420813	0.324127852
cg25383605	TIAM2;TIAM2	0.000420813	0.324016469
cg18173263	PEMT;PEMT;PEMT	0.001569481	0.324004446
cg07037725	SPRY4;SPRY4	0.000636165	0.323927537
cg07974367		0.000420813	0.323883238
cg00930515		0.000420813	0.323872065
cg08552043	KCNQ1;KCNQ1	0.000636165	0.323865869
cg17051239	CCDC121;CCDC121;GPN1;GPN1;CCDC121;GPN1	0.000420813	0.323751725
cg17711527	LAX1;LAX1;LAX1;LAX1	0.000420813	0.323696884
cg21336373	P2RX1	0.002321262	0.323685687
cg13671299	EHD1	0.007567702	0.323653933
cg21142904	KIRREL	0.000420813	0.323627335
cg15002163	HDAC4	0.007567702	0.323622615
cg11296767	SERPINB9	0.003185744	0.323615329
cg14774440	RAB11FIP1;RAB11FIP1;RAB11FIP1;RAB11FIP1	0.000420813	0.323589277
cg03567896	GRPEL1	0.001038267	0.323559942

cg08791347	FRMD4A	0.000420813	0.323386457
cg16649560	IL4R;IL4R	0.000420813	0.323381103
cg12292060	GABBR1;GABBR1	0.000420813	0.323305838
cg00122628	WISP1;WISP1	0.000420813	0.323303611
cg03718845		0.00961166	0.323298242
cg01048349	FLJ10357	0.000420813	0.323266947
cg25461186	MLXIP	0.001569481	0.323236443
cg14667192	ADARB2	0.000420813	0.323223714
cg13762691	CECR2	0.002321262	0.3231543
cg09323092	LMF1	0.001569481	0.323056719
cg01833923	GNGT2;ABI3;ABI3;GNGT2	0.000420813	0.323049853
cg07517739		0.00961166	0.323044955
cg22360765	TTC7A	0.000420813	0.323024691
cg15690760	POLS	0.007567702	0.322958772
cg07048516	C22orf15	0.000420813	0.322847223
cg27471192		0.001038267	0.322839385
cg19737746	ELK3	0.003185744	0.322776929
cg26539468	CCDC111	0.00961166	0.322725382
cg20306694	BIN2	0.00961166	0.32272304
cg14603886	ERN1	0.001038267	0.322676353
cg00813264		0.000420813	0.322663547
cg12882572	DUSP14	0.000420813	0.322643776
cg26203136	PRKAR1B;PRKAR1B;PRKAR1B;PRKAR1B;PRKAR	0.008581131	0.322593425
cg23157419	PTPN9	0.001038267	0.32258852
cg04022194		0.000636165	0.322547376
cg03619840		0.002321262	0.322528323
cg24706966	MAD1L1;MAD1L1;MAD1L1	0.000420813	0.322526998
cg10118167	PLXNC1	0.002321262	0.322510961
cg25215890	CD48	0.000420813	0.322464406
cg20651389	GP6;GP6	0.001038267	0.322458318
cg18601229	EHD4	0.001038267	0.322432999
cg25843651	KLHL25	0.000420813	0.322414792
cg26239850		0.000420813	0.322266781
cg18517055	WDR45L	0.005790932	0.322197898
cg23347250	BTBD6;BRF1;BRF1;BRF1	0.004405484	0.322156928
cg14613228		0.001038267	0.322129183
cg09318283		0.001038267	0.322122085
cg20450198		0.002321262	0.322098221
cg04667114		0.000420813	0.322076919
cg01797450		0.001569481	0.322066688
cg09190408	PPP1CA;PPP1CA;TBC1D10C;PPP1CA	0.000420813	0.322066494
cg20630690	ZGLP1	0.000420813	0.321989073
cg02113385	NFATC1;NFATC1;NFATC1;NFATC1;NFATC1	0.007567702	0.32197644
cg16570223	CHRNA3;CHRNA3	0.000636165	0.321953658
cg27223728		0.003185744	0.321953541
cg21118780	MAP4K4;MAP4K4;MAP4K4	0.001569481	0.321917918
cg26415516	IPCEF1;OPRM1;IPCEF1;IPCEF1	0.000420813	0.321884906
cg13681496		0.001569481	0.321876861
cg22548485	TULP1	0.002321262	0.321851985
cg11129609	WDR46;WDR46	0.00961166	0.321755312
cg13060454		0.000420813	0.321753442
cg20722088	DUSP6;DUSP6	0.001038267	0.321736238
cg10621597		0.000420813	0.321625299
cg02978140		0.000636165	0.321603892
cg05703230		0.004405484	0.321597917
cg24534583	RNF216;RNF216	0.004405484	0.321540825
cg14335159		0.002321262	0.321533643
cg20591695	FLJ32065	0.002321262	0.321510101
cg25467710	HMCN1	0.001569481	0.321451751

cg21932452	NOS3	0.000420813	0.321442091
cg18352721		0.002321262	0.321439053
cg21721489	HMGB1	0.000420813	0.321430594
cg25314111	AACSL	0.000636165	0.321423849
cg26452915	GNAS;GNAS;GNAS;GNAS;GNAS;GNAS;GNAS;GN	0.002321262	0.321421882
cg10937494	SLC16A3;SLC16A3;SLC16A3	0.000420813	0.321398845
cg01534887	C10orf55	0.000420813	0.321379718
cg21686213	IFITM1	0.001038267	0.321335039
cg13641082	SNX8	0.000636165	0.321305994
cg07172701	SERINC5	0.001569481	0.321282932
cg09879253		0.000420813	0.321279509
cg00827893	PDS5B	0.002321262	0.321103252
cg26922854		0.005790932	0.321042303
cg07241457		0.000420813	0.321012524
cg24585377	RPS6KA1	0.000420813	0.320937669
cg12549180		0.000420813	0.320919397
cg00770754	ANKRD11	0.007567702	0.320914258
cg06974034	ACVR1C;ACVR1C;ACVR1C;ACVR1C	0.004405484	0.320900634
cg04381888	HDAC7;HDAC7	0.000420813	0.320859406
cg14221700		0.000420813	0.320847127
cg12799029	ABR;ABR;ABR	0.002321262	0.320828919
cg05404787	C2orf48;C2orf48	0.000420813	0.320803431
cg20623350	ENTHD1	0.007567702	0.320780372
cg25959472	FBXW11;FBXW11;FBXW11	0.002321262	0.320668247
cg10075360	DPYSL2	0.000420813	0.320578555
cg02059176		0.005790932	0.320556228
cg12186909	PDE4A	0.000420813	0.32054613
cg06686156	ACSM5	0.000420813	0.320534849
cg01400685	FADS2	0.00961166	0.320526656
cg19040012	SCUBE3	0.000420813	0.320488657
cg18289508		0.002321262	0.32046313
cg02393699	PRKCZ;PRKCZ;PRKCZ	0.005790932	0.320430872
cg23709231		0.000420813	0.320327313
cg13026137	CLIC1	0.000420813	0.320317052
cg03717584	CCDC109A	0.001038267	0.320310713
cg01598733	ILDR1	0.002321262	0.320287566
cg13621113	SFRS8	0.004405484	0.320258308
cg27020966		0.000420813	0.320253642
cg01579884	ELMO1;ELMO1;ELMO1	0.000420813	0.320232084
cg08129092	INTS3	0.000420813	0.32021402
cg26509012	STX2;STX2	0.007567702	0.320183586
cg09347578	GRID2IP	0.001569481	0.320149807
cg15474579	CDKN1A;CDKN1A	0.000420813	0.32010899
cg27342781		0.000420813	0.320105231
cg09344631	COL11A2;COL11A2;COL11A2	0.001038267	0.320060641
cg20560862	NAP1L4	0.000420813	0.320041069
cg01243072		0.003185744	0.320007344
cg00951869	ADCY4;RIPK3	0.000420813	0.319967388
cg18332229	ADAP1	0.003185744	0.319887053
cg16096836	TXN2	0.000420813	0.319805874
cg17098965	ZNF217;ZNF217	0.00961166	0.31975774
cg09173565	RPTOR;RPTOR	0.000420813	0.319719544
cg14334460	NELF;NELF;NELF;NELF	0.000420813	0.319713697
cg07096238		0.005790932	0.31971058
cg10603757	RAC2	0.001569481	0.31961749
cg19736994	MAD1L1;MAD1L1;MAD1L1	0.000420813	0.319577669
cg14503357	C9orf117	0.000420813	0.319568055
cg23204276	AP1B1;AP1B1;SNORD125;AP1B1	0.002321262	0.319535146
cg24091819	GPR133	0.005790932	0.319498175

cg01490728	SARDH;SARDH	0.001569481	0.319474921
cg25028542	ANLN;KIAA0895	0.000420813	0.319434867
cg06627334	PCDH12	0.004405484	0.319403378
cg23822407	ZHX2	0.000420813	0.319367541
cg08189139		0.000420813	0.319362928
cg24768187	LY6G5C	0.002321262	0.319326057
cg05448032		0.001038267	0.319321494
cg15491094		0.000420813	0.319290625
cg00812557	TBC1D1	0.000420813	0.319263609
cg07633835	FBXO18;FBXO18	0.007567702	0.319214334
cg16801797	NAV2;NAV2;NAV2;NAV2	0.000420813	0.319198648
cg23534245	BMP2	0.000420813	0.319158766
cg25152942	MIA	0.000420813	0.319127106
cg27278668		0.000420813	0.319098629
cg03443467	SH3BP2;SH3BP2	0.000420813	0.319085262
cg01103597	RUNX3;RUNX3	0.000420813	0.319060422
cg00729133	ZNF804B;MGC26647	0.000420813	0.319052798
cg14582049	GPM6A;GPM6A;GPM6A	0.002321262	0.319046234
cg01204911	ARHGAP26;ARHGAP26	0.002321262	0.31902836
cg14016363		0.000636165	0.318954773
cg09365259	TUBA8	0.002321262	0.318953773
cg15404019	CHST11	0.000420813	0.318944757
cg22189286	HSPB8	0.001038267	0.318943477
cg08574558		0.002321262	0.31893231
cg20407861	AKAP12	0.000636165	0.318896136
cg01381934	STAB1	0.000636165	0.318883133
cg24757978		0.000420813	0.318876961
cg08730070	COMT;COMT;COMT	0.000420813	0.31880596
cg00473501	MPO	0.00961166	0.318783689
cg20631351	PALM;PALM	0.000636165	0.318780194
cg23181573	ATP2A3;ATP2A3;ATP2A3;ATP2A3;ATP2A3;ATP2A	0.000420813	0.318710667
cg25902229	RPTOR;RPTOR	0.000420813	0.318606764
cg14265709	SLCO5A1;SLCO5A1;SLCO5A1	0.001569481	0.318593547
cg11555067	INPP4A;INPP4A;INPP4A;INPP4A	0.000420813	0.318559517
cg14136214	DAB2IP	0.001038267	0.318534413
cg24481882	PDE7B	0.002321262	0.318489349
cg03739177	ABHD15	0.001569481	0.318476461
cg22979807	TMEM150B	0.000420813	0.318472203
cg08428949	TTBK2	0.000636165	0.318406393
cg11498228	ARHGEF10	0.00961166	0.318375899
cg19506849	TCF7L2;TCF7L2;TCF7L2;TCF7L2;TCF7L2;TCF7L2	0.000420813	0.318342315
cg13541073		0.001038267	0.318331138
cg19641315	P2RY6;P2RY6;P2RY6	0.007567702	0.318315866
cg18325841	ARHGAP21	0.004405484	0.318300147
cg22891003	PDGFA;PDGFA	0.000420813	0.318258565
cg07727594	SLC13A3;SLC13A3	0.001569481	0.318206637
cg03493211	BAIAP2L1	0.000420813	0.318195509
cg03296935	OSTalpha	0.000420813	0.318109529
cg18998365	NR3C1;NR3C1;NR3C1;NR3C1;NR3C1;NR3C1;NR3	0.001569481	0.318055366
cg16328023	KIAA0895;KIAA0895	0.003185744	0.318012179
cg00388154	CALML4;CALML4	0.001777515	0.317983736
cg02641801		0.000420813	0.31795193
cg00387090		0.000420813	0.317887093
cg04948649	LSP1;LSP1;LSP1;LSP1	0.007567702	0.317853412
cg09169516	MAPKAPK2;MAPKAPK2	0.001569481	0.317830207
cg02311801	ARHGEF17	0.003185744	0.317761871
cg13967936	GJA4	0.001038267	0.31773674
cg02172058	C17orf64;C17orf64	0.002321262	0.317718637
cg22488259	NAV1;NAV1	0.000420813	0.317672479

cg01686739	BTBD11	0.000420813	0.317649583
cg17804886	RASA3	0.003185744	0.317649567
cg23036452	ATP2B4;ATP2B4	0.004405484	0.317634221
cg16251626	WDR65;WDR65;WDR65;WDR65	0.002321262	0.317631934
cg26415789	SEPT9;SEPT9;SEPT9;SEPT9;SEPT9;SEPT9;SEPT	0.000420813	0.317613507
cg05447955	GAB2	0.001038267	0.317600707
cg11654325	C2orf85	0.005790932	0.317578107
cg09241885	C20orf118	0.000420813	0.3175614
cg06557644	NOD1	0.000420813	0.317481808
cg20847746	S100A13;S100A13;S100A13;S100A1;S100A13;S10	0.000420813	0.317415179
cg16626670	CLEC4G;CLEC4G	0.000420813	0.317348456
cg10044184		0.001569481	0.317342466
cg14816825		0.002321262	0.317339937
cg15057046		0.003185744	0.317309483
cg11408036	DDX56	0.000420813	0.317280668
cg09719269		0.001038267	0.317269011
cg00931181		0.007567702	0.317253764
cg15704408	NFATC1;NFATC1;NFATC1;NFATC1	0.000466745	0.317244207
cg03154580	RALB	0.000420813	0.317243182
cg09790226		0.000420813	0.317231422
cg26875137		0.000420813	0.317201697
cg13221767	UCP3;UCP3	0.000636165	0.317188887
cg08623942	SMURF1;SMURF1	0.002321262	0.317183828
cg21220403		0.001569481	0.317178575
cg11812071	TNFAIP3	0.000420813	0.31717161
cg04355222	CD81	0.001569481	0.317158175
cg17841440	KLF16	0.000420813	0.317136371
cg01984854		0.003185744	0.31713447
cg13333954		0.000420813	0.317123047
cg14784664		0.001569481	0.317116924
cg04571584	WNT2B	0.000420813	0.317088931
cg18043514		0.002321262	0.317026886
cg18817487	HAL	0.000420813	0.316985005
cg08119655	PPFIBP2	0.001569481	0.316984249
cg21523688	SORD	0.001038267	0.316976605
cg01192051	POLG;POLG	0.001569481	0.316864908
cg11690724	EBF1	0.000636165	0.316863377
cg07092805	CRYL1	0.000466745	0.316768165
cg12467090	PIK3C2B	0.001038267	0.316693528
cg13123009	LY6G6E;LY6G6D;LY6G6E	0.000420813	0.316610958
cg16961269		0.000420813	0.316590669
cg06561728	GEMIN4	0.000636165	0.316590286
cg26628100	IFT140	0.000420813	0.31657853
cg15770575		0.007567702	0.316559747
cg11886358	FLI1	0.002321262	0.316551213
cg01964170		0.003613402	0.31652385
cg01520105	TRAF3IP2;TRAF3IP2;TRAF3IP2;TRAF3IP2;TRAF3	0.000420813	0.316508582
cg09685472	CMIP	0.000420813	0.316463903
cg17968037	ZCWPW1	0.001569481	0.316449531
cg10117369	LAX1;LAX1	0.001038267	0.316409085
cg14381712	VPS13D;VPS13D	0.000420813	0.316403511
cg03778138	KRTAP12-3;C21orf29	0.002321262	0.316394866
cg07165029	MAGI1;MAGI1;MAGI1	0.001038267	0.316385763
cg22535523	BAHCC1	0.000466745	0.316379957
cg04270013		0.007567702	0.316349943
cg10024799	CACNA1C;CACNA1C;CACNA1C;CACNA1C;CACN	0.000420813	0.316316876
cg23627828		0.000420813	0.316233755
cg19060120	RPTOR;RPTOR	0.000420813	0.316221001
cg00223767	UVRAG	0.000636165	0.316215062

cg24033471	CACNA1C;CACNA1C;CACNA1C;CACNA1C;CACN	0.002321262	0.316197964
cg21808448	MACF1;MACF1	0.000420813	0.316181922
cg13080487		0.000420813	0.316174706
cg25673241	RPTOR;RPTOR	0.000420813	0.316108757
cg04002220	SPG7;SPG7	0.000420813	0.316088666
cg08497239		0.000420813	0.316085459
cg18737081	ZMIZ1	0.007567702	0.316074583
cg02673002	RNF169	0.000420813	0.316069634
cg01178099		0.00961166	0.316049307
cg22133826	RAPGEF6;RAPGEF6;RAPGEF6;RAPGEF6;RAPGE	0.000420813	0.316048193
cg22455720	TBCD	0.000420813	0.316036963
cg23047992	CUX1;CUX1;CUX1	0.000420813	0.315985843
cg04636331	ADARB2	0.000420813	0.315978183
cg27144670	TMOD3	0.004405484	0.315946607
cg18145759	SH3RF3	0.000636165	0.315915967
cg24131343	LIMS2;GPR17;GPR17;LIMS2;LIMS2;GPR17;GPR17	0.000420813	0.315885399
cg07051833	UBE2N	0.000420813	0.315865246
cg17973661	MAP2K5;MAP2K5	0.000420813	0.315821654
cg08758727	QDPR	0.001836098	0.315813652
cg06720713		0.000636165	0.315769792
cg06663615	BAHCC1	0.004405484	0.31574193
cg26017492	RPS6KA2;RPS6KA2	0.005790932	0.315654535
cg07893986	LRRFIP1;LRRFIP1	0.000420813	0.315636033
cg25252561	CAMK2G;CAMK2G;CAMK2G;CAMK2G;CAMK2G	0.000420813	0.315593899
cg12361262		0.002321262	0.315536469
cg04952324	CLIP2;CLIP2	0.004405484	0.315509709
cg11922164	SYT15;SYT15	0.000420813	0.315508075
cg25510614	MFSD7	0.000420813	0.315482235
cg16432031	DHRS3	0.000420813	0.315434498
cg06785060	HIVEP3;HIVEP3	0.000420813	0.315428062
cg17910470	USP36	0.000636165	0.315402524
cg19014730	FKBP5;FKBP5;FKBP5;FKBP5	0.000420813	0.315367798
cg19513876		0.004405484	0.315365013
cg11998307	SCARF1;SCARF1;SCARF1;SCARF1;SCARF1	0.000636165	0.315315915
cg20820876		0.002629558	0.315300021
cg15817163	SFRS8	0.002321262	0.315201997
cg08927754	ANKRD11	0.001038267	0.315058475
cg12537379	CUX1;CUX1;CUX1	0.000420813	0.315045828
cg21840948	SNAI1	0.000420813	0.314968515
cg26672202	GAB2;GAB2	0.002321262	0.314963134
cg09965419	DDR1;DDR1	0.001569481	0.31487078
cg01749539	SEPT9;SEPT9;SEPT9;SEPT9;SEPT9;SEPT9;SEPT	0.000420813	0.314836445
cg18951367	SPNS3	0.000420813	0.314808914
cg20308817	CLEC11A	0.004405484	0.314806374
cg12800962		0.00961166	0.31475743
cg17420028	HDAC4	0.000420813	0.314733138
cg21702188	STX5;NXF1;NXF1	0.001569481	0.31457994
cg04160876	MYH11;MYH11;MYH11;MYH11	0.004405484	0.314577962
cg20573931	IL7	0.000420813	0.314548982
cg22616343	TNXB;TNXB	0.000420813	0.314547703
cg08268075	INSC	0.000420813	0.314540642
cg00402381		0.004405484	0.314526032
cg12700039	TBC1D16	0.000420813	0.314467474
cg14688662		0.000420813	0.314388313
cg14310198	RAPGEF1;RAPGEF1	0.003004124	0.314376019
cg00074771	WDR76;WDR76	0.007567702	0.31434405
cg24787238	MAD1L1;MAD1L1;MAD1L1	0.004405484	0.314279756
cg00569418	C19orf76;C19orf76	0.007567702	0.314224441
cg22546130	COMT;COMT;COMT;COMT	0.000636165	0.314137544

cg21504005	CDYL;CDYL;CDYL;CDYL	0.000420813	0.314106598
cg11858213	CRTAP	0.000420813	0.314084369
cg14029001	CCND3;CCND3	0.000420813	0.314075738
cg25431535		0.000420813	0.31403813
cg24190223	CSPP1;CSPP1	0.007567702	0.313987059
cg27635976	CBFA2T3;CBFA2T3	0.000636165	0.313951419
cg08727202	MPST;MPST;TST;MPST;MPST	0.000420813	0.313890287
cg06517940	LCK	0.001777515	0.313889039
cg14349937	RNLS;RNLS	0.005790932	0.313860954
cg17168630	PLAUR;PLAUR;PLAUR	0.001038267	0.313853664
cg27573935	C3orf54	0.001569481	0.313839182
cg08112313	XRCC1	0.000420813	0.313782841
cg01115058	MFSD3	0.00961166	0.313781359
cg20565082		0.000420813	0.313705614
cg05412222	PACS2;PACS2	0.001038267	0.313606105
cg06855137	FAM38A	0.005790932	0.313597071
cg07380540		0.000420813	0.313545565
cg14152591	FAM107B	0.000420813	0.313532651
cg13707005	CUGBP2;CUGBP2;CUGBP2;CUGBP2	0.001038267	0.313467036
cg14037769		0.001038267	0.313342467
cg15636519	STAT4	0.001038267	0.313269688
cg21301258	RCAN1;RCAN1;RCAN1	0.000420813	0.313139302
cg16155207		0.000420813	0.313078726
cg00421624	SLC27A3	0.000420813	0.313027397
cg20485874	LRRC17;FBXL13;LRRC17;LRRC17;LRRC17;FBXL1	0.000420813	0.313023115
cg00972246		0.001569481	0.312930488
cg12692386	BAHCC1	0.001038267	0.312876511
cg23984176		0.001569481	0.312860344
cg20956114	PTPRN2;PTPRN2;PTPRN2	0.005790932	0.312837442
cg12032655	CD177	0.00961166	0.312827926
cg07855575		0.001569481	0.312811517
cg06925389		0.001038267	0.312787262
cg12563847		0.000420813	0.312773854
cg21746120	LRP5	0.000420813	0.312765732
cg06307913	PRDM8;PRDM8;PRDM8	0.004405484	0.312765614
cg26632171	MSGN1	0.00961166	0.312750703
cg18739367		0.001038267	0.312659116
cg04018023		0.001836098	0.312515517
cg26079706	SEPT9;SEPT9;SEPT9;SEPT9;SEPT9	0.00961166	0.312440816
cg04139359	RNF220	0.000636165	0.312400781
cg14692552	RINL	0.000420813	0.312386051
cg18866792	4-Mar	0.000420813	0.312334997
cg12550597	FAM129C	0.004405484	0.312291313
cg20360395		0.005790932	0.312163479
cg19186105		0.000420813	0.312152057
cg15612257		0.000420813	0.31211208
cg17300047	RCBTB2	0.000420813	0.312103661
cg02039171	CEBPE	0.003185744	0.31208129
cg14840966	CDC42BPB	0.007567702	0.312066363
cg19266014	MAD1L1;MAD1L1;MAD1L1	0.000420813	0.312056674
cg09007354	GLIS1	0.005790932	0.31195994
cg22618337	C10orf81	0.007567702	0.311936504
cg14495033	EHMT1	0.000532358	0.311847908
cg00436219		0.000420813	0.311820748
cg18597411	CUL1	0.000420813	0.311817016
cg03823431	SLC38A10;SLC38A10	0.001569481	0.311775328
cg21901847	NOS3	0.007567702	0.311751808
cg10233454	LRP1	0.000636165	0.311701298
cg12810800		0.001569481	0.311649444

cg08161931	PLEC1;PLEC1;PLEC1	0.000420813	0.311611967
cg14703454	HHLA2	0.002321262	0.311610314
cg26907472	TMCO7	0.001038267	0.311591968
cg07502892		0.000420813	0.3115856
cg21648069	ERC1;ERC1;ERC1;ERC1;ERC1	0.007567702	0.311573949
cg06353069	RAB17	0.002321262	0.311535353
cg21466229	SNTG1	0.002321262	0.311496514
cg26507422	CBFA2T3;CBFA2T3	0.000420813	0.311460397
cg21739584	BCL6;BCL6;BCL6	0.002321262	0.311408421
cg04265492	BAI2	0.000420813	0.311319563
cg06300222	ZFAND3	0.002321262	0.311223346
cg24215776		0.000420813	0.311152161
cg22687766		0.002321262	0.311129115
cg10035737	ADAM11	0.001038267	0.311099366
cg19649018		0.007567702	0.311083382
cg18796319	DPCR1	0.000420813	0.311072433
cg04871173	MAD1L1;MAD1L1;MAD1L1	0.001038267	0.311053284
cg22648375	PIK3CG	0.000636165	0.311023729
cg03571301	DUS1L	0.000420813	0.310984996
cg03977385		0.000420813	0.310956913
cg04337270	DHX15	0.004405484	0.310947594
cg11225330	UBE2J2;UBE2J2;LOC100128842;UBE2J2;UBE2J2	0.005790932	0.310927363
cg12022621	LAX1;LAX1;LAX1	0.001038267	0.310881074
cg00589002	TP73;TP73;TP73;TP73	0.000636165	0.310804594
cg11535839		0.005790932	0.310798856
cg00516966	ALDH3A1;ALDH3A1	0.000420813	0.310791795
cg18908419	VCL;VCL	0.002321262	0.310764828
cg01858712	SNX29	0.002321262	0.310742823
cg15224459	METRNL	0.007567702	0.310742359
cg10303721		0.000420813	0.31072353
cg24577455	PRKCZ;PRKCZ;PRKCZ	0.003185744	0.310678085
cg22850451	MAP1S	0.000420813	0.310677244
cg11478249	EXOC3	0.000420813	0.310672193
cg24199006	ITPRIP	0.002629558	0.310645604
cg14375205		0.001569481	0.310644826
cg07273304	ANXA13;ANXA13	0.001038267	0.310639987
cg24035043	HHIPL1;HHIPL1	0.000420813	0.310627961
cg25172145	PPM1F	0.000420813	0.310586872
cg05549611	NCRNA00114	0.000420813	0.310574288
cg10785340	H2AFY;H2AFY;H2AFY;H2AFY	0.000420813	0.310522466
cg15987934		0.000420813	0.310496404
cg16583987		0.002321262	0.310453452
cg13569051	GSN;GSN;GSN;GSN;GSN;GSN;GSN	0.005790932	0.310417536
cg02030275		0.001569481	0.310390317
cg21775279	XKR8;SMPDL3B	0.001038267	0.310387521
cg08887025	SMYD3;SMYD3	0.000420813	0.310384222
cg10859192	ATP5G2	0.000636165	0.310369465
cg02304580		0.000420813	0.31030781
cg06516476	NT5E	0.005790932	0.310296629
cg04821129	ATF6B;ATF6B	0.000420813	0.310285342
cg15323828	TMEM63A	0.000420813	0.310194255
cg22713958	LGALS3BP	0.005790932	0.310185078
cg14483391	HTR3D;HTR3D;HTR3D	0.001569481	0.310127258
cg03536664	UQCRC1	0.000420813	0.310126665
cg07762961	NLRC3	0.000420813	0.310122385
cg12999579		0.000420813	0.310084459
cg01505878		0.000420813	0.310077407
cg06647600		0.000420813	0.310058363
cg26039623	RBM47;RBM47	0.002321262	0.310054122

cg17565299	TMEM129;TMEM129	0.007567702	0.310026434
cg15953461		0.000420813	0.310017961
cg09595020	IQCE;IQCE	0.000636165	0.309977105
cg15489196	PXDN	0.000420813	0.309960481
cg20651456	RPL23AP53	0.000420813	0.309929489
cg19179572	HEXDC;C17orf101;C17orf101	0.001569481	0.309885513
cg14137939	CCDC89	0.000420813	0.309851477
cg11826008	CPNE1;CPNE1;CPNE1;CPNE1;CPNE1;RBM12;CP	0.002321262	0.309782349
cg04245026	ADARB2	0.000420813	0.309762046
cg17097293	PIP5K1C	0.000420813	0.309644566
cg25482983	COL11A2;COL11A2;COL11A2	0.001569481	0.309591972
cg01880929	CRIM1	0.000636165	0.309562416
cg07676145	CDH23	0.007567702	0.309524245
cg16211990		0.002321262	0.309516928
cg06892907		0.000420813	0.309498513
cg05962522		0.000420813	0.309497235
cg25963429	CREB3L1	0.000420813	0.309467823
cg23815312	ARHGAP22	0.005790932	0.309466806
cg16592101		0.005790932	0.309432169
cg11521563	MCF2L;MCF2L	0.000420813	0.309422087
cg02223351	CDH5	0.000466745	0.309311632
cg26840062	C5orf62	0.001569481	0.309263889
cg19180827	MSH2	0.001038267	0.309249849
cg19682835	FAM116B	0.000636165	0.309200172
cg02108620	ULK4	0.000420813	0.309181614
cg05854892		0.000420813	0.309124135
cg00870449	SLC8A1;SLC8A1;SLC8A1;SLC8A1	0.004405484	0.30910611
cg02397552	CLN5	0.000420813	0.309057221
cg26723162		0.000420813	0.309007583
cg11838898	ARHGEF4;ARHGEF4	0.004405484	0.308986
cg22676000	CEACAM6	0.004405484	0.308953625
cg01861740		0.000466745	0.308918011
cg14414903	HDAC4	0.001038267	0.308899355
cg19190519		0.003185744	0.308884644
cg01936091	ABLIM2;ABLIM2;ABLIM2;ABLIM2;ABLIM2;ABLIM2;/	0.000420813	0.308866192
cg20732076	TRERF1	0.001038267	0.308865128
cg21653105	TG;SLA;SLA	0.000420813	0.308821024
cg13987029	VOPP1	0.000420813	0.308818755
cg19419650	EVI5L;EVI5L	0.005790932	0.308790859
cg08615650	ADRBK1	0.000636165	0.308772976
cg13254586		0.001038267	0.308733538
cg23902994	ARHGEF10	0.001038267	0.30871597
cg04848957		0.000420813	0.308683938
cg26955290	ASGR2;ASGR2;ASGR2;ASGR2	0.00961166	0.308633886
cg18775149	ITGA2	0.000420813	0.308611472
cg16015285		0.007567702	0.308606447
cg16144314	ADARB2	0.004405484	0.308560312
cg16361867	GRIK4	0.007567702	0.308537877
cg09826895	DENND2D	0.003185744	0.308475588
cg10566660		0.001569481	0.308445199
cg05386769	TNS1	0.002321262	0.308388068
cg27635453		0.002321262	0.308373241
cg00854273		0.000420813	0.308352426
cg09804858	TRERF1	0.000420813	0.308343895
cg02769104	FAM46B	0.000420813	0.308334081
cg23754934	MS4A4A	0.002321262	0.308315169
cg26332052	UNK	0.002321262	0.308307726
cg04510459		0.004405484	0.308300508
cg05264101	FLJ45079	0.001569481	0.308284432

cg12361772	STX8	0.000420813	0.308261779
cg24040155	LRP5	0.004405484	0.308219929
cg10932907	TMEM45B	0.00961166	0.308212623
cg05899984		0.001038267	0.308122078
cg26580761	MAD1L1;MAD1L1;MAD1L1	0.000636165	0.308096954
cg15076811		0.000420813	0.308090475
cg24750513	BIN1;BIN1;BIN1;BIN1;BIN1;BIN1;BIN1;BIN1;B	0.001038267	0.308033991
cg03888814	DNAH7	0.003185744	0.308018123
cg18281910	EDARADD;EDARADD	0.00961166	0.308014036
cg15561778	GAB1;GAB1	0.000420813	0.308008584
cg24101039	SP4	0.001038267	0.307947987
cg13436378	DYSF;DYSF;DYSF;DYSF;DYSF;DYSF;DYSF;DYSF	0.000636165	0.307921857
cg05609039		0.000420813	0.30791873
cg12218747		0.000420813	0.307899196
cg05136737		0.002321262	0.307879359
cg22478679	ADPRHL1;ADPRHL1	0.002321262	0.307848433
cg26747290		0.000420813	0.307788984
cg22633932	SETD7	0.000420813	0.307787118
cg21338225	C7orf41	0.001038267	0.307750256
cg18815120	EGLN1	0.007567702	0.307742516
cg05073720	PHLPP1	0.000420813	0.307692195
cg02788021	KATNAL1	0.000420813	0.307689652
cg23025804	SVIL;SVIL	0.004405484	0.307683006
cg01419914	BAHCC1	0.003185744	0.307659808
cg17002510	ARHGEF10	0.001038267	0.307644241
cg12743398	SULT1A2	0.001038267	0.307638855
cg01226748	PXMP2	0.000420813	0.307603868
cg15006828		0.007567702	0.307599789
cg04951819	CCDC77;CCDC77;CCDC77;CCDC77	0.001038267	0.307572712
cg11724055	HNRNPR;HNRNPR;HNRNPR;HNRNPR	0.000420813	0.307564618
cg17769009	RPL23AP53	0.000420813	0.307524903
cg03531687	RARB;RARB	0.000636165	0.307520794
cg19081470		0.000420813	0.307434253
cg21128953	MTUS2;MTUS2	0.003185744	0.307373378
cg07956000		0.003185744	0.307373275
cg04028874	ZNF683;ZNF683	0.002629558	0.307369177
cg15089620	ARHGAP26;ARHGAP26	0.000420813	0.307239682
cg05582286		0.000420813	0.307238602
cg18757228	SCARA5	0.000420813	0.307194471
cg21160357	TNS3	0.000420813	0.307190332
cg00155732	SLC2A5;SLC2A5;SLC2A5	0.001569481	0.307188423
cg11944933	FAM167A	0.001038267	0.30716027
cg05242065	C2orf89	0.004405484	0.307104587
cg04837616	FLJ45079	0.001038267	0.307070103
cg22924563		0.000420813	0.307017359
cg01561157	TBCD	0.001569481	0.307007762
cg02480666	GJA4	0.007567702	0.306879763
cg02442640	SEPT9;SEPT9;SEPT9;SEPT9;SEPT9;SEPT9	0.005790932	0.30685038
cg23230158	GALNT2	0.000420813	0.306841213
cg26906737	TMEM206	0.000420813	0.30683688
cg05075308	IRF2	0.001569481	0.306812964
cg26874367		0.000420813	0.30673707
cg08363067	ABCC1;ABCC1;ABCC1;ABCC1;ABCC1	0.000636165	0.306728043
cg02472906	CA5A	0.000420813	0.306724902
cg03620886	ZGLP1	0.000636165	0.306581469
cg02947125		0.001569481	0.306518802
cg21880392	FRZB	0.000636165	0.306472716
cg15306012	EXOC3L	0.001038267	0.306465872
cg00262054	MYO18A;MYO18A	0.000420813	0.306449216

cg13630878	MTMR15	0.000420813	0.306445959
cg05233155		0.000420813	0.306362386
cg11452501	LY6G5C	0.000420813	0.306301792
cg19353947	ZEB2;ZEB2;ZEB2	0.002321262	0.306264121
cg14733031	B3GNT3	0.000420813	0.306238046
cg09273716		0.005790932	0.306207871
cg06872019		0.007567702	0.306184041
cg15706657	GPR31	0.000719053	0.306171611
cg09154256	RAB1A;RAB1A	0.003185744	0.306147402
cg02551745		0.002321262	0.306136341
cg24927174	SHC1;SHC1;SHC1;SHC1	0.007567702	0.306127254
cg19097280	LRRN4	0.001569481	0.306114557
cg24152264		0.000420813	0.306042824
cg07054502	ANKRD11	0.000420813	0.305991587
cg04879645	LMF1	0.002321262	0.305984727
cg11810135	FCRL1;FCRL1;FCRL1	0.000420813	0.305942283
cg08795084	DEPDC1B;DEPDC1B	0.000420813	0.305940709
cg15452381	HIVEP3;HIVEP3	0.001038267	0.305922541
cg00101457	CUGBP2;CUGBP2;CUGBP2;CUGBP2	0.000636165	0.305915633
cg10104480	ZFHX3;ZFHX3	0.000636165	0.305912781
cg12821702		0.000636165	0.305907054
cg07589034		0.00961166	0.305906883
cg09026253	IFITM1	0.00961166	0.305891189
cg16277071		0.000420813	0.305858857
cg23427362	CACNA1C;CACNA1C;CACNA1C;CACNA1C;CACN	0.000420813	0.30583245
cg14641757	C21orf96;RUNX1	0.000420813	0.305817063
cg04261556	ITGB8	0.000420813	0.305767264
cg21877464	SPARC	0.001569481	0.305758779
cg26329274	ITK	0.007567702	0.305702803
cg15302379	KAZALD1;KAZALD1	0.002321262	0.305696568
cg06362313	GAPDH	0.000420813	0.305673271
cg04369903		0.001038267	0.305664142
cg12962778	ZNF385A;ZNF385A;ZNF385A;ZNF385A	0.007567702	0.305646175
cg08875605	ETV5	0.001569481	0.305593099
cg24575128	NISCH	0.007567702	0.305555661
cg09504131	STK24;STK24	0.003185744	0.305544654
cg27526649		0.005790932	0.305537474
cg18281939	LHFPL2	0.000636165	0.305466483
cg03252770		0.000636165	0.305457522
cg00945443	MTHFD1L	0.000420813	0.305416377
cg14895029	GNA12	0.000420813	0.305399425
cg09803959	RPTOR;RPTOR	0.001038267	0.305395845
cg00502597	FGGY;FGGY	0.000420813	0.305377361
cg14139311		0.00961166	0.30534794
cg14861545		0.001569481	0.305314536
cg15507500	SLC27A1	0.003185744	0.305271006
cg26120813		0.000636165	0.305244173
cg12434889	PTPRN2;PTPRN2;PTPRN2	0.004405484	0.305231786
cg12592276		0.002321262	0.305182935
cg16928487	SREBF1;SREBF1	0.000420813	0.305176967
cg18062205	KCNG1	0.000420813	0.305174241
cg20952652	UXS1	0.000420813	0.305163715
cg04364752	GALNS	0.003185744	0.305083539
cg24924502	AATK	0.003185744	0.304930143
cg18396992		0.00961166	0.304920511
cg01522095	GGA2	0.000420813	0.304898384
cg13629388	FAM120B	0.000420813	0.304871224
cg07711397	SH2D4B	0.000420813	0.304870458
cg26217846	DIAPH1;DIAPH1	0.004405484	0.304766938

cg01046511	KDM6B	0.001777515	0.304705882
cg10122452	SMARCB1;SMARCB1	0.001569481	0.304701505
cg06270401	DYRK4	0.000420813	0.304677388
cg19650253	PTGDR	0.004405484	0.304657855
cg01097897	PPM1H	0.000420813	0.304629892
cg13377102	KCNAB3	0.00961166	0.304622096
cg23288563	NDRG2;NDRG2;NDRG2;NDRG2;NDRG2;NDRG2;N	0.000420813	0.304604297
cg15151059	SETDB1;SETDB1	0.003185744	0.304596856
cg26035892		0.000420813	0.304580293
cg22344162	CREG1	0.002321262	0.304533973
cg06779553	TOX	0.000636165	0.304510424
cg04120413	HLTF;HLTF	0.000420813	0.304508978
cg09774649	C4orf23	0.000420813	0.304426337
cg11308170	SNORA1;SNORA8;SNORD5;SNORA18	0.000420813	0.304383489
cg01135648	DCAF4L1	0.003185744	0.304314128
cg02538833	CLSTN1;CLSTN1	0.001038267	0.304301856
cg17919667	FGFR4;FGFR4	0.002321262	0.304292836
cg19410609		0.000420813	0.304273142
cg01455981	UBXN10	0.003185744	0.304268033
cg04445427		0.001038267	0.304253892
cg16616918	SMYD4	0.003185744	0.304242726
cg04903159		0.000420813	0.30421086
cg00534022		0.000420813	0.304204902
cg04372182		0.000420813	0.304186402
cg12759523	CYFIP1	0.001569481	0.30418211
cg17112975	SEPT9;SEPT9;SEPT9;SEPT9;SEPT9	0.000636165	0.304177203
cg09665332	SGIP1	0.001569481	0.304127607
cg08929197	ZDHHC14;ZDHHC14	0.000420813	0.304072684
cg26738160	RAPSN;RAPSN	0.003185744	0.304023622
cg22959409	LRRC33	0.001038267	0.303942142
cg10341242	ADCY7	0.002321262	0.303919186
cg08822625	NEDD9	0.000420813	0.303890694
cg02522367	LCN6	0.001569481	0.303875167
cg05824594	CACNA1C;CACNA1C;CACNA1C;CACNA1C;CACN	0.000420813	0.303860367
cg07964097		0.000420813	0.303859487
cg21971799		0.000636165	0.303837414
cg02290880	CTSE;CTSE	0.000636165	0.303815384
cg00158770	CSGALNACT1;CSGALNACT1;CSGALNACT1	0.004405484	0.303725116
cg26684673	UBASH3B	0.000636165	0.303713421
cg19210858	ELF2	0.007567702	0.303669069
cg04071430		0.000420813	0.303641917
cg24592782	CTNNBIP1;CTNNBIP1	0.000420813	0.303617128
cg06615154	S100A3	0.000420813	0.303613334
cg10521711		0.000466745	0.303586496
cg02466892		0.001038267	0.303573025
cg09228833	ZNF217	0.001038267	0.30357033
cg19382136		0.002629558	0.30347904
cg19107511		0.004405484	0.303478272
cg00917471	LEPRE1;LEPRE1	0.000420813	0.30347679
cg06372654	IMPDH1;IMPDH1;IMPDH1;IMPDH1;IMPDH1;IMPDH	0.000420813	0.303475711
cg00384553		0.000420813	0.303472556
cg15420926	EFR3A	0.004405484	0.303471442
cg05077231	MLNR	0.005790932	0.303430831
cg11791710	DOCK1	0.000420813	0.303417122
cg14758065		0.000636165	0.303385343
cg03225003	NPHP4	0.000420813	0.303358051
cg00239117	UBE2F	0.000420813	0.303346412
cg16672637	FOXJ1	0.000420813	0.303341667
cg08123339		0.005790932	0.303322842

cg23416081	FKBP5	0.002321262	0.303285124
cg19881557		0.000420813	0.303282516
cg08463932	LAPTM5	0.000420813	0.303260381
cg03889700	ADD1;ADD1;ADD1;ADD1	0.00961166	0.303170869
cg07574163		0.004405484	0.303161641
cg08580187	NECAB2	0.002321262	0.303155574
cg08477158	BCAS3;BCAS3	0.000636165	0.303048591
cg16499923	DENND3	0.007567702	0.302729062
cg15009090		0.000636165	0.302686022
cg06976521	SNORD125;AP1B1;AP1B1;AP1B1	0.000420813	0.302677912
cg00812761	SCFD2	0.002321262	0.302657114
cg25688583	TCF12;TCF12;TCF12;TCF12;TCF12	0.000466745	0.302636081
cg20482280	MTA1	0.007567702	0.302611627
cg04498801	MFSD10;MFSD10	0.000420813	0.302527494
cg26250585	MS4A6A;MS4A6A;MS4A6A	0.000420813	0.302513046
cg11014468	DCP1A	0.000420813	0.302482369
cg16681421		0.003185744	0.302476167
cg25156198	CDK6;CDK6	0.000420813	0.302473238
cg02750743	TESC;TESC;TESC	0.005790932	0.302464183
cg05869699	TSPAN14;TSPAN14	0.000420813	0.302451354
cg06985664	MET;MET	0.000420813	0.30244658
cg02543462	IL1RN;IL1RN;IL1RN;IL1RN	0.007567702	0.302411363
cg11333968	PPFIBP2	0.002321262	0.302394708
cg12271317	LEF1;LEF1;LEF1;LEF1	0.000636165	0.302383739
cg12798970		0.00961166	0.302324203
cg13698860	BIK	0.000466745	0.302293268
cg04618171	HPCAL1;HPCAL1	0.001038267	0.302250711
cg06469570	ACOX3;ACOX3	0.003185744	0.30216628
cg14829516	THRA;NR1D1	0.000420813	0.302159833
cg26771582	FBXO31;FBXO31	0.000636165	0.302157107
cg21186560		0.004405484	0.302145403
cg26288715	MYB;MYB;MYB;MYB;MYB;MYB;MYB;MYB	0.000420813	0.302091178
cg09888186	RGL1	0.000636165	0.302051925
cg06899483		0.000719053	0.302043934
cg09123760	DIABLO;DIABLO;DIABLO;DIABLO	0.000420813	0.302020345
cg09510202	PHACTR1	0.000636165	0.302018472
cg02824983		0.002321262	0.301980528
cg01924561	SLC2A1	0.005790932	0.301974369
cg16983110	PTPRK;PTPRK	0.001569481	0.301971168
cg25865120	DUT;DUT;DUT	0.000420813	0.301948758
cg23251200		0.001038267	0.301935645
cg04590303	IFT140	0.000420813	0.301896537
cg03427849	RWDD4A;C4orf41;C4orf41	0.003185744	0.301874784
cg13673960	TACC3	0.005790932	0.301869516
cg01827761	KLHDC7A	0.003185744	0.301862009
cg18909389	CLIC1	0.000420813	0.301814772
cg18915456		0.000420813	0.301803666
cg08490349	MPRIP;MPRIP	0.000420813	0.301793438
cg10532310	FLYWCH1;FLYWCH1	0.002321262	0.301769341
cg09179248	PRKCZ;PRKCZ;PRKCZ	0.002321262	0.301741995
cg04956382	MYOT;MYOT;MYOT;MYOT	0.001038267	0.301724471
cg15072619	SLC39A12;SLC39A12	0.005790932	0.301683049
cg02359160	C1orf228	0.007567702	0.301640304
cg13643509	RPTOR;RPTOR	0.000420813	0.301624076
cg04369306		0.001038267	0.301585253
cg24300845		0.002321262	0.30157167
cg20476058	LIMS2	0.000420813	0.301553835
cg06244417	FCN1	0.002321262	0.301538795
cg13665021		0.000420813	0.301530662

cg21816393		0.000420813	0.301524419
cg11249762	SEPT9;SEPT9;SEPT9;SEPT9;SEPT9	0.001569481	0.301521539
cg00736984	PLEC1;PLEC1;PLEC1	0.001569481	0.301518146
cg09427016	ACSF3;ACSF3;ACSF3	0.003185744	0.301493341
cg01043583	MSRA;MSRA;MSRA	0.000420813	0.301486311
cg12093241	ARID1B;ARID1B;ARID1B	0.003185744	0.301446088
cg16344173	MAD1L1;MAD1L1;MAD1L1	0.000420813	0.301444867
cg26891210	CTSS	0.003185744	0.30140266
cg22431093	FGD4	0.000420813	0.301393259
cg09234973	PAX8;PAX8;PAX8;PAX8;LOC440839;PAX8	0.000420813	0.301388723
cg11010552	ACSL6;ACSL6	0.001569481	0.301383222
cg03656863		0.003185744	0.301349818
cg06450952	C8orf56	0.000420813	0.301339066
cg14835981	PRKAR1B;PRKAR1B;PRKAR1B;PRKAR1B;PRKAR	0.004405484	0.301311983
cg05570654	HDAC4	0.000420813	0.301234398
cg14801864	BFSP1	0.003185744	0.301219184
cg23898497	PVT1	0.003185744	0.301219034
cg00450651		0.002321262	0.301170512
cg02895948	PLXNA2	0.001569481	0.301168521
cg02217184	GPR18;GPR18;UBAC2;UBAC2;UBAC2	0.00961166	0.301160797
cg14470121	IFT140;TMEM204	0.003185744	0.301136785
cg07750741	CYFIP2;CYFIP2	0.000420813	0.301094784
cg00081919		0.000420813	0.301086444
cg26188726	TRIM72	0.000420813	0.301048271
cg09435170		0.00961166	0.301048001
cg07617814	ZNF217	0.000420813	0.301030361
cg16946840	MED12L	0.003185744	0.301003609
cg07298299	GYLTL1B;GYLTL1B	0.000420813	0.300994606
cg01164574		0.001569481	0.300986661
cg26307814	PIK3CD	0.004405484	0.300960241
cg02550151	BRD1;BRD1	0.002321262	0.300929891
cg16469740	HDAC9;HDAC9	0.001038267	0.300918628
cg21741708		0.007567702	0.300892643
cg04815334	NADSYN1	0.005790932	0.30089037
cg20847701	PVT1	0.002321262	0.300875824
cg18402166	LOC146880;LOC146880	0.005790932	0.300853678
cg00288844	TXNDC11	0.000420813	0.300811026
cg06033531	FAM193B;FAM193B	0.005790932	0.300805383
cg10254970		0.000420813	0.300786398
cg17830682	LGR6;LGR6;LGR6	0.003185744	0.300780754
cg11071202		0.003185744	0.300777752
cg02059268		0.007567702	0.300747498
cg24544082	SPTBN1	0.002321262	0.300718454
cg09186101	SLC16A3;SLC16A3;SLC16A3	0.002321262	0.300667299
cg27037305	BRF1;BRF1	0.001173833	0.300656179
cg12104022	FERMT2;FERMT2;FERMT2	0.000420813	0.300649061
cg26140802		0.003185744	0.300638578
cg16589830		0.000420813	0.300611759
cg00658405	ADAMTSL5	0.001569481	0.300560294
cg09202659	JARID2	0.000420813	0.300491943
cg16346422	SLC22A18;SLC22A18AS	0.000420813	0.300443213
cg07466788	SLC16A3;SLC16A3;SLC16A3	0.000420813	0.300439813
cg09506001	ADPRHL2	0.000636165	0.300409664
cg13175282	COMT;COMT;COMT;COMT	0.000420813	0.300279241
cg03822873	AHCYL2;AHCYL2	0.00961166	0.300277837
cg07570113	LOC644649	0.005790932	0.300247912
cg05426662		0.000420813	0.300245135
cg14511156	OSCAR;OSCAR;OSCAR;OSCAR;OSCAR;OSCAR;	0.000420813	0.30023874
cg03690812	SH2B3	0.001569481	0.300209541

cg26917640		0.007567702	0.300162933
cg22143766	WNT5A	0.001173833	0.300159983
cg22005565	SLFNL1;SLFNL1	0.000636165	0.300148809
cg17112266	ABCB8	0.000420813	0.300144367
cg07701837	MAP2;MAP2;MAP2;MAP2	0.002321262	0.300138061
cg16392860		0.002321262	0.300124849
cg27416489	KIF5C	0.000420813	0.300106751
cg13488011		0.004405484	0.300081412
cg21805118	DIABLO;DIABLO;DIABLO;DIABLO	0.000420813	0.300053679
cg07270021		0.000420813	0.300001889
cg07119830	TRIM8	0.00961166	-0.300041469
cg26597982	TSNARE1	0.000420813	-0.300105054
cg02774855	CLIP4	0.000420813	-0.300122644
cg27118761	KCNC1;KCNC1	0.007567702	-0.300288158
cg00113020	LILRB4;LILRB4	0.004405484	-0.300694566
cg13257636	CLIP4	0.000420813	-0.300901868
cg07512814	LDLR	0.007567702	-0.300935958
cg13416213		0.005790932	-0.30110643
cg20804700	MCOLN2	0.003185744	-0.301626903
cg09294077	GPHN;GPHN	0.005790932	-0.301713067
cg00409309	HLA-DOB	0.007567702	-0.30189799
cg11206634	SFT2D3	0.002321262	-0.302343374
cg03405515	MAP9	0.002321262	-0.302426111
cg13356117	TTC12	0.004405484	-0.30314003
cg25333258	CAMK2B;CAMK2B;CAMK2B;CAMK2B;CAMK2B;CA	0.002321262	-0.303228449
cg22513924	NMNAT2	0.005790932	-0.303994746
cg16296829	KCNN3;KCNN3	0.002321262	-0.304040121
cg15861196		0.003185744	-0.304389209
cg02935904	TTC22;TTC22	0.001038267	-0.304422521
cg06237487	SLC6A16	0.002321262	-0.304693428
cg00073780	PKIB;PKIB;PKIB;PKIB;PKIB	0.001038267	-0.304826914
cg20598560	SGPP2	0.002321262	-0.304956257
cg13136933	LOC283663	0.00961166	-0.305210995
cg17285225	OXTR;OXTR	0.007567702	-0.305602547
cg19882132		0.000420813	-0.305691195
cg02539402		0.000420813	-0.305697413
cg10137084	CACNA2D3	0.007567702	-0.30634587
cg06161738	GALNT11	0.007567702	-0.306510436
cg23840223	NMU	0.001569481	-0.306688887
cg02466321	ANKRD34B;ANKRD34B	0.001569481	-0.306898822
cg12306156	THRB;THRB;THRB	0.000420813	-0.306904939
cg07563944	CBFA2T3	0.000636165	-0.306906604
cg14583606	SLC1A1	0.002629558	-0.306915336
cg00682125	CDKAL1	0.000420813	-0.307099609
cg16086373	TMEM200C	0.005790932	-0.307356211
cg11017382	MAST4;MAST4	0.007567702	-0.307442448
cg12277366	TCEA1;TCEA1	0.001569481	-0.308013009
cg02896872	ITPKB	0.007567702	-0.308106129
cg05048199	FBXL2;FBXL2	0.00961166	-0.308492112
cg18240143	C14orf39	0.001569481	-0.308497122
cg05206657		0.003185744	-0.309707661
cg17157198	IKZF1	0.007567702	-0.310041947
cg19529326	EPB41L4A;FLJ11235;EPB41L4A	0.000466745	-0.310265272
cg10911619	TBC1D12	0.000420813	-0.310375059
cg06313119	FGF14	0.001569481	-0.310695676
cg26381364	C12orf42;C12orf42	0.007567702	-0.310725183
cg06562372	ABCB1	0.000420813	-0.310961091
cg26364899	PPARG;PPARG;PPARG	0.000636165	-0.311031774
cg19873491	FAM149A	0.002321262	-0.311105285

cg15958828	FOXP1;FOXP1	0.000420813	-0.311190406
cg15410236	ARID3A	0.000420813	-0.312274579
cg25068347	ETS1;ETS1;ETS1	0.003185744	-0.312432524
cg12112870	E2F5;E2F5;E2F5	0.000636165	-0.31250092
cg17052813	GPR37	0.000636165	-0.31256259
cg15378605	CHST15	0.004405484	-0.312564207
cg10353539	TTC12	0.004405484	-0.312597094
cg21062931	DHCR24	0.002321262	-0.312753896
cg22520644	WDR17;WDR17	0.001569481	-0.313080349
cg16289355	FAM149A	0.002321262	-0.313088982
cg03710481	PKIB;PKIB;PKIB;PKIB;PKIB	0.001569481	-0.313090049
cg03339817	SFT2D3	0.000420813	-0.31313059
cg06945523	SFTA3	0.000420813	-0.313239051
cg14169158	FAM78B	0.002321262	-0.313274241
cg11021317		0.000420813	-0.313661368
cg11012046	GLB1L2	0.00657282	-0.313739409
cg08163918	SHMT2;SHMT2;SHMT2;SHMT2;SHMT2;SHMT2;SH	0.007567702	-0.31449094
cg03328673		0.007567702	-0.314797659
cg06335343	TMEM20;TMEM20	0.00961166	-0.315074556
cg22152931	PRKRIP1	0.001569481	-0.315087155
cg24631970	XKR6	0.000420813	-0.31521985
cg02048922	BAI1	0.003185744	-0.315325613
cg17037963		0.000420813	-0.315362029
cg22685009	MRPS27	0.000420813	-0.315394118
cg11777523	GPR148	0.007567702	-0.315456226
cg18175036	MAST4;MAST4	0.002024793	-0.315993671
cg12816198	IRF5;IRF5;IRF5;IRF5;IRF5;IRF5	0.003185744	-0.316215456
cg19631815	DTX1	0.000636165	-0.316251784
cg09197279		0.005790932	-0.316282203
cg11156873	LPCAT1	0.000420813	-0.316357143
cg12387700	MCOLN3;MCOLN3	0.002321262	-0.316369692
cg17774764	SPIB	0.005790932	-0.316551204
cg05488043	P2RY1;P2RY1	0.001569481	-0.316663068
cg01604404		0.001569481	-0.316829825
cg23231729	THEM4	0.00961166	-0.316872754
cg21197594	EHMT1;FLJ40292;EHMT1	0.000420813	-0.3173363
cg02007288	GTF2E2	0.000420813	-0.317667641
cg15944060		0.004405484	-0.318556706
cg00418880	HS6ST3	0.001569481	-0.318744207
cg02668248	KLF2	0.007567702	-0.318756154
cg00893424	SACS	0.007567702	-0.319304223
cg00631837	PHF21B;PHF21B	0.007567702	-0.319353239
cg02452966	RYR1;RYR1	0.00961166	-0.319396301
cg12748332	MPPED2	0.002321262	-0.319471564
cg11769456	SNED1	0.007567702	-0.319488692
cg12243375	GUCY1A2	0.002321262	-0.319917111
cg06547386	MPPED2	0.005790932	-0.320104865
cg13378394	KCNC3	0.000636165	-0.320434758
cg24789487	FAM171B;FAM171B	0.000420813	-0.320528759
cg04741853		0.007567702	-0.320752135
cg08726863	SFT2D3	0.001038267	-0.321598328
cg08055910	FAM149A	0.004405484	-0.32227123
cg09287328		0.001569481	-0.322363727
cg19053239	KIF5C	0.000420813	-0.322381944
cg10865087	BICC1	0.000420813	-0.322671492
cg08808128	CLIP4;CLIP4	0.000636165	-0.322757892
cg05919561	TTLL7	0.000636165	-0.322757998
cg04657224	XKR6	0.003185744	-0.322998424
cg01823925	ADAM19	0.002321262	-0.323203554

cg23249922		0.001038267	-0.323341631
cg05022673	DST;BEND6	0.001777515	-0.323627337
cg16296017		0.000420813	-0.323989214
cg01646461	CCR6;CCR6	0.00961166	-0.323999801
cg27207756		0.003185744	-0.324205457
cg05549575	C17orf87	0.000420813	-0.324338272
cg15138339	COASY;COASY;COASY;COASY;COASY	0.000420813	-0.325175988
cg03521656	TMC5;TMC5;TMC5;TMC5	0.002321262	-0.325652546
cg27375072	SMPDL3A	0.001569481	-0.325920849
cg04748988	PPARG;PPARG;PPARG	0.007567702	-0.326044394
cg26685735	COL4A3;COL4A4;COL4A3;COL4A4;COL4A3;COL4	0.001038267	-0.326359264
cg04757345	C4orf29	0.000420813	-0.326379586
cg12258785	THRB;THRB;THRB	0.002321262	-0.326575564
cg15931721	C1QL2	0.003613402	-0.327052435
cg15446670	ABCB1;RUNDC3B;RUNDC3B;RUNDC3B	0.00961166	-0.327287259
cg08551725	SCRN1;SCRN1;SCRN1;SCRN1	0.000420813	-0.327461979
cg00677986	PAWR	0.000420813	-0.327516999
cg10880973	ZDHHC14;ZDHHC14	0.000420813	-0.327692167
cg08232264	FAM164A	0.000420813	-0.328027653
cg16142306	UCHL1;UCHL1	0.000420813	-0.328039474
cg09277575	AKR1D1	0.001569481	-0.32808963
cg18166862	FAM78B	0.001038267	-0.32844028
cg25375340	MCOLN2	0.000420813	-0.329031483
cg04324308	COL4A3;COL4A4;COL4A3;COL4A4;COL4A3;COL4	0.005790932	-0.329151686
cg14375890		0.004405484	-0.329505361
cg15847198	GUCY1A2	0.000420813	-0.329621579
cg03730703	PEA15	0.007567702	-0.32963333
cg23934477	TCOF1;TCOF1;TCOF1;TCOF1;TCOF1;TCOF1	0.002321262	-0.329665824
cg18817459	SOBP	0.000420813	-0.330023651
cg19954286	CCR6;CCR6	0.00961166	-0.330458804
cg13603533		0.00961166	-0.330627811
cg27275352	CILP2	0.003185744	-0.33084501
cg00323305	THRB;THRB;THRB	0.002321262	-0.330920878
cg16419066	MGC2889;HRASLS	0.001173833	-0.331008911
cg05302420	ENTPD7	0.000636165	-0.331133188
cg00393585	C4orf39;TRIM61	0.005790932	-0.331614588
cg09601770	DPP4;DPP4	0.007567702	-0.33170705
cg13702005	C12orf42;C12orf42	0.002321262	-0.331859557
cg05253577	WWC1;WWC1;WWC1	0.00961166	-0.331940127
cg04442576	SLC35F1	0.003185744	-0.331941856
cg02445447	FHL2;FHL2;FHL2;FHL2;FHL2;FHL2	0.000420813	-0.332507484
cg19039464	C4orf31;C4orf31	0.001038267	-0.332663927
cg17838026	KCNC3	0.001038267	-0.333183218
cg11881599	CLLU1OS;CLLU1;CLLU1	0.000420813	-0.333467332
cg09915835	UGT8	0.001038267	-0.333475178
cg04168675	DOCK10	0.001173833	-0.333875986
cg10739095	CLSTN2	0.001569481	-0.334213824
cg10248492		0.001569481	-0.334277709
cg16787199	BCL2L10	0.002321262	-0.334386556
cg25033076	MPPE1	0.002321262	-0.334587847
cg21774136	RERE;RERE	0.003185744	-0.334649428
cg27236875	ERBB4;ERBB4	0.003185744	-0.334705984
cg16235962	CXCR5	0.000420813	-0.335197003
cg21303011	THRB;THRB;THRB	0.000636165	-0.335242957
cg12212657	LRRN1	0.000420813	-0.335651732
cg00674706		0.001569481	-0.335668385
cg19224713	SAMD12;SAMD12	0.000636165	-0.336073837
cg19396666		0.007567702	-0.336489006
cg15939347	TSNARE1	0.004405484	-0.337289518

cg21902325	LHFPL2	0.005790932	-0.337473123
cg00950381	QRICH2	0.000420813	-0.337655122
cg15129663	FMO1	0.001038267	-0.337896914
cg19753526	FHL2;FHL2;FHL2;FHL2;FHL2;FHL2;FHL2	0.003185744	-0.339308935
cg27205687	GUCY1A2;GUCY1A2	0.004405484	-0.339423234
cg00342532	KCNC3;KCNC3	0.004997918	-0.339454525
cg24974423	HS6ST3	0.000636165	-0.339584127
cg01510903	KRT18;KRT18	0.004405484	-0.339624655
cg01300410	TMEM20;TMEM20	0.004405484	-0.339812743
cg06477663	LCP1	0.002321262	-0.340098159
cg22982654		0.00961166	-0.34056374
cg17958423	ZNF215	0.002321262	-0.341363022
cg21868774	FAM78B	0.001569481	-0.341492018
cg24458474	GRID1	0.001038267	-0.341515938
cg08951903	ZNF239;ZNF239;ZNF239	0.004405484	-0.341777012
cg20043258	CDC42BPB	0.001173833	-0.342173121
cg16031136	LYN;LYN	0.000420813	-0.342180283
cg18533282	SPATA18;SPATA18	0.005790932	-0.342371607
cg27448015	SMPDL3A	0.001569481	-0.34250588
cg16735130		0.004405484	-0.342552282
cg25912827	DPP4;DPP4	0.007567702	-0.343899668
cg13205384	ANO5;ANO5	0.001038267	-0.343972352
cg06177053	DTD1;DTD1	0.004405484	-0.344336236
cg14412134	MTHFD1	0.00961166	-0.345335062
cg13626582	LOC283663	0.008581131	-0.345529085
cg12260653	TMEM20;TMEM20	0.003613402	-0.346166039
cg16703576	RUNDC3B;RUNDC3B;RUNDC3B;ABCB1;RUNDC3	0.005790932	-0.346197877
cg13919148	INADL;INADL	0.005790932	-0.346308296
cg08961187	MDGA1	0.005790932	-0.346378193
cg19908577	UGT8	0.001569481	-0.346568787
cg12693702	MIR148A	0.002321262	-0.346893689
cg17229371	LPCAT1	0.002321262	-0.347102727
cg13429095		0.007567702	-0.347155128
cg16643706	C5orf13;C5orf13	0.002321262	-0.347162814
cg22117805	PRR5L;PRR5L;PRR5L;PRR5L	0.003185744	-0.347220477
cg27510257	CARS2	0.000420813	-0.347381401
cg08622098	SIPA1L3	0.000420813	-0.348000039
cg26789732	KCNB2	0.000420813	-0.348230249
cg21383151	TBC1D12	0.005790932	-0.348653953
cg24843474	RGS7	0.007567702	-0.349319094
cg07508229	TBC1D12;TBC1D12	0.000420813	-0.34963756
cg14851108	GRID1	0.000420813	-0.349660543
cg23604012	SGPP2	0.002321262	-0.349673941
cg24593832		0.000420813	-0.349941883
cg20755651	SMOC2;SMOC2	0.007567702	-0.350444775
cg23255835	CLIP4	0.002321262	-0.351113651
cg08343042	TMEM163	0.001569481	-0.35120628
cg07301433	CYP1B1	0.003185744	-0.351824422
cg16429499	NLRC3	0.000420813	-0.351949128
cg00900735	GRB2;GRB2	0.000466745	-0.352166136
cg22750001	MPEG1	0.000420813	-0.352533383
cg14508705	DNM3;DNM3	0.001569481	-0.352598081
cg12145080	SGPP2	0.007567702	-0.35259936
cg23889440	MAPK4	0.000636165	-0.352753921
cg19544662	FOXI3	0.00961166	-0.352904921
cg05290695	SAMD12;SAMD12	0.003613402	-0.352991501
cg04922681	TNFSF11;TNFSF11	0.003185744	-0.354634763
cg17134153	FCRL3	0.000420813	-0.355247972
cg17607231	SP140;SP140	0.000420813	-0.355267469

cg27236007	RASGRP3;RASGRP3;RASGRP3	0.000420813	-0.355325527
cg16523158	CCR6;CCR6	0.000420813	-0.355414491
cg07052390	IGSF9B	0.001569481	-0.355655336
cg15461105		0.004997918	-0.356712713
cg09132102		0.001038267	-0.35749011
cg05478631	T	0.000420813	-0.357612753
cg08844849	SIGLEC10;SIGLEC10;SIGLEC10;SIGLEC10;SIGLE	0.000420813	-0.357640142
cg16272981	LPCAT1	0.000420813	-0.357775173
cg08229018	PRSS12	0.000420813	-0.359466289
cg15418826	KIF21A	0.003185744	-0.359528156
cg23679819	RNMT	0.000636165	-0.359606829
cg11430157	SLC35F1;SLC35F1	0.002321262	-0.359682607
cg16148346		0.002321262	-0.360743997
cg17187287	UGT8	0.004997918	-0.361446161
cg03562952	LPCAT1	0.000636165	-0.361709102
cg10512951	LMBRD1	0.007567702	-0.362021268
cg10507275	LRRN1;LRRN1	0.004405484	-0.362471815
cg25834419	GOLSYN;GOLSYN;GOLSYN;GOLSYN;GOLSYN;G	0.000420813	-0.362686635
cg00254017	MAP3K1	0.007567702	-0.362765748
cg00688963	THRB;THRB;THRB;THRB;THRB;THRB	0.001038267	-0.36301485
cg20966357	C6orf10	0.000420813	-0.363174941
cg14255337	KDM2B;KDM2B	0.000420813	-0.363295403
cg25208863	GOLSYN;GOLSYN;GOLSYN;GOLSYN;GOLSYN;G	0.001038267	-0.364003936
cg13560871	C1orf115	0.005790932	-0.364253771
cg15301489	XKR6	0.000420813	-0.365119713
cg02132714	HOXB4	0.007567702	-0.366224205
cg16931707	PRICKLE1;PRICKLE1;PRICKLE1;PRICKLE1	0.000420813	-0.36688159
cg01529149	FAM83H	0.003185744	-0.367019894
cg17881660	SLC22A15;SLC22A15	0.000420813	-0.36755881
cg23428985	CLIP4	0.000636165	-0.367842457
cg20518096	AKR1D1	0.001569481	-0.367888048
cg21524538	SDCBP2	0.002321262	-0.368757847
cg05068452	UGT8	0.001569481	-0.369353466
cg07441944	CD82;CD82	0.000420813	-0.369479212
cg23045258	COL4A3;COL4A4;COL4A3;COL4A4;COL4A3;COL4	0.001569481	-0.369929646
cg02386311	RUNDC2A	0.000420813	-0.370453205
cg12976883	SP140;SP140	0.000420813	-0.371459576
cg26164712	CXCR5;CXCR5	0.000636165	-0.372435697
cg27486637	WDR17;WDR17;WDR17;WDR17	0.000420813	-0.372588656
cg02087075	SLC35B2;NFKBIE	0.000420813	-0.373170943
cg20849025	MPEG1	0.000420813	-0.373223392
cg03769371	LCA5;LCA5	0.007567702	-0.373416745
cg07069934	ISG20	0.003185744	-0.373835805
cg08352439	VOPP1	0.002321262	-0.374539784
cg02266771	PRSS16	0.004405484	-0.375969307
cg00380172	SASH1	0.003185744	-0.376060747
cg14409559	MSC	0.00961166	-0.376357959
cg15120525	LPP;LPP;LPP	0.002321262	-0.376442654
cg26146027	THRB;THRB;THRB	0.002321262	-0.376977568
cg17319142	C1QL2	0.003185744	-0.37909331
cg07018090	BANP;BANP	0.007567702	-0.379175171
cg22515937	FCRL1;FCRL1;FCRL1	0.000420813	-0.379279648
cg23514324	PPARG;PPARG;PPARG	0.001038267	-0.380414619
cg27487839	XKR6	0.001569481	-0.380774875
cg01035689		0.007567702	-0.381553852
cg26217402	C14orf43	0.000420813	-0.383584435
cg23234832	THRB;THRB;THRB	0.000420813	-0.385844347
cg26404422	ETS1;ETS1;ETS1	0.000420813	-0.386365979
cg04787728	NLRC3	0.000420813	-0.388197842

cg06963205	RIMS1;RIMS1;RIMS1;RIMS1;RIMS1;RIMS1	0.007567702	-0.38821076
cg20280350	MCOLN3	0.005790932	-0.388614346
cg08482682	RAB39	0.000420813	-0.389007916
cg19846040	EML6	0.000636165	-0.38927034
cg10605766	IQCB1;IQCB1	0.000420813	-0.390042366
cg03860038		0.005790932	-0.390421331
cg03147210	THEM4	0.00961166	-0.390451772
cg26828909	SAMD12;SAMD12;SAMD12;SAMD12	0.003185744	-0.391440184
cg02892755	STX12	0.000420813	-0.391643872
cg22697239	CD82;CD82	0.002321262	-0.392241529
cg00865356	FAM190A	0.005790932	-0.392348738
cg23683962		0.001569481	-0.392386646
cg04928005	THRB;THRB;THRB	0.007567702	-0.392880756
cg26703511		0.005790932	-0.393215507
cg00107970		0.000636165	-0.393473884
cg17694130		0.001038267	-0.393792778
cg11650874	CHID1;CHID1;CHID1;CHID1;CHID1	0.000420813	-0.394418086
cg25138484	ALPP	0.007567702	-0.395143106
cg23202468	ZBTB47	0.007567702	-0.39614216
cg16851385		0.000420813	-0.396377027
cg14622549	EP400	0.000420813	-0.39926399
cg19861842	RHOBTB2;RHOBTB2;RHOBTB2	0.000420813	-0.399731746
cg00362381	RAB40C	0.000420813	-0.399862391
cg00464927	LRP5	0.000420813	-0.400382954
cg01199327	CD82;CD82	0.007567702	-0.400845609
cg14733637	CLPTM1L	0.001836098	-0.401110775
cg08045301	ATXN1L;ATXN1L	0.001038267	-0.401549265
cg17588003	C17orf87	0.000420813	-0.401802761
cg16280667	CXCR5;CXCR5	0.000420813	-0.401884044
cg11821440	TBC1D12	0.007567702	-0.405271412
cg19672873	FAM177B	0.000420813	-0.40555274
cg21097090	TNFAIP8;TNFAIP8	0.000420813	-0.406863295
cg19602479	FCRL3	0.000420813	-0.408424421
cg16376000	FGF12;FGF12	0.005790932	-0.408505149
cg18345806	NMU	0.005790932	-0.409565503
cg14940871		0.000420813	-0.411097876
cg27602263	RAX	0.000420813	-0.411554936
cg00770471	C4orf34	0.000420813	-0.412670948
cg09734791	MSC	0.000636165	-0.413176052
cg21467365		0.000420813	-0.413306734
cg05094429	CCR6;CCR6	0.007567702	-0.414273871
cg24074594	FCRLA	0.000420813	-0.415057476
cg04576021	HLA-DOB	0.000420813	-0.415355728
cg18418928	MDS2	0.002321262	-0.415979757
cg24188017	SLC6A16	0.000420813	-0.416292334
cg01192487	SAMD12;SAMD12;SAMD12;SAMD12	0.002321262	-0.417182688
cg00303548	HS6ST3	0.007567702	-0.419293959
cg21794222	CCR6;CCR6	0.007567702	-0.422560193
cg10067737		0.000420813	-0.422574325
cg06996599	C6orf136;C6orf136;C6orf136	0.000420813	-0.422971884
cg25087423	CXCR5	0.000420813	-0.429204927
cg26399903		0.000636165	-0.429448636
cg04950342		0.000420813	-0.429507628
cg01218206	SIK3	0.000420813	-0.430023023
cg21585409	WIPI2;WIPI2;WIPI2;WIPI2;WIPI2	0.000420813	-0.432564441
cg26121053		0.00961166	-0.433858125
cg22160073	TRIM34;TRIM34;TRIM34;TRIM6-TRIM34;TRIM34	0.004405484	-0.435525065
cg02393640	LUZP6;MTPN	0.000420813	-0.435857534
cg24540521	THEM4	0.000420813	-0.435874659

cg15787744	NFKBIE	0.000420813	-0.437138367
cg04352288	CA5A	0.000420813	-0.438422528
cg16287740	TNPO3	0.000420813	-0.438826086
cg24110396	CCNY	0.000420813	-0.441173661
cg03356747	GRID1	0.001569481	-0.442085088
cg17587997	FYN	0.002321262	-0.443207824
cg13414750	CACNA1D;CACNA1D;CACNA1D	0.005790932	-0.44373015
cg17622855	ZDHHC14;ZDHHC14	0.000420813	-0.444228545
cg02231062	TNFRSF13C	0.000420813	-0.444387313
cg06125903	CTSZ	0.001038267	-0.444451491
cg14066207	RGS6	0.003185744	-0.444642656
cg26810157	TCF12;TCF12;TCF12;TCF12;TCF12	0.001038267	-0.446318134
cg16266809		0.000420813	-0.447884488
cg07493874	CLPTM1L	0.000420813	-0.450715184
cg04804377	RAD51AP2	0.007567702	-0.451142949
cg13325231	CLPTM1L	0.000420813	-0.4564231
cg13031210	DBX2	0.001038267	-0.457733311
cg18368845	FOXO1	0.000420813	-0.45809867
cg01396567	MCOLN3	0.001038267	-0.460256836
cg26675876	XKR6	0.005790932	-0.461724225
cg17976229	TRIM34;TRIM34;TRIM34;TRIM6-TRIM34;TRIM34	0.001038267	-0.462171663
cg00183239	DHTKD1	0.000420813	-0.463447511
cg00191102		0.000420813	-0.464403269
cg00539174	CTSZ	0.000420813	-0.46641547
cg00043095	SGPP2	0.000420813	-0.472698634
cg11193201	FCRLA	0.000420813	-0.474981732
cg08684639	WDR17;WDR17	0.001038267	-0.47980316
cg08786003	FCRL3	0.002321262	-0.484099097
cg22324981	NFATC1;NFATC1;NFATC1;NFATC1	0.001569481	-0.486922795
cg10699171		0.000420813	-0.487076078
cg06567596	LMTK2	0.000420813	-0.491455324
cg18563860	CTSZ	0.000420813	-0.493007817
cg22930549	RAD51L1;RAD51L1;RAD51L1	0.000420813	-0.494734723
cg06821460	ARF3	0.000420813	-0.496969166
cg17768768	CARD11	0.005790932	-0.497052516
cg03140624	LYST	0.000420813	-0.50708563
cg03969079	ARID5B	0.000420813	-0.507256166
cg17382048	CXCR5	0.000420813	-0.508117549
cg13584784	C6orf136;C6orf136;C6orf136	0.000420813	-0.509554382
cg07575896	ADAM19	0.000420813	-0.517795484
cg10141261	CHST15	0.005790932	-0.518009322
cg01453814	SLC6A16	0.000420813	-0.532219757
cg19590591	GOLGA3	0.001569481	-0.537253191
cg23217386	TRIM34;TRIM34;TRIM34;TRIM6-TRIM34;TRIM34	0.000420813	-0.542117389
cg26959945	MACC1	0.000420813	-0.556787147
cg19942083		0.000420813	-0.559861927
cg11199827		0.000420813	-0.560465307
cg02621376	MCOLN2	0.000420813	-0.573738197
cg26053840	GLO1	0.000420813	-0.583320634
cg23755933	GOLGA3	0.001038267	-0.585540614
cg00041047	SLC6A16	0.000466745	-0.595789196
cg19849557	C6orf136;C6orf136;C6orf136	0.000466745	-0.612356675
cg09390241		0.000420813	-0.614280316
cg24157392	BTLA;BTLA	0.002321262	-0.619782083
cg15602298	FCRL3	0.000532358	-0.626135479
cg13738327	LRP5	0.000420813	-0.658526517

Supplementary table 2:
20,661differentially methylated CpG sites comparing B-cell group ALL-1 and ALL-2.

TargetID	Gene name	FDR	δ β-value
cg18077971	PAX3	8.60E-06	0.709486709
cg23113963	SCNN1B	1.18E-05	0.706386408
cg00901264		8.60E-06	0.702210629
cg22848598	ADAM32	8.60E-06	0.696020857
cg03102879	SCNN1B	8.60E-06	0.693136932
cg16647921		8.60E-06	0.687866386
cg10440578	SYCP1	1.18E-05	0.685781917
cg27260772	TFAP2B	8.60E-06	0.680622978
cg24368848	ZSCAN1	8.60E-06	0.678535696
cg14507560	IRX4	8.60E-06	0.677878854
cg26001902	SLC5A7	8.60E-06	0.676110416
cg14416371	MIR129-2	8.60E-06	0.675962168
cg03356595		8.60E-06	0.67446449
cg25078444	FOXG1	8.60E-06	0.674085788
cg00665492	SLITRK3	1.96E-05	0.669702734
cg03024760	GABRG3	1.18E-05	0.668927708
cg26014538	ZIC1	3.34E-05	0.668665462
cg22797031		2.58E-05	0.666418019
cg02653559	ZIC4	3.21E-05	0.665869332
cg04569082		8.60E-06	0.665014937
cg01923218	CCDC67	8.60E-06	0.664481699
cg25566568		1.96E-05	0.66241709
cg18924324	PRDM13	7.23E-05	0.662169036
cg03705384	ALX1	1.11E-05	0.66196368
cg26796283	MSX2	1.11E-05	0.661882707
cg17526573	NRXN1	8.60E-06	0.661251347
cg10380328		1.96E-05	0.661124191
cg02468050	GRID2	8.60E-06	0.660428561
cg17302155	PRDM13	8.60E-06	0.658845932
cg21026830		8.60E-06	0.658384379
cg27376136		8.60E-06	0.658027817
cg19972558	PRSS12;PRSS12	8.60E-06	0.656831108
cg09111223	GRID2	1.50E-05	0.656221352
cg18286501	TMEM26	8.60E-06	0.655937224
cg17259183	NKX6-2	8.60E-06	0.655605811
cg18655441	MIPOL1	8.60E-06	0.654980607
cg26896155	FAM190A	1.96E-05	0.654238365
cg16150752	MARCH11	8.60E-06	0.653335554
cg13561592	PAPPA	8.60E-06	0.652183243
cg18235050		8.60E-06	0.651938495
cg25950625	PLCXD3	8.60E-06	0.650685659
cg06463958	T	8.60E-06	0.650458182
cg21152690	LIN7A;LIN7A	8.60E-06	0.649917886
cg13620034	MSC	8.60E-06	0.649469801
cg21289015	USH1C	8.60E-06	0.649243829
cg16038120	FOXG1	8.60E-06	0.649101898
cg06546806		8.60E-06	0.64833414
cg25900085	MIR124-2	8.60E-06	0.646670586
cg26422161	FOXQ1	8.60E-06	0.645750747
cg01431993	NEUROD1	1.60E-05	0.645235513
cg27336026	FOXQ1	8.60E-06	0.645048974
cg21219692		8.60E-06	0.64458906
cg20386316		8.60E-06	0.643787683
cg08684639	WDR17	1.96E-05	0.643211383
cg13031210	DBX2	8.60E-06	0.642780805

cg24980653		1.50E-05	0.641836388
cg01791874	MARCH11	8.60E-06	0.641553629
cg20365074	GULP1	1.11E-05	0.641452509
cg24843380	ZNF454	8.60E-06	0.640232109
cg25996119	NKX2-1	8.60E-06	0.640161734
cg27363829	SLITRK3	1.50E-05	0.639213104
cg08569799		8.60E-06	0.639143296
cg10454246		1.11E-05	0.638721813
cg08070771	ZIC4;ZIC1	2.08E-05	0.638521905
cg14425863	USH1C;USH1C	8.60E-06	0.63841919
cg07790615	RYR2	1.50E-05	0.638357431
cg12296772	MTMR7	8.60E-06	0.638208422
cg00043095	SGPP2	1.50E-05	0.637947388
cg19809499	FOXE3	8.60E-06	0.637512367
cg20647610	WDR69	8.60E-06	0.63682723
cg02864844		1.18E-05	0.635552697
cg06500337		8.60E-06	0.635390651
cg01414185	HOXD4	1.50E-05	0.633484722
cg05864326	HOXD3	8.60E-06	0.633197659
cg08231348	HOOK1	8.60E-06	0.633005987
cg12079322	GALNT13;GALNT13	8.60E-06	0.632526736
cg08189989		1.18E-05	0.632498901
cg23200020	LOC643719	1.11E-05	0.632321222
cg26452868		0.000110797	0.632180073
cg13539545		1.11E-05	0.632086572
cg11433319	CHODL	1.11E-05	0.631972238
cg17712694	MARCH11	8.60E-06	0.631908265
cg13692446		1.11E-05	0.631649114
cg22001496		1.50E-05	0.631379303
cg15690342		8.60E-06	0.631061192
cg16528511	RIPPLY2	1.11E-05	0.630738712
cg05835105	PITX2;PITX2;PITX2	2.58E-05	0.628563361
cg19619405	ADAMTS20	1.11E-05	0.627461612
cg26235215	PTPRZ1;PTPRZ1	8.60E-06	0.626969288
cg11033835	ROBO2	5.59E-05	0.626484318
cg24659054	NKX6-2	8.60E-06	0.626081983
cg23347718	PCDHAC2	8.60E-06	0.626048679
cg09918510	HYDIN;HYDIN	2.08E-05	0.626011575
cg21479226		2.08E-05	0.625889322
cg00469207		1.50E-05	0.625784099
cg14000361	GCM2	1.11E-05	0.625034993
cg19849428	LRRC4C;LRRC4C	2.58E-05	0.624934852
cg24892966		1.11E-05	0.62485546
cg12799689	DOK6	8.60E-06	0.624629883
cg25114913	FUT9;FUT9	8.60E-06	0.624222351
cg24452128		8.60E-06	0.624098146
cg24169669		1.11E-05	0.624043635
cg26533745	NPNT;NPNT	2.58E-05	0.623861218
cg04021856	LYPD6	8.60E-06	0.623743202
cg07126525		0.000117986	0.623076077
cg16987305	LEPR;LEPR;LEPR	1.50E-05	0.622449748
cg16670809	ADAMTS16	1.50E-05	0.622420343
cg02664812		8.60E-06	0.621993206
cg09858188		8.60E-06	0.621970681
cg00793935	TRPM8	8.60E-06	0.621902829
cg07882671	IRX4	8.60E-06	0.621715837
cg07195011	CTNND2	2.58E-05	0.621552863
cg23302649	C14orf23;C14orf23	1.50E-05	0.621412013
cg00853103	A2BP1;A2BP1	8.60E-06	0.621241923
cg16642284	FOXI2	1.11E-05	0.620935037

cg12646385		1.96E-05	0.62085948
cg19859290		8.60E-06	0.620563012
cg17030173	MARCH11	8.60E-06	0.62055452
cg25316663		1.11E-05	0.620486065
cg11549265	LOC100270746;C6orf41	5.59E-05	0.620302228
cg21426003	GBX2	1.50E-05	0.620252718
cg23189410	ZIC4;ZIC1	1.11E-05	0.620061095
cg05942459	GRIK2;GRIK2;GRIK2	8.60E-06	0.619903066
cg20809378		8.60E-06	0.619680946
cg02519751	ZIC1	3.34E-05	0.619538336
cg18856388		1.11E-05	0.619534303
cg19047660		8.60E-06	0.619520542
cg27555582		2.58E-05	0.61935422
cg07897248	DCC	8.60E-06	0.618964676
cg18093372		8.60E-06	0.618644675
cg06711831	GREB1L	1.96E-05	0.617932462
cg10168149	FLJ32063	1.50E-05	0.617892391
cg08753373	DPYSL4	8.60E-06	0.617865662
cg09513990		8.60E-06	0.61752493
cg14858267		8.60E-06	0.617504767
cg02527669	OBSL1	1.11E-05	0.617496008
cg17498101	GJA1	1.11E-05	0.617237873
cg14487131	FOXB2	1.50E-05	0.616718801
cg12832313	PCDHAC2;PCDHAC2;PCDHA7;PCDHA12;PCD	1.11E-05	0.616235424
cg09088988	STK32A;STK32A	1.11E-05	0.615690943
cg18349138	CHODL	1.18E-05	0.615686119
cg09008705	NGF	2.58E-05	0.615443057
cg19108881	CASR	1.50E-05	0.615364219
cg00228475		8.60E-06	0.615291634
cg16288089	TAC1;TAC1;TAC1;TAC1;TAC1;TAC1;TAC1;TAC	8.60E-06	0.615119826
cg16871519	TFAP2A;TFAP2A	2.58E-05	0.615003537
cg15222899		8.60E-06	0.614957759
cg12164612	KCNA4	8.60E-06	0.614902205
cg08109850		8.60E-06	0.614762454
cg10893095		1.50E-05	0.61442018
cg22498143	SPTBN4	1.50E-05	0.614140548
cg17861455	TRPV3	1.50E-05	0.613701777
cg12845520	C8orf47;C8orf47	1.96E-05	0.613477579
cg13155001	PRDM5;PRDM5	2.58E-05	0.612479636
cg12112529		8.60E-06	0.612271128
cg15304699	NKX6-2	8.60E-06	0.61206236
cg17823751		8.60E-06	0.611343358
cg26122980	AMPH;AMPH;AMPH;AMPH	3.34E-05	0.611282898
cg17319142	C1QL2	1.11E-05	0.611105225
cg06516947	ARHGAP8;PRR5-ARHGAP8;ARHGAP8	1.11E-05	0.610843457
cg01396567	MCOLN3	2.58E-05	0.610583355
cg18074954		8.60E-06	0.610556448
cg09012633	LOC100130015;LOC100130015	3.34E-05	0.610263237
cg09795588	PTF1A	8.60E-06	0.609846092
cg13246235	PHACTR1	1.50E-05	0.609815955
cg01192487	SAMD12;SAMD12;SAMD12;SAMD12	2.58E-05	0.609586083
cg21599230	TRAM1L1;TRAM1L1	1.11E-05	0.609101608
cg25026529	BARHL2	8.60E-06	0.608894467
cg24946597	NRG1;NRG1	8.60E-06	0.608482119
cg13330559	SHISA6	0.000233422	0.60835289
cg21306240	EPHA10;EPHA10	8.60E-06	0.608199462
cg18823007		1.11E-05	0.608196119
cg21901718	MARCH11	8.60E-06	0.608182041
cg02326806		8.60E-06	0.60789722
cg10147797	SDR42E1;SDR42E1	1.50E-05	0.607810084

cg25537993	ZSCAN1	8.60E-06	0.607661936
cg04842146	RALYL;RALYL;RALYL;RALYL	1.96E-05	0.607302712
cg14441976	SLC27A6;SLC27A6	8.60E-06	0.606520786
cg15774465	KCNB2	3.34E-05	0.606282006
cg23930856	TFAP2B	1.18E-05	0.606227159
cg22212691	OBSL1	8.60E-06	0.606077868
cg01389445	LRAT	8.60E-06	0.605729436
cg20229534	GCM2	8.60E-06	0.605567373
cg13481969		3.21E-05	0.605553897
cg04347874	NKX2-1;NKX2-1	8.60E-06	0.605533157
cg00989853	IRF6;IRF6	1.50E-05	0.605400387
cg08729318	TMTC1	8.60E-06	0.604728467
cg19155518	GRIK2;GRIK2;GRIK2	8.60E-06	0.604546536
cg01518607		8.60E-06	0.604251857
cg25032595	CLDN10;CLDN10;CLDN10;CLDN10	1.50E-05	0.604185003
cg26115633	FOXI2	8.60E-06	0.604146764
cg18388380	RGS7BP	8.60E-06	0.603959582
cg04100532	UNC80;UNC80	1.50E-05	0.603015584
cg16729415	GJD2	8.60E-06	0.602848416
cg19839798	FAM155A;FAM155A	1.50E-05	0.602786081
cg02725370	PITX2;PITX2	8.60E-06	0.602327507
cg03502002	GALR1;GALR1	1.11E-05	0.602207212
cg18118685	GALNT13	1.11E-05	0.601993269
cg22868282		1.11E-05	0.601922785
cg01105494	SPAG17	1.50E-05	0.601774053
cg12272837	PPAP2C;PPAP2C;PPAP2C	8.60E-06	0.601705932
cg11525643	GJD2	1.11E-05	0.601630419
cg25145765	GABRA2;GABRA2;GABRA2	3.21E-05	0.601507709
cg10156125	UGT8;UGT8	4.34E-05	0.601209049
cg24150623	MIR125B1;LOC399959	4.34E-05	0.60091972
cg26902216	MAL2	1.96E-05	0.600587875
cg06781135	LOC100270746;C6orf41	0.000148457	0.600182462
cg05494604		8.60E-06	0.599996571
cg17632579	PRSS12	8.60E-06	0.59922418
cg26871372		8.60E-06	0.599020003
cg05647859	LIN7A	1.50E-05	0.598977871
cg16086620	CHGA;CHGA	8.60E-06	0.598822426
cg20984053	MNX1;MNX1	8.60E-06	0.598771763
cg07150062	ASPG	4.34E-05	0.598551873
cg20872937	GALR1	1.11E-05	0.598512548
cg25485875	NKX6-2	1.11E-05	0.598464802
cg24938830	UNC80;UNC80	8.60E-06	0.598374958
cg17211349	SLITRK3	8.60E-06	0.598365049
cg08445802		1.96E-05	0.598295946
cg07204280	CIDEA;CIDEA	3.21E-05	0.598146157
cg08564661	PLEKHA1	7.23E-05	0.598122138
cg23709528		8.60E-06	0.598059821
cg09799983	CYP1B1	3.34E-05	0.597666024
cg03352106	KCNJ8	8.60E-06	0.597316667
cg13879483	USP44;USP44	2.58E-05	0.597183269
cg00058329	P2RX2;P2RX2;P2RX2;P2RX2;P2RX2;P2RX2	8.60E-06	0.597078387
cg03686593	FAM38B	3.34E-05	0.596844595
cg00795341	THBS4	1.96E-05	0.596702748
cg00303548	HS6ST3	8.60E-06	0.596494893
cg10530851	NKX2-8	1.50E-05	0.596095242
cg21195256	C14orf23;C14orf23	3.34E-05	0.595719297
cg24862510		2.08E-05	0.5956401
cg25218716	FOXA2;FOXA2	2.58E-05	0.595630873
cg26636193		8.60E-06	0.595375493
cg01261798	DPYSL4	1.11E-05	0.595319731

cg07449450	USH1C;USH1C	1.18E-05	0.595015396
cg23034799	CADM1;CADM1	8.60E-06	0.594965478
cg21039708	OTX2OS1	5.59E-05	0.594957004
cg12778215	DKK3;DKK3;DKK3;DKK3	0.000117986	0.594955552
cg13445796	DBX1	1.11E-05	0.594949496
cg18560328	SYT9	2.58E-05	0.594945384
cg02764245		2.58E-05	0.594936636
cg10961753	HOXC13	8.60E-06	0.594606209
cg10298557	RORA	1.96E-05	0.594533562
cg07149609		1.50E-05	0.59449661
cg26307359	SEZ6;SEZ6	8.60E-06	0.594343992
cg12530864		2.58E-05	0.594195771
cg27032232	DPP6;DPP6	1.11E-05	0.594095718
cg22538054	USP44;USP44	1.11E-05	0.593788409
cg01735384		1.11E-05	0.593771988
cg00063174	WDR69	8.60E-06	0.593696974
cg13919148	INADL;INADL	3.34E-05	0.593431498
cg05728754		1.80E-05	0.59320795
cg08234308	LEPR;LEPR;LEPR	8.60E-06	0.593042497
cg14644001	PRRT1	1.96E-05	0.592991901
cg15556502	MIR129-2	1.11E-05	0.592805093
cg14564616		1.11E-05	0.592504573
cg08597761	HYDIN;HYDIN;HYDIN;HYDIN	8.60E-06	0.592452079
cg24480695	IGSF22	1.11E-05	0.592431092
cg00034076	RALYL;RALYL;RALYL;RALYL;RALYL	8.60E-06	0.592413498
cg23291534	KCNV1	8.60E-06	0.592346869
cg23992410	PROX1	1.96E-05	0.592291346
cg04804377	RAD51AP2	1.96E-05	0.592257262
cg12912737	REM1	1.50E-05	0.592145517
cg06619066	CHST9;CHST9	8.60E-06	0.59207177
cg08183141	MYH14;MYH14;MYH14	8.60E-06	0.591381858
cg14523847	DPP6;DPP6	8.60E-06	0.591232482
cg13096007	SOD3	1.96E-05	0.591107999
cg04747226	GRIA4;GRIA4;GRIA4;GRIA4	0.000205664	0.591028766
cg13916459	ALX1	8.60E-06	0.590856145
cg23342718		8.60E-06	0.59084896
cg03580297	LPPR4;LPPR4	1.11E-05	0.590759751
cg01995743	SLC35F1	8.60E-06	0.590718583
cg00683332		8.60E-06	0.590615674
cg05362548	TDH	1.96E-05	0.59049024
cg02758612		8.60E-06	0.590444921
cg14356608		8.60E-06	0.590382516
cg00697992	NXPH2	8.60E-06	0.589938044
cg02466815	HOXD1	8.60E-06	0.589877668
cg18542829	ABCB1;RUNDC3B;RUNDC3B;RUNDC3B	4.34E-05	0.589868251
cg03738025		1.50E-05	0.589774147
cg13696942	DBX1	8.60E-06	0.589396699
cg27323784		8.60E-06	0.589330254
cg10192893	PHOX2B	1.11E-05	0.589220569
cg17484671		8.60E-06	0.589189444
cg09979478		1.50E-05	0.589053796
cg17280346	ZIC1	1.50E-05	0.588961216
cg26134895	FBXL7	1.11E-05	0.588791838
cg08782022	MGRN1;MGRN1;MGRN1;MGRN1	1.96E-05	0.588540023
cg02023345	TMEM26	5.59E-05	0.588496621
cg05452524	HLF	0.000122184	0.588473996
cg05452406	GCM2	1.96E-05	0.588440357
cg12518442	BMP7	0.000233422	0.588288298
cg08073312	SOX14	1.96E-05	0.588202268
cg25566352	RFX4	1.11E-05	0.588126904

cg11174855	NKX6-2	2.80E-05	0.587975615
cg19597382	POU4F2	8.60E-06	0.587896024
cg12072964	LAMA1	8.60E-06	0.587442695
cg01959730		1.96E-05	0.586481162
cg27199384	A2BP1;A2BP1;A2BP1	8.60E-06	0.58608194
cg12473406	GREB1L	9.24E-05	0.585812921
cg27122213	OTX2	2.58E-05	0.585761453
cg24744430	RAB32	7.23E-05	0.585657849
cg10658542	SYT10;SYT10	1.96E-05	0.585166681
cg19052355	GBX2	5.59E-05	0.584993328
cg14118515	EVX2	1.96E-05	0.584703773
cg09626894	CFTR	8.60E-06	0.584699238
cg09064304		4.34E-05	0.584611287
cg04555373	NRG1	8.60E-06	0.584553953
cg17768491	SPTBN4	8.60E-06	0.584294435
cg07129523	PCDHB4	8.60E-06	0.584189075
cg09942248	NEUROD1	1.11E-05	0.583931334
cg07014673		8.60E-06	0.583892843
cg01697732	FAM20A	1.96E-05	0.583510846
cg01184975		1.11E-05	0.583462782
cg21995304		1.96E-05	0.583435806
cg01227537	ZIC1	3.34E-05	0.583355824
cg04475027	TMEM132C	2.58E-05	0.583310301
cg14991984	LPPR5;LPPR5	2.58E-05	0.583262065
cg25834419	GOLSYN;GOLSYN;GOLSYN;GOLSYN;GOLSYN	1.50E-05	0.58309747
cg14520424		1.11E-05	0.583096069
cg05901765	MARVELD2;MARVELD2	1.11E-05	0.582957717
cg23160016	GABRA2;GABRA2;GABRA2	2.58E-05	0.582954148
cg06926934	NR2F2;NR2F2;NR2F2;MIR1469;NR2F2	1.18E-05	0.582500188
cg07962143		8.60E-06	0.582134794
cg03098837	DPY19L2P4	1.60E-05	0.582123
cg08464190	CBLN1	1.11E-05	0.581842829
cg08002883	FGF12;FGF12	8.60E-06	0.581803563
cg08146483	LHX8	8.60E-06	0.581790708
cg04249522		3.34E-05	0.581750738
cg10355837	CACNG4	2.58E-05	0.581693918
cg23065934	MARCH11	1.96E-05	0.58166775
cg19456540	SIX6	8.60E-06	0.581579239
cg12836011	TLL1	8.60E-06	0.581550232
cg27513979	HS6ST3	8.60E-06	0.58148608
cg00582971	GRM6	3.34E-05	0.581331511
cg00757731	MAGI1;MAGI1;MAGI1	1.11E-05	0.581161383
cg03007522	GATA4	1.50E-05	0.581058034
cg27505273	SOX14	1.96E-05	0.581022383
cg14603098	ACCN1	3.21E-05	0.580904741
cg14084091		8.60E-06	0.580844829
cg03823904	SEMA3C;SEMA3C	5.59E-05	0.580816991
cg03970036	PTPRN	1.11E-05	0.580813944
cg11821440	TBC1D12	1.11E-05	0.58078253
cg22795586		8.60E-06	0.580759149
cg04632671	PPARG;PPARG;PPARG	1.50E-05	0.580645593
cg13096208	ST8SIA3;ST8SIA3	8.60E-06	0.580407181
cg27393010		8.60E-06	0.580262516
cg25051529	SLC25A21;SLC25A21;LOC100129794	8.60E-06	0.5802523
cg14445814	DBC1	8.60E-06	0.580226952
cg11499970	FBLL1	8.60E-06	0.580190486
cg09919570		8.60E-06	0.58015708
cg11361636		8.60E-06	0.580136154
cg14745270	GOLSYN;GOLSYN;GOLSYN;GOLSYN	1.50E-05	0.580056981
cg06418871	MARVELD2;MARVELD2	8.60E-06	0.579995209

cg00515457		8.60E-06	0.57989448
cg23727983	DDX25;PUS3	8.60E-06	0.579873018
cg15133351	EVX2	2.39E-05	0.57981194
cg04865180		1.96E-05	0.579809102
cg04996219	CTNND2	2.58E-05	0.57973596
cg10568066	RNF39;RNF39	0.000117986	0.579600665
cg17677490		1.11E-05	0.579508605
cg06083330	POU4F2	3.34E-05	0.579447887
cg11649981		8.60E-06	0.579407998
cg10566121	MPPED2	0.000117986	0.57920377
cg13208832	FAM149A	0.000290372	0.579199062
cg10034890	DNER	3.34E-05	0.57914362
cg19776515	SDR16C5	8.60E-06	0.579126552
cg04111071	TMEM196	8.60E-06	0.578767924
cg11723850		8.60E-06	0.578672939
cg23997263	FUT9	8.60E-06	0.578544685
cg02064106	C6orf118	8.60E-06	0.578484368
cg03117976	FAM38B	5.59E-05	0.578413119
cg27275352	CILP2	8.60E-06	0.578210195
cg25547580	ONECUT2	4.34E-05	0.577957478
cg00396667	PITX1	8.60E-06	0.577804356
cg26947626		1.11E-05	0.577716568
cg03553786	LOC285375	2.58E-05	0.577714569
cg11792516	HOOK1	8.60E-06	0.577712295
cg25088758	COL25A1;COL25A1;COL25A1;COL25A1	8.60E-06	0.577492992
cg16887264	POU4F2	3.21E-05	0.577479869
cg00309582	BMP7	0.000187194	0.577331458
cg10511988	VAX1	8.60E-06	0.576586512
cg14094960	EGFR;EGFR;EGFR;EGFR;EGFR;EGFR;EGFR;	1.50E-05	0.57654239
cg16240368	SPAG17	8.60E-06	0.576195317
cg03646889	LPPR4;LPPR4	8.60E-06	0.576092235
cg11929643	CELSR3	7.23E-05	0.57591098
cg04360793	ELTD1;ELTD1	1.96E-05	0.575806675
cg17840719	ZNF454;ZNF454	3.34E-05	0.575781351
cg16392213	FEZF1;FEZF1	8.60E-06	0.57545655
cg23944251	LOC100128811;GPR158	1.11E-05	0.575163835
cg02147443		1.11E-05	0.574880379
cg12892506	ZIC4;ZIC4;ZIC4	7.23E-05	0.574829021
cg01739725		8.60E-06	0.574748194
cg26860935		8.60E-06	0.57432902
cg22731271	MEIS1	1.60E-05	0.574304946
cg19029181	ZIC1	1.11E-05	0.574301406
cg12248614	SPTBN4	1.11E-05	0.574296787
cg26650638		1.96E-05	0.574213738
cg02217786		8.60E-06	0.573937463
cg19697530	CADM1;CADM1	1.96E-05	0.573553796
cg06782035	MARCH11	1.50E-05	0.573359788
cg07411620	EVX2	1.11E-05	0.573313207
cg13390867		8.60E-06	0.573287018
cg24393316	FOXE1	8.60E-06	0.573080182
cg05983315	ZSCAN1	8.60E-06	0.57304461
cg12516059	AVPR1A;AVPR1A	8.60E-06	0.57293603
cg13786089	ZFR2;ZFR2;ZFR2	8.60E-06	0.572651327
cg05375728	DAB1	8.60E-06	0.5726451
cg06785999	SIX6;SIX6	8.60E-06	0.572570157
cg13251269	GPR85;GPR85;GPR85;GPR85;GPR85	8.60E-06	0.572442993
cg26460471	GRP;GRP;GRP	5.59E-05	0.572405578
cg16847696	GPR37	8.60E-06	0.572277808
cg22635491	TBX3;TBX3	8.60E-06	0.572258349
cg26721264	GALR1	8.60E-06	0.572088196

cg13072241	GPR126;GPR126;GPR126;GPR126	1.50E-05	0.57191514
cg11130317		8.60E-06	0.571681312
cg27655158		1.18E-05	0.571439475
cg16727201	CIDEA;CIDEA	1.96E-05	0.57131973
cg26828909	SAMD12;SAMD12;SAMD12;SAMD12	3.34E-05	0.571174964
cg26993729	LRAT	8.60E-06	0.571167219
cg06335867	NXPH1	1.50E-05	0.571115238
cg06598836		1.11E-05	0.570934181
cg26678605		8.60E-06	0.570930347
cg15471815	HIST3H2BB;HIST3H2A	8.60E-06	0.570663919
cg09438147	UNC80;UNC80	8.60E-06	0.570646451
cg09602288		8.60E-06	0.570567164
cg17949440	LRRC4C;LRRC4C	2.58E-05	0.570489877
cg25092681	MARCH11	1.50E-05	0.570370777
cg05387519	AGBL4	8.60E-06	0.570369663
cg10512875	TMTC1	8.60E-06	0.570185592
cg05781968	WNT5A	5.59E-05	0.570136916
cg04737916	P2RX2;P2RX2;P2RX2;P2RX2;P2RX2;P2RX2	8.60E-06	0.570047156
cg05327835	IDUA	4.34E-05	0.569962491
cg09930033	RFX6	8.60E-06	0.569962489
cg05904135	NKX6-2	8.60E-06	0.569832867
cg24710320	MGC2889;HRASLS	3.34E-05	0.569787535
cg15718581	ZFP42	2.80E-05	0.569632436
cg12848065	LOC200726	8.60E-06	0.569602531
cg22455914	LAMA1	3.34E-05	0.569501821
cg02910208	VSTM2A;VSTM2A	8.60E-06	0.569148893
cg05876564	EFNB2	3.92E-05	0.568989761
cg15820062		4.34E-05	0.568872461
cg13080565	GRIK2;GRIK2;GRIK2	1.96E-05	0.568788495
cg02681442	FOXG1	4.34E-05	0.568784337
cg18322569	BARHL2;BARHL2	5.59E-05	0.568769493
cg04584301	PCSK2	8.60E-06	0.568554701
cg16485558	RNF180;RNF180	1.50E-05	0.56851155
cg13495205	AJAP1;AJAP1	2.58E-05	0.568395399
cg02991338	FOXG1	1.96E-05	0.568361548
cg07792478	MIR124-2	1.50E-05	0.568327341
cg01851399	PKP2;PKP2	1.50E-05	0.567974482
cg03111404		1.50E-05	0.567739298
cg17470327	ROBO2	8.60E-06	0.567685629
cg15146859	GALR1	1.11E-05	0.56761376
cg26059153	MACROD2;MACROD2	0.000187194	0.567600832
cg21199922	FLJ13197	1.50E-05	0.567564665
cg01422476	C16orf73;C16orf73	8.60E-06	0.567551301
cg24436446	UGGT2;UGGT2	8.60E-06	0.567510995
cg10741025	PAX9	1.96E-05	0.567435591
cg25150243	SLC27A2;SLC27A2	1.50E-05	0.567330583
cg26621790		8.60E-06	0.567257684
cg08827307	ADAM32	8.60E-06	0.567239708
cg25741487		2.58E-05	0.56710571
cg20888142	SYN2;SYN2	1.50E-05	0.567086486
cg09424526	PAX3;PAX3;PAX3;CCDC140;PAX3;PAX3;PAX3	1.96E-05	0.567022289
cg08607018		3.34E-05	0.567006663
cg21705897	HMGCLL1;HMGCLL1	2.58E-05	0.56698938
cg06629130		1.96E-05	0.566891748
cg14511946		1.96E-05	0.566850497
cg20551181	SLC6A5	3.21E-05	0.566791572
cg11856810	KCNJ3	1.11E-05	0.566673935
cg12836959		8.60E-06	0.566622737
cg13347257	LHFPL4	8.60E-06	0.566596406
cg18952796	NPTX2;NPTX2	1.50E-05	0.566514027

cg24638326	C1orf92	1.50E-05	0.566444306
cg04515996	TBX18	1.50E-05	0.566191873
cg07068327	GPR123	4.34E-05	0.565878333
cg19019537	C8orf42	9.24E-05	0.5657848
cg27236875	ERBB4;ERBB4	8.60E-06	0.565760234
cg04022063	TJP1;TJP1;TJP1;TJP1	1.11E-05	0.565505617
cg08190858		1.50E-05	0.565495847
cg01426853	TMEM229A	4.34E-05	0.565404924
cg18147485	KCNV1;KCNV1	8.60E-06	0.565397297
cg01116059	SNCAIP	2.58E-05	0.565334244
cg17025835	TMEM229A;TMEM229A	3.34E-05	0.565201765
cg09639725	GPR123	0.000117986	0.56515611
cg11775837	STXBP6	5.11E-05	0.565136522
cg15415452		3.34E-05	0.565013836
cg26320830	MARCH1;MARCH1	8.60E-06	0.56497823
cg12400336	TJP1;TJP1	3.34E-05	0.564936869
cg04534765	GALR1;GALR1	8.60E-06	0.564929826
cg19099050	LHFPL4	8.60E-06	0.564886772
cg25702335	CLDN10;CLDN10;CLDN10	8.60E-06	0.564862269
cg04248446		8.60E-06	0.564638162
cg26466587	MCHR2;MCHR2	9.24E-05	0.564628795
cg02090033	MAGI1;MAGI1;MAGI1	8.60E-06	0.564625567
cg11930554		0.000117986	0.564609256
cg11203512	CCDC67	2.58E-05	0.564562874
cg21517947	SFRP1	8.60E-06	0.564555462
cg10188823	NEFH;NEFH	8.60E-06	0.564419644
cg08699935		7.23E-05	0.564388781
cg20122943	CACNG8	1.11E-05	0.564302318
cg15124757	RAD51AP2	4.34E-05	0.564131883
cg25368122	LHX8	1.11E-05	0.564108736
cg13023623	OTX2	8.60E-06	0.563969096
cg22302985	FAM155A	8.60E-06	0.563919494
cg27464184		8.60E-06	0.563880891
cg06515144	NPHS2	8.60E-06	0.563858093
cg04742780	GATA5	2.39E-05	0.56377222
cg18206017	RIPPLY2	8.60E-06	0.563687757
cg15133018		2.08E-05	0.563615833
cg11163975	TRAM1L1	1.50E-05	0.563590289
cg24378692	PLOD2;PLOD2;PLOD2;PLOD2	8.60E-06	0.563542712
cg27264249	CNTN4	8.60E-06	0.563506227
cg03364108	C22orf45;C22orf45;UPB1	8.60E-06	0.56346851
cg26547924	KCNK2;KCNK2;KCNK2	1.60E-05	0.563322569
cg27189087	RAX	4.34E-05	0.563260394
cg09082903	NPY1R	3.34E-05	0.563252673
cg23546474	PAX3;PAX3;PAX3;CCDC140;CCDC140;PAX3;P	8.60E-06	0.563007356
cg21331821	ZSCAN1	8.60E-06	0.562980041
cg04251368	KCNQ1DN	8.60E-06	0.562966415
cg16462462	C8orf85	1.96E-05	0.56294261
cg02656891	C1orf94;C1orf94	8.60E-06	0.562875637
cg22841810	EBF2	1.96E-05	0.562846209
cg16232126	SLC5A7;SLC5A7	8.60E-06	0.562794764
cg15031661	FMN2	1.11E-05	0.562644271
cg25539045	FOXI2	1.50E-05	0.562617728
cg19898128	TLL1;TLL1	1.60E-05	0.562546337
cg01445580	C1orf94;CSMD2	4.34E-05	0.562362976
cg12200038		1.11E-05	0.562235426
cg03177042		1.50E-05	0.561931172
cg19761848	GBX2	1.50E-05	0.561910232
cg18794839	KCNK2;KCNK2;KCNK2	8.60E-06	0.561861783
cg16967640	LMX1A	8.60E-06	0.561859933

cg22096450	ZNF215;ZNF215	3.34E-05	0.561727391
cg18249580		1.11E-05	0.561546091
cg05290695	SAMD12;SAMD12	1.60E-05	0.561537194
cg22572159	NGF	1.96E-05	0.561359147
cg10941747	BMP7	0.000233422	0.56132911
cg16937268		1.50E-05	0.561326909
cg05013064	TFAP2C	9.24E-05	0.561274093
cg08566455		1.11E-05	0.561120088
cg14781189	KCNH7;KCNH7;KCNH7;KCNH7	1.96E-05	0.561090717
cg12378187	FEZF2	0.000290372	0.561069443
cg25124276	LOC100128811;GPR158	1.11E-05	0.561059667
cg09273054	MAL2	0.000290372	0.560910951
cg00767642	NMNAT2;NMNAT2	9.24E-05	0.56085188
cg24714106	LOC145845	1.96E-05	0.56084786
cg19391276	PRDM13	8.60E-06	0.560841014
cg15825786	GPR123	5.59E-05	0.560804634
cg06304097	TCERG1L	8.60E-06	0.560801031
cg27659049		1.50E-05	0.560781982
cg22491927	CACNA1A;CACNA1A;CACNA1A;CACNA1A	1.50E-05	0.560548563
cg08535600	OXTR;OXTR	1.11E-05	0.560493607
cg05649108	BRUNOL4;BRUNOL4;BRUNOL4;BRUNOL4	8.60E-06	0.560425423
cg07676859	SSTR4	1.50E-05	0.560242503
cg03572772	PCDHB15	8.60E-06	0.56023637
cg08227526		1.50E-05	0.560236185
cg26559315	LYNX1;LYNX1;LYNX1;LYNX1;LYNX1	3.34E-05	0.560234386
cg10028929		8.60E-06	0.560155395
cg12097222	EFHA2	2.58E-05	0.559977529
cg20265661	LYPD1;LYPD1	1.96E-05	0.559725062
cg23906738	NKX2-1;NKX2-1	3.21E-05	0.559686368
cg15489250		8.60E-06	0.559639521
cg05860723	MYF6	8.60E-06	0.559603187
cg02051771	PCDHA7;PCDHA12;PCDHA6;PCDHA10;PCDH/	1.11E-05	0.559595849
cg07166654		1.96E-05	0.559528314
cg07780095	ALX1	8.60E-06	0.559509346
cg10277927	TRAM1L1	5.59E-05	0.559466969
cg24727311	C8orf48	8.60E-06	0.559378787
cg17405234	FAM190A	1.11E-05	0.559363938
cg06346974	DNAJC6	1.11E-05	0.559357138
cg21145136	SH3GL3;SH3GL3;SH3GL3	1.96E-05	0.55928753
cg14743462	HIST3H2A	1.50E-05	0.559210439
cg00575005	RIPPLY2	1.11E-05	0.559140204
cg11413039	PUS3;DDX25	2.58E-05	0.559136179
cg02788400	DZIP1;DZIP1	8.60E-06	0.559132646
cg20014960	PAMR1;PAMR1	1.96E-05	0.559065397
cg20388294	RND3	1.50E-05	0.559039181
cg11777523	GPR148	7.23E-05	0.558969902
cg26558485	CYP4X1;CYP4X1	1.50E-05	0.558935492
cg24675150		4.34E-05	0.558694552
cg14699728	NPAS4;NPAS4	8.60E-06	0.558678251
cg11254700		2.58E-05	0.558537107
cg13682019	MATN2;MATN2	2.58E-05	0.558313192
cg09876651	MIR618;LIN7A	8.60E-06	0.558236481
cg12250896	DPYSL4	1.11E-05	0.558169932
cg05064645	EGFR;EGFR;EGFR;EGFR;EGFR;EGFR;EGFR;	3.34E-05	0.558157615
cg00699993	GRIA2;GRIA2;GRIA2	1.11E-05	0.558145201
cg26817217	TOX3;TOX3	3.34E-05	0.558131502
cg26866482	ASCL1	5.59E-05	0.558062666
cg22280038	MAGI2	1.11E-05	0.558023304
cg02874376	DLK1;DLK1	1.11E-05	0.557982813
cg03692651		1.96E-05	0.557849117

cg23649435	STOX2	2.58E-05	0.557719322
cg23999695		1.96E-05	0.557570034
cg05167251	HOXD9;HOXD9	8.60E-06	0.557500988
cg25984671	TCF15	1.50E-05	0.557496444
cg19206146		8.60E-06	0.557489934
cg02770054	ATP4A	8.60E-06	0.557358139
cg15123692	KLHL29	8.60E-06	0.557321212
cg15457058	FOXE3	5.59E-05	0.557310657
cg00663077	ZFP42	1.50E-05	0.557272241
cg15637465	SLIT3	1.50E-05	0.557007898
cg21189438	C9orf122	1.96E-05	0.556854537
cg18863595	GRM7;GRM7;GRM7;GRM7	1.50E-05	0.556799151
cg00001747		1.50E-05	0.556660739
cg26675876	XKR6	5.59E-05	0.556517526
cg17774559	IRX4	8.60E-06	0.556508117
cg15030849	COX6B2;COX6B2	1.96E-05	0.556481414
cg23026864	PCDHA7;PCDHA12;PCDHA6;PCDHAC1;PCDH	1.11E-05	0.556322956
cg22403811	RALYL;RALYL;RALYL;RALYL;RALYL	8.60E-06	0.556309964
cg08182446	GABRG3	1.96E-05	0.556268256
cg19885761	CPLX2;CPLX2	8.60E-06	0.5562392
cg17928584	STK32A;STK32A	1.50E-05	0.556176032
cg06769546	NKX6-2	8.60E-06	0.556164796
cg18363918	IGLON5	8.60E-06	0.556089251
cg06783737		1.50E-05	0.555971988
cg14029968	TBC1D12	5.59E-05	0.555954635
cg11857704		8.60E-06	0.555816847
cg20438306	PHOX2B	1.50E-05	0.555791476
cg25191850	KCNK1	2.58E-05	0.555770594
cg24353443	PDZRN3	8.60E-06	0.555742162
cg12456714	MARCH11	1.11E-05	0.555713103
cg03062564		8.60E-06	0.555696485
cg20560075	PPP2R2B;PPP2R2B;PPP2R2B;PPP2R2B;PPP2	0.000102908	0.555696064
cg17397028	FAM190A	2.16E-05	0.555685673
cg19871940		8.60E-06	0.555682094
cg21646598	ADAMTS5	0.000123078	0.555611873
cg17426273	NEBL	5.59E-05	0.555555899
cg05513806	SOX2OT	8.60E-06	0.555551653
cg08480901		2.58E-05	0.555475518
cg04140663	CPEB1	8.60E-06	0.555454438
cg11528849	NEUROD1	2.08E-05	0.555450656
cg27109129	CXCL5	1.96E-05	0.555243428
cg06060191	LPPR4;LPPR4	8.60E-06	0.555193227
cg07124520	LRIG3;LRIG3	8.60E-06	0.555185833
cg11668923	ADAM12;ADAM12;ADAM12;ADAM12	1.18E-05	0.555080231
cg26841013	WNT3A	2.58E-05	0.555061462
cg25950325		1.11E-05	0.554969251
cg12392473	KCNB1	4.34E-05	0.55480254
cg23405575	SLC6A1	7.23E-05	0.554790083
cg15471953	SULT4A1	1.96E-05	0.55471842
cg17900854	C21orf88;C21orf88	9.04E-05	0.5546284
cg08288406	PLK5P	1.96E-05	0.554624834
cg22620090		8.60E-06	0.554621481
cg08049853		8.60E-06	0.55461073
cg05807991	TRAM1L1	1.50E-05	0.554571616
cg26422458	ELTD1;ELTD1	8.60E-06	0.554437791
cg25488669	PAK7;PAK7	8.60E-06	0.554332305
cg02432280		1.50E-05	0.554227403
cg25638997	SOX9	0.000148457	0.554224977
cg01431972	C10orf41;C10orf41	4.34E-05	0.554169881
cg05063999		1.11E-05	0.554158697

cg19523082	ARMC3	2.58E-05	0.554066761
cg16620382	TTBK1	0.000187194	0.554047676
cg07759394	GLB1L2;GLB1L2	4.34E-05	0.554012019
cg17269633	C17orf104	8.60E-06	0.553988962
cg22741823	SGMS2	9.24E-05	0.55395329
cg04711852	PAX9	3.21E-05	0.553925728
cg24896649	ZFP42	4.34E-05	0.553837559
cg17264670	RGS17	1.96E-05	0.553836556
cg25691167	FERD3L	1.96E-05	0.553822779
cg22546374		1.11E-05	0.553781419
cg06401021	HMGCLL1;HMGCLL1;HMGCLL1;HMGCLL1	1.96E-05	0.553743249
cg23290344	NEFM;NEFM	8.60E-06	0.55364901
cg02773086	HOXD3	4.34E-05	0.553622516
cg08854306	FAM190A	1.50E-05	0.553591723
cg14007067	SALL3	8.60E-06	0.553453661
cg25351606		8.60E-06	0.553421531
cg21819468	PDPN;PDPN;PDPN;PDPN	2.58E-05	0.553234007
cg27664844		3.34E-05	0.553172328
cg23495748	CPLX2;CPLX2	2.58E-05	0.553143812
cg11430157	SLC35F1;SLC35F1	1.50E-05	0.553024327
cg18582824	CNTNAP5	8.60E-06	0.552943576
cg25496678		1.11E-05	0.552936469
cg00221709		1.50E-05	0.552932956
cg21884231	SLC7A14;SLC7A14	1.96E-05	0.552862615
cg10182317	CLVS2	2.39E-05	0.552727914
cg07585876	ZFHX4;ZFHX4;LOC100192378	8.60E-06	0.552615958
cg10165167	SLIT3	1.96E-05	0.552607987
cg08797194	UGGT2	1.50E-05	0.552601759
cg03532926	DPP6	8.60E-06	0.552588678
cg02320740	SORCS3	1.11E-05	0.552568258
cg25958283	GNAL;GNAL;GNAL;GNAL	2.58E-05	0.552487555
cg14583673	FBXL7	8.60E-06	0.552302473
cg09935282	VAX1;VAX1	2.58E-05	0.551985028
cg08430009	GSC2	8.60E-06	0.551979423
cg21779611	SALL3	2.58E-05	0.551954789
cg07495363	BOLL;BOLL	1.50E-05	0.551945431
cg23042706	NR2F2;NR2F2;NR2F2;MIR1469;NR2F2	8.60E-06	0.551943897
cg27096779	MEOX2	4.34E-05	0.55193872
cg10831607		8.60E-06	0.551881796
cg21962423		8.60E-06	0.551823886
cg19254119	ZNF560	4.34E-05	0.551818915
cg08236767	ST6GAL2;ST6GAL2;ST6GAL2	1.11E-05	0.551816924
cg09167553		3.40E-05	0.551652006
cg13646020	PHOX2B	8.60E-06	0.551641027
cg08273957		0.000117986	0.551630037
cg23075337	NXPH1	8.60E-06	0.551621327
cg23751407		1.50E-05	0.55158894
cg20585530	SIX6	8.60E-06	0.551393399
cg22126211	SLITRK3	1.50E-05	0.551308212
cg23089825		1.18E-05	0.551308102
cg24727399	QRFPR	1.11E-05	0.551281758
cg16846645	FAM84A	8.60E-06	0.551276384
cg04188273	COL25A1;COL25A1	8.60E-06	0.551246779
cg23712342	PHYHIPL;PHYHIPL	1.50E-05	0.551222796
cg10317138	ADAM12;ADAM12	8.60E-06	0.551198422
cg11760593		1.96E-05	0.551139871
cg20114732	T	9.04E-05	0.551046038
cg10111292		8.60E-06	0.551006167
cg00740088		8.60E-06	0.551004786
cg14006181	C14orf23	1.96E-05	0.550849006

cg10341154	NPY5R	1.50E-05	0.550817498
cg20014049	PTF1A	1.96E-05	0.550811898
cg05256793	PRSS12	1.11E-05	0.550753438
cg13141009	PEX5L	8.60E-06	0.550438886
cg18252903		1.11E-05	0.55043705
cg21126583	CNTNAP2	3.34E-05	0.550425617
cg02469909	C22orf45;C22orf45;UPB1	8.60E-06	0.550411324
cg27142536		8.60E-06	0.550326703
cg18249634	TRIM71	2.58E-05	0.550284159
cg11898486	LYPD5;LYPD5	1.96E-05	0.550223807
cg25379026	DNAH14;DNAH14;DNAH14;DNAH14;DNAH14;[illegible]	8.60E-06	0.550208596
cg16437728	SYT9	1.96E-05	0.550092967
cg18529845	SRD5A2	4.34E-05	0.550091127
cg02253760		1.50E-05	0.549908967
cg17160382	GJA1	8.60E-06	0.549836555
cg01529149	FAM83H	3.34E-05	0.549615419
cg27202073	SAMD5	1.11E-05	0.549526089
cg05836965		1.96E-05	0.549501789
cg20214477	TLL1	1.50E-05	0.549492645
cg23011597	KCNH8;KCNH8	1.50E-05	0.549449143
cg20844851	VGLL2;VGLL2	1.50E-05	0.549446193
cg22749589		2.58E-05	0.54934566
cg17760405		5.59E-05	0.549339794
cg21026257	PHACTR2;PHACTR2;PHACTR2;PHACTR2	0.000117986	0.549337846
cg14743594	FBLL1	1.96E-05	0.54933499
cg14582400		8.60E-06	0.549291145
cg22367191		1.11E-05	0.549267715
cg07451080		1.96E-05	0.549219551
cg07508229	TBC1D12;TBC1D12	8.60E-06	0.549143364
cg00506811	SH3BP4	0.000290372	0.549078823
cg23881278	DRD2;DRD2	1.11E-05	0.549018577
cg19701540	NKX6-2	8.60E-06	0.549017975
cg05884032	SALL3	8.60E-06	0.548986722
cg13434411		8.60E-06	0.548902887
cg14005139	NKX2-1;NKX2-1	8.60E-06	0.548798031
cg04025150	C1orf94;C1orf94;C1orf94	8.60E-06	0.548775084
cg12698487	LRRC49;THAP10;THAP10	8.60E-06	0.548615962
cg20755651	SMOC2;SMOC2	3.34E-05	0.54859686
cg17288142	PCDHB4	1.11E-05	0.548570424
cg09469554	CLDN10;CLDN10;CLDN10	1.50E-05	0.548452447
cg04945216		0.000357885	0.548451834
cg02392553		2.58E-05	0.548282833
cg03958798	SORCS3	8.60E-06	0.548272344
cg19271111		2.58E-05	0.548146066
cg12662072	FAM159B	1.50E-05	0.548128334
cg00963169	ELAVL4	8.60E-06	0.547925066
cg17385936	TMEM108;TMEM108	1.50E-05	0.547841531
cg06323837	C10orf35	1.96E-05	0.547825436
cg23420260	HOXD1	8.60E-06	0.547807129
cg09868336	FAM19A4;FAM19A4	1.50E-05	0.54772243
cg01307130		5.59E-05	0.547553001
cg09839635	RNF180;RNF180	4.34E-05	0.547488241
cg00699945	NKX6-2	8.60E-06	0.547435377
cg15067806		1.96E-05	0.547399552
cg07849944	A2BP1;A2BP1	8.60E-06	0.547283954
cg11833861	TMEM98;TMEM98	1.50E-05	0.547229344
cg14817655	ZFP42	5.59E-05	0.547133329
cg02774186	CCBE1;CCBE1	1.50E-05	0.547032298
cg01667837	GPR37;GPR37	8.60E-06	0.546931062
cg03713642	HS3ST2	8.60E-06	0.546751013

cg24034005		2.58E-05	0.546702767
cg05655915	NARF;NARF;NARF;NARF	1.11E-05	0.546687162
cg23291305	SLC25A21;SLC25A21;SLC25A21;LOC1001297	8.60E-06	0.546629131
cg11855526	MPPED2	0.000570759	0.546588463
cg12974388		1.50E-05	0.546558767
cg06495961	DPP6;DPP6	1.11E-05	0.546421995
cg19792599	DDX25;PUS3;DDX25	4.90E-05	0.546398426
cg27018380	CHGA	1.11E-05	0.546385376
cg26369667	YAP1;YAP1	3.92E-05	0.546327883
cg08120263	FOXG1	1.11E-05	0.546224487
cg06781712	CDH18;CDH18;CDH18;CDH18	1.50E-05	0.546186009
cg22396555		1.11E-05	0.546168122
cg13198321	ALDH1A2;ALDH1A2;ALDH1A2;ALDH1A2	1.96E-05	0.546141129
cg20914464	NOL4	2.58E-05	0.546084285
cg08448665	C21orf88;C21orf88	4.34E-05	0.546042178
cg22889163		4.34E-05	0.54602894
cg17349389	CTNNA2;LRRTM1;CTNNA2	8.60E-06	0.546010726
cg18197377		1.11E-05	0.545990145
cg10853637	FOXQ1	1.11E-05	0.545957407
cg00865356	FAM190A	1.96E-05	0.545880677
cg22524061	OSR2;OSR2	7.23E-05	0.545833264
cg07349208		1.50E-05	0.545803511
cg08117309		4.34E-05	0.545775485
cg11410023	EPHA6	1.96E-05	0.545733745
cg07603330		2.58E-05	0.545566186
cg19156483	FIGN	0.000148457	0.54530814
cg25039722	CA10;CA10;CA10	1.11E-05	0.545146241
cg16278512	VWDE;VWDE	1.60E-05	0.54513472
cg24605553	ATP8A2	5.59E-05	0.545119791
cg15552843	C22orf45;C22orf45;UPB1	8.60E-06	0.545112055
cg17911318	GALR1	8.60E-06	0.545112029
cg27549952	KITLG;KITLG	8.60E-06	0.545050155
cg08368617		1.11E-05	0.545046572
cg05602862	FAM84A	2.58E-05	0.545026806
cg00765312	ALX1	2.58E-05	0.544938766
cg24021956	MAGI2	8.60E-06	0.544875619
cg21938261	PPP2R2B;PPP2R2B;PPP2R2B;PPP2R2B;PPP2	2.39E-05	0.544865208
cg22889573	KY	1.11E-05	0.544764328
cg16116512	SNX7;SNX7;SNX7;SNX7	8.60E-06	0.544734841
cg18384778		2.58E-05	0.544640266
cg20346912	BRUNOL4;BRUNOL4;BRUNOL4;BRUNOL4	4.34E-05	0.544587361
cg26446499		4.34E-05	0.544528002
cg18034737	ZFP42	3.34E-05	0.544525808
cg07907386	PHOX2B	3.34E-05	0.544518521
cg14486338	KCNS2	1.11E-05	0.544438203
cg09473510	MTNR1A	1.18E-05	0.54443175
cg24399830	FREM2	1.11E-05	0.544351796
cg12398645		2.58E-05	0.544233598
cg24033330	MAFB	2.58E-05	0.544180553
cg02523640	FOXI2	1.11E-05	0.544159423
cg07837556	TRAM1L1	8.60E-06	0.544151393
cg22489498	PROX1	1.11E-05	0.544140001
cg06087185		1.50E-05	0.543975045
cg12821804	KCNQ1DN	8.60E-06	0.543956399
cg27120816	CDH8	8.60E-06	0.543901889
cg04061117	LHFPL4	8.60E-06	0.543783366
cg05255522	TFAP2D	2.58E-05	0.543769422
cg14909730	TFAP2A;TFAP2A	0.000117986	0.543750001
cg02828023	ST6GAL2;ST6GAL2;ST6GAL2	8.60E-06	0.543737786
cg16963144	FOXA2	0.000117986	0.543567391

cg05034702	PCDH20	4.34E-05	0.543440394
cg18429863	KCNB2;KCNB2	3.34E-05	0.543346486
cg21635870	GRIK2;GRIK2;GRIK2;GRIK2;GRIK2;GRIK2	1.96E-05	0.543270277
cg13368756	CTNND2	1.96E-05	0.543169313
cg25124241	FEZF1;FEZF1	3.34E-05	0.543162372
cg21245277	ZNF804A;ZNF804A	8.60E-06	0.543023049
cg21563597	SEPT10;ANKRD57;SEPT10	1.96E-05	0.542990123
cg27553667	GABRG3	1.96E-05	0.542990063
cg03731268	L1TD1;L1TD1	1.50E-05	0.542914944
cg05849857	FREM2;FREM2	1.50E-05	0.54285665
cg11642106		1.96E-05	0.542801815
cg14326196	KIF12	5.59E-05	0.542727647
cg06177053	DTD1;DTD1	7.23E-05	0.542718474
cg02027945		0.000334462	0.542694742
cg00711088		8.60E-06	0.542679003
cg12188986	CCDC67;CCDC67	8.60E-06	0.5426667
cg04543008	PHOX2A	1.11E-05	0.542498675
cg27006650	FOXG1	4.34E-05	0.542476045
cg14394692		1.96E-05	0.542470659
cg19512268	GRM1;GRM1;GRM1;GRM1	8.60E-06	0.542455253
cg12523924		1.11E-05	0.542446817
cg03709792		5.59E-05	0.542435189
cg17721710	SLC30A10	0.000117986	0.542432038
cg12566756		8.60E-06	0.542410729
cg23889391	ENPP5;ENPP5	1.50E-05	0.542404985
cg25246431	PARVA	1.11E-05	0.542380426
cg22866015	GPR39	4.34E-05	0.54235695
cg18705773		8.60E-06	0.542327429
cg05733135	BDNF;BDNF;BDNF;BDNF;BDNF	8.60E-06	0.542237744
cg16718263		1.96E-05	0.542190291
cg03190219		1.50E-05	0.542179643
cg13685314	DBC1	8.60E-06	0.542120597
cg00250500		1.18E-05	0.542100778
cg05075118	TMEM108;TMEM108	1.11E-05	0.54194215
cg26092471	MSX2	8.60E-06	0.541804666
cg03161803		3.34E-05	0.541754454
cg26987597	FOXF2	3.34E-05	0.541410587
cg25570913		9.24E-05	0.541246457
cg14230666	ADAMTSL3;ADAMTSL3	8.60E-06	0.541196412
cg04397433	MALL;MALL	5.59E-05	0.541195067
cg08572611	ACTL6B	8.60E-06	0.541173123
cg19868631	VSTM2A	2.58E-05	0.541143697
cg05385513	EFEMP1;EFEMP1;EFEMP1	1.96E-05	0.541103037
cg13954457	FBLL1	8.60E-06	0.541051125
cg07225598	HIST3H2A	1.11E-05	0.540932168
cg10002178	PRIMA1	0.000233422	0.540828144
cg25527090	VAX1;VAX1	8.60E-06	0.540754888
cg05652533	DPYSL4	8.60E-06	0.540729685
cg23144681		1.18E-05	0.54069701
cg24996482		1.50E-05	0.540695931
cg22991512		4.34E-05	0.540693863
cg04609245	LIMCH1;LIMCH1;LIMCH1;LIMCH1;LIMCH1	4.34E-05	0.54053903
cg21620246		1.96E-05	0.540528596
cg10536999	NFE2L3	9.24E-05	0.54045194
cg15921949		8.60E-06	0.540411143
cg08130572	FYN;FYN	1.96E-05	0.540389243
cg07571509	CBLN1	1.50E-05	0.540298624
cg03171770		1.11E-05	0.540295134
cg05099387	HOXD1	8.60E-06	0.540203656
cg16183361	NMNAT2	4.34E-05	0.540113298

cg05809668		2.58E-05	0.54009034
cg12892303	C17orf104	8.60E-06	0.540053131
cg01188396	NKX2-8	1.96E-05	0.539937866
cg05789704	ADAM32	8.60E-06	0.539875911
cg12835048	FGFR2;FGFR2;FGFR2;FGFR2;FGFR2;FGFR2;I	1.11E-05	0.539657135
cg09441363	KCNK2;KCNK2;KCNK2	8.60E-06	0.539641211
cg27510182	DAB1	1.18E-05	0.539622293
cg13101087	GALNT13	1.11E-05	0.539511524
cg04837025	NID1	7.23E-05	0.539480515
cg00854242	GALR1	8.60E-06	0.539417976
cg06545361	FAM84A	1.50E-05	0.539380142
cg16065021		8.60E-06	0.539315748
cg14250833	GCM2	1.50E-05	0.539256866
cg02458000		1.50E-05	0.539138455
cg22796507	FOXE3	2.58E-05	0.539081701
cg10595547		8.60E-06	0.539054167
cg07352438	EPB41L3	9.24E-05	0.539010838
cg03563308	GRP;GRP;GRP	2.58E-05	0.538951938
cg19578835		4.34E-05	0.538934363
cg23291886		1.11E-05	0.538893957
cg08258526	EFCAB1;EFCAB1;EFCAB1;EFCAB1;EFCAB1	1.50E-05	0.538737173
cg05310764		1.96E-05	0.538735781
cg27272679		4.34E-05	0.538697022
cg01942962	GRIA2;GRIA2;GRIA2	8.60E-06	0.538483159
cg12915892	NCKAP5;NCKAP5	1.11E-05	0.538293206
cg20723355	FBXO39	4.34E-05	0.538170253
cg26354128	KIAA1409	9.24E-05	0.538019825
cg00017437		8.60E-06	0.537888043
cg16376000	FGF12;FGF12	8.60E-06	0.537873374
cg03745002	TOX3;TOX3	2.58E-05	0.537828309
cg01384143	C3orf14	8.60E-06	0.537790797
cg08364561		5.59E-05	0.53771417
cg05142982	C8orf42	5.59E-05	0.537709238
cg14944647	MIR129-2	8.60E-06	0.537691203
cg25557432	MACROD2	0.000148457	0.537678524
cg10788217	TTC12	5.59E-05	0.537499506
cg06875227	ISL1	1.50E-05	0.537486711
cg03451670	AGAP1;AGAP1	0.000357885	0.537406497
cg13914083	GRIA2;GRIA2;GRIA2	1.50E-05	0.537346555
cg23952663	LHX8	8.60E-06	0.537336321
cg04738965	ZIC1;ZIC1	1.11E-05	0.537206842
cg00530925		2.58E-05	0.537105637
cg00907842	FYB;FYB	5.59E-05	0.537099456
cg07204550	KCNIP4	8.60E-06	0.537072572
cg06034933	HIST3H2A;HIST3H2BB	1.96E-05	0.536902006
cg23021771	TRPC4;TRPC4;TRPC4;TRPC4;TRPC4;TRPC4	8.60E-06	0.536896978
cg25627226		4.34E-05	0.536855213
cg16664405		1.50E-05	0.536845997
cg23010538	FAM5C	0.000117986	0.536825439
cg15504461		2.58E-05	0.536798681
cg15916399	DOK6;DOK6	8.60E-06	0.536735811
cg26708319	PITX2;PITX2	9.24E-05	0.536631268
cg27252696	SIM1	8.60E-06	0.536596127
cg14698448		8.60E-06	0.536571372
cg03216846		4.34E-05	0.536556867
cg19807257		1.11E-05	0.536498308
cg06001519		9.24E-05	0.536449646
cg04549460	RSPO2	8.60E-06	0.536425104
cg22282405	TFAP2B	1.50E-05	0.53641426
cg09350341	MMP2;MMP2	5.59E-05	0.536353695

cg25918303	GUCY1A2	5.59E-05	0.536270796
cg11268983	PCDHA7;PCDHA13;PCDHA12;PCDHA6;PCDH/	1.18E-05	0.536233278
cg13690525	GOLSYN;GOLSYN	8.60E-06	0.536212078
cg15092561	C21orf33;C21orf33	1.50E-05	0.536182346
cg13205384	ANO5;ANO5	4.34E-05	0.536145726
cg25500080	PCDHAC2;PCDHAC2;PCDHA7;PCDHA12;PCD	4.34E-05	0.536049167
cg16976370	GABRA4	1.50E-05	0.535959012
cg27328797		8.60E-06	0.53585783
cg20208633		3.34E-05	0.535778141
cg06010588	UNC5D	2.08E-05	0.535761877
cg17410236	FLRT2;FLRT2	8.60E-06	0.53571015
cg00503383		1.11E-05	0.535637394
cg24654264	GULP1	1.50E-05	0.535533912
cg09500815	NR2F2;NR2F2;NR2F2;NR2F2;MIR1469	8.60E-06	0.535442232
cg20106459	COX6B2;COX6B2	2.58E-05	0.535429949
cg05951860	CTTNBP2	8.60E-06	0.535389431
cg13569486		9.04E-05	0.535378458
cg27511169	GOLSYN;GOLSYN	8.60E-06	0.535224875
cg10399099		2.58E-05	0.535190481
cg06765217		5.11E-05	0.535154258
cg07036370	PKIB;PKIB;PKIB	1.96E-05	0.535150795
cg19675288	T	2.58E-05	0.535112138
cg13826890	OTX2	8.60E-06	0.535061681
cg15449956	ZFPM2	0.000148457	0.534876961
cg16703956	SLC6A3	1.11E-05	0.534842776
cg04818943	LOC100130015;LOC100130015	0.000187194	0.534837335
cg04864807		1.11E-05	0.534793223
cg03278146	LOC642597	3.40E-05	0.534731634
cg23919479	MIPOL1;MIPOL1	1.11E-05	0.53469515
cg13065169	PLOD2;PLOD2;PLOD2;PLOD2	8.60E-06	0.534689651
cg05855618	SEMA3E;SEMA3E	1.50E-05	0.534667656
cg04283162	FAM189A1	7.23E-05	0.534664913
cg24183909	TRAM1L1	8.60E-06	0.534656541
cg24488891	LOC100128811;GPR158	4.34E-05	0.534643964
cg13678973	ELAVL4	1.11E-05	0.534628111
cg09968630	ANO5;ANO5	0.000117986	0.534506652
cg04148794	SHC2	1.11E-05	0.534452477
cg05697976	FAR2	8.60E-06	0.534372133
cg23045258	COL4A3;COL4A4;COL4A3;COL4A4;COL4A3;C(	3.34E-05	0.534345346
cg16269733	BCAN;BCAN	1.50E-05	0.534301343
cg14157844		3.34E-05	0.534218172
cg05732750		8.60E-06	0.534215384
cg09805010	THRB;THRB;THRB	0.000187194	0.534136514
cg09452082		1.50E-05	0.534115222
cg12189551	C14orf39	8.60E-06	0.534089065
cg03200166	SYT7	8.60E-06	0.534086368
cg10263682	SOX2OT	1.96E-05	0.534085465
cg12421755	ONECUT1	1.50E-05	0.534072299
cg13643914	QRFPR	3.34E-05	0.534055005
cg17438030		1.96E-05	0.53404678
cg16310415	EBF2	1.11E-05	0.53390416
cg10948359	ME1;ME1	5.59E-05	0.53387003
cg04217778	LOC389458	8.60E-06	0.533800928
cg24783211		7.23E-05	0.533761709
cg18183163	SP5	8.60E-06	0.533736221
cg07565505		8.60E-06	0.533724612
cg01408508		8.60E-06	0.533643782
cg25645064		5.59E-05	0.533609877
cg23807890	WDR69;WDR69	8.60E-06	0.5335534
cg14875171	NRXN1;NRXN1;NRXN1;NRXN1	3.34E-05	0.533519804

cg24876960	IRX4	1.11E-05	0.53347726
cg24567424	FAM155A	8.60E-06	0.53344381
cg03184290	PITX2;PITX2	5.59E-05	0.533306946
cg06424065		2.58E-05	0.533265344
cg07183372	SNX7;SNX7;SNX7;SNX7	8.60E-06	0.533233283
cg14317609	PAX5	3.34E-05	0.533129032
cg19760898	TMEM229A	8.60E-06	0.533074763
cg10040329	RFX4	8.60E-06	0.532977305
cg26515460	RSPO4;RSPO4	8.60E-06	0.532924579
cg24966702	DACT2	2.58E-05	0.532907274
cg18239431	EBF2	1.50E-05	0.532895291
cg03867465	MGC45800	0.000262362	0.532866728
cg18116971	BRUNOL5	8.60E-06	0.532767617
cg22821947		8.60E-06	0.532721734
cg15133719	PAX3;PAX3;PAX3;CCDC140;PAX3;PAX3;PAX3	8.60E-06	0.5327107
cg16933181	CHST9	4.34E-05	0.532709298
cg02189719	ENTPD7	1.11E-05	0.53270269
cg13952745	GRID2	8.60E-06	0.532697758
cg05197062	GALNTL4	8.60E-06	0.532679756
cg15118872		8.60E-06	0.532652598
cg00046499	GPR149	7.23E-05	0.532581865
cg02010196		8.60E-06	0.532471914
cg10106412	ID4	0.000187194	0.532382217
cg19864758	PCSK2	8.60E-06	0.532326153
cg25868998		3.34E-05	0.532286468
cg07333191		1.50E-05	0.532270324
cg08958294	GRM1;GRM1	9.24E-05	0.532141218
cg04710402	RUNDC3B;RUNDC3B;ABCB1;RUNDC3B	1.50E-05	0.532006418
cg04190870	UGT8	5.59E-05	0.531991978
cg06594404		2.58E-05	0.531986847
cg05583608		3.34E-05	0.531940977
cg27068490	TTC12	5.59E-05	0.531926888
cg26477488	HAND1	4.34E-05	0.531697928
cg17751435		5.59E-05	0.531681146
cg12219082	FAM19A4;FAM19A4	8.60E-06	0.531676072
cg24945701		8.60E-06	0.531620798
cg03817667	VSTM2A	3.34E-05	0.531614255
cg17010394	TTLL7	1.11E-05	0.531451893
cg15602740		2.58E-05	0.531432104
cg11439821	CTXN2	2.08E-05	0.531405206
cg16640855	NEUROD1	2.58E-05	0.531353728
cg03659519	GALR1	1.11E-05	0.531276873
cg05852231	ACTC1	4.34E-05	0.531181633
cg02646491	KCNQ1DN	8.60E-06	0.531149722
cg04319659		8.60E-06	0.531146986
cg00943360		1.11E-05	0.531126749
cg25192855	IRF6	2.39E-05	0.53112662
cg06183338	COL23A1	9.24E-05	0.531126431
cg12161228	NOX4;NOX4;NOX4;NOX4;NOX4;NOX4	8.60E-06	0.531102694
cg04336379	LOC389458	4.34E-05	0.531098828
cg19223299	PRDM13	3.34E-05	0.531090101
cg02595832	FOXI2	1.11E-05	0.531053947
cg26365545	OTP	8.60E-06	0.531021728
cg08453926	WASF3	0.000117986	0.531013754
cg21281009	ANKRD30B;ANKRD30B	0.003189589	0.530975712
cg00768993	FLJ32063	1.11E-05	0.530966958
cg24914278	TMEM74;TMEM74	1.96E-05	0.530900234
cg22770135	TRPC7;TRPC7;TRPC7	1.50E-05	0.530849193
cg06577205	FBXL7;FBXL7	8.60E-06	0.530841691
cg10092878	MLXIPL;MLXIPL;MLXIPL;MLXIPL;MLXIPL;MLXI	8.60E-06	0.530809781

cg24964368	FPR2	0.000117986	0.530786038
cg27348223	SLC6A3	8.60E-06	0.530764175
cg02769705	TMEM183B;TMEM183A	4.34E-05	0.530756124
cg03322161		2.58E-05	0.53074165
cg03533519		4.34E-05	0.530734708
cg10592245	KCNK2;KCNK2;KCNK2	1.60E-05	0.53072705
cg23989821	C14orf39	3.34E-05	0.530669388
cg11115235		8.60E-06	0.530650113
cg11544692	TMEM151A	1.96E-05	0.530579605
cg08065241	ERC2	4.34E-05	0.530553362
cg19288904		8.60E-06	0.530530064
cg21760402	TFAP2D	8.60E-06	0.530492444
cg23279522		2.58E-05	0.53047553
cg00178249	LOC100192378	8.60E-06	0.530303223
cg24891520	OLFM2	8.60E-06	0.530278554
cg19249107	PCSK9	3.34E-05	0.530222354
cg18614734	NR2F2;NR2F2;NR2F2;MIR1469;NR2F2	5.59E-05	0.530203335
cg03323696	PDE4D;PDE4D	0.000117986	0.530193579
cg16364709	ERN2	1.96E-05	0.530115538
cg24563570	PHYHIPL;PHYHIPL	8.60E-06	0.529988323
cg22305167		1.50E-05	0.529969841
cg00151810	BCAR1;BCAR1;BCAR1;BCAR1;BCAR1	8.60E-06	0.529787914
cg22723056	SYT9;SYT9	1.11E-05	0.529707562
cg13393978	MAGI2	2.58E-05	0.529684403
cg11338643	T	1.11E-05	0.529664664
cg23353952	NEFH	8.60E-06	0.529622713
cg13442960	ELAVL2;ELAVL2;ELAVL2	1.50E-05	0.529516387
cg02305765		8.60E-06	0.529270913
cg15711902		8.60E-06	0.529235464
cg25789277		1.11E-05	0.529229871
cg16703576	RUNDC3B;RUNDC3B;RUNDC3B;ABCB1;RUND	1.96E-05	0.529214346
cg10477621	FERD3L	1.11E-05	0.529144695
cg20388823	GRID2	1.50E-05	0.529140685
cg23302682	T	1.50E-05	0.529073384
cg23913679	LAMA1	8.60E-06	0.528991589
cg03226737	SYT9	1.11E-05	0.528948024
cg20593831		4.34E-05	0.528930274
cg10992014	GJA1	8.60E-06	0.528911924
cg20527270		0.000117986	0.528903835
cg09157727	NR2F2;NR2F2	8.60E-06	0.528751106
cg14334441		1.50E-05	0.528727721
cg18420965	EPHA5;EPHA5	1.96E-05	0.528701215
cg10143811	LMO3;LMO3	8.60E-06	0.528688406
cg00076538	EGFLAM	2.39E-05	0.528678633
cg10821115	ADAMTS19	1.50E-05	0.528671352
cg10601616	SORCS3	8.60E-06	0.52860023
cg10349065	WNT2;WNT2	8.60E-06	0.528581764
cg12145080	SGPP2	1.50E-05	0.528565258
cg20079899	MSC	8.60E-06	0.528544179
cg18392016	VGLL2;VGLL2	1.11E-05	0.528526037
cg00150882	AADAT;AADAT	0.000117986	0.528390436
cg24686610	ZIC4;ZIC1	0.000117986	0.528377848
cg08110874	DCLK1	1.96E-05	0.528289888
cg14643892	C12orf56;C12orf56	8.60E-06	0.528174668
cg14189141		8.60E-06	0.528085068
cg26942829	GFOD1	2.58E-05	0.528028707
cg26014391	PHYHIPL;PHYHIPL;PHYHIPL	8.60E-06	0.527946485
cg06630204	TMTC1;TMTC1	8.60E-06	0.527930257
cg06931815	GPR85;GPR85;GPR85;GPR85	8.60E-06	0.527898695
cg10741422		8.60E-06	0.527835295

cg10410142	GRIA2;GRIA2;GRIA2	1.50E-05	0.5277103
cg23440413	PCDHAC2;PCDHAC2;PCDHA7;PCDHA12;PCD	3.34E-05	0.52770222
cg01718742	CDH8	1.50E-05	0.527700161
cg21642176	RAD51AP2	1.11E-05	0.527624464
cg20738500	LOC254559	1.96E-05	0.527624416
cg19876814	PCDHA7;PCDHA12;PCDHA6;PCDHA10;PCDH/	1.11E-05	0.527608763
cg13002493	KRT19	2.58E-05	0.527557144
cg03650342	GPR158;LOC100128811	8.60E-06	0.527529003
cg17747005	PREX2;PREX2	1.96E-05	0.527482533
cg19129369	LRAT;LRAT	8.60E-06	0.527479904
cg17579667	LRRC4C;LRRC4C	1.50E-05	0.5274356
cg18780412	PEX5L	8.60E-06	0.527433991
cg19079194	ERBB4;ERBB4;ERBB4;ERBB4	1.18E-05	0.527358599
cg21278102	HSPBP1;HSPBP1	4.34E-05	0.527295867
cg13986130	PTGS2	8.60E-06	0.527201552
cg22299454	MARCH1	1.11E-05	0.527201255
cg19947104	KCNC1;KCNC1	1.11E-05	0.527138582
cg04652097	MAGI2	1.96E-05	0.526946446
cg05505803	DZIP1;DZIP1	1.11E-05	0.526852769
cg19766441	SLC34A2	3.34E-05	0.526774984
cg15084543	ELTD1;ELTD1	1.50E-05	0.52675526
cg04402007	UNC5D	8.60E-06	0.52675334
cg10982364		8.60E-06	0.526747387
cg11438011	HTR4;HTR4;HTR4;HTR4;HTR4;HTR4;HTR4	1.11E-05	0.52663413
cg02554246	ASTN2;ASTN2;ASTN2	5.59E-05	0.526547279
cg05994094	C18orf34;C18orf34	3.34E-05	0.526546791
cg00714184	LRAT	8.60E-06	0.526542031
cg07547549	SLC12A5;SLC12A5	8.60E-06	0.526521732
cg07034362	ALDH1L1	2.58E-05	0.526479906
cg11811513	ZNF804A	8.60E-06	0.526415714
cg27094244	SYT7	8.60E-06	0.52640882
cg25629773	RFX6	1.11E-05	0.526340793
cg24035245		8.60E-06	0.526261268
cg03225817	GRIA4;GRIA4;GRIA4;GRIA4	0.000205664	0.526130733
cg18789918	SLITRK1;SLITRK1	0.002450672	0.526014069
cg17437939	TAC1;TAC1;TAC1;TAC1	2.58E-05	0.525955487
cg21649442	SDR42E1	1.96E-05	0.525892993
cg00265634	MAPK15	1.11E-05	0.525766141
cg07914084	RYR2	9.24E-05	0.525759951
cg18577280	EFHA2	1.50E-05	0.525722322
cg04703241	SNX7;SNX7	1.50E-05	0.525675537
cg24590788		2.58E-05	0.525595851
cg19098763	ELAVL4	4.34E-05	0.525569834
cg05759718	AGBL4;AGBL4	1.96E-05	0.525564862
cg00594560	ZFHX4;LOC100192378	5.59E-05	0.525516157
cg23049458	L1TD1;L1TD1	1.50E-05	0.525496734
cg21242918	VAX1;VAX1	8.60E-06	0.525492564
cg24880701	LBXCOR1	8.60E-06	0.525437036
cg08706670	KCNS2	2.58E-05	0.525424406
cg02332982	DBX2	8.60E-06	0.525391614
cg12483476	GRM6	8.60E-06	0.525382204
cg20449590		0.000117986	0.525342772
cg18070676	FLRT2	4.34E-05	0.525296733
cg22799141		1.11E-05	0.525278014
cg18208707	B4GALNT2;B4GALNT2;B4GALNT2	1.50E-05	0.525213887
cg00839579	MEOX2	6.94E-05	0.525159774
cg05295557	NKX2-1;NKX2-1	1.18E-05	0.525125516
cg21453443	HIST3H2A;HIST3H2BB	1.50E-05	0.525094804
cg19519964	SGPP2	1.96E-05	0.525074302
cg11355215	SNAP25;SNAP25	3.92E-05	0.525048368

cg25831075	SLITRK3	2.58E-05	0.525011575
cg25311666	MYH14;MYH14;MYH14	8.60E-06	0.524975611
cg21241410		0.000117986	0.52496018
cg27583307	SATB2	0.000357885	0.52489405
cg20219381	RGS22	1.96E-05	0.524831197
cg01510903	KRT18;KRT18	3.34E-05	0.524810946
cg21179088	VSTM2A	8.60E-06	0.524731182
cg17202313	SOX2OT	9.24E-05	0.524575452
cg20755170	POU4F3	7.23E-05	0.524470183
cg03506640	FLRT2	5.59E-05	0.524448848
cg22855900		2.58E-05	0.524382237
cg13441730	PTPRD;PTPRD	2.58E-05	0.524303861
cg11702085		7.23E-05	0.524290608
cg14859204	SNX7;SNX7	7.23E-05	0.52425865
cg01939477	MIR129-2	1.96E-05	0.524256885
cg14417099	PCMT1	3.34E-05	0.524227761
cg11901043		8.60E-06	0.524219211
cg07161172	LRRC49;THAP10	1.11E-05	0.524201465
cg25678815	ALX1	1.96E-05	0.524122524
cg17199247	LRRN1	5.59E-05	0.524118477
cg17800654	DMRTA2	8.60E-06	0.52410976
cg02143877	CYP24A1;CYP24A1;CYP24A1;CYP24A1	4.34E-05	0.524091097
cg22531183	FLJ26850	8.60E-06	0.524020515
cg09599062		9.24E-05	0.523806992
cg10789050	OSBPL10	4.34E-05	0.523786275
cg18745566		1.11E-05	0.523756026
cg04559779	MIR124-2	8.60E-06	0.523684177
cg21355828		8.60E-06	0.523680575
cg22488256	PDE4B;PDE4B	8.60E-06	0.523640026
cg14507868	ST6GAL2;ST6GAL2;ST6GAL2	1.11E-05	0.523574189
cg02983424		8.60E-06	0.523495318
cg00987080	ZBTB8B	8.60E-06	0.523462007
cg24779941	CDC42BPB	2.58E-05	0.523405872
cg03905847	NKX6-2	8.60E-06	0.523359495
cg27211284		7.23E-05	0.523357217
cg13554207	LPAR3	1.96E-05	0.52334856
cg24011073	LPIN1	4.34E-05	0.523305613
cg22533517		1.11E-05	0.523284313
cg12656475	STEAP2;STEAP2;STEAP2;STEAP2	5.59E-05	0.523250973
cg18303215	ABCG5	1.18E-05	0.523221243
cg17641861		2.58E-05	0.523218668
cg02164046	SST;SST	1.50E-05	0.523200686
cg25950235	MIR9-3	2.58E-05	0.522975394
cg07382554	SALL3	1.11E-05	0.522950826
cg23191956	FOXG1	1.11E-05	0.522948893
cg03467725	C16orf73;C16orf73	2.58E-05	0.522872115
cg15617814	NTM;NTM;NTM;NTM	4.34E-05	0.522865115
cg01566526	ALDH1L1;ALDH1L1	1.50E-05	0.522659266
cg01837657	RHCG	1.96E-05	0.522640887
cg04478251	ABR;ABR;ABR	1.50E-05	0.522615947
cg26068551	PRKAA2	7.23E-05	0.522600362
cg17855264		1.50E-05	0.522579389
cg04224064		1.50E-05	0.522504484
cg17152484	GPR37	8.60E-06	0.522422889
cg07136998	SLIT3;SLIT3	4.34E-05	0.522408354
cg14591786	RNF180;RNF180	1.11E-05	0.52240319
cg21834207	AGBL4	1.11E-05	0.522339089
cg23867718	SASH1	8.60E-06	0.522313056
cg05884394	DPY19L2P4	3.34E-05	0.522220685
cg24306585	PCSK2	0.000187194	0.522214637

cg25851789	ESRRG	8.60E-06	0.522195156
cg02650266		8.60E-06	0.522188578
cg20168806		1.96E-05	0.522156689
cg03465206	CPE	1.96E-05	0.522138643
cg01718116	DPP10;DPP10	2.58E-05	0.522135991
cg05571581		1.96E-05	0.521982114
cg24851854	TACSTD2	8.60E-06	0.521974672
cg18898125	NEFM	8.60E-06	0.521868534
cg15818307		1.18E-05	0.521826325
cg12105941		2.58E-05	0.521799399
cg07077665		1.50E-05	0.521769495
cg13453904		9.24E-05	0.521742561
cg26635219	CFTR	5.59E-05	0.521739524
cg12926938		8.60E-06	0.521728939
cg14631386		1.18E-05	0.521640569
cg02970836		2.58E-05	0.521468628
cg02164615		9.24E-05	0.521442201
cg01898661	LOC100130015;LOC100130015	2.58E-05	0.521369896
cg01614102		1.11E-05	0.521344629
cg10282162	SHISA6	0.000117986	0.521339269
cg12166610	NRG1	1.96E-05	0.521291141
cg23998645	DPY19L2P4	1.11E-05	0.521277395
cg21484749	BRUNOL4;BRUNOL4;BRUNOL4;BRUNOL4	8.60E-06	0.521220192
cg01283246	FBXL21;FBXL21	3.34E-05	0.521206901
cg02072400		1.50E-05	0.521141681
cg02163885		0.000357885	0.521131604
cg19046725	LEPREL1;LEPREL1	1.50E-05	0.521121345
cg07850527		5.59E-05	0.52111682
cg23057648	C21orf88;C21orf88	3.34E-05	0.521115031
cg08086724	SFTA3	2.58E-05	0.521090564
cg16549308	NID1	9.24E-05	0.52103931
cg26682094	AGBL4	1.11E-05	0.521037306
cg18290848		1.50E-05	0.521033287
cg14442421	SLC24A2	8.60E-06	0.52103078
cg18343437		1.11E-05	0.520980195
cg02937674	LOC440040	8.60E-06	0.520941956
cg11163620	GLRB;GLRB;GLRB;GLRB	1.50E-05	0.520910015
cg03045425	NOL4	1.96E-05	0.520878217
cg00544449	KIAA1024	4.34E-05	0.520870126
cg24847685		4.34E-05	0.520836987
cg11769456	SNED1	5.59E-05	0.520803454
cg15259986	FIGN	1.96E-05	0.520755389
cg11648968	DDAH1;DDAH1	3.34E-05	0.520741924
cg09826050	ESRRG	3.34E-05	0.520728868
cg14200170	ADCYAP1;ADCYAP1	1.50E-05	0.520705068
cg09734791	MSC	1.50E-05	0.520694645
cg27504802	TRIM67	1.11E-05	0.520674895
cg25576011	FOXI2	1.50E-05	0.520622608
cg03357798		8.60E-06	0.520621439
cg15416735		7.23E-05	0.520559282
cg25657713		1.11E-05	0.520559074
cg08490115	KCNA4	4.34E-05	0.520554
cg15654121	CSMD2	3.34E-05	0.520551593
cg05041351	C16orf73;C16orf73;C16orf73	8.60E-06	0.520453723
cg07813142	SP5	4.34E-05	0.520359189
cg26296488	DRD5	0.002714607	0.520298274
cg10471574	SOX9	1.96E-05	0.520251001
cg10894483	SULT4A1	8.60E-06	0.520230927
cg00344443		2.58E-05	0.520219155
cg18442362	OGDH;OGDH;OGDH	7.23E-05	0.520100063

cg13122377		8.60E-06	0.520073742
cg25140190	C18orf1;C18orf1;C18orf1;C18orf1;C18orf1;C18o	1.96E-05	0.52007073
cg02149189		1.50E-05	0.520069672
cg03730958	FOXQ1	8.60E-06	0.51999554
cg07016184		5.59E-05	0.519983917
cg10135483	HTR1E;HTR1E	5.59E-05	0.519975516
cg00275741	PCDHA7;PCDHA12;PCDHA6;PCDHA10;PCDHA	1.11E-05	0.519961167
cg12633154	RNF39;RNF39	9.24E-05	0.519935254
cg24981833	FOXG1	1.18E-05	0.51989788
cg01710865	SPATA6	0.000233422	0.519771034
cg14537713		1.50E-05	0.519747553
cg11546137	HOXD11	8.60E-06	0.519435006
cg08768569	SORCS3	5.59E-05	0.519409612
cg09491410		8.60E-06	0.519407352
cg18249173	TBX20;TBX20;TBX20;TBX20	1.11E-05	0.519375338
cg04091563	HTR4;HTR4;HTR4;HTR4;HTR4;HTR4;HTR4	7.23E-05	0.519337994
cg03243226	GRIA4;GRIA4;GRIA4;GRIA4;GRIA4;GRIA4;GRI	4.34E-05	0.519308991
cg27510832		1.11E-05	0.519305163
cg18554789	HIPK2;HIPK2	1.96E-05	0.519228884
cg14638272	GULP1	8.60E-06	0.519203866
cg03671700	CDH8	8.60E-06	0.519141374
cg14409559	MSC	1.50E-05	0.51910387
cg06240450	ALDH1L1	3.34E-05	0.519037422
cg15786180	CACNG8	1.50E-05	0.518898435
cg11021744	SLC6A1	1.96E-05	0.51879422
cg12412079	FAM19A4;FAM19A4	1.50E-05	0.518788874
cg17450733	DBX2	8.60E-06	0.518783827
cg03779241	PHF21B;PHF21B	1.50E-05	0.518743455
cg05016408	LOC134466	9.24E-05	0.518696354
cg25317585	FGF14;FGF14	1.96E-05	0.518678591
cg17037282	ISL1	1.50E-05	0.518658395
cg13401893	RNF39;RNF39	0.000187194	0.518608867
cg02403395	FGF12	4.34E-05	0.518587596
cg20804050	EN1;EN1	2.58E-05	0.518567069
cg24167118	FREM2	8.60E-06	0.518534855
cg18143243	CACNA2D3	9.24E-05	0.518530524
cg00152008	DMRTA2	1.18E-05	0.518521179
cg08613144	LYPD1;LYPD1;LYPD1	7.23E-05	0.518510977
cg23248887	SSTR1	0.000117986	0.518506122
cg05051043	SLC35F3;SLC35F3	2.58E-05	0.518479755
cg02054792	FHL2;FHL2;FHL2;FHL2	1.11E-05	0.518475119
cg08322102	ZFHX4;LOC100192378	4.34E-05	0.518441846
cg05897091	SMOC2;SMOC2	1.96E-05	0.51838682
cg26014036	ZIC4;ZIC4;ZIC4	5.59E-05	0.518367695
cg13670833	KCNC3	1.96E-05	0.518320027
cg23068913		1.50E-05	0.518245077
cg15316843		1.96E-05	0.518150403
cg05293738	PREX2;PREX2	2.58E-05	0.518135042
cg14834938	ISL1	5.59E-05	0.518103755
cg08249988	NCAN;NCAN	1.96E-05	0.518047652
cg19053239	KIF5C	3.34E-05	0.517969377
cg17869514		1.11E-05	0.517899639
cg10248492		2.58E-05	0.517864037
cg24766334	SLC24A4;SLC24A4;SLC24A4	0.000290372	0.517860474
cg11797949	DNAH9	8.60E-06	0.517850689
cg14305278		8.60E-06	0.517717262
cg27628089	MATN2;MATN2	7.23E-05	0.517651727
cg01287975	TAC1;TAC1;TAC1;TAC1	1.50E-05	0.517635627
cg25720804	TLX3	8.60E-06	0.51760386
cg23230830	LOC646627	2.58E-05	0.517602119

cg07211212		1.50E-05	0.517377949
cg21895526	CHST9	2.58E-05	0.51736012
cg00463631	GRIA2;GRIA2;GRIA2	3.30E-05	0.517337835
cg11108269	NKX2-1;NKX2-1;NKX2-1	8.60E-06	0.517246189
cg02523844	MAGI2	8.60E-06	0.517234573
cg20034792		5.59E-05	0.517194532
cg06339629	AADAT;AADAT	9.24E-05	0.517185171
cg07870307	PCDHGA4;PCDHGA2;PCDHGB2;PCDHGA5;PC	4.34E-05	0.517167996
cg14794076		4.34E-05	0.517148986
cg04586579	SDR42E1	1.11E-05	0.517127188
cg24266670		1.50E-05	0.517025686
cg08274234	SEMA3E;SEMA3E	8.60E-06	0.517003659
cg03064592	COL21A1	8.60E-06	0.516986307
cg14454907		2.58E-05	0.516925754
cg19504702		1.11E-05	0.516883474
cg19725343	IGLON5	2.58E-05	0.516843779
cg08915603	MTNR1A	1.11E-05	0.516779119
cg10599900	REM1	1.11E-05	0.516733419
cg03839709	HS6ST3	8.60E-06	0.516714995
cg00948275	HIST3H2BB;HIST3H2A	3.34E-05	0.516697986
cg07556134	PPARG;PPARG;PPARG	5.59E-05	0.516676066
cg15379354	LGI2	9.24E-05	0.516667932
cg07120346		1.11E-05	0.516640253
cg05471845	CCK	3.34E-05	0.516534238
cg08530237		8.60E-06	0.516518509
cg13446474		3.34E-05	0.516475125
cg00604209	STK32A;STK32A;STK32A;STK32A	1.96E-05	0.516425647
cg01202150	PCDHA7;PCDHA12;PCDHA6;PCDHA10;PCDH/	1.18E-05	0.516401581
cg17138769	VAX1	3.34E-05	0.516382751
cg18271025	DNAJC6	1.11E-05	0.516376834
cg17364114	SLC22A3	1.50E-05	0.516274678
cg12757155		1.11E-05	0.516248473
cg15160198	CYP7B1	0.000148457	0.516241795
cg05895353		7.23E-05	0.516221886
cg17810176	GAPDHS;TMEM147	1.50E-05	0.516209799
cg21678377	DPP10;DPP10	0.000117986	0.516177403
cg11823511	BARHL2	1.11E-05	0.516158135
cg10443049	NXPH1	1.50E-05	0.516096526
cg01288372	PTH2R	8.60E-06	0.515964521
cg09083947		1.11E-05	0.515903608
cg07515422	SPTBN4	1.11E-05	0.515852966
cg07811110	SLC6A15;SLC6A15;SLC6A15	3.34E-05	0.515811598
cg22965511	PDLIM3;PDLIM3	1.11E-05	0.515717667
cg00379648	RFX4	5.59E-05	0.515693364
cg23217126	DOK6	8.60E-06	0.515679168
cg08087868		8.60E-06	0.515648646
cg18229521	SLC24A4;SLC24A4;SLC24A4	7.23E-05	0.515626359
cg22635008	OGDHL;OGDHL;OGDHL	2.58E-05	0.515624054
cg23267759	GATA6	3.34E-05	0.51560867
cg07336350		4.34E-05	0.515605479
cg08165960	CHSY1	2.58E-05	0.515558025
cg01580888	RHPN2	8.60E-06	0.515539258
cg04100595		0.000148457	0.515521301
cg12060713	FEZF1;FEZF1	8.60E-06	0.515474477
cg19031658	CXCL2;CXCL2	0.000537905	0.515470005
cg24540521	THEM4	9.24E-05	0.515406202
cg18945335		9.24E-05	0.515359489
cg21718267		1.11E-05	0.51534293
cg01303723	NKX6-2	1.60E-05	0.515291841
cg04138185	ADAMTSL3	3.34E-05	0.515227789

cg13458212	PDLIM3;PDLIM3	1.18E-05	0.515212823
cg16733705		1.96E-05	0.515184407
cg13131185		1.50E-05	0.515174461
cg22368664	P2RX2;P2RX2;P2RX2;P2RX2;P2RX2;P2RX2	1.11E-05	0.515095304
cg23489883	TMEM171;TMEM171	1.96E-05	0.51509504
cg00266983		0.000148457	0.515086321
cg27351358	BDNF;BDNF;BDNF;BDNF;BDNF	1.50E-05	0.515029655
cg22457984	PAK7;PAK7	3.34E-05	0.514996095
cg11538641	SIM1	2.58E-05	0.514963433
cg24087887	FREM2;FREM2	8.60E-06	0.514943722
cg08315202	NPTX2	4.34E-05	0.514925522
cg15412759	TFAP2D	2.58E-05	0.514915577
cg03264601		0.000117986	0.514882371
cg04733537	TMEM132D	8.60E-06	0.514847204
cg18501029		3.34E-05	0.514842948
cg17162024	FAM150A	1.11E-05	0.514819657
cg01610632	ZIC1;ZIC1	1.50E-05	0.514699861
cg15931721	C1QL2	2.39E-05	0.514695324
cg07087293	SCNN1B	0.000117986	0.514573955
cg10485664	FEZF1;FEZF1	1.96E-05	0.514565312
cg19854376	INTU	1.50E-05	0.514498914
cg22504204	PCSK2	4.34E-05	0.514484925
cg18854419		2.58E-05	0.514417622
cg23244289	THBS4	2.58E-05	0.514393112
cg24531494	C4orf31	3.34E-05	0.514366581
cg01142635		8.60E-06	0.514319738
cg17119521	LIN28B	8.60E-06	0.514306875
cg19704805	LMO3;LMO3	1.18E-05	0.514200848
cg16348470	C1orf94;C1orf94;C1orf94	1.11E-05	0.514189319
cg02935627	PPAP2C;PPAP2C;PPAP2C	4.34E-05	0.514171653
cg02801786		3.34E-05	0.514140157
cg17677030	NRXN1;NRXN1;NRXN1	2.58E-05	0.514086847
cg12597389	NXPH1	1.96E-05	0.514043227
cg14209299	ST6GAL2;ST6GAL2;ST6GAL2	8.60E-06	0.513993514
cg21643086		4.34E-05	0.513953901
cg01783070	PAX1	1.11E-05	0.513940029
cg19761272		1.11E-05	0.513907835
cg09797337		1.11E-05	0.513876865
cg07923233		0.000187194	0.513854579
cg08900101	TBX5;TBX5;TBX5;TBX5	5.59E-05	0.513848998
cg07295964	CPLX2	5.59E-05	0.513842516
cg13912117		3.34E-05	0.513835423
cg13478228	CBLN2	7.23E-05	0.513833428
cg24158187		9.24E-05	0.513747779
cg19822251	LHFPL4;LHFPL4	8.60E-06	0.513746656
cg10826999		1.50E-05	0.513682384
cg18233746	MGST1;MGST1;MGST1	8.60E-06	0.513587531
cg16006349	HOTAIR	1.96E-05	0.513511783
cg21229570		1.11E-05	0.513467565
cg07733446	GRID2	3.21E-05	0.513411575
cg10992267	LPHN2	0.000357885	0.513349021
cg11382529	LOC401463	8.60E-06	0.513242868
cg07302959	FAM133B;FAM133B	4.34E-05	0.513198093
cg24809973		1.11E-05	0.513187669
cg20661138		1.11E-05	0.512945115
cg10553088	CADM1;CADM1	3.34E-05	0.512935544
cg14348397		4.34E-05	0.512924916
cg26132298	C14orf39	8.60E-06	0.51285594
cg04453065	CYR61;CYR61	2.58E-05	0.512842751
cg02057391	TRPC4;TRPC4;TRPC4;TRPC4;TRPC4;TRPC4;	8.60E-06	0.512840901

cg14839351	BRUNOL4;BRUNOL4;BRUNOL4;BRUNOL4	8.60E-06	0.512737387
cg11312896	ZSCAN1	8.60E-06	0.512711112
cg02305377		0.000122184	0.512689501
cg12388309	PAK7;PAK7	3.30E-05	0.512658205
cg13273396	ACSS3;ACSS3	8.60E-06	0.512644441
cg19501982		8.60E-06	0.512628649
cg06960784	ESRRG;ESRRG;ESRRG;ESRRG;ESRRG	5.59E-05	0.512619012
cg07301433	CYP1B1	0.000955793	0.512607307
cg00966099		4.34E-05	0.512578282
cg13591783	ANXA1	3.34E-05	0.512523656
cg11327657	C21orf70	3.34E-05	0.512508553
cg18411108	ATXN7;ATXN7	2.58E-05	0.512490384
cg18750960	HOXD4	8.60E-06	0.512363056
cg25316339	ANKRD34B	1.50E-05	0.512310235
cg16572375	SHISA2	4.34E-05	0.512299543
cg27602263	RAX	1.50E-05	0.512275303
cg00380172	SASH1	1.96E-05	0.512192354
cg22626169		1.11E-05	0.512176461
cg12955441	CCDC68;CCDC68	8.60E-06	0.512160614
cg16543094		8.60E-06	0.512120604
cg22802813	USP44;USP44	8.60E-06	0.512008608
cg02849693	PRKCG	3.34E-05	0.512004803
cg06172871	HP;HP	1.11E-05	0.511987175
cg15076145	ATOH1	8.60E-06	0.511956898
cg01815538	PRDM13	1.18E-05	0.511879226
cg25737323	CLIP4	0.000117986	0.511851242
cg12877723	PENK;PENK	4.34E-05	0.511826609
cg26606286		1.11E-05	0.511807321
cg06981439	LOC100130015;LOC100130015	3.34E-05	0.511795832
cg19804605	FAM163A;FAM163A	1.50E-05	0.51177114
cg19237294	BRUNOL4;BRUNOL4;BRUNOL4;BRUNOL4	8.60E-06	0.511711264
cg00549772		1.96E-05	0.511661667
cg25597894	IGSF21	8.60E-06	0.511645753
cg22380921	CADM2;CADM2	8.60E-06	0.511620364
cg26609631	GSX1;GSX1	1.96E-05	0.511554249
cg22982654		1.50E-05	0.51142564
cg24911113		3.34E-05	0.511379706
cg15542741	SLC6A15;SLC6A15;SLC6A15	1.50E-05	0.511318667
cg26164488		5.59E-05	0.511292955
cg01307387	VSNL1;VSNL1	8.60E-06	0.511269803
cg08644841	FAM149A	0.000233422	0.511169914
cg08808128	CLIP4;CLIP4	0.000654035	0.511087457
cg04883903	NOS1	5.59E-05	0.511079385
cg14558114	THNSL2	8.60E-06	0.511055003
cg16783279	GPR26	8.60E-06	0.510992897
cg21669679	DCC;DCC	1.11E-05	0.510888048
cg05711235		1.11E-05	0.510821672
cg26836696		8.60E-06	0.510787854
cg21868774	FAM78B	0.000187194	0.510783427
cg20307896		1.50E-05	0.510667764
cg00490655	FIGN	2.58E-05	0.510665669
cg20219457	DCLK1;DCLK1	2.58E-05	0.510642529
cg05876623	NR2F2	8.60E-06	0.510544455
cg02315271		8.60E-06	0.510531784
cg04688351	PAX3;PAX3;PAX3;PAX3;PAX3;PAX3	9.24E-05	0.510498615
cg18844382	EFS;EFS	5.59E-05	0.510421884
cg04075191	DPP10;DPP10;DPP10	1.50E-05	0.510356071
cg10439765	SLC12A5;SLC12A5;SLC12A5	1.11E-05	0.510337245
cg21350575	CHODL;CHODL	1.96E-05	0.510275521
cg26872137	UNC5D	1.50E-05	0.510242442

cg18280830		1.11E-05	0.510229549
cg11382963	MSC	1.60E-05	0.51020725
cg09259037	TBX18	8.60E-06	0.510201639
cg18961681		1.96E-05	0.510192777
cg05139788	TFAP2D	1.50E-05	0.510169191
cg12919520	GABRG3	7.23E-05	0.510026818
cg18639233	SIX6	2.58E-05	0.509978892
cg14123942	GRIN3A;GRIN3A	2.58E-05	0.509943752
cg09499787		0.000148457	0.509939109
cg10923662	TNXB	0.000290372	0.509869558
cg22880677	ARHGEF10	0.000117986	0.509851608
cg09351156	RNF144B	0.000233422	0.509823653
cg04972745		0.000357885	0.509767808
cg25287071	ITPKB	5.59E-05	0.509717944
cg21402921	GABRA5;GABRA5;GABRA5	0.00043997	0.509699097
cg04976324	PROX1	0.000233422	0.509692313
cg01022219	C18orf1;C18orf1;C18orf1;C18orf1;C18orf1;C18o	8.60E-06	0.509668988
cg08833577	VAX1;VAX1	7.23E-05	0.509656261
cg06976250	ANKK1	9.24E-05	0.50963236
cg08034379	WDR69	1.18E-05	0.509586252
cg11737232		2.58E-05	0.509585095
cg00991875		8.60E-06	0.509561827
cg17903776	PCDH20	1.96E-05	0.509543238
cg14548882	MORN4;MORN4	3.34E-05	0.509337443
cg22836229	EFCAB1;EFCAB1;EFCAB1;EFCAB1;EFCAB1	1.50E-05	0.509279191
cg20027133	PAMR1;PAMR1	1.50E-05	0.509271075
cg20494374		8.60E-06	0.50920376
cg17985646	TBX20;TBX20	8.60E-06	0.509196896
cg13201808		1.50E-05	0.509129954
cg05627029		8.60E-06	0.509083319
cg01815671	CCDC37	1.60E-05	0.508989267
cg15985184	IGLON5	8.60E-06	0.508919764
cg01587896	ISL1	2.58E-05	0.508820631
cg09205945	CREB3L2	3.34E-05	0.508773512
cg23763043	NR2F2;NR2F2;NR2F2;MIR1469;NR2F2	1.96E-05	0.508768226
cg23152235		1.96E-05	0.508759122
cg19544662	FOXI3	4.34E-05	0.508728282
cg22987487	EBF2	4.34E-05	0.508723137
cg05206884	SYN3;SYN3	1.50E-05	0.508707813
cg14473924	PDZRN3	1.11E-05	0.508689281
cg00664416	MANEAL;MANEAL;MANEAL;MANEAL	1.96E-05	0.508613334
cg05500840	HOXD11	8.60E-06	0.508598543
cg19542816	HOXD1	8.60E-06	0.508515729
cg08540953	C7orf52	1.50E-05	0.5084931
cg10089527		3.21E-05	0.50844085
cg11398511	KCNK2;KCNK2;KCNK2;KCNK2;KCNK2	1.50E-05	0.508420846
cg12146673	SST	1.11E-05	0.508416151
cg04945331	SOX14	2.58E-05	0.508342063
cg03994318		1.11E-05	0.508337839
cg09863441	HTR1E;HTR1E	0.000148457	0.508337623
cg18177414		1.96E-05	0.508302181
cg23921342	CSMD2	1.96E-05	0.508273385
cg22531668	VAX1	1.96E-05	0.508253394
cg17305436	ID4	0.000148457	0.508185469
cg11007759	FAM84A	5.59E-05	0.508169263
cg10981909	FAM159B;FAM159B	9.24E-05	0.508163105
cg02300154	WBSCR17	0.000290372	0.508148435
cg02463418	EPHA5;EPHA5;EPHA5;EPHA5	9.24E-05	0.508141823
cg26560222	DMRTA2;DMRTA2	3.92E-05	0.508136651
cg09772154	FGFR2;FGFR2;FGFR2;FGFR2;FGFR2	7.23E-05	0.508125241

cg16086559		2.58E-05	0.508074019
cg20699586		8.60E-06	0.507950962
cg16596604	CRYM	9.24E-05	0.507908907
cg03691958	DRD2;DRD2	8.60E-06	0.507887781
cg03981954	FEZF1;FEZF1	8.60E-06	0.507878465
cg03217995	HOXA9	3.34E-05	0.507873874
cg02348751	MGC45800	2.58E-05	0.507801731
cg27606499	ZIC4;ZIC4;ZIC4;ZIC4;ZIC4	1.50E-05	0.507798577
cg05181041	ADAM12;ADAM12;ADAM12;ADAM12	1.50E-05	0.507783494
cg14248715	OTX2OS1	1.50E-05	0.507765828
cg09442654	FAM150A;FAM150A	1.96E-05	0.507762274
cg02012703	VSTM2B	1.96E-05	0.507614456
cg20222926	FEZF1;FEZF1	4.34E-05	0.507488031
cg27228578	DNAH9	8.60E-06	0.507410645
cg17112089	CTTNBP2	1.18E-05	0.507408473
cg04643822		3.34E-05	0.507313205
cg14658067	UNCX	2.58E-05	0.507305808
cg11781389	GLB1L2	8.60E-06	0.507143978
cg17177632	FAM123C;FAM123C;FAM123C;FAM123C	1.50E-05	0.507129881
cg11708616	FOXQ1;FOXQ1	8.60E-06	0.507075806
cg19427188	ACADL	2.58E-05	0.507068771
cg05311412	SLC5A7	5.59E-05	0.507062518
cg07333792		2.58E-05	0.507020349
cg12212657	LRRN1	3.34E-05	0.507016872
cg03943218		1.96E-05	0.506961913
cg07972135	GRIA4;GRIA4;GRIA4;GRIA4	0.000148457	0.50692005
cg13283952		1.50E-05	0.506894471
cg13971892	PPP2R2B;PPP2R2B;PPP2R2B;PPP2R2B;PPP2	1.96E-05	0.506872923
cg23349790	IGSF21;IGSF21	3.34E-05	0.506753658
cg13745201		1.11E-05	0.506714077
cg17509967	CACNA1A;CACNA1A;CACNA1A;CACNA1A	8.60E-06	0.506695869
cg05768427		2.08E-05	0.506652135
cg23121993	IRX4	4.34E-05	0.506605677
cg21106407		1.96E-05	0.506604547
cg00437985	CRYM	0.000233422	0.506586972
cg20280350	MCOLN3	8.60E-06	0.506559256
cg05937737	SLC7A14	1.50E-05	0.506527915
cg26686277	CCKBR;CCKBR	2.58E-05	0.50642064
cg22954449	ADAMTS16	5.59E-05	0.506417011
cg15996480		0.000117986	0.506351975
cg21153898		8.60E-06	0.506278936
cg13184823	MTNR1A	1.11E-05	0.506250462
cg11244340	HS6ST3	1.50E-05	0.506226662
cg18396137	FAM123C;FAM123C;FAM123C;FAM123C	2.39E-05	0.506138316
cg10167837	RAD51AP2	4.34E-05	0.506135307
cg11003573	ZNF502;ZNF502;ZNF502;ZNF502	1.11E-05	0.506007588
cg26252281	RAB32;RAB32	7.23E-05	0.505977563
cg09022943		8.60E-06	0.505943291
cg12744820	OLIG3	3.34E-05	0.505930919
cg09688601	LEPREL1;LEPREL1;LEPREL1	1.11E-05	0.505837146
cg07918545	FOXI2	1.50E-05	0.505832012
cg25012919	DCLK1	2.58E-05	0.505824694
cg21425099	CNTN4	8.60E-06	0.505821992
cg01645753	CPEB1	8.60E-06	0.505811712
cg13707794	RCAN1;RCAN1;RCAN1	3.34E-05	0.50572818
cg06833110	MEIS1	9.24E-05	0.505724459
cg06448705	TRPC7;TRPC7;TRPC7;TRPC7;TRPC7;TRPC7	4.34E-05	0.505687636
cg10165847	NPAS2	0.000117986	0.505613188
cg07547000	GALNT13	1.50E-05	0.505610733
cg18943957	HLF	0.000148457	0.505591576

cg02644510		0.000148457	0.505590272
cg03897252	ZNF365;ZNF365;ZNF365	1.50E-05	0.505555389
cg03044249	WBSCR17	0.000205664	0.505497636
cg23701643	PPFIA2	8.60E-06	0.505488019
cg06822816	MAL2	2.58E-05	0.505467694
cg21053831		8.60E-06	0.505450689
cg02374745	FBXO39;FBXO39	1.96E-05	0.505424562
cg16100120	EFEMP1;EFEMP1;EFEMP1	1.11E-05	0.505384278
cg24884142	TBX15;TBX15	8.60E-06	0.505383523
cg07926691	AMPH;AMPH;AMPH;AMPH	3.34E-05	0.505372287
cg07971089		0.000148457	0.505323936
cg27554565	NPHS1	1.96E-05	0.50532293
cg25288068		8.60E-06	0.505313475
cg01323964	SNORA22;CCT6P1	5.59E-05	0.505181215
cg03052970	CACNG4	0.000648283	0.505179286
cg15044957	KCNA4	2.58E-05	0.505160845
cg18057109		1.11E-05	0.505149086
cg11835068	AJAP1;AJAP1	9.24E-05	0.505106811
cg06911121	TBX5;TBX5	1.50E-05	0.504994408
cg04228468	SLITRK3	8.60E-06	0.504965349
cg07697895	WNT2;WNT2;WNT2	3.34E-05	0.504937468
cg04003136	HOXD1	8.60E-06	0.504911238
cg08169778		8.60E-06	0.504871618
cg06954658	SALL3	1.11E-05	0.504867365
cg24774306	CXCL6	1.60E-05	0.50486479
cg03564793	COL12A1;COL12A1	1.50E-05	0.504841164
cg00373707		1.96E-05	0.504763003
cg24452260	GRIA2;GRIA2;GRIA2	1.11E-05	0.504757369
cg15528437		8.60E-06	0.504754017
cg22991101	DZIP1;DZIP1	1.11E-05	0.504735995
cg24346629		8.60E-06	0.504717742
cg05062676	CYP1B1	0.000233422	0.504687377
cg07893544	KDR	2.58E-05	0.504654125
cg03077331	FN3K	1.11E-05	0.504636667
cg01921432	BARHL2	8.60E-06	0.504524106
cg16561893		1.11E-05	0.504512149
cg26040903	HSPA12A	1.50E-05	0.50450865
cg11414560	PHACTR3;PHACTR3	3.34E-05	0.50448073
cg00579717	RANBP17	8.60E-06	0.50447511
cg02246426	GDF6	9.24E-05	0.504467205
cg20750832		1.50E-05	0.504460876
cg02328010	RYR2	8.60E-06	0.504411634
cg00466268	SLITRK3	1.96E-05	0.504400288
cg23972738	MEIS1	8.60E-06	0.504366259
cg15042080	TMEM74;TMEM74	1.50E-05	0.504148936
cg23931421	SYNE1;SYNE1	2.58E-05	0.504113014
cg18866015	DCC	1.96E-05	0.504050319
cg19824907	GPC6;GPC6	4.34E-05	0.504019386
cg22295787	ADRA1A;ADRA1A;ADRA1A;ADRA1A	0.000148457	0.504015011
cg10541517	TMEM132C	4.34E-05	0.504005852
cg10700424	GLB1L2;GLB1L2	4.34E-05	0.503991865
cg07813249	GABRA2;GABRA2	2.58E-05	0.503984358
cg19312427	NEK7	1.96E-05	0.503971333
cg06585566		2.58E-05	0.503951999
cg20443254	ASCL4	4.34E-05	0.503864988
cg05078238	SNED1	7.23E-05	0.503847195
cg11382055	KCNK1	8.60E-06	0.503844973
cg03544320	CRMP1	4.34E-05	0.503833254
cg25048344	C1QL2	4.34E-05	0.503798887
cg27039662		1.50E-05	0.503778394

cg25403721		8.60E-06	0.503765929
cg00342532	KCNC3;KCNC3	1.60E-05	0.503713347
cg02649987	GUCY1A2	5.59E-05	0.503713269
cg14653643	LCA5;LCA5	0.000357885	0.503696068
cg23553442		8.60E-06	0.503687598
cg22354782	NGF	1.96E-05	0.50350502
cg07395354	MEOX2	8.60E-06	0.503473857
cg24886257		4.34E-05	0.50344458
cg19180624	HOXD1	8.60E-06	0.503439259
cg04628434	GPR126;GPR126;GPR126;GPR126	3.34E-05	0.503430146
cg07908870	C3orf59	8.60E-06	0.503397512
cg13169011		8.60E-06	0.503371521
cg26116485	WNT5A	1.96E-05	0.503362202
cg02167438	PAK7;PAK7	1.11E-05	0.503336716
cg26121591	CPLX2;CPLX2;CPLX2	5.59E-05	0.503314245
cg23460843		4.34E-05	0.503245748
cg24239148	C10orf107;C10orf107	4.34E-05	0.503235934
cg13371788	VGLL2;VGLL2;VGLL2;VGLL2	8.60E-06	0.503214622
cg23627335		3.34E-05	0.503211981
cg09654247		0.000148457	0.503198648
cg18488157		2.58E-05	0.503173128
cg26544742		1.11E-05	0.503147526
cg23474211	ZNF804B	7.23E-05	0.503145819
cg07625840	PRSS12	8.60E-06	0.503124323
cg18989709	SLITRK3	8.60E-06	0.503118892
cg24721899	NKX3-2	1.96E-05	0.503115307
cg03556653		0.000205664	0.503094385
cg25045219	C17orf97	0.000187194	0.503074657
cg04928005	THRB;THRB;THRB	5.59E-05	0.503013188
cg04532952	CA4	4.34E-05	0.502835377
cg14500486	FNDC1	4.34E-05	0.502765071
cg00804628	NXPH2	2.58E-05	0.502716602
cg04481181	SLITRK3	8.60E-06	0.502697092
cg27407147	LOC254559	2.58E-05	0.502683707
cg23379425	MAPK15	1.11E-05	0.502681333
cg21461100	NOVA2	8.60E-06	0.502664568
cg13833484		8.60E-06	0.502652521
cg14510812	SLC6A15;SLC6A15;SLC6A15	8.60E-06	0.502543782
cg26916166	NMNAT2;NMNAT2	4.34E-05	0.502537242
cg18815943	FOXE3	4.34E-05	0.502537198
cg25756435	MAST1	8.60E-06	0.50251488
cg10774877	NALCN	1.11E-05	0.50239199
cg20637307	ERBB4;ERBB4	5.59E-05	0.502336786
cg23234832	THRB;THRB;THRB	0.00043997	0.502312077
cg27029179	CADM1;CADM1	3.34E-05	0.502282808
cg05085500		8.60E-06	0.50228021
cg26938597		1.50E-05	0.502271123
cg04805619	ZBBX	8.60E-06	0.502269331
cg03147210	THEM4	9.24E-05	0.502172531
cg19861741		8.60E-06	0.502137283
cg06501832	ROBO3	9.24E-05	0.5021165
cg24569447		8.60E-06	0.502087801
cg12352124		1.50E-05	0.501967484
cg15490715		1.18E-05	0.501831781
cg12727398		0.000148457	0.501813151
cg00335712	ATOH1	2.58E-05	0.50174253
cg25382400	SOX2OT	1.11E-05	0.50167929
cg19962116	NUDT16P;NUDT16P	8.60E-06	0.501642731
cg02441747	COL25A1;COL25A1;COL25A1;COL25A1	8.60E-06	0.501538361
cg24496021	FCHSD2	4.34E-05	0.501527437

cg11531021	OBSL1	7.23E-05	0.501517389
cg22484737		4.34E-05	0.501453733
cg17716664	LMX1A	1.18E-05	0.501453172
cg12417685	FAM19A4;FAM19A4	2.58E-05	0.501382389
cg00399483	DCC	1.11E-05	0.501363186
cg12041848	EYA2;EYA2;EYA2;EYA2	0.000122184	0.501349458
cg04454086	PCDHAC2;PCDHA7;PCDHA12;PCDHA6;PCDH	8.60E-06	0.501318948
cg11664500	EYA4;EYA4;EYA4	0.000233422	0.501299172
cg12605662	RAX	4.34E-05	0.501277522
cg07612562	CNTNAP2;CNTNAP2	1.96E-05	0.501251447
cg17500587		1.11E-05	0.50122461
cg26886381	DZIP1;DZIP1	2.16E-05	0.501211125
cg06411724	MEOX2	8.60E-06	0.501211121
cg11421604	MAP1B	2.58E-05	0.50120681
cg12545687	PCDH8;PCDH8	8.60E-06	0.501172742
cg02250594	ONECUT2	1.11E-05	0.501099103
cg25385529	MPPED2	2.58E-05	0.501093057
cg11142389	ZIC4;ZIC1	8.60E-06	0.500990906
cg09670128	KRT7;KRT7	4.34E-05	0.500962423
cg14480116	SPRED2;SPRED2	0.000117986	0.500937356
cg26745332	ATOH1	2.58E-05	0.500914402
cg13337047		8.60E-06	0.500895322
cg01904183	TRAM1L1	1.11E-05	0.5008718
cg02640260	ATOH1	1.96E-05	0.500866388
cg13974632	BDNF;BDNF;BDNF;BDNF;BDNF	2.58E-05	0.500828789
cg23774356	GSC2	5.59E-05	0.500761829
cg16400794	CD164L2	9.24E-05	0.500702908
cg00310968	SPAG17	1.50E-05	0.500702368
cg06802658	MIPOL1	8.60E-06	0.50069115
cg10293925	AMPH;AMPH;AMPH;AMPH	4.34E-05	0.500686231
cg02378440		4.34E-05	0.500634592
cg25828350	LOC201651	1.96E-05	0.500606793
cg09103619	ANKRD34C	3.21E-05	0.500564045
cg12598235	LOC254559	3.34E-05	0.50052586
cg07570470		5.59E-05	0.500482838
cg20309794	CLDN3	3.21E-05	0.50037803
cg06269753	MSC	8.60E-06	0.500348779
cg07115626		1.11E-05	0.500342342
cg24172269	IGSF9B	1.50E-05	0.500334275
cg08154348	RIPPLY2	2.58E-05	0.500288161
cg23696752	GRM1;GRM1	3.34E-05	0.500280069
cg24834659		1.11E-05	0.500266931
cg24164564		1.11E-05	0.500257825
cg18273840	HCN1	2.58E-05	0.500222769
cg09834794		1.96E-05	0.500188349
cg18115040	HOXD10	8.60E-06	0.500187483
cg02725885	PPAP2C;PPAP2C;PPAP2C	0.000117986	0.500171731
cg25439496		1.96E-05	0.500170077
cg26983177		8.60E-06	0.500163939
cg05502701	PDZRN3	1.50E-05	0.500130341
cg00384539	PRDM14	3.34E-05	0.500086963
cg17130063	KCNK2;KCNK2;KCNK2	1.11E-05	0.500082968
cg25177452	TMEM171;TMEM171	2.58E-05	0.500061027
cg23620822	LOC285375	0.000233422	0.500056041
cg27615388	HTR1A	1.96E-05	0.500009386
cg09735723	SORCS3	1.50E-05	0.499900852
cg27416337	CNOT10	4.34E-05	0.499781014
cg07291836	TP53RK	0.000117986	0.499712165
cg24439256	LOC100128811;GPR158	8.60E-06	0.499706758
cg08230483	GRID1	7.23E-05	0.49963349

cg00636427	NFATC1;NFATC1;NFATC1;NFATC1;NFATC1	7.23E-05	0.499578476
cg02837536	AJAP1;AJAP1	0.000187194	0.499543609
cg05283542	COL8A1;MIR548G;COL8A1	1.50E-05	0.499516894
cg13410614	SLC2A6;SLC2A6	1.11E-05	0.499434541
cg24403845	SORCS1;SORCS1	0.000290372	0.499410154
cg07115542	NKX2-1;NKX2-1	1.96E-05	0.499371585
cg03860256	MOV10L1;MOV10L1;MOV10L1	1.96E-05	0.499363012
cg02290110	SHC2	8.60E-06	0.499336113
cg23676439	LRAT	8.60E-06	0.4993337
cg09874822	CACNA1H;CACNA1H	0.000187194	0.499281791
cg21678813		8.60E-06	0.49923349
cg05755408	TRIM35	5.59E-05	0.499231232
cg18235734		8.60E-06	0.499208823
cg22620221	DPP6	8.60E-06	0.499110273
cg16856049		8.60E-06	0.499093643
cg19613722	DCC	8.60E-06	0.498991462
cg12234855	CLDN3	8.60E-06	0.498970772
cg06490869	CRYM	0.000233422	0.498962927
cg21266502		2.58E-05	0.498926709
cg01861537		4.34E-05	0.498852673
cg04118306	NEFM;NEFM	0.000290372	0.498848635
cg11979589	COL25A1;COL25A1	8.60E-06	0.498838399
cg27663938	C12orf42;C12orf42	5.59E-05	0.498829781
cg25051341	PRDM13	8.60E-06	0.498827999
cg02658488	REM1	2.58E-05	0.498824712
cg11916609	IL1RL1	4.34E-05	0.498800043
cg02789965	PLK5P	8.60E-06	0.498763855
cg24722073		8.60E-06	0.498741646
cg08732352		0.000334462	0.498735917
cg19873491	FAM149A	0.000955793	0.498703959
cg25067242	NGF	1.96E-05	0.498689905
cg18326021	SORCS3	8.60E-06	0.498635065
cg16750440	C10orf79	9.24E-05	0.498604079
cg05120028	CYP24A1;CYP24A1	5.59E-05	0.498567714
cg03418552	CADM1;CADM1	8.60E-06	0.498545613
cg14184078	CBLN4;CBLN4	8.60E-06	0.498534131
cg16046980	KRT18;KRT18;KRT18	1.96E-05	0.498518344
cg10324219	GLB1L2	2.08E-05	0.498487756
cg05003422	MCOLN3	1.18E-05	0.498487133
cg16849609	C1QL2	1.50E-05	0.498477064
cg03793270	NOX4;NOX4;NOX4;NOX4	3.34E-05	0.498453634
cg10778841	SORCS3	8.60E-06	0.498427239
cg22135105	PRDM5	1.50E-05	0.49837581
cg18470839		1.11E-05	0.498309123
cg07201456	CHST6	7.23E-05	0.498284038
cg27549720	HTR6;HTR6	5.59E-05	0.498266496
cg13906811	NRXN1;NRXN1;NRXN1	2.58E-05	0.49825461
cg25976242	SLC35F1	8.60E-06	0.498251531
cg11878331		3.34E-05	0.498214577
cg20492807	DNAH14;DNAH14;DNAH14	5.59E-05	0.498187256
cg10125626	FOXQ1	8.60E-06	0.498147675
cg24663256	KCNIP4;KCNIP4	2.58E-05	0.498115918
cg15545772	TDRD5;TDRD5	7.23E-05	0.498103369
cg14298200		2.58E-05	0.498077131
cg04487296		8.60E-06	0.498044266
cg18197392	SSTR4	5.59E-05	0.498022465
cg27375072	SMPDL3A	1.11E-05	0.498012902
cg18345806	NMU	7.23E-05	0.497908767
cg03356747	GRID1	4.34E-05	0.497864512
cg04487855		8.60E-06	0.497795334

cg17697633	TFAP2A	5.59E-05	0.497795047
cg11964564	KCNS2	0.000117986	0.497743145
cg23900203		8.60E-06	0.497739905
cg12825070	HTR4;HTR4;HTR4;HTR4;HTR4;HTR4;HTR4;HT	1.11E-05	0.497719255
cg02388150	SFRP1	2.58E-05	0.497710406
cg24202123	STAC	5.59E-05	0.497582917
cg07245225		2.58E-05	0.497516121
cg11028201		2.58E-05	0.497476633
cg12001304	SPHKAP;SPHKAP	8.60E-06	0.49746896
cg23322933		7.23E-05	0.497420739
cg01978237	SIM1	8.60E-06	0.497415835
cg21149266	FAM63B;FAM63B	9.24E-05	0.497409648
cg12111714	ATP8A2	1.11E-05	0.497396737
cg20232986		8.60E-06	0.497347306
cg04293902		8.60E-06	0.497297146
cg03900143	ZIC4;ZIC4;ZIC4;ZIC4;ZIC4	8.60E-06	0.497241043
cg08233654	SNED1	9.24E-05	0.497232603
cg18710278	CDH18;CDH18	2.58E-05	0.497227205
cg10166283	ZNF229	0.000117986	0.49721719
cg07737781	MLXIPL;MLXIPL;MLXIPL;MLXIPL;MLXIPL;MLXI	8.60E-06	0.49717229
cg19042459	DCC	1.11E-05	0.497130443
cg11955474	CD164L2;CD164L2	5.59E-05	0.497130286
cg17017591		8.60E-06	0.497088302
cg19268498	CCDC39	2.58E-05	0.497030792
cg22630875	DOCK5	1.96E-05	0.497016129
cg26249106	RFX6	1.50E-05	0.496987804
cg09780241		0.000148457	0.496951017
cg25201811	NALCN	2.58E-05	0.496828316
cg19991022	DOK5	0.00156564	0.496800613
cg03205140	PLEKHA1	2.58E-05	0.496746204
cg06247406	GRIK2;GRIK2;GRIK2	1.11E-05	0.496731684
cg20107759	RTBDN;RTBDN	1.50E-05	0.496668458
cg07157830	NID1	4.34E-05	0.496646481
cg15202954	NALCN	9.24E-05	0.496460838
cg26355573	LOC642597	8.60E-06	0.496411715
cg00812438		1.96E-05	0.496397838
cg20961469		2.58E-05	0.49639667
cg03769371	LCA5;LCA5	0.000233422	0.496370001
cg15484988	GALR1	1.11E-05	0.496368812
cg08161323	CXCL1	8.60E-06	0.496248771
cg05965392	FAM150A	8.60E-06	0.496221051
cg14878520	MNX1;MNX1	8.60E-06	0.496206458
cg13632816	SVEP1;SVEP1	8.60E-06	0.49611256
cg09423283	GRM1;GRM1	8.60E-06	0.496094595
cg08992872	CALB1;CALB1	4.34E-05	0.496019209
cg08356637		5.59E-05	0.495988884
cg07237939	SLC22A3	1.96E-05	0.495976024
cg21184369	HTRA4;PLEKHA2	7.23E-05	0.495952846
cg27135692	RTN1;RTN1;RTN1	1.50E-05	0.495930642
cg20148575		0.000233422	0.495854495
cg25732462	LYPD5	4.34E-05	0.495805654
cg09852127	PCDHA12;PCDHA7;PCDHA6;PCDHA10;PCDHA	1.11E-05	0.49579972
cg08462988		1.96E-05	0.495772214
cg06697267	FOXA2;FOXA2	2.58E-05	0.4957405
cg13062406	PTPN5;PTPN5;PTPN5	1.96E-05	0.495582016
cg14502484	SLC6A3	1.96E-05	0.495580043
cg03738352	SLC34A2	4.34E-05	0.495533778
cg00989765	KDR	3.34E-05	0.495522826
cg23873669	RANBP17	8.60E-06	0.495476273
cg17912835		1.96E-05	0.495469968

cg12535280	PARVA	8.60E-06	0.495351063
cg23649708	RALYL;RALYL;RALYL;RALYL	2.80E-05	0.495331921
cg08917478	ISL1	8.60E-06	0.495311849
cg19965221	TJP1;TJP1;TJP1;TJP1	8.60E-06	0.495269977
cg21295575	TM7SF4;TM7SF4	5.59E-05	0.495255827
cg24312063		8.60E-06	0.495180489
cg26827247	CCDC67	8.60E-06	0.495116084
cg19157819	SYNPO2L;SYNPO2L	3.34E-05	0.495098516
cg26144658	SNCAIP	3.34E-05	0.495092818
cg09578475	ZNF177	2.58E-05	0.495061404
cg02093112	FBXO39	7.23E-05	0.495024137
cg06000951	MOCS1;MOCS1;MOCS1	2.58E-05	0.495021388
cg07305933	PVT1	1.50E-05	0.49502092
cg18023283	SLC6A15;SLC6A15;SLC6A15	8.60E-06	0.494993834
cg16002355	PITX2;PITX2;PITX2	3.34E-05	0.494992472
cg17463745		1.96E-05	0.494992404
cg14344261	PAX5	0.000233422	0.494980986
cg11354594	STOX2;STOX2	2.58E-05	0.494972833
cg05378938	TDRD5	5.59E-05	0.494954784
cg24724428	ELOVL2	4.34E-05	0.494932637
cg22871668	EYA4;EYA4;EYA4	0.000955793	0.49488383
cg27315556	NLGN1	8.60E-06	0.494850312
cg18920423	PTF1A	1.50E-05	0.494817621
cg05604079	QRFPR	3.21E-05	0.494799178
cg05133179		1.96E-05	0.494703733
cg25430838		4.34E-05	0.494660951
cg05666607	POU4F3	5.59E-05	0.494566837
cg08939095	BNC1	1.11E-05	0.494539239
cg12746059	PCDH10;PCDH10	5.59E-05	0.494510192
cg12781700	C17orf104	2.58E-05	0.494440357
cg20574436	BCL11B;BCL11B	7.23E-05	0.494411498
cg07448060	MAGI2	1.11E-05	0.494322947
cg23746497		2.58E-05	0.494316205
cg19922137	SYT14;SYT14;SYT14;SYT14;SYT14;SYT14;SY1	8.60E-06	0.494278268
cg13791254	FOXE1	3.34E-05	0.494224328
cg02853616		1.11E-05	0.494216331
cg21187769	GRID2	1.50E-05	0.494211803
cg26963271	PDE4B;PDE4B	1.60E-05	0.494202249
cg01068944		4.34E-05	0.494188683
cg12560987		8.60E-06	0.494094594
cg17673237	FAM155A	1.11E-05	0.494045994
cg11826826	PPP2R2B;PPP2R2B;PPP2R2B;PPP2R2B;PPP2	4.34E-05	0.494028584
cg00690148	C1QL2;C1QL2	0.000117986	0.494023979
cg17397150		2.58E-05	0.493969408
cg06374165	FGF12	7.23E-05	0.493949388
cg07052390	IGSF9B	4.34E-05	0.493901585
cg03718824		3.34E-05	0.493887206
cg18437365		0.000357885	0.493862397
cg18011364	RELN;RELN	8.60E-06	0.493857193
cg27112897	WSCD1	0.000233422	0.493852539
cg07615497		0.000117986	0.493852083
cg05191076	EPHA5;EPHA5	2.58E-05	0.493845521
cg10755235		8.60E-06	0.493789279
cg26660002	GALNT13	8.60E-06	0.493734273
cg17382198	STL	0.003189589	0.493648371
cg19355087	NKX6-2	3.30E-05	0.493626865
cg23842255	NEFH	8.60E-06	0.493622308
cg27321750	ARHGAP8;PRR5-ARHGAP8;ARHGAP8	2.58E-05	0.493587792
cg10374084	C19orf41	1.96E-05	0.493582971
cg04204831	LOC255167;LOC255167	8.60E-06	0.493509851

cg04688051	LYNX1;LYNX1;LYNX1;LYNX1	1.50E-05	0.493500035
cg22450968		8.60E-06	0.493485487
cg01082512		1.11E-05	0.493458913
cg02286091	BHMT;BHMT	2.58E-05	0.493397078
cg03682581	C2orf68	9.24E-05	0.493336907
cg16108230		1.96E-05	0.493336667
cg23663774	PITX2;PITX2	0.00043997	0.493226402
cg01405040	HOXD12	1.11E-05	0.493206917
cg10059660	KCTD8	8.60E-06	0.493188933
cg00777689	KCNK1	0.000117986	0.493162718
cg25942450	TLX3	8.60E-06	0.493155954
cg18327061	CLVS1	8.60E-06	0.493101536
cg04290346	DBX1	7.23E-05	0.493060805
cg20168751	FAM149A	0.000148457	0.492928487
cg16052198	FPR2	7.23E-05	0.492915388
cg18294691		1.50E-05	0.492868019
cg00582524	SALL1;SALL1;SALL1	0.00043997	0.492852912
cg11377136	PKDREJ	5.59E-05	0.49282139
cg01201519		8.60E-06	0.492792365
cg26595278		1.11E-05	0.492747438
cg25165358	GALR1	1.96E-05	0.492747291
cg26280713	THBS2	1.96E-05	0.492705554
cg18240143	C14orf39	3.34E-05	0.492689051
cg26252167	GPR6	1.18E-05	0.492672318
cg07029873	ESRRG;ESRRG	2.58E-05	0.49261911
cg08813099	MRAS;MRAS	1.96E-05	0.492610932
cg22653976	MYOD1	3.21E-05	0.492574269
cg14018471	C9orf156	7.23E-05	0.4925372
cg20611882	GPR177;GPR177	1.96E-05	0.492501192
cg03963198	IRX4;IRX4	8.60E-06	0.492466797
cg14271531	ID4;ID4	0.000117986	0.492437834
cg06550984	PTF1A	1.50E-05	0.492406134
cg14189571	ZFP42;ZFP42	1.50E-05	0.492375451
cg22389375	KCNQ1DN	1.11E-05	0.492358087
cg25049597	CPLX3;CPLX3	8.60E-06	0.492339259
cg16629702	SLC6A2	1.50E-05	0.492307138
cg23615676	KCNN2	1.50E-05	0.492306757
cg16877924	NOTO	3.34E-05	0.492285992
cg11592503	CNTNAP2	2.58E-05	0.492276063
cg04899175	NOS1	3.34E-05	0.492273586
cg11369071	LOC100270746;C6orf41	7.23E-05	0.492175978
cg00619126		8.60E-06	0.492119415
cg01277542	SLC6A2	8.60E-06	0.492054621
cg26133769	PCDH7;PCDH7;PCDH7	8.60E-06	0.492028327
cg14923640	NXPH2	1.11E-05	0.492001261
cg23890008		3.34E-05	0.491960723
cg04617948	FOXI2	1.60E-05	0.491950003
cg02995271	FAM83H	4.34E-05	0.491829478
cg11842415	LHX8	8.60E-06	0.491754338
cg02642914	CADM1;CADM1	1.50E-05	0.491733019
cg27403635	KCNN2;KCNN2	1.50E-05	0.491701034
cg09605164		1.50E-05	0.491602791
cg08659357	HRASLS;HRASLS;MGC2889	8.60E-06	0.491596277
cg24041541	LOC100192378	8.60E-06	0.491584852
cg22049569	CACNG8	1.96E-05	0.491553474
cg06312848	KBTBD2	9.24E-05	0.491492841
cg21161891	KDR	3.34E-05	0.491446012
cg18716164	VSTM2B	3.34E-05	0.491437423
cg03308628	USP44;USP44	1.11E-05	0.491386227
cg26527487	LPPR5;LPPR5	1.11E-05	0.491373612

cg04476927		1.50E-05	0.49131886
cg26595643	VAX1;VAX1	2.58E-05	0.491314773
cg09360770	FOXC2	0.000148457	0.491314133
cg23651826		8.60E-06	0.491276696
cg17959327		5.59E-05	0.491188051
cg13473356	PEX5L	8.60E-06	0.491173541
cg01899437	CCDC149;CCDC149	0.000290372	0.491145612
cg24881420	TLX3	8.60E-06	0.491127305
cg07604651	FEZF1;FEZF1	8.60E-06	0.491120058
cg27526665	THRB;THRB;THRB	0.00043997	0.491070017
cg02230017	A2BP1;A2BP1	1.11E-05	0.491060787
cg10964367	ARHGEF10	0.000117986	0.491032134
cg00289546	ACADL	3.34E-05	0.491019686
cg02974320	TPBG;TPBG	0.001147425	0.490990798
cg16257051		2.58E-05	0.49099005
cg07719492	PRDM14	1.96E-05	0.490985628
cg07915921		7.23E-05	0.490910288
cg16556145	CLDN10;CLDN10;CLDN10	1.11E-05	0.490897057
cg18396984	NKX2-8	8.60E-06	0.490881056
cg00596508	CNTN1;CNTN1	1.11E-05	0.490817754
cg05361559	EGR3	0.000117986	0.49081473
cg24842260	C10orf41;C10orf41	8.60E-06	0.490813937
cg24561661	HOXD11	8.60E-06	0.490804781
cg23428985	CLIP4	0.000357885	0.490756579
cg14688104	KCNS2;KCNS2	7.23E-05	0.49071059
cg25943503	DCLK1	3.21E-05	0.490678938
cg20377305	DLX5	5.59E-05	0.490673199
cg00840332	LEP	1.96E-05	0.490662862
cg15285250	NXPH1	8.60E-06	0.490642749
cg06375628		7.23E-05	0.490580528
cg20959460		8.60E-06	0.490576514
cg08305436		8.60E-06	0.490536088
cg06648277	NKX6-2	8.60E-06	0.490491715
cg14996220	ALX1	2.39E-05	0.490485885
cg02344833	ZSCAN1	1.11E-05	0.490472361
cg01448863	SLC10A1	4.34E-05	0.490443785
cg07354556	NMU	7.23E-05	0.490354606
cg21649258	ST6GAL2;ST6GAL2;ST6GAL2	1.18E-05	0.490347326
cg27230882		1.96E-05	0.490337158
cg18410685		0.000187194	0.490285156
cg24504349		1.50E-05	0.490196359
cg06831576	CDH8	4.34E-05	0.490195462
cg03078043	SYT12	1.50E-05	0.490193554
cg04807594	AGTR1;AGTR1;AGTR1;AGTR1;AGTR1;AGTR1	2.58E-05	0.490129097
cg25497250	GPR120	5.59E-05	0.490070124
cg10197862	TMEM20;TMEM20	3.34E-05	0.490054194
cg13671919	LOC642597	1.11E-05	0.490044279
cg16919569	NBLA00301;HAND2	1.96E-05	0.490034371
cg17124829		1.96E-05	0.490015441
cg09559189	EBF2	4.34E-05	0.489991125
cg25372296		2.58E-05	0.489945205
cg15707093	NBLA00301;HAND2	3.34E-05	0.489918254
cg15237494	ADAMTS5;ADAMTS5	1.11E-05	0.489902149
cg18663063	MARVELD2;MARVELD2	1.96E-05	0.48989499
cg23141355		5.59E-05	0.489842506
cg05589784		7.23E-05	0.489831456
cg15461105		2.39E-05	0.4898312
cg14616514	MOCS1;MOCS1	3.34E-05	0.489762303
cg14306734	BRUNOL4;BRUNOL4;BRUNOL4;BRUNOL4	7.23E-05	0.489706588
cg25092838	OTX2OS1	8.60E-06	0.489687131

cg14123543		1.11E-05	0.489677329
cg20026939		1.50E-05	0.489675359
cg04741853		5.59E-05	0.489656572
cg04624110	MACROD2	7.23E-05	0.489650537
cg22869726	OLIG2;OLIG2	5.59E-05	0.489600032
cg08461949		3.34E-05	0.489571769
cg08058544		2.58E-05	0.489526393
cg17877704		1.11E-05	0.489488905
cg04926361	SFTA3;SFTA3	5.59E-05	0.489470886
cg24248713	NOL4	8.60E-06	0.48943201
cg18136062	RORA	0.000290372	0.489424529
cg27600205	PCDH10;PCDH10	5.59E-05	0.489380978
cg17616554	CFTR	1.11E-05	0.489352199
cg21176048	PEX5L	1.50E-05	0.489345825
cg14383135	NPAS2	7.23E-05	0.489342636
cg02019444	ITSN1;ITSN1	1.50E-05	0.489318186
cg18247055	SPAG6;SPAG6	3.92E-05	0.489178165
cg15978561	HDAC4	4.34E-05	0.489172468
cg22876812		0.000233422	0.489169327
cg24569492		8.60E-06	0.489150048
cg24292761		2.58E-05	0.489084094
cg17641904	C9orf4	3.34E-05	0.489060694
cg13084677	LOC401127	1.11E-05	0.489046083
cg05948940	SMPD3	0.00043997	0.489042069
cg18512515		7.23E-05	0.489022376
cg24881205	SP8;SP8	0.000187194	0.489000244
cg23733177	ADAMTS1	5.11E-05	0.488933723
cg23604012	SGPP2	7.23E-05	0.488931465
cg14142007	HOXD9	1.50E-05	0.488931451
cg22043168	BDNF;BDNF;BDNF;BDNF;BDNF;BDNF	1.96E-05	0.488928799
cg02456288		8.60E-06	0.488868189
cg18990313	HTRA4	1.96E-05	0.48885248
cg07857251	BICC1	1.96E-05	0.488794738
cg21183256		1.96E-05	0.488792291
cg12161122	BRUNOL4;BRUNOL4;BRUNOL4;BRUNOL4	8.60E-06	0.488784081
cg19975933	P4HA3	1.50E-05	0.488762587
cg03440556	SCD	2.80E-05	0.488755595
cg19878482	C8orf73	1.96E-05	0.488707202
cg27448015	SMPDL3A	1.50E-05	0.488695689
cg18786593		0.000357885	0.488688209
cg16289355	FAM149A	7.23E-05	0.488680191
cg07099331		1.96E-05	0.488670311
cg23181844	TMEM196	7.23E-05	0.488642781
cg03958038	C10orf41;C10orf41	8.60E-06	0.488575336
cg16042149	NEFH	8.60E-06	0.488532776
cg18060185	TMEM171;TMEM171	1.96E-05	0.488485953
cg15361750	GPR77	0.000187194	0.488408559
cg03799283		8.60E-06	0.488398574
cg17940587	MINPP1	0.000187194	0.488393901
cg02571816	PPP1R14A	0.000205664	0.488331898
cg03625109	DBC1	1.96E-05	0.48832411
cg14885748		1.50E-05	0.488300398
cg20090283	SRCIN1	2.58E-05	0.488262481
cg04281464		5.59E-05	0.488244595
cg25718467	LOC145845	2.58E-05	0.488223869
cg04663790		7.23E-05	0.488208882
cg16190732	CHST8;CHST8;CHST8;CHST8	5.59E-05	0.488179013
cg20191310	C20orf103;C20orf103	0.000262362	0.4881644
cg15173134	SLC18A2	4.34E-05	0.488138861
cg17885226		8.60E-06	0.488119072

cg13272701	MAL2	8.60E-06	0.488115486
cg23479922	MARCH11	1.96E-05	0.488098499
cg00495860		4.34E-05	0.488091968
cg11849798	ZNF706;ZNF706;ZNF706	0.000148457	0.488031742
cg07222309		1.96E-05	0.488008256
cg03099208	NXPH1	8.60E-06	0.487992206
cg08608974	PRKD1;PRKD1	1.96E-05	0.487979879
cg18478319	SLC15A1	4.34E-05	0.487959767
cg23786625	SORCS3	8.60E-06	0.487941095
cg11779273	INTU	5.59E-05	0.487918619
cg20902817		8.60E-06	0.487884926
cg02447304		8.60E-06	0.487830935
cg27363327	TTBK1	0.00043997	0.487812446
cg06719445	FOXN3	0.000117986	0.487770659
cg08445263	GABRG3	2.58E-05	0.487745844
cg18061259	RFX4	8.60E-06	0.487738984
cg26682580	TMEM132C	2.58E-05	0.487728832
cg18132916		9.24E-05	0.487724281
cg21512644	NPY2R;NPY2R	1.11E-05	0.487714482
cg27376707	A2BP1;A2BP1	8.60E-06	0.48769386
cg17942639	RPRM;RPRM	7.23E-05	0.487580173
cg16105687		1.96E-05	0.487554996
cg22958090	KRT7	5.59E-05	0.487515441
cg05863502	CACNA1B	1.50E-05	0.48750773
cg20906291		8.60E-06	0.487506837
cg14882700	OTOP1	1.50E-05	0.48747915
cg13046832	C14orf23	3.34E-05	0.487434952
cg25829666	THRB;THRB;THRB	0.000308169	0.48742784
cg23142394	LOC283392;TRHDE;LOC283392	9.24E-05	0.487344384
cg19971716	QRFPR	4.34E-05	0.48731784
cg05821789		5.59E-05	0.487307006
cg07551545	FBXO17;FBXO17	1.96E-05	0.487272977
cg01827633	TTLL4	1.96E-05	0.487231975
cg20398486		0.000148457	0.4872116
cg20122925		1.11E-05	0.487191398
cg05700339	CIDEA;CIDEA	7.23E-05	0.487135281
cg25634742	RAB32	7.23E-05	0.487125476
cg15447787		4.34E-05	0.487071044
cg07780543		1.11E-05	0.487066782
cg02128567	KCNK5;KCNK5	7.23E-05	0.487061995
cg18166862	FAM78B	4.34E-05	0.487059515
cg14386496	EPHA5;EPHA5	5.59E-05	0.487055315
cg19714279	GBX2	1.50E-05	0.487032637
cg02306630	NKX6-2	8.60E-06	0.486997603
cg12853633		1.96E-05	0.486994454
cg24613080	ACCN1	9.24E-05	0.486992618
cg25999442	CHST8;CHST8	0.000117986	0.486976646
cg08119884	CTNNA2;CTNNA2	4.34E-05	0.486967374
cg20780105	C5orf43	3.34E-05	0.486926617
cg09403666		1.11E-05	0.48685848
cg26831119	PITX2;PITX2	2.58E-05	0.486834654
cg19945554		8.60E-06	0.486798145
cg05475440	NLK	5.59E-05	0.486765448
cg27563985	CBLN4	4.34E-05	0.486741424
cg10200388		4.34E-05	0.486726482
cg27456468	FLJ32063	3.34E-05	0.486660381
cg24052359		8.60E-06	0.486660371
cg16949120	NKX6-2	8.60E-06	0.48665509
cg06003986	BRUNOL4;BRUNOL4;BRUNOL4;BRUNOL4	0.000117986	0.486615798
cg19806642	GRM6	0.000148457	0.486585799

cg10679182	EXOC4	3.34E-05	0.486543234
cg17991823	CIDEA;CIDEA	2.08E-05	0.486525063
cg03064067	SLC6A15;SLC6A15;SLC6A15	1.11E-05	0.486511643
cg24416513	HOXD8;HOXD8	0.00043997	0.486463592
cg14009588	TFAP2A;TFAP2A	9.24E-05	0.486397113
cg08202754		7.23E-05	0.48638881
cg04562217	ROBO1	8.60E-06	0.486379735
cg09547099	WWC1;WWC1;WWC1	4.34E-05	0.486379184
cg18325622	MARCH11	3.34E-05	0.486353655
cg23070026	ADAMTS9	0.000233422	0.486331596
cg24843474	RGS7	5.59E-05	0.486316096
cg18710929	SLC35F1	4.34E-05	0.486273219
cg18194734	FAM123C;FAM123C;FAM123C;FAM123C	0.000148457	0.486244846
cg11098259	AQP9	1.96E-05	0.486243761
cg19925849	CACNG7	8.60E-06	0.486243265
cg18781240	WASF3	0.000117986	0.48622869
cg15142192	EIF2C3;EIF2C3	5.59E-05	0.48619409
cg17397592	IQCA1	3.34E-05	0.486165685
cg24772753	SP5	3.34E-05	0.486152357
cg22694818	LHX8	1.50E-05	0.486127079
cg26861460	PARVG;PARVG;PARVG;PARVG	5.59E-05	0.486005215
cg10715092	MIR663	8.60E-06	0.485974546
cg17504999	PCDH10;PCDH10	8.60E-06	0.485965125
cg15015920	MARCH11;MARCH11	1.96E-05	0.485958183
cg16073349	CAMK2B;CAMK2B;CAMK2B;CAMK2B;CAMK2B	0.000122184	0.485931731
cg14763548	VSX1;VSX1	0.000117986	0.485908027
cg10980436	NPHS2;NPHS2	1.50E-05	0.485874689
cg04935434		1.11E-05	0.485804317
cg01229798	GRID1	0.000117986	0.485785854
cg21748223	NPY5R	7.23E-05	0.485760468
cg11751645	LRRC55	1.96E-05	0.485758237
cg22442454	IRF6;IRF6	3.34E-05	0.485748497
cg12882697	SLC6A3	8.60E-06	0.485730205
cg15132565		5.59E-05	0.485717844
cg11390378		8.60E-06	0.485702924
cg03705912	MARCH11	8.60E-06	0.485677752
cg15715477		4.34E-05	0.485659658
cg10958452	KDM4A	9.24E-05	0.485651066
cg16771578	ALDH1L1	4.34E-05	0.485587226
cg00557947		9.24E-05	0.485581982
cg02036261	OTX2	0.00043997	0.485574792
cg24163575		8.60E-06	0.485554724
cg21609106	EGFLAM	3.21E-05	0.485512633
cg16573782	CBLN1;CBLN1	7.23E-05	0.485506507
cg21537235	PCDHAC2;PCDHA7;PCDHA12;PCDHA6;PCDH	1.11E-05	0.485504882
cg11581865		3.34E-05	0.485484089
cg15672437	F7;F7	3.92E-05	0.485469319
cg10463150	HELT	2.58E-05	0.485436339
cg23619365		7.23E-05	0.485366223
cg25123102	CDH22	8.60E-06	0.485308446
cg07535928	SLC25A21;SLC25A21;LOC100129794	8.60E-06	0.485275002
cg04842426	CHRM2;CHRM2;CHRM2;CHRM2;CHRM2;CHRI	5.59E-05	0.485263455
cg10795659	TMEM171;TMEM171	2.58E-05	0.485213206
cg08124446		1.11E-05	0.485175832
cg08482682	RAB39	5.59E-05	0.485148417
cg22747746	NKX2-6	5.59E-05	0.485122213
cg07201620	EDIL3	1.50E-05	0.485107191
cg13434308	TMTC1	1.60E-05	0.485077268
cg05926722		3.34E-05	0.485035092
cg12861945	C4orf39;TRIM61	0.001393182	0.485001423

cg20941820	ADAMTS17	0.000537905	0.484993383
cg15025240	C2orf65	9.24E-05	0.484983231
cg02606840	MGAT4A;MGAT4A	4.34E-05	0.484978063
cg00791024		8.60E-06	0.484905257
cg11492102		3.34E-05	0.484896128
cg00631837	PHF21B;PHF21B	1.11E-05	0.484833582
cg17316316		1.50E-05	0.484829762
cg24329557	GCM2	1.11E-05	0.484821195
cg24864161	MOV10L1;MOV10L1;MOV10L1	4.34E-05	0.484800982
cg08610901		0.000537905	0.484766245
cg12478384	ADAMTS9	0.000187194	0.484731349
cg00056074		1.50E-05	0.484717006
cg22501388	PDZRN4	0.000148457	0.484693497
cg06809544	MAGI2	2.58E-05	0.484691635
cg24324985	FOXA2	2.58E-05	0.484659427
cg21475834	UGGT2	8.60E-06	0.484620483
cg02215641	KCNIP4;KCNIP4;KCNIP4;KCNIP4	8.60E-06	0.484609615
cg02629106	PCDP1	3.34E-05	0.484609528
cg27199820	GRM7;GRM7;GRM7;GRM7	1.96E-05	0.484600637
cg24947764		1.50E-05	0.484591526
cg08330404		3.34E-05	0.484546798
cg01429039	SPATA18	4.34E-05	0.48448581
cg13533340	GDA	1.50E-05	0.484451555
cg23528247	C3orf26;FILIP1L;FILIP1L;C3orf26	3.34E-05	0.484406236
cg15485728	PRUNE2	8.60E-06	0.484345269
cg09696939	BICC1	1.50E-05	0.484339432
cg23466339	MRAS;MRAS	2.58E-05	0.484263403
cg06622999	LOC283392;TRHDE;LOC283392	5.59E-05	0.48421515
cg20072171	FEZF2	0.000148457	0.48419588
cg04021697	WDR8	9.24E-05	0.484190298
cg27578811	CPEB1	5.59E-05	0.484138444
cg03540175	CCDC36;CCDC36;CCDC36	1.11E-05	0.484050409
cg00503302		7.23E-05	0.483978653
cg23657299		8.60E-06	0.483957746
cg14168923	GSX1	5.59E-05	0.483925818
cg09614415	FOXI2	2.58E-05	0.483922023
cg04674956		9.24E-05	0.483881651
cg04967578	C18orf1;C18orf1;C18orf1;C18orf1;C18orf1;C18o	8.60E-06	0.483841707
cg27458987	PKP2;PKP2	1.96E-05	0.483840204
cg22558303	UGGT2	1.11E-05	0.483823171
cg07152216		0.000290372	0.483802639
cg23891360	TMEM132D	2.58E-05	0.483802292
cg26119543		1.50E-05	0.483791783
cg26983710	GALNT13	1.96E-05	0.483734819
cg23642130	ZNF536	2.58E-05	0.483707276
cg02409351	ALX1	8.60E-06	0.483664458
cg16822939	MATN4;MATN4;MATN4	2.58E-05	0.483570793
cg08696727	MKX	1.96E-05	0.483569659
cg22028075		1.11E-05	0.483540107
cg25134283	SMOC2;SMOC2;SMOC2;SMOC2	7.23E-05	0.483535238
cg22604123	ZBTB8B	2.80E-05	0.483523842
cg10887386		2.58E-05	0.483501441
cg00871358		1.50E-05	0.483498139
cg19236679	GFRA1;GFRA1;GFRA1	0.000233422	0.483416423
cg06737494	GHSR;GHSR	5.59E-05	0.483333233
cg10652393	GABRA5;GABRA5;GABRA5	0.000148457	0.483327399
cg22474464	NKX2-2	0.000117986	0.483324789
cg24937747	IRX4	8.60E-06	0.483313939
cg16400492	PCDHA7;PCDHA12;PCDHA6;PCDHAC1;PCDH	1.50E-05	0.483222852
cg03036557	GPC5	2.58E-05	0.48322103

cg04176888	CYP2A13	4.34E-05	0.483195796
cg24188415	EMX2;EMX2OS;EMX2	4.34E-05	0.483182109
cg04227922		1.96E-05	0.483141003
cg25951981	GABRA4	1.96E-05	0.48302515
cg15575222	RAD21L1	0.000187194	0.483005509
cg16619395	GPR149	5.59E-05	0.482986849
cg21197774	FGF12;FGF12;FGF12	1.11E-05	0.482985577
cg27296293	TRPC6	1.50E-05	0.482909377
cg24506862	BRUNOL4;BRUNOL4;BRUNOL4;BRUNOL4	1.50E-05	0.482861968
cg24199834	POU4F2;POU4F2	1.50E-05	0.482837095
cg21751684	LOC254559	2.58E-05	0.482833747
cg24871414		3.34E-05	0.482812621
cg01627823	PAX9	3.34E-05	0.482805431
cg14524936	SLC6A5	8.60E-06	0.482780443
cg18840956	PCSK1	2.58E-05	0.482759783
cg18678353	PTPN5;PTPN5;PTPN5	2.58E-05	0.482727498
cg21545862		3.34E-05	0.482586155
cg10758057		9.24E-05	0.482547309
cg03834767	CDK14	2.58E-05	0.482539681
cg18021902	WDR8	4.34E-05	0.482494636
cg08194879	CLVS2	8.60E-06	0.482483603
cg08346159	NPY5R	5.59E-05	0.482427727
cg20600210	APCDD1L	1.96E-05	0.482345056
cg16331920	MEOX2	8.60E-06	0.482335885
cg07441518		8.60E-06	0.482304186
cg10790429		8.60E-06	0.48228568
cg08620154		8.60E-06	0.482281364
cg14451382		1.11E-05	0.482277565
cg17603132	RIC3;RIC3	2.58E-05	0.482233704
cg27603796	CTTNBP2	8.60E-06	0.482155368
cg11154070	BRUNOL4;BRUNOL4;BRUNOL4;BRUNOL4;BR	8.60E-06	0.482126901
cg10174867	DNAH14;DNAH14;DNAH14;DNAH14;DNAH14;D	8.60E-06	0.481957085
cg00564737		1.11E-05	0.481811824
cg08882038		0.000148457	0.481799063
cg22839075	SYN2;SYN2	3.34E-05	0.481794883
cg11955385		1.18E-05	0.481790041
cg25761791		3.34E-05	0.481749961
cg14287112	EYA4;EYA4;EYA4	0.000537905	0.481725419
cg00003298	LHX5	4.34E-05	0.481712016
cg14156405	RGS7;RGS7	3.34E-05	0.481632175
cg14243778	CNTN1;CNTN1	1.11E-05	0.481455603
cg09907509	C13orf36;C13orf36	1.11E-05	0.481417658
cg18324798	DNAH9	1.96E-05	0.481396643
cg06274159	ZFP42	0.000117986	0.481379705
cg19320476	FAM181B	3.34E-05	0.481371023
cg25122395	DCC	1.11E-05	0.481346807
cg02391906	LPPR1	8.60E-06	0.481336956
cg17573292	SOD3	1.96E-05	0.481334797
cg12019801	ZNF516	1.96E-05	0.481301072
cg24762932		8.60E-06	0.481300642
cg21932814	CSTA	4.34E-05	0.481277225
cg05252264	FCAR;FCAR;FCAR;FCAR;FCAR;FCAR;FCAR;F	7.23E-05	0.481266122
cg17091361	TEKT3	1.18E-05	0.481245862
cg07520506	CD163L1	1.96E-05	0.481234861
cg03014326	ANKRD30B;ANKRD30B	0.001948248	0.481154216
cg15286044	CRTAC1	2.58E-05	0.481067531
cg23708361	CNTNAP2	3.34E-05	0.48102669
cg25859468	MEOX2;MEOX2	1.96E-05	0.480999794
cg00590139	A2BP1;A2BP1;A2BP1	8.60E-06	0.480964603
cg04592811	PIGL	7.23E-05	0.480941964

cg19029325		2.58E-05	0.480923481
cg27319123	KCNA4;KCNA4	1.50E-05	0.480867798
cg11747183		8.60E-06	0.480861719
cg10211058		2.58E-05	0.480859241
cg25432696	CXCL6	1.50E-05	0.48085032
cg20429104	ZNF516	3.34E-05	0.480811927
cg14664412	CSTA;CSTA	8.60E-06	0.480803855
cg11012046	GLB1L2	9.24E-05	0.480743588
cg22158769	LOC375196;LOC100271715	8.60E-06	0.480715389
cg17275781		1.96E-05	0.480669695
cg11286023	CPLX1;CPLX1	9.24E-05	0.480667554
cg03688058	STEAP2;STEAP2	5.59E-05	0.480652161
cg23248150	TLL1	8.60E-06	0.480594252
cg00368636		0.000187194	0.480573591
cg04346283	FAM46A	2.58E-05	0.480538995
cg15540138	TLL1	0.000148457	0.480536111
cg14117138	HIF3A	0.000148457	0.480515864
cg07837260		8.60E-06	0.480479628
cg04098866		1.96E-05	0.480463714
cg27357571	OLIG2	4.34E-05	0.48044886
cg19754554	PDE4B;PDE4B	1.11E-05	0.480445849
cg10792302		7.23E-05	0.480438484
cg04834436	IRX6	1.50E-05	0.48038569
cg16028201	KANK4;KANK4	7.23E-05	0.480377092
cg16050974	RBP7	0.000122184	0.480370671
cg07846220	LAMA1	4.34E-05	0.480357458
cg16580499	SALL3	1.96E-05	0.480333553
cg23286646	SIM2	0.000233422	0.480328392
cg21961487	RFX4	3.34E-05	0.480327596
cg15244223	SLC35F1	8.60E-06	0.48028279
cg12492885	C22orf45;C22orf45;UPB1	8.60E-06	0.480264011
cg25312594	KCNK1	8.60E-06	0.480263942
cg06893138	HIST3H2BB;HIST3H2A	8.60E-06	0.480247946
cg02858606	CDH8	1.11E-05	0.480244795
cg02281208	GABRG3	1.50E-05	0.480241138
cg22249612	CD63;CD63	5.59E-05	0.480234285
cg03882585	SYNE1;SYNE1;SYNE1	1.11E-05	0.480231291
cg08231709	KCNS2	1.96E-05	0.480209117
cg25317664	SLC30A10;SLC30A10	2.58E-05	0.480189297
cg02912326	GPR63;GPR63	9.24E-05	0.480110613
cg02033582		2.58E-05	0.480069066
cg04556126	ZIC4;ZIC4;ZIC4;ZIC4;ZIC4	2.58E-05	0.480062558
cg03478199	GRM1;GRM1;GRM1;GRM1	7.23E-05	0.480052276
cg02311414	FAM84A	3.34E-05	0.480045484
cg03735496	GREB1L	5.59E-05	0.480039869
cg21566689		7.23E-05	0.480038821
cg21357629		4.34E-05	0.479999856
cg27493010		1.50E-05	0.479981169
cg12260653	TMEM20;TMEM20	2.08E-05	0.479976228
cg05307752	ARHGAP15	0.000148457	0.47991245
cg08832906	CLEC2L	0.000290372	0.47988183
cg07543626	NOVA1;NOVA1;NOVA1	0.005897668	0.479843958
cg01060861	MKX	3.34E-05	0.479733202
cg07402669	APCDD1L	1.96E-05	0.479681847
cg21929943		8.60E-06	0.479665838
cg03429643	MGAT4A;MGAT4A	9.24E-05	0.479626661
cg08272731	LHX8	7.23E-05	0.479612733
cg17299899		1.11E-05	0.4796045
cg26630231		2.58E-05	0.479567453
cg13591352	ABCG5	1.96E-05	0.479551217

cg22874858	PDE11A;PDE11A	1.50E-05	0.47951746
cg03450948	HOXD1	8.60E-06	0.479482485
cg08376141	PRRT1	3.34E-05	0.479406005
cg10469329	MFAP3L	0.000117986	0.479399559
cg00735667		1.18E-05	0.479369327
cg24384244	ZNF662;ZNF662;ZNF662	0.000357885	0.479350651
cg26564040	PTGS2	3.21E-05	0.479322068
cg03167496	BDNF;BDNF;BDNF;BDNF	8.60E-06	0.479318072
cg20443778	LRP1B	9.24E-05	0.479303822
cg16675507		3.34E-05	0.479280027
cg19178853	NBLA00301;HAND2	3.34E-05	0.479249248
cg09417809		8.60E-06	0.479247671
cg18471993		1.11E-05	0.479189457
cg13453374		9.24E-05	0.479181403
cg16562959		0.000117986	0.479176769
cg00017489	DPP6	4.34E-05	0.479146575
cg21468929	EMX2OS	1.50E-05	0.479110884
cg21678312	PTPRR	9.24E-05	0.479038937
cg11795854		3.34E-05	0.47903237
cg23697855		8.60E-06	0.479001748
cg07537734	PDGFC	3.34E-05	0.478991132
cg09334277		1.11E-05	0.478982299
cg23564700	EPB41L3	5.59E-05	0.478954047
cg12951849	FAM38B	1.96E-05	0.478953045
cg24456002		4.34E-05	0.478928183
cg08961408	NPNT	3.34E-05	0.478922701
cg03109827	TCERG1L	8.60E-06	0.478921372
cg22637867	ZNF709	1.60E-05	0.478899212
cg16856286	HOXC13	4.34E-05	0.478851852
cg03298319		3.34E-05	0.478842902
cg14428146	NKX2-6	2.58E-05	0.478810189
cg22095604	NOL4;NOL4	1.96E-05	0.478736156
cg05501996		2.58E-05	0.478730105
cg24933645	SLC30A10	1.50E-05	0.478723791
cg10761315	ASPG	5.59E-05	0.478720982
cg16020747	RALYL;RALYL;RALYL	2.58E-05	0.478697235
cg22496809	TBX18	8.60E-06	0.478694912
cg18441661	VGLL2;VGLL2	2.58E-05	0.478657608
cg17861230	PDE4C	1.96E-05	0.478635652
cg06926782	NRG1;NRG1;NRG1;NRG1;NRG1;NRG1;NRG1;	5.59E-05	0.478626014
cg06554120	CTNND2	1.96E-05	0.478584877
cg18764577	LAMA1;LAMA1	8.60E-06	0.478558357
cg09411719		1.96E-05	0.478546765
cg07540103	FBXO39	9.24E-05	0.478538527
cg11499681	DNAH9	8.60E-06	0.478533855
cg16762030		3.34E-05	0.478484681
cg19536810	LRIG3;LRIG3	8.60E-06	0.478482275
cg16118212	EFEMP1;EFEMP1;EFEMP1	9.24E-05	0.478471302
cg17816908	SFRP1	8.60E-06	0.478464108
cg01354961	NXPH1	1.18E-05	0.478424501
cg24909548		1.96E-05	0.478363539
cg16617910	CALCR;CALCR	1.11E-05	0.478343954
cg16170614	SOD3	1.50E-05	0.478338272
cg16764274	SGPP2	2.58E-05	0.478324179
cg13073773	GSC	0.000187194	0.478212689
cg13836627	TJP1;TJP1	3.34E-05	0.478212125
cg27003782	CPXM2;CPXM2	2.58E-05	0.478201586
cg12009023	EDNRA;EDNRA;EDNRA;EDNRA;EDNRA	1.50E-05	0.478195567
cg19717586	NTM;NTM;NTM;NTM;NTM;NTM;NTM	3.34E-05	0.478179052
cg15260721	GULP1	2.58E-05	0.478175657

cg07544187	CILP2	2.58E-05	0.478118845
cg20916066	NKX2-1;NKX2-1	1.50E-05	0.478095172
cg26685735	COL4A3;COL4A4;COL4A3;COL4A4;COL4A3;C(	2.58E-05	0.47806618
cg21493633	TLL1	1.11E-05	0.477999171
cg17973164	BRUNOL4;BRUNOL4;BRUNOL4;BRUNOL4	1.96E-05	0.477989275
cg10333808	ST8SIA1;ST8SIA1	3.34E-05	0.477981193
cg01663018		6.94E-05	0.477964572
cg26542254	MAGI2	8.60E-06	0.477961695
cg08890418	KIF17;KIF17	0.000290372	0.477949886
cg06030535		0.000290372	0.477948575
cg04812475		1.96E-05	0.477936825
cg15562220	SCGN	1.50E-05	0.47793195
cg10690440		3.34E-05	0.477921424
cg12947833	HSPB2;CRYAB;HSPB2	1.50E-05	0.477905695
cg12373617	KCNN2;KCNN2	2.58E-05	0.477891224
cg21193143	PPFIA2	1.11E-05	0.477885962
cg13633270		8.60E-06	0.477880323
cg05127369	NR2F2;NR2F2;NR2F2;NR2F2;MIR1469	8.60E-06	0.477861937
cg12122597	KCNA4	5.11E-05	0.477857052
cg25832771	MSC	2.58E-05	0.477834157
cg00745389		8.60E-06	0.477815012
cg21700332	STEAP2;STEAP2	8.60E-06	0.477764071
cg11630554	TRIM61;C4orf39;C4orf39	8.60E-06	0.477761537
cg07243366		1.11E-05	0.477721972
cg09554951	KCNQ1DN	8.60E-06	0.477675862
cg13062455	SEL1L	2.58E-05	0.477636534
cg08975922		8.60E-06	0.477619124
cg02825373		1.11E-05	0.477581836
cg17509220	CACNA1A;CACNA1A	1.50E-05	0.477579841
cg09405169	PPARG;PPARG;PPARG	7.23E-05	0.477550008
cg16238815		4.34E-05	0.47748281
cg14750948	ZIC1	4.34E-05	0.477471377
cg27583690	NKX6-2	1.11E-05	0.477386509
cg15839219	PCDH20	1.50E-05	0.47736067
cg07553626	LPAR3	2.58E-05	0.477295869
cg12206199	LOC375196;LOC100271715	8.60E-06	0.477279211
cg21034023	UGGT2	3.34E-05	0.477243177
cg15814504		1.50E-05	0.477236213
cg20776240	ADAMTS19	1.60E-05	0.477231105
cg26990587	ACTL6B	8.60E-06	0.477219963
cg10541864	HAND2;NBLA00301;HAND2	8.60E-06	0.477184321
cg22344611	MYOD1	0.000117986	0.477178784
cg13566610	RELN;RELN	1.50E-05	0.477164452
cg24604013	SOX1	5.59E-05	0.477123536
cg25643118	ELOVL4	1.50E-05	0.477117271
cg07541744	IRX6	1.96E-05	0.477091111
cg27088072		8.60E-06	0.477088567
cg14179908	PNPLA3;PNPLA3	7.23E-05	0.477042327
cg09252495		7.23E-05	0.477042088
cg16226866		0.000187194	0.47696973
cg04739647	HOXD9;HOXD9	1.50E-05	0.476903881
cg03783062	C7orf63;C7orf63	0.000233422	0.476874287
cg06719900	C11orf87;C11orf87	2.58E-05	0.476871791
cg03320372	LOC100192378	1.11E-05	0.476856432
cg26282384	PCDHGA4;PCDHGA6;PCDHGA1;PCDHGA5;P(	1.11E-05	0.476822191
cg08393317	CACNG7	1.11E-05	0.47681598
cg08551725	SCRN1;SCRN1;SCRN1;SCRN1	7.23E-05	0.476800251
cg14176930		8.60E-06	0.476754693
cg15621260	FIBIN;FIBIN	8.60E-06	0.476721462
cg23346462	PEX5L	1.50E-05	0.476715558

cg03758150	ZNF536	0.000122184	0.476706456
cg13298841	HECW1	8.60E-06	0.476703625
cg09016242	SALL1;SALL1	0.000357885	0.476676811
cg12040830	NCAM1;NCAM1;NCAM1	0.000117986	0.476654457
cg01894498		1.50E-05	0.476617364
cg12868067	TMEM132C	0.000117986	0.476563574
cg03239178		2.58E-05	0.4765475
cg06085994	PPFIA2	8.60E-06	0.476544347
cg01301382		2.58E-05	0.476530763
cg03685886		3.34E-05	0.476516013
cg02741554	CXCL2	0.000233422	0.476500047
cg13652336	PREX2;PREX2	8.60E-06	0.476482024
cg27513574	SLC34A2;SLC34A2	7.23E-05	0.476431515
cg09557387	CR1L	0.000357885	0.476427837
cg05991454		7.23E-05	0.476415072
cg19423622	PTPRN2;PTPRN2;PTPRN2	1.50E-05	0.476368785
cg25509174	PCOLCE2	9.24E-05	0.47636037
cg19313043	NGF	2.58E-05	0.476341185
cg03547745	SOX9;SOX9	7.23E-05	0.476339525
cg16434657		3.34E-05	0.476337221
cg12687157	MARVELD2;MARVELD2	1.96E-05	0.476324647
cg01181415	LMO3;LMO3	8.60E-06	0.476311307
cg23433370	STK38L	0.000148457	0.476310853
cg09712306	AURKA;AURKA;AURKA;AURKA;AURKA;AURK	0.000537905	0.476304346
cg13482432	FOXB2	2.58E-05	0.476287229
cg20442599		8.60E-06	0.476286185
cg06906472	ST6GAL2;ST6GAL2;ST6GAL2	1.11E-05	0.476284848
cg25617092	GRID2	1.96E-05	0.476284706
cg04304705	FAM19A5	8.60E-06	0.476275495
cg05376374	MIR129-2	4.90E-05	0.476269147
cg06374079		0.000357885	0.476232772
cg08003628	ZFPM2	7.23E-05	0.476225644
cg22702328	CA10;CA10;CA10;CA10	8.60E-06	0.476218869
cg22784954	ADAMTS16	3.34E-05	0.476177204
cg20711639		1.11E-05	0.476136558
cg27436118	MGRN1;MGRN1;MGRN1;MGRN1	3.34E-05	0.476035029
cg26568762		9.24E-05	0.476000766
cg07365816	SGEF	3.34E-05	0.476000409
cg14094983	NXPH1	1.11E-05	0.475990244
cg26091021		3.34E-05	0.475951673
cg19343464	GRIA4;GRIA4;GRIA4;GRIA4;GRIA4	3.34E-05	0.475947986
cg21583850	KCND3;KCND3	0.000148457	0.475936528
cg27591502	DOCK2;FAM196B	2.58E-05	0.475923709
cg05403555	TACR1;TACR1	0.000117986	0.475902504
cg25916711	NKX2-5;NKX2-5;NKX2-5	8.60E-06	0.475876356
cg16049690	BTNL9	4.34E-05	0.475860347
cg14711366	TMED9	0.000117986	0.475834602
cg04585378	CASR	1.11E-05	0.475832872
cg02266771	PRSS16	1.50E-05	0.475816524
cg01799671	CMIP;CMIP	4.34E-05	0.475812457
cg10088985	CXCL5;CXCL5	1.11E-05	0.475809021
cg16355785	GABRA1;GABRA1;GABRA1;GABRA1;GABRA1	1.50E-05	0.475776446
cg00516513		3.34E-05	0.475759472
cg08448589		8.60E-06	0.47560705
cg24144083		3.34E-05	0.475595912
cg15603568	GRIA4;GRIA4;GRIA4;GRIA4	7.23E-05	0.475590665
cg25287257	MNX1;MNX1	2.58E-05	0.475547644
cg11068946	NKX6-2	8.60E-06	0.475530135
cg13721404	NOL4	4.34E-05	0.475522059
cg04652961	GJA1	1.96E-05	0.475521904

cg21534423		1.96E-05	0.475471423
cg18009496	IRX4	3.34E-05	0.47546532
cg15638709		2.58E-05	0.475451059
cg05446629	B3GAT1;B3GAT1;B3GAT1;B3GAT1	0.000334462	0.475365912
cg03792162		1.50E-05	0.475324534
cg02436788	TEKT3	1.18E-05	0.475283994
cg16924337	RGS17	5.59E-05	0.475262534
cg13239420		1.96E-05	0.475256477
cg02662277	PHYHIPL;PHYHIPL;PHYHIPL	1.96E-05	0.475235337
cg24081819	EPHX2	1.50E-05	0.47522237
cg07797397	HOXD1	1.50E-05	0.475205048
cg14103680		5.59E-05	0.475201454
cg07005778		1.11E-05	0.475161476
cg21133153		8.60E-06	0.475143338
cg03753331	DPP10;DPP10	7.23E-05	0.475139795
cg16524049	LMX1A	1.96E-05	0.47512698
cg04723401		8.60E-06	0.47512416
cg20675505	MSC	8.60E-06	0.475112853
cg25912827	DPP4;DPP4	0.000357885	0.475074425
cg14338425	MAL2	1.50E-05	0.475042237
cg24114014		4.34E-05	0.475034871
cg06896909	TMEM196	8.60E-06	0.475029388
cg14506196	NKX2-3	4.34E-05	0.475003173
cg15679813	PHF21B;PHF21B	4.34E-05	0.474994833
cg00910695	RSPO2;RSPO2	2.58E-05	0.474989647
cg21800232	ANKRD34B	1.50E-05	0.474952656
cg06994420	MEIS1	0.000117986	0.47491685
cg16196211		8.60E-06	0.474910276
cg14454680	C3orf26;FILIP1L;FILIP1L	0.000233422	0.474866939
cg02866106	CHRM2;CHRM2;CHRM2;CHRM2;CHRM2;CHR	8.60E-06	0.474829656
cg13125157	CA10;CA10;CA10	2.58E-05	0.474793661
cg01030121	LOC283392;TRHDE;LOC283392	8.60E-06	0.474786748
cg26151467	SOX1;SOX1	5.59E-05	0.474783778
cg02630214		8.60E-06	0.474763635
cg10146199	GLRB;GLRB;GLRB	1.50E-05	0.474743528
cg17110586		8.60E-06	0.474739929
cg23948240		8.60E-06	0.474721066
cg16521917	CNTNAP2;CNTNAP2	2.58E-05	0.474709767
cg14393469	STEAP2;STEAP2	3.34E-05	0.474706728
cg25482900	FBLL1	3.34E-05	0.474685051
cg13396713	VPS53;VPS53	4.34E-05	0.474595166
cg06226567	C20orf56	0.000290372	0.47454455
cg24198558	TMEM108;TMEM108	4.34E-05	0.474535067
cg11907729	HMGCLL1;HMGCLL1	2.58E-05	0.474505561
cg05380019		1.11E-05	0.474488289
cg26065057	GALR1	7.23E-05	0.474483779
cg23331421	ZNF536	1.96E-05	0.474470144
cg18465694		8.60E-06	0.474455534
cg01546568	CTNNA2;CTNNA2;CTNNA2;CTNNA2	1.18E-05	0.474449972
cg03589296	MEIS1	8.60E-06	0.474387139
cg06966811	OTX2	2.58E-05	0.474344147
cg07095997	SIX4	1.60E-05	0.474325389
cg22639011	KCNA4	8.60E-06	0.474307543
cg23380558	GPR123	0.000148457	0.474283816
cg17222500	SLC6A15;SLC6A15;SLC6A15	1.50E-05	0.474275793
cg04324308	COL4A3;COL4A4;COL4A3;COL4A4;COL4A3;C(	3.34E-05	0.474255582
cg12821278	DBC1	1.96E-05	0.474227369
cg03450844	GIMAP1	1.96E-05	0.474198691
cg19632594	FBXL7	8.60E-06	0.474188055
cg23514324	PPARG;PPARG;PPARG	4.34E-05	0.474187902

cg03287574		8.60E-06	0.474185163
cg01236063		8.60E-06	0.474175841
cg16655338	RIMS1	1.50E-05	0.474167949
cg01851378		8.60E-06	0.474142501
cg24886267	FBLL1	1.18E-05	0.474130831
cg05381746	MKX	1.11E-05	0.474125901
cg21699044		1.96E-05	0.474107299
cg06497848	VAX1;VAX1	1.50E-05	0.474096409
cg22216413	ANO3	1.96E-05	0.47409396
cg23169957	MACROD2	4.34E-05	0.474082935
cg26870460	ZNF215;ZNF215	3.34E-05	0.474048046
cg07295678	DPYSL4	1.50E-05	0.473928209
cg06580551	ZNF662;ZNF662;ZNF662	2.58E-05	0.47391804
cg22235018	TFAP2D	1.11E-05	0.47391489
cg00904966	LOC146336	0.000791389	0.473834803
cg07135032	LMX1A	5.59E-05	0.47378704
cg16398329	FGF14	0.000955793	0.473730808
cg17039236		3.34E-05	0.473711547
cg17380661	SIM1	1.96E-05	0.473691565
cg02004851	PCDHA2;PCDHA1;PCDHA1;PCDHA6;PCDHA5	0.000148457	0.473677774
cg05224741		4.34E-05	0.473659749
cg10755058	ENTPD3;ENTPD3	1.96E-05	0.47363379
cg23495279	CSMD1	8.60E-06	0.473616565
cg12636169	ESRRG	8.60E-06	0.473577023
cg26709285	KCNB1;KCNB1	8.60E-06	0.473489818
cg16563171	PAX3;PAX3;PAX3;PAX3;PAX3;PAX3	8.60E-06	0.473425244
cg01035689		8.60E-06	0.473412607
cg26824006		1.11E-05	0.473410262
cg11563680		3.34E-05	0.473397055
cg04832466		3.34E-05	0.473396075
cg20847110	LOC401127	1.50E-05	0.473356128
cg15680624	CRYBA2;CRYBA2;CRYBA2	0.000117986	0.47333341
cg14752336	SORCS3	8.60E-06	0.473283904
cg06122871		1.50E-05	0.473259296
cg04993279		1.96E-05	0.47325504
cg23665824	CTNNA2;LRRTM1;CTNNA2	1.11E-05	0.473233682
cg07789132		8.60E-06	0.473198678
cg19988449	BNC1	8.60E-06	0.473147425
cg18267381	ZNF385D;ZNF385D	8.60E-06	0.473143014
cg20019019	GPR85;GPR85;GPR85;GPR85	8.60E-06	0.473116891
cg05917460	PAX9;PAX9	7.23E-05	0.473086603
cg01969701		0.000187194	0.473013043
cg02835467	CDH7;CDH7	8.60E-06	0.473006871
cg17958423	ZNF215	8.60E-06	0.472974137
cg17988460	NKX6-2	8.60E-06	0.472968793
cg27638126	MEOX2	2.80E-05	0.472954402
cg17780246	CACNG8	1.11E-05	0.472887295
cg04330371	NR2F2;NR2F2;NR2F2;MIR1469;NR2F2	1.60E-05	0.472885735
cg21739754	ZNF804A	8.60E-06	0.47287061
cg01844178	CCDC39	9.24E-05	0.472863438
cg26078977	PDE4D;PDE4D;PDE4D	8.60E-06	0.472853356
cg11686427	PCDHGA4;PCDHGA6;PCDHGA1;PCDHGA8;PC	3.34E-05	0.472767749
cg15700437	CRCT1	5.59E-05	0.472764573
cg14600824		8.60E-06	0.472724165
cg09260089	NKX6-2	8.60E-06	0.472679996
cg12682684	EPHA5;EPHA5	1.50E-05	0.472675982
cg26557404	MYO5B	0.000148457	0.472628382
cg09527126		1.96E-05	0.472627914
cg13356896	BOLL;BOLL	1.11E-05	0.472581356
cg15581269		8.60E-06	0.472578905

cg05627639	CHST8;CHST8	4.34E-05	0.472568764
cg19485202	COMP;COMP	1.96E-05	0.472559658
cg18771173	SLC5A7	1.96E-05	0.472556797
cg16848524	KIAA1688	1.50E-05	0.47253313
cg24997896	LOC100128811;GPR158	8.60E-06	0.472518535
cg02312409	STL	0.000791389	0.472510993
cg04825327	C4orf31	7.23E-05	0.472493636
cg26931862	HOXC12	1.50E-05	0.472439334
cg00708105		8.60E-06	0.472429682
cg22079161	AFAP1;AFAP1	0.00043997	0.472429436
cg00563824		1.96E-05	0.472428643
cg09795809	MGC45800	5.59E-05	0.472410764
cg04352272		1.96E-05	0.472384743
cg03048654	VAX1;VAX1	9.24E-05	0.472363606
cg14305313		0.000290372	0.472360204
cg01815529	PCSK2	0.000148457	0.472351869
cg14583871	CLIC6	1.11E-05	0.472312585
cg10434152	GOLSYN;GOLSYN	4.34E-05	0.472292109
cg23243267		3.34E-05	0.472290549
cg18722847	ZNF502;ZNF502;ZNF502;ZNF502	3.34E-05	0.472270675
cg14874351	LIN7A	0.000435122	0.472259734
cg14051544	SLC7A14	5.11E-05	0.472233834
cg06246357		8.60E-06	0.472202958
cg25928603	SATB2	0.000357885	0.472166953
cg01583131	BDNF	4.34E-05	0.472158687
cg16728539	CACNA1C;CACNA1C;CACNA1C;CACNA1C;CA	7.23E-05	0.472157699
cg02090656	SGEF	1.50E-05	0.472122952
cg07451261		0.000290372	0.472122483
cg16652741	CDK5R2	9.24E-05	0.472075569
cg15085899	NCOR2;NCOR2	0.001376172	0.472052636
cg03065447		9.04E-05	0.47200705
cg05601456	ARSJ;ARSJ	9.24E-05	0.472006802
cg05021643	HOXD3	1.96E-05	0.471949956
cg14002960	NOL4	2.58E-05	0.471948056
cg20985755	THRB;THRB;THRB	0.000357885	0.471940115
cg07432626		1.96E-05	0.471930834
cg05398903		1.96E-05	0.471922001
cg21389743	CLDN11	8.60E-06	0.471874202
cg08893692		9.24E-05	0.471834139
cg16948320	HRASLS;MGC2889	3.34E-05	0.471831663
cg17482033	ZIC1	2.58E-05	0.471829099
cg20170533	DLEU7;DLEU7	0.000148457	0.471820178
cg06574716	ZNF283	1.96E-05	0.471815305
cg12070159	RGS20	4.34E-05	0.471774901
cg15563027	APBB2;APBB2;APBB2	3.34E-05	0.471750378
cg15873149		1.96E-05	0.471722311
cg12067687	HOOK1	8.60E-06	0.471688428
cg05251676	EGFLAM	0.000233422	0.471682671
cg10233825	LOC100190940	0.000117986	0.47166014
cg17101296		1.11E-05	0.47163855
cg12891160	C1QL2	8.60E-06	0.471596097
cg01939453		3.34E-05	0.471587399
cg18279094	FOXD3	0.000187194	0.471562116
cg08084616		8.60E-06	0.471501398
cg13834623	SCGN	8.60E-06	0.471492483
cg27361134	RIMS4	2.58E-05	0.471483593
cg12961607	SFRS7	0.000187194	0.471454737
cg08229018	PRSS12	2.58E-05	0.471452507
cg04120686		0.000357885	0.471387814
cg23778149	LOC401463	2.58E-05	0.471273224

cg16503924	CDH18;CDH18	1.50E-05	0.471273131
cg14231297	ZSCAN18;ZSCAN18	4.34E-05	0.471241766
cg15987885	NMNAT2	2.58E-05	0.471232554
cg27154343	KCNC2;KCNC2;KCNC2;KCNC2;KCNC2;KCNC2	3.34E-05	0.471221105
cg14414154	APP;APP;APP;APP;APP	3.34E-05	0.471207142
cg12812583		7.23E-05	0.471150281
cg17285225	OXTR;OXTR	3.34E-05	0.471141245
cg11743349	KCNIP4	8.60E-06	0.471113918
cg04395954		4.34E-05	0.471082489
cg17093995	VWC2	5.11E-05	0.471050211
cg23300368	PREX2;PREX2	1.96E-05	0.471041274
cg19853833	BRUNOL4;BRUNOL4;BRUNOL4;BRUNOL4	1.11E-05	0.471007597
cg11389172	SLC18A3;CHAT	2.58E-05	0.471007162
cg01642653	BDNF;BDNF;BDNF;BDNF;BDNF	8.60E-06	0.470983002
cg22548438		8.60E-06	0.470982437
cg04890851		1.96E-05	0.470974785
cg01492330	KRT3	7.23E-05	0.470923222
cg27547954		0.000233422	0.470913281
cg16270399	LOC284276	3.34E-05	0.470864922
cg27486637	WDR17;WDR17;WDR17;WDR17	3.34E-05	0.470852107
cg20525183	PHF21B;PHF21B;PHF21B	4.34E-05	0.470849968
cg14520947		1.96E-05	0.470830217
cg13895765		8.60E-06	0.470791443
cg26348348	LYPD1;LYPD1;LYPD1	9.24E-05	0.470785341
cg13519035		1.96E-05	0.470784878
cg12807794	TCF15	8.60E-06	0.470756205
cg23180938	CDO1	0.000148457	0.4707473
cg20194314		8.60E-06	0.470716519
cg02812207	PRSS12	8.60E-06	0.470653929
cg17054060		0.000117986	0.470647852
cg16235861	TMEM20;TMEM20	1.50E-05	0.470619595
cg17807172		9.24E-05	0.470618169
cg00343633	GRIA4;GRIA4;GRIA4;GRIA4	0.000117986	0.470565757
cg26464221	PRR16	0.000117986	0.470533578
cg20939084	ZIC4;ZIC4;ZIC4;ZIC4;ZIC4	8.60E-06	0.470528819
cg03135351	CLIP4	5.59E-05	0.470491402
cg14001664		1.11E-05	0.470490659
cg08875948	GRM1;GRM1	2.58E-05	0.470484142
cg14390758	LOC100128811;GPR158	8.60E-06	0.470480017
cg09774787	SLC6A2	0.000117986	0.47047969
cg22518696	LPHN2	0.000148457	0.470475139
cg05968145		1.96E-05	0.470472437
cg11357013	NEO1	2.58E-05	0.470412517
cg15900248	HOOK1	2.58E-05	0.470287804
cg05888181	KBTBD11	0.000148457	0.470282001
cg18096962		1.11E-05	0.470272977
cg24530250	PHOX2A	7.23E-05	0.470269486
cg01537995	FLRT2	8.60E-06	0.470258621
cg24611608	EYA1	8.60E-06	0.470257206
cg16377609		1.11E-05	0.470249196
cg04875128	OTUD7A	1.96E-05	0.470183321
cg07629355	IGSF9B	1.11E-05	0.470178346
cg01802258	SLC24A4;SLC24A4;SLC24A4	9.24E-05	0.470166312
cg13321967		8.60E-06	0.470163516
cg03205103	EGFLAM	2.58E-05	0.470154322
cg06849504		1.50E-05	0.470096345
cg25278941		0.000117986	0.470064868
cg01995480	PHF21B;PHF21B	1.11E-05	0.470048242
cg22600043	RSPO2	2.58E-05	0.470029466
cg23032674	COL9A1;COL9A1	1.50E-05	0.470026722

cg14583675	ABCG5	5.59E-05	0.470024991
cg08382774	NKX6-2	1.50E-05	0.469953373
cg23982858	USP44;USP44	1.11E-05	0.469943724
cg17916490	NTSR2	5.59E-05	0.469940945
cg00212988	GPR120	9.24E-05	0.469897882
cg03679755		3.34E-05	0.469897418
cg24683222		4.34E-05	0.469869502
cg25739043		1.50E-05	0.469868526
cg17585615	C21orf29	7.23E-05	0.469859864
cg07162198	SLC2A10	3.34E-05	0.469780119
cg05165940		8.60E-06	0.469775208
cg06610484	MCHR2;MCHR2;MCHR2	1.50E-05	0.469695504
cg04894619	PEX5L	8.60E-06	0.46962234
cg26777729	SGEF	3.34E-05	0.469527554
cg15720995	TMTC1	8.60E-06	0.469525366
cg12363722	MTNR1A	8.60E-06	0.469506364
cg23001131		4.34E-05	0.469495739
cg11893763	GPR26	8.60E-06	0.469463437
cg25861340	SLC9A3	7.23E-05	0.469456057
cg26565021	CHST8;CHST8	4.34E-05	0.469434318
cg12816961	NKX6-2	1.11E-05	0.469399362
cg17326555		4.34E-05	0.469374111
cg03740167		8.60E-06	0.469318541
cg09788416		9.24E-05	0.46931528
cg25260137		2.58E-05	0.469237614
cg18789663	PLD5	4.34E-05	0.469167025
cg12936121		8.60E-06	0.46915208
cg25837803	PTGS2	3.34E-05	0.469117087
cg00824141	FHL2;FHL2;FHL2;FHL2;FHL2;FHL2;FHL2	0.000148457	0.4691151
cg09068528	ACADL;ACADL	1.50E-05	0.469092271
cg26861946	CDH6;CDH6	3.34E-05	0.469084321
cg13545212	SLC13A5;SLC13A5	5.59E-05	0.468961611
cg06850283	FGF10	5.59E-05	0.468960978
cg10386483	GJD2	2.58E-05	0.468949655
cg14146100	PCDH10;PCDH10	1.11E-05	0.4688234
cg10784386	THBS4	2.58E-05	0.468782747
cg26705953	PLA2G7;PLA2G7;PLA2G7	1.96E-05	0.468770204
cg06746118		4.34E-05	0.468768869
cg18161374	BRUNOL4;BRUNOL4;BRUNOL4;BRUNOL4	8.60E-06	0.468755021
cg15843567	MYO3A;MYO3A	7.23E-05	0.468748021
cg23632875	TCERG1L	2.58E-05	0.468717975
cg24496223		3.34E-05	0.468704282
cg01832737	PDE10A;PDE10A	2.58E-05	0.468691958
cg10838091	EPHA8;EPHA8	8.60E-06	0.468683809
cg07472373	DRGX	5.59E-05	0.468660363
cg11439592	SASH1	8.60E-06	0.468658174
cg14516183	SEC22A	4.34E-05	0.46865133
cg13437581		1.50E-05	0.468640318
cg23833452	RAB32	9.24E-05	0.468568818
cg00233079	SGMS2	2.58E-05	0.46856601
cg12294121	GABRB1;GABRB1	1.11E-05	0.468562542
cg11332236	RHCG	3.34E-05	0.468549721
cg02925601		2.58E-05	0.468515297
cg06507579		7.23E-05	0.468511596
cg23771603	MYO3A	7.23E-05	0.468502607
cg15511738	CELSR3	3.34E-05	0.46848993
cg21308575	EMR1	0.000187194	0.468461653
cg07893584	PTPRD;PTPRD;PTPRD	3.34E-05	0.468453574
cg16426537	PTPN5;PTPN5;PTPN5	1.50E-05	0.468392324
cg18953784		1.96E-05	0.468340885

cg02983950		0.000148457	0.468310828
cg12917718	KLHL1;KLHL1;ATXN8OS	8.60E-06	0.468284161
cg18438777	NPY5R	8.60E-06	0.468255225
cg06160853		0.000357885	0.468227167
cg17001566	PRDM16;PRDM16	1.96E-05	0.468221771
cg18150439	NXPH2	1.11E-05	0.468189751
cg22857085		8.60E-06	0.468165833
cg11931596	IL7	8.60E-06	0.468121492
cg03530754	TMEM132C	4.34E-05	0.468101803
cg11400953	HPSE2;HPSE2;HPSE2;HPSE2	0.000187194	0.468094788
cg01168865	SLC25A21;SLC25A21;LOC100129794	1.50E-05	0.46807318
cg03365733	PRDM13	8.60E-06	0.468060935
cg03430846	NRG1	8.60E-06	0.468043676
cg11229543	PRDM16;PRDM16;FLJ42875;FLJ42875	3.34E-05	0.467999986
cg12769506		0.00045851	0.467972367
cg01625087		1.50E-05	0.467959411
cg27131891		0.000148457	0.467932375
cg22264930	TEKT3	8.60E-06	0.467928398
cg03354340	MARVELD3;MARVELD3	1.96E-05	0.467918391
cg12628659	ANKRD34C	1.96E-05	0.467912744
cg16318053	ISL1;ISL1	2.58E-05	0.467899172
cg06671842	PTPN5;PTPN5;PTPN5	7.23E-05	0.467893016
cg10512745	DMRTA2	0.000117986	0.467876603
cg07489048	FOXG1	1.50E-05	0.467800398
cg10387551	KCNA4	7.23E-05	0.467793825
cg26467069	RND3	4.34E-05	0.467760167
cg20453394		2.58E-05	0.467679526
cg14997413	COBL	1.50E-05	0.467660042
cg13897348	MIB2;MIB2;MIB2;MIB2;MIB2	0.000233422	0.467659384
cg01615704	MALL;MALL	3.34E-05	0.467636869
cg12379383		8.60E-06	0.467609311
cg25633678		0.000148457	0.467581813
cg10303487	DPYS	1.11E-05	0.467567756
cg13929328	FOXI2	1.50E-05	0.467535451
cg27210464		1.96E-05	0.467515922
cg04674315		1.96E-05	0.467515267
cg17534009		1.11E-05	0.467504636
cg19389293	C18orf1;C18orf1;C18orf1;C18orf1;C18orf1;C18o	1.96E-05	0.467442954
cg08055910	FAM149A	0.0050758	0.467431939
cg12946880	MAGI1;MAGI1;MAGI1	3.34E-05	0.467427358
cg03650674	GRHL2	7.23E-05	0.467424391
cg27360326	PCDH8;PCDH8;PCDH8;PCDH8	0.000117986	0.467416973
cg17052813	GPR37	1.50E-05	0.467412501
cg19230755		1.50E-05	0.467288359
cg11254053	AK2;AK2	1.11E-05	0.467239379
cg15179725	BRUNOL4;BRUNOL4;BRUNOL4;BRUNOL4	3.34E-05	0.467238362
cg01382864	PTPRN	1.11E-05	0.467235067
cg18044663	GFRA1;GFRA1;GFRA1	0.001147425	0.467220005
cg01023672		0.000117986	0.467209709
cg27443071		7.23E-05	0.467166401
cg25684999	PTF1A	8.60E-06	0.467156752
cg05539745	HCN1	1.11E-05	0.467118971
cg20193324	SHISA2	0.000357885	0.467110768
cg06510438	FAM38B	1.96E-05	0.467098059
cg13437164	EPHA8;EPHA8;EPHA8;EPHA8	3.34E-05	0.467061799
cg14530834		3.34E-05	0.467047202
cg26084529	LRAT	3.34E-05	0.467045485
cg26131286		8.60E-06	0.467021258
cg13509195	CPNE8	0.001376172	0.467016029
cg08460732		2.58E-05	0.467012358

cg12453014	PRR16	0.000187194	0.466994734
cg18588768	FAM155A	1.11E-05	0.466987103
cg03967627	C8orf73	3.34E-05	0.466957059
cg23242697		0.000117986	0.466940453
cg24495585		3.34E-05	0.466939529
cg26170805		1.50E-05	0.466936122
cg04830357	PCDHGA1;PCDHGA2;PCDHGA2	2.58E-05	0.466935336
cg14134497	DTNA;DTNA;DTNA	1.96E-05	0.466915112
cg08370996	NR2F2;NR2F2	1.11E-05	0.466910741
cg17078116	NEFM;NEFM	7.23E-05	0.466891355
cg23713724	KCNIP4;KCNIP4;KCNIP4;KCNIP4	9.24E-05	0.46684384
cg10074727	GCM2	4.34E-05	0.466797144
cg12475759	AJAP1;AJAP1	4.34E-05	0.466796145
cg26272220	ACSS3;ACSS3	1.50E-05	0.466760619
cg12693702	MIR148A	0.000148457	0.466743607
cg04597433	DRD5	9.24E-05	0.466740913
cg23030863	JAM3	4.34E-05	0.466729111
cg21714266	GRM1;GRM1	7.23E-05	0.466692971
cg03573068	SLC6A5	2.58E-05	0.466673926
cg06410537	TLX3	8.60E-06	0.466654191
cg03152033		3.21E-05	0.466652044
cg11357746	TBX5;TBX5;TBX5;TBX5	0.000117986	0.466643821
cg00962913	FAM163A	1.96E-05	0.466621037
cg05230054	CHST8;CHST8;CHST8	3.34E-05	0.466614582
cg13872688	ROR1;ROR1	0.000205664	0.466600946
cg19908577	UGT8	1.96E-05	0.466594506
cg12513880		1.96E-05	0.466575923
cg13983319	KIF12	0.000187194	0.466575495
cg06480695		1.96E-05	0.466575175
cg04415798	PAX9	9.24E-05	0.466549578
cg18856652	PLEKHH2	1.50E-05	0.466500402
cg23516463		2.58E-05	0.466471197
cg22388982	COL4A3;COL4A3;COL4A3;COL4A4;COL4A3	4.34E-05	0.466411341
cg21232620	HTR1B	1.50E-05	0.466362906
cg01519253		1.96E-05	0.466327111
cg16549389	SCNN1B	4.34E-05	0.466248437
cg23447233	MAEA;MAEA	7.23E-05	0.466178357
cg04304036	SLC7A6;SLC7A6;SLC7A6OS	1.96E-05	0.466174412
cg13023870		0.000233422	0.466152675
cg12993715		1.96E-05	0.466072398
cg04671611	A2BP1;A2BP1	1.50E-05	0.466063582
cg02285791		1.18E-05	0.466058783
cg06704122	EN1	9.24E-05	0.466041236
cg25465938	CACNA1E	8.60E-06	0.466026064
cg05080154	SALL3	8.60E-06	0.466000767
cg11189837	ADAMTS1	9.24E-05	0.465967846
cg24637364	PDE4B;PDE4B	1.96E-05	0.465876073
cg26953727		4.34E-05	0.465849941
cg17186168	NTSR2	5.59E-05	0.465848111
cg25027798	PCDHA7;PCDHA13;PCDHA12;PCDHA6;PCDH/	0.000148457	0.465827425
cg14621053	ADAM12;ADAM12	1.11E-05	0.465825119
cg22601348		8.60E-06	0.465822657
cg16166796	PTPRN	3.21E-05	0.465810395
cg13641185		1.96E-05	0.465772797
cg21486465	STEAP2;STEAP2;STEAP2	0.000117986	0.465765086
cg05492839	ELOVL4	1.50E-05	0.465701631
cg07028533	CNTNAP2	3.34E-05	0.465698594
cg13570101	NKX2-5;NKX2-5;NKX2-5	1.11E-05	0.465694932
cg04910970	PRDM13	1.18E-05	0.465646778
cg26947450	LRIG3;LRIG3	7.23E-05	0.465639992

cg14369648	PRSS3	8.60E-06	0.465638197
cg12045337	SP5	5.59E-05	0.465581481
cg25383699	COL21A1	1.50E-05	0.465540831
cg22227354	RAD21L1	0.000122184	0.465506882
cg07627556		2.58E-05	0.465498866
cg07664990		0.000122184	0.465494577
cg24106636	NHLH2;NHLH2	0.000117986	0.465493149
cg17045801	HECW1	0.000233422	0.465491932
cg16409650	HS6ST3	8.60E-06	0.465466001
cg00851389		5.59E-05	0.465448099
cg04645150		7.23E-05	0.465443825
cg06571075	MACROD2	9.24E-05	0.46544316
cg02121529		3.34E-05	0.465438229
cg24205914	RHOBTB1;RHOBTB1	4.34E-05	0.465415219
cg21243919	PDE11A;PDE11A	3.34E-05	0.465393159
cg12595013	ZIC1	8.60E-06	0.46538727
cg26549701		1.96E-05	0.465333539
cg21662160	FREM2	1.11E-05	0.465332618
cg12598635	CPLX1	4.34E-05	0.465318879
cg23889440	MAPK4	0.000187194	0.465310491
cg18131689	ANO5;ANO5	1.11E-05	0.465276954
cg03790988	DOK6	8.60E-06	0.465256713
cg03962691	NGF	1.96E-05	0.465238924
cg08871964	SPTBN4	4.34E-05	0.465215598
cg07028914	TBX18	8.60E-06	0.465188638
cg11341610	CALR	5.59E-05	0.465161648
cg06496484	EVX2	1.96E-05	0.465151043
cg15089487	LRRC49;THAP10	8.60E-06	0.465149997
cg25898550		3.34E-05	0.46514785
cg16788322	PPAPDC1A	1.50E-05	0.465147562
cg15506477	LYPD1;LYPD1	5.59E-05	0.465144471
cg14805347	ALDH1A2;ALDH1A2;ALDH1A2;ALDH1A2	1.96E-05	0.465143219
cg10348234		8.60E-06	0.465130225
cg15048660	FGF12;FGF12	5.59E-05	0.465112615
cg06139908	VSTM2A	3.34E-05	0.465106929
cg07681938	CDH8	1.11E-05	0.465057823
cg19555331	POU4F2	8.60E-06	0.465042738
cg00480389	CBLN4	8.60E-06	0.465034564
cg04319611	FNDC3B;FNDC3B	3.34E-05	0.465026241
cg06094615	C10orf53;C10orf53	1.11E-05	0.464881069
cg03182958	SGPP2	2.08E-05	0.464873001
cg02992632	FGF12;FGF12	8.60E-06	0.464844808
cg16846653		9.24E-05	0.464844142
cg13546414		9.24E-05	0.464820562
cg16283362		0.000117986	0.464811402
cg14604568	PDE1C	0.000233422	0.464805807
cg17546247	ZIC4;ZIC1	8.60E-06	0.464779969
cg23665778	DOCK1	0.000290372	0.464772417
cg01961447	GALR3	8.60E-06	0.464752206
cg14794428	ASCL1	5.59E-05	0.464746226
cg05967403	GSX1	4.90E-05	0.464745362
cg11938014	SH3RF2;SH3RF2	2.58E-05	0.464712332
cg02383130	AMPH;AMPH;AMPH;AMPH	0.000122184	0.464703248
cg27528660	TRPA1	1.11E-05	0.464687672
cg13063344	GALNT9	1.96E-05	0.464683424
cg23934404		0.000122184	0.464680109
cg19760241	LHX1	1.96E-05	0.464629748
cg19509393	HOXD4	2.58E-05	0.464604464
cg10378348		0.000117986	0.464581585
cg22617773		4.34E-05	0.464556911

cg10879116	LPAR3	8.60E-06	0.464555003
cg02722596	PCDHGA4;PCDHGA7;PCDHGA6;PCDHGA1;PC	1.11E-05	0.464537512
cg03983645	DBX1	5.59E-05	0.46453738
cg08957652	IHH	9.24E-05	0.464517584
cg05883874	ZNF214;NLRP14	9.24E-05	0.464517502
cg13670911	SGEF	2.58E-05	0.464504931
cg23683588	PAX9	0.000233422	0.464492623
cg17935297	CILP2	8.60E-06	0.464481029
cg02119363	PEX5L	1.96E-05	0.464431742
cg06481168	C4orf39;TRIM61	0.000187194	0.464414416
cg00162231	SLC6A1	1.50E-05	0.464402903
cg02826439		3.34E-05	0.464401713
cg07095995	COL25A1;COL25A1	8.60E-06	0.464388402
cg11557901		2.58E-05	0.464369457
cg26366091	CHI3L2;CHI3L2	0.000117986	0.464365815
cg01725586	FAM123C;FAM123C;FAM123C;FAM123C	9.24E-05	0.464365679
cg02898293	VSX1;VSX1	8.60E-06	0.464326481
cg01916333	SNCAIP	2.58E-05	0.464311788
cg24141863	WDR17;WDR17	0.000117986	0.464309474
cg04178787		2.58E-05	0.464300068
cg05205953	CXCL2	0.000365373	0.464291431
cg08561856	LYPD5	0.000290372	0.464286823
cg16254309	CNTNAP2	1.50E-05	0.464281796
cg01419567	ZFHX4;LOC100192378	1.96E-05	0.464219662
cg05237641	ADAM12;ADAM12	1.11E-05	0.464210956
cg24543696	DKK3;DKK3;DKK3	5.59E-05	0.464172006
cg16999370	QRFPR	5.59E-05	0.464171673
cg06360427	GALR1	8.60E-06	0.464150804
cg07173635		1.50E-05	0.464116663
cg13314145	NPTX2	5.59E-05	0.464114572
cg14965220	MKX	5.59E-05	0.464103627
cg24871540	MYH14;MYH14;MYH14	1.96E-05	0.464103556
cg13177747	RSPO2	8.60E-06	0.464049341
cg12466737	BRUNOL4;BRUNOL4;BRUNOL4;BRUNOL4	6.94E-05	0.46398792
cg13033090	BRUNOL4;BRUNOL4;BRUNOL4;BRUNOL4	8.60E-06	0.463966724
cg24154839	GABRA4	3.21E-05	0.463943921
cg15529692	BMP7	8.60E-06	0.463908582
cg25444386	KDR	0.000334462	0.463868833
cg07449069	CACNA2D3	0.000187194	0.46380968
cg24767148	PTF1A	1.50E-05	0.463788698
cg23842170	EPHA5;EPHA5	1.11E-05	0.463788315
cg01924292	ADIPOR2	5.59E-05	0.463626423
cg02512920		8.60E-06	0.463600109
cg14676407	CPXM2;CPXM2	2.58E-05	0.46354627
cg09643136		8.60E-06	0.463523465
cg00003994	MEOX2	8.60E-06	0.463507803
cg00929798	CALY	9.24E-05	0.463504634
cg26184474	KIF2A;KIF2A	1.96E-05	0.46348943
cg23683254		8.60E-06	0.463407401
cg12468774	CCDC36;CCDC36	1.50E-05	0.463405176
cg19248676		8.60E-06	0.463390422
cg23173307	CPLX1	1.96E-05	0.463358725
cg10370599	HS3ST2	8.60E-06	0.463356997
cg06187770		1.96E-05	0.463324462
cg07090701	C1QL2	1.50E-05	0.463267485
cg08812889		1.11E-05	0.463267399
cg00258409	CPE	1.11E-05	0.463246438
cg23536473	FERD3L	7.23E-05	0.463233568
cg17078253		1.96E-05	0.463213818
cg10250663	IRX6	2.58E-05	0.463175274

cg18778433	PARVA;PARVA	1.50E-05	0.463173257
cg00191178		4.34E-05	0.463156671
cg12965599	ZIC1	3.34E-05	0.463095681
cg00974941	C7orf57	5.59E-05	0.463087198
cg10819238	SBNO2	2.58E-05	0.463039517
cg17977409	SH3GL2;SH3GL2	3.34E-05	0.462988471
cg12628145	SLC9A3	7.23E-05	0.46298501
cg06165395	GRIK3	1.96E-05	0.462958659
cg00970313	PAX9	1.96E-05	0.462906073
cg26063563	KCNN2	0.000187194	0.462885525
cg24376776		2.58E-05	0.462824556
cg09655666		2.58E-05	0.462778375
cg15854847	NKX2-6	3.34E-05	0.462725541
cg10288541		2.02E-05	0.462710926
cg05253577	WWC1;WWC1;WWC1	0.000148457	0.462654969
cg14412322	MAGI1;MAGI1;MAGI1	1.96E-05	0.462638426
cg03219968	ESRRG	9.24E-05	0.462601111
cg27031435		2.58E-05	0.462593607
cg16433156	SALL3	8.60E-06	0.462546714
cg18250028	CARTPT	4.34E-05	0.46251208
cg08631504		3.34E-05	0.462502778
cg21117734		3.34E-05	0.462495525
cg27205687	GUCY1A2;GUCY1A2	1.11E-05	0.462485935
cg17404915	OVOL2	9.24E-05	0.462459411
cg05290058	KCNQ1DN	3.34E-05	0.462439398
cg15310492	KCNA4	5.59E-05	0.462430396
cg13931925	NPHS2	1.50E-05	0.462403708
cg25800765	ALX1	1.11E-05	0.462398925
cg24073466	PHYHIPL;PHYHIPL	1.11E-05	0.462347814
cg13196796	MORN4;MORN4;MORN4	9.24E-05	0.462329065
cg17203063	KCNC2;KCNC2;KCNC2	4.34E-05	0.462319203
cg24719321	BSX	8.60E-06	0.462305619
cg18391899		1.96E-05	0.462280404
cg06645033		2.58E-05	0.462272524
cg03045635	DRD5	0.000117986	0.462270896
cg09495418	GLDN	1.50E-05	0.462212118
cg00110654		6.94E-05	0.46220722
cg08439930	SALL1;SALL1	0.001376172	0.462168633
cg18438793	HPCAL4	9.04E-05	0.462165307
cg26781129	ZNF502;ZNF502;ZNF502;ZNF502	4.34E-05	0.462149694
cg23445003	CUX1;CUX1;CUX1	1.50E-05	0.462122864
cg16792800	GPC6;GPC6	3.34E-05	0.462108926
cg06407470	SV2C	4.34E-05	0.462106478
cg20023231	HS3ST2	1.11E-05	0.462088208
cg17714025	KCNA4	4.34E-05	0.462070682
cg09912667	LPAR3	2.58E-05	0.462058826
cg21010859	BDNF;BDNF;BDNF;BDNF;BDNF	8.60E-06	0.462048512
cg24974423	HS6ST3	1.50E-05	0.462039054
cg27139836	CCDC108	2.58E-05	0.462036176
cg10776919	DMRTA2	1.11E-05	0.462034435
cg10573386	CELSR3	0.000187194	0.462024995
cg21836370		5.59E-05	0.462010051
cg19635401	C6orf204;PLN;C6orf204	2.58E-05	0.461988031
cg09042277	TBX5;TBX5;TBX5;TBX5	1.11E-05	0.461970416
cg00095594	NID1	0.000187194	0.461949783
cg18655369		3.34E-05	0.461944241
cg18952647	BNC1	1.50E-05	0.461939692
cg12678562	GPC5	3.34E-05	0.461902818
cg05488043	P2RY1;P2RY1	0.000187194	0.461867058
cg03514351	LEPR;LEPR;LEPR	8.60E-06	0.461827187

cg16135716	GRIN2B	1.96E-05	0.461810633
cg21858380	C1QL2	8.60E-06	0.461781463
cg27315742	VGLL2;VGLL2	8.60E-06	0.461759304
cg21621248	CTNNA2;CTNNA2;LRRTM1	8.60E-06	0.461750713
cg27347269	DOK6	1.96E-05	0.461717298
cg10931190	TSLP	9.24E-05	0.461692068
cg04546041	ZNF304	5.59E-05	0.461680696
cg23536629		7.23E-05	0.461625536
cg00674706		2.58E-05	0.461614574
cg10983199		4.34E-05	0.461613565
cg26843074	STAC	5.59E-05	0.461595533
cg11641791	KRT222	4.34E-05	0.461586157
cg01300410	TMEM20;TMEM20	1.96E-05	0.461585512
cg10177394		3.34E-05	0.461580444
cg07566080	DAB1	9.24E-05	0.461534134
cg25739875	TFAP2A;TFAP2A	1.96E-05	0.461532381
cg09140723	SGPL1	8.60E-06	0.461518887
cg10966580		1.50E-05	0.461518094
cg16630482		3.34E-05	0.4615069
cg26846959	PHACTR2;PHACTR2;PHACTR2;PHACTR2	3.34E-05	0.461486188
cg23255835	CLIP4	0.000187194	0.461477213
cg14510359	MMEL1	3.34E-05	0.46146356
cg08457011		4.34E-05	0.461367858
cg05200313	SLC24A4;SLC24A4;SLC24A4	4.34E-05	0.461366591
cg12521353	TFAP2C;TFAP2C	5.59E-05	0.461358038
cg10767350		8.60E-06	0.46135669
cg23524195	GFRA1;GFRA1	0.000654035	0.461340288
cg13921352	FAM19A4;FAM19A4	1.50E-05	0.461330446
cg01908954	SCG3;SCG3;SCG3;SCG3	7.23E-05	0.461312275
cg05555111		8.60E-06	0.461241608
cg18530551		5.59E-05	0.461233773
cg14703605	TRPA1	8.60E-06	0.461222746
cg24941342		0.000117986	0.461159271
cg26690949		0.000187194	0.461125724
cg12630082	PKDCC	2.58E-05	0.461081695
cg22335876		1.96E-05	0.461073995
cg25082959	FGF14	0.000290372	0.461069812
cg00369414	CACNA1H;CACNA1H	0.000148457	0.461066038
cg19352038	PAX3;PAX3;PAX3;PAX3;PAX3;CCDC140;PAX3	8.60E-06	0.461037404
cg21263566		4.34E-05	0.461024581
cg14789818		8.60E-06	0.461023916
cg06634914		1.11E-05	0.461022823
cg02796279		3.34E-05	0.461021281
cg11437784		0.000458355	0.46097955
cg18517195	LRRC49;THAP10	8.60E-06	0.460975424
cg26394257	ADAM32	8.60E-06	0.460949815
cg10055501	PITX2;PITX2;PITX2	0.000117986	0.46094543
cg04904784		7.23E-05	0.460944881
cg02990869	MARCH1	8.60E-06	0.46092859
cg21773967	PMP22;PMP22	8.60E-06	0.460928112
cg06207120		1.11E-05	0.460924731
cg21801378	BRUNOL6	1.50E-05	0.460916989
cg03192598		7.23E-05	0.460860684
cg07068756	UCHL1;UCHL1	1.96E-05	0.460844358
cg02172857	LCA5;LCA5	0.00043997	0.460812709
cg11659501	ACSS3	1.11E-05	0.460806355
cg01671895	PGR	9.24E-05	0.460792301
cg13996155	FGF10	1.96E-05	0.460789902
cg06972969	PHYHIPL	8.60E-06	0.460759293
cg19119032	CDH7;CDH7	8.60E-06	0.460755925

cg02492791	SLC6A15;SLC6A15;SLC6A15;SLC6A15;SLC6A	1.11E-05	0.460742633
cg07744841	SLC6A11	1.50E-05	0.460721339
cg22065614	GLRB;GLRB;GLRB;GLRB;GLRB	0.000117986	0.460717461
cg22766145	BHLHE22	8.60E-06	0.460712785
cg21385666	SOX1;SOX1	1.50E-05	0.460698639
cg12456799	SORCS3	1.96E-05	0.460687208
cg14891209	LOC145845	6.94E-05	0.460686058
cg02485347	DOCK5	3.34E-05	0.460650531
cg03521736	PPAP2C;PPAP2C;PPAP2C	2.58E-05	0.460639958
cg15233892	VGLL2;VGLL2	2.58E-05	0.460614403
cg10679301		0.000233422	0.460593798
cg12881363	MOV10L1;MOV10L1;MOV10L1	1.96E-05	0.46059038
cg03614193	ST8SIA3	1.11E-05	0.460590325
cg24813736		3.34E-05	0.460560587
cg00379720		3.34E-05	0.46050715
cg13138137	BRUNOL4;BRUNOL4;BRUNOL4;BRUNOL4	0.000148457	0.460454039
cg06807397	SERP2	8.60E-06	0.460444018
cg06792340		1.50E-05	0.460417122
cg03078269		0.000233422	0.460375901
cg01894048	KCNN2	1.50E-05	0.460373619
cg16312002		4.34E-05	0.46035465
cg24369728		8.60E-06	0.460348257
cg22538780	LOC100192378	1.50E-05	0.460346296
cg21913681	TBX3;TBX3	9.24E-05	0.460302272
cg11343211		0.000117986	0.460297969
cg13088556		1.96E-05	0.460288122
cg13356117	TTC12	8.60E-06	0.460270617
cg13208922		1.11E-05	0.460267003
cg13022679	PCDHA2;PCDHA1;PCDHA1;PCDHA6;PCDHA5	7.23E-05	0.460266452
cg19102955	TRMT6	0.000187194	0.460256048
cg20714110		3.92E-05	0.460237
cg16204818		2.58E-05	0.460234442
cg22773899		8.60E-06	0.460233388
cg20591472	SYPL2	8.60E-06	0.460188169
cg06008724	PHF21B;PHF21B	8.60E-06	0.460161197
cg03145658		4.34E-05	0.460154659
cg09938511	LOC100192378	2.58E-05	0.460137112
cg21820677	GABRA2;GABRA2	8.60E-06	0.460124155
cg15094819	DNAH9	8.60E-06	0.460070769
cg17227156	BRUNOL4;BRUNOL4;BRUNOL4;BRUNOL4	8.60E-06	0.460042266
cg09727046	MIR548H4	2.58E-05	0.460030006
cg05679507	ART5;ART5;ART5	0.000117986	0.459939218
cg24637426	FGF12	1.96E-05	0.459904136
cg01138867	GABRB2;GABRB2	1.50E-05	0.459827386
cg08036492	COX10	2.58E-05	0.459819786
cg21928701	LPPR3	4.34E-05	0.459806347
cg08992305	TRIM61;C4orf39	1.11E-05	0.459793661
cg23358612	UNCX	8.60E-06	0.459776049
cg03646916	ZNF167;ZNF167	0.000537905	0.459762842
cg12165551		1.50E-05	0.459740157
cg14455170		0.000290372	0.459739963
cg26788672		1.96E-05	0.459734939
cg21278103	PTPRA;PTPRA;PTPRA	0.000357885	0.459710518
cg25078150		4.34E-05	0.459708548
cg02029926	CXCL1	1.50E-05	0.459685196
cg07599133	LOC643719	7.23E-05	0.459665593
cg09062595	RND3	1.96E-05	0.459650232
cg07254054	EPHA6	4.34E-05	0.459649871
cg21399832		8.60E-06	0.459639089
cg25330843		2.58E-05	0.459593606

cg20727434		3.34E-05	0.459586707
cg16589214	CALCR;CALCR	1.11E-05	0.459584845
cg01254303	SRRM4	0.000537905	0.459543363
cg13331196		2.58E-05	0.459498017
cg07821629	AMPH;AMPH	2.58E-05	0.45949636
cg09896412		2.58E-05	0.459493341
cg10717463	SALL3	1.96E-05	0.459474906
cg07068674		2.58E-05	0.459474822
cg03927037	ARHGAP20	9.24E-05	0.45946707
cg15104158	FGF12	1.96E-05	0.459445032
cg03378876	KIAA1755;KIAA1755	2.58E-05	0.459406821
cg04954559	SLC17A6	3.34E-05	0.459386654
cg09558195	GRM1;GRM1	1.50E-05	0.459372952
cg12399700	LHX5	3.34E-05	0.459329242
cg02884176	FOXI3	1.96E-05	0.45932657
cg24131595	GRP;GRP;GRP	1.50E-05	0.459303908
cg06954481	GBX2	1.96E-05	0.459290206
cg16804284	FOXG1	1.96E-05	0.459269467
cg11678324	TLL1	1.50E-05	0.459243787
cg24234899		3.34E-05	0.459240108
cg16441068	RCOR1	0.000117986	0.45920688
cg21660960	SLC40A1	4.34E-05	0.459182278
cg16457786	ADCYAP1;ADCYAP1	5.59E-05	0.459165247
cg17478979	ZC3H12D	5.59E-05	0.459115477
cg04688828	LYNX1;LYNX1;LYNX1;LYNX1;LYNX1	5.59E-05	0.459110752
cg00405688	FOXI2	8.60E-06	0.45908976
cg08279184	WASF3	5.59E-05	0.459076269
cg24490542	CSMD2	5.59E-05	0.459059216
cg26844246	TLX3	8.60E-06	0.458994858
cg08441170	MYO3A;MYO3A	0.000148457	0.458982484
cg12331389	RBP4	2.58E-05	0.458869092
cg05676400		1.50E-05	0.458821621
cg17591816	DNM3;DNM3	0.000117986	0.458791639
cg21549299	C21orf88;C21orf88	7.23E-05	0.45879101
cg02229775	CACNG4	2.58E-05	0.458786957
cg02928699	GRM5;GRM5	1.96E-05	0.458783922
cg06989930	DBX1	0.000158823	0.45878143
cg20247486		5.59E-05	0.458742209
cg09022422	SLC6A11	8.60E-06	0.458732809
cg04749646	MARCH4;MARCH4	2.58E-05	0.458705695
cg12158471	RGS7BP	5.59E-05	0.458691113
cg17459204	PREX2;PREX2	2.58E-05	0.458669314
cg07622493		8.60E-06	0.45862775
cg20357538	CHSY1	7.23E-05	0.458605693
cg05043349		4.34E-05	0.458604679
cg24847829	SPOCK1	0.000187194	0.458580898
cg00310215	SALL1;SALL1	9.04E-05	0.458564143
cg19753526	FHL2;FHL2;FHL2;FHL2;FHL2;FHL2;FHL2	0.000148457	0.458550313
cg11551464		7.23E-05	0.458509704
cg03134157		0.000187194	0.458488634
cg17994569	CHAT;CHAT;CHAT;CHAT;CHAT;CHAT;CHAT	9.24E-05	0.458468253
cg10735015	PHACTR2;PHACTR2	0.000148457	0.458435781
cg26124318	ADAM32;ADAM32	1.11E-05	0.458427966
cg11427510	ADAMTS9	0.000290372	0.458423287
cg01642521		0.000117986	0.458411545
cg06001524	STX8	4.34E-05	0.458410219
cg00159243	SELPLG	3.34E-05	0.458404862
cg24497541	DHCR24;DHCR24	1.96E-05	0.458357268
cg18292664	DBX1	2.58E-05	0.458356718
cg15235798		1.50E-05	0.458349998

cg24624901		8.60E-06	0.458331778
cg07382454	TM6SF2	4.34E-05	0.458269509
cg08441806	NKX6-2	8.60E-06	0.458236441
cg10961604	CD164L2;CD164L2	5.59E-05	0.458224364
cg13513864	REM1	4.34E-05	0.458182611
cg03625287		8.60E-06	0.458180132
cg16640599	SEC24D	2.58E-05	0.458120905
cg12116288		1.11E-05	0.458112961
cg01357429	EVX1	0.00043997	0.458062706
cg21331088	ADCYAP1;ADCYAP1	1.96E-05	0.458052176
cg21882300		8.60E-06	0.458025765
cg19365673	LPPR5;LPPR5	5.59E-05	0.458022985
cg22528270	PRKAG2;PRKAG2	5.59E-05	0.458011544
cg23334433	AATK	3.34E-05	0.457977658
cg21568106		2.58E-05	0.457973349
cg04763554		1.50E-05	0.457947705
cg16768018	ZIC4;ZIC4;ZIC4;ZIC4;ZIC4	5.59E-05	0.457929276
cg03857198		2.58E-05	0.45792403
cg07212778	C1QL2	8.60E-06	0.457908012
cg04671932	MAGI2	1.11E-05	0.457897958
cg06842954	PITX2;PITX2	0.000187194	0.457890487
cg25923609		0.000791389	0.45783987
cg19350360		3.34E-05	0.457804959
cg03949391	PTGFR;PTGFR;PTGFR;PTGFR	1.11E-05	0.457767973
cg13416213		5.59E-05	0.457767657
cg18181323	PRDM5	4.34E-05	0.457759329
cg09054633	SPOCK1	8.60E-06	0.457703
cg11663376		1.96E-05	0.457698024
cg25208479		7.23E-05	0.457687394
cg04184232	C14orf23;C14orf23	1.50E-05	0.457672651
cg05663341	DLX6AS;DLX6	0.001147425	0.457659298
cg03175653	GALR1	8.60E-06	0.457654593
cg02938205	CCDC36;CCDC36	1.96E-05	0.457643513
cg26444528	PCDH17	3.34E-05	0.457637563
cg10903451	FIGN	3.34E-05	0.457631519
cg12092090	CACNA1A;CACNA1A	0.000233422	0.45757661
cg00112952	OR2B11	4.34E-05	0.457567974
cg27487839	XKR6	0.000537905	0.457551792
cg24753760	GRIK2;GRIK2;GRIK2	1.11E-05	0.457514717
cg07091500		0.000791389	0.457489724
cg17003293	INSM2	9.24E-05	0.457475274
cg24436715		2.58E-05	0.457474624
cg04940329	KRT23	8.60E-06	0.457329734
cg06066700	FAM124B;FAM124B	0.00043997	0.457314023
cg23936531		2.58E-05	0.457305627
cg22724998		0.000158823	0.457265568
cg20513548	GPR85;GPR85;GPR85;GPR85;GPR85	8.60E-06	0.45719324
cg07726139		8.60E-06	0.457165708
cg26464821	DLC1;DLC1;DLC1	0.000526175	0.457138497
cg13382322	BRUNOL4;BRUNOL4;BRUNOL4;BRUNOL4	9.24E-05	0.45711853
cg15720043	LOC100192378	8.60E-06	0.457115293
cg09279240	ZNF385B;MIR1258	9.24E-05	0.457074526
cg10186131		4.34E-05	0.457066366
cg21383151	TBC1D12	2.58E-05	0.457057051
cg22864077		1.11E-05	0.457012876
cg22557662	PPP1R14A	0.00043997	0.457005312
cg01225698	BDNF;BDNF;BDNF;BDNF;BDNF	1.11E-05	0.456993511
cg23497704	LHX5	3.30E-05	0.456983614
cg26156256		4.34E-05	0.456956789
cg11733675	NLGN1	1.96E-05	0.456955355

cg04090392	BNC1	1.50E-05	0.456943193
cg18417423	ELOVL4	1.96E-05	0.456908682
cg08774443		0.000148457	0.456903283
cg00525823	FAM38B	1.50E-05	0.4568936
cg06955484	TAL1;TAL1	2.58E-05	0.456857429
cg13092806		0.00043997	0.456826171
cg11213369		1.11E-05	0.456768258
cg15139644	BRUNOL4;BRUNOL4;BRUNOL4;BRUNOL4	4.34E-05	0.456761214
cg14814195	FOXQ1	8.60E-06	0.456758442
cg11711420	ISL1;ISL1	4.34E-05	0.456735608
cg17515837	C15orf27	3.34E-05	0.456719125
cg20532370	RBP1;RBP1;RBP1	0.000290372	0.456699529
cg08690859	FLJ32063	4.34E-05	0.456685448
cg18175036	MAST4;MAST4	0.001195582	0.456658375
cg13974192		2.58E-05	0.456614075
cg08004278		8.60E-06	0.456556045
cg23487201	APCDD1L	1.96E-05	0.456545953
cg07811198	DPP6	1.50E-05	0.456539354
cg22340508		1.50E-05	0.456538054
cg23015696	LEPREL1;LEPREL1	8.60E-06	0.456522831
cg13358636	CNTNAP5	4.34E-05	0.456499702
cg03757784	HS3ST2	8.60E-06	0.456499445
cg17486097	UNC5D	4.34E-05	0.456478732
cg06315974	ADAR	8.60E-06	0.456450325
cg01552272	MACROD2	5.59E-05	0.456420361
cg26896572	C3orf14	0.000148457	0.456408533
cg27052403	TTC12	7.23E-05	0.456372743
cg03145999	ZSCAN18;ZSCAN18	9.24E-05	0.456367764
cg01662869	MGRN1;MGRN1;MGRN1;MGRN1	4.34E-05	0.456342404
cg03469054	TMEM132D	8.60E-06	0.456330223
cg16248329	FAT1	0.001376172	0.456326404
cg19055828	DIP2B	1.11E-05	0.456322645
cg23169883	ADAMTS14;ADAMTS14	7.23E-05	0.456315374
cg05791136	COL25A1;COL25A1	8.60E-06	0.45629939
cg13407456		1.96E-05	0.456275444
cg06651311	KIAA1239	2.58E-05	0.45624089
cg19275261		2.58E-05	0.456232702
cg01765111		3.34E-05	0.456204066
cg03422911	RYR2	1.96E-05	0.45619601
cg24675998	KY	8.60E-06	0.456066822
cg26490054	SLC5A8	1.96E-05	0.456049635
cg08107689		1.96E-05	0.456042089
cg18490614		8.60E-06	0.456008936
cg16019434		8.60E-06	0.45599767
cg14615336	AGBL4;AGBL4	8.60E-06	0.455947765
cg10094616	FAM150A	1.96E-05	0.455886588
cg20457796	SLC18A3;CHAT	8.60E-06	0.455885543
cg23166865		1.96E-05	0.455876461
cg21957905	SORCS2	1.50E-05	0.45586974
cg09686443	CCDC37	1.11E-05	0.455865938
cg08894594	INADL	0.000357885	0.455860331
cg03384825	DAB1	0.000117986	0.455740399
cg11452391	MARCH11	8.60E-06	0.455720871
cg22916722		7.23E-05	0.455713538
cg15823845	ZFR2;ZFR2;ZFR2	5.59E-05	0.455684002
cg27046936	PTGFR;PTGFR;PTGFR;PTGFR	8.60E-06	0.455666053
cg05956498	EFHA2	1.11E-05	0.455626845
cg11908131	C2orf40	4.34E-05	0.45561603
cg22135752	LOC255167;LOC255167	1.11E-05	0.455613204
cg14283944		1.50E-05	0.455612265

cg12728578	ZNF204P	9.24E-05	0.455584227
cg01729827	CBLN4	4.34E-05	0.455570253
cg05880330		2.58E-05	0.455561081
cg20659435		3.34E-05	0.455559767
cg25024717		8.60E-06	0.455555906
cg10715223	SNX31;SNX31	3.34E-05	0.455545205
cg18836626	ATP8A2	8.60E-06	0.455535396
cg11107212		4.34E-05	0.455431538
cg01178451	GALR1;GALR1	3.21E-05	0.455352132
cg21054521		2.58E-05	0.455340649
cg26461267		4.34E-05	0.455339475
cg02745211	TMEM108;TMEM108	0.000117986	0.455336547
cg00563873		5.59E-05	0.455330015
cg11183632		2.58E-05	0.455284946
cg09501509		1.96E-05	0.455255046
cg10171448	NKX6-2	1.50E-05	0.455230944
cg12072560	CIDEA;CIDEA	7.23E-05	0.455198705
cg08688659	CD44;CD44;CD44;CD44;CD44	3.34E-05	0.455165526
cg09090724	TMEM108;TMEM108;TMEM108;TMEM108	1.96E-05	0.455122217
cg25201047		8.60E-06	0.455112109
cg17204129	CFTR	1.11E-05	0.455104187
cg24645214	RGS20	0.000117986	0.455090092
cg27341472	TMEM196	9.04E-05	0.455044686
cg11486133	GRID2	1.11E-05	0.455013312
cg06313119	FGF14	0.001376172	0.454987085
cg26812418	CPE	1.11E-05	0.454976188
cg02746381	FAM84A;FAM84A	7.23E-05	0.454969268
cg02391713		2.08E-05	0.454952394
cg01382110		8.60E-06	0.454950975
cg08181476	NCAN	8.60E-06	0.454895993
cg13321077	RESP18	8.60E-06	0.454892011
cg22865470		3.34E-05	0.454875095
cg10263370	ZNF502;ZNF502;ZNF502;ZNF502	1.50E-05	0.454849488
cg08931129		5.59E-05	0.454828026
cg04184836	CPEB1;CPEB1	1.96E-05	0.454802613
cg06572160	KCNC3	3.34E-05	0.454776543
cg00667789	TACSTD2	9.24E-05	0.45473843
cg05667348	VAX1	9.24E-05	0.454736543
cg04053392	DACT2	3.34E-05	0.454723941
cg20006924	RORA	5.11E-05	0.454656767
cg25862644	C10orf41;C10orf41	4.34E-05	0.45463714
cg15191798	LRRC49;THAP10;THAP10	8.60E-06	0.454606805
cg12036735		0.000187194	0.454554639
cg13482209	CELSR3	2.58E-05	0.45455072
cg27584135		0.000290372	0.454510077
cg02058408		3.34E-05	0.454505249
cg24437715		2.58E-05	0.454494295
cg19998368	C12orf56;C12orf56	7.23E-05	0.454437724
cg02973171	FAM181B	0.000233422	0.454434785
cg09141965		5.59E-05	0.454391892
cg19883384		3.34E-05	0.454362999
cg12409982		8.60E-06	0.454349967
cg06002370	COL4A3;COL4A3;COL4A3;COL4A3;COL4A4	1.50E-05	0.454324627
cg05560435		4.34E-05	0.454320673
cg03568673	GJB6;GJB6;GJB6;GJB6	2.58E-05	0.454304224
cg15026241		2.58E-05	0.454238055
cg08643994		1.96E-05	0.454221769
cg24700959	ESRRG;ESRRG;ESRRG;ESRRG;ESRRG	3.34E-05	0.454201122
cg07729537		8.60E-06	0.454186879
cg21884421	IGDCC3	8.60E-06	0.454168792

cg16459364	NEFM;NEFM	8.60E-06	0.454162308
cg22047387	NEUROD4;NEUROD4	1.11E-05	0.454142818
cg11064034		1.50E-05	0.454133156
cg12307787	KCNB1	0.000122184	0.454118239
cg15090727	SVEP1;SVEP1	8.60E-06	0.454113824
cg07861448	MSC	5.11E-05	0.454095126
cg22848646		1.96E-05	0.454044655
cg14272075	RNF126P1	5.59E-05	0.454025115
cg27072996	LPPR3	0.000233422	0.454017689
cg11039614	ISL1;ISL1	8.60E-06	0.453998428
cg19082230	ODZ3	5.59E-05	0.453992833
cg22879515	MIR34B;BTG4;MIR34C	0.000187194	0.453979961
cg25999578	HOXA9	4.34E-05	0.453927113
cg20631750	BMP7	0.000117986	0.453920218
cg17985029	EYA4;EYA4;EYA4	0.000791389	0.453917885
cg26551913	PAX2;PAX2;PAX2;PAX2;PAX2	4.34E-05	0.453917599
cg23231729	THEM4	0.000955793	0.453910968
cg23434186	DOK6	8.60E-06	0.45389566
cg19754707		8.60E-06	0.453887339
cg11610350		5.59E-05	0.45387627
cg08123277	THRB;THRB;THRB	0.000233422	0.453875691
cg00046625	PDE4B;PDE4B	1.11E-05	0.453845389
cg23563014		1.11E-05	0.45381768
cg00462168	KIAA1024	3.34E-05	0.453806161
cg10028217	CADPS2;CADPS2;CADPS2	0.000117986	0.453769699
cg26435670	BMPR1A	0.000187194	0.453758543
cg13297865	ELOVL4	1.50E-05	0.453739879
cg16654911		8.60E-06	0.453737622
cg21042456		5.59E-05	0.453729046
cg17528648	OSMR;OSMR;OSMR;OSMR	0.000117986	0.45371857
cg06573787		0.000233422	0.453689948
cg19170015	CXCL1	8.60E-06	0.453677424
cg20292547	BMP7;BMP7	5.59E-05	0.453652796
cg15731056	C12orf56;C12orf56	4.34E-05	0.453640951
cg16043357	VAX1;VAX1	7.23E-05	0.453561996
cg15060599		8.60E-06	0.453556584
cg05081498		1.11E-05	0.453555344
cg08857063	TFAP2B	0.00041966	0.453551438
cg20289688	MYOD1	2.58E-05	0.453506538
cg18734428	ADAMTSL3	1.50E-05	0.453504396
cg11638399		9.24E-05	0.453483041
cg16749235	EBF2	4.34E-05	0.453451431
cg10836392	SULT4A1	1.50E-05	0.453396061
cg20786074	EFEMP1;EFEMP1;EFEMP1;EFEMP1	1.11E-05	0.453383879
cg25514273	KLHL1;ATXN8OS	7.23E-05	0.453362418
cg18540492	AGBL4	1.11E-05	0.453352594
cg10074409	IRF6;IRF6	2.58E-05	0.453344707
cg17754510		1.50E-05	0.453316271
cg14314029		3.34E-05	0.453304771
cg12302070	KCNH7;KCNH7	8.60E-06	0.453293861
cg02623400	ELAVL4;ELAVL4	2.58E-05	0.453285319
cg04895288		3.34E-05	0.453284213
cg08229358	NPHS2	1.96E-05	0.453280623
cg00270497	RIPPLY2	1.50E-05	0.453270065
cg17790852	ODZ3	5.59E-05	0.453257643
cg14858469	NR2F2;NR2F2	8.60E-06	0.453231413
cg07206208	LOC100128811;GPR158	8.60E-06	0.45322339
cg08414570		1.11E-05	0.453198332
cg24374738	CRYBA2;CRYBA2;CRYBA2	7.23E-05	0.453156235
cg14568830		8.60E-06	0.453150952

cg04977834		0.000117986	0.453134259
cg25345178		8.60E-06	0.453092424
cg24762231	NFE2;NFE2	1.50E-05	0.453023758
cg03551161	OSBP2	1.96E-05	0.453008216
cg27642470	DCBLD1	1.11E-05	0.45299797
cg25181751		1.50E-05	0.452972894
cg25235766	CACNG8;MIR935	0.001376172	0.452947383
cg26398467		1.50E-05	0.452944095
cg18451814	OTX2	8.60E-06	0.452938555
cg08867825	OLFM3	2.58E-05	0.452919676
cg20249252		9.24E-05	0.452918811
cg26608883	CALCB	0.000233422	0.452890621
cg18781988	PCDHGA2;PCDHGA3;PCDHGA1;PCDHGA3	0.000290372	0.452765798
cg20909645		1.96E-05	0.452750689
cg26763877	CSMD1	5.59E-05	0.452725939
cg15837233	TBR1	1.60E-05	0.452716928
cg18806140	CTNNA2;CTNNA2	1.50E-05	0.452683549
cg16910830	CNTNAP2	3.34E-05	0.452665774
cg00423153	SPHKAP;SPHKAP	1.96E-05	0.452593128
cg21925310	SULT4A1	1.11E-05	0.452543224
cg20055426		1.11E-05	0.452533721
cg21982950		2.08E-05	0.452520571
cg12699371	GALR1	1.11E-05	0.452394554
cg05372242	SFTA3	2.80E-05	0.452354764
cg05736768	DPYS;DPYS	1.11E-05	0.452342695
cg13574390	NTRK1;INSRR	1.18E-05	0.452339812
cg11611600	ADAMTSL3	8.60E-06	0.45232179
cg14370314	EFS;EFS	5.59E-05	0.452301303
cg24418420	SLC6A15;SLC6A15;SLC6A15;SLC6A15;SLC6A	4.34E-05	0.452267794
cg17062829		4.34E-05	0.452236106
cg23193759	C10orf35	9.24E-05	0.452218911
cg16566627	LRAT	4.34E-05	0.452159899
cg24427895		1.11E-05	0.452152464
cg25253677	CACNG8	0.001948248	0.452126671
cg01832005	C3orf14	1.11E-05	0.452124487
cg19001226	HOXD1;HOXD1	0.000117986	0.452111289
cg06507551	CTTNBP2	1.96E-05	0.452070479
cg04903759	WDR49	1.96E-05	0.452059771
cg00926400	BOLL;BOLL	8.60E-06	0.452051421
cg04682193	KDM2B;KDM2B	1.96E-05	0.452049767
cg04222358		8.60E-06	0.45204532
cg11165158	FOXL1	0.000357885	0.452018068
cg11428724	PAX7;PAX7;PAX7;PAX7;PAX7;PAX7	5.59E-05	0.451998299
cg17300051	KCNJ3	1.96E-05	0.451961984
cg07790752		9.24E-05	0.451932791
cg21878275	ADAMTS9	0.000187194	0.45193276
cg20720059	FAM84A	9.24E-05	0.451903614
cg06617456	PREX2;PREX2	2.58E-05	0.451896013
cg20759084	ADCY2	0.000290372	0.451869284
cg03902462	LONRF1	0.000117986	0.451867111
cg03276408		0.002450672	0.451862048
cg20318608	FAM163A	7.23E-05	0.451850364
cg19095600		1.96E-05	0.451831116
cg12389770	GRIK2;GRIK2;GRIK2	9.24E-05	0.451803279
cg00393585	C4orf39;TRIM61	0.000117986	0.451783669
cg01764252		0.000187194	0.451773383
cg21725716		8.60E-06	0.451759496
cg21123160	SLC6A11	8.60E-06	0.451728729
cg04645070		9.24E-05	0.451724997
cg25460753		1.11E-05	0.451686273

cg01502428	TIMP2	9.24E-05	0.451671962
cg10123247	C8orf85	4.34E-05	0.451669831
cg04981088	MPV17L;MPV17L;MPV17L;MPV17L	0.000526175	0.451656
cg18897025	TSLP;TSLP	2.58E-05	0.45165235
cg08047376	CTNNA2;CTNNA2	3.21E-05	0.45163529
cg02262553		9.24E-05	0.451609772
cg13452260		1.11E-05	0.451600157
cg12258042	CSMD1;CSMD1	7.23E-05	0.451599043
cg18930994	NPY2R	2.58E-05	0.45152955
cg19079845	PAX1;PAX1	8.60E-06	0.451475748
cg09954385	ARHGAP8;ARHGAP8;PRR5-ARHGAP8	2.58E-05	0.451453333
cg21784036	CPNE8	0.000537905	0.45144248
cg19039464	C4orf31;C4orf31	0.000117986	0.451381648
cg00500359	OSBPL5;OSBPL5;OSBPL5	2.58E-05	0.451359744
cg07318073	CXCL3;CXCL3	0.000148457	0.451331784
cg09935045	PRKAA2	3.34E-05	0.451271201
cg11675881		9.24E-05	0.451262008
cg15827779	CDC42BPB	4.34E-05	0.451251335
cg10524152	ADRA2A	2.58E-05	0.45124322
cg24496475		8.60E-06	0.451229396
cg17128947	CPLX1	7.23E-05	0.451222476
cg12497564	RBP1;RBP1;RBP1	9.24E-05	0.451220746
cg15093079	EPHA6	4.34E-05	0.451144019
cg13368923		3.34E-05	0.451136503
cg01631764	SHC2	1.96E-05	0.451135101
cg16002818	BOLL	8.60E-06	0.451125586
cg03936663		1.50E-05	0.451117676
cg18438944	MT1A	5.59E-05	0.451101145
cg17826753	KCNV1	8.60E-06	0.451081351
cg00044107	COL12A1;COL12A1	1.96E-05	0.451061693
cg24583615	UGGT2;UGGT2	1.96E-05	0.451057632
cg24207698	TTC21B	7.23E-05	0.451029385
cg23654401	VOPP1	4.34E-05	0.451023798
cg25648746	RGS7BP	3.34E-05	0.451010328
cg21918126	NRXN3;NRXN3;NRXN3	7.23E-05	0.450997589
cg23365739	OTX2;OTX2	1.50E-05	0.450993156
cg08448701	PAX1	1.96E-05	0.450992783
cg14258623		1.96E-05	0.450983088
cg26639039	SLC12A5;SLC12A5	9.24E-05	0.450965427
cg24566400	RBP4	1.96E-05	0.450946274
cg13255542	THYN1;THYN1;THYN1;THYN1;THYN1	3.34E-05	0.450946149
cg11423323		4.34E-05	0.450942767
cg18802754	VSTM2B	0.000262362	0.450938788
cg07723130		1.50E-05	0.450918614
cg20222562		7.23E-05	0.450911631
cg16172814	OGDHL;OGDHL;OGDHL	1.96E-05	0.450858236
cg10164885	NELL2;NELL2;NELL2;NELL2;NELL2	9.24E-05	0.450832526
cg07558455	KANK4	5.59E-05	0.450798238
cg09465746	GRM7;GRM7	2.08E-05	0.450783226
cg12338552		1.11E-05	0.450763879
cg03389653	CBLN1	3.34E-05	0.450722367
cg04022561		2.58E-05	0.450715266
cg10865087	BICC1	1.50E-05	0.450693321
cg14019317	PROX1	0.000290372	0.45068743
cg20747380	HOXA2;HOXA2	1.96E-05	0.450683142
cg10442325		5.59E-05	0.450655044
cg24597363	MAEA;MAEA	4.34E-05	0.450609788
cg20047489		1.50E-05	0.450601299
cg12897942	EIF5A2;EIF5A2	1.11E-05	0.450561174
cg06839631		2.58E-05	0.450528234

cg18300589	PLK5P	1.18E-05	0.450525652
cg23730181		2.58E-05	0.450521708
cg09498572		3.34E-05	0.450511005
cg05981335	MYF6	8.60E-06	0.450502798
cg02604290	CYP24A1;CYP24A1	7.23E-05	0.450497295
cg23816347	CCDC37	1.50E-05	0.450492833
cg22374233	ADCYAP1;ADCYAP1	8.60E-06	0.450492743
cg11058154	FAM20A	0.000117986	0.450458024
cg08983097	VGLL2;VGLL2	1.11E-05	0.450457701
cg25349154	LOC642597	8.60E-06	0.450444544
cg26649384	RPRM;RPRM	0.000148457	0.450374206
cg01280202	BTC	0.000148457	0.450346834
cg14704758		8.60E-06	0.450300711
cg03070297	DOCK1;FAM196A	4.34E-05	0.450267143
cg03181899	PDLIM3;PDLIM3	1.11E-05	0.450259402
cg07035961	PCDHA1;PCDHA1;PCDHA2;PCDHA2	2.58E-05	0.450255486
cg07329360		8.60E-06	0.450254546
cg18029761	GPR123	7.23E-05	0.450249635
cg09880551	DSCAM;DSCAM	3.34E-05	0.450242933
cg07401324	PTPRJ;PTPRJ	4.34E-05	0.450233811
cg04799343		8.60E-06	0.450232992
cg12071888		5.59E-05	0.450211291
cg19909349	LAMA1	3.34E-05	0.450201228
cg04707332	TBX15	4.34E-05	0.450174779
cg13933080	SPAG6;SPAG6	1.11E-05	0.450141332
cg07548607	ZSWIM2	4.34E-05	0.450127407
cg10472919	FBXL21	1.50E-05	0.450094139
cg15688918	PLAC2	2.58E-05	0.450089856
cg04759439	ERC2	1.11E-05	0.450086515
cg17279458	ZNF502;ZNF502;ZNF502;ZNF502	4.34E-05	0.450081959
cg26848524		2.58E-05	0.450075965
cg20607287	VWDE	8.60E-06	0.450003125
cg01889143		0.001147425	0.449957887
cg11213520	LHX5	2.58E-05	0.44991211
cg23895340	HOXD11	8.60E-06	0.449900866
cg14456683	ZIC1	4.34E-05	0.449888328
cg15228694	CYB5R2	4.34E-05	0.449873066
cg26938272	SIM2;SIM2	0.000148457	0.449856925
cg06522681	CCDC105;CCDC105	1.11E-05	0.449845772
cg07536910	EVX2	2.58E-05	0.449840241
cg19589939		1.50E-05	0.449826223
cg13557668	FBXL21	7.23E-05	0.44982501
cg27642554	GRM1;GRM1	1.96E-05	0.449818098
cg17996619	NKX6-2	8.60E-06	0.44981432
cg02908900	MEOX2	5.59E-05	0.449758891
cg07995179		3.34E-05	0.449717869
cg25437886	LIMD1	0.000537905	0.449712589
cg18159840	CASR;CASR	1.11E-05	0.449700008
cg22534145	SSTR4	2.58E-05	0.449686709
cg03521656	TMC5;TMC5;TMC5;TMC5	3.34E-05	0.449675109
cg24575234	CHRM2;CHRM2;CHRM2;CHRM2;CHRM2;CHRI	8.60E-06	0.449660841
cg18040241	GLB1L2	1.96E-05	0.449647221
cg08871855	FOXI3	7.23E-05	0.449647089
cg13952840		1.96E-05	0.449615466
cg03332469		8.60E-06	0.449568603
cg26116542		1.50E-05	0.449513485
cg26256401		4.34E-05	0.449492413
cg16987524	FBLN7;FBLN7	8.60E-06	0.449490559
cg14001023	PGLYRP1;PGLYRP1	9.24E-05	0.449470623
cg25585712		2.58E-05	0.449439009

cg07557260	ADCY8	8.60E-06	0.449413999
cg17524017	SULF1;SULF1;SULF1;SULF1	1.96E-05	0.449392014
cg25102370	HAND2;NBLA00301;HAND2	1.11E-05	0.449370168
cg03679565	IRX4;IRX4	1.11E-05	0.449366369
cg14985891	CASQ2	0.000117986	0.449366071
cg20435725		9.24E-05	0.449347115
cg04497176	WTIP	9.24E-05	0.44934294
cg12708634	KITLG;KITLG	1.50E-05	0.449322909
cg05760402		8.60E-06	0.449316128
cg22381196	DHODH	4.34E-05	0.449301729
cg27143688		0.000233422	0.449293418
cg24769821	PTH2R	1.11E-05	0.449292394
cg19133221	B4GALNT2;B4GALNT2;B4GALNT2	1.11E-05	0.449257836
cg05383490	FAM155A;FAM155A	1.50E-05	0.449253386
cg19876672	VAX1	1.50E-05	0.449242293
cg26086468		8.60E-06	0.449214297
cg02002551	NEFM;NEFM	0.000148457	0.449200924
cg14983586		1.50E-05	0.449200455
cg12541454		1.96E-05	0.449179486
cg27316626	KCNB2	8.60E-06	0.449176357
cg04945735	PDLIM3;PDLIM3	8.60E-06	0.449174381
cg26718878		0.001948248	0.449122287
cg16504626		3.34E-05	0.4491192
cg26789732	KCNB2	0.000233422	0.449118414
cg11699435	SOX21	1.11E-05	0.449116891
cg11698888		3.34E-05	0.449113975
cg18058689	GPC6;GPC6	0.000357885	0.449107671
cg09022993	SPATA18	3.34E-05	0.449101132
cg06766427	RICH2	0.000148457	0.449083307
cg16718624	ROBO2;ROBO2	8.60E-06	0.449080322
cg21656205	RIMS4	0.000290372	0.449067857
cg21856256		8.60E-06	0.449062175
cg00604202		8.60E-06	0.44905397
cg04612444	PENK;PENK	3.34E-05	0.449016319
cg18177533	KDR	9.24E-05	0.449005747
cg19990744	AIFM3;AIFM3;AIFM3;AIFM3	8.60E-06	0.448988423
cg23805357	LOC100128811;GPR158	1.96E-05	0.448980275
cg11250773	PNPLA5	2.58E-05	0.448972655
cg20944305	P2RY1	0.000233422	0.448955774
cg01915609	RAB32	9.24E-05	0.448955324
cg10721834		8.60E-06	0.448947989
cg09656389	PAX6	1.50E-05	0.448919285
cg10406295	SFRP1	8.60E-06	0.448910162
cg04730794	DOCK2	1.96E-05	0.448885869
cg19384531	TFAP2D	1.96E-05	0.448878672
cg21816539	GRIK1;GRIK1	8.60E-06	0.448873228
cg12670347	TBX5;TBX5	1.11E-05	0.448872149
cg00913949	ESRRG	2.58E-05	0.448870466
cg16181396	ZIC1	8.60E-06	0.44885764
cg11821068		7.23E-05	0.448824066
cg27225309	IKZF2;IKZF2	1.50E-05	0.448782984
cg00375819	SLC6A1	7.23E-05	0.448772343
cg15228928		0.000148457	0.44877048
cg09727050	TNFAIP6;TNFAIP6	4.34E-05	0.448756041
cg26290632	CALB1	2.58E-05	0.44870713
cg04181546		1.11E-05	0.448683454
cg17319486	BRUNOL4;BRUNOL4;BRUNOL4;BRUNOL4	3.34E-05	0.448682831
cg20242427	DAK	7.23E-05	0.448656664
cg11131532	KCNB1	7.23E-05	0.448648386
cg20495645	PHF21B;PHF21B	3.34E-05	0.448638635

cg03242819	DOCK1;FAM196A	0.000117986	0.448630275
cg24507144	FGF14	0.000290372	0.448560473
cg02657401		0.000117986	0.448511554
cg13320291	GFRA1;GFRA1;GFRA1	0.000955793	0.448501745
cg13618516	AATK	1.50E-05	0.44849351
cg18914514	GREB1L	2.58E-05	0.448479574
cg00554413	TACSTD2	8.60E-06	0.448447207
cg00334063	ZIC4;ZIC4;ZIC4;ZIC4;ZIC4	5.59E-05	0.44842733
cg16003102		1.96E-05	0.448374579
cg17037963		2.58E-05	0.448368827
cg22696167		1.11E-05	0.448355543
cg26783127	AATK	5.59E-05	0.448350126
cg12338417	TRIM71	2.58E-05	0.44833919
cg20523861	KLHL1;KLHL1;ATXN8OS	5.59E-05	0.448332213
cg14658804	SLIT3	0.000187194	0.44832147
cg23847712	DRD5	0.000233422	0.448298359
cg05200037	ZC3H12D	5.59E-05	0.448289703
cg10196720	PCDH10;PCDH10	9.24E-05	0.448282865
cg27533288	VAX1;VAX1	2.58E-05	0.448282045
cg13977235		0.000117986	0.448262255
cg08169394	SHISA6	4.34E-05	0.448236297
cg24139093		8.60E-06	0.448225819
cg15075988	VDAC2	5.59E-05	0.448219816
cg00209038	LHCGR	1.11E-05	0.448218844
cg27495903	NEBL;NEBL	0.000187194	0.448193771
cg02861071	OLFM3;OLFM3	1.96E-05	0.448187952
cg16262614	TF	9.24E-05	0.448162548
cg06804312	FGF12	5.59E-05	0.448128039
cg24626554	LOC389458	7.23E-05	0.448109027
cg06647751		1.50E-05	0.448108645
cg00875511	TACR3	8.60E-06	0.448084043
cg22735222	NKX2-3	0.000148457	0.448083567
cg02862467	UBR4	0.000117986	0.44794561
cg09303642	NFE2;NFE2	4.34E-05	0.447939194
cg20698924	OCA2	5.59E-05	0.447910908
cg13944175	HAS1	1.50E-05	0.447904022
cg27297576		1.11E-05	0.447887382
cg00741836	DOK5	9.24E-05	0.447870746
cg02501779	CBLN4;CBLN4	1.11E-05	0.447866731
cg11284147		0.000117986	0.447836592
cg07462540	NXPH1	7.23E-05	0.447770185
cg18236477	ATP8A2	7.23E-05	0.447768982
cg15847198	GUCY1A2	0.000233422	0.447760422
cg22480742	CALD1;CALD1;CALD1;CALD1;CALD1	0.000290372	0.447715579
cg14186937	TRPC6	5.59E-05	0.447714688
cg13601435		0.000290372	0.447705516
cg16184803	PITX2;PITX2;PITX2	8.60E-06	0.447670425
cg12307306	KY	1.50E-05	0.44766579
cg11379081		3.34E-05	0.447658329
cg07859439	PGAP1	0.000187194	0.447630899
cg16817826	FEZF2	0.001376172	0.447587797
cg18274480		1.11E-05	0.447574373
cg07028821		0.000233422	0.447550224
cg11671688	GPR6	1.96E-05	0.447531983
cg19224713	SAMD12;SAMD12	0.000187194	0.447485412
cg20634967		0.003189589	0.447468196
cg17435266		2.58E-05	0.447458969
cg15397374	EFCAB1;EFCAB1;EFCAB1	8.60E-06	0.447430478
cg20892260	NKX2-6	9.24E-05	0.447429415
cg07434271	PAX6	3.34E-05	0.447422802

cg02381539		3.34E-05	0.447415052
cg19438674	GRID1	1.50E-05	0.447411493
cg04876835	PRKAA2	0.000158823	0.447394221
cg08576864	GPM6A;GPM6A	9.04E-05	0.447390354
cg14730085	FBXO27	0.000148457	0.447335351
cg19861117	NOVA2	1.96E-05	0.447324636
cg16125725		5.59E-05	0.447301694
cg07275179	ATXN7;ATXN7	3.34E-05	0.447266558
cg16826874	DOCK5	0.000148457	0.447263276
cg02202980	C3orf15;C3orf15	0.000187194	0.447239047
cg24394172	GABRA4	2.58E-05	0.447236502
cg21285555	PCMTD1	2.58E-05	0.447230485
cg02854695	KCNC3	1.11E-05	0.447227883
cg23956737	CADPS2;CADPS2;CADPS2	0.000148457	0.44722713
cg01857829	CDH1	0.000290372	0.447199028
cg21821833	CCK	8.60E-06	0.447190651
cg17491850	DAB1	8.60E-06	0.447168382
cg26327071	HOXB4	0.000117986	0.447150512
cg07251711	NKX6-2	8.60E-06	0.447115168
cg05886087		2.58E-05	0.447103891
cg10527010	SLC6A11	1.11E-05	0.447103706
cg18588589		1.60E-05	0.447101223
cg23650423	TRPC6	1.50E-05	0.447090535
cg19610529		1.11E-05	0.447082106
cg27185978	DOCK5	5.59E-05	0.447075807
cg26730369	ADRA1A;ADRA1A;ADRA1A;ADRA1A	7.23E-05	0.447065909
cg05783915	EMX2;EMX2;EMX2OS;EMX2;EMX2	7.23E-05	0.447048961
cg19502867	FAM110B	0.003154996	0.447033476
cg00613752	GPR6;GPR6	8.60E-06	0.44700008
cg10507275	LRRN1;LRRN1	0.000233422	0.446980343
cg14473102	HOXD8	0.000537905	0.446950878
cg01370541		1.11E-05	0.446914966
cg14268557	TRIM27	2.58E-05	0.446891694
cg18184411	ZYG11A	8.60E-06	0.446890575
cg10116864	CDK5R2;CDK5R2	1.96E-05	0.446873282
cg12744859	LOC404266;HOXB5;LOC404266;LOC404266;L(	0.000357885	0.446857428
cg09793121	OLIG2;OLIG2	4.34E-05	0.446855474
cg07912789	PRMT8	9.24E-05	0.446833078
cg15759056	ABCB1	1.96E-05	0.446817061
cg06092815	SPHKAP;SPHKAP	8.60E-06	0.446804801
cg18543270	EPB41L3	9.24E-05	0.446766631
cg13951042	COL4A3;COL4A3;COL4A3;COL4A3;COL4A3;C(	1.96E-05	0.44675878
cg07576142	GPC6	0.000148457	0.446743587
cg24686551		7.23E-05	0.446728253
cg11014468	DCP1A	1.50E-05	0.446692315
cg12368612		8.60E-06	0.446654892
cg16174029	PCDHAC2;PCDHAC2;PCDHA7;PCDHA12;PCD	5.59E-05	0.446621222
cg02631838	HPCA	4.34E-05	0.446610617
cg09713234	MKX	8.60E-06	0.446589355
cg07700233		3.34E-05	0.446573393
cg25062797	VAX1;VAX1	8.60E-06	0.446568585
cg11710851		8.60E-06	0.446536993
cg27598107		8.60E-06	0.446531593
cg24262066	ADAMTS1	9.24E-05	0.446501315
cg24083324	PROX1	0.000187194	0.446497998
cg21546184		1.11E-05	0.446427964
cg00240734	SLC35F1	7.23E-05	0.446408933
cg01491219	C1orf115	0.000537905	0.446350996
cg26342078		8.60E-06	0.446330552
cg14419187	UNC80;UNC80	5.59E-05	0.446290517

cg07791011		7.23E-05	0.446286693
cg20787173	EYA4;EYA4;EYA4	0.000187194	0.446257451
cg21170041	GDA;GDA	1.50E-05	0.446235088
cg24079727	TCF12;TCF12;TCF12;TCF12	3.34E-05	0.446222965
cg09848096		0.000148457	0.446172283
cg00690431	PTGS2	1.11E-05	0.446129721
cg15140703	STAG3;GPC2	0.000357885	0.446110135
cg00358442	DPY19L2P4	1.50E-05	0.446092336
cg23745290	PPA2;PPA2;PPA2;PPA2;PPA2	3.34E-05	0.446090148
cg07283015	HRH4;HRH4;HRH4	1.50E-05	0.44603023
cg10911619	TBC1D12	1.50E-05	0.446028427
cg03730428		8.60E-06	0.446025942
cg25919177		0.000233422	0.446018025
cg05455720	MIR124-2	8.60E-06	0.446014515
cg10141715	SLC5A8	8.60E-06	0.445941724
cg06065125		8.60E-06	0.445918131
cg00824018	INA	1.11E-05	0.445893844
cg00318320	BOK	0.000233422	0.445868604
cg15446670	ABCB1;RUNDC3B;RUNDC3B;RUNDC3B	0.000117986	0.445857947
cg19166660	DAB1	7.23E-05	0.445841067
cg05266564		1.11E-05	0.445820159
cg14022913	NEIL3	1.11E-05	0.445794068
cg18450168	TACC2;TACC2	0.000290372	0.445785817
cg20025003	TFCP2L1	0.000791389	0.445749157
cg08505243		8.60E-06	0.445736017
cg25023058	TEKT3	8.60E-06	0.445731095
cg16438655	DOC2B	7.23E-05	0.445727627
cg21102477	CSMD2	1.96E-05	0.44572119
cg06062984		1.50E-05	0.445718894
cg00754253	HRASLS5;HRASLS5;HRASLS5	0.001376172	0.445697673
cg21357291		5.59E-05	0.445688839
cg07124687		0.005897668	0.445684777
cg02987928		8.60E-06	0.445622959
cg00988148		2.58E-05	0.445622796
cg14894848	FAM19A5	8.60E-06	0.445617046
cg22689833		0.000148457	0.445571019
cg16884042	SGPP2	1.50E-05	0.445571004
cg17206393	TRPC4AP;TRPC4AP	0.000290372	0.445569127
cg11717507	TBX5;TBX5;TBX5;TBX5	5.59E-05	0.445554597
cg23051578	TMEM74	3.34E-05	0.445530282
cg22616881	DLX6AS	1.50E-05	0.445530186
cg05079049		2.58E-05	0.445484612
cg06304401	NLGN1	0.000148457	0.445435967
cg00333512		7.23E-05	0.445433809
cg14583606	SLC1A1	0.000117986	0.445409628
cg17298751	SLC17A6	1.11E-05	0.445392711
cg02773433	GPR83	1.11E-05	0.445390632
cg13942157	FAM189A1	3.34E-05	0.445373057
cg18016365	GABRB1;GABRB1	1.11E-05	0.445365251
cg11017382	MAST4;MAST4	0.000537905	0.445358747
cg21164131	AVPR1A;AVPR1A	2.58E-05	0.445353048
cg09227138	SNAP91;SNAP91	8.60E-06	0.445351444
cg22187630	CACNA1A;CACNA1A	8.60E-06	0.44534895
cg25079102	TBX5;TBX5;TBX5;TBX5	1.50E-05	0.445328004
cg00012397	KCNC1;KCNC1	0.000290372	0.445310003
cg09535475	EML4;EML4	4.34E-05	0.445305949
cg27019126	RAX	1.96E-05	0.44529181
cg03052869	GRID1	0.000187194	0.445257994
cg17230874		9.24E-05	0.445250613
cg24826696	FOXI2	1.96E-05	0.445226593

cg01593834	POU4F3	4.34E-05	0.445214894
cg02551743	MEIS1	8.60E-06	0.445202127
cg24717159	SLC27A2;SLC27A2	1.96E-05	0.445151406
cg20677901	TP73	7.23E-05	0.445090773
cg04118119	ESD	5.59E-05	0.445070756
cg16793394	OTX2	8.60E-06	0.445070286
cg06907418	TRPV3	2.58E-05	0.44505597
cg06312813	CXCL2	0.000187194	0.445054726
cg10740902	KDR;KDR	3.34E-05	0.445021831
cg09767602	ODZ3	5.59E-05	0.445005761
cg19439706	TRPA1	1.11E-05	0.444984788
cg17023770	PCDHB15	8.60E-06	0.444971168
cg20585869	NEFM;NEFM	0.000187194	0.444969492
cg06226256		8.60E-06	0.444953678
cg04442576	SLC35F1	1.50E-05	0.444941524
cg14777768	SLC2A2	5.59E-05	0.444932182
cg11419506	MKX	1.96E-05	0.444922546
cg12071328	NELL1;NELL1	0.000537905	0.444912135
cg00705730	NCK2;NCK2	8.60E-06	0.444883409
cg00630958	LEPR;LEPR;LEPR	1.50E-05	0.444870071
cg16787600	SORCS3;SORCS3	8.60E-06	0.444867588
cg09601770	DPP4;DPP4	0.001147425	0.444837556
cg02447229	TMEM181	0.00043997	0.444828038
cg10741153		1.11E-05	0.444827191
cg21814550		1.11E-05	0.444816415
cg17799946		1.50E-05	0.444791543
cg11054631	LOC283392;TRHDE;LOC283392	7.23E-05	0.444789288
cg09873164	CRCT1	0.000233422	0.444788848
cg05273205		1.50E-05	0.444782219
cg13582816		8.60E-06	0.444715755
cg04922681	TNFSF11;TNFSF11	0.000654035	0.444712888
cg11762839	USH1C;USH1C;USH1C;USH1C	7.23E-05	0.444632822
cg10453719	MARCH1	1.50E-05	0.44461812
cg25586069	KIAA1598;KIAA1598	2.58E-05	0.444596045
cg15301489	XKR6	0.000290372	0.444586652
cg20090108	GRM5;GRM5	1.96E-05	0.444549758
cg11722183	CPE	2.58E-05	0.444544644
cg11805669		0.000187194	0.444543679
cg09302895	GBX2	8.60E-06	0.444475431
cg14959580		1.11E-05	0.444464779
cg20686479	NAV2;NAV2;LOC100126784;NAV2	0.001061731	0.444444207
cg15427886		0.000148457	0.444428286
cg24789180		1.96E-05	0.444423834
cg05852008	EYA2;EYA2;EYA2;EYA2	7.23E-05	0.444411447
cg05687686	MIR1258;ZNF385B	1.50E-05	0.444393116
cg22160448	TLX3	5.59E-05	0.444338386
cg02098075	EFCAB1;EFCAB1;EFCAB1	0.000117986	0.444328645
cg21068269	SLC6A15;SLC6A15;SLC6A15	3.34E-05	0.444313118
cg17061244	LIN7A	1.11E-05	0.444311258
cg12395205	CIDEA;CIDEA	5.11E-05	0.444301597
cg04865491		1.96E-05	0.444288755
cg19719547	PLD5	4.34E-05	0.44425297
cg07664198	CHRM2;CHRM2;CHRM2;CHRM2;CHRM2;CHRI	8.60E-06	0.444249702
cg25616547		8.60E-06	0.444244173
cg26076724		9.24E-05	0.444217301
cg00802728	LHX5	4.34E-05	0.444199417
cg03778909	TBCD	1.96E-05	0.444183972
cg16689800	PPAPDC1A	0.000117986	0.444161009
cg05616999	RIPK4	8.60E-06	0.444157052
cg15661753		0.000290372	0.444111291

cg24519084	ACTC1;ACTC1	1.96E-05	0.4440741
cg26832294	TNIP3;TNIP3	0.00043997	0.444043032
cg09559047	XKR6	0.000537905	0.444016227
cg06245037	ALX4	1.96E-05	0.443982183
cg23920953	C14orf162	9.24E-05	0.443981813
cg17674725	LYPD1;LYPD1	4.34E-05	0.443963857
cg19472098	IQSEC3	2.58E-05	0.44396126
cg04773818	NRG1	2.58E-05	0.443931835
cg11596902		5.59E-05	0.443914516
cg09009536	EPHA5;EPHA5	8.60E-06	0.443913949
cg22057050	KIAA1310;KIAA1310	3.34E-05	0.443905614
cg17448336		1.50E-05	0.443882006
cg25478600	KCNQ1DN	5.78E-05	0.443857516
cg00168694	ETS2	3.34E-05	0.443813996
cg20426866	TACC2;TACC2;TACC2;TACC2	0.000187194	0.443746019
cg18991549	PCDHAC2;PCDHAC2;PCDHA7;PCDHA12;PCD	5.59E-05	0.443726014
cg24967811	PITPNM2	0.000357885	0.443713828
cg18780288	XPNPEP1;XPNPEP1;XPNPEP1	9.24E-05	0.443699243
cg00927554	USP44;USP44	8.60E-06	0.443666486
cg02538681	ANKRD22	4.34E-05	0.443656165
cg16325777		0.000117986	0.443649203
cg12083217	RAX	1.96E-05	0.443563987
cg18770350	ACTN2;ACTN2	9.24E-05	0.443562792
cg13753351	PSMB7	3.34E-05	0.443554037
cg04210284	SLC6A3	8.60E-06	0.443541347
cg13980609		1.96E-05	0.443537964
cg20557687	EDNRA;EDNRA;EDNRA;EDNRA;EDNRA	3.34E-05	0.443477967
cg04974290		2.58E-05	0.443465077
cg07961015		1.11E-05	0.443425266
cg05436231	CD164L2;CD164L2	7.23E-05	0.443423432
cg00549910	CTNNA2;LRRTM1;CTNNA2	2.58E-05	0.44341713
cg21896766	CSMD3;CSMD3	8.60E-06	0.443416573
cg13895867	GBX2	0.000233422	0.443392851
cg11260097		5.59E-05	0.443392489
cg24221947	NPAS2	5.59E-05	0.443377783
cg10552126	DPP6;DPP6;DPP6	8.60E-06	0.443372046
cg15108705	KCNK1	9.24E-05	0.443323112
cg06948294	STXBP6;STXBP6	4.34E-05	0.443311486
cg23664186	FOXA1	4.34E-05	0.4433042
cg03594078	PIWIL2;PIWIL2	0.000187194	0.443287469
cg20061155	RSPO2	1.50E-05	0.443283513
cg06132028	EYA4;EYA4;EYA4	0.00041966	0.443281674
cg20974724	FEZF1;FEZF1	3.34E-05	0.443265208
cg10362542	HRNBP3	0.000357885	0.44322625
cg16201674	SOX21	3.34E-05	0.443198134
cg20905516	GUCY1A2	0.000365373	0.443173359
cg01711160	FLRT2;FLRT2	8.60E-06	0.443161016
cg16334314	SNAP91;SNAP91	0.000187194	0.443158936
cg13051977	C1orf228	0.000117986	0.443120446
cg09184832		1.96E-05	0.443108477
cg14472384		1.96E-05	0.443105413
cg19788831	DBX2	1.50E-05	0.44309471
cg07783282	USP44;USP44	5.59E-05	0.443089168
cg02318784		4.34E-05	0.44307948
cg06631317	CCK	5.59E-05	0.443079093
cg20631014	SNAP91;SNAP91	1.50E-05	0.443067397
cg10204868	FAM123C;FAM123C;FAM123C;FAM123C	3.34E-05	0.443063861
cg25120290	FLRT2;FLRT2	3.21E-05	0.443025791
cg20334313		8.60E-06	0.443020449
cg15895690		8.60E-06	0.443002228

cg14730445	SLC5A8	2.58E-05	0.442962665
cg15484532	AJAP1;AJAP1	3.21E-05	0.442939949
cg02907374	CALCB	0.000357885	0.442934264
cg14304469	VSTM2A;VSTM2A	8.60E-06	0.442901764
cg25984344	DOCK1;FAM196A	3.34E-05	0.44289064
cg00565688	TP73	9.24E-05	0.442866823
cg20519121	NCRNA00164;MIR663B	1.11E-05	0.442852213
cg04236178	ESRRG	2.58E-05	0.442817764
cg11144753		2.58E-05	0.442802948
cg01908462		9.24E-05	0.442794066
cg13102079	CHRM2;CHRM2;CHRM2;CHRM2;CHRM2;CHR	8.60E-06	0.442778121
cg20895785	STEAP2;STEAP2	1.11E-05	0.442737034
cg13448605	RSPO1;RSPO1	5.59E-05	0.442728364
cg23724641	LYPD1;LYPD1	1.96E-05	0.442726148
cg07690181	EDIL3	0.000117986	0.442723381
cg06526693		0.000117986	0.442710914
cg15054725		5.59E-05	0.442702953
cg16729714	GALNT11	7.23E-05	0.442696494
cg23480730		8.60E-06	0.442668978
cg16048568	NR2F2;NR2F2;NR2F2;NR2F2;MIR1469;NR2F2	2.58E-05	0.442654574
cg02593932	GALNT13	8.60E-06	0.442637836
cg22159514	ELAVL3;ELAVL3	2.58E-05	0.442627741
cg19404444	SKI	7.23E-05	0.442625757
cg08528626	ST6GAL2;ST6GAL2;ST6GAL2	8.60E-06	0.442608202
cg23803337		1.11E-05	0.44260452
cg07136325	PRKD1	9.24E-05	0.442548934
cg27252395	KAAG1;KAAG1;DCDC2	1.96E-05	0.442506502
cg02132714	HOXB4	7.23E-05	0.442505731
cg17737314	KDM4A	7.23E-05	0.442460448
cg00698771		0.000187194	0.442458992
cg13481638		2.58E-05	0.442453515
cg19327614		1.50E-05	0.442453122
cg04650654		1.11E-05	0.442432636
cg13084458	INTU	4.34E-05	0.442423735
cg26678920	PRKCDBP	0.000117986	0.442416888
cg16865953		5.59E-05	0.44231856
cg10890302	TNXB	0.000187194	0.442308846
cg19647755		4.34E-05	0.442278353
cg07695835	KCNIP4;KCNIP4;KCNIP4;KCNIP4	4.34E-05	0.442275165
cg20464719	FCHO1;FCHO1;FCHO1;FCHO1	0.00043997	0.442274144
cg13816999	PARVA	8.60E-06	0.442251201
cg24791025		8.60E-06	0.442234148
cg02574509	FMN2;FMN2	5.59E-05	0.442198759
cg12147137	WASF3	3.34E-05	0.442155429
cg01766396	RFTN2	7.23E-05	0.442154985
cg14784497	LRRN1;LRRN1	0.000233422	0.442080043
cg09915835	UGT8	2.58E-05	0.442060805
cg03523785	FOXG1	8.60E-06	0.44205883
cg25756406		8.60E-06	0.442041808
cg17093212	MEOX2	1.11E-05	0.442034344
cg27425996	COBL	2.58E-05	0.442016061
cg10999312	NKX2-1;NKX2-1	0.00041966	0.441973117
cg05095591	ZIC1	8.60E-06	0.441928805
cg21322436	CNTNAP2	4.34E-05	0.441915193
cg18129621	NEFH	1.96E-05	0.441911689
cg02235562		0.000148457	0.441879189
cg13221924	KIAA0753	1.11E-05	0.441862638
cg09829319	GCM2	1.50E-05	0.441826212
cg01692842	ACAD8	3.34E-05	0.441815516
cg05422029		0.000148457	0.441802351

cg11344566	CNTNAP5;CNTNAP5	1.50E-05	0.441768744
cg25590826	CCDC33	2.58E-05	0.441752494
cg01257889	RPH3A;RPH3A	4.34E-05	0.441748084
cg24296452	C19orf41	8.60E-06	0.441730454
cg23633856	PRRX1;PRRX1	3.34E-05	0.441714683
cg03300589	PLCXD3	2.58E-05	0.441679495
cg09820378	PCDHA2;PCDHA1;PCDHA1;PCDHA6;PCDHA5	8.60E-06	0.44162096
cg17603689		8.60E-06	0.441615057
cg16680438		1.50E-05	0.441606723
cg14559356		4.34E-05	0.441591823
cg14617810	CDC14B;CDC14B	1.50E-05	0.441591246
cg05478631	T	1.96E-05	0.441590151
cg24308389	FEZF1;FEZF1	1.50E-05	0.441574334
cg24121424	MNX1;MNX1;MNX1	8.60E-06	0.44157384
cg16542478		4.34E-05	0.441570807
cg20107506		4.34E-05	0.441559752
cg02152120	SPAG17;SPAG17	2.58E-05	0.44155256
cg02333852	THRB;THRB;THRB;THRB;THRB;THRB	0.000357885	0.441528166
cg02220965	MYST1;MYST1	0.000117986	0.441492554
cg10453420		1.96E-05	0.441488615
cg24067911	ATXN1;ATXN1	4.34E-05	0.441456434
cg27177554	C1QL3	1.50E-05	0.441427936
cg18503912	FXYD1;FXYD1	1.96E-05	0.441420446
cg05783139	BOLL;BOLL	8.60E-06	0.441411883
cg24124029	C20orf103	0.000233422	0.441404333
cg07917901		5.59E-05	0.441403503
cg08263589		0.000117986	0.441400056
cg08734740	ISL1;ISL1	1.50E-05	0.441386248
cg06776999	RNF180;RNF180	1.50E-05	0.441378057
cg19302722		9.24E-05	0.441333505
cg06907391	GABRA1;GABRA1;GABRA1;GABRA1;GABRA1	1.50E-05	0.441330849
cg14463412		2.58E-05	0.441316499
cg17465305	OCLN	1.11E-05	0.441285106
cg24789447	EDNRA;EDNRA;EDNRA	0.000187194	0.441276314
cg11157253		8.60E-06	0.441266122
cg21057907	SFTA3	1.11E-05	0.441232603
cg07658508	SLC26A1	0.000187194	0.441215642
cg11941546	CLDN7	0.000117986	0.441215637
cg10718056	TRIM27	0.000122184	0.441211352
cg07577799	SLC6A1	3.34E-05	0.441203575
cg17370163	RNF180;RNF180	1.11E-05	0.441203228
cg27358097	GALNTL4	4.34E-05	0.441170966
cg23806894		3.34E-05	0.44116917
cg25605731	CALR	2.58E-05	0.44116016
cg01535733	RGS17;RGS17	9.24E-05	0.441126399
cg03609960	ANKS1B;ANKS1B;ANKS1B	8.60E-06	0.441114835
cg06697536	HOXD1;HOXD1	5.59E-05	0.441095202
cg27112565	NRXN1;NRXN1;NRXN1;NRXN1	9.24E-05	0.441062017
cg20605886		8.60E-06	0.441053395
cg09452568	ESM1;ESM1	7.23E-05	0.441028104
cg11176990	LOC375196;LOC100271715	8.60E-06	0.441014211
cg01962937	HTRA1;HTRA1	0.000148457	0.441005193
cg27176138	HS6ST3	1.50E-05	0.440998394
cg22459755		9.24E-05	0.440997586
cg01997272		0.000117986	0.440990982
cg04050867	RSPO2;RSPO2	1.96E-05	0.440987303
cg21864259	ERC2	1.11E-05	0.440967896
cg23606718	FAM123C;FAM123C;FAM123C;FAM123C;FAM1	4.34E-05	0.440950737
cg01498609		1.50E-05	0.440946339
cg13111733	HIST3H2BB;HIST3H2A;HIST3H2BB	1.11E-05	0.440946017

cg11353250	COL12A1;COL12A1	1.96E-05	0.440938035
cg04491367		3.34E-05	0.440929831
cg06270401	DYRK4	1.96E-05	0.440902109
cg15148933		2.58E-05	0.440890132
cg24421216	MEIS1	0.000357885	0.440883108
cg01002253	NPY5R	2.58E-05	0.440862805
cg27218767	TRPC1	0.000654035	0.440859831
cg01761758	TIMP2	4.34E-05	0.440848862
cg20866694		1.11E-05	0.440839231
cg25375340	MCOLN2	0.000148457	0.440828534
cg03881775	ZIC4;ZIC4;ZIC4;ZIC4;ZIC4	1.96E-05	0.440822942
cg23367107		4.34E-05	0.440801985
cg12762763		8.60E-06	0.440797505
cg19717347	SLC15A4	1.96E-05	0.440795512
cg17918501	FNDC4	3.34E-05	0.440780464
cg26502671	CLDN3	1.11E-05	0.44076835
cg10358981		3.34E-05	0.440710775
cg02067712		1.96E-05	0.440696007
cg18347642	KCNIP4	1.11E-05	0.440689069
cg26472133		2.58E-05	0.440676611
cg04244170	TOMM70A	0.000148457	0.440582576
cg01384488	NKX6-2	8.60E-06	0.440582174
cg17318297	RGS7BP	1.50E-05	0.440575876
cg19697475	HCN1	3.34E-05	0.440559448
cg26783057	VAX1	3.34E-05	0.440533712
cg12196294	TMCC1;TMCC1	8.60E-06	0.440528795
cg00346208	VWA5B1	0.00043997	0.440520662
cg13452258	EPHA10	7.23E-05	0.440518665
cg17189465	ROBO2	7.23E-05	0.440505339
cg27565555	BMP7	0.000357885	0.44049154
cg20463862	RTN1;RTN1;RTN1;RTN1	1.96E-05	0.440491443
cg23244559	CXCL2	0.000357885	0.440476131
cg11052780	TRPA1	9.24E-05	0.440431646
cg07934812	TCERG1L	0.000705485	0.440419135
cg09234518	NEFM;NEFM	5.59E-05	0.440408342
cg03354992		1.11E-05	0.440401555
cg24633978	HOXD11	8.60E-06	0.440401325
cg21038156	VIPR2	0.000654035	0.440389575
cg13796804	EPHA5;EPHA5	2.58E-05	0.440369521
cg00458681	DGCR10	9.24E-05	0.440352772
cg04552418		0.000148457	0.440343913
cg03503516	C22orf45;C22orf45;UPB1	8.60E-06	0.44032957
cg23514016	BHMT	2.58E-05	0.440325994
cg12600174	HOXA9	1.96E-05	0.440324643
cg23509344		1.50E-05	0.440321081
cg22454660	GPR177;GPR177	0.000148457	0.44031281
cg11398452	VAX1;VAX1	2.58E-05	0.440300428
cg08795964		1.11E-05	0.440267698
cg26146027	THRB;THRB;THRB	0.000117986	0.440251478
cg11294835	CELSR3	1.96E-05	0.440244867
cg02155398		7.23E-05	0.440240377
cg06553312	MYH14;MYH14;MYH14	8.60E-06	0.440212793
cg22934295	CCK;CCK	3.34E-05	0.440174196
cg11124364	TMEM196	8.60E-06	0.440156457
cg05924652	GLP1R	9.24E-05	0.440118049
cg12757011	TBR1	1.11E-05	0.440114415
cg05936004	ATP2C1;ATP2C1;ATP2C1;ATP2C1	1.50E-05	0.440111581
cg17555373		4.34E-05	0.440098965
cg12157761	UACA	1.96E-05	0.440090184
cg21632158		8.60E-06	0.440073579

cg21743623	ATP2C1;ATP2C1;ATP2C1;ATP2C1	3.34E-05	0.440054319
cg17315500		9.24E-05	0.440047126
cg15207742	RIMS4	8.60E-06	0.440033181
cg21114773	CIDEA;CIDEA	5.59E-05	0.440025144
cg00650809	ANO8	1.96E-05	0.440024105
cg02637318	ADAM32;ADAM32	3.34E-05	0.439987625
cg03731974		2.58E-05	0.439979282
cg14308313		0.000357885	0.439977763
cg08110693	PXT1	3.34E-05	0.439926605
cg18542177	ARL4A;ARL4A;ARL4A	0.000791389	0.439918238
cg09683824	FAM163A	2.58E-05	0.439917539
cg11991058		1.50E-05	0.439901705
cg00840310		5.59E-05	0.439888836
cg01126567	GPR139;GPR139	4.34E-05	0.439842643
cg12483340	TMEM87A	3.34E-05	0.439838429
cg24255371	LOC401463	0.001147425	0.439837427
cg09515953	PPP1R14A	0.000187194	0.439836612
cg23676588	GABRA2;GABRA2	8.60E-06	0.439808322
cg22670329	CXCL6	2.58E-05	0.439789486
cg10589443	SCRN1;SCRN1;SCRN1;SCRN1	5.59E-05	0.439786518
cg12231969	EN1;EN1	1.11E-05	0.439768003
cg05128922		1.11E-05	0.439767534
cg05841659		0.000148457	0.439724284
cg16832407	TRPM3	1.11E-05	0.439714314
cg09537620	PAX6;PAX6;PAX6	5.59E-05	0.43964819
cg03602280	FAM38B	7.23E-05	0.439632103
cg20609302		9.24E-05	0.439622908
cg00247557	LOC642597;LOC642597	8.60E-06	0.439621132
cg01434649	TP73;WDR8	4.34E-05	0.439616865
cg19582538		8.60E-06	0.439601085
cg02172312	CDH8	3.34E-05	0.439562253
cg18018027		2.58E-05	0.439561512
cg01138981	KIF6	0.000290372	0.439555771
cg16378352	HOOK1	8.60E-06	0.439551305
cg05892568		8.60E-06	0.439521111
cg14473568	ROBO2	2.58E-05	0.439498719
cg17922359		5.59E-05	0.439486661
cg01520867	MIR203	4.34E-05	0.439484294
cg14278853		9.24E-05	0.439464039
cg18252039		1.11E-05	0.439446106
cg23077820	PAX3;PAX3;PAX3;PAX3;PAX3;PAX3	2.58E-05	0.439446075
cg14757228	PRRT1	2.80E-05	0.439426546
cg11931762		7.23E-05	0.43939103
cg10036918	LHFPL3;LHFPL3	3.34E-05	0.439377368
cg26385074	C7orf52	9.24E-05	0.439366449
cg18110483	THBS4	1.96E-05	0.43934893
cg11824172	HOOK1	8.60E-06	0.439281802
cg22117805	PRR5L;PRR5L;PRR5L;PRR5L	0.000654035	0.43927751
cg13237033	SIX1	1.50E-05	0.439244837
cg02009256	GLS2	0.000205664	0.439240848
cg02860282		7.23E-05	0.439231032
cg02742906		3.34E-05	0.439230223
cg23658477		0.000187194	0.439225258
cg10721149	SCGN	1.11E-05	0.439217184
cg15744359	LRRC67	0.000148457	0.439194491
cg26898932	CCDC55	2.58E-05	0.439155162
cg11985220		3.34E-05	0.439135653
cg22994281	COL4A3;COL4A3;COL4A3;COL4A3;COL4A4	1.50E-05	0.439132434
cg24243314	PCDH20	4.34E-05	0.439118794
cg24879335	TF;TF	5.59E-05	0.439109422

cg05678749	GABRG3	1.50E-05	0.439105254
cg19161112	PTF1A	8.60E-06	0.43910432
cg14028400		8.60E-06	0.439077827
cg18609078		9.24E-05	0.439061392
cg15027907		0.00043997	0.439050612
cg23619357		1.50E-05	0.439026319
cg24399122	COL9A1;COL9A1;COL9A1	1.96E-05	0.438993827
cg06458358	ARHGEF10;ARHGEF10	5.59E-05	0.438991436
cg23181580		2.58E-05	0.438983238
cg13024709	ARHGEF4;ARHGEF4	7.23E-05	0.438919524
cg11051055	XKR6	0.000148457	0.438863483
cg23538901		1.50E-05	0.438841229
cg05068452	UGT8	1.96E-05	0.438829024
cg13526007	LRFN5	0.000187194	0.438799883
cg18438853	NHLH2;NHLH2	2.58E-05	0.438794277
cg19777067		9.24E-05	0.438789203
cg06984025		1.11E-05	0.438774746
cg03506489	KCNA4	0.000117986	0.438768549
cg18082337	ZIC4;ZIC4;ZIC4	2.58E-05	0.438755511
cg17305181	CRH;CRH	3.34E-05	0.438744369
cg02806156		2.58E-05	0.438710629
cg13184872	PNPLA3;PNPLA3	9.24E-05	0.438691922
cg09969277	LOC254559	1.96E-05	0.438684778
cg19651694	PHOX2A;PHOX2A	1.11E-05	0.438684131
cg12002745		3.34E-05	0.438678314
cg26163458		1.11E-05	0.438649491
cg01486814		8.60E-06	0.438647669
cg22746058	PTF1A	1.50E-05	0.438640163
cg10372476	ACTN2	8.60E-06	0.438630113
cg08645720	LCA5;LCA5	0.000187194	0.438603548
cg06482428	KCNIP4	4.34E-05	0.438576853
cg06305891		0.000117986	0.438548129
cg18173235		8.60E-06	0.438517982
cg08928882	LOC100192378;ZFHX4	5.59E-05	0.438508431
cg19908812	NPY1R	8.60E-06	0.438500107
cg20403557	LVRN	3.34E-05	0.438490943
cg07909265	ITGAV;ITGAV	9.24E-05	0.438464541
cg26928972	CSTA	2.58E-05	0.43845357
cg19809039	C11orf70	8.60E-06	0.438444826
cg09804380	PDPN;PDPN;PDPN;PDPN	2.58E-05	0.438421285
cg09231862	FOXG1	1.96E-05	0.43841098
cg17018946		7.23E-05	0.438405696
cg11359210	RGS7BP	3.34E-05	0.438387372
cg09686317	PRKAA2	0.000148457	0.438326749
cg23861715	CDC14B;CDC14B	1.11E-05	0.438325024
cg03290530		9.24E-05	0.438284914
cg23298047		0.000205664	0.43827661
cg23288973	TRPC7;TRPC7;TRPC7;TRPC7;TRPC7;TRPC7	8.60E-06	0.438247277
cg22809871	FGF14	5.59E-05	0.438242907
cg23629998	TMEM229A	1.96E-05	0.438211686
cg14276379	C9orf3	0.000117986	0.438207464
cg09683350		1.50E-05	0.438185896
cg23378033	MSRB3;MSRB3	0.000537905	0.438184188
cg12409525	DSCAM	8.60E-06	0.438183393
cg02986266		2.58E-05	0.438144642
cg18782604	SIM1	0.000791389	0.438106468
cg06833978	FAM155A	1.11E-05	0.438065425
cg09214254	SMOC2;SMOC2	0.000233422	0.438060317
cg10877086		1.11E-05	0.438043036
cg02783103	ZNF365;ZNF365;ZNF365	4.34E-05	0.438040928

cg05573761	SGMS2	7.23E-05	0.438031201
cg22029189		7.23E-05	0.438022944
cg18802021	MKX	3.34E-05	0.438015245
cg17879189	PRDM13	8.60E-06	0.437980618
cg24693225		3.34E-05	0.437940165
cg12028548	TCF15	8.60E-06	0.437928866
cg02856132		8.60E-06	0.437927876
cg18805457	TCERG1;TCERG1	2.58E-05	0.437922293
cg14369938		5.59E-05	0.437908764
cg14015441	DPYS	4.34E-05	0.437907963
cg16199747		2.58E-05	0.437892939
cg12126706	C1orf92	7.23E-05	0.437876086
cg15644174	TMEM171;TMEM171;TMEM171;TMEM171	2.58E-05	0.437855068
cg24906819	PRAGMIN	8.60E-06	0.437827912
cg17273911		1.11E-05	0.437806704
cg00181834		2.58E-05	0.437805768
cg05583020		7.23E-05	0.437780686
cg00157668		0.000233422	0.437770061
cg16101739	PRRX1;PRRX1	1.50E-05	0.437768676
cg22725685		7.23E-05	0.437743221
cg06005892	OSBPL5;OSBPL5;OSBPL5	3.34E-05	0.437730018
cg24524352	ANKRD20B	2.58E-05	0.437709177
cg00529958	ZIC5	3.34E-05	0.437700346
cg13555101	SLC1A1	3.34E-05	0.437689297
cg15133301	SNED1	0.000122184	0.437688407
cg26478599	LOC285954;LOC285954	8.60E-06	0.437658855
cg15125684	NLRP14;ZNF214;NLRP14	5.59E-05	0.43765203
cg20536983	DLC1	0.001147425	0.437642416
cg24132481		8.60E-06	0.437617867
cg25793931		8.60E-06	0.437586283
cg14367229	GSX1	8.60E-06	0.437531169
cg17007640	C14orf23;C14orf23	8.60E-06	0.437525376
cg06128198	DOCK1	0.000290372	0.437516411
cg00637687	PCSK1;PCSK1	5.59E-05	0.437493055
cg09297288	GBX2	1.50E-05	0.437484623
cg06522288	LOC100192378	1.11E-05	0.437463675
cg07978472	TMEM155;LOC100192379	3.34E-05	0.437457368
cg27472295	TPBG;TPBG	0.001639597	0.437433018
cg08325191	C10orf93	0.000117986	0.437406009
cg04999479	ID4	0.000233422	0.437385832
cg26399778	SH3RF2;SH3RF2	2.58E-05	0.437367939
cg02914422	PDE1C	0.000117986	0.43736478
cg15488722	FREM2	8.60E-06	0.437351013
cg26277754		8.60E-06	0.437350051
cg19867914	ARHGAP15	9.24E-05	0.437345838
cg14689623	NELL1;NELL1;NELL1;NELL1	0.001147425	0.437343388
cg23210118	TMEM20;TMEM20	8.60E-06	0.437339432
cg20487710	PTF1A	1.96E-05	0.437338436
cg18117347	SHANK1	1.11E-05	0.437330751
cg16411256	EPHX2	8.60E-06	0.437321008
cg09740671	ADCY2	9.24E-05	0.437316139
cg18422875	KCNN2	1.96E-05	0.437315238
cg04685534	NELL2;NELL2;NELL2;NELL2;NELL2	0.000148457	0.437314113
cg09798888		2.58E-05	0.437289788
cg10649903	PCDHA2;PCDHA1;PCDHA3;PCDHA3;PCDHA1	2.58E-05	0.437279074
cg19275632	TRPC4;TRPC4;TRPC4;TRPC4;TRPC4;TRPC4	0.000148457	0.437274829
cg01630690		1.50E-05	0.437272219
cg01851450	RAB32	9.24E-05	0.437264843
cg02490516	FAM150A	1.18E-05	0.437258144
cg13488201	ADAM12;ADAM12	1.96E-05	0.437247788

cg24312537	HTRA4;PLEKHA2	0.000117986	0.437237426
cg27253807		7.23E-05	0.437208932
cg21158087		1.96E-05	0.437200558
cg14362758	LHX3;LHX3	0.000117986	0.437151215
cg23081580	STEAP2;STEAP2;STEAP2	7.23E-05	0.437144906
cg10408731	CCT6P1	1.11E-05	0.437141744
cg23230584	PON3	9.24E-05	0.437133668
cg04919274	GPR149	8.60E-06	0.437114841
cg22757399	PNMA2	0.000951187	0.437103814
cg25174438		8.60E-06	0.437033429
cg19209689	BRUNOL4;BRUNOL4;BRUNOL4;BRUNOL4	4.34E-05	0.437027799
cg17384380		2.58E-05	0.436936309
cg14396421		2.58E-05	0.436931468
cg12892004	RFX6	8.60E-06	0.436852687
cg20869673	SEMA6D;SEMA6D;SEMA6D;SEMA6D;SEMA6D	0.000187194	0.436833427
cg17087331	PPAPDC1A	4.34E-05	0.436803062
cg17121747	MFSD7	5.59E-05	0.436800168
cg10603275	CHL1	0.001948248	0.436782947
cg11810743	DACT2	4.34E-05	0.436758791
cg01942558	TNFAIP6	5.59E-05	0.436748231
cg13826452		1.96E-05	0.436737462
cg14270434		2.58E-05	0.436730206
cg03107235	ATP8A2	0.002304201	0.436727444
cg03110368	ARHGAP20	0.000290372	0.436711643
cg21410991	ISL1	1.50E-05	0.436701813
cg00109302		1.50E-05	0.436688791
cg25724283	DCLK1	1.11E-05	0.436680602
cg14060111	ISM1	0.002304201	0.436680052
cg06436185	PRKAG2;PRKAG2	2.58E-05	0.436678818
cg11670605	PLCXD3	1.50E-05	0.436672795
cg02770745	STIM1	2.58E-05	0.436626722
cg08444220	NKX6-2	8.60E-06	0.436556548
cg11942956	EYA4;EYA4;EYA4	9.24E-05	0.43655418
cg02709321	TOX3;TOX3	1.11E-05	0.43652597
cg20664238	NTRK3;NTRK3;NTRK3	2.58E-05	0.436523598
cg22674699	HOXD9	0.000290372	0.436520404
cg09077752		3.34E-05	0.436485055
cg13849378	RGS7;RGS7	3.34E-05	0.436413093
cg11552072	NCRNA00164;MIR663B	1.11E-05	0.436386591
cg08136772	PCDH8;PCDH8	1.96E-05	0.436369269
cg18689958	RXFP3	0.000290372	0.436347795
cg11817899	SORCS1;SORCS1	3.34E-05	0.436332631
cg02496256	CDH6;CDH6	0.000117986	0.436330403
cg09987596	FAM123C;FAM123C;FAM123C;FAM123C	9.24E-05	0.436293639
cg05314350	TRIM27	1.11E-05	0.436293148
cg07016258	BNC1	4.34E-05	0.436290195
cg14206172	FBXO17	8.60E-06	0.436283765
cg10297792		1.11E-05	0.436276382
cg12367621	MORN4;MORN4	7.21E-05	0.436243922
cg23328622	SNAP25;SNAP25	5.59E-05	0.436228819
cg13434842	GATA4	3.34E-05	0.43621779
cg00471371	ZNF516	1.50E-05	0.436199728
cg18437792		5.59E-05	0.436193844
cg15244049	GCM2	8.60E-06	0.436133045
cg03476195	ANK2;ANK2;ANK2	4.34E-05	0.436099692
cg24804768	NINJ2	0.000187194	0.436088089
cg00907288	SRGAP3;SRGAP3	0.001948248	0.436087207
cg02466321	ANKRD34B;ANKRD34B	4.34E-05	0.436063084
cg17187287	UGT8	0.000262362	0.436061092
cg26785954	TMEM26	1.96E-05	0.436057985

cg26923490	KCNA7	7.23E-05	0.436046867
cg14617041	TMEM171;TMEM171	0.000955793	0.436044752
cg08918749	LPL	3.34E-05	0.436043293
cg03658236	STAB2	1.50E-05	0.436036053
cg25134515	WWC2;C4orf38	0.000187194	0.436005003
cg20411843		2.58E-05	0.435997922
cg12664209	ISM1	0.000705485	0.435973342
cg22820233	FCAR;FCAR;FCAR;FCAR;FCAR;FCAR;FCAR;F	0.000148457	0.435965773
cg02606808	MAP1B	7.23E-05	0.435956064
cg04043742	PLA2G7;PLA2G7;PLA2G7	1.96E-05	0.43592881
cg26293546	PROX1	1.96E-05	0.435862746
cg14471364		1.96E-05	0.435857853
cg14034197	GREB1L	8.60E-06	0.435832333
cg09571972	GMDS	4.34E-05	0.435803111
cg02490989	MTMR7	1.11E-05	0.435802495
cg00579520	TMEM132C	3.34E-05	0.435769465
cg01689438		0.000148457	0.435755494
cg11611644		2.58E-05	0.435721432
cg17275700	FAM190A	1.50E-05	0.435662452
cg11651237	GABRG3;GABRG3	4.34E-05	0.435647279
cg12249728	SH3BGRL2	1.96E-05	0.435618566
cg17252960	ID4	0.000233422	0.435595548
cg25050951	TJP1;TJP1	1.50E-05	0.435578608
cg02878244	DBX1	2.58E-05	0.435537646
cg12927990	GRP;GRP;GRP	0.000236512	0.435497542
cg02346970	PDZD2	9.24E-05	0.435474066
cg11153529	OGDHL;OGDHL;OGDHL	5.59E-05	0.435439467
cg14855519	EBF2	0.000117986	0.435415416
cg06820388		8.60E-06	0.435406062
cg09338032		1.96E-05	0.435395561
cg20099104		0.000955793	0.43538738
cg20673721	KSR1	5.59E-05	0.435384933
cg05221167	ZNF560	1.11E-05	0.43536884
cg14576062	DPY19L2P4	8.60E-06	0.435357017
cg07333924	MATN2;MATN2	9.24E-05	0.43534631
cg18726691	RHCG	3.34E-05	0.435341979
cg12594001		8.60E-06	0.435340285
cg03120289	LPPR5;LPPR5	1.11E-05	0.435336761
cg20945085	C2orf65	1.96E-05	0.435318921
cg23407655	ABR;ABR;ABR	0.000148457	0.435317019
cg22342329		5.59E-05	0.435314062
cg21684012	SIM1	3.34E-05	0.435293752
cg14423557	BRUNOL4;BRUNOL4;BRUNOL4;BRUNOL4	8.60E-06	0.435265368
cg11071231		4.34E-05	0.435251231
cg00073780	PKIB;PKIB;PKIB;PKIB;PKIB	0.000148457	0.435249358
cg13524919		9.24E-05	0.435245294
cg07950000	GRIK1;GRIK1	1.50E-05	0.435226856
cg26150514		3.34E-05	0.435213643
cg09877744	MAPK8IP2;MAPK8IP2	8.60E-06	0.43521245
cg06682039		1.96E-05	0.435163612
cg22131234	VSTM2A	9.24E-05	0.435146137
cg05700079	ZIC1	1.50E-05	0.435139826
cg18372607	PAX6;PAX6;PAX6	4.34E-05	0.435136848
cg11293190	PLK5P	8.60E-06	0.435122681
cg26678978	CCKBR	8.60E-06	0.435116963
cg00452257	SELV	8.60E-06	0.435068935
cg14400498	FLRT2	1.96E-05	0.435037709
cg22694191	HAPLN4;HAPLN4	1.96E-05	0.435035763
cg02044219	LGI2	0.000290372	0.435026606
cg00902153	LPP;LPP;LPP	0.000537905	0.435014962

cg24345062	FLJ32063	7.23E-05	0.434995326
cg22231908	FAM83H;FAM83H	4.34E-05	0.43499299
cg00984146	EGFLAM	9.24E-05	0.43498473
cg25408950	NEK7	0.000187194	0.434982249
cg16256414	GULP1	1.96E-05	0.434980584
cg12432236	PCDH17	3.34E-05	0.43495456
cg16081281		1.50E-05	0.434943586
cg01376826	ZIC1	1.11E-05	0.434922745
cg14622879		5.59E-05	0.434815764
cg20011562	PHACTR1	8.60E-06	0.434808925
cg22666015	INPP5D;INPP5D	0.000233422	0.434808673
cg26434048	IGFBP3;IGFBP3	1.11E-05	0.434765209
cg16909783		1.11E-05	0.434759823
cg04217706		1.50E-05	0.434750922
cg15034393		7.23E-05	0.434747333
cg19628148	CCDC37	1.96E-05	0.434721516
cg14095438		5.59E-05	0.434606112
cg09106903	ESRRG	2.58E-05	0.434547266
cg11201710	FGF12	8.60E-06	0.434544902
cg10132208	ZSCAN1	1.50E-05	0.434544361
cg23680282	LRRIQ1;TSPAN19;LRRIQ1	0.000148457	0.434539066
cg19171596	STL	0.00043997	0.434537228
cg15182360	PCDH20	4.34E-05	0.434532211
cg00335124	PDE4D;PDE4D;PDE4D	5.59E-05	0.434519617
cg01781617		0.000233422	0.434489434
cg18375860	RYR2	1.50E-05	0.434453077
cg04940570	TEAD1	5.11E-05	0.434451399
cg08425796		3.34E-05	0.434445311
cg03333330	TFPI2	8.60E-06	0.434441011
cg23699196	NTM;NTM;NTM;NTM	2.58E-05	0.434437539
cg26831566		1.11E-05	0.434426551
cg25130993	USH1C;USH1C	8.60E-06	0.43437004
cg11389756	TRIM27	3.34E-05	0.434331643
cg08095196	GJD2	1.50E-05	0.434300981
cg13234643	CTSL2;CTSL2	5.59E-05	0.434286021
cg04529066	ACADL;ACADL	3.34E-05	0.434272198
cg01777121	WNT5A	3.34E-05	0.434262224
cg23920642		2.58E-05	0.434254113
cg15020425	FGF3;FGF3	8.60E-06	0.43422276
cg08409113	WNK4	7.23E-05	0.434189154
cg01057573	TNFAIP8	3.34E-05	0.434166507
cg03112087	RNF150	3.34E-05	0.434147204
cg07249459	FOXC2	7.23E-05	0.434129235
cg04154138	ZNF677	0.00043997	0.434127898
cg01007828	CDH8	8.60E-06	0.434088186
cg26316023	CADPS2;CADPS2;CADPS2	0.000117986	0.43406794
cg07861456	ADAT1	0.000117986	0.434038572
cg10242602	ZFP42	0.000117986	0.434036217
cg11416384	KIAA1239	0.000187194	0.434017
cg16640633	TPBG;TPBG	0.001948248	0.434003913
cg10680051	ROBO3	0.000117986	0.433943832
cg20264481		8.60E-06	0.433935648
cg15418499	IL18	9.24E-05	0.43392791
cg16346032		3.34E-05	0.433912444
cg21609640	ILDR2	8.60E-06	0.433860934
cg15929078	TAPBP;TAPBP;RGL2;RGL2	0.000117986	0.433851055
cg04822518	TMTC1	8.60E-06	0.43382696
cg23558842	CALCB;CALCB	0.001147425	0.433815686
cg23130097	MAL2	5.59E-05	0.433805255
cg17864046		8.60E-06	0.433804322

cg10537220	ZNF365;ZNF365;ZNF365	4.34E-05	0.433790874
cg09921610	UCHL1	4.34E-05	0.433754087
cg17288397		0.000357885	0.433728516
cg02873885		4.34E-05	0.433724885
cg16934178	TFPI2	0.000148457	0.433708686
cg10884788	IGSF9B	1.50E-05	0.433660584
cg13536757	ZNF804A;ZNF804A	1.50E-05	0.433627714
cg04621578	IGSF9B	4.34E-05	0.43360992
cg14959729	T	8.60E-06	0.433589315
cg10119075	ITIH5;ITIH5	0.000233422	0.433579309
cg08000065	UNC5D	1.96E-05	0.433577116
cg09560082		1.96E-05	0.433573838
cg10109500	GHSR;GHSR	4.34E-05	0.433557419
cg26867890	KIF6	0.000955793	0.433528802
cg07116393	MUL1	9.24E-05	0.433519453
cg23819411	MCF2L2	5.59E-05	0.433512745
cg09679234	GABRA2;GABRA2	4.34E-05	0.433501196
cg17319007	FBLN5	0.000117986	0.43349336
cg18378453		0.000233422	0.433481181
cg01688536		0.000187194	0.433479626
cg15613567	GPR123;GPR123	2.58E-05	0.433417253
cg07847863	PCDH8;PCDH8	3.34E-05	0.433414822
cg08003353		0.002714607	0.433412095
cg18328894	CPLX2	1.50E-05	0.433398814
cg03052078	STXBP5;STXBP5	0.000148457	0.433342608
cg05387146	ME1	0.000117986	0.433339911
cg25840237		1.50E-05	0.433333377
cg00329270	KY	7.23E-05	0.433326664
cg21574822		3.34E-05	0.433277556
cg05025071	EMR1	0.000187194	0.433233791
cg23676682	GRIA4;GRIA4;GRIA4;GRIA4	4.34E-05	0.433215419
cg16758813		8.60E-06	0.433208107
cg02681173	LOC100190940	3.34E-05	0.433170068
cg10396546	ZSWIM2	3.34E-05	0.433141487
cg18538958	TACR3	1.50E-05	0.433138248
cg10519368	PCDHA6;PCDHA2;PCDHA1;PCDHA9;PCDHA7	0.000187194	0.433118205
cg06613392		1.50E-05	0.433105782
cg05646686		1.50E-05	0.433098267
cg06611922	VAX1;VAX1	2.58E-05	0.433078211
cg08764162	ZNF438;ZNF438;ZNF438;ZNF438;ZNF438;ZNF	9.24E-05	0.43304183
cg01479396	SHBG;SAT2	1.96E-05	0.433008691
cg16242629		0.000187194	0.432988063
cg11375458		3.34E-05	0.432956683
cg16703762	OTP	1.11E-05	0.432940259
cg11148130	PDZRN3;PDZRN3	8.60E-06	0.432932847
cg24704211	CDC16;CDC16	0.001147425	0.432919479
cg16954341	SCGN	3.34E-05	0.432907927
cg01994307		4.34E-05	0.432896889
cg16800165	FLRT2	8.60E-06	0.432888358
cg14722140	C6orf141	0.000117986	0.432867851
cg15550398	SNED1	3.34E-05	0.43286686
cg13577744	STEAP2;STEAP2	4.34E-05	0.432836252
cg05593641		3.34E-05	0.432827015
cg24755163		8.60E-06	0.432821553
cg12210890	KCNV1	1.11E-05	0.432813359
cg25099490		1.96E-05	0.432807557
cg24799921	C10orf41;C10orf41	1.50E-05	0.432783787
cg15585794	NTM;NTM;NTM;NTM	8.60E-06	0.432769395
cg12591668		0.000357885	0.432739717
cg27058486	CHST8;CHST8	4.34E-05	0.432721508

cg06584494	VSTM2A	5.59E-05	0.432713496
cg20563910	MSX2	1.50E-05	0.432709373
cg23449696	ZIC1	1.50E-05	0.432706405
cg26163699	LRRN1	4.34E-05	0.432706149
cg05492071		2.58E-05	0.432700564
cg19852958	NKX3-2	0.000290372	0.432684506
cg21706229		1.50E-05	0.432681029
cg04572930	FOXK1	0.000526175	0.432661185
cg21746997	BMPR1B	0.004357816	0.432638507
cg16304950	EPB41L3;EPB41L3	9.24E-05	0.43259226
cg27472905	SNED1	9.24E-05	0.432581275
cg25798792	EPHA5;EPHA5	1.11E-05	0.432570148
cg02461363	TMEM26	8.60E-06	0.432566137
cg07078841	CYP1B1;CYP1B1	1.50E-05	0.43253454
cg01104724		7.23E-05	0.432526237
cg23781495	PAPPA	0.000117986	0.432524624
cg00027083	EPB41L3	0.000122184	0.432522629
cg03859162	SLC15A1;SLC15A1	3.34E-05	0.432517664
cg12162100	CPNE3	9.24E-05	0.43250465
cg27552955	TRPC1	0.000357885	0.432483202
cg06541501	EBF2	2.58E-05	0.432475922
cg02612963	ZNF483;ZNF483	8.60E-06	0.432457
cg12929487		2.58E-05	0.432439555
cg11192416		8.60E-06	0.43241954
cg16026922	UCHL1	8.60E-06	0.432417652
cg06003656		2.58E-05	0.432394112
cg20544852	TRIM27	1.50E-05	0.432372651
cg08998950	ITPR1;ITPR1;ITPR1	0.000357885	0.432340302
cg22488970	CAMTA1	0.000290372	0.432283047
cg05656934	PRKAA2	0.000117986	0.432265343
cg22873539		8.60E-06	0.432241737
cg27627524	ASAP1	7.23E-05	0.432225684
cg21117476	CYP2S1	0.000148457	0.432213892
cg00769843	OTUB1;OTUB1	0.000148457	0.432212522
cg02721176	C10orf96	8.60E-06	0.432136418
cg24319902	SFRP1	1.50E-05	0.432110103
cg13755795	SALL1;SALL1	5.59E-05	0.432072779
cg21180286		0.000357885	0.432065042
cg05129325	KCNK2;KCNK2;KCNK2	1.50E-05	0.432062491
cg05697849	ELAVL4;ELAVL4	8.60E-06	0.432053987
cg03462380	GABRA5;GABRA5	2.58E-05	0.432013723
cg02499494	LRIG3;LRIG3;LRIG3	4.34E-05	0.432013582
cg20139706		7.23E-05	0.431931852
cg10353539	TTC12	1.50E-05	0.431907603
cg08376828		0.005897668	0.43190681
cg08627720		3.34E-05	0.431898137
cg24301620	GRIK2;GRIK2;GRIK2;GRIK2;GRIK2;GRIK2	1.96E-05	0.431873867
cg01990910	SNX29	0.000262362	0.431858799
cg04168670	CNTNAP5	1.96E-05	0.431829199
cg06564232	TFAP2B	2.58E-05	0.431815277
cg22259831	B4GALNT2;B4GALNT2;B4GALNT2	1.96E-05	0.431809214
cg11690666	NARF;NARF;NARF;NARF	2.58E-05	0.431781554
cg09833789	PCDP1	5.59E-05	0.431754893
cg14754787	LHX1	0.000290372	0.43175375
cg00332937		0.000117986	0.431722172
cg24500832	HOXC9	5.59E-05	0.431714439
cg21938436	ADAMTS9	1.50E-05	0.431712453
cg08097281		0.000654035	0.431699153
cg26055770	PDZRN3;PDZRN3	8.60E-06	0.431695409
cg03711485	NKX2-3	0.000187194	0.431685966

cg15653173	SOX1	0.00043997	0.431657658
cg19866866	ZNF648	5.59E-05	0.431633034
cg17371081	NELL1;NELL1	0.000187194	0.431574241
cg08470742	THBS2	2.58E-05	0.431571747
cg24759795	CSMD3;CSMD3	1.11E-05	0.431541241
cg23474501	GPR123	4.34E-05	0.431536943
cg02446647	EYA4;EYA4;EYA4	0.001639597	0.431517574
cg27282264	A2BP1;A2BP1;A2BP1	8.60E-06	0.431475662
cg18181607		8.60E-06	0.43140324
cg10906284	AVPR1A	8.60E-06	0.431393509
cg14614314	CALY;CALY	0.001147425	0.43138739
cg02506984	RHEB	2.58E-05	0.43136252
cg07060551	SHANK1	4.34E-05	0.431361843
cg16316649	ACADL	1.96E-05	0.431360975
cg23949389		8.60E-06	0.431337912
cg04996277	KCNN2	1.11E-05	0.431287945
cg01146232	SALL1;SALL1	0.000187194	0.431283335
cg18404308	CNGA3;CNGA3;CNGA3;CNGA3	2.58E-05	0.431275548
cg13586599	SCGN	8.60E-06	0.431272795
cg12591770		8.60E-06	0.431269455
cg24565369	KCND2	0.000290372	0.431269137
cg24719005	EFEMP1;EFEMP1;EFEMP1	1.50E-05	0.431259604
cg00755448	KCNB2	4.34E-05	0.431236245
cg06261937	DCC;DCC	2.58E-05	0.431198786
cg04797725		4.34E-05	0.431185356
cg18533282	SPATA18;SPATA18	2.58E-05	0.431178076
cg08475953	COL23A1	0.000233422	0.431151869
cg21250356		4.34E-05	0.431137115
cg10437245	VAX1;VAX1	1.96E-05	0.43113423
cg17087479		5.59E-05	0.431121182
cg16705627	SOX1;SOX1	4.34E-05	0.431103192
cg24784794	ROBO1;ROBO1;ROBO1;ROBO1	3.34E-05	0.431077135
cg05373457	KCNS2	1.96E-05	0.431047497
cg23080354	PHF21B;PHF21B	0.000117986	0.431046049
cg11497372		2.58E-05	0.431039094
cg03257575		3.34E-05	0.431000272
cg06613095	SOX14;SOX14	0.000187194	0.430995834
cg22454769	FHL2;FHL2;FHL2;FHL2	1.96E-05	0.430989597
cg23984434	GUCY1A2	1.50E-05	0.430982915
cg21430539	MOGAT1	2.08E-05	0.430946101
cg24189721		1.50E-05	0.430936299
cg22888418	LRAT	8.60E-06	0.430909838
cg03091551	OTX2	8.60E-06	0.430899322
cg05767421	MAL2	8.60E-06	0.430854835
cg08476595	LOC169834	8.60E-06	0.430835495
cg15593298	PAQR9	9.24E-05	0.430830798
cg11041835	GULP1	1.96E-05	0.430812449
cg15071611	C11orf70	0.000117986	0.430765347
cg14632696	PDLIM3;PDLIM3	0.000148457	0.430757959
cg05009601	FEZF1;FEZF1;FEZF1;FEZF1	8.60E-06	0.43070561
cg21672843	PTPRT;PTPRT	0.000148457	0.43070042
cg19707040	CTNNA2;CTNNA2;CTNNA2;CTNNA2	3.34E-05	0.430684703
cg15808426		1.50E-05	0.43067287
cg27095222		0.000537905	0.430670087
cg23953697	PCDHA7;PCDHA12;PCDHA6;PCDHAC1;PCDH	2.58E-05	0.430658518
cg01088070	B3GAT2	1.96E-05	0.43062536
cg04768697	CCDC105	8.60E-06	0.430610151
cg25451082	ARSJ	0.000357885	0.430608081
cg17514558	PCDHB19P	5.59E-05	0.430591284
cg17741501		7.23E-05	0.430584771

cg02696327	CBLN1	7.23E-05	0.430576982
cg16416793	YAP1;YAP1	0.005897668	0.430575763
cg07139509	C14orf162	0.000148457	0.430563593
cg23729107	FBXO39	0.000117986	0.430516841
cg18516557		5.59E-05	0.430507914
cg26139133	THRB;THRB;THRB	0.000233422	0.430503919
cg13438337	PCSK5	2.58E-05	0.430483163
cg06554535	PARVA	8.60E-06	0.430460791
cg08602008		0.000148457	0.430446446
cg01065349		1.11E-05	0.430437257
cg26364899	PPARG;PPARG;PPARG	1.96E-05	0.430400692
cg23861668	NELL1;NELL1;NELL1;NELL1	0.000537905	0.430398859
cg10611016	C11orf42	1.96E-05	0.430375092
cg11461298	UNCX	8.60E-06	0.430336838
cg22763718	PCDH8;PCDH8	4.34E-05	0.430317399
cg09437327	LOC100128811;GPR158	1.50E-05	0.430312585
cg19989043	CD2AP	4.34E-05	0.430305337
cg24125828	PRRT1	1.50E-05	0.430300476
cg22865798	NRG1;NRG1;NRG1;NRG1;NRG1;NRG1;NRG1;	0.000117986	0.430276151
cg19739596	MS4A3;MS4A3;MS4A3;MS4A3;MS4A3;MS4A3	1.96E-05	0.430268375
cg15312943	SERGEF	4.34E-05	0.430265596
cg23409774	CPLX2;CPLX2	1.50E-05	0.430251143
cg13839457		0.000233422	0.430236776
cg26049998	PITPNC1;PITPNC1	0.003189589	0.430206612
cg18502142	DLX6AS	0.000187194	0.430180552
cg21088983	CLVS1	1.50E-05	0.43015469
cg09976984		1.11E-05	0.430145914
cg14823851	TBX4	8.60E-06	0.430144849
cg19478298	RELN;RELN	1.96E-05	0.430133727
cg12078605	CTNNA2;LRRTM1;CTNNA2	1.96E-05	0.430122697
cg03129384	FAM196A;DOCK1	7.23E-05	0.430102443
cg26980244	NEFM;NEFM;NEFM	0.000187194	0.430093759
cg12300724		2.58E-05	0.430079658
cg09868598		1.50E-05	0.430066056
cg07365127		5.59E-05	0.430064729
cg08184159	TBX5;TBX5;TBX5;TBX5	1.11E-05	0.430058444
cg06755438	CCDC108	1.50E-05	0.430045123
cg21338532	RPRM	0.000117986	0.430033415
cg04847920	TMEM98;TMEM98	8.60E-06	0.430028334
cg19937938	RSPO4;RSPO4	1.96E-05	0.430002739
cg18790597	FAM184B	0.000117986	0.429973916
cg23062112	ANO3	8.60E-06	0.429969833
cg22732749	PCDHB15	2.58E-05	0.429956749
cg27200257	P4HB	1.96E-05	0.429932543
cg19712603	PCDH8;PCDH8	0.000122184	0.429929351
cg16727923	SLITRK1	0.000290372	0.42988158
cg26781575	FAM171B	8.60E-06	0.429879173
cg12800244	SGEF	1.96E-05	0.42984864
cg18940047	DPP6;DPP6	1.50E-05	0.429794739
cg06678279	ERC2;ERC2	9.24E-05	0.429741019
cg13962186	FOXB2	3.34E-05	0.429723805
cg22399790		8.60E-06	0.429710905
cg01119452	ELMO1	0.000148457	0.429701992
cg00295572	TP73	0.000233422	0.429695293
cg03024372	FAM83B	0.000233422	0.429662062
cg20509869		0.000537905	0.429654638
cg00476955	TRPC2	7.23E-05	0.429646496
cg16971589	RHOBTB1;RHOBTB1	0.000187194	0.429645407
cg13670601	SLC6A15;SLC6A15;SLC6A15	3.34E-05	0.42964473
cg04450459		8.60E-06	0.429643632

cg04005707	CWH43;CWH43	1.11E-05	0.429608394
cg00439981	FAM120B	0.000148457	0.429537786
cg14146740		0.000148457	0.429536934
cg27656786	GPR126;GPR126;GPR126;GPR126	8.60E-06	0.429478732
cg06874656	NTM;NTM;NTM;NTM	4.34E-05	0.4294748
cg18877506	PDPN;PDPN;PDPN;PDPN	2.58E-05	0.429473709
cg08545287	A2BP1;A2BP1	3.34E-05	0.429469312
cg18759209	BMP7	0.000117986	0.429462349
cg25553799	SHISA2	8.60E-06	0.429458535
cg16404865	KCNC1;KCNC1	1.11E-05	0.429436401
cg14627348		0.000148457	0.429433921
cg03405909		5.11E-05	0.429414205
cg14294758	LSAMP	1.96E-05	0.429406513
cg24685134	TTC22;TTC22	0.000334462	0.429398352
cg06769296	LOC100128239	1.50E-05	0.429373994
cg09118258	LMX1A	1.18E-05	0.429373649
cg07224726	LVRN	3.34E-05	0.429362948
cg10989763	NXPH2	8.60E-06	0.42935891
cg01435766	SLC5A11	3.21E-05	0.429351066
cg13176012		2.58E-05	0.429348464
cg13021619	NKX2-6	1.11E-05	0.429339209
cg00510598	IRX6	4.34E-05	0.429285149
cg03307911	CYP24A1;CYP24A1	3.34E-05	0.429280248
cg02062409		7.23E-05	0.429275904
cg25930786	TARM1	4.34E-05	0.42927508
cg01474011	CUL3	4.34E-05	0.429273671
cg22583065	FGF14	0.000148457	0.429246821
cg03558805	CLDN3	1.50E-05	0.429238074
cg18878616	PCDHGA1;PCDHGA2;PCDHGA2	8.60E-06	0.429226574
cg17291435	FHL2;FHL2;FHL2;FHL2;FHL2;FHL2;FHL2	0.000570759	0.429209561
cg19937061		5.59E-05	0.429196029
cg00862117	IQSEC3	8.60E-06	0.429183265
cg21127597	MPZL3	9.24E-05	0.429182324
cg13908315		0.000187194	0.429181254
cg11342198		0.000537905	0.42916393
cg17299437		0.000187194	0.429146475
cg24596998	C1orf115	0.00043997	0.429140165
cg12243375	GUCY1A2	0.000117986	0.429094557
cg17670477		5.59E-05	0.429090077
cg22541254	GRIK2;GRIK2;GRIK2	1.11E-05	0.42903733
cg18761549		1.11E-05	0.429035421
cg05625362	TLL1	1.50E-05	0.429028108
cg12792250		1.11E-05	0.429010577
cg00186909	PLEKHG7	0.000537905	0.428985428
cg17629929		0.000537905	0.428983903
cg26333822	SLC5A7;SLC5A7	1.11E-05	0.428982163
cg17453778	CASR;CASR	2.58E-05	0.42897725
cg03689552	MEFV	9.24E-05	0.428968689
cg27590049	LMX1A	1.96E-05	0.428967688
cg17875483		0.000233422	0.428950885
cg25908985	IHH	0.000357885	0.428941648
cg07955752	VSTM2B	8.60E-06	0.428934537
cg03585912		8.60E-06	0.428927986
cg13238990	NLGN1	1.11E-05	0.428915075
cg23760687		9.24E-05	0.428894381
cg08949339		1.50E-05	0.428892302
cg22657780	ZNF385B;MIR1258;ZNF385B	0.000357885	0.428870531
cg20060206	LMO3;LMO3	8.60E-06	0.428862762
cg05627557		4.34E-05	0.428819709
cg13533151	CHST9	8.60E-06	0.428804996

cg21282549		9.24E-05	0.428776362
cg13519184		9.24E-05	0.428738961
cg21753226	KCNQ1DN	1.11E-05	0.42871872
cg00630212	CXCL2	0.000290372	0.428695085
cg11873482	TAC1;TAC1;TAC1;TAC1	4.34E-05	0.428669191
cg22079008		8.60E-06	0.428666318
cg12127472	C17orf104	7.23E-05	0.428645856
cg18729001	DBX2	2.58E-05	0.428637672
cg06881965	DCAF5	7.23E-05	0.428617639
cg14055896	CACNG8	8.60E-06	0.428615955
cg06425919	MYOD1	1.11E-05	0.428592295
cg11051843	SEMA3C	0.000148457	0.428557312
cg04508964	RFX4	8.60E-06	0.428550829
cg15235987	EP400	3.34E-05	0.428537176
cg17167852	PCSK9	1.96E-05	0.428498778
cg26723355	PDE10A;PDE10A	0.000290372	0.428452305
cg17619823	ADRB3	1.11E-05	0.428444698
cg25137403		8.60E-06	0.428442553
cg11581706	CACNA2D1	7.23E-05	0.428370538
cg20092531		4.34E-05	0.428367529
cg12172441		7.23E-05	0.428337848
cg13331559	MCOLN3	8.60E-06	0.428334767
cg21762788		0.000187194	0.428322349
cg21173447	DSCAM;DSCAM	7.23E-05	0.428321481
cg01614020	NEFL	0.000290372	0.428273277
cg16909109	KIF6	0.001376172	0.428268679
cg20168230	GRIK3	2.58E-05	0.42826139
cg23519626	P4HA3	5.59E-05	0.428252622
cg16990168	TBX15	9.24E-05	0.428237815
cg25307881	MUL1	0.000233422	0.428230681
cg04523524	NMU	4.34E-05	0.428215578
cg09062550	INSM1;INSM1	9.24E-05	0.428194718
cg21474897	CLDN11	8.60E-06	0.428193106
cg17194154		5.59E-05	0.428187419
cg03863591		8.60E-06	0.42818354
cg02048505	C7orf57	1.50E-05	0.428159592
cg06373940	ERCC3	7.23E-05	0.428152267
cg08979895	PITX2;PITX2;PITX2	8.60E-06	0.428149233
cg07006091		7.23E-05	0.428143741
cg18803104		0.000791389	0.428134374
cg02774855	CLIP4	0.000187194	0.428131507
cg12160258	TMEM26	8.60E-06	0.428113033
cg27180153		1.50E-05	0.428097858
cg02963248		0.000233422	0.428087576
cg01432561	NEFL	1.11E-05	0.428074655
cg25135018	IL6R;IL6R	0.005897668	0.428037222
cg09023820	MATN2;MATN2	3.34E-05	0.42803427
cg20598560	SGPP2	4.34E-05	0.428032347
cg05171937	STK38L	7.23E-05	0.428015266
cg23815582	SLC30A10;SLC30A10	9.24E-05	0.428011748
cg17991436	RFX6	3.34E-05	0.428011421
cg27319917	N4BP1	4.34E-05	0.427991049
cg20086208	GPR139	5.59E-05	0.427944863
cg07427438		1.50E-05	0.427886318
cg05735180		9.24E-05	0.427861289
cg07749412	C3P1	0.000117986	0.427843175
cg26930596	PRKCZ;PRKCZ;PRKCZ	7.23E-05	0.427834238
cg27612364	SLC25A27;CYP39A1	9.24E-05	0.427831423
cg01388649		5.59E-05	0.427829056
cg16023545	GBX2	1.50E-05	0.427814867

cg26960562	DNAH9	1.11E-05	0.427809997
cg12114834	BSX	5.59E-05	0.427804893
cg26517714	TLX3	1.96E-05	0.427791569
cg15543551	FGF12	1.96E-05	0.427784537
cg14419046		0.000148457	0.427771382
cg25889711		1.50E-05	0.427732868
cg25784308	FAM123C;FAM123C;FAM123C;FAM123C;FAM1	1.50E-05	0.427715917
cg26669044	RALYL;RALYL;RALYL;RALYL;RALYL;RALYL;R	3.34E-05	0.427710395
cg24935332		2.58E-05	0.427701387
cg16419066	MGC2889;HRASLS	3.21E-05	0.427697702
cg09112514	PDGFRA	1.96E-05	0.427689184
cg07649491	FAM190A	1.50E-05	0.427651986
cg17508941		8.60E-06	0.427642668
cg19358493	EMX2;EMX2OS;EMX2	1.11E-05	0.427633474
cg25285904		8.60E-06	0.427628829
cg10447327	LANCL1;LANCL1;LANCL1	0.000148457	0.427617166
cg10752508	CPOX	7.23E-05	0.427608784
cg17007896		9.24E-05	0.427592701
cg16701939	KCND3;KCND3	1.96E-05	0.427582001
cg16849041	DOCK5;DOCK5	0.000537905	0.427579469
cg12989574	GPC6;GPC6	3.34E-05	0.427557372
cg10603004	NKX2-6	8.60E-06	0.427553733
cg07473471	ESRP1;ESRP1;ESRP1;ESRP1;ESRP1	0.000955793	0.427542963
cg04121771	TM4SF4	3.34E-05	0.427523191
cg23215729	RIPPLY2	3.34E-05	0.42751077
cg06294712	BHLHE22	4.34E-05	0.427472195
cg19636519		8.60E-06	0.427470834
cg01763916	SMAP2	0.000148457	0.427441251
cg24740868	C1orf87;C1orf87	1.11E-05	0.427441158
cg21578219	IGSF21;IGSF21	5.59E-05	0.427436901
cg02824386		3.34E-05	0.427433103
cg08675717	PHACTR3	0.000233422	0.427431715
cg12423584	SCIN	1.11E-05	0.42741428
cg11781306	PCDHA2;PCDHA1;PCDHA1;PCDHA3;PCDHA4	4.34E-05	0.427388082
cg14886059	ZSCAN1	1.50E-05	0.427378069
cg08413157	PTPRT;PTPRT	0.000117986	0.427373272
cg27493301	PRICKLE1	0.000357885	0.427372059
cg12563372		8.60E-06	0.427343496
cg22861116	PCDHGA4;PCDHGA7;PCDHGA6;PCDHGA1;PC	0.000148457	0.427316781
cg10137084	CACNA2D3	0.000117986	0.427315442
cg12472483	PTPRN2;PTPRN2;PTPRN2	8.60E-06	0.427307737
cg07493197		0.000365373	0.42729823
cg26664161	FBXL21	4.34E-05	0.427282949
cg10976975	BMP10;BMP10	2.58E-05	0.427234778
cg09488203	GPR120	3.92E-05	0.427220776
cg21473142	EOMES	0.000955793	0.427216453
cg17019292	MARVELD2;MARVELD2	8.60E-06	0.427210305
cg04306063	CRHBP	4.34E-05	0.427208116
cg08491188	PCDHGA4;PCDHGA6;PCDHGA1;PCDHGA8;PC	1.96E-05	0.427200416
cg06998965	MARVELD2;MARVELD2	8.60E-06	0.427184824
cg02318926	BNC1	5.59E-05	0.427171379
cg07149296	KIAA1755	1.96E-05	0.427166648
cg10652091	SNED1	9.24E-05	0.427166187
cg05754435	KDR	9.24E-05	0.427129406
cg07779444	GPR123	8.60E-06	0.427120706
cg08312626		2.80E-05	0.427117616
cg12505170		0.000117986	0.427110086
cg18026227	LOC100192378;ZFHX4	0.000117986	0.427097721
cg01038450	MATN2;MATN2	7.23E-05	0.427070263
cg18104012	FEZF2	0.001639597	0.427045531

cg05663573	SLC7A14	4.34E-05	0.427026952
cg18556676		1.96E-05	0.426972093
cg24728018	CLVS2;CLVS2	2.58E-05	0.42695984
cg11238048	DACT2	5.59E-05	0.426920749
cg18635723	DPYSL3	1.96E-05	0.426911952
cg21113740	ELTD1;ELTD1	4.34E-05	0.426908271
cg00522056	RPRM	0.000187194	0.426906346
cg14664759	KCNT2	8.60E-06	0.426898874
cg12258785	THRB;THRB;THRB	0.000117986	0.426898047
cg07442479	GDNF;GDNF	1.11E-05	0.426873087
cg05375487	GALNT11	4.34E-05	0.426861492
cg20019985		9.24E-05	0.426857248
cg04599026	DLX6AS;DLX6	0.000791389	0.426849603
cg02160684	TRIB1	0.000148457	0.42682574
cg07850604	INSM2;INSM2	1.96E-05	0.426816479
cg10443195		0.001376172	0.426755056
cg26163578		3.34E-05	0.426722076
cg15381304	GPR6	8.60E-06	0.426719246
cg06103031	BRUNOL4;BRUNOL4;BRUNOL4;BRUNOL4	9.24E-05	0.42671359
cg26758986	P4HA3	0.000110797	0.426691079
cg14788242	SEC61A1	8.60E-06	0.426685325
cg23087992	SOX2OT;SOX2	0.000187194	0.426682256
cg07654569	FAM164A	8.60E-06	0.426677228
cg16921310	ASCL1;ASCL1	0.000290372	0.426670378
cg12918457		9.24E-05	0.426668863
cg16419354	FAM163A	8.60E-06	0.426664888
cg09168808		8.60E-06	0.426664318
cg04823455	ARSJ;ARSJ	1.50E-05	0.426657396
cg00167654	WSCD1	0.000791389	0.426653481
cg26892444	FAM159B	1.50E-05	0.426619163
cg14353137		1.96E-05	0.426610812
cg07121856	PON3	5.59E-05	0.42659886
cg20230721	TFPI2	9.24E-05	0.426597512
cg13348059		0.000187194	0.426593508
cg23770904	GATA5	9.24E-05	0.426587608
cg24229589	CILP2	8.60E-06	0.426579293
cg16068263		2.58E-05	0.42656132
cg23336892		3.34E-05	0.426559019
cg17453460		9.24E-05	0.426521069
cg11671363	MIR145;LOC728264	3.34E-05	0.426519608
cg23400512	FLRT2	1.96E-05	0.426461349
cg11078221		8.60E-06	0.426449737
cg00418880	HS6ST3	4.34E-05	0.426447317
cg18417954	C19orf51	3.34E-05	0.426439237
cg05109245	GREB1L	5.59E-05	0.426438248
cg07781332	ADAMTS19	1.11E-05	0.426435497
cg22614239	ZIC4;ZIC4;ZIC4;ZIC4;ZIC4	1.50E-05	0.426427764
cg09422450		0.000537905	0.426415483
cg02041484	NRF1	1.96E-05	0.426379958
cg26954174	NOD2	0.000117986	0.426368661
cg15712559	PHACTR3	0.000148457	0.426366185
cg21200129	ELAVL2;ELAVL2;ELAVL2	1.96E-05	0.426345088
cg06168950		3.30E-05	0.426333336
cg09871315	HOXA2	4.34E-05	0.426330005
cg02621287		5.59E-05	0.426321393
cg03701992	C21orf88;C21orf88	2.58E-05	0.426313596
cg12414557	RNF212;RNF212;RNF212;RNF212	0.000117986	0.426302963
cg18626709	LEPREL1;LEPREL1;LEPREL1	2.58E-05	0.426292945
cg24280540		1.11E-05	0.426290308
cg27579953	LOC283392;TRHDE;LOC283392	5.11E-05	0.426286356

cg13713830		3.34E-05	0.426264024
cg08253808	WDR20	7.23E-05	0.426256598
cg17846016		7.23E-05	0.426255457
cg25649000	PTPRT;PTPRT	0.000290372	0.426234542
cg02081052		4.34E-05	0.426227273
cg14289542		1.11E-05	0.426219534
cg13468144	ANKFY1	1.50E-05	0.426207205
cg16366473	FGF12;FGF12	4.34E-05	0.426198872
cg00215432	MIR375	0.000290372	0.426184634
cg26721193		0.000117986	0.426166062
cg14717170	SLITRK3	4.34E-05	0.426117322
cg06124827	MORN4;MORN4	0.000791389	0.426105735
cg11106652		5.59E-05	0.426085096
cg06533244		8.60E-06	0.426022045
cg05757967	TPBG;TPBG	0.002714607	0.426015371
cg13274254	GULP1;GULP1	4.34E-05	0.426007846
cg05421564	FAM150A	1.11E-05	0.426003462
cg00348762	DKFZP434H168;GNAO1;GNAO1	9.24E-05	0.425994631
cg07516556	LPCAT1	1.96E-05	0.425985036
cg15058464	C20orf56	0.00043997	0.425973977
cg15577595	ZNF804A	2.58E-05	0.425969372
cg08830502		8.60E-06	0.425966872
cg16615154		8.60E-06	0.425952993
cg08624648		0.00043997	0.425948451
cg03308652		3.34E-05	0.425945859
cg21053529	GJD2	8.60E-06	0.425938183
cg24304919	KCNN2	1.11E-05	0.425923644
cg06614469	MAPK4	0.000537905	0.425920844
cg07603511		1.11E-05	0.425919873
cg14290904	NKX2-1;NKX2-1	1.50E-05	0.425902237
cg07525077	RNASE3	0.000187194	0.425883332
cg04006554	ENPP5	9.24E-05	0.425868238
cg00026703	NR2F2	7.23E-05	0.425825577
cg02574502	CACNG8	0.001948248	0.425812612
cg16845486	NELL2;NELL2;NELL2;NELL2;NELL2	0.000537905	0.425773713
cg24621437	SORCS1;SORCS1	0.000654035	0.425761848
cg00370229		5.59E-05	0.425760306
cg12315713	CCDC67;CCDC67	8.60E-06	0.42575227
cg26654798	BNC1	8.60E-06	0.425751571
cg01141721	SRC;SRC	7.23E-05	0.425748096
cg09697978	PRKD3	1.50E-05	0.425737928
cg13595556		1.11E-05	0.425733355
cg07504763		8.60E-06	0.425714592
cg04657224	XKR6	0.000357885	0.425713503
cg20432211		2.58E-05	0.425711891
cg27501759	OXTR	3.34E-05	0.425709368
cg15010903	TIMP2	1.50E-05	0.425686392
cg14314071	PMP22;PMP22;PMP22	1.11E-05	0.425666216
cg16546016	C4orf31	1.50E-05	0.425593336
cg19764599	LHX8	9.24E-05	0.425592131
cg15834072	DCHS2;DCHS2	1.18E-05	0.425573254
cg03726556		2.58E-05	0.425573176
cg08516516	CDO1	0.000526175	0.425568614
cg15221604	ROBO3	0.000290372	0.425567965
cg26894523	ZNF718	8.60E-06	0.425554313
cg20723129		3.34E-05	0.425548051
cg12544191		7.23E-05	0.425514927
cg17403699		1.11E-05	0.425498876
cg17112958	LYPD1;LYPD1	1.11E-05	0.425495041
cg24362812	FAM38B	0.000117986	0.425486252

cg05302420	ENTPD7	0.000290372	0.425484516
cg01295392	ASCL4;ASCL4	8.60E-06	0.425468516
cg23002590	RPS6KA2;RPS6KA2	0.000233422	0.425465317
cg16080876	NEFL	1.50E-05	0.4254512
cg25684151		3.34E-05	0.42543469
cg00893471	HOXD3	7.23E-05	0.425431164
cg03167683	B4GALNT2;B4GALNT2;B4GALNT2;B4GALNT2	1.11E-05	0.42542413
cg14526047	C10orf53;C10orf53	1.50E-05	0.425383842
cg06015422		8.60E-06	0.425383601
cg27307298	RNF207	1.11E-05	0.425378751
cg07197785		0.000537905	0.425375069
cg01088410		0.000187194	0.425357833
cg17939805		3.34E-05	0.425339851
cg10601582	ZNF98	9.24E-05	0.425337648
cg14626375	WASF3	0.000117986	0.425330258
cg25107254	MTNR1A	8.60E-06	0.425327919
cg25752703	KIAA1257	3.34E-05	0.42531297
cg08231710	MMP23A;MMP23B	2.58E-05	0.425282495
cg04194947		1.96E-05	0.425274179
cg14609407	SLPI	2.58E-05	0.425271575
cg00066854		4.34E-05	0.425191003
cg02069715	GABRG3	1.50E-05	0.425179557
cg17320707	EMX2;EMX2	3.34E-05	0.425173176
cg24868359	GRIK1;GRIK1	3.34E-05	0.425171721
cg24091995		5.59E-05	0.42516592
cg16506970	KIAA0232;KIAA0232	5.59E-05	0.425163998
cg02572729	DDAH1;DDAH1;DDAH1	7.23E-05	0.425149391
cg18591496	CYP2A13	5.59E-05	0.425130605
cg17342807		7.23E-05	0.425111793
cg02106850	TBX18	1.96E-05	0.425101932
cg13611347	NOL4	0.000262362	0.425081498
cg12886942		0.000148457	0.425080807
cg05495351	NRXN1;NRXN1	3.34E-05	0.425029128
cg00452199	ACCS;ACCS	9.24E-05	0.425025742
cg11661868	BOK	0.000187194	0.424999326
cg24827600	LASS6	9.24E-05	0.424990843
cg18568589	ZNF560	2.58E-05	0.424988991
cg14344102	PRDM13;PRDM13	2.58E-05	0.424983195
cg11672037	SEMA6D;SEMA6D;SEMA6D;SEMA6D;SEMA6D	0.000791389	0.424972341
cg05336395	PCDH8;PCDH8	8.60E-06	0.424971593
cg25448856	EEA1	3.34E-05	0.424955605
cg20779964	CBLN4	0.000148457	0.424954852
cg02800607	HPSE2;HPSE2;HPSE2;HPSE2	8.60E-06	0.424951188
cg15523958	PLEKHH2	4.34E-05	0.424936866
cg03402459		1.11E-05	0.424931224
cg16063112	C10orf107	8.60E-06	0.424914983
cg09588792	SEPT10;ANKRD57;SEPT10	0.000233422	0.424896904
cg03637815		7.23E-05	0.424889426
cg03167429	KIRREL3;KIRREL3;KIRREL3;KIRREL3	3.34E-05	0.424880883
cg11793699	NTRK1;INSRR	0.000148457	0.424870075
cg16962008	C7orf57	8.60E-06	0.424854326
cg09052453	ADAMTS19	1.96E-05	0.424851897
cg04202620	ARHGEF10	2.58E-05	0.424848844
cg00130165	EPHA5;EPHA5	1.96E-05	0.424844666
cg03457729		0.000148457	0.424836399
cg12931029	MNX1;MNX1	8.60E-06	0.424810835
cg04205943	SCIN	4.34E-05	0.424800523
cg24921634	MYO5B	1.50E-05	0.424779836
cg10741333	RGS6	1.96E-05	0.424766667
cg08509172	TM6SF2	2.39E-05	0.424765589

cg19730379	C10orf26;C10orf26	1.96E-05	0.424751531
cg17838026	KCNC3	1.11E-05	0.424742922
cg10173774		8.60E-06	0.424707609
cg26712080	WNT5A	0.000187194	0.424675654
cg11345323	PRKCZ	0.004357816	0.424673418
cg00174428	LMX1A	1.96E-05	0.42467135
cg19529326	EPB41L4A;FLJ11235;EPB41L4A	0.002266401	0.424616686
cg14270292	EYA4;EYA4;EYA4;EYA4;EYA4;EYA4	0.000654035	0.424567074
cg08498254		7.23E-05	0.424549555
cg15466862	SOX1	1.96E-05	0.424535907
cg17661642		8.60E-06	0.424509862
cg11036833	CDO1	0.000187194	0.424508344
cg05893614	FAM19A4;FAM19A4	8.60E-06	0.424501329
cg18446110	SLC23A1;SLC23A1	9.24E-05	0.424489317
cg17412886	ISL1	0.000148457	0.424477505
cg25933341	SOX2OT;SOX2	2.58E-05	0.424471014
cg12565637	IHH	0.000334462	0.424461852
cg07013734	TRPC6	4.34E-05	0.424458773
cg05418105		1.11E-05	0.424458676
cg19727641	MRAS;MRAS;MRAS	3.34E-05	0.42443763
cg08475096	GRIA2;GRIA2;GRIA2	1.96E-05	0.424434901
cg04415731		8.60E-06	0.424429723
cg06894334		1.50E-05	0.4244165
cg01495122	SFRP1	1.96E-05	0.424415913
cg22713444	GNRHR2	0.000187194	0.424370922
cg09313917	PRKAA2	1.11E-05	0.424364563
cg17138852		5.59E-05	0.424363409
cg14116122	ALX1	8.60E-06	0.42434849
cg02961004		0.000187194	0.424343081
cg03531247	NRXN1;NRXN1;NRXN1	2.58E-05	0.424319934
cg09767822	DBX1	1.18E-05	0.424252148
cg09477453		1.11E-05	0.424248061
cg00939495	DRD5;DRD5	0.000187194	0.424240993
cg26837399	NID1	5.59E-05	0.424222755
cg12666279	DPP10;DPP10	0.000148457	0.424220172
cg00263760	VAX1;VAX1	8.60E-06	0.424219009
cg17301902	GNA14;GNA14	8.60E-06	0.424218392
cg25391815		9.24E-05	0.424198781
cg10614021	GRIN2B	4.34E-05	0.424178426
cg05460130	EIF5A2	0.000117986	0.424174736
cg20098887	RAB32	3.34E-05	0.424171367
cg25796439	ISM1	0.000117986	0.424153679
cg20318166	ALPK3	0.00043997	0.424150115
cg04350675		0.001376172	0.42413711
cg15058210	HDAC4	0.000148457	0.424131984
cg04028634	ADCY1	0.000290372	0.424124982
cg26205771	NPBWR1	5.59E-05	0.42412366
cg22537474	SNAP25;SNAP25	1.96E-05	0.424110217
cg04006722	GLRB;GLRB;GLRB	3.34E-05	0.424091232
cg17783401	RGS20	7.23E-05	0.42408541
cg04267526		0.000233422	0.424079143
cg27288226		2.58E-05	0.424066446
cg21606928		0.00043997	0.424056165
cg07495389	MAPRE3	1.96E-05	0.424049116
cg03336086	THBS4;THBS4	1.50E-05	0.424019007
cg09222749	LHX8	1.50E-05	0.423998317
cg03574723	GPR26	8.60E-06	0.42398773
cg22822803	WDR8	8.60E-06	0.423977322
cg07351192		3.34E-05	0.423957943
cg04075726		3.34E-05	0.423931526

cg18674980	CA3	1.50E-05	0.423922448
cg01036409	FAM20A	0.000148457	0.423917918
cg19205994	PLD5	0.000148457	0.423909233
cg06391468	SOX9;SOX9	7.23E-05	0.423908706
cg10236224		0.000262362	0.423895105
cg00773902	GDF1;LASS1;LASS1	0.000955793	0.423892424
cg22665692	DOCK1	0.000537905	0.423884827
cg09831105	ANKS1B;ANKS1B;ANKS1B	3.34E-05	0.423871814
cg06582394	CASR;CASR	1.50E-05	0.423868854
cg15166311		4.34E-05	0.423867069
cg14201424	MGC45800	0.000187194	0.423853054
cg11196437	LHX8	2.58E-05	0.423850714
cg18243760	PRRT1	4.34E-05	0.423849967
cg16316624	TMEM216	0.000791389	0.423831966
cg03348161	MS4A3;MS4A3;MS4A3	0.000117986	0.423831034
cg24430140		9.24E-05	0.423819008
cg23243463	BRUNOL4;BRUNOL4;BRUNOL4;BRUNOL4	0.000187194	0.423808655
cg24202131	BRUNOL4;BRUNOL4;BRUNOL4;BRUNOL4	8.60E-06	0.423806286
cg08063317	GABRB3;GABRB3	2.58E-05	0.423758942
cg25985659	FAM155A	1.50E-05	0.423744436
cg01791371		1.96E-05	0.423743777
cg27280396	RORA	5.59E-05	0.423737568
cg19940537	BICC1	1.96E-05	0.423714331
cg17227967	C20orf103	0.000148457	0.423713678
cg09571420	CNTNAP2	0.000187194	0.423707493
cg26142412		0.000148457	0.42370723
cg21529405	CBLN2	8.60E-06	0.423683913
cg21121843	HTT	0.000148457	0.423668326
cg22007227	ZBTB8B	5.59E-05	0.423666024
cg07480762	PLCH1	7.23E-05	0.423640137
cg24275315	NKX6-1	8.60E-06	0.423627022
cg23990012		4.34E-05	0.423622502
cg17817709	PLOD2;PLOD2	8.60E-06	0.423617675
cg04430911	OSCP1;OSCP1	2.39E-05	0.423606392
cg22130145		1.96E-05	0.423605158
cg09017434	MARCH11	2.08E-05	0.423588089
cg20676716	HOXD1	8.60E-06	0.423577674
cg19503977	HTR1B	3.34E-05	0.423569908
cg16940012		1.11E-05	0.423567474
cg12323274	BRUNOL4;BRUNOL4;BRUNOL4;BRUNOL4	9.24E-05	0.423560019
cg02347074	PAX3;PAX3;PAX3;PAX3;PAX3;PAX3	1.11E-05	0.423513903
cg09500672	LYPD6	1.50E-05	0.423501248
cg05710997	ISL1	1.50E-05	0.423483472
cg26582643	C20orf103;C20orf103	0.001948248	0.423480151
cg25308508		1.96E-05	0.423475499
cg17046890	HOXB8	8.60E-06	0.423468355
cg23588462		0.000117986	0.423456839
cg13454407	KCNV1	8.60E-06	0.423445649
cg25336332	THRB;THRB;THRB	0.000357885	0.423428103
cg09980058	COMP	5.59E-05	0.423411737
cg24218109	SH3BGRL2;SH3BGRL2	2.58E-05	0.423398153
cg00592781		3.34E-05	0.423386514
cg22714094	NPY1R	5.59E-05	0.423385197
cg06468908	COL25A1;COL25A1;COL25A1;COL25A1	8.60E-06	0.42337416
cg00261690	SNHG3-RCC1;SNHG3-RCC1;SNHG3-RCC1;RC	3.34E-05	0.423357267
cg20419307	PPFIA2	1.11E-05	0.423353571
cg09273641	PHF21B;PHF21B	4.34E-05	0.423351696
cg19890879		1.11E-05	0.423349055
cg02928664	CDC42EP5	0.000117986	0.423285117
cg24789487	FAM171B;FAM171B	0.000117986	0.423278437

cg04684516	SNCAIP;SNCAIP	5.59E-05	0.42327511
cg15462887	BDNF	8.60E-06	0.423267032
cg26945715	TBC1D12	1.50E-05	0.423245074
cg26476852	HOXA9	3.34E-05	0.423232365
cg18595065		1.50E-05	0.423159831
cg02352240		1.50E-05	0.423158967
cg13161658	KCNA4	4.34E-05	0.42315677
cg17410650		1.50E-05	0.423152746
cg13654588	PRLHR	2.58E-05	0.423136059
cg07788348	CADPS2;CADPS2;CADPS2	3.34E-05	0.42312846
cg02503763	MGLL;MGLL	0.000955793	0.423080114
cg06704093		8.60E-06	0.423064153
cg02753029		8.60E-06	0.423058466
cg10249705	GUCY1A2	7.23E-05	0.423048064
cg22834653	FGF12	1.18E-05	0.42304479
cg06296331	NCKAP5;NCKAP5	2.58E-05	0.423005934
cg22642777	PPP2R2B;PPP2R2B;PPP2R2B;PPP2R2B;PPP2	1.11E-05	0.423005168
cg10809491	TDH	8.60E-06	0.422977523
cg24004483		0.000187194	0.422977027
cg23987444	SLC18A3;CHAT	3.34E-05	0.422965289
cg24393673	OLFM3	2.58E-05	0.422946693
cg03805475		7.23E-05	0.422937968
cg27123392	GABPB1;GABPB1	2.58E-05	0.422933482
cg14096889	ADAMTSL3	8.60E-06	0.422927684
cg13496979		1.96E-05	0.422915204
cg16802646	WWC1;WWC1;WWC1	0.000117986	0.422893545
cg10599384	PCDHGA1;PCDHGA1	0.000187194	0.422884012
cg02313850	BMPR1A	0.000791389	0.42288131
cg14777561	DBX1	4.34E-05	0.422875226
cg19821713		8.60E-06	0.422860826
cg03758467	PCDHA2;PCDHA1;PCDHA1;PCDHA6;PCDHA5	8.60E-06	0.422855897
cg17941572		0.000955793	0.422850252
cg12756396	DMRTA2	4.34E-05	0.422846932
cg20702559	FAM110B	0.000791389	0.42282444
cg05543049	NLGN1	1.11E-05	0.422815371
cg01996714		1.11E-05	0.422798273
cg27191517	NMU;NMU	0.000187194	0.422791077
cg01670677	PHOX2A	5.59E-05	0.42278083
cg23294090	FAM135B	0.000117986	0.422770699
cg16032162	C10orf41;ZNF503	9.24E-05	0.422769748
cg23600372		2.58E-05	0.422755555
cg25365934		1.96E-05	0.422751087
cg00852549	NXPH1	5.59E-05	0.422713108
cg13086983	ECE1	0.000233422	0.422676742
cg21170682		1.11E-05	0.422667531
cg00976453	KCNB1	0.000357885	0.422647972
cg03919781	EFHA2	8.60E-06	0.422646403
cg09123188		5.59E-05	0.422619412
cg07704934	ALX4	1.11E-05	0.422619405
cg06159603	CADPS2;CADPS2;CADPS2	0.000117986	0.422615212
cg23748340		1.96E-05	0.422594733
cg01938650	PCDH7;PCDH7;PCDH7	1.96E-05	0.422583351
cg07744166	WASF3	1.50E-05	0.42257099
cg01758512	FUT9;FUT9	1.96E-05	0.422563791
cg10912240	FOXG1	1.11E-05	0.422544188
cg26094789	PAX9	2.58E-05	0.422542565
cg06987468	WNT2;WNT2	1.50E-05	0.422532767
cg07586235		0.000290372	0.422532628
cg20100745	NDRG1;NDRG1	0.000537905	0.422519111
cg00963378	GJA3	9.24E-05	0.422516537

cg02570563	GPR39;GPR39	2.58E-05	0.422507251
cg11459714	KLK10;KLK10;KLK10	1.11E-05	0.422506618
cg26911220		1.11E-05	0.422506409
cg12589298	KCNC3	0.000233422	0.422501482
cg13236389		3.34E-05	0.422458489
cg22380033	GRB14;GRB14	0.000148457	0.422455807
cg11097433	SORCS1;SORCS1	0.000187194	0.422453246
cg13658899	LHX3;LHX3	4.34E-05	0.42245107
cg14567963		0.000117986	0.422422001
cg26890189	SLC8A2	4.34E-05	0.422419188
cg06090660	CDH2	0.000654035	0.422369087
cg00720159		0.000290372	0.422368046
cg13549845	GRID2	1.50E-05	0.42234536
cg13448753	LOC100128811;GPR158	8.60E-06	0.422328035
cg09416908	ME3;ME3;ME3	0.000290372	0.422325169
cg08612100	HS6ST3	1.50E-05	0.422324629
cg17459431	KCNK1	0.001147425	0.422322814
cg11247817	LNPEP;LNPEP;LNPEP	9.24E-05	0.422295996
cg02683759		0.000233422	0.422288988
cg04452203		7.23E-05	0.422279065
cg22266749	COL25A1;COL25A1	1.11E-05	0.422259602
cg18902090	PCDHA7;PCDHA12;PCDHA6;PCDHAC1;PCDH	8.60E-06	0.422248991
cg03801286	KCNE1	4.34E-05	0.422232191
cg25217100	C13orf36	8.60E-06	0.422187204
cg14512563		1.96E-05	0.422172756
cg17164747	CCDC149;CCDC149	0.000290372	0.422170066
cg22717014	PITX2;PITX2;PITX2	1.11E-05	0.422167253
cg05858607	DOCK1	0.000654035	0.422164521
cg02746725	HOXD1	1.50E-05	0.422143276
cg06289802	SLC9A3R1	3.34E-05	0.422105832
cg07754999	PITX3	9.24E-05	0.422100242
cg07463541	PCDHA2;PCDHA1;PCDHA3;PCDHA3;PCDHA1	7.23E-05	0.422087454
cg04506585	SOX9	3.34E-05	0.422078317
cg24244374		1.11E-05	0.422073119
cg00078299	ZHX1;ZHX1	7.23E-05	0.422041781
cg18369866	POU4F3	1.96E-05	0.42203273
cg24244752	GABRA2;GABRA2	5.59E-05	0.422025695
cg22336004	PDE4B;PDE4B	8.60E-06	0.422023
cg08042975	ERBB4;ERBB4	5.59E-05	0.42200116
cg20875821	SERPINB2;SERPINB2	1.11E-05	0.421994862
cg18915856		1.50E-05	0.421970235
cg09147777	GRIA4;GRIA4;GRIA4;GRIA4	4.34E-05	0.421966679
cg25208863	GOLSYN;GOLSYN;GOLSYN;GOLSYN;GOLSYN	9.24E-05	0.421965243
cg01794426	PAX9	9.24E-05	0.421950028
cg00678890	SLC12A5;SLC12A5	8.60E-06	0.421933396
cg02387803	ZIC4;ZIC4;ZIC4	5.59E-05	0.421917018
cg26332560	ADCY8	1.50E-05	0.421902799
cg07186154	SLC5A7	8.60E-06	0.421898814
cg03340466	FAM163A	8.60E-06	0.421882704
cg18278638	SLC7A14	8.60E-06	0.421875198
cg12859211	ROBO3	0.000866489	0.421868479
cg01763719		0.000537905	0.42186282
cg05603546		9.24E-05	0.421861773
cg05107535		1.96E-05	0.42184481
cg08315174	NKX2-1	3.34E-05	0.421842015
cg24872782	FBXL7	0.000148457	0.421838159
cg13443627	TACSTD2;TACSTD2	4.34E-05	0.42182381
cg18932798	INA	0.000117986	0.421816606
cg13378934	MEIS3;MEIS3	1.96E-05	0.421806681
cg05716166	RALYL;RALYL;RALYL;RALYL;RALYL	0.000148457	0.421792767

cg03476860		8.60E-06	0.421789955
cg25105688		5.59E-05	0.421776214
cg00234691	SV2C	0.000117986	0.421765554
cg01058360	PRKAG2;PRKAG2	7.23E-05	0.421728336
cg12306156	THRB;THRB;THRB	0.000148457	0.421724123
cg01240056		4.34E-05	0.421723118
cg14457782	WNK4	2.58E-05	0.421701321
cg08641579	MAGI2	1.50E-05	0.421700719
cg05945059	RAX;RAX	9.24E-05	0.421660181
cg00419321	LHX8	2.08E-05	0.421642182
cg10836101	VSTM2B	8.60E-06	0.421637741
cg05547777		1.96E-05	0.421637524
cg26248075	SIM1	1.96E-05	0.421581864
cg03160466	OTOP1	0.000117986	0.421566098
cg05372113	CYP2A13	9.24E-05	0.421543294
cg00677986	PAWR	0.000233422	0.421532888
cg13940693	ADCYAP1;ADCYAP1	8.60E-06	0.421530765
cg24328125		8.60E-06	0.42151956
cg19089701		0.000148457	0.421458491
cg06374325	NKX6-1	1.11E-05	0.421451017
cg20140110	TMTC1	8.60E-06	0.42144554
cg26124980	ATOH1	8.60E-06	0.42141394
cg10617909		0.001948248	0.42140714
cg19021076	TMEM101	3.34E-05	0.421371657
cg25363885	PPP2R2B;PPP2R2B;PPP2R2B;PPP2R2B;PPP2	0.000117986	0.421366206
cg10591652	FBLL1	8.60E-06	0.421351399
cg19931348	PI3	1.11E-05	0.421344776
cg05619892	ZMAT4;ZMAT4	3.34E-05	0.421293333
cg04716990	MACROD2	9.24E-05	0.421279825
cg21200539	POU4F2	1.96E-05	0.421273932
cg08684893	ZNF225	0.001147425	0.421251825
cg19188182	LOC283392;TRHDE;LOC283392	1.50E-05	0.421251426
cg14312538	CDH2	0.000117986	0.421237823
cg14314744	SIM1	2.58E-05	0.421222664
cg07578695	SLC34A2	0.000290372	0.421172723
cg10827754		5.59E-05	0.421140203
cg24412079	GALNT13	8.60E-06	0.421126096
cg24992181	GOLSYN;GOLSYN;GOLSYN;GOLSYN;GOLSYN	0.000187194	0.421117815
cg09662034	TFAP2C	3.34E-05	0.421113883
cg18759960	PTF1A	3.34E-05	0.421102578
cg25025545	CD83;CD83	7.23E-05	0.421079542
cg09342997	NRF1;NRF1	7.23E-05	0.421069284
cg19403014	LHCGR	1.50E-05	0.421061792
cg24691336		1.50E-05	0.421017721
cg15839448	SFRP1	5.59E-05	0.421016897
cg11935248	CDYL;CDYL;CDYL;CDYL	0.000357885	0.421007972
cg04136610	ADAMTS16	0.000117986	0.421002009
cg06684850	BDNF;BDNF;BDNF;BDNF;BDNF	4.34E-05	0.420963143
cg19721867	SLC18A2	0.00043997	0.420949739
cg03062549		4.34E-05	0.420938135
cg09550083	MEIS1	0.000148457	0.420929745
cg20618622	NPY5R	4.34E-05	0.420918856
cg07489890	NLGN1	8.60E-06	0.420859288
cg09169320		0.000117986	0.420829002
cg22066180		1.50E-05	0.420815729
cg14731653	CRYBA2;CRYBA2;CRYBA2	4.34E-05	0.420796909
cg02952913	ZNF124	0.000148457	0.420793908
cg06452665	TNFSF11;TNFSF11;TNFSF11	0.000148457	0.420766376
cg24196693	PCDH21	8.60E-06	0.420711948
cg11483915	SLC40A1	2.58E-05	0.42070567

cg10639440	SCRN1;SCRN1;SCRN1;SCRN1	0.001376172	0.420702335
cg01581018	ZIC4;ZIC4;ZIC4;ZIC4;ZIC4	8.60E-06	0.420694379
cg00796963	KIAA1009	9.24E-05	0.420689558
cg27442308	SOX21	0.000148457	0.420686332
cg06596654		1.50E-05	0.420658791
cg11089595	RALYL;RALYL;RALYL	1.11E-05	0.420654233
cg20804700	MCOLN2	0.000537905	0.420645602
cg09563216	C1orf51;C1orf51	2.58E-05	0.420639138
cg04637478	PCDHGA4;PCDHGA1;PCDHGA6;PCDHGA5;PC	1.50E-05	0.420619247
cg26699292		1.96E-05	0.42060761
cg23710218	MSC;MSC	2.58E-05	0.420599069
cg25295727		0.000148457	0.420591875
cg01012089		8.60E-06	0.420586366
cg15133448	GPR120	8.60E-06	0.420585485
cg18801691	DCC	1.50E-05	0.420584824
cg06564900	FIGN	0.000187194	0.420563534
cg26808747	ADAM12;ADAM12	3.34E-05	0.420547154
cg13262687	POU4F2	1.50E-05	0.420540478
cg20554228	SCRN1;SCRN1;SCRN1;SCRN1	0.000290372	0.420526864
cg01972418	PAX9	7.23E-05	0.420509386
cg17090611	ZDHHC2	3.34E-05	0.420498435
cg05817517	CSMD2	3.34E-05	0.420493142
cg06399302	CHST8;CHST8	1.50E-05	0.420488615
cg26320696	PARVA	8.60E-06	0.420483342
cg00341742	RPRM	1.96E-05	0.420430377
cg06102602		0.00043997	0.42042776
cg05372730		9.24E-05	0.420418844
cg07931391		0.000187194	0.420404582
cg22782271	FUT9	8.60E-06	0.420384369
cg16421526	ARSJ	0.000148457	0.420366133
cg27492103	ASXL2	7.23E-05	0.420339234
cg12709880	NPC1	1.11E-05	0.420336465
cg01302656	C14orf23	9.24E-05	0.420335859
cg15106134	SOX2OT;SOX2	1.50E-05	0.42030543
cg23318063		2.58E-05	0.420286673
cg20598190		0.000537905	0.420262036
cg07676002	CDH22;CDH22	8.60E-06	0.420249461
cg17939295		2.58E-05	0.420244217
cg08347047	C12orf39	4.34E-05	0.420216087
cg15069906	BMP7	0.000357885	0.420201329
cg26345105		8.60E-06	0.420190476
cg00116092		2.58E-05	0.420189475
cg04293733	MIR137	4.34E-05	0.420183781
cg11284316	UNC80;UNC80	0.000117986	0.420183713
cg07394446	ADAMTS17	0.000148457	0.420171509
cg17228942	NLRP14;ZNF214;NLRP14	0.000117986	0.42015484
cg21953346	CADPS2;CADPS2;CADPS2	0.000537905	0.420141052
cg26070319		1.50E-05	0.420134061
cg02225716	MAL2	8.60E-06	0.420133987
cg10464312	MEIS1	1.50E-05	0.420120636
cg05871694	HOXD1;HOXD1	1.50E-05	0.420117837
cg23016129	SPAG6;SPAG6	0.000117986	0.420100772
cg23839680	CCT6A;PSPH;CCT6A	0.000205664	0.420097566
cg05822100	CDH6	1.96E-05	0.420093338
cg11940177	PGAM1	3.34E-05	0.420066379
cg23670353	FGGY;FGGY	0.001147425	0.420031551
cg11787785	NEBL	0.000654035	0.420016307
cg26802289		0.000262362	0.420002786
cg14530233	XKR6	0.000148457	0.419985273
cg11755405	KCNH5;KCNH5;KCNH5	0.000148457	0.419966664

cg12387713	MSX2	1.60E-05	0.419951786
cg22324567	EBF2	8.60E-06	0.419931545
cg03250019	LOC642597;LOC642597	3.34E-05	0.419918828
cg26959358	RUNX1T1	1.50E-05	0.419913152
cg09497201		0.000187194	0.419883133
cg11708136		1.50E-05	0.419882102
cg07345999		1.50E-05	0.419841947
cg13243169	GRID2	1.96E-05	0.419835495
cg00626110	NKX6-2	1.18E-05	0.419834331
cg07691705	MNX1;MNX1	8.60E-06	0.419825586
cg13146839	LOC647309;LOC647309	0.000290372	0.419811477
cg00400733		2.58E-05	0.419799123
cg03850957	SNRPB2;SNRPB2	5.59E-05	0.419791117
cg15602074	HTR1B	8.60E-06	0.41976899
cg08969532	CRTAC1;CRTAC1	5.59E-05	0.419762453
cg21276413	GABRA5;GABRA5;GABRA5	0.000654035	0.419759998
cg15085791		4.34E-05	0.419746715
cg01806520		9.24E-05	0.419721742
cg22190437	SPHKAP;SPHKAP	5.59E-05	0.41967695
cg04151278	GPR149	1.50E-05	0.419670075
cg12217987	UGT8	5.59E-05	0.419654096
cg09841889		0.000187194	0.419639945
cg05927068	THEM4	5.59E-05	0.41962487
cg06484146	VWDE	2.58E-05	0.419616279
cg13468418		1.50E-05	0.419589487
cg02004641	PCDHA7;PCDHA12;PCDHA6;PCDHAC1;PCDH	5.59E-05	0.41956398
cg22437405		1.96E-05	0.419546741
cg14317285	SOX2OT	2.58E-05	0.419530104
cg12839593	SIX1	8.60E-06	0.419522961
cg21792134	IQCB1;IQCB1	2.58E-05	0.419518318
cg11139646	BMP8B	0.000117986	0.419507423
cg03012170		8.60E-06	0.419502377
cg14654533	NUDT16P;NUDT16P	3.34E-05	0.419468494
cg01787574	LMO3;LMO3	8.60E-06	0.419467765
cg08592761	LARP6;LARP6	1.96E-05	0.419463195
cg24988625	EDIL3	8.60E-06	0.419441844
cg01213381	OTX2	7.23E-05	0.419428463
cg04002608	CXCL14	8.60E-06	0.419414893
cg14880228	PCDP1	1.96E-05	0.419353787
cg02098029		1.50E-05	0.419342514
cg18652346		9.24E-05	0.419331728
cg10994511	ADCY2	7.23E-05	0.419326392
cg15029631	SCN8A	2.58E-05	0.419319385
cg21688264	SNAP91;SNAP91	0.000791389	0.419318552
cg10876767	SORCS2	3.34E-05	0.419281215
cg17276590	EMX2OS;EMX2;EMX2	0.000187194	0.419275547
cg00067824		1.96E-05	0.419267391
cg02800810	ADAMTS5	8.60E-06	0.419239435
cg15090005	RBP7	1.11E-05	0.419227129
cg15728256	BMP8B;BMP8B	9.24E-05	0.419205509
cg15471177	PARD3B;PARD3B;PARD3B	4.34E-05	0.419186595
cg22513924	NMNAT2	9.24E-05	0.419183746
cg09462808	SYNPR	3.34E-05	0.419166027
cg26885268		8.60E-06	0.419144458
cg12475879		1.50E-05	0.41910235
cg27109600	FLJ32063	0.000122184	0.419099313
cg08400494	CARS2	7.23E-05	0.419073299
cg17393267	FGF12;FGF12	1.50E-05	0.419069058
cg23668285	SOX1;SOX1	4.34E-05	0.419060216
cg15425280	GRIA2;GRIA2;GRIA2	8.60E-06	0.419016912

cg01100912	EFNA5	0.000187194	0.419016819
cg23589035	ASH1L	0.000148457	0.419001113
cg10644072	DSCAM	8.60E-06	0.41899562
cg12743978	CHODL	8.60E-06	0.418989663
cg09048251		0.000233422	0.418970124
cg04264042	ATP6V0A1;ATP6V0A1;ATP6V0A1	8.60E-06	0.418967118
cg06272543	LOC440925;SP5	0.000148457	0.418957562
cg24857620	NPBWR1	0.000654035	0.418957029
cg12586150	SERPINB1	0.000357885	0.418943384
cg10028625	ECT2	9.24E-05	0.418931029
cg23662142		4.34E-05	0.418904693
cg08368654		3.34E-05	0.418855022
cg08279189	TRIM27	4.34E-05	0.418850648
cg17487050	RUNX1T1	8.60E-06	0.418836548
cg20704555	JAZF1	1.96E-05	0.418805934
cg27510066	CSGALNACT1;CSGALNACT1	1.96E-05	0.418785062
cg16695576		5.59E-05	0.41878487
cg24420366		2.58E-05	0.418782893
cg05872306	FOXG1	8.60E-06	0.418775755
cg07333715	ASCL2	0.003734675	0.418705333
cg18867200		3.34E-05	0.418696767
cg16509851		0.000148457	0.418691581
cg08535260		0.000158823	0.418684577
cg15978039	MEIS1	1.50E-05	0.418665186
cg26177213	FOXK1	9.24E-05	0.418662777
cg00325750		0.000290372	0.418656293
cg05297121	NPNT	3.34E-05	0.418624103
cg08017326	CXCL2	0.000233422	0.41861304
cg10453758	UBA5;UBA5;ACAD11	7.23E-05	0.418609056
cg09124223	DPP6;DPP6	1.50E-05	0.418595068
cg24466241	ZYG11A	1.50E-05	0.41858885
cg26813646	SLC18A3;CHAT	5.59E-05	0.41858163
cg13558403	NKX2-1;NKX2-1	0.000290372	0.418550669
cg12547166	ZNF876P	8.60E-06	0.418543336
cg14899716	RIMKLA	0.000187194	0.418540913
cg08358166		0.000117986	0.418537249
cg00266920	ST8SIA3	0.000290372	0.418528293
cg14154651	ESRP1;ESRP1;ESRP1;ESRP1;ESRP1	0.000654035	0.41850732
cg22195627		4.34E-05	0.418495068
cg06232807	SALL1;SALL1	2.58E-05	0.418489522
cg01276475	RIMS1	1.50E-05	0.418488833
cg15585318	WNT5A	1.11E-05	0.418488575
cg09515947	CDH8;CDH8	4.34E-05	0.418483948
cg26322865	LPPR2;LPPR2;LPPR2;LPPR2	5.59E-05	0.418474648
cg15873301	SYN2;SYN2	5.59E-05	0.418468058
cg21784383	ESRRG	2.58E-05	0.418444532
cg06122635		5.11E-05	0.418427896
cg11005250		8.60E-06	0.418417464
cg04651781		1.50E-05	0.41837535
cg10300684	FOXG1;FOXG1	1.96E-05	0.418372022
cg14507337	SIX6	0.000654035	0.418361207
cg19132462	AMPD3;AMPD3;AMPD3	4.34E-05	0.418357118
cg07673230	LAPTM4B	1.50E-05	0.418348449
cg00682263	MEGF11	0.001639597	0.418341713
cg10059810	PLEK2	0.000187194	0.418339795
cg23250574		0.000233422	0.418311763
cg12668482	TRPA1;TRPA1	8.60E-06	0.41831101
cg03759346		5.59E-05	0.418284726
cg05618934		5.59E-05	0.418256681
cg16651347	TNN	5.59E-05	0.418240728

cg05588496	DOK6	8.60E-06	0.418238741
cg14175690	TBX15	0.001639597	0.41823861
cg20424531		2.58E-05	0.418184756
cg05009934	CLVS2	8.60E-06	0.418177225
cg20091795		0.00043997	0.418176333
cg13210467	STAG3;GPC2	2.58E-05	0.418172581
cg19478500	STL	0.00043997	0.418168069
cg03473387	GATA6	0.0050758	0.418161706
cg11258164	KCTD8	0.000233422	0.418161478
cg14835443		3.34E-05	0.418132694
cg22300957	PHOX2B	8.60E-06	0.418113477
cg24830738	KCNN2;KCNN2	1.96E-05	0.418103721
cg19952303	SLC6A15;SLC6A15;SLC6A15;SLC6A15;SLC6A	2.58E-05	0.418101319
cg17080697	TRIM39;TRIM39	4.34E-05	0.418083232
cg06222851	OGDHL;OGDHL;OGDHL;OGDHL;OGDHL;OGD	8.60E-06	0.418068625
cg24610231		1.11E-05	0.418068331
cg11469098	EFCAB1;EFCAB1;EFCAB1;EFCAB1;EFCAB1	3.34E-05	0.418005345
cg18733974	PHF21B;PHF21B	1.11E-05	0.417972712
cg24239329	DCLK1	4.34E-05	0.417963397
cg10118210	FAM155A	2.58E-05	0.417947631
cg01757312	SOX1	0.000357885	0.417935856
cg00939735	HELT	4.34E-05	0.417931691
cg26463328		1.96E-05	0.417929915
cg00083059	MOCS1;MOCS1	1.11E-05	0.417909534
cg13600489	NKX2-1;NKX2-1	2.58E-05	0.417891622
cg04784315	GPR21;RABGAP1	7.23E-05	0.417889585
cg12615165	GATA6	3.34E-05	0.417885919
cg22127570		1.11E-05	0.41787185
cg15508809		0.00043997	0.417846283
cg24539234	TPBG;TPBG	0.0050758	0.417830054
cg14944944	UPK3A;UPK3A	0.000290372	0.417808312
cg15979173	BARHL2	1.50E-05	0.417805735
cg10948929	PTPRZ1	2.58E-05	0.417800621
cg24599434	GHSR;GHSR	8.60E-06	0.417791944
cg24165638	AZU1	2.58E-05	0.417770703
cg10071824	GSX1	0.000117986	0.417769429
cg17537073		0.000537905	0.417764492
cg15814717		1.50E-05	0.417757884
cg06046431	BDNF	3.34E-05	0.417742831
cg19081101	CHI3L1	2.58E-05	0.417731857
cg09186006	SLC16A12;SLC16A12	0.000357885	0.417730175
cg13346411	CCKBR	1.11E-05	0.417725552
cg01759562		1.11E-05	0.417709208
cg23480619	KCNE1;KCNE1;KCNE1;KCNE1;KCNE1	1.96E-05	0.417705632
cg17870270	LCA5;LCA5	0.000334462	0.417686726
cg17953764	ZAR1	8.60E-06	0.417668003
cg19591056	FMN2	7.23E-05	0.417655189
cg05478291	GOLSYN;GOLSYN;GOLSYN;GOLSYN;GOLSYN	0.000187194	0.417644397
cg25793377	RBP4	1.11E-05	0.417643138
cg19629292	FEZF2;FEZF2	0.000148457	0.417612531
cg22236250		2.58E-05	0.417570458
cg25412831	BDNF;BDNF;BDNF;BDNF;BDNF;BDNF;BDNF	0.000308169	0.417545285
cg08628584	SLC35F1	1.50E-05	0.417543586
cg11764900		1.96E-05	0.417532696
cg10667895		1.50E-05	0.417468647
cg03790804	LOC348840	0.000955793	0.41745918
cg24501381	CCDC30	3.34E-05	0.417451903
cg17188046	T	1.50E-05	0.417420173
cg18245660	RGS20;RGS20;RGS20	5.59E-05	0.417374356
cg15964132	OSBPL5;OSBPL5;OSBPL5	1.96E-05	0.417369228

cg22148695	SMOC2;SMOC2	2.58E-05	0.417367916
cg16407471	MIR129-2	8.60E-06	0.417349335
cg22976224	SIM2;SIM2	0.001376172	0.417320497
cg26425256		2.58E-05	0.417315514
cg25176746	MYF6	1.11E-05	0.417269651
cg06602847	DBC1;DBC1	4.34E-05	0.417255702
cg17295225	OLIG3	8.60E-06	0.417246142
cg22833612	ASAP2;ASAP2	3.92E-05	0.417241601
cg14638883	T	9.24E-05	0.417233795
cg03625260		0.000187194	0.417219705
cg02511156	LOC283392;TRHDE;LOC283392	4.34E-05	0.417215935
cg10091335		4.34E-05	0.417214427
cg03876032	MYO3A	0.000187194	0.417189872
cg16485975		5.59E-05	0.417185302
cg25773344	LRIG3;LRIG3;LRIG3	1.50E-05	0.41715879
cg06100973	ELANE	7.23E-05	0.417157296
cg04218812		5.59E-05	0.417122006
cg11428482	PHF21B;PHF21B	7.23E-05	0.417111428
cg11831238	DBX1	2.58E-05	0.417063278
cg20073686	GRIA4;GRIA4;GRIA4;GRIA4	9.24E-05	0.417047149
cg03732087	AGBL4	7.23E-05	0.417044484
cg00333824	CDH7;CDH7;CDH7	8.60E-06	0.417007817
cg24556441	SYN3	1.50E-05	0.417002486
cg13452317	ARHGAP8;ARHGAP8;PRR5-ARHGAP8;ARHGA	7.23E-05	0.416978281
cg26299169	PDX1	3.34E-05	0.416935837
cg20156659	LHCGR;LHCGR	8.60E-06	0.416925357
cg02595760		2.58E-05	0.41692212
cg02328332	LHX5	5.59E-05	0.416916766
cg13790603	THRB;THRB;THRB	0.000233422	0.416901798
cg09130556	CYP1B1	0.000955793	0.416878752
cg20670075	LHX5;LHX5	1.50E-05	0.416846279
cg12065138	AJAP1;AJAP1	1.50E-05	0.416844916
cg07070341	PRDM5	2.58E-05	0.416832606
cg04425920		0.000791389	0.416831495
cg23289079	PRDM6	2.80E-05	0.41682786
cg02853355	FAM92B	5.59E-05	0.416820316
cg14558529		0.000290372	0.416801395
cg26459372	ADCY1	0.000233422	0.416780318
cg11334771	PHOX2A	4.34E-05	0.416757287
cg07788369	DLX6AS	4.34E-05	0.416744025
cg16203203	STL	0.001222233	0.416740408
cg23244253	SATB2	0.000233422	0.416715043
cg00143045	RPRM	1.96E-05	0.416696953
cg15902390	MEIS2;MEIS2;MEIS2;MEIS2;MEIS2;MEIS2;MEI	0.000187194	0.416678433
cg22825487	VNN3;VNN3	0.000117986	0.416678313
cg08017858	P4HA3	4.34E-05	0.416674175
cg13604337		0.001147425	0.41658833
cg14312063	CADM2;CADM2	0.000187194	0.416584427
cg24013620	MAPK15	7.23E-05	0.416573385
cg07766263	SLC7A14	2.80E-05	0.416548388
cg26593946	NR2F2	9.24E-05	0.416520844
cg10305451		0.000117986	0.416518074
cg05371578	ZIC1	8.60E-06	0.416513979
cg00481644	LOC254559	1.50E-05	0.416467561
cg08078694		9.24E-05	0.416467516
cg04937080	DAB1	2.80E-05	0.41646014
cg05830425		0.000117986	0.416428406
cg06551697		1.96E-05	0.416420194
cg16955889	SOX2OT	2.08E-05	0.416403604
cg03229780		1.96E-05	0.416394359

cg16409955	TRPC4;TRPC4;TRPC4;TRPC4;TRPC4;TRPC4	8.60E-06	0.416379254
cg10320659		1.50E-05	0.416374551
cg06588201		5.59E-05	0.416293454
cg23845450	LHX8	1.11E-05	0.416282461
cg00721315	LHX5	0.000117986	0.416281858
cg25756066	DDAH1;DDAH1	0.000290372	0.416273491
cg08604097	NXPH1	0.000357885	0.416270247
cg21014159	DSCAML1	0.000117986	0.416266392
cg20874785	LOC100189589	3.34E-05	0.416241712
cg07892422	HOXC13	2.58E-05	0.416209954
cg05620980		0.000290372	0.416205758
cg12191293	GLS2	1.96E-05	0.416205384
cg06523224	BNC1	9.24E-05	0.416190924
cg16197925	FEZF1;FEZF1	2.58E-05	0.416166626
cg05976481		2.58E-05	0.416163516
cg07160277	MARCH1	1.96E-05	0.416134043
cg25771113		0.001376172	0.416111774
cg16896079	PDZRN4	0.000357885	0.416109945
cg13323756		3.34E-05	0.416108746
cg01345315	ADAMTS20	0.000148457	0.416096992
cg26067203	KIAA1751	0.000357885	0.416069453
cg17176573	POU2F3	0.000187194	0.416028182
cg20041105	LYNX1;LYNX1;LYNX1;LYNX1;LYNX1;LYNX1	0.000148457	0.416016792
cg22823644	HPSE2;HPSE2;HPSE2;HPSE2	0.000475639	0.415988756
cg21180599	TLE6;TLE6	1.50E-05	0.415982321
cg06787731		1.50E-05	0.415981592
cg07379703	ARSB;ARSB	1.96E-05	0.415971377
cg04986579	UNC13A	8.60E-06	0.415940478
cg08244959	IGSF21	4.34E-05	0.415923437
cg02227623	CTNNA2;CTNNA2	2.58E-05	0.415918776
cg04560225		2.58E-05	0.415915203
cg15680288		0.000955793	0.415901186
cg06157334	WNT6	0.00043997	0.415820475
cg26271891	ZNF385B;MIR1258	9.24E-05	0.415811533
cg13834844		0.000148457	0.415795336
cg20803857	ST6GAL2;ST6GAL2;ST6GAL2	5.59E-05	0.41579505
cg15469709	C1orf161	1.96E-05	0.415782649
cg07055616	NKX6-2	8.60E-06	0.415759227
cg26238041	MTNR1A	1.50E-05	0.415745759
cg05102394	EDNRA;EDNRA;EDNRA	1.11E-05	0.41570482
cg23641145	PGR	2.58E-05	0.415703643
cg16664584		5.59E-05	0.415700113
cg16060486	CYP1B1	0.000187194	0.415694282
cg06885823		0.000117986	0.415685607
cg27626102	STEAP2;STEAP2	1.50E-05	0.415664518
cg19380001	DMRTA2	2.58E-05	0.415653681
cg20359994		1.11E-05	0.415614331
cg01487187	CCKBR	1.96E-05	0.415607523
cg13267264	PRDM14	0.000117986	0.415604881
cg22160121	ESYT3	1.11E-05	0.415592463
cg12058385	ARHGAP15	5.59E-05	0.415589997
cg15368905	HTR1B	3.34E-05	0.415535704
cg21854408		1.50E-05	0.415527102
cg06479604	GABRA1;GABRA1;GABRA1;GABRA1;GABRA1	1.50E-05	0.415486048
cg02830749	KIAA0922;KIAA0922	0.000537905	0.415474501
cg27315191	SYT10	8.60E-06	0.415471669
cg20897830		4.34E-05	0.415464149
cg10739095	CLSTN2	7.23E-05	0.415426026
cg17454831	MEIS1	1.96E-05	0.415421209
cg07625131	USH1C;USH1C;USH1C;USH1C	1.96E-05	0.415417281

cg27331524	WNT2;WNT2;WNT2	1.50E-05	0.415401507
cg26989202	C8orf47;C8orf47;C8orf47;C8orf47	0.000357885	0.415348363
cg25670685	NALCN	0.000233422	0.415272696
cg16924702	EPB41L3	0.000334462	0.415272076
cg20482521	SORCS3	4.34E-05	0.415258814
cg15404858	NKX2-2	1.11E-05	0.415250478
cg22178238	PTPRN2;PTPRN2;PTPRN2	2.58E-05	0.415213252
cg04747180	KLHL5;KLHL5;KLHL5;KLHL5	3.34E-05	0.415208711
cg17152757	GHSR;GHSR	1.50E-05	0.415132462
cg23934731		2.58E-05	0.415124215
cg12930392	PAK2	3.34E-05	0.415113429
cg05454562	WDR46;WDR46	1.50E-05	0.415089955
cg07545317		1.50E-05	0.415082546
cg07211972	CBS	3.34E-05	0.415065788
cg24265969	ERC2	9.24E-05	0.41505938
cg07570573		0.000187194	0.415058365
cg24415208	CACNG8	7.23E-05	0.415053942
cg19814981		1.96E-05	0.415053932
cg17156803		1.11E-05	0.415049605
cg20557595		8.60E-06	0.41504893
cg17214381	ZFP42	0.000148457	0.415014071
cg26885517		0.000117986	0.415002992
cg03827337	RGS7BP	1.50E-05	0.414999811
cg09393254	MCHR2;MCHR2	0.000187194	0.414970946
cg03992259	NCKAP5;NCKAP5	1.11E-05	0.414962745
cg23571812	FOXG1	3.34E-05	0.414946781
cg26648818	TOX3;TOX3	8.60E-06	0.414923848
cg22147660		2.58E-05	0.414905015
cg07563793	LHX8	2.58E-05	0.414900653
cg13449778	FAM163A;FAM163A	2.58E-05	0.414900405
cg05933904	SOX8	0.000233422	0.414868728
cg06743610	FXYD1;FXYD1	0.000148457	0.414866664
cg13526469		0.000187194	0.414866339
cg05667379	DPP10;DPP10	2.58E-05	0.414863848
cg06759058		0.000148457	0.414863153
cg19731044		5.59E-05	0.414859098
cg08840010	TNFRSF9	5.59E-05	0.414829514
cg18171240	CDH2	3.34E-05	0.414823859
cg07465387		0.000290372	0.414813914
cg19356191		0.000233422	0.414790661
cg04617755	DPP10;DPP10	0.000187194	0.414788616
cg19815904	SEMA3C;SEMA3C	9.24E-05	0.414787357
cg24766821		1.96E-05	0.414785652
cg20698769	CTTNBP2	8.60E-06	0.414765044
cg02624770	TRIL	0.000122184	0.414758253
cg17098147	SPAG6;SPAG6	1.50E-05	0.41474542
cg06991300	QRFPR	1.96E-05	0.414743787
cg22796353		2.58E-05	0.414715466
cg07002201	FUT9	8.60E-06	0.414708964
cg18559579		1.96E-05	0.41467708
cg03775422	VSTM2A;VSTM2A	1.50E-05	0.414676838
cg26980033	WWC1;WWC1;WWC1	0.000148457	0.41465183
cg14768785	GHSR;GHSR	7.23E-05	0.414643795
cg13400248		1.96E-05	0.414630524
cg09648315	MAST4;MAST4	0.004131428	0.414617768
cg25286482	PDPN;PDPN	0.000205664	0.414590575
cg02081006	PRDM6	9.24E-05	0.414578427
cg14560001		4.34E-05	0.414576204
cg12209946		0.000117986	0.414563941
cg04419610	GRIN2D	0.000233422	0.414548188

cg16174681	FER	7.23E-05	0.414527548
cg03239552	HOXC13	0.000122184	0.414520885
cg26959655	UBR5	1.96E-05	0.414518992
cg12277666	TDRD5	1.96E-05	0.414512785
cg15920867	ERC2	0.000290372	0.41445974
cg05733181		5.59E-05	0.414443555
cg20950167	LHX1	8.60E-06	0.414419102
cg05523911	TCHH	9.24E-05	0.414391722
cg00956199		5.59E-05	0.414391389
cg00468146	ID4	0.000233422	0.414380689
cg15509687	GRP;GRP;GRP	2.58E-05	0.414380255
cg17231999		0.00043997	0.41436774
cg03444245		4.34E-05	0.41436435
cg06638827	LRIG3;LRIG3	8.60E-06	0.414360215
cg14003035	KIAA1755	0.001887355	0.414354636
cg25589001	LCOR;LCOR;LCOR	1.50E-05	0.414352784
cg16202564	MATN2;MATN2	1.50E-05	0.414323416
cg04200224	SCGN	8.60E-06	0.414313354
cg11022432	ALDH1L1	0.00043997	0.414279045
cg20328736	SCIN	2.58E-05	0.414253345
cg17892178	GRM7;GRM7;GRM7;GRM7	9.24E-05	0.414248194
cg15021439	OBSL1	1.11E-05	0.414241646
cg10828337	FOXG1;FOXG1	3.34E-05	0.414241024
cg01839464	DCC	8.60E-06	0.414193742
cg00773459	TTLL7;TTLL7	2.58E-05	0.414187936
cg03384992	OSBPL1A;OSBPL1A	0.001061731	0.41417523
cg07602980		0.00043997	0.41415417
cg08244848	EGLN3	0.006823935	0.414122797
cg01800521		3.34E-05	0.414085709
cg01926877		9.24E-05	0.414077525
cg10763234		0.00043997	0.414058846
cg14120010	CDH7;CDH7	8.60E-06	0.414057668
cg10206397	STK35	9.24E-05	0.414037293
cg11896633	FAM83H	4.34E-05	0.414017048
cg13689681	CDC14B;CDC14B	8.60E-06	0.413958684
cg03468072		5.59E-05	0.413942835
cg07098902	VSNL1	0.000187194	0.413932848
cg14655855		1.50E-05	0.413837044
cg00809988	ELAVL2;ELAVL2;ELAVL2	4.34E-05	0.413827189
cg21540437	GABRA2;GABRA2	1.11E-05	0.413818787
cg10767665	KCNN4	8.60E-06	0.413815333
cg04460771		0.000148457	0.413719971
cg19595234	TOX2;TOX2;TOX2	0.000654035	0.413699947
cg05020625	TTBK1	3.34E-05	0.413688844
cg23893898		1.96E-05	0.413663864
cg03032214	GALR1	8.60E-06	0.413663587
cg26249873	LOC100128811;GPR158	8.60E-06	0.413663491
cg16300637		2.80E-05	0.413650018
cg18028999	C15orf26	1.96E-05	0.413627308
cg26101277	CCL1	8.60E-06	0.413623592
cg10453230		0.000117986	0.41361047
cg12506971	AADAT;AADAT	2.58E-05	0.413605032
cg10794058	ALX1	5.59E-05	0.413595498
cg12488187	MSRB3;MSRB3	0.000654035	0.41358981
cg03709938	MMEL1	8.60E-06	0.413576071
cg21949333	PID1;PID1	0.000357885	0.413574422
cg24924779	KCNG1	0.000357885	0.413570552
cg15965134		8.60E-06	0.413568766
cg10108237	ESRRG;ESRRG;ESRRG;ESRRG;ESRRG	1.96E-05	0.413567258
cg14401466		8.60E-06	0.413560464

cg04949225		3.34E-05	0.413553147
cg24366557	TFAP2B	8.60E-06	0.413504208
cg06547386	MPPED2	0.000117986	0.413492845
cg06365535	TBX4	5.59E-05	0.413477274
cg26665224	SLC6A15;SLC6A15;SLC6A15	0.000117986	0.413462905
cg21998983	GALNT13	1.11E-05	0.413459057
cg16059957		0.000233422	0.413453608
cg03344031	MYH14;MYH14;MYH14	9.24E-05	0.413450936
cg03413097		1.50E-05	0.413420445
cg02928916	HTR1E;HTR1E	5.59E-05	0.413381826
cg00509610	TSEN2;TSEN2;TSEN2;TSEN2;TSEN2	5.59E-05	0.413375944
cg09450020	STEAP2;STEAP2	1.50E-05	0.413370739
cg25806808	CXCL1	1.11E-05	0.413356674
cg14323675		1.50E-05	0.413346008
cg04157138		8.60E-06	0.413335275
cg19239040		2.58E-05	0.413323816
cg26967100		0.000117986	0.413265437
cg05012825	PVR;PVR;PVR;PVR	1.96E-05	0.41324137
cg08718490	SPATA18	0.000117986	0.413226812
cg05486872	MYOM1;MYOM1	7.23E-05	0.413215849
cg21537214	REN	0.000187194	0.413207795
cg21842274	CRHBP	4.34E-05	0.413198415
cg12076463	NKX6-1	4.34E-05	0.413196673
cg25757598	RALYL;RALYL;RALYL;RALYL;RALYL	2.58E-05	0.413195189
cg25740565	FLJ32063	7.23E-05	0.413181869
cg12995800	GLRA1;GLRA1;GLRA1;GLRA1	3.34E-05	0.413179711
cg21186708		1.50E-05	0.413141914
cg15092343	MSX1	7.23E-05	0.413127987
cg21623566	DCC	8.60E-06	0.413069204
cg13920435	CDK20;CDK20;CDK20;CDK20;CDK20;CDK20;C	0.000187194	0.413063265
cg25911468	GLRB;GLRB;GLRB	2.58E-05	0.413049478
cg15688396	LSAMP;LSAMP	8.60E-06	0.413015011
cg18721420	CCDC105	2.58E-05	0.412983242
cg24750614	FAM123C;FAM123C;FAM123C;FAM123C	9.24E-05	0.412956889
cg26170604	NXPH1	7.23E-05	0.412932769
cg16396907		3.34E-05	0.412913226
cg10959353	SPHKAP;SPHKAP	5.59E-05	0.412883836
cg14953590	VGLL2;VGLL2	8.60E-06	0.412879489
cg27596495	HCRTR2;HCRTR2	1.11E-05	0.412874768
cg14334298	MOGAT1;MOGAT1	3.34E-05	0.41286353
cg11630154	SOX14;SOX14	1.96E-05	0.412807743
cg03012544	NEFM;NEFM	9.24E-05	0.412794853
cg23489137	RBMS1;RBMS1	0.000117986	0.412787925
cg21093192		1.50E-05	0.412754399
cg17333109	NKX2-8	1.50E-05	0.412752876
cg06695611	ZNF385B;MIR1258	0.000955793	0.412750604
cg21299542	PITX2;PITX2	8.60E-06	0.41274937
cg23037403	ZNF454	1.96E-05	0.412740697
cg08521987	SLC18A2	0.000290372	0.412740626
cg00481951	SST	8.60E-06	0.412706455
cg08620474	CYP1B1	0.00043997	0.41268499
cg25012577	ADAR	8.60E-06	0.412661317
cg08992911	MLPH;MLPH	2.58E-05	0.412647596
cg09053680	UTF1	0.000357885	0.412617492
cg19867649	DKK3;DKK3;DKK3;DKK3	5.59E-05	0.41260121
cg18237551	ZNF300	0.000791389	0.412591324
cg20981848	BTBD3;BTBD3	0.001376172	0.412563138
cg26129353	ATP8B1	3.34E-05	0.412556141
cg22418909	SFRP1;SFRP1	8.60E-06	0.412554965
cg19851909	GPR83	0.000117986	0.412550665

cg18447772	HOXA9	1.96E-05	0.412543632
cg06654956		9.24E-05	0.412542392
cg16822174	AEBP2;AEBP2	1.11E-05	0.412540706
cg13424673	AGBL4	1.11E-05	0.412539389
cg22837767	PAX1	2.58E-05	0.41253326
cg00859441	PLBD2;PLBD2	0.000187194	0.412531472
cg09805405	PKIB;PKIB;PKIB	1.50E-05	0.41248072
cg06526020	NUDT3	0.000233422	0.412471741
cg17508300	HPSE2;HPSE2;HPSE2;HPSE2	0.000357885	0.412464653
cg02002583		3.34E-05	0.412444105
cg23168531		3.34E-05	0.412444092
cg12790391	C11orf70	7.23E-05	0.412441899
cg13234863	TMEM132D	1.11E-05	0.412441304
cg26646867		0.000233422	0.412426151
cg26344227		0.000117986	0.412385226
cg08458292	DAB1	0.000117986	0.412303857
cg11580493	NR2F2;NR2F2	3.34E-05	0.412300237
cg26457809	KCNQ1DN	8.60E-06	0.412294147
cg26254792	HOOK1	8.60E-06	0.412288957
cg16852588	ZNF365;ZNF365;ZNF365	3.34E-05	0.412282031
cg02582387	FAM43B	0.000148457	0.412274691
cg08076830		1.50E-05	0.412272982
cg12426652	CLDN14;CLDN14	0.000654035	0.412256262
cg16104584	SLC2A5	0.000148457	0.41224719
cg25199552	KDM1A;KDM1A	1.50E-05	0.412183221
cg08199953	SALL1;SALL1	5.59E-05	0.412180502
cg08110488		0.000187194	0.412157166
cg03403065	PTPRT;PTPRT	0.000148457	0.412149848
cg01692233		1.11E-05	0.412130522
cg26114043		1.50E-05	0.41211892
cg21013866	EFS;EFS	9.24E-05	0.412116434
cg05372753		1.96E-05	0.412106213
cg07665387	PCDH10;PCDH10	1.50E-05	0.4120924
cg07380496	MAP1B	0.000117986	0.412091141
cg10846615		0.001135306	0.412087383
cg01822050		1.11E-05	0.412068451
cg08739233	SHISA4;SHISA4;SHISA4	0.000233422	0.41206751
cg21851351	GSX1	0.000187194	0.412061548
cg25615585	MAPK4	0.000537905	0.412058458
cg11836119	ZNF876P	1.50E-05	0.412019335
cg03406844	TNFAIP6	3.34E-05	0.411993567
cg09236284	TAC1;TAC1;TAC1;TAC1	7.23E-05	0.411990605
cg21902544	CBLN2;CBLN2	0.000187194	0.411984578
cg21024826	MNX1;MNX1	2.58E-05	0.411980709
cg23097402	DMRTA2	0.000357885	0.411967182
cg02579959	FLJ42289;FLJ42289	5.59E-05	0.411960771
cg09371439	ADAMTSL3	8.60E-06	0.411956804
cg14361409		2.58E-05	0.411948237
cg15558882		1.11E-05	0.411946244
cg02844545	GCM2;GCM2	5.59E-05	0.411940349
cg16263887		9.24E-05	0.411931732
cg08812189	ZIC4;ZIC4;ZIC4;ZIC4;ZIC4	1.96E-05	0.411931327
cg08943714	HECA	0.000654035	0.411900946
cg14614901	TMEM215	3.34E-05	0.411898903
cg18567924		0.000233422	0.411897408
cg20248516	EN1	1.50E-05	0.411880341
cg16882450		2.58E-05	0.411876356
cg12212453		2.58E-05	0.41185053
cg07944863	RGS7	8.60E-06	0.411850311
cg16536329	ZNF454	7.23E-05	0.411848886

cg09147985	LRRC4C;LRRC4C	1.50E-05	0.411801478
cg23208513	RGS22	8.60E-06	0.411798063
cg05204798	MPPED2;MPPED2	0.00043997	0.411779129
cg02115911		1.50E-05	0.411778581
cg05221057		0.000290372	0.411770121
cg27649239	LBXCOR1	0.000187194	0.411766376
cg22640233	OPCML	3.34E-05	0.411750182
cg17143696		0.000955793	0.411733286
cg13427728		2.58E-05	0.411714143
cg03022510		3.34E-05	0.411696626
cg01064265	IRX6	1.96E-05	0.411686416
cg26729217		9.24E-05	0.411674565
cg27546977		5.59E-05	0.411663211
cg23889730	PRIMA1	8.60E-06	0.411656299
cg20122727	BTC	0.000117986	0.411648201
cg19064258	HS3ST2	8.60E-06	0.411644742
cg19047868	LOC404266;HOXB5;LOC404266;LOC404266;L(	0.000117986	0.411619394
cg01236132	SOX1;SOX1	4.34E-05	0.411609777
cg06146698		5.59E-05	0.411594379
cg11505338	KDR	7.23E-05	0.411554587
cg18618429	CSTA	0.00043997	0.411491127
cg09650880		8.60E-06	0.411473753
cg26673012	TBX20;TBX20	1.96E-05	0.411464809
cg09671258	LHX4	9.24E-05	0.411415366
cg16406225		8.60E-06	0.41139742
cg07103129	TFAP2B	1.50E-05	0.411381438
cg11850773	ADCYAP1;ADCYAP1;ADCYAP1	1.50E-05	0.411379792
cg01110759	MT1A	0.000117986	0.411368594
cg22339356	NXPH1	3.34E-05	0.411351751
cg00413617	KCNC1;KCNC1	8.60E-06	0.411321926
cg25953239	HOXD10	7.23E-05	0.411307967
cg16142306	UCHL1;UCHL1	8.60E-06	0.411292542
cg26717554	ELANE	4.34E-05	0.411290374
cg17347634	CYP7B1	0.000148457	0.411286514
cg07410872	HAND2;NBLA00301	0.000187194	0.411285067
cg14156751		1.50E-05	0.411284222
cg08232264	FAM164A	0.000117986	0.411263133
cg13935962	HAND2;NBLA00301	0.000187194	0.411246438
cg01729717	SLITRK1;SLITRK1	7.23E-05	0.411233239
cg04037038	FRZB	0.002714607	0.411221802
cg23850899	LRRC4C;LRRC4C	1.50E-05	0.411211642
cg02938303	CDCP1;CDCP1	0.002714607	0.411202332
cg13165109	TDH	5.59E-05	0.411193594
cg27279991	AP3B2	1.96E-05	0.411192785
cg26625518		9.24E-05	0.411183467
cg06883126		0.000537905	0.411177431
cg14834285		4.34E-05	0.411126257
cg15272362	VSX1;VSX1	3.34E-05	0.411115967
cg07315010	MAP1LC3C	3.34E-05	0.411097608
cg14007757		1.60E-05	0.411063307
cg20596329		4.34E-05	0.411061613
cg12106728		3.34E-05	0.411061361
cg02367133		2.58E-05	0.411061092
cg27044841		8.60E-06	0.411057204
cg19712746	KDR	1.11E-05	0.411048906
cg02620694	GABRA2;GABRA2	1.11E-05	0.411033529
cg02168303		0.000117986	0.411001953
cg26235748	FLRT2	0.000537905	0.410981156
cg14900814	LGI2	0.000290372	0.410968512
cg04690482	FAM83B	4.34E-05	0.410928677

cg03724990	TBX18	1.50E-05	0.41090886
cg20405017	CA10;CA10;CA10;CA10	2.58E-05	0.410892898
cg12510028	CALCR;CALCR	4.34E-05	0.410891783
cg15261499		1.96E-05	0.410882769
cg24603464	MLPH;MLPH	8.60E-06	0.410869955
cg09467433		0.000357885	0.410863992
cg22040469	MYOD1	0.000117986	0.41084858
cg10318510	PRKD1	0.000187194	0.410831005
cg04834204	ANTXR1	5.59E-05	0.410765088
cg16247183		0.001147425	0.410731479
cg11283860		3.34E-05	0.410731413
cg13995774	MAML1	5.59E-05	0.410705447
cg04304802	CDH23	9.24E-05	0.410700348
cg25600606	HIPK3;HIPK3	1.96E-05	0.410697085
cg03475665	HHIPL1;HHIPL1	0.000537905	0.410689403
cg21187352	GRM7;GRM7	4.34E-05	0.410679487
cg22178798		5.59E-05	0.410672854
cg01105058	NALCN	4.34E-05	0.41065488
cg03156546	TNRC6A	0.000148457	0.410620066
cg21159568	CHST12	0.007873393	0.410609429
cg01142710		1.96E-05	0.410592749
cg08806408	SALL1;SALL1	0.000955793	0.410588064
cg00589371	CDH7;CDH7	1.96E-05	0.410587271
cg24133115	PDE10A;PDE10A;PDE10A;PDE10A	1.11E-05	0.410542454
cg00851770	CIDEA;CIDEA	0.000187194	0.410533007
cg10966582	MST1P9	1.11E-05	0.410520798
cg21062931	DHCR24	2.58E-05	0.410494441
cg08458487	SFTPD	0.000117986	0.410481677
cg04698114	SALL1;SALL1;SALL1	0.001948248	0.410471095
cg18881684		0.000148457	0.410460537
cg18739950		0.000290372	0.410442068
cg10126205	TWIST1	0.000357885	0.410415411
cg01166925		1.96E-05	0.410402633
cg06498267	HCN1	2.58E-05	0.410380563
cg11663667		1.96E-05	0.410370183
cg14003416		8.60E-06	0.410362679
cg05293994	CADPS2;CADPS2;CADPS2	0.000223017	0.410328408
cg14425564	LHX2	9.24E-05	0.410326184
cg19681956	RAB32	0.000117986	0.410320614
cg01920657	DNAJC6	7.23E-05	0.410296282
cg06793562		8.60E-06	0.410294668
cg23811789	LOC642597	3.34E-05	0.410282945
cg24553417	EBPL	2.58E-05	0.410278589
cg13016179	PLSCR4;PLSCR4;PLSCR4;PLSCR4;PLSCR4	9.24E-05	0.41027728
cg04273811	STAC	0.000117986	0.41023975
cg25037730		1.11E-05	0.410230163
cg26597242	GABRA1;GABRA1;GABRA1;GABRA1;GABRA1	1.11E-05	0.410230034
cg12424785	GALNT13	8.60E-06	0.410228044
cg17918227	CADM1;CADM1	1.50E-05	0.410221426
cg05125795	LPHN2	4.34E-05	0.410215198
cg10752406	AZU1	2.58E-05	0.410207885
cg24395452	SPATA18	1.50E-05	0.410187623
cg12685846	TJP1;TJP1	1.96E-05	0.410174566
cg16764494	DSCAML1	0.000290372	0.410161289
cg25734490	ASCL1	9.24E-05	0.410118314
cg22830113		0.001147425	0.410115273
cg10827893	PROX1	0.000955793	0.410081377
cg20449685	ZSCAN1	2.58E-05	0.410072014
cg27076454	AGAP1;AGAP1	0.00043997	0.41006914
cg18507018	TMEM55A	0.000117986	0.410063242

cg02694427	HOXD12	0.000233422	0.410042114
cg00625334	C18orf34;C18orf34	1.50E-05	0.410036309
cg10972973		0.000187194	0.410022445
cg27414655	PRDM5	5.59E-05	0.410002333
cg20674577	PHACTR3	8.60E-06	0.409993216
cg24996179		5.59E-05	0.409991004
cg02273398		7.23E-05	0.409990019
cg16494192	SLC18A3;SLC18A3;CHAT	1.96E-05	0.409979825
cg06202228		8.60E-06	0.409978585
cg19942495	ACCN1	2.58E-05	0.409977176
cg21384492	SNED1	9.24E-05	0.409975887
cg09051775	TOX3;TOX3	0.000233422	0.409965873
cg00149947		0.000148457	0.409950048
cg11842610	GFRA1;GFRA1	0.000187194	0.409940251
cg16157435	NPAS2	2.58E-05	0.409919173
cg10358533	CHST8;CHST8	5.59E-05	0.409908858
cg19174211	SASH1	2.58E-05	0.409904519
cg22892110	MAPK15	8.60E-06	0.409864901
cg20505223	SCTR;SCTR	4.34E-05	0.409844478
cg11849717	EGFR;EGFR;EGFR;EGFR	7.23E-05	0.409841724
cg22826333	CWH43	8.60E-06	0.409840984
cg02013838		9.24E-05	0.409838158
cg01178680	ISLR2;ISLR2;ISLR2;ISLR2	0.000187194	0.40983517
cg06829830	RASGRF2	0.000233422	0.409817976
cg21466150	RFX6	2.58E-05	0.409779949
cg02541778		1.96E-05	0.409777061
cg17264240	PDLIM3;PDLIM3	1.11E-05	0.409736346
cg21657955	TMEM196	4.34E-05	0.409690009
cg09454925	KCNH7;KCNH7	8.60E-06	0.409680984
cg20099830	TBX5;TBX5;TBX5;TBX5	1.50E-05	0.409654902
cg06829686		0.00043997	0.409646853
cg20930366	KCNN2	0.000334462	0.409645211
cg21874829		0.000233422	0.409634734
cg05195067	PAX2;PAX2;PAX2;PAX2;PAX2	0.000187194	0.409626728
cg03306221	SULT4A1	1.50E-05	0.409624713
cg11935021	COL25A1;COL25A1;COL25A1;COL25A1	8.60E-06	0.4096244
cg01971160	LHX8	8.60E-06	0.409619507
cg10972897	UCK2	7.23E-05	0.409614086
cg18525486	FEZF2	2.58E-05	0.409582628
cg19453938		5.59E-05	0.409565179
cg23715830		5.59E-05	0.40956328
cg02530022	DLX6AS	3.34E-05	0.409557119
cg23442672		1.96E-05	0.409547526
cg10193817	CADM1;CADM1;CADM1;CADM1	8.60E-06	0.409530027
cg00880018	TNFSF11;TNFSF11	0.000537905	0.409525421
cg10943458	STXBP5L;STXBP5L	0.000187194	0.40951414
cg17619311	ZNF662;ZNF662;ZNF662	4.34E-05	0.409505844
cg13734972	DOCK5	1.11E-05	0.409469614
cg00340958	B4GALT5	3.34E-05	0.409444012
cg06945523	SFTA3	0.000357885	0.409431638
cg18319029	ADCYAP1;ADCYAP1	0.000117986	0.409400193
cg23559689	GRIA4;GRIA4;GRIA4;GRIA4	0.000117986	0.409395857
cg25184481	TMEM196	5.59E-05	0.409373791
cg19494588	PPP2R2B;PPP2R2B;PPP2R2B;PPP2R2B;PPP2	5.59E-05	0.409353755
cg15503722	BSX	1.50E-05	0.409346833
cg14228146		7.23E-05	0.409345149
cg10157715		8.60E-06	0.409339136
cg08154963	RAD51L3;RAD51L3;RAD51L3	1.50E-05	0.409322117
cg06567373		5.59E-05	0.409306154
cg04961582		0.000148457	0.40929965

cg14626309		4.34E-05	0.409277906
cg02315940	CLSTN2	5.59E-05	0.409239464
cg06763054	MTMR7;MTMR7	1.50E-05	0.409226211
cg08990926	CNTN1;CNTN1;CNTN1;CNTN1	3.34E-05	0.40921165
cg17256532	GFRA1;GFRA1;GFRA1	0.000955793	0.409202567
cg09892426		0.000117986	0.409194349
cg04914562	INADL	0.000148457	0.409184345
cg25578609	WSCD1	0.000357885	0.40918212
cg18777119	VSTM2B	0.000187194	0.409172339
cg22727581	NEFL	5.59E-05	0.409169579
cg08495770	SORCS3	1.11E-05	0.409168365
cg04908380		4.34E-05	0.409167531
cg25739938	CPSF3	1.50E-05	0.409162664
cg04878955	RSPO1;RSPO1	0.000187194	0.409155977
cg10880928	C9orf86;C9orf86	1.11E-05	0.409144625
cg00995854	CD5L	0.000290372	0.409130451
cg20357628	PHACTR3;PHACTR3	7.23E-05	0.409125213
cg25895948	ALPK3	0.000233422	0.4091116
cg12843518	STOX2	4.34E-05	0.409101516
cg06739029	ID4	9.24E-05	0.409092308
cg14488905	NRG1;NRG1;NRG1;NRG1;NRG1;NRG1;NRG1;	0.000148457	0.409082506
cg04946561	ARRDC5	8.60E-06	0.40905651
cg00912625	CNTN4	8.60E-06	0.409010476
cg22460896		0.000233422	0.40900967
cg12391690	RSPO1	7.23E-05	0.40898794
cg14059339	TRIM27	7.23E-05	0.408976433
cg03930088		0.000791389	0.408975496
cg03699623		1.50E-05	0.408956451
cg07583894	RTBDN;RTBDN	7.23E-05	0.408930221
cg13563725		8.60E-06	0.408927556
cg02893823	KRT19;KRT19	5.11E-05	0.408905473
cg00374016		7.23E-05	0.408905351
cg18020065	RASA3	0.002304201	0.408894731
cg22327646		1.11E-05	0.408891548
cg02869649	PHF21B;PHF21B	4.34E-05	0.408885051
cg15414833	RUNDC3B;RUNDC3B;RUNDC3B;ABCB1;RUND	0.000187194	0.408881681
cg06545268		0.000233422	0.408874212
cg27635267	PAMR1;PAMR1	8.60E-06	0.408862881
cg11301556	SLC12A5;SLC12A5	7.23E-05	0.408818532
cg00626856		7.23E-05	0.408809651
cg19923650	NKX2-5;NKX2-5;NKX2-5	9.24E-05	0.408784318
cg08141424	PDZRN3	8.60E-06	0.408772834
cg23363202	ANKRD20B	8.60E-06	0.40876995
cg14564076	DPP6	8.60E-06	0.408766935
cg06641153	ST8SIA3	1.11E-05	0.408753751
cg04711050	SLC1A1	3.34E-05	0.408746937
cg20611680		8.60E-06	0.408739861
cg03437186	ADCY1	5.59E-05	0.408739652
cg16633951	LOC84856	1.96E-05	0.408727185
cg19623438		0.000290372	0.408711523
cg02564291	PRDM6	8.60E-06	0.408707124
cg24693053	MFSD7	4.34E-05	0.408700387
cg17412248	CDH22	1.11E-05	0.408689952
cg04274288		8.60E-06	0.408688644
cg21923525	RALBP1	8.60E-06	0.40868845
cg08276289		0.000233422	0.408682441
cg16284459	MTERF	0.005897668	0.408680163
cg04132917	RORA	0.000290372	0.408668852
cg02344527		0.000654035	0.408649988
cg15969227		3.34E-05	0.408637087

cg27540865		5.59E-05	0.408616908
cg20801476	EVX1	5.59E-05	0.408607752
cg05655534		1.11E-05	0.408575186
cg24537383	ROR1;ROR1	0.000290372	0.408569973
cg24513433	LIPG	0.000117986	0.408552188
cg21745612	TMEM132E;C17orf102	4.34E-05	0.40854758
cg24630419	ESRRG	3.34E-05	0.408529299
cg17519645	NCRNA00085	0.001147425	0.408486992
cg01154966	TRPC4AP;TRPC4AP	0.00043997	0.408478652
cg04553690	PCDHGA4;PCDHGA6;PCDHGA1;PCDHGA8;PC	1.96E-05	0.408471011
cg25023275	BRUNOL4;BRUNOL4;BRUNOL4;BRUNOL4	4.34E-05	0.408465519
cg17436134		5.59E-05	0.408458227
cg12754421	GRIA4;GRIA4;GRIA4;GRIA4	5.59E-05	0.408450136
cg27294268	SFTA3	1.11E-05	0.408449449
cg02515601	GLRA1;GLRA1	8.60E-06	0.408441518
cg01447112		4.34E-05	0.408436845
cg06465076	CAST;CAST;CAST;CAST;CAST	1.50E-05	0.408434971
cg16596102	FAM78B	7.23E-05	0.408431877
cg11391335	TBX15	8.60E-06	0.408413498
cg16662477	RFFL	5.11E-05	0.408373017
cg06951245	PTH2R	8.60E-06	0.408318827
cg17477445	DPY19L2	2.58E-05	0.408317429
cg04359129	PSD;FBXL15	7.23E-05	0.408311233
cg13561879	UNC5D	5.59E-05	0.408284916
cg05876738	CALCR;CALCR	2.58E-05	0.408273514
cg16502747	ACIN1;ACIN1;ACIN1;ACIN1;ACIN1	3.34E-05	0.408266868
cg03160135	VGLL2;VGLL2;VGLL2;VGLL2	8.60E-06	0.408252865
cg24771121	KDELC1;BIVM;KDELC1;BIVM	4.34E-05	0.408231898
cg16526416		0.000233422	0.408228413
cg04759648		1.11E-05	0.408220532
cg02631468	VSX1;VSX1	0.000148457	0.408218835
cg21784917	MAGI2	8.60E-06	0.408208544
cg03355524	CBLN4	5.59E-05	0.408168258
cg01561869	PCDHGA2;PCDHGA3;PCDHGA3;PCDHGA1	1.50E-05	0.408157249
cg11576590		0.000290372	0.408157122
cg22279027	PLA2R1;PLA2R1;PLA2R1;PLA2R1	0.000290372	0.408147298
cg06713632	KCNA7;KCNA7	5.59E-05	0.40809871
cg12988534	COBL;COBL	0.000148457	0.408089185
cg08214995	HS3ST2	8.60E-06	0.408085599
cg14058647		4.34E-05	0.408085071
cg24302235	SLC2A2	2.58E-05	0.408074569
cg16277479	PDPN;PDPN	7.23E-05	0.408063658
cg07072722	NTM;NTM;NTM;NTM;NTM;NTM;NTM	3.34E-05	0.408056272
cg11763509	BMP8A	3.34E-05	0.408038525
cg15089219	C1QL2	1.11E-05	0.40803232
cg03217173		0.000187194	0.408011464
cg13288164	PCDHB4	1.96E-05	0.408008637
cg01114937	TBX18	1.96E-05	0.408000608
cg19050990		8.60E-06	0.407999422
cg23679344	MED1	7.23E-05	0.407997519
cg01050010	MYO1D	0.000117986	0.407945801
cg03760951	INS-IGF2;IGF2AS;IGF2;IGF2;IGF2AS;IGF2	0.000205664	0.407923163
cg21303011	THRB;THRB;THRB	0.000357885	0.407915397
cg14733048	RSPO2;RSPO2	3.34E-05	0.407885219
cg18144593		0.000233422	0.407875472
cg22945605	ARHGEF10L	5.59E-05	0.407816996
cg01477971	TMEM182	0.000357885	0.407813846
cg25319067		5.59E-05	0.407794156
cg10820926	PRKD1	2.58E-05	0.407772418
cg03667047	KL	7.23E-05	0.407767894

cg03159124	LAMA1	0.000148457	0.407754584
cg16925459	HDAC9;HDAC9;HDAC9;HDAC9;HDAC9	2.58E-05	0.407725295
cg10237419	COL25A1;COL25A1	1.96E-05	0.407719882
cg01352175		1.11E-05	0.407718449
cg04085025	SCRN1;SCRN1;SCRN1;SCRN1	0.000357885	0.407717793
cg18400281	KIAA1217;KIAA1217;KIAA1217	0.000290372	0.40771412
cg13307880	PCDHGA4;PCDHGA2;PCDHGA5;PCDHGB2;PC	8.60E-06	0.40769468
cg10162019		3.34E-05	0.407684633
cg07393736		0.000117986	0.407678656
cg05903736	HDAC4	2.58E-05	0.40761003
cg19063061	SLC17A7	0.000537905	0.407605105
cg14971895	GPR158;LOC100128811	1.96E-05	0.407600278
cg09179211	FAM3B;FAM3B	5.59E-05	0.407578748
cg06219793	BHLHE22	8.60E-06	0.407563755
cg02345961	BRUNOL4;BRUNOL4;BRUNOL4;BRUNOL4	8.60E-06	0.407531488
cg19827223		1.50E-05	0.407519365
cg02279108		1.96E-05	0.407491822
cg00506629	PLOD2;PLOD2	1.96E-05	0.407485858
cg08393041	ZIC4;ZIC4;ZIC4;ZIC4;ZIC4	1.11E-05	0.407484357
cg09354241		0.000117986	0.407476582
cg07708622		0.000357885	0.407470485
cg13511000	ATOH1	1.50E-05	0.407469924
cg18920097	GJD2	9.24E-05	0.407466146
cg00124695	SALL1;SALL1	0.00041966	0.407460651
cg13010502	FAM123C;FAM123C;FAM123C;FAM123C	8.60E-06	0.407453048
cg06616729		0.000955793	0.407434959
cg25842602	BRUNOL4;BRUNOL4;BRUNOL4;BRUNOL4	8.60E-06	0.407433361
cg19304150	SYT6;SYT6	9.24E-05	0.407409223
cg03128890		4.34E-05	0.407407291
cg04837832		0.000654035	0.407387261
cg07739205	SNTG1	0.000148457	0.407386926
cg15645638	TMEM74	0.000262362	0.407372247
cg02319986	PRAM1	4.34E-05	0.407371928
cg08845123	KANK4	1.96E-05	0.407362141
cg19831575	FGF4;FGF4	5.59E-05	0.407333834
cg00610583	CXCL1	4.34E-05	0.407333737
cg17673897	TFAP2A	9.24E-05	0.407326976
cg10362591	SLC6A2	1.11E-05	0.407324879
cg17424999	SLC6A5	4.34E-05	0.407324125
cg07147449		9.24E-05	0.407316807
cg08004425	GRIK3	0.000357885	0.407308634
cg05384102	SNAP91;SNAP91	0.002040485	0.40729854
cg15069295		1.50E-05	0.407292869
cg09253179		4.34E-05	0.407292026
cg24813038		7.23E-05	0.407288105
cg21448057	SLITRK1	1.50E-05	0.407284105
cg03637218	AP3S1	6.94E-05	0.407269369
cg16563230	AJAP1;AJAP1	0.000955793	0.407247473
cg26105956	SHE	0.000117986	0.407240122
cg19968403	MIR548G;COL8A1;COL8A1	5.59E-05	0.407232583
cg00874051		0.000357885	0.407220621
cg03207054	CADPS;CADPS;CADPS	7.23E-05	0.407199398
cg23004758		0.000654035	0.407161405
cg06281795	CALB1	0.000117986	0.407148004
cg10131657	PKHD1L1	0.000117986	0.407107655
cg04266202	MPO	4.34E-05	0.407107067
cg24524470	EFCAB2;EFCAB2;EFCAB2	0.000526175	0.407095642
cg12771118	MRAS;MRAS	5.59E-05	0.407065775
cg17344770	C19orf71	2.58E-05	0.407053837
cg21328033	CA10;CA10;CA10;CA10	1.96E-05	0.407033982

cg07147033	MIB2;MIB2;MIB2;MIB2;MIB2	0.000233422	0.407006825
cg26258273	ESYT3;ESYT3	0.000233422	0.406975546
cg10640845	ONECUT3	2.58E-05	0.406972094
cg03203223	RELN;RELN	0.000148457	0.406940673
cg00666763	MAPKAP1;MAPKAP1;MAPKAP1;MAPKAP1;MA	0.000290372	0.406898047
cg15861196		2.58E-05	0.406874673
cg20764887	COL23A1	0.00043997	0.406865944
cg19594305	CHST8;CHST8;CHST8;CHST8	5.59E-05	0.406864801
cg02087954		2.58E-05	0.406829583
cg26385172		8.60E-06	0.406825567
cg08058191	KRT17	4.34E-05	0.40678515
cg03111498	VSX1;VSX1	1.11E-05	0.406779492
cg22972989	VSTM2A	0.000290372	0.406763581
cg04467618	TCF21;TCF21	9.24E-05	0.40676097
cg20523393		0.000290372	0.406760863
cg12204732	DOCK1;FAM196A	3.34E-05	0.406747524
cg11118422		1.11E-05	0.406726209
cg07108441	SORBS2;SORBS2;SORBS2;SORBS2;SORBS2	4.34E-05	0.406716776
cg07298177		1.11E-05	0.406702464
cg16101346	PTGS2	0.000357885	0.40669718
cg26333652	IRX2;IRX2	0.000148457	0.406637406
cg14169521	ARL4A;ARL4A;ARL4A	0.009066563	0.406625397
cg07356342	NDUFS2;NDUFS2;FCER1G	1.96E-05	0.40662397
cg06710937		4.34E-05	0.40661276
cg15957055	CADPS;CADPS;CADPS;CADPS;CADPS;CADP	0.000654035	0.406609743
cg07065111		0.007873393	0.406584606
cg08238215	MEIS1	1.50E-05	0.406583434
cg01851088	CELSR3;CELSR3	0.001147425	0.406577338
cg16240162	ZIC4;ZIC4;ZIC4;ZIC4;ZIC4	0.000117986	0.406570862
cg04988476	FGF12	1.50E-05	0.406551673
cg02561482	TFAP2B	1.11E-05	0.406548783
cg13413955	DMRT1	5.59E-05	0.406537395
cg12339328	SEC23B;SEC23B;SEC23B	1.96E-05	0.406537091
cg23095584	GBX2	1.11E-05	0.406529377
cg27113059	LIPG	0.000148457	0.406518479
cg17741839	PCDHA6;PCDHA2;PCDHA1;PCDHA10;PCDHA	1.50E-05	0.406518374
cg26267388		3.34E-05	0.406500268
cg00272971		2.58E-05	0.406480519
cg11880727	KCNH5;KCNH5;KCNH5	1.11E-05	0.406478958
cg03717836		8.60E-06	0.406474592
cg18947175	HRNBP3	2.58E-05	0.40646413
cg02458945	MMP2;MMP2	9.24E-05	0.40645852
cg20785796	SATB2	9.24E-05	0.406431653
cg16043651	ZNF536	0.000233422	0.406412507
cg21893651	NCRNA00171	0.000290372	0.40639615
cg15531512		0.000117986	0.406378216
cg00637104		0.000187194	0.406360292
cg15119027	FGF3	0.000117986	0.406356926
cg02547269	FOXF2	1.96E-05	0.406294372
cg14701491		0.00043997	0.406283117
cg08259514		0.000290372	0.406283113
cg12758687	DRD2;DRD2	1.96E-05	0.406254742
cg01946042		1.11E-05	0.406236366
cg24154951		3.34E-05	0.406213825
cg12704157		0.000117986	0.406208952
cg10179911		5.59E-05	0.40619453
cg01946451		8.60E-06	0.406191526
cg12075445	GALNT9	0.00043997	0.406184499
cg16377881	PRDM13	4.34E-05	0.406178353
cg02699167	FBXL2	0.000187194	0.406174036

cg24715245	UCHL1	7.23E-05	0.406148552
cg18511526	FLJ44817;FLJ44817	5.59E-05	0.406139975
cg03207439	C20orf103	0.000791389	0.406104238
cg12173558		3.34E-05	0.406089197
cg06529756	SV2C	7.23E-05	0.406082699
cg12174804		0.000187194	0.406081928
cg08979319	SIM1	1.96E-05	0.406055313
cg07272264		4.34E-05	0.406054044
cg17974166		7.23E-05	0.40604102
cg18789958	HCN1	0.000117986	0.40602162
cg27271486	DKFZP434H168;GNAO1;GNAO1	0.000117986	0.406005873
cg18510056	C10orf41;C10orf41	7.23E-05	0.406001381
cg05501357	HIPK3;HIPK3	1.11E-05	0.405986302
cg13121699	ZNF804A	1.50E-05	0.405983378
cg24331598		0.000187194	0.405975249
cg16420199	LPL	3.34E-05	0.40597062
cg23291280		2.58E-05	0.405964591
cg15377283	SOX17	3.34E-05	0.405963154
cg20540714	TFAP2A;TFAP2A	5.59E-05	0.405946825
cg24680602	ZNF232;ZNF232	8.60E-06	0.405943204
cg03427905	ADAMTS9	0.000290372	0.405939255
cg05106294	DKK3;DKK3;DKK3	0.000262362	0.405899145
cg22400420	RGL1	1.50E-05	0.405898144
cg20257553	PLA2R1;PLA2R1	0.000117986	0.405892123
cg02935904	TTC22;TTC22	3.34E-05	0.405879501
cg10585648	CCT3;C1orf182;C1orf182;CCT3;CCT3	0.000537905	0.405867816
cg15835339	SFTA1P	3.34E-05	0.405860121
cg03326762	LHX8	3.34E-05	0.405832899
cg06757399	HAGH;FAHD1;FAHD1;FAHD1;HAGH;FAHD1	0.000955793	0.405800513
cg16785912	PKP2;PKP2	1.50E-05	0.405773096
cg03870777	KRT18;KRT18	9.24E-05	0.405762455
cg09306214	TACR1;TACR1	9.24E-05	0.405753723
cg16573328	ZNF804A;ZNF804A	3.92E-05	0.405743487
cg22941573	TMEM189;TMEM189-UBE2V1;TMEM189;TMEM	0.000117986	0.405742025
cg06994692	LOC389458	0.000290372	0.405729963
cg24085946	NFASC;NFASC;NFASC;NFASC;NFASC;NFASC	8.60E-06	0.405728532
cg26269881	BHLHE40	0.000537905	0.405724064
cg20593611	FIGN	0.000791389	0.40571729
cg08734647	C16orf73;C16orf73	1.96E-05	0.405711125
cg17934871	C13orf36	0.000117986	0.405705385
cg15174623		1.11E-05	0.405702986
cg14144025		8.60E-06	0.405700088
cg26355577	CTNNA2;CTNNA2;LRRTM1	8.60E-06	0.405696166
cg04334723	CALR	5.59E-05	0.405683772
cg18267049		7.23E-05	0.405678416
cg09619271	IGFBP3;IGFBP3	0.000955793	0.405661348
cg07770968	NPBWR1	0.000187194	0.405660864
cg14629075	VGLL3	7.23E-05	0.40565045
cg00767010		0.000654035	0.405624153
cg15831060		0.000955793	0.405618207
cg06401579		9.24E-05	0.405569207
cg01521397	TAF4	0.000290372	0.405559068
cg09205920		8.60E-06	0.40555841
cg14674720		1.96E-05	0.405557531
cg23424407		3.34E-05	0.405554007
cg18158990	SYT10	8.60E-06	0.405548333
cg26855918		1.96E-05	0.405531277
cg11569159		1.50E-05	0.405482626
cg22685409	KCNA4	3.34E-05	0.405466795
cg08651937	PA2G4P4	7.23E-05	0.405457459

cg00336700		0.000117986	0.405433905
cg13186884		8.60E-06	0.405420276
cg10011091	SLC25A32	0.000117986	0.405410146
cg16291880		1.50E-05	0.405393631
cg08898253	UPK3A;UPK3A	0.000290372	0.405384814
cg27375198	PYY	4.34E-05	0.405375051
cg03293015	MATN2;MATN2;MATN2;MATN2	3.34E-05	0.405374604
cg07479670	NID1	0.00043997	0.405373897
cg25757820		3.34E-05	0.405365678
cg00810956		0.000187194	0.405332683
cg00089091	DPP10;DPP10	0.000187194	0.405321456
cg20433822	C10orf88	8.60E-06	0.405306394
cg01255414	FGF12;FGF12	8.60E-06	0.405270146
cg14301932		1.50E-05	0.405245435
cg13079571	TRIM39;TRIM39	1.96E-05	0.405230985
cg23062198	FAM84A	1.11E-05	0.405215143
cg14508508	GABRA5;GABRA5	8.60E-06	0.405208855
cg26817755		7.23E-05	0.405207224
cg02159402	GALNT11	9.24E-05	0.405180991
cg04362858		9.24E-05	0.405179587
cg15402529		7.23E-05	0.405159821
cg02093732		8.60E-06	0.405157661
cg21432954	TRPC4;TRPC4;TRPC4;TRPC4;TRPC4;TRPC4;T	8.60E-06	0.405144526
cg05076914	SMOC2;SMOC2	3.34E-05	0.405142974
cg15732768	DLX5	2.58E-05	0.405125635
cg13019491	SIX6	0.000233422	0.405123407
cg01786704	SNRPN;SNRPN;SNRPN;SNRPN	0.000137134	0.4050887
cg21810188	VCAN;VCAN;VCAN;VCAN	0.000290372	0.405081242
cg16397794	MKX	0.00043997	0.405057906
cg13960084	CACNA1B	4.34E-05	0.405046371
cg23990723	BIVM;BIVM;KDELC1;BIVM;BIVM	0.000187194	0.405035845
cg04027548	KCNJ8	8.60E-06	0.405009834
cg22337128	C14orf23;C14orf23	2.58E-05	0.404979391
cg22930445	SLC25A21;SLC25A21;LOC100129794	1.96E-05	0.404959073
cg08165796		0.000290372	0.404958596
cg02226645	MIR124-2	1.96E-05	0.404883962
cg04365452		8.60E-06	0.404873607
cg19772399	IGSF9B	5.59E-05	0.404867967
cg00593536	NXPH2	1.50E-05	0.404847265
cg10905495	EBF2	7.23E-05	0.404828939
cg03988540		5.59E-05	0.404798448
cg05712610	GALNT11	4.34E-05	0.404777528
cg00364016		1.11E-05	0.40476825
cg16123202		2.58E-05	0.404761913
cg02925039	ADAMTS20	8.60E-06	0.404760574
cg08231870		0.000955793	0.404757888
cg05962092	KCNA7	1.11E-05	0.404727667
cg02584498	GREB1	2.58E-05	0.40472673
cg05336115	RSPO4;RSPO4	3.34E-05	0.404726224
cg16624482	UMODL1;UMODL1	7.23E-05	0.404724576
cg09949949	RELN;RELN	1.50E-05	0.404704543
cg08185661	SYT9	2.58E-05	0.404701657
cg10157251		1.11E-05	0.404691894
cg20954870		4.34E-05	0.404661018
cg04917181	TSPYL5	0.000955793	0.404654686
cg26104143		9.24E-05	0.404648795
cg24822696	STL	0.006823935	0.404626769
cg15590989	MGC45800	1.50E-05	0.404617309
cg06829299		1.96E-05	0.404614756
cg08864105	DENND2D	0.000290372	0.404610432

cg09861917	FOXA2	9.24E-05	0.404538749
cg00495775	HOXD11	1.11E-05	0.404535556
cg04426311	LRRN1	0.000117986	0.404489118
cg02500300	STOX2;STOX2	3.34E-05	0.404479257
cg18557131	ZIC5	0.000117986	0.404447176
cg14179581		1.96E-05	0.404440566
cg17391928	SLC27A6;SLC27A6;SLC27A6;SLC27A6	4.34E-05	0.404412917
cg05763228	LRAT	1.11E-05	0.404411849
cg19480724	EVX1	0.001147425	0.404396831
cg23049291	LMX1A	1.96E-05	0.404382622
cg26150071	CSMD2	7.23E-05	0.404370463
cg00831222		3.34E-05	0.404344678
cg08843809	FBXO39	0.000187194	0.404336514
cg23122109	SLC19A3;SLC19A3	1.50E-05	0.404324679
cg18872497		8.60E-06	0.404293585
cg10590857	ADCY2	0.000233422	0.404293411
cg00321480		1.50E-05	0.404289343
cg14545970	CDC14B;CDC14B;CDC14B;CDC14B	0.000148457	0.404264107
cg04927931	SIM1	0.001948248	0.404221552
cg01879591		1.11E-05	0.404202582
cg05864140	RESP18	3.34E-05	0.404193757
cg07940072	FOXA1	7.23E-05	0.404165283
cg23359394	DOK5	0.000791389	0.404138342
cg07653946	GREB1L	8.60E-06	0.404119855
cg25023298		5.59E-05	0.404118218
cg00405190	WIPF1	9.24E-05	0.404108174
cg16052975		1.96E-05	0.40410128
cg08712932	EYA4;EYA4;EYA4	0.000187194	0.404096023
cg09577841	KLHL31	0.000537905	0.404095959
cg27037551	CCBE1	1.96E-05	0.404084594
cg08671647	PCDH7;PCDH7;PCDH7	1.11E-05	0.404081162
cg11727383	LBX1;FLJ41350	4.34E-05	0.404048486
cg13574488	EMX2;EMX2	1.50E-05	0.404046006
cg21725954	GRM1;GRM1;GRM1;GRM1	1.50E-05	0.404037513
cg10639888	RPRM	0.000117986	0.404037339
cg11480800	PYGO1	8.60E-06	0.404022709
cg19904058	LOC619207	1.11E-05	0.404019119
cg09871685		1.50E-05	0.403988619
cg03227021		3.08E-05	0.403988508
cg13770399	PTP4A1	3.92E-05	0.403988223
cg19611809	ONECUT1	1.18E-05	0.403984495
cg15194943	ADCYAP1;ADCYAP1	1.50E-05	0.403975098
cg03031234	EXOC6	9.24E-05	0.403970328
cg15060366	PCSK2	2.58E-05	0.403927055
cg16739441	KCNJ8	1.96E-05	0.403925103
cg05619587	SLC6A15;SLC6A15;SLC6A15	1.11E-05	0.403920509
cg17386057		2.58E-05	0.403920458
cg12974637	CCDC68;CCDC68;CCDC68	2.58E-05	0.403915785
cg13486410	C4orf31	2.58E-05	0.403905399
cg03976877	VIPR2;VIPR2	0.001639597	0.403904661
cg26006870		4.34E-05	0.403899472
cg08288811	PCDHA2;PCDHA1;PCDHA3;PCDHA3;PCDHA1	8.60E-06	0.403863675
cg12208258	CDH2;CDH2	0.000290372	0.403855147
cg02721665	GPR26	1.96E-05	0.403851968
cg04730685		1.50E-05	0.403831483
cg20113824		0.001948248	0.403814327
cg12592359	ASXL1;ASXL1	0.000148457	0.403809754
cg04342955	SHISA9;SHISA9	0.007873393	0.403773442
cg17812664		0.000148457	0.403759387
cg19677607	NEFM;NEFM	3.34E-05	0.403756776

cg23825335	CADPS;CADPS;CADPS	0.002304201	0.403738575
cg21699330	NFE2L3	0.001376172	0.403704812
cg20979852	SPSB4	0.000955793	0.403695118
cg20300343		5.59E-05	0.403665673
cg19786084	CACNG4	9.24E-05	0.403660506
cg06288251	TMEM108;TMEM108	0.000357885	0.403659289
cg16559598	TBX5;TBX5	0.000233422	0.403648452
cg17794169	FGFR2;FGFR2;FGFR2;FGFR2;FGFR2	0.000290372	0.403630511
cg24535910	NPY1R	8.60E-06	0.403621754
cg10588150	CSMD2	1.11E-05	0.403620691
cg23797411	SORCS3	5.59E-05	0.403584257
cg11782779	CDH6	9.04E-05	0.403583009
cg00711072	LOC404266;HOXB5;LOC404266;LOC404266;L(	0.000570759	0.403579847
cg25571189	LYPD1;LYPD1;LYPD1	9.24E-05	0.403579484
cg07600871	BRUNOL4;BRUNOL4;BRUNOL4;BRUNOL4	0.000117986	0.403568129
cg06680481	C20orf56	1.11E-05	0.403561412
cg18693673	ZSCAN18;ZSCAN18	7.23E-05	0.403558406
cg13382769	MAGI2	8.60E-06	0.403553357
cg07300408	RNASE12;RNASE11;RNASE12;RNASE11	0.000187194	0.403545024
cg24115264	TNFSF11;TNFSF11	0.000117986	0.403540307
cg15020568		1.50E-05	0.403536091
cg12690119	ZBTB38;ZBTB38	0.000233422	0.40352789
cg26429925	BNC1	1.96E-05	0.403526379
cg06368590	TRPS1	0.000262362	0.403519404
cg25198847		4.34E-05	0.403516836
cg15015109	HELZ	4.34E-05	0.403506469
cg14235846	NXN	0.000233422	0.4035051
cg23889010	SLPI	0.000148457	0.403489454
cg19673549		7.23E-05	0.403488948
cg09813525	PCDH8;PCDH8	7.23E-05	0.403453716
cg13733708	ZNF335	1.96E-05	0.403415614
cg16899991		0.000187194	0.403408863
cg04317962		0.000955793	0.403390813
cg24808280	C1orf114	0.000117986	0.40334749
cg04339360	KLF5	4.34E-05	0.403341414
cg19535896	ZSWIM2;ZSWIM2	0.000122184	0.403338808
cg21794225	PRKD1	1.11E-05	0.403334585
cg08682625	LOC727677	0.000187194	0.403329562
cg23441441	LIN7A	3.34E-05	0.403297313
cg02699336	ERC2	3.34E-05	0.403296216
cg07241925	MAEA;MAEA	1.50E-05	0.403289088
cg04047221	SOX1;SOX1	0.00043997	0.403287581
cg19787694	PRTN3	1.96E-05	0.403255234
cg10660909	CGREF1;CGREF1;CGREF1;CGREF1;CGREF1	0.000357885	0.403238081
cg14381623	LHX3;LHX3	8.60E-06	0.403231148
cg02491276	FGF14	8.60E-06	0.403226711
cg10762626	OCA2	9.24E-05	0.403208453
cg08297751	HAND2	1.96E-05	0.403191387
cg09997082	GIPR	2.58E-05	0.403102229
cg21977377	NKX2-2	0.000148457	0.403092942
cg18932726	DOCK1	0.000791389	0.403088914
cg19019214		0.000233422	0.40307265
cg18021368	PCDHB5;PCDHB5	1.50E-05	0.403069072
cg07222505	FAM169A	7.23E-05	0.403055093
cg00924255		1.11E-05	0.403053351
cg14551002	DOC2B	0.000122184	0.403041317
cg03343571	RNF39;RNF39	0.000654035	0.403019387
cg02662828	ZAR1	8.60E-06	0.403016568
cg22623649		5.59E-05	0.403004007
cg04861929	C11orf87	4.34E-05	0.402990194

cg03629943	CNNM1	3.34E-05	0.402980109
cg12638731	NBEA	8.60E-06	0.402977375
cg21711132	GABRG3	7.23E-05	0.402957509
cg21502255		0.001147425	0.402954049
cg16107724		1.50E-05	0.402894313
cg02741440	PLCXD3	1.11E-05	0.402877369
cg03847716		1.96E-05	0.402858321
cg03820688	MTIF2;MTIF2	3.92E-05	0.402839842
cg01493740	GJA1	1.50E-05	0.402835534
cg22562942	NEFM;NEFM	7.23E-05	0.402827039
cg00031165	RUNDC3B;ABCB1;RUNDC3B;RUNDC3B	0.000117986	0.402816937
cg02392634	ANK1	2.58E-05	0.402806631
cg00028935	ZIC4;ZIC4;ZIC4	1.96E-05	0.402789154
cg03169018	NEFM;NEFM	2.58E-05	0.402777062
cg26650064		1.50E-05	0.402769202
cg20584905	PAX3;PAX3;PAX3;CCDC140;PAX3;PAX3;PAX3	1.96E-05	0.40276449
cg20965743	ASPG	4.34E-05	0.402751192
cg26330116	LOC145845	7.23E-05	0.402735951
cg00954382	NPY2R	1.50E-05	0.402714073
cg08605326	CA10;CA10;CA10	2.58E-05	0.402711087
cg15970666		0.000117986	0.402707086
cg08658787		1.96E-05	0.402698267
cg16802439	GALNS	5.59E-05	0.402695789
cg26818625		0.000117986	0.402687717
cg11873147	C1QL2	3.34E-05	0.402665527
cg06085877	CCK	0.00043997	0.402655693
cg14519115	PLCXD3;PLCXD3	1.50E-05	0.402643843
cg22456692	WWC1;WWC1;WWC1	0.000117986	0.402635942
cg07143083	WBSCR17	3.34E-05	0.402611796
cg05474726	MIR124-2	1.96E-05	0.402609778
cg04974062	PRDM13	1.50E-05	0.40259009
cg08398132		1.11E-05	0.402583809
cg05344430	GSX1	0.000148457	0.402565474
cg04382396	ELANE;ELANE	2.58E-05	0.402543817
cg24059119		2.58E-05	0.402540445
cg20375342	FAM19A4;FAM19A4	1.96E-05	0.402494823
cg17100158	ZNF385B;ZNF385B;ZNF385B	0.000187194	0.402474974
cg20340302	BMP7	3.34E-05	0.402467595
cg13509702	SCRIB;SCRIB;MIR937	5.59E-05	0.402466047
cg24827660	PHACTR3	8.60E-06	0.402449756
cg26332253	GJA1	1.96E-05	0.402441151
cg15680720		5.59E-05	0.402436454
cg00652796	EPHA6	3.34E-05	0.402429333
cg25406138	POU4F2	8.60E-06	0.402408018
cg08867893	ZNF365;ZNF365;ZNF365;ZNF365;ZNF365;ZNF	0.000233422	0.402404833
cg22488859	CYP1B1	0.000955793	0.402388914
cg02841000		1.96E-05	0.402362638
cg25318270	PPP2R2C	0.000117986	0.402352834
cg27308329	FAM19A5	7.23E-05	0.40234008
cg12412390	UNC5C	5.59E-05	0.402332452
cg15736165	BNC1	8.60E-06	0.402316309
cg08343042	TMEM163	0.000357885	0.402310875
cg23217512		0.000537905	0.402305275
cg22072795		7.23E-05	0.402291055
cg01297721	HAPLN4;HAPLN4	4.34E-05	0.402276881
cg07897451		7.23E-05	0.402256848
cg16048517	CTNND2	9.24E-05	0.402239289
cg14276488	SLC1A1	0.000148457	0.402232129
cg26730416		0.00043997	0.402227355
cg26981309	MCHR2;MCHR2;MCHR2	1.11E-05	0.402221624

cg20072442	CTNNA2;LRRTM1;CTNNA2	7.23E-05	0.402213709
cg15927927	PPP2R2B;PPP2R2B;PPP2R2B;PPP2R2B;PPP2	3.34E-05	0.40220038
cg00811334	CTNNA2;CTNNA2	8.60E-06	0.402198315
cg12473912		0.000537905	0.402145471
cg09793172	KIF17;KIF17	4.34E-05	0.402137825
cg23207710	FGF12;FGF12	0.000290372	0.402121495
cg26267430		0.000955793	0.402108477
cg23491599	KCNJ3	5.59E-05	0.402090415
cg02215115	KCNK5	0.000357885	0.402088974
cg06352352	C16orf70	1.96E-05	0.402074592
cg07592963	KCNQ1DN	0.000290372	0.402043596
cg00204501	TJP1;TJP1;TJP1;TJP1	3.34E-05	0.402043174
cg12516359		1.96E-05	0.402029752
cg16781264	NMS	1.96E-05	0.401990408
cg02456451		2.58E-05	0.401989805
cg26224018	CACHD1	0.000290372	0.401965881
cg08640634	NR2E1	4.34E-05	0.401955251
cg19715410	OPRD1	0.000654035	0.401947582
cg13984515	CYR61	9.24E-05	0.401933348
cg08473752	NLK	1.50E-05	0.401925615
cg23828301	IL5RA;IL5RA	9.24E-05	0.401905272
cg19270858	KRT18;KRT18	9.24E-05	0.401888325
cg15975574	RUNX1T1	8.60E-06	0.401886157
cg03340036	PIGY;PIGY	7.23E-05	0.401876945
cg22945387	SFTA3	8.60E-06	0.401873894
cg18430156	FAM171B;FAM171B	9.24E-05	0.40186266
cg23310835		0.000117986	0.401855367
cg25147376	ILDR2	1.11E-05	0.401851956
cg00097146	SIX3	3.34E-05	0.401843305
cg08031368	VSNL1	4.34E-05	0.401807374
cg08933227	ELOVL4	7.23E-05	0.401805223
cg04832177		1.11E-05	0.401800215
cg07093324	ACTR3	0.000233422	0.401797098
cg24593372	C1orf83	4.34E-05	0.401791964
cg16391955		5.59E-05	0.401789318
cg07015629	ERBB4;ERBB4	1.50E-05	0.401764597
cg17837191	TEX2	9.24E-05	0.40175245
cg23117778	RAD21L1	0.000122184	0.401742274
cg22507154		7.23E-05	0.401723228
cg05208605		1.50E-05	0.401704746
cg17314538	MTNR1A	1.96E-05	0.401699103
cg15379412	NR2F2;NR2F2;NR2F2;NR2F2;MIR1469;NR2F2	1.11E-05	0.40166606
cg03096126	ZNF532	5.59E-05	0.401656935
cg27391816	SLC35E2	0.000148457	0.401633601
cg12664464	GATA5;GATA5	0.000791389	0.401630137
cg06938264	PCDHA6;PCDHA2;PCDHA1;PCDHA7;PCDHA1	2.58E-05	0.401606843
cg06708634	SIM1	7.23E-05	0.401602386
cg25623768		0.000148457	0.401595262
cg26709988	CRISPLD2	0.001639597	0.401591891
cg13378394	KCNC3	1.50E-05	0.401590209
cg02481000	PRKCZ;PRKCZ;PRKCZ	2.58E-05	0.401579438
cg00688962	KCNIP4	1.11E-05	0.401575393
cg26364205	EPHA6	1.11E-05	0.401568836
cg00675229		0.000955793	0.401556256
cg26509328		1.96E-05	0.401526239
cg09225287	SEMA3C	1.11E-05	0.401517989
cg02935154	VWDE;VWDE	3.34E-05	0.401487521
cg09408098		9.24E-05	0.40147898
cg24880665	KDM5B	9.24E-05	0.401472942
cg20986996	TCF21;TCF21	8.60E-06	0.401459539

cg19724555	GALNT9	2.58E-05	0.401458621
cg18732032	TFAP2A	1.50E-05	0.401443142
cg02309841	VGLL4	0.000148457	0.401423571
cg19165390		2.58E-05	0.401417942
cg12271981	SLITRK5	0.000117986	0.401415957
cg06474225	HTRA1	0.000791389	0.401413198
cg18343474		8.60E-06	0.401401317
cg00147462		5.59E-05	0.401399048
cg02055132	EPHA6	0.000148457	0.401383634
cg10338518	AFF1	0.000290372	0.401373342
cg13011388	EFS;EFS	0.000117986	0.40135807
cg00088130	SOX14	1.50E-05	0.401350075
cg23056157	NALCN	2.58E-05	0.401340268
cg22914729	PITX2;PITX2;PITX2	5.59E-05	0.401338462
cg10375890		9.24E-05	0.401329926
cg05211057	COL9A1;COL9A1	8.60E-06	0.401321644
cg17147211		0.00043997	0.401316812
cg03509106	C4orf31	2.58E-05	0.401311715
cg26839512	SPATA18	0.003734675	0.401288484
cg17152981	GPR6	2.58E-05	0.401287407
cg01057656	CBLC;CBLC;CBLC;CBLC	0.003189589	0.401255186
cg21302951	KCNH7;KCNH7	3.34E-05	0.401252169
cg09891125		0.000537905	0.401229684
cg13457172	VAX1;VAX1	2.58E-05	0.401221121
cg14468658	PCDHGA2;PCDHGA3;PCDHGA1;PCDHGA3	1.96E-05	0.401217053
cg23413924	DUOX1;DUOX1	3.34E-05	0.401199597
cg23847381	EPB41L3	0.000654035	0.40116608
cg12507125	WWTR1;WWTR1;WWTR1	4.34E-05	0.401147402
cg07961994	GRID2	1.11E-05	0.401143751
cg14069192	CDC14B;CDC14B	1.50E-05	0.401138438
cg00345443		0.000148457	0.401136183
cg02898094	DLX6AS;DLX6	9.24E-05	0.401102449
cg08633074	TXNDC11	4.34E-05	0.401093261
cg14636534	LOC645323;LOC645323;LOC645323	3.34E-05	0.401076628
cg14851108	GRID1	0.000233422	0.401061161
cg25588844	TAF1B	9.24E-05	0.40104909
cg15696906	TRPC4;TRPC4;TRPC4;TRPC4;TRPC4;TRPC4	8.60E-06	0.401005419
cg14362630		7.23E-05	0.400997892
cg07380506	CYP1B1	0.000357885	0.400975697
cg10702770		8.60E-06	0.400953029
cg14612428	GNAL;GNAL;GNAL;GNAL;GNAL	8.60E-06	0.400931455
cg05446424	FAM84A	9.24E-05	0.400886331
cg19819285	ZSCAN1	1.11E-05	0.400853788
cg02918054	SSTR1	9.24E-05	0.400841579
cg26283893	HOXC13	1.96E-05	0.400826581
cg15365032		8.60E-06	0.400794707
cg15445554	SLC6A1	1.50E-05	0.400792716
cg25143824	PAK7;PAK7	2.58E-05	0.400782606
cg19091036		1.50E-05	0.400780691
cg20449670		0.000187194	0.400759474
cg18376288		8.60E-06	0.400747464
cg04859466	SOX2OT	1.96E-05	0.400745634
cg04598121	PENK;PENK	8.60E-06	0.400739816
cg14972743	ZSCAN18;ZSCAN18;ZSCAN18;ZSCAN18	0.000233422	0.400724776
cg07104706	SLITRK1;SLITRK1	5.59E-05	0.400721273
cg24923694		3.34E-05	0.400704878
cg09851951		0.000117986	0.400695826
cg26296364	TMEM26	1.50E-05	0.400692892
cg23202722	PHC2;PHC2	7.23E-05	0.400679875
cg08700032		3.34E-05	0.400664573

cg02647941	ZIC1	3.34E-05	0.400664295
cg10297191	CYP39A1;SLC25A27	1.96E-05	0.400649314
cg02162886	SIX1	0.000262362	0.400645969
cg24705960	FEZF2	9.24E-05	0.400626689
cg25355316		1.50E-05	0.40061823
cg21547982	MAGI1;MAGI1;MAGI1	5.59E-05	0.400592577
cg18443253	SSTR1	0.000654035	0.400591373
cg03307177		0.000791389	0.400588737
cg12787323		0.00043997	0.400566879
cg20635409		8.60E-06	0.400536028
cg00590036	TMEM181	0.000290372	0.400528764
cg04943986	NR2F2;NR2F2	8.60E-06	0.400527221
cg23626122	SLITRK3	0.000148457	0.400504583
cg00074145	ONECUT1	0.000117986	0.400499037
cg12069267		1.50E-05	0.400485257
cg22680204	CRMP1	1.11E-05	0.400477049
cg02824202		0.000187194	0.400471592
cg26517171	TLX3	8.60E-06	0.400469076
cg27117982	MGST1;MGST1;MGST1;MGST1	1.96E-05	0.400465662
cg11327408	TRIM39;TRIM39	2.58E-05	0.400458901
cg13794993	SALL3	8.60E-06	0.400439905
cg14676529	PCDHA6;PCDHA2;PCDHA1;PCDHA9;PCDHA7	7.23E-05	0.400390951
cg13560871	C1orf115	0.001948248	0.400386016
cg08437576	PAQR5;PAQR5	0.000537905	0.400381282
cg07489502	PCDHGA4;PCDHGA11;PCDHGA11;PCDHGA9;	0.009066563	0.400366759
cg06486190	MDFIC;MDFIC	0.000537905	0.400357605
cg14031054	PBX1	3.34E-05	0.400351707
cg17866667	CACNG8	1.11E-05	0.400351326
cg23632985		1.50E-05	0.400332889
cg22520644	WDR17;WDR17	0.000357885	0.400331607
cg13043862	EYA1	5.59E-05	0.40029462
cg07973435	SIM1	4.34E-05	0.400283913
cg18394533	MOCS1;MOCS1	1.96E-05	0.400259646
cg06416733		2.39E-05	0.400251272
cg19414741	PENK;PENK	1.11E-05	0.400250697
cg01926051	ESRRG	9.24E-05	0.400244316
cg11832210	SLC16A12	0.00041966	0.40023925
cg20890210	KCNB2	8.60E-06	0.400238465
cg07009376		0.000290372	0.4002229
cg06827562		1.96E-05	0.400218725
cg07393734		5.59E-05	0.400187004
cg07408835	PCDHAC1;PCDHA7;PCDHAC1;PCDHA12;PCD	1.50E-05	0.400182573
cg21669441	AP3B2	1.50E-05	0.400174736
cg09054949	CXCL1	1.50E-05	0.40017198
cg02879662	HIF3A;HIF3A	0.000148457	0.400157059
cg22575603		1.96E-05	0.400116849
cg01296889		0.000148457	0.400101116
cg10182697	NKX6-2	0.000148457	0.400098555
cg14984684	RASGRF1;RASGRF1	1.11E-05	0.400095395
cg23335460	CRTAC1	4.34E-05	0.400052166
cg26925231	SGCZ	0.000117986	0.400037705
cg04778012	STAG3;GPC2	7.23E-05	0.400032249
cg14714797	AHRR	0.00043997	0.400023655
cg00959431		1.11E-05	0.400019272
cg02484469	GATA5	0.000537905	0.400014095
cg08637438		8.60E-06	0.400004605
cg04261192	NELL1;NELL1	0.000357885	0.399930616
cg25198049	CGN	3.34E-05	0.399919614
cg21914290	PRRX1;PRRX1	7.23E-05	0.399909498
cg17035091	CAMK2B;CAMK2B;CAMK2B;CAMK2B;CAMK2B	0.000537905	0.399892162

cg14557699	PCDHA12;PCDHA7;PCDHA6;PCDHA10;PCDH/	0.000187194	0.399866672
cg09076077	C20orf197	0.000117986	0.399862855
cg04801617	RFX4	7.94E-05	0.399830304
cg02052762	ANKFY1	4.34E-05	0.399785571
cg10551134		0.00043997	0.399772737
cg00721170	CXCL5	2.58E-05	0.399748519
cg26836233	SLC6A11	8.60E-06	0.399737189
cg16935065	ST6GAL2;ST6GAL2;ST6GAL2	1.96E-05	0.399736236
cg25248415		0.000117986	0.399693386
cg04172348	SYN2;SYN2;SYN2;SYN2	4.34E-05	0.399688155
cg24899571	JAM3	0.000117986	0.399679553
cg10572355		4.34E-05	0.399647252
cg00574907	SEPT10;SEPT10;ANKRD57	9.24E-05	0.399619432
cg11926610		8.60E-06	0.399604424
cg06788631		9.24E-05	0.399603284
cg24284973	NTM;NTM;NTM;NTM	0.000187194	0.399577201
cg21200408	NKX2-4	9.24E-05	0.399571234
cg06724588	SALL1;SALL1	0.000357885	0.399564346
cg04205769	DPH5;DPH5;DPH5	0.000654035	0.399555533
cg05350411		8.60E-06	0.399547746
cg03555227	RANBP17	8.60E-06	0.399544162
cg16099804	EDIL3;EDIL3	9.24E-05	0.399540835
cg09375033	DZIP1L;DZIP1L	0.000187194	0.399513988
cg27265170	DMRTA2	1.96E-05	0.399490051
cg24190603	SNAP91;SNAP91	0.000654035	0.39944392
cg22926931	MYOD1	1.11E-05	0.399423233
cg00754604	RBMS1;RBMS1	2.58E-05	0.399404129
cg19592472	OXT;OXT	5.59E-05	0.399391725
cg01357292	ZNF662;ZNF662;ZNF662	2.58E-05	0.399390901
cg12515917	TG	5.59E-05	0.399382574
cg19915738	ZNF229	0.000117986	0.399379055
cg14345154	TTC8;TTC8;TTC8	0.000955793	0.399372664
cg00631482	RAP1GAP;RAP1GAP;RAP1GAP	0.000290372	0.399359162
cg03365311	MIR129-2	8.60E-06	0.399348637
cg03287339	WDR90	8.60E-06	0.399340142
cg13679303		2.58E-05	0.39931301
cg18358869		1.50E-05	0.399304177
cg05048199	FBXL2;FBXL2	0.000187194	0.399297032
cg02124887		0.006823935	0.399291855
cg01866955		2.58E-05	0.399291477
cg03598338	BCAR1;BCAR1;BCAR1;BCAR1;BCAR1	3.34E-05	0.399274573
cg22595235	LRRN1	7.23E-05	0.399264116
cg01822130	EPHA6	0.000117986	0.399255729
cg06634716	ESRRG	2.58E-05	0.399241723
cg13982454	PRTG	0.000117986	0.399240906
cg00590324	TCF21;TCF21	0.000290372	0.399240621
cg14074052		1.18E-05	0.39922702
cg27480727	PAX3;PAX3;PAX3;PAX3;PAX3;CCDC140;PAX3	1.50E-05	0.399216845
cg18713028		2.58E-05	0.399201817
cg27592331	GATA6	0.000148457	0.399193748
cg16218721	HOXD4	1.11E-05	0.399189064
cg00780971		0.000117986	0.399185789
cg03983476	NOL10	5.59E-05	0.399131073
cg16126816	EGFLAM;EGFLAM	0.000117986	0.399120834
cg10151685		2.58E-05	0.399112648
cg13867865	C12orf56;C12orf56	0.000526175	0.399112145
cg10487428	PDE4D	1.11E-05	0.39908383
cg24505892		0.000955793	0.39908144
cg02161900	MKX	1.11E-05	0.399068711
cg05826255	ST8SIA3	7.23E-05	0.399049867

cg13724788	NCAN	5.59E-05	0.399000892
cg16956686	SDK1;SDK1	9.24E-05	0.398963544
cg07270078	ISL1	3.34E-05	0.398961885
cg15169266		4.34E-05	0.39895653
cg15424250	SCRN1;SCRN1;SCRN1;SCRN1	0.001639597	0.398952004
cg09183753	TFAP2C	2.58E-05	0.398946295
cg11527913	GALNT2	2.58E-05	0.398933962
cg01461410	TBX18	4.34E-05	0.398919672
cg23854488	FGF4	0.000148457	0.398910515
cg00812833	PLD5	0.000955793	0.398902058
cg00955967		0.000117986	0.398896074
cg04488111	PMVK	0.000187194	0.398869194
cg20374054	ROBO3	0.000117986	0.398868688
cg18758906		1.96E-05	0.398868269
cg05022673	DST;BEND6	0.00045851	0.398831164
cg27058257	VSTM2B	0.001376172	0.398831126
cg27593384	CDH2	0.000148457	0.398823898
cg14985989	MADCAM1;MADCAM1	0.000357885	0.398823528
cg03489969	MATN2;MATN2	3.34E-05	0.398821008
cg27425452	PARK2;PACRG;PARK2;PACRG;PACRG;PARK	4.34E-05	0.398809887
cg22821324	GPM6A;GPM6A	0.000357885	0.398807047
cg03193328	TPST1	0.000654035	0.398800139
cg03882167	FAM123C;FAM123C;FAM123C;FAM123C	2.58E-05	0.398799666
cg01924596	PDZD2	0.000148457	0.398774596
cg26703456	KIAA1486	7.23E-05	0.398754227
cg06161738	GALNT11	0.00043997	0.398748317
cg02043159	PCDH10;PCDH10	0.000290372	0.398738879
cg04609694		2.58E-05	0.398733984
cg05344949		5.59E-05	0.398727172
cg02728595		0.001393182	0.398709811
cg22535307	FBN1	0.000187194	0.398674355
cg03819783		1.96E-05	0.398668993
cg24550026	BCL2L15;BCL2L15	0.000357885	0.398651191
cg14592474	MAGI2	0.00043997	0.398640514
cg25192419	DOCK5;DOCK5	0.000537905	0.398624619
cg01097384		0.000148457	0.398610521
cg24279418		1.96E-05	0.398603575
cg19228118	CSMD2;C1orf94	4.34E-05	0.398602753
cg01281911	FOXE3	7.23E-05	0.398573246
cg21184585	SEPT10;ANKRD57;SEPT10	3.34E-05	0.398550838
cg01490094		1.96E-05	0.398489089
cg05158197	SPOCK3;SPOCK3	0.000187194	0.398482439
cg04938966		8.60E-06	0.39847945
cg08790676		0.001948248	0.398476043
cg09342766	RIMS1	3.34E-05	0.398465134
cg02192300		0.000955793	0.398457543
cg00779065		0.000148457	0.398447629
cg02256049		0.000537905	0.398425739
cg16854917	NCRNA00085	0.000654035	0.398420667
cg14480507		1.50E-05	0.39840609
cg06997767	TRPS1	9.24E-05	0.398399855
cg08857994		1.50E-05	0.398399018
cg13980454	NKX2-1;NKX2-1	0.000233422	0.398395482
cg05008854	KIN	1.11E-05	0.398395342
cg09624466	OTX2OS1	0.00043997	0.398370613
cg06312785	PPP2R3A	0.000117986	0.398349506
cg07212327	FAM49B	0.000117986	0.398344791
cg21249659	OLR1	1.96E-05	0.398331023
cg11564711		1.96E-05	0.398329824
cg18473521	HOXC4;HOXC4	0.000117986	0.398312894

cg02954212	LOC283856;GNAO1;GNAO1	3.34E-05	0.398302679
cg06056415		2.58E-05	0.398286504
cg24746100	FOXI3	7.23E-05	0.398245131
cg17309441		7.23E-05	0.398243326
cg24934063	FEZF1;FEZF1	5.59E-05	0.398236391
cg06820905		1.50E-05	0.398236067
cg20504856		4.34E-05	0.398188427
cg12992827		9.24E-05	0.398187284
cg11241498		1.96E-05	0.398132247
cg13257636	CLIP4	0.000233422	0.398125049
cg16659043	SAMD12;SAMD12	3.34E-05	0.398113198
cg24777950	CTSG	4.34E-05	0.398094196
cg21122424		0.000148457	0.398077804
cg08714996	PCDHGB1;PCDHGA2;PCDHGA1;PCDHGB1;PC	0.001948248	0.398059665
cg10544031	PAX3;PAX3;PAX3;PAX3;PAX3;CCDC140;PAX3	8.60E-06	0.3980321
cg09363443		8.60E-06	0.398026654
cg22632840	SSH3	0.000357885	0.39802363
cg19047707	ECEL1	4.34E-05	0.398014749
cg02353916	LOC285550	4.34E-05	0.398000903
cg05580181		5.59E-05	0.397964359
cg12440258		5.59E-05	0.397953891
cg00584450	FOXL1;FOXL1	0.000290372	0.39794804
cg16002305		0.000148457	0.397947802
cg17029168	NKX2-2;NKX2-2	8.60E-06	0.397930258
cg15202102	PRKCDBP	9.24E-05	0.397929274
cg00059024	WDR93;PEX11A	5.59E-05	0.397903985
cg03322353	MAML3	0.001376172	0.397868308
cg08822075	NFE2L3	7.23E-05	0.397858847
cg02296171		3.34E-05	0.397845779
cg20820063	C18orf34;C18orf34;C18orf34	0.000148457	0.397837239
cg22682154		1.50E-05	0.39783643
cg11905007	CADPS;CADPS;CADPS	0.000233422	0.397800965
cg09841488	GLRA3;GLRA3	3.34E-05	0.397780008
cg06034527		0.000117986	0.397719211
cg06266613		0.000148457	0.397708661
cg15927720	SLIT3	0.00043997	0.397706744
cg18413131	NUDT16P;NUDT16P	0.000654035	0.397697091
cg14330189		2.58E-05	0.39769574
cg11828070	GOLSYN;GOLSYN;GOLSYN;GOLSYN;GOLSYN	0.000654035	0.397668292
cg16384774	FOXN3;FOXN3	2.58E-05	0.397666333
cg11276093	MYOF;MYOF	2.58E-05	0.397660989
cg27044455		4.34E-05	0.397660647
cg22762615	FGFR2;FGFR2;FGFR2;FGFR2;FGFR2	2.58E-05	0.39765634
cg18314424		0.000233422	0.397649485
cg14904662	ANK1;ANK1;ANK1;ANK1;ANK1	9.24E-05	0.397643787
cg17954152	PROX1	4.34E-05	0.397631102
cg25541209	ZMYND8;ZMYND8;LOC100131496;ZMYND8	7.23E-05	0.397624446
cg24151352	FOXF2	0.000148457	0.397621826
cg05942128	HOXD11	1.50E-05	0.397620107
cg21964551	LAD1	4.34E-05	0.397614722
cg11548303		4.34E-05	0.397610438
cg16689481	TCHH	0.000117986	0.397604379
cg07246273		2.58E-05	0.397594718
cg12857945	PAX9	1.11E-05	0.397592496
cg10770187	LYPD6	2.58E-05	0.397581174
cg00829990		9.24E-05	0.397569361
cg17177455		1.96E-05	0.397552662
cg02711929	HTRA1	7.23E-05	0.397531236
cg19083779	PRKAA2	0.000233422	0.397523798
cg16075649	LOC283663	2.58E-05	0.397505341

cg04564030	CDH7;CDH7	8.60E-06	0.397499505
cg07633317	ZNF423	0.002304201	0.397483613
cg06984156	CDA	1.50E-05	0.397481933
cg16823083	WDR8	4.34E-05	0.397473929
cg10880485		8.60E-06	0.397465254
cg16798252	FLJ32063	5.11E-05	0.39746091
cg05927432	GOLSYN;GOLSYN	1.50E-05	0.397446348
cg08065231	ZNF177	0.000117986	0.397442856
cg17151990	FAM110B;FAM110B	0.004357816	0.397440071
cg09499856	PLCXD3	1.50E-05	0.397431422
cg07529754	NRXN1;NRXN1;NRXN1	0.000290372	0.397426642
cg12883523	SOX17	2.58E-05	0.397419914
cg04241652	FLJ42875;PRDM16;PRDM16;FLJ42875	0.000117986	0.397418819
cg11660146	POU2F3	0.000290372	0.397402819
cg26633107	FAM84A	3.34E-05	0.397387615
cg11732642	SOX9	9.24E-05	0.397382234
cg22007666	ESRRG;ESRRG;ESRRG;ESRRG;ESRRG	2.58E-05	0.397371633
cg06761921	BRUNOL4;BRUNOL4;BRUNOL4;BRUNOL4	4.34E-05	0.397359058
cg02471153	KCNS2	4.34E-05	0.397344156
cg07198881	FAM78B	2.58E-05	0.397341588
cg09789874		1.11E-05	0.397321748
cg18699744	PNLIPRP2	8.60E-06	0.397319491
cg27022956	SOX2OT	9.24E-05	0.397309393
cg06927812		0.000122184	0.397297918
cg17685628	NKX2-8;NKX2-8	1.50E-05	0.397291296
cg15649236	MIR10A	0.000187194	0.39728223
cg14458834	HOXB4	0.000290372	0.397278373
cg07641160	FAM162B;FAM162B	0.000290372	0.397273169
cg07721203	ALDH1A2;ALDH1A2	0.000117986	0.397268285
cg07220782	LOC100270746;C6orf41	0.000148457	0.397255717
cg26577454	CNTNAP4;CNTNAP4	7.23E-05	0.397233276
cg16370875	ELOVL4	3.34E-05	0.397215856
cg14153637	RALYL;RALYL;RALYL;RALYL	4.34E-05	0.397194298
cg05053562	NOTO	9.24E-05	0.397172044
cg06392753	IL1R1	0.00043997	0.397126094
cg04656042		5.59E-05	0.397119433
cg00149716		3.34E-05	0.397095239
cg00316839		7.23E-05	0.397082909
cg04166294		5.11E-05	0.397080947
cg17918002	MKX	2.58E-05	0.397077469
cg14039246	ECHDC2	7.23E-05	0.39706156
cg10933003	HAND2	1.50E-05	0.397045317
cg17412005	MUTYH;MUTYH;MUTYH;TOE1;MUTYH;MUTYH	4.34E-05	0.397029203
cg10864319	FIGN	5.59E-05	0.397019898
cg20431191	DDAH1;DDAH1	0.000117986	0.397018892
cg02154186	PNMA2;PNMA2	1.11E-05	0.397002185
cg26360616		3.34E-05	0.396985289
cg13237068	CENPJ	0.000233422	0.396961552
cg18364120	AMICA1	5.59E-05	0.396960057
cg05658487		3.34E-05	0.396955321
cg04469059	NEUROG1	4.34E-05	0.396948767
cg11222127	LHX5	8.60E-06	0.396942721
cg25532501	GLB1L2	4.34E-05	0.396941589
cg15245709		9.24E-05	0.396906821
cg18432099	TACR1;TACR1	0.000122184	0.396905313
cg19656282	TLX2	0.001147425	0.396903629
cg24436906	BOK	3.34E-05	0.396899364
cg09543255	SLC7A14	5.59E-05	0.396889405
cg00875989		4.34E-05	0.396882299
cg16078649	RNF39;RNF39	7.23E-05	0.396879754

cg22805603	NLRC4;NLRC4	1.50E-05	0.396867586
cg00157572	SIX6	8.60E-06	0.396862928
cg27349081	RIC3;RIC3	2.58E-05	0.396852923
cg05311388	SCIN	0.000233422	0.396779598
cg13140167	SYN3	8.60E-06	0.396763232
cg05363616	TBX15	8.60E-06	0.396747598
cg21190228	HDAC4	9.04E-05	0.396701341
cg01161204		7.23E-05	0.396693587
cg14290576	NFIL3	0.000654035	0.396684013
cg21412973		1.50E-05	0.396678949
cg08977536		8.60E-06	0.396640304
cg09965258	NFIB;NFIB	7.23E-05	0.396629628
cg14579819	IGSF9B	1.96E-05	0.396601675
cg01404163	TOX3;TOX3	1.11E-05	0.396589306
cg10171357		2.58E-05	0.396576236
cg06149159		0.0050758	0.39655046
cg19736128	EFS;EFS;EFS;EFS	0.00043997	0.396546257
cg22025849		4.34E-05	0.396524402
cg18623980		0.000233422	0.396515173
cg09660365	SLC6A11	3.34E-05	0.396499281
cg10660256	BHMT;BHMT	1.50E-05	0.396498159
cg02993352	PNMA2	0.000233422	0.396479583
cg26614154	C1orf92	2.58E-05	0.396460614
cg00622837		9.24E-05	0.396445898
cg10244340		0.000955793	0.396427839
cg08019736		1.96E-05	0.396418967
cg18361098	PAX9	1.50E-05	0.396417521
cg08928408		1.11E-05	0.39639241
cg11199046		1.11E-05	0.396381589
cg11206067	PHYHIPL;PHYHIPL	0.000233422	0.39636333
cg00390775	PRUNE2	8.60E-06	0.396362019
cg27376182		0.000357885	0.396356637
cg04664153	GPR39	0.000187194	0.396346518
cg23506143		0.000705485	0.396341475
cg03992323	TP53BP2;TP53BP2	3.34E-05	0.396333279
cg07962338		0.000357885	0.396331903
cg18926797	DCAF5	3.34E-05	0.396300208
cg16452651	ITSN1;ITSN1	0.000148457	0.396293417
cg23532300	LOC100270746;C6orf41	4.34E-05	0.396273215
cg15612945	VPS54;VPS54	2.58E-05	0.396267782
cg07294406		9.24E-05	0.396264445
cg19522511		0.00043997	0.396261244
cg22946150	SH3GL3;SH3GL3;SH3GL3	3.34E-05	0.396247785
cg14213590	GPSM1	2.58E-05	0.396217684
cg20107395	DOK5;DOK5	0.000290372	0.396214369
cg05694245	MKX	1.50E-05	0.396211883
cg07099161	RAX	1.11E-05	0.396210125
cg04182865	RNF14	4.34E-05	0.396203948
cg17605476	GRIA2;GRIA2;GRIA2	1.50E-05	0.396183633
cg14439622	GATA6	3.34E-05	0.396177944
cg08217124	NKX2-5;NKX2-5;NKX2-5	5.59E-05	0.396177629
cg26469608		2.58E-05	0.39617594
cg05919360	DAB1	4.34E-05	0.396173282
cg03699843	SNX20;SNX20	0.000791389	0.396165283
cg21174746	CTNND2	7.23E-05	0.396164606
cg22970357	EN1	1.50E-05	0.396164014
cg08658621	DPYSL4	4.34E-05	0.396133677
cg18121003	CCDC81;CCDC81	0.000290372	0.39611616
cg23338668		8.60E-06	0.396105642
cg00688963	THRB;THRB;THRB;THRB;THRB;THRB	0.002304201	0.396096343

cg11941920		3.34E-05	0.396087102
cg09125430	KCNJ8	4.34E-05	0.396081629
cg07131533	KCNN2	8.60E-06	0.396081296
cg23003783	GRM6;GRM6	8.60E-06	0.396080854
cg10804656		5.59E-05	0.39601787
cg26130488		0.000117986	0.396006836
cg11296759	CD163L1	8.60E-06	0.396003545
cg12265623	NXPH1	4.34E-05	0.395974807
cg19649900	SBNO2	1.96E-05	0.395971535
cg10366797	HOXC4	8.60E-06	0.395955399
cg20934416		0.000187194	0.395913901
cg21200703	SLC34A2	1.50E-05	0.395913677
cg27169020	BNC1	2.58E-05	0.395913482
cg15610437	AZU1	1.50E-05	0.395911056
cg01589129		0.000187194	0.395883374
cg16927040	SST;SST	5.59E-05	0.395878809
cg26033349	ZSWIM2	2.58E-05	0.395875818
cg13359415	LGI2	0.000290372	0.395849386
cg24500630		0.000117986	0.395847827
cg05641882	CXCL3	2.58E-05	0.395841839
cg14946389	GPC6	3.34E-05	0.395841169
cg21816308		4.34E-05	0.395829801
cg13649056		8.60E-06	0.395822712
cg17370272	UQCRFS1	4.34E-05	0.395786846
cg19311153	MARVELD3;MARVELD3	2.58E-05	0.395759707
cg02842496	ADCY8	1.50E-05	0.395757841
cg01141812	CLVS2	2.58E-05	0.395734667
cg06275859		1.50E-05	0.395718902
cg07749597		0.000117986	0.395716153
cg16387467	CNDP2;CNDP2	0.001639597	0.395706118
cg18329187	CKB	0.002714607	0.395677601
cg20158867	DLC1;DLC1	0.000187194	0.395674669
cg02249292	MKX	1.11E-05	0.395666254
cg14679587	GLS2	9.24E-05	0.395663524
cg02996869	LPHN2	4.34E-05	0.395655607
cg15301316		5.59E-05	0.395649756
cg12748332	MPPED2	0.000955793	0.395635253
cg08073882	JAKMIP1;JAKMIP1	0.005897668	0.395603766
cg09128567	ECHDC2	0.000187194	0.395581645
cg08570521	TLL1;TLL1	5.59E-05	0.39557567
cg07142201	C11orf87	7.23E-05	0.395572095
cg10758306	OVOL2	3.34E-05	0.395563309
cg24024056		0.000290372	0.395557903
cg04303509	ZNF792	0.000791389	0.395504396
cg00425918	NKX2-6	1.11E-05	0.395498812
cg11308643	GRIA4;GRIA4;GRIA4;GRIA4	1.96E-05	0.395487593
cg16319691	USH1C;USH1C	7.23E-05	0.395479373
cg23818621	CSMD1	3.34E-05	0.395476331
cg11846956	KLK10;KLK10;KLK10	2.58E-05	0.395476029
cg17598466		0.000187194	0.395467479
cg12064372		9.24E-05	0.395467253
cg21255942		0.000290372	0.395458456
cg10600178	RUNDC3B;RUNDC3B;RUNDC3B;ABCB1;RUND	7.23E-05	0.395447841
cg00791253	KSR2	0.000233422	0.395437229
cg05162166		9.24E-05	0.395429437
cg01573562	RPH3A;RPH3A	1.96E-05	0.395407092
cg27545615	PFKFB3;PFKFB3	0.000955793	0.395407054
cg04107140	ADRA2C	0.000791389	0.395402581
cg17807528	EIF5A2	0.000148457	0.395384618
cg22807449	HOXB2	0.000117986	0.39536455

cg26389950	PCDHB16	3.34E-05	0.395351851
cg16633901		0.001147425	0.395347312
cg14410016	HOXD9	1.11E-05	0.395347045
cg16056105	INSM2	0.000148457	0.395298494
cg03266453	EN1	4.34E-05	0.39529437
cg21515785	FAM174A	2.58E-05	0.395291681
cg15232894	NOVA2	8.60E-06	0.3952519
cg02162880	TFAP2A	1.50E-05	0.395238669
cg21678461	DBX1	9.24E-05	0.395236394
cg16378063		0.000357885	0.395231792
cg06653898		5.59E-05	0.395222723
cg14776321	NCRNA00202	1.11E-05	0.395216141
cg12348202	PTPRN2;PTPRN2;PTPRN2	1.50E-05	0.395191037
cg24430580	PITX2;PITX2;PITX2;PITX2	3.34E-05	0.395185162
cg20303399	ADRA1A;ADRA1A;ADRA1A;ADRA1A	8.60E-06	0.395171601
cg14414464		1.11E-05	0.395165632
cg13227806		7.23E-05	0.395131932
cg25928819		1.11E-05	0.395124535
cg02235249	LOC440925	1.50E-05	0.395098375
cg21326139	MAEA;MAEA	4.34E-05	0.395098164
cg21426759	TRIM39;TRIM39	1.50E-05	0.395083255
cg14009098	SLC35F3	1.50E-05	0.395055165
cg17942121	SIM1	8.60E-06	0.395040673
cg08949408	C1QL3	1.96E-05	0.395036807
cg15516558	DEDD;DEDD;DEDD	2.58E-05	0.395006831
cg08472633	GALNS	1.11E-05	0.394997916
cg21762728	C6orf70	4.34E-05	0.394993252
cg02207200	SLC7A2	5.59E-05	0.394979429
cg11869770	WTIP	5.59E-05	0.394951987
cg00024086		4.34E-05	0.394899879
cg21009965	LHX8	8.60E-06	0.394879807
cg05299793	KCNH5;KCNH5;KCNH5	8.60E-06	0.394874989
cg06659338	C1orf83	1.50E-05	0.394857309
cg15966610	IL7	4.34E-05	0.394856429
cg16329650	ERBB4;ERBB4	0.000262362	0.394839534
cg03226000	SLC6A2	3.34E-05	0.394816695
cg23582644	SERPINB2;SERPINB2	1.11E-05	0.394810869
cg20117153		2.58E-05	0.394801065
cg02221302	C3orf14	1.96E-05	0.394799507
cg08465346	HOXC4	0.000357885	0.394799414
cg14411873	GABRA2;GABRA2	1.96E-05	0.394794644
cg15506609	HOXA9	0.001376172	0.394792912
cg16389399		8.60E-06	0.394792775
cg17313269	TRIM38	7.23E-05	0.394778171
cg16622906	PCDHB6	0.000791389	0.394760356
cg12865675	CANX;CANX	5.59E-05	0.394757541
cg23718606	PRSS16	8.60E-06	0.394746702
cg00065905	NHLH2;NHLH2	7.23E-05	0.394731888
cg04272011	NRXN3;NRXN3;NRXN3;NRXN3;NRXN3	7.23E-05	0.394725025
cg24410255		1.11E-05	0.394717198
cg20340866		7.23E-05	0.394695782
cg24660670		1.96E-05	0.394689933
cg07055315	NLRC4	0.000117986	0.394687915
cg05210258	FGF14;FGF14	1.11E-05	0.394681247
cg14131824	SLC43A2	0.000117986	0.394678123
cg03351487	STK39	1.50E-05	0.394675672
cg06739107	LPP;LPP;LPP	0.000148457	0.394674108
cg01780493	SEMA3C	0.000187194	0.394662437
cg26282566	PCDHA2;PCDHA1;PCDHA1;PCDHA4;PCDHA3	0.000187194	0.394650953
cg10930308	RNF39;RNF39	0.000290372	0.394642458

cg19526659		1.50E-05	0.39461803
cg10241484	CDH8	1.11E-05	0.394609741
cg27264049		4.34E-05	0.394603031
cg18765366	DOC2B	0.000187194	0.394600319
cg12217400		4.34E-05	0.39458924
cg06545389	CCDC108;CCDC108	7.23E-05	0.394580359
cg24084422	HSPA4L	3.34E-05	0.394575204
cg19069553	ANXA3	1.50E-05	0.394574549
cg15454726		0.000187194	0.394558595
cg22519422		2.58E-05	0.39452746
cg17270257	PDE10A;PDE10A	9.24E-05	0.394526352
cg14041283		2.58E-05	0.394495585
cg09099868		0.000187194	0.394489954
cg06329574	FGF3	2.58E-05	0.394467706
cg10094651	IGFBP3;IGFBP3	0.001061731	0.394459864
cg14503291	FAM117A	0.005897668	0.394458897
cg23153481		0.000791389	0.394445693
cg01655008	C14orf109;C14orf109	7.23E-05	0.394443311
cg03349020	FBXL16	5.59E-05	0.394436149
cg16660547	VTI1A	3.21E-05	0.394428675
cg17525102	FOXG1	3.34E-05	0.394417412
cg24737761		4.34E-05	0.394305633
cg11324832	SOX2OT	2.58E-05	0.394275936
cg25203962		8.60E-06	0.394272149
cg12431517		0.000187194	0.394271089
cg05323533	PHOX2B	0.000187194	0.394256206
cg15876676		0.000148457	0.394250968
cg14448830		9.24E-05	0.394237138
cg08752433	PPTC7	0.000187194	0.394223293
cg26157386		1.50E-05	0.394203714
cg00614182	NRG1	8.60E-06	0.394189356
cg05542757	FREM2	1.50E-05	0.394183449
cg08086720		0.000117986	0.394180148
cg01153166	NR2F2;NR2F2;NR2F2;NR2F2	9.24E-05	0.39417496
cg23752923	MCOLN3	8.60E-06	0.394174035
cg19317211		0.000148457	0.394171164
cg12091396	C10orf35	3.34E-05	0.394163375
cg25725843	ST6GAL2;ST6GAL2	2.58E-05	0.394158144
cg21339923		0.000233422	0.394155936
cg12419052	ZIC1	3.34E-05	0.394151247
cg26994225		1.96E-05	0.394092075
cg13411789	GCNT2	0.000537905	0.394091681
cg10462778	ERGIC1	3.34E-05	0.394071263
cg02728558	EIF5A2	0.000290372	0.394064868
cg20265733	GATA5	0.000654035	0.394064545
cg15205435	CHD5	0.005897668	0.394063254
cg20941110	BNC1	1.50E-05	0.394061834
cg15193473	BIVM;BIVM	0.000357885	0.394060864
cg01980222	TREM2;TREM2	3.34E-05	0.394032805
cg10000424	WNT6	0.000357885	0.394011118
cg14449051	SLC6A15;SLC6A15;SLC6A15;SLC6A15;SLC6A	8.60E-06	0.39400369
cg14663451	NRG1;NRG1;NRG1;NRG1;NRG1;NRG1;NRG1;	8.60E-06	0.39398949
cg06562372	ABCB1	2.58E-05	0.393989211
cg26981076	MS4A3;MS4A3;MS4A3;MS4A3;MS4A3;MS4A3	5.59E-05	0.393979159
cg15548427	MFSD1;MFSD1	0.003189589	0.393956755
cg13682477	TTPA	9.04E-05	0.39395512
cg22807585		0.000233422	0.393925071
cg04562589		1.11E-05	0.393921343
cg19163194	CACNG4	5.59E-05	0.393918891
cg18888137	BRD2;BRD2	1.96E-05	0.393916764

cg20963227		5.59E-05	0.393916073
cg26107850	HIST3H2BB;HIST3H2A	1.96E-05	0.393915947
cg15312264	TBPL2	9.24E-05	0.393915015
cg13675051	RNF19A;RNF19A	5.59E-05	0.393877637
cg00969119		7.23E-05	0.39386951
cg05870586	HDAC4	4.34E-05	0.393864354
cg10659805	DLX6AS	0.000117986	0.393847153
cg25463470	SOX1	0.000654035	0.393828772
cg01050423	SLC9A2	0.000187194	0.393817626
cg08008931	NRXN1;NRXN1;NRXN1;NRXN1	7.23E-05	0.393813415
cg02770534	PAX2;PAX2;PAX2;PAX2;PAX2	2.58E-05	0.393805652
cg23210971	AFF1;AFF1	4.34E-05	0.393801316
cg04747322	SNCAIP	1.50E-05	0.393801192
cg06658468	OTOP1	4.34E-05	0.393775052
cg25977958	SYNE1;SYNE1	5.59E-05	0.393734426
cg24825966	RFX4	7.23E-05	0.39372056
cg21231400		3.34E-05	0.393692695
cg20192747		1.11E-05	0.393671338
cg26707845	ODZ4	4.34E-05	0.393659632
cg00297075		2.58E-05	0.393646276
cg19969733	IL1RL1	0.001639597	0.393632432
cg07045816	IHH	0.000158823	0.393619354
cg10331779	CTNND2	1.11E-05	0.393610114
cg12393697	FAM171B	0.000148457	0.393608919
cg26209058	EIF5A2	7.23E-05	0.393596435
cg09926747	COL25A1;COL25A1	1.11E-05	0.393556707
cg12598803	EIF5A2	9.24E-05	0.393545984
cg14102128	SEPT10;SEPT10;SEPT10;ANKRD57;SEPT10	4.34E-05	0.39354013
cg08334899	LOC401463	3.92E-05	0.393527589
cg05849676	CLEC12B	0.001376172	0.393520335
cg04086531	ZNF423	1.96E-05	0.393501886
cg25742246		3.34E-05	0.393468377
cg25098208	RIC3;RIC3	1.60E-05	0.393465018
cg10348756	EPHX2;EPHX2	1.96E-05	0.393459388
cg11437253		8.60E-06	0.393456614
cg20209308	GSC2	1.96E-05	0.393411139
cg26776551	INTS6;INTS6	4.34E-05	0.393373243
cg12118843		0.000148457	0.393350301
cg19736503	RIPPLY2	8.60E-06	0.393340698
cg13128937		2.58E-05	0.393338505
cg04245373	CALB2;CALB2;CALB2	0.000187194	0.39330131
cg20869501	ESRRG;ESRRG;ESRRG;ESRRG;ESRRG	5.59E-05	0.393300422
cg07180307	C11orf87	2.58E-05	0.393295572
cg13890706	GFRA1;GFRA1;GFRA1;GFRA1	0.00043997	0.393295401
cg24071532	TMEM132E	8.60E-06	0.393290066
cg19929126	TRIL	0.001147425	0.393286111
cg13932501	AUH	2.58E-05	0.393277964
cg11215976	ACTN2;ACTN2	0.000117986	0.393224302
cg00777445		1.50E-05	0.39322061
cg18351939		5.59E-05	0.393211186
cg14350197	BRUNOL4;BRUNOL4;BRUNOL4;BRUNOL4	0.000117986	0.39318268
cg10536898		2.58E-05	0.393170334
cg19523213	KCNJ8	0.000537905	0.393153226
cg07362969	TTPA	7.23E-05	0.39312401
cg23989963	BNC1	3.34E-05	0.393107671
cg00387704	LEPREL1;LEPREL1	1.50E-05	0.393098734
cg13852284	C10orf11	0.000148457	0.393097437
cg00911351	PCDHGA4;PCDHGA6;PCDHGA1;PCDHGA5;PC	1.96E-05	0.393093611
cg06644373	SATB2	0.000654035	0.3930796
cg19412031		0.000233422	0.393077105

cg26330518	NEFM	0.000117986	0.393067475
cg02811232		0.00043997	0.393064728
cg22481695		0.000233422	0.393009669
cg26069919		1.50E-05	0.393001288
cg22648135	PCDHGA4;PCDHGA6;PCDHGA1;PCDHGA5;PC	1.96E-05	0.392988287
cg01501819	KCNA7	9.24E-05	0.392964275
cg01054402	TGOLN2	0.000290372	0.392956155
cg14265823	PAX3;PAX3;CCDC140;PAX3;PAX3;PAX3;PAX3	2.58E-05	0.392951619
cg20917891	DCC	8.60E-06	0.392931982
cg07077013		8.60E-06	0.392931561
cg03202564		8.60E-06	0.392920139
cg19977966	HOXC13	1.18E-05	0.392868033
cg03506028		1.11E-05	0.392825613
cg05674602		1.96E-05	0.392822304
cg26198463	TRPA1;TRPA1	8.60E-06	0.392813818
cg08755224		8.60E-06	0.392802143
cg15240082	UPK3A;UPK3A	0.000791389	0.392788963
cg22687807	LYNX1;LYNX1;LYNX1;LYNX1	0.000233422	0.392788256
cg06920037		1.96E-05	0.392756381
cg09257796	GABRG3	0.000148457	0.392716497
cg14809191	FOXQ1	8.60E-06	0.392701047
cg09887220		1.50E-05	0.392691837
cg11691561		5.59E-05	0.392678336
cg02111032		2.58E-05	0.392666402
cg20439889	CHST8;CHST8;CHST8;CHST8	8.60E-06	0.392639711
cg14547461	KIAA0146	1.96E-05	0.392628324
cg05520409	SOX8	4.34E-05	0.392626983
cg02707176	PCDHGA4;PCDHGA9;PCDHGA1;PCDHGB1;PC	0.000148457	0.392621176
cg03943081	TCERG1L	8.60E-06	0.392614805
cg22331862	SLC6A11	8.60E-06	0.392606918
cg14426785	TGFBR1;TGFBR1	2.58E-05	0.392602423
cg13348877	PARD6G;PARD6G	0.006823935	0.392587558
cg24074033	DBX1	5.59E-05	0.392584758
cg02078690	BSX	0.000148457	0.392561472
cg01882866		3.34E-05	0.392558005
cg00764612	C1orf51	1.11E-05	0.392557955
cg19584136	MXI1;MXI1	1.96E-05	0.392530703
cg11639615		0.000187194	0.39252697
cg12122146	TMEM132D	5.59E-05	0.392521781
cg03534058	CDC14B;CDC14B	4.34E-05	0.392508396
cg07116712		0.00043997	0.392466831
cg06733419	TBX18	1.11E-05	0.39244585
cg14243481	SYT9	7.23E-05	0.392441833
cg21314480	TACR1;TACR1	7.23E-05	0.392432086
cg04568492	ACTA1	7.23E-05	0.392423084
cg21700214	CNDP1	1.50E-05	0.39241957
cg09157302	SLC34A2	0.000537905	0.392408334
cg24468070	CDC42EP5	0.000148457	0.392402334
cg11867257	GPR98;GPR98	9.24E-05	0.392391121
cg15056773	SYNPR	9.24E-05	0.392382912
cg16475721		2.58E-05	0.392377292
cg08356262	IQGAP2	0.001147425	0.392364696
cg05756933	GPR6	1.50E-05	0.392353501
cg24099067		1.11E-05	0.392352515
cg26583481	CALY	0.000187194	0.392350445
cg13384781		2.58E-05	0.392346092
cg02657836		1.50E-05	0.392303097
cg17312004		3.34E-05	0.392299882
cg24458474	GRID1	0.000148457	0.392297066
cg06680852		0.00043997	0.392288469

cg08053686		1.96E-05	0.392278822
cg09988853	KCNC3	0.000205664	0.392266553
cg04549162	GPR26	8.60E-06	0.392256275
cg19161124	DPP6	1.11E-05	0.392249118
cg09990962	PRDM16;PRDM16	0.000233422	0.392212944
cg12249234	KSR1	0.000148457	0.392172762
cg26998274	SLITRK1;SLITRK1	0.000148457	0.392091365
cg11710969	ARMC3	1.50E-05	0.392090308
cg02480199		2.58E-05	0.392079141
cg16786808	EPHA4	0.000233422	0.392064129
cg19144684		9.24E-05	0.392044239
cg03440799	C1orf125;C1orf125	3.34E-05	0.392042665
cg13409645	COX6B2	0.000148457	0.392001403
cg00154357	ZIC4;ZIC4;ZIC4;ZIC4;ZIC4	8.60E-06	0.391998665
cg08283882	EBF2	0.000357885	0.39199665
cg22105145	LOC642597	1.11E-05	0.391984762
cg10176110	SMOC2;SMOC2	0.000233422	0.39196868
cg02562740	SLC19A3	1.11E-05	0.39196661
cg08874512	IGLON5	8.60E-06	0.391962478
cg27096144	MSX2	8.60E-06	0.391953178
cg05335886	TMC5;TMC5	1.50E-05	0.391945334
cg17294725	UNCX	3.34E-05	0.391940809
cg27468145	AQP4;AQP4	2.58E-05	0.391940307
cg26929700	ZNF423	7.23E-05	0.391936406
cg03486986		0.000117986	0.391919887
cg03076319	MLPH;MLPH	4.34E-05	0.391914329
cg14597534		9.24E-05	0.391914022
cg12317601	EMX2;EMX2OS;EMX2	0.000117986	0.391895734
cg26187205		0.000791389	0.391886167
cg18876826	INPP5B	0.000187194	0.391880579
cg14912728	PMP22;PMP22;PMP22	0.000290372	0.39187292
cg01563031	NELL1;NELL1	0.001147425	0.391853852
cg12276123	KCNJ3;KCNJ3	5.59E-05	0.391840236
cg08480223	LIN7A	3.34E-05	0.391833277
cg12700145		0.000148457	0.391818369
cg11612786		1.11E-05	0.391798172
cg20672711		7.94E-05	0.39178157
cg21213973		0.000290372	0.391775851
cg04645914		4.34E-05	0.391755634
cg18308184	KCNIP4;KCNIP4	1.50E-05	0.391729356
cg25771677		7.23E-05	0.391728678
cg11231249	CTNNA2;LRRTM1;CTNNA2	3.21E-05	0.391710129
cg00670915	RPL31;RPL31;RPL31	8.60E-06	0.391707772
cg10201297	ZBTB8B	8.60E-06	0.391686882
cg22891988	MPPED2	0.000187194	0.391654967
cg08873805	ZSCAN18;ZSCAN18	0.000187194	0.391636492
cg14940260		3.34E-05	0.391615378
cg10063179	MTNR1A	8.60E-06	0.391607058
cg17847723		5.59E-05	0.391571235
cg02601475		1.50E-05	0.391569992
cg08697503	CCDC140	1.11E-05	0.391566764
cg16998950	ELL2	0.000290372	0.39153932
cg15927196	PON3	2.58E-05	0.391530048
cg09768093	ZBTB8B	1.50E-05	0.391527166
cg25333258	CAMK2B;CAMK2B;CAMK2B;CAMK2B;CAMK2B	0.00043997	0.391523525
cg20274950		0.002304201	0.391523179
cg19157034		7.23E-05	0.391521999
cg05897809		1.50E-05	0.391482609
cg18190187	DRD1	7.23E-05	0.391468709
cg27391267	ZSCAN1	2.58E-05	0.391463069

cg07714657	WNT6	0.000654035	0.391454504
cg14492293	LHX3;LHX3	7.23E-05	0.391449266
cg22509539		8.60E-06	0.391443524
cg21282131	MAP7	0.001147425	0.391442234
cg21649051	MCOLN3	7.23E-05	0.391438782
cg06758255	TOX3;TOX3	0.004357816	0.391432822
cg08292023	GRM5;GRM5	3.34E-05	0.39143029
cg00323305	THRB;THRB;THRB	0.002304201	0.391417487
cg07769015	SLC45A4	1.50E-05	0.391412234
cg08773226		3.34E-05	0.391389089
cg00333154		0.000233422	0.391375692
cg26679004	GRID1	4.34E-05	0.391349827
cg10074544	COL25A1;COL25A1;COL25A1;COL25A1	8.60E-06	0.391343164
cg15025103		0.00043997	0.391320052
cg18839637	PCDHGA2;PCDHGA3;PCDHGA3;PCDHGA1	1.11E-05	0.391314264
cg13481132	KCNT1	0.000290372	0.391304498
cg26254045	SPRED3	8.60E-06	0.391276854
cg16142855		0.000117986	0.391258348
cg27021357	SH3GL3;SH3GL3	8.60E-06	0.391255691
cg02467990	VWC2	0.002304201	0.39124212
cg05726764	USP13	1.11E-05	0.391236261
cg00841693	MNX1;MNX1;MNX1	1.96E-05	0.391234802
cg03710481	PKIB;PKIB;PKIB;PKIB;PKIB	0.000187194	0.391233903
cg18888464		2.58E-05	0.391222021
cg13323701	SLC35F1	1.11E-05	0.391210959
cg27090062		0.000654035	0.391199513
cg07962392	DSCAM	1.50E-05	0.391177238
cg05085230	EYA4;EYA4;EYA4	0.000233422	0.391165582
cg23258615	ANXA11;ANXA11;ANXA11	2.58E-05	0.391163317
cg02923706		8.60E-06	0.391156591
cg12547959	TRIO	3.34E-05	0.391142165
cg02327997	ADAM12;ADAM12	8.60E-06	0.39114067
cg02540477	SMOC2;SMOC2;SMOC2;SMOC2	0.000290372	0.391129089
cg11723848	UNC5C	0.000233422	0.391113177
cg02943604	PPAPDC1A	1.11E-05	0.391090617
cg06314761	SYNPR	1.96E-05	0.391088751
cg01081737	SLC45A4	0.000187194	0.391086175
cg14385245	BNC1	2.58E-05	0.391083005
cg04661457		0.000233422	0.391082117
cg20059140	GNAL;GNAL;GNAL	3.34E-05	0.391070063
cg16579438	THRB;THRB;THRB	0.000537905	0.391061316
cg07185131		0.00043997	0.391038533
cg18944010	TBX15	1.96E-05	0.391003702
cg25057743	PTH2R	8.60E-06	0.390962121
cg07605211	SUSD4;SUSD4	0.000187194	0.390957263
cg01609420		1.96E-05	0.390950303
cg22350070	CTNNA2;CTNNA2	0.000290372	0.390937031
cg09809932	ESPN	0.000357885	0.390927782
cg15159605	LHX5	0.00045851	0.390927765
cg24778248	CNGA3;CNGA3	3.34E-05	0.390920787
cg24005685	PITX2;PITX2	9.24E-05	0.390918257
cg01617317	MS4A3;MS4A3;MS4A3;MS4A3;MS4A3;MS4A3	5.59E-05	0.390877577
cg22695532		2.58E-05	0.390870601
cg09385093		0.000117986	0.390843857
cg18505401	TMEM196	5.11E-05	0.390838023
cg09010699		2.58E-05	0.390817417
cg18097532	ABCC8	9.24E-05	0.39080913
cg02520281	PCDHB17	1.50E-05	0.390806587
cg21629500	C5orf38	0.005897668	0.390747606
cg17398595	SH3GL2	4.34E-05	0.390697908

cg06692537	TMEM229A	1.96E-05	0.390695338
cg22489321		0.000537905	0.390683124
cg01962428	DKK2;DKK2	0.000526175	0.390663035
cg16175713	CYP39A1	0.001147425	0.390659562
cg25095722	LRRN1	0.000117986	0.390653097
cg22316269		0.000148457	0.390645569
cg07122178	SFRP1	8.60E-06	0.390634735
cg02717150		0.000537905	0.390616729
cg08080029	CHD5	0.000290372	0.390615476
cg18612589	CLDN7	2.58E-05	0.390610768
cg08765317	WWC1;WWC1;WWC1	2.58E-05	0.390590719
cg08230059	TPBG;TPBG	0.000290372	0.390585028
cg09883286	USP44;USP44	7.23E-05	0.390577266
cg18294707	RNASE2	7.23E-05	0.390562101
cg00404641	NUDT16P;NUDT16P	0.000148457	0.390552469
cg07360792	PRDM6	0.000357885	0.390546805
cg01922095	LOC650226	1.96E-05	0.390518066
cg26562772	DUSP10;DUSP10;DUSP10	0.000187194	0.390509534
cg26768712	SCNN1B	4.34E-05	0.39050614
cg02970297	DPY19L2P2;DPY19L2P2	8.60E-06	0.390498268
cg10878114	SLC35F3	0.000117986	0.390493248
cg01913568	RXRG;RXRG	0.000205664	0.390488297
cg21726372	PITX2;PITX2;PITX2	9.24E-05	0.390478893
cg07136133	PRR5L;PRR5L;PRR5L;PRR5L	7.23E-05	0.390449168
cg04789529	DOCK5	1.96E-05	0.390444439
cg22117918	SLC22A3	7.23E-05	0.390439084
cg18793404		4.34E-05	0.390419978
cg14227486		1.50E-05	0.390415442
cg13948082	CYP26B1	1.11E-05	0.3904131
cg02269161	SYT9	1.96E-05	0.390398405
cg11042561		5.59E-05	0.390357832
cg14346208	PAMR1;PAMR1	4.34E-05	0.390349402
cg05924485		1.50E-05	0.390336205
cg05699739		0.000233422	0.390329907
cg00986824	KCNC2;KCNC2;KCNC2	1.96E-05	0.390314695
cg13525837	KCNV1	1.50E-05	0.390304602
cg13857354		0.000537905	0.390300828
cg19861138	RPRM	0.000148457	0.390300519
cg26502666	SPDYA;SPDYA	7.23E-05	0.390299754
cg24293903	ENTPD7	0.000187194	0.390272779
cg23016734	ROBO2	1.96E-05	0.390252162
cg12091542	CELSR3	8.60E-06	0.390250068
cg12743460		2.58E-05	0.39024895
cg13924432		1.11E-05	0.390215975
cg22378919	TBX15	2.58E-05	0.390213635
cg07210669	S100P	0.000955793	0.390167087
cg20498414	RIMS4	0.000187194	0.39016277
cg04351049	LMX1A	0.000117986	0.390140753
cg15431821	KIAA1024	3.34E-05	0.390129856
cg07676361		0.000233422	0.390105679
cg24708006		8.60E-06	0.390105099
cg10916569	BNC2	0.000117986	0.39007702
cg07636117		1.50E-05	0.390076338
cg21856067	MACROD2	2.58E-05	0.390066613
cg23260547	PROX1	0.00043997	0.390064937
cg14732248	FOXQ1	8.60E-06	0.390030335
cg21063722	SIM1	0.000654035	0.390026263
cg03305017		0.000537905	0.390013292
cg13212435		8.60E-06	0.39000233
cg27648738	SH3GL3;SH3GL3	0.000357885	0.389996895

cg12954230	ADAMTS17	0.001948248	0.389976871
cg07822928	HCN2	1.50E-05	0.389969202
cg12969595	TMEM98;TMEM98	8.60E-06	0.389961919
cg23198559	NPNT	8.60E-06	0.389958619
cg14264795	TBX5;TBX5;TBX5	1.50E-05	0.389958301
cg18263365	SPOCK1	1.96E-05	0.389901451
cg00100800	ZNF462	0.004357816	0.389898149
cg07561747	KIF21B	3.34E-05	0.389892473
cg22823146	EIF4E3;EIF4E3;GPR27	7.23E-05	0.389892394
cg13012916	SFTA3	0.000233422	0.389890869
cg10253847	NKAPL	0.000357885	0.389866264
cg21174055	LHX3	0.000290372	0.389856521
cg20208600	TMEM216	0.000233422	0.38985453
cg06132283		8.60E-06	0.389848296
cg27216259	FAM46A	0.000955793	0.389838843
cg14061619	SH3BP4	4.34E-05	0.389835695
cg26170569		7.23E-05	0.389820179
cg07848601	RANBP17	1.96E-05	0.389814721
cg27192248		0.001376172	0.38979979
cg17362052	DKK2	1.50E-05	0.389799235
cg18991611		0.003486652	0.389796305
cg25201881	SYT6	0.000187194	0.389781979
cg03204605	AFAP1;AFAP1	0.000791389	0.389779809
cg23500122	GHSR;GHSR	5.59E-05	0.389778059
cg01047586		0.000117986	0.38977654
cg00208967	OLFM2	0.000791389	0.389760155
cg16472050		9.24E-05	0.389759577
cg27635069	NGF	3.34E-05	0.389747556
cg23130131	PITX2;PITX2;PITX2	3.34E-05	0.389746999
cg18567682		0.000117986	0.389739182
cg08625556		0.000117986	0.389710389
cg01582980	SP8;SP8	0.000290372	0.38967858
cg23389465		5.59E-05	0.389663498
cg23508667		0.000117986	0.389650011
cg07777349		0.000537905	0.389643478
cg09157668	KIAA1239	3.34E-05	0.389638814
cg26790247	ZIC4;ZIC4;ZIC4;ZIC4;ZIC4	3.34E-05	0.389636907
cg07741016	VTRNA1-3	9.24E-05	0.389633279
cg03166753		0.000205664	0.389610952
cg09341793	ASB2	0.004357816	0.389595382
cg24575676	ZNF385B;MIR1258;ZNF385B	9.24E-05	0.389591351
cg22090773	NCAN	8.60E-06	0.389558067
cg17039391	DKK3;DKK3;DKK3	0.000187194	0.389550602
cg22643811	FAM19A5	8.60E-06	0.389539993
cg05495949	RADIL	7.23E-05	0.389508484
cg04080282	PON3	4.34E-05	0.389507764
cg00059225	GLRA1;GLRA1;GLRA1;GLRA1	1.11E-05	0.389486186
cg00908526	CTNND2;CTNND2	2.58E-05	0.38947189
cg25701418	MAGI1;MAGI1;MAGI1	0.000233422	0.389447056
cg03736795		0.000148457	0.389439533
cg09617579	FAM181B;FAM181B	0.000357885	0.38941764
cg03925294		8.60E-06	0.38938318
cg18956933		0.000148457	0.389373626
cg17444904	PRR15	0.00043997	0.389372227
cg04890607	HMGA2;HMGA2	1.96E-05	0.389342942
cg03223072	ABLIM1;ABLIM1;ABLIM1	1.11E-05	0.389319496
cg19037107	ACAD8	3.34E-05	0.389264236
cg10112017		4.34E-05	0.389248794
cg18160402	SHISA6	5.59E-05	0.389188657
cg14489474	ADCYAP1;ADCYAP1	8.60E-06	0.38918254

cg25797887	DLX6AS	0.000117986	0.389178335
cg11100804	PCDHGA4;PCDHGA6;PCDHGA1;PCDHGA8;PC	3.34E-05	0.389148376
cg19678828	CYYR1;CYYR1	5.59E-05	0.389136652
cg24856726	ASCL4	5.59E-05	0.389132823
cg14563954		4.34E-05	0.389125521
cg15287806	ZMYND11;ZMYND11;ZMYND11	1.96E-05	0.389122142
cg20350518		2.58E-05	0.389120766
cg01963134	CPNE8;CPNE8	0.0050758	0.389119143
cg10042106	OTX2	4.34E-05	0.38905517
cg12878812	SRRM4	8.60E-06	0.389010804
cg08913523		0.000148457	0.389006684
cg10083898		1.50E-05	0.389004394
cg04432319	ISM1	0.002304201	0.388984653
cg15745900	PREX2;PREX2	7.23E-05	0.388981925
cg02010481	JAZF1	0.000955793	0.388963524
cg20977312	STC2	9.24E-05	0.38894966
cg02119792		0.001639597	0.388936505
cg02840109	CDH2	0.00041966	0.388934702
cg02445447	FHL2;FHL2;FHL2;FHL2;FHL2;FHL2	0.000117986	0.388929273
cg24033042	BRUNOL5	1.50E-05	0.388900902
cg18792904	TPBG;TPBG	0.001948248	0.388883563
cg21547690	LRFN5;LRFN5	9.24E-05	0.388855307
cg07991288	C20orf56	0.000187194	0.388853125
cg09214112		8.60E-06	0.388847079
cg05184377		8.60E-06	0.388843872
cg00745725	GABRG3	4.34E-05	0.388836464
cg23913421		1.50E-05	0.388822257
cg13324546	NKX2-6	5.59E-05	0.388817718
cg26444097		3.34E-05	0.388817261
cg26654934		0.000148457	0.388811564
cg10227191	KRT18;KRT18	4.34E-05	0.388793748
cg06872705	LMX1A	3.34E-05	0.388788094
cg22681934	NCAN	0.002304201	0.388780546
cg00002719	C1orf114	0.000262362	0.388774206
cg01867395	PAX6	3.34E-05	0.388728287
cg03574651	SLC30A9	0.000148457	0.38870187
cg06657142	FGFR2;FGFR2;FGFR2;FGFR2;FGFR2	7.23E-05	0.388690177
cg27020216	SGEF	0.000117986	0.388645362
cg15081351	MYO3A	1.50E-05	0.388614387
cg12298905	PROX1	0.001948248	0.388607871
cg10506318	ERCC8;NDUFAF2	0.003734675	0.388591367
cg01558916		5.59E-05	0.388578188
cg11342452	NKX6-2	1.50E-05	0.388550574
cg25648436	ZSCAN1	8.60E-06	0.388538607
cg01811815	RASA3	9.24E-05	0.388537542
cg22762844	MAL;MAL;MAL;MAL	4.34E-05	0.388514688
cg05328547	FERD3L	1.96E-05	0.38849353
cg10084644	STAG3;GPC2	0.000187194	0.388468013
cg26983198	TNNT3;TNNT3;TNNT3;TNNT3	0.000290372	0.388451607
cg12190994	SDK1;SDK1	0.000791389	0.38844365
cg06338421		1.50E-05	0.388439064
cg27385032	ZNF334;ZNF334	1.96E-05	0.388425019
cg16564824	SNAP25;SNAP25;SNAP25;SNAP25	1.11E-05	0.388415841
cg02869486		1.11E-05	0.388403881
cg06374962		0.00043997	0.388394404
cg08280383	GPR139	8.60E-06	0.388392428
cg06091013	FBXO39	0.000117986	0.388378987
cg22071805	PNPLA3	4.34E-05	0.388368917
cg09266149	ZNF697	0.000791389	0.388328781
cg01830294	WNT2;WNT2	1.96E-05	0.388274621

cg02011392	GRIK2;GRIK2;GRIK2	0.000233422	0.388269512
cg10864878		0.000148457	0.388232763
cg07871947	SLC12A5;SLC12A5	3.34E-05	0.38821909
cg24120696		0.000357885	0.388208672
cg18115215	TMEM108;TMEM108	0.00043997	0.38819796
cg26195356		0.001376172	0.388179133
cg26948603	DRD1	7.23E-05	0.388173844
cg04457069		0.000148457	0.388170803
cg27492467	PPP2R2C	0.000233422	0.388170655
cg11608958		3.34E-05	0.388164115
cg14898260	CA8	0.00043997	0.388155582
cg24676071	ADCY1	0.001376172	0.388153561
cg27036111	SLC6A5	8.60E-06	0.388138654
cg14778074	GABRA2;GABRA2	8.60E-06	0.388118179
cg06340987	DBX1	5.59E-05	0.388088398
cg16752876	OSR1	2.58E-05	0.388072606
cg16323245	ZBTB20;ZBTB20	0.002714607	0.388056327
cg27032146	DLX5	0.001376172	0.388055275
cg01148441	MPPED1	0.000233422	0.388007683
cg11957400	CACNA2D4	1.11E-05	0.388007307
cg24631970	XKR6	0.00043997	0.38799635
cg11354629	GSX1	9.24E-05	0.387964723
cg20728105	SCIN	7.23E-05	0.387963502
cg15842216	HMCN1;HMCN1	9.24E-05	0.387961575
cg19620994	AADACL3;AADACL3	0.000148457	0.387948585
cg10269365	CCDC140	1.50E-05	0.387934935
cg06942701	TBR1	1.96E-05	0.387927154
cg21534264	CADPS;CADPS;CADPS	8.60E-06	0.387921605
cg13523557	ADCY1	0.000233422	0.387902966
cg22712983	PNKD;PNKD	1.50E-05	0.387890453
cg27416372	ZIC4;ZIC4;ZIC4;ZIC4;ZIC4	8.60E-06	0.387875419
cg24442454	MDGA1	2.58E-05	0.387868701
cg14749465	NELL1;NELL1	0.000537905	0.387866038
cg19229344	NKX2-3	0.000955793	0.38785636
cg10593922		0.000537905	0.387832196
cg20417527	SLC27A6;SLC27A6	1.96E-05	0.387798067
cg23565942	NEBL	9.24E-05	0.38778956
cg06977689	PARD6G	0.000187194	0.387789258
cg18786535	CCDC67	0.000187194	0.387770584
cg21252105		3.34E-05	0.387764223
cg13837721		5.59E-05	0.387747039
cg23493016	SORCS3	1.11E-05	0.387743704
cg00920970	ESR1;ESR1;ESR1;ESR1	9.24E-05	0.387700398
cg22769941	NELL1;NELL1	0.001147425	0.387681393
cg03024537		8.60E-06	0.387679872
cg17069914		0.000955793	0.387673509
cg04931256		5.59E-05	0.387664011
cg07969984	CALCR;CALCR	2.58E-05	0.387657945
cg03304380	DLC1	0.000233422	0.387655761
cg01489686	FUT9	8.60E-06	0.38764964
cg13663300	GAS2;GAS2	1.50E-05	0.387648901
cg03350900	SOBP	4.34E-05	0.387639071
cg25506797		0.003189589	0.387638753
cg14427009	PCDH17	8.60E-06	0.387631632
cg11438428	PTF1A	8.60E-06	0.38761802
cg07688490	TMEM45B	9.24E-05	0.387612157
cg02196294	KCNK5	0.000791389	0.387601536
cg09455342		1.50E-05	0.387550292
cg21462844	SGK3	0.000122184	0.387525908
cg14867569	NKX2-1;NKX2-1	0.000117986	0.387509218

cg22491141		9.24E-05	0.387489318
cg22496102	CRABP1;CRABP1	0.00043997	0.387476671
cg03986829	FEZF2	0.001376172	0.387467052
cg19291576		0.000117986	0.387447105
cg23146534	C7orf38	1.96E-05	0.387439598
cg22939193	WDR35;WDR35	7.23E-05	0.387438811
cg06511917	PTF1A	1.50E-05	0.387424364
cg00504690	ROR1;ROR1	0.002450672	0.387421604
cg21141234		1.11E-05	0.387416482
cg02573176	SLC10A5	0.003189589	0.387402667
cg03244690	TMEM20;TMEM20	7.23E-05	0.387391416
cg13021841	FAM84A	2.58E-05	0.387334831
cg08158952	ZNF835	2.58E-05	0.387328364
cg27088150		0.000654035	0.387302979
cg10904109	GRM1;GRM1	8.60E-06	0.387294462
cg05714732		1.11E-05	0.387282495
cg02383154	HLF	4.34E-05	0.387280591
cg06778429	DRD2;DRD2	1.96E-05	0.387262719
cg20661985	C20orf3	0.00043997	0.387258845
cg03074925		1.11E-05	0.387241252
cg24978630		0.00043997	0.387234676
cg09576143	HTRA1	0.000290372	0.387204805
cg00454592		2.58E-05	0.387163366
cg15617155		5.59E-05	0.387137254
cg02330121	SLC35F1	1.96E-05	0.387131757
cg27118761	KCNC1;KCNC1	0.000955793	0.387127617
cg21671806	RALYL;RALYL;RALYL;RALYL	1.50E-05	0.387126029
cg05537380	SHC2	9.24E-05	0.387100484
cg14685796		5.59E-05	0.387081927
cg08400962	CHST8;CHST8	1.11E-05	0.387076397
cg03480337		0.000357885	0.387065331
cg21873251		0.002304201	0.387060629
cg06757585	PCDHGA4;PCDHGA1;PCDHGA6;PCDHGA5;PC	0.000233422	0.387052301
cg13451025	FAM150A	2.58E-05	0.387034979
cg14191024		2.58E-05	0.387034913
cg04961235	PAPOLB;RADIL	1.96E-05	0.387033379
cg16646024		4.34E-05	0.387015734
cg16374656	EXT1	4.34E-05	0.387000627
cg06476463		1.96E-05	0.386984579
cg12351433	LHCGR	0.000117986	0.386978991
cg02619205	AKNA	5.59E-05	0.38697562
cg21845273		3.34E-05	0.38697253
cg20163166	LRIG3;LRIG3	8.60E-06	0.386970077
cg26640601		0.00043997	0.386965001
cg19721889	HAND1;HAND1	1.11E-05	0.386946966
cg01402255	GATAD2B	2.58E-05	0.386922705
cg04456916		8.60E-06	0.386886515
cg06867755	CUL3	3.34E-05	0.38688385
cg14717796	EPHX2;EPHX2	9.24E-05	0.386881396
cg13761440	MPPED2	0.000233422	0.38686251
cg12053762	RIC8B	5.59E-05	0.386819996
cg24849872	ZNF287	8.60E-06	0.386808969
cg15717926	RFX6	7.23E-05	0.386798819
cg18451022	CYP1B1;CYP1B1	1.96E-05	0.386798414
cg09268729		7.23E-05	0.386798345
cg14089267		7.23E-05	0.386795546
cg09606941	SLC40A1	3.34E-05	0.386793712
cg03442425	TMEM132E;C17orf102	7.23E-05	0.386793164
cg16086373	TMEM200C	0.000187194	0.386772338
cg16648355	ESRP1;ESRP1;ESRP1;ESRP1;ESRP1	0.000117986	0.386727478

cg15541630	PSKH2	7.23E-05	0.386722578
cg14377594	DMRT2;DMRT2;DMRT2	1.50E-05	0.386701763
cg22637435	TRAPPC9;TRAPPC9	5.59E-05	0.386700158
cg16842423	GALNTL4	8.60E-06	0.386681664
cg13950603	CLEC4G	0.000117986	0.386672724
cg00509108	MT1A	0.001147425	0.38667117
cg17361203	DSCAML1	2.58E-05	0.386666857
cg21984150	SNCAIP	0.000290372	0.38666412
cg02108033	FAM184B	0.000357885	0.386663804
cg07150045	KCNH7;KCNH7	8.60E-06	0.386647194
cg15794859	TCF21	7.23E-05	0.386625878
cg00457403	SST	2.58E-05	0.386623431
cg16349827	VWA3B	8.60E-06	0.386606406
cg25806307	FBXO17	1.96E-05	0.386605498
cg13397436	PDE4D;PDE4D	0.00043997	0.386589352
cg11204212		1.11E-05	0.386584046
cg27113440	ANO5;ANO5	2.58E-05	0.386578892
cg10372047	RNF180;RNF180	1.11E-05	0.386550315
cg01568784	RAB38	0.000537905	0.386543444
cg01572694	MIR10A	7.23E-05	0.386540747
cg25576997	C14orf34;C14orf34	0.000654035	0.386537543
cg11980129	ROBO1	8.60E-06	0.386535123
cg07147475		1.11E-05	0.386533756
cg08067566		0.000537905	0.386529939
cg24213507	GRM7;GRM7	0.000148457	0.386529492
cg05642546	ELMO1	0.000148457	0.386525978
cg04499648	MYOD1	2.58E-05	0.386505597
cg09109411	PDE6A	9.24E-05	0.38650115
cg10530281	TBX3;TBX3	0.000187194	0.386496226
cg15531403	NPBWR1	0.000955793	0.386468936
cg00430036	SLC35D2	5.59E-05	0.386451666
cg08878368	SALL1;SALL1	1.50E-05	0.386423074
cg25347098	PROX1	0.000955793	0.386414897
cg20947377	PTPRZ1	2.58E-05	0.386410215
cg05638287	KCNK1	1.96E-05	0.386398027
cg05550276	TOX3;TOX3	0.000148457	0.386378697
cg15123984	MSX2;MSX2	4.34E-05	0.386376828
cg06636427	UNCX	4.34E-05	0.386375203
cg11590772	ADAMTS10	1.11E-05	0.386374655
cg13570972	PAX6	1.96E-05	0.386369757
cg19600750	OTX2	1.50E-05	0.386368172
cg05152589	CADM2;CADM2;CADM2;CADM2	3.34E-05	0.386359991
cg05931096	PTPRZ1	1.50E-05	0.386357587
cg22995692		5.59E-05	0.386356076
cg27392792	PTPRN2;PTPRN2;PTPRN2	9.24E-05	0.386351033
cg06193578		1.96E-05	0.38634849
cg18742346	PENK;PENK	0.000117986	0.386343113
cg08104202	C1orf114	0.000148457	0.386340482
cg22168369	MCOLN2	0.000537905	0.386325659
cg27527630	WIPF3	4.34E-05	0.38629
cg00661970		0.000117986	0.386279157
cg27630311	TBX3;TBX3	4.34E-05	0.386277509
cg15180867	TCF15	3.34E-05	0.386267671
cg02483449		5.59E-05	0.386237206
cg02086467	OTX2	3.34E-05	0.386229147
cg05748163	EBF2	1.96E-05	0.386215152
cg13564742	FOXE1	1.96E-05	0.386211143
cg08110542		0.00043997	0.386195075
cg16810605		0.000187194	0.386175699
cg00032205	TSPYL5	0.001147425	0.386150819

cg26143452	TBPL2	4.34E-05	0.386148537
cg20641748		8.60E-06	0.386132421
cg05214235	TEAD1	0.000117986	0.386125884
cg25539505		4.34E-05	0.386124821
cg04823311	TRIL	0.002304201	0.386122635
cg22941668	MIR145;LOC728264	5.59E-05	0.386121416
cg19542987	ARMC3	9.24E-05	0.386120469
cg20525917	GATA5	9.24E-05	0.386116336
cg06971092		5.59E-05	0.386111412
cg12690369	PTPRR	1.11E-05	0.38610683
cg11071401	CACNA1G;CACNA1G;CACNA1G;CACNA1G;CA	5.59E-05	0.386100242
cg20436262	MGC2889;HRASLS	1.96E-05	0.386078257
cg24803719		3.34E-05	0.386059528
cg15331039	SH3BGRL2	8.60E-06	0.386037717
cg14034757	CAMK1G	0.00043997	0.386032697
cg21105916	PCDHB19P	4.34E-05	0.386004615
cg05890727	FBXO34;FBXO34	3.34E-05	0.385977077
cg16648426		1.96E-05	0.385960637
cg11492723		5.59E-05	0.385948311
cg11176095	GPR126;GPR126;GPR126;GPR126	8.60E-06	0.385939305
cg01157404	RYR2	8.60E-06	0.385931535
cg19064523	TWIST1	2.58E-05	0.385925829
cg02743713		0.000148457	0.385911798
cg14511698	SIX6	1.11E-05	0.385900406
cg11837766	ROR1;ROR1	0.000233422	0.385867554
cg20624923	PRLHR	1.11E-05	0.385861023
cg16041412		0.000654035	0.385850013
cg15737290	CCDC67	1.50E-05	0.38583141
cg21776417		1.96E-05	0.385789473
cg13573745		0.000187194	0.385777178
cg00822495	OTX2	1.50E-05	0.385774598
cg22111527		3.34E-05	0.385766522
cg17389519	PTF1A	4.34E-05	0.385751338
cg12146386		0.000357885	0.385748053
cg10785443		0.00043997	0.385744577
cg13564533		4.34E-05	0.385722311
cg13422921		4.34E-05	0.385712909
cg04961466	NPY5R	1.50E-05	0.385707891
cg22123464	SLC8A2	2.58E-05	0.385706278
cg11038322	TFAP2D	0.000117986	0.385696805
cg25486757	PDZRN3	8.60E-06	0.385678674
cg18084609	COCH;COCH	8.60E-06	0.385650387
cg18669209		0.000233422	0.385619877
cg13016408	LYPD1;LYPD1	5.59E-05	0.385612274
cg19220719		0.0050758	0.385610394
cg02576468	SPTBN4	1.96E-05	0.385610238
cg27335600	CACNA1D;CACNA1D;CACNA1D	9.24E-05	0.385608557
cg05653707	EMX2;EMX2;EMX2OS	0.000117986	0.385606734
cg06763078	KCNC1;KCNC1	0.000290372	0.385595209
cg16671160		1.11E-05	0.385558406
cg10810799	HOXC12	4.34E-05	0.385547542
cg04529785	NOS1	0.000526175	0.385543174
cg11945839	ART5;ART5	0.000233422	0.385524603
cg14793086	LCA5;LCA5;LCA5;LCA5	0.000117986	0.385511953
cg06660395		8.60E-06	0.385495189
cg00101712	ROPN1	7.23E-05	0.385477207
cg16747714	KRT18;KRT18	0.000117986	0.385468093
cg26163880	MYH14;MYH14;MYH14	9.24E-05	0.385462175
cg21350115	CALCRL;CALCRL	1.11E-05	0.385452412
cg18525352	NUAK1	0.000117986	0.385418232

cg06779604	CADPS2;CADPS2;CADPS2;CADPS2;CADPS2;	0.000357885	0.385416947
cg02515725	PDLIM3;PDLIM3	8.60E-06	0.385368792
cg07678449	AADAT;AADAT	0.000233422	0.385354754
cg22534097	ANKRD1	0.000290372	0.385352925
cg06364757	PYGO1	8.60E-06	0.385311299
cg22401679		0.000537905	0.385300338
cg05486213	MPPED2	0.000187194	0.385268769
cg01667646	SH3GL3;SH3GL3	2.58E-05	0.385267994
cg16491960		7.23E-05	0.385264461
cg10456459	ETNK1	0.000233422	0.385259545
cg17443359		0.000955793	0.385258157
cg25527618	NDFIP2;NDFIP2	3.21E-05	0.385242123
cg00252615	GABRB2;GABRB2	8.60E-06	0.385233218
cg22851295	GCM2	1.96E-05	0.38521709
cg14711866	STOX2	0.000117986	0.385205482
cg04088945		7.23E-05	0.385191197
cg22797570		0.001147425	0.385183218
cg00896347		1.11E-05	0.385174663
cg00499700	F2RL1	7.23E-05	0.385154167
cg06324373	CRTAC1	5.59E-05	0.385141883
cg09469566		0.000357885	0.385133571
cg16399049	HS3ST2	3.34E-05	0.385113059
cg22154659	HOXA1;HOXA1	8.60E-06	0.385099643
cg22711111	TRIM15	0.000148457	0.385091211
cg21821214		0.000117986	0.385091032
cg01298010		0.000290372	0.385085333
cg22646324	ADRA2C	0.00043997	0.3850328
cg02346713	TNFAIP8L3	0.000117986	0.385031642
cg05979020	HOXD10	1.96E-05	0.385021381
cg02981443	TMEM98;TMEM98	8.60E-06	0.385015906
cg01295203	PRDM14	2.58E-05	0.385004971
cg00902847	FGF12	0.000148457	0.384998247
cg25967418	PCDHA7;PCDHA12;PCDHA6;PCDHA10;PCDHA	0.000117986	0.384993768
cg13764991	DMRT2;DMRT2;DMRT2	9.24E-05	0.384990575
cg06966660	TACC2;TACC2;TACC2;TACC2	0.000654035	0.38497688
cg09467508		2.58E-05	0.384952432
cg26124242	GUCY1A2	7.23E-05	0.384944239
cg15085086	OPRM1;OPRM1;OPRM1;OPRM1;OPRM1;OPRI	1.96E-05	0.384896371
cg06496261	PDE11A;PDE11A	0.00043997	0.384873585
cg01978558		7.23E-05	0.384870483
cg26945996	SYT9	2.58E-05	0.384854368
cg24773418		0.00041966	0.384845956
cg10915772	SCIN	2.58E-05	0.384833994
cg05769349	TBX5;TBX5	2.58E-05	0.384831346
cg15633664	GRP;GRP;GRP	2.58E-05	0.384823403
cg14258853	TMTC1	4.34E-05	0.384795644
cg23654127	GPR120	5.59E-05	0.384788101
cg25950112		5.59E-05	0.384779746
cg25563233	RGL3;RGL3	4.34E-05	0.384779232
cg14511782	GPR85;GPR85;GPR85;GPR85	9.24E-05	0.384761719
cg00874990		8.60E-06	0.384755864
cg14996783	TRIL	0.003189589	0.38474012
cg06493664	CALB2;CALB2;CALB2;CALB2;CALB2	5.59E-05	0.384739845
cg23929344	SFTA3	2.58E-05	0.38472979
cg26917140		2.58E-05	0.384697002
cg03464514	PTN;PTN	1.50E-05	0.38469252
cg09300089	ANKRD11	2.58E-05	0.384685811
cg14473327	DMRT3	2.58E-05	0.38468421
cg05027458	ADAMTS17;ADAMTS17	0.00043997	0.384662468
cg05472874	SULT4A1	8.60E-06	0.384651695

cg07893512	HBM	2.58E-05	0.384643563
cg02305242	FLRT2	1.96E-05	0.384634665
cg24494316		0.000537905	0.384619467
cg23333146	EIF4E3;EIF4E3;EIF4E3;EIF4E3	1.96E-05	0.384579055
cg05539515	KANK4	8.60E-06	0.384577395
cg13601496	ADAMTS5	4.34E-05	0.3845648
cg21852439	GRID2	0.000117986	0.384561611
cg26063106	FREM2	1.11E-05	0.384560103
cg26669806	COMP	7.23E-05	0.384514069
cg26738987	GPR83	4.34E-05	0.384511155
cg03964958	HOXD12	0.000148457	0.384508963
cg21516341	NELL2;NELL2;NELL2;NELL2;NELL2	0.000148457	0.384505813
cg16974909	WDR17;WDR17	2.58E-05	0.384504432
cg03774514	FOXA2;FOXA2	0.000537905	0.384485073
cg18591228	OSBPL5;OSBPL5;OSBPL5	1.50E-05	0.384482484
cg16051245	TMPRSS2;TMPRSS2	0.000537905	0.384452063
cg14192957		0.000205664	0.384416489
cg05324789		1.11E-05	0.384408881
cg23948080	VASH1	0.000148457	0.384407097
cg01862172	SAMD5	1.50E-05	0.38440326
cg24495234	CTXN2	3.34E-05	0.384364868
cg01419757	C11orf70	0.000122184	0.384358239
cg07093155	USP7	0.000233422	0.384347421
cg09464194	FBXO27	0.000357885	0.384341555
cg07456114	HELT	0.000233422	0.384319696
cg17554604		0.000262362	0.384304377
cg15986587		1.50E-05	0.384289522
cg18855674		1.50E-05	0.384284357
cg00740020	KCNC3	2.58E-05	0.384265437
cg04865692	KCNC3	1.96E-05	0.384260155
cg26195583		2.58E-05	0.38425858
cg14991487	HOXD9	1.96E-05	0.384253453
cg05790451	APBA2;APBA2	0.000233422	0.384252869
cg06268694	CELSR1	0.002304201	0.384235645
cg15861585	DRD4	7.23E-05	0.384219414
cg24617568	DPY19L2P4	5.59E-05	0.384212019
cg10455940	BTRC;BTRC	0.00043997	0.384208288
cg11012061		5.59E-05	0.38420531
cg03631455	PCDHB13	1.50E-05	0.384197957
cg10437806	C12orf39	0.000148457	0.384178206
cg09166091		0.000148457	0.384159945
cg08036121	SH3BP4	8.60E-06	0.384158088
cg01904063	ISL1	2.58E-05	0.384153255
cg14647451	EPHA5;EPHA5	2.58E-05	0.384131157
cg12299361		1.50E-05	0.384118115
cg04445871		1.11E-05	0.384099072
cg11070069		8.60E-06	0.384095517
cg18585903	RBP4	2.58E-05	0.384078655
cg04484126		0.000233422	0.384068494
cg04640250	LOC283392;TRHDE;LOC283392	5.59E-05	0.384064813
cg03620853	CYP2S1	7.23E-05	0.384044913
cg20813518		0.003734675	0.384035492
cg05746795		1.11E-05	0.384015424
cg19047265		1.96E-05	0.38401311
cg04921989		0.000537905	0.383997755
cg20312012	FER1L5	0.000357885	0.383996608
cg12142521	ACOXL;ACOXL;ACOXL	9.24E-05	0.383993473
cg27016494	DLX5	4.34E-05	0.383984578
cg05172259		8.60E-06	0.38397797
cg27403265	HS3ST2	2.58E-05	0.38396353

cg13144843		0.000791389	0.383954296
cg06493334	LHFPL3	0.000117986	0.383947449
cg05477514	NKX2-6	5.59E-05	0.383931068
cg26842121		1.96E-05	0.383922517
cg08279075	IRX3	0.000791389	0.383880978
cg14613271	SLC5A7	4.34E-05	0.383868946
cg04515001	KAAG1;DCDC2;KAAG1;DCDC2	4.34E-05	0.383867681
cg21286402	SLC22A15	0.000537905	0.383866222
cg11797092	SH3GL3;SH3GL3	1.50E-05	0.383842837
cg10542883		0.000537905	0.383836556
cg20778451	ZNF454	9.24E-05	0.383834497
cg00739120	GAPDHS;TMEM147	8.60E-06	0.383818524
cg18638018		1.96E-05	0.383809956
cg15243856	MATN4;MATN4;RBPJL;MATN4	4.34E-05	0.38380791
cg19194373	EDN3;EDN3;EDN3;EDN3;EDN3;EDN3;EDN3;E	0.000117986	0.383780016
cg19258784	ROBO1;ROBO1;ROBO1;ROBO1	3.34E-05	0.383768623
cg14024461	HIST3H2A;HIST3H2BB	0.000205664	0.383757758
cg04845053	P2RY1;P2RY1	0.001639597	0.383753191
cg05040472		9.24E-05	0.38373671
cg03308092	GAB4	7.23E-05	0.383724382
cg08520743	PON3	0.000233422	0.383718642
cg00142106	HCG4	0.000233422	0.383712462
cg04871472		0.000148457	0.383704514
cg06444282	VWA5B1	0.000290372	0.383691376
cg04237663	KCNV1	0.000117986	0.383685241
cg13702005	C12orf42;C12orf42	0.000955793	0.383677166
cg03072621	A2BP1;A2BP1;A2BP1;A2BP1	0.000233422	0.383671605
cg05446322	MIR548F5;NBEA	3.34E-05	0.38366759
cg05756220	KCNA4	8.60E-06	0.38366624
cg05306070		0.006738759	0.383653006
cg08218734	FAM196A;DOCK1	0.000148457	0.383640436
cg10767192		1.50E-05	0.383628283
cg10080155	PHF21B;PHF21B	0.000148457	0.383594421
cg07883823	SLC22A3	7.23E-05	0.383572156
cg25189564	VIPR2	0.006823935	0.383571626
cg05994320		2.58E-05	0.383548958
cg05293820	STXBP5L	5.59E-05	0.383546632
cg12325536	LOC645323;LOC645323;LOC645323	0.000233422	0.383544544
cg14073571	VIPR2	0.000654035	0.383540958
cg09301294	EPHA6	3.34E-05	0.383540853
cg05259545	LOC440040	7.23E-05	0.383538631
cg14554468		0.000290372	0.383523579
cg07602998	ROR1;ROR1	0.001639597	0.383516366
cg05030680	PITX2;PITX2;PITX2	0.000187194	0.383513448
cg05135569	DBX2	0.000148457	0.383507692
cg00096922	DLX5	0.001948248	0.383507482
cg20413471	ADAMTS19	1.96E-05	0.383505326
cg14225678	VSTM2A	2.58E-05	0.383504295
cg05615464		5.59E-05	0.383487962
cg02438781		1.11E-05	0.383486931
cg07534843	MATN4;MATN4;MATN4;RBPJL	4.34E-05	0.383462769
cg04139641	C9orf170;C9orf170	0.000117986	0.383422652
cg02243157		0.000148457	0.383396891
cg17121178	BSX	8.60E-06	0.383393251
cg15716405	EDIL3	0.000148457	0.383383399
cg13498757		0.002304201	0.383378944
cg11347582		0.00043997	0.383372814
cg17632372		8.60E-06	0.383365503
cg04830146	MYO1D	1.11E-05	0.383363197
cg07167946	IRX4	3.34E-05	0.383346311

cg05295015		1.50E-05	0.383316913
cg13939185	RORB	0.000233422	0.383286956
cg21860429		8.60E-06	0.383283914
cg21799469		1.96E-05	0.38327805
cg27498114	ADRB3	2.58E-05	0.38327671
cg24185576	MPPED2	0.001376172	0.383264605
cg20919680	TMEM198;CHPF;TMEM198	0.000233422	0.383236138
cg01464835	VSTM2B	0.000654035	0.383232847
cg14335275	LYPD6	1.50E-05	0.383224662
cg23462242	CYFIP2;CYFIP2;CYFIP2	0.000791389	0.383221605
cg22316614		1.11E-05	0.38321818
cg07419144		3.34E-05	0.383217318
cg07833420	HTR1E;HTR1E	0.000148457	0.3831588
cg26880493	PDZD2	0.000233422	0.38314897
cg21230493	ADRA1A;ADRA1A;ADRA1A;ADRA1A;ADRA1A;A	2.58E-05	0.383147047
cg07957294	PRRX1;PRRX1	5.59E-05	0.383144077
cg01579912	CDC16;CDC16	0.001376172	0.38313254
cg15986841	ECHDC2	0.000526175	0.383121555
cg26164310	LPPR4;LPPR4	1.96E-05	0.383107011
cg25722465	KDR;KDR	0.000148457	0.383102147
cg08378017		0.000654035	0.383094969
cg09465834	IGSF11;IGSF11	2.58E-05	0.383086655
cg16732654	AATK	0.000117986	0.383056824
cg01556706	FHIT;FHIT	3.34E-05	0.383055837
cg19403534	PRLHR	7.23E-05	0.383047914
cg15418826	KIF21A	0.003734675	0.383042192
cg07297964	RB1	8.60E-06	0.383037364
cg03139435	AURKA;AURKA;AURKA;CSTF1;AURKA;CSTF1	0.000117986	0.383034417
cg08793877		0.000187194	0.382994936
cg16769152	KCTD14	0.000537905	0.382994821
cg03840647	SFTA3	1.50E-05	0.38299442
cg00884093	CELSR1	0.007873393	0.382946693
cg17110006		7.23E-05	0.382946191
cg04012266	PDE3A	0.000233422	0.382941711
cg05986288		1.11E-05	0.382924231
cg05673756	NALCN	4.34E-05	0.382919388
cg19696317	SLITRK1;SLITRK1	7.23E-05	0.382918486
cg23148701	DPY19L2	5.59E-05	0.382917432
cg15033269	SS18L2	5.59E-05	0.382915945
cg23922259		5.59E-05	0.382898761
cg01620379	RNASE11	4.34E-05	0.382879976
cg18403551	SPSB4	0.001639597	0.382853832
cg19609242	OSMR;OSMR	3.34E-05	0.382844696
cg14328641	MS4A3;MS4A3;MS4A3	9.24E-05	0.382843566
cg03526459		0.002266401	0.382835256
cg23137881	C4orf45	0.00043997	0.38283387
cg11657808	RYR2	1.11E-05	0.382816502
cg06798106		4.34E-05	0.38279754
cg13891978	KCNH5;KCNH5;KCNH5	8.60E-06	0.382794515
cg22274539	C12orf42;C12orf42	0.000233422	0.382762567
cg06333058	CLIP4	0.000290372	0.382757259
cg19048251		3.34E-05	0.382748482
cg08602190	ADAM12;ADAM12	1.50E-05	0.382745613
cg20889841		8.60E-06	0.382740769
cg00989492		3.34E-05	0.382734569
cg11642382	CNTNAP2	0.000187194	0.382733976
cg05417127	HS3ST6	0.000357885	0.38272748
cg06510074		8.60E-06	0.382727154
cg04747634	HYDIN;HYDIN	9.24E-05	0.382698272
cg05640128	DPP6;DPP6;DPP6	8.60E-06	0.382692588

cg08447525	GPR149	4.34E-05	0.38269149
cg06667761	PCDH10;PCDH10	8.60E-06	0.382656062
cg07218663	GRM1;GRM1	1.11E-05	0.382650734
cg16968596	ACAN;ACAN	0.000357885	0.382648205
cg25888561		0.001147425	0.382612214
cg09090017		9.24E-05	0.382610728
cg01925738	PCDHB3	4.34E-05	0.382607958
cg23538854	IL7;IL7	2.58E-05	0.382607164
cg24842733	SNAP91;SNAP91	1.96E-05	0.382565844
cg06444755	NXPH1;NXPH1	3.34E-05	0.382561981
cg25863967	TSGA14	3.34E-05	0.382561532
cg00842595	C17orf97	3.34E-05	0.382549382
cg20701486	NPHS2	0.000235312	0.382539599
cg08447739	HTRA1	0.000537905	0.382534153
cg17536603		2.58E-05	0.382531696
cg02694017	CACNG8;MIR935	0.001948248	0.382526592
cg04599965		2.58E-05	0.382522529
cg08837627		3.34E-05	0.38251755
cg24708471		9.24E-05	0.382511845
cg18007959		4.34E-05	0.382501185
cg01439876		0.000537905	0.382496425
cg05201970	HCN1;HCN1	0.00043997	0.382477246
cg17586302	PHACTR2;PHACTR2;PHACTR2;PHACTR2	1.11E-05	0.382477096
cg02199174	TMEM98;TMEM98	8.60E-06	0.382437108
cg19227130	FEZF2	0.000791389	0.382418712
cg24913868	NKX2-3	0.000791389	0.38241628
cg02943578	FOXC2	1.11E-05	0.382412107
cg16317181	PCDH21	0.000205664	0.382410389
cg13671601	OSBP2	2.58E-05	0.382407687
cg19810038	PHACTR3	2.58E-05	0.382403368
cg22629111	FAM78B	8.60E-06	0.382402455
cg17478228	ELAVL3;ELAVL3;ELAVL3;ELAVL3	8.60E-06	0.382399519
cg19906284	TTN;TTN;TTN;MIR548N;TTN	2.58E-05	0.382397178
cg18698884		1.11E-05	0.382392949
cg05004108	DDAH1;DDAH1	2.58E-05	0.382364099
cg01232717	TCEB1	9.24E-05	0.382343729
cg10849854	GREB1	0.00043997	0.382323872
cg03700449	ASCL1	0.000148457	0.382309504
cg06721014	C7orf63;C7orf63	9.24E-05	0.382301899
cg19542707		8.60E-06	0.382294251
cg24678502	ANO7	1.50E-05	0.382285454
cg19612191		3.34E-05	0.382265452
cg07706352	COL5A3	8.60E-06	0.382259786
cg11735997	KCNA4	8.60E-06	0.382244784
cg11722699	DSC3;DSC3	0.000537905	0.382236031
cg16435571	SLC6A11	5.59E-05	0.382234756
cg17767285	SNAP91;SNAP91	0.000290372	0.382219543
cg14672921	GLB1L2	1.50E-05	0.382219143
cg02126788	NHLH2;NHLH2	0.000233422	0.382201377
cg08803263		0.000187194	0.382201174
cg12093775		0.000537905	0.382196998
cg20081969	ARNT2	0.000791389	0.382173741
cg11756095		0.001639597	0.382167707
cg00173659		1.60E-05	0.382161378
cg13552710	ALK	0.000290372	0.382160403
cg18424634	ZIC1	3.34E-05	0.382136782
cg13759674	GRIN1;GRIN1;GRIN1	0.002714607	0.382128848
cg05855917	ZIC4;ZIC4;ZIC4	1.96E-05	0.38210737
cg18430555		1.96E-05	0.382096918
cg07375836	ACACA;ACACA	0.003189589	0.382088446

cg04882273	CDH8	2.39E-05	0.382088104
cg06195987		1.96E-05	0.382069755
cg05681757	FGD4	3.34E-05	0.382068201
cg16434547	SYNE1;SYNE1	0.000223017	0.382068148
cg14854503	CSGALNACT1;CSGALNACT1	0.000233422	0.382046301
cg16009633	PREX2;PREX2	1.96E-05	0.382032531
cg26701826	SGMS2;SGMS2;SGMS2;SGMS2	7.23E-05	0.382029058
cg02193956	MANBA	0.001147425	0.382017672
cg27425314	DHDDS;HMGN2;DHDDS	0.000117986	0.382012155
cg23897913	MYOD1	0.000537905	0.38200757
cg05071334	CA13	1.50E-05	0.382000438
cg02826735		0.000187194	0.381999705
cg06701529		5.11E-05	0.381995256
cg02332525	GRM7;GRM7	8.60E-06	0.381982622
cg23524735	PTH2R	8.60E-06	0.381979498
cg08005774	IGSF21	8.60E-06	0.381976348
cg04326854	PDE10A;PDE10A;PDE10A	1.96E-05	0.381970093
cg19453181	SEMA6D;SEMA6D;SEMA6D;SEMA6D;SEMA6D	0.000117986	0.381965698
cg22652378	RPTOR;RPTOR	1.96E-05	0.38195421
cg18855080		0.000654035	0.381946871
cg07896832	CSPP1;CSPP1;COPS5	0.000955793	0.381929104
cg27664496	DTX1	0.000654035	0.381910183
cg03947814	KCNQ1DN	1.50E-05	0.381903567
cg07147166		1.50E-05	0.381877637
cg04535008	TACR3	1.11E-05	0.381870406
cg26557693		4.34E-05	0.381860359
cg19619956	FAM193B;FAM193B	1.50E-05	0.381829292
cg07227049	VRK2;VRK2;VRK2;VRK2;VRK2;VRK2	1.96E-05	0.381808721
cg06372391	KANK4;KANK4	0.000357885	0.381791843
cg06784991	ZYG11A	5.59E-05	0.381773013
cg00455372	KCNC1;KCNC1	0.001147425	0.381764585
cg00343906	NADK	0.000117986	0.381763433
cg09521703	SHISA7	1.50E-05	0.381745324
cg25968394		5.11E-05	0.381742702
cg10479082	TRIL	0.000955793	0.381732268
cg12176709	DRD2;DRD2	4.34E-05	0.381700331
cg19884262	FOXI2	8.60E-06	0.381680835
cg26419880	ESPN	0.000148457	0.381664105
cg16359550	C11orf87	8.60E-06	0.381659467
cg20175390		2.58E-05	0.381604594
cg12343182	RIMS4	0.000290372	0.381581002
cg09491991	ISM1	0.000654035	0.38157611
cg04002618	PHACTR2;PHACTR2;PHACTR2;PHACTR2;PHA	7.23E-05	0.381567813
cg04441847	SFRS7	0.000117986	0.381562393
cg16643542	AZU1;AZU1	1.96E-05	0.381553942
cg24545961	CLDN10;CLDN10;CLDN10	0.001147425	0.381547823
cg00264591	IRX4	4.34E-05	0.3815426
cg14085358	EVX2	4.34E-05	0.381531256
cg05580141	C12orf41	4.34E-05	0.381519405
cg16848624	ADCY1	0.00043997	0.381502879
cg14586939	NPY2R;NPY2R	1.96E-05	0.381463118
cg23996071	HOOK1	8.60E-06	0.381450109
cg20747266	NMBR	1.11E-05	0.38144377
cg18279326		4.34E-05	0.381406192
cg24071719		0.000955793	0.381404626
cg06581175	DLX6AS	5.59E-05	0.381404511
cg20657674		3.34E-05	0.381397959
cg27051231		1.11E-05	0.381387196
cg10175795	TCERG1L	5.59E-05	0.381373699
cg07365960	GRIN2C	3.34E-05	0.381363836

cg11169641		1.11E-05	0.381346672
cg02809746		0.000290372	0.381337612
cg25976257	PHF21B;PHF21B	4.34E-05	0.3813272
cg24319171	IQSEC3;IQSEC3	0.00041966	0.381326793
cg14717557	SIM1	7.23E-05	0.381321628
cg10363951	CHST9;CHST9	8.60E-06	0.38132098
cg20980055	EYA4;EYA4;EYA4	0.001147425	0.381316215
cg18845377		3.21E-05	0.381313079
cg13462129	DLX5	0.000117986	0.381298998
cg13503413	PHYHIPL;PHYHIPL;PHYHIPL	5.59E-05	0.381293062
cg23187653	PCSK1	3.34E-05	0.381287762
cg20739526	DPP6;DPP6;DPP6	0.001691157	0.381276919
cg16803083		0.000233422	0.381251027
cg16821446	SLITRK5	0.000537905	0.381222492
cg08077337	FZD7	0.000654035	0.381214653
cg10475970	PCDHGA4;PCDHGA6;PCDHGA1;PCDHGA5;PC	8.60E-06	0.381204912
cg09074468	CDK20;CDK20;CDK20;CDK20;CDK20	3.34E-05	0.381160176
cg03319497		4.34E-05	0.381157049
cg03175677	SORCS3	9.24E-05	0.381138463
cg05001964	IRX3	0.001639597	0.381136192
cg26063037	EPHX2	1.96E-05	0.381135939
cg07840120	ARSJ	7.23E-05	0.381134331
cg18268547	TSPAN10	0.000117986	0.381131549
cg24870568	RNF207	0.000233422	0.381110906
cg14254748	L1TD1;L1TD1	5.59E-05	0.381105143
cg04476865	MIR548F5;NBEA	7.23E-05	0.381104262
cg12149911	CA8	3.34E-05	0.381057543
cg01609275		5.59E-05	0.381056795
cg00881585		2.58E-05	0.381029248
cg26855801		2.58E-05	0.381008005
cg23164982		2.58E-05	0.380994353
cg27406373	PAQR9	3.34E-05	0.38099215
cg03833046		0.000654035	0.380986787
cg10180406	PTGS2	1.11E-05	0.380976464
cg12809098	DENND3	0.000233422	0.380960775
cg26567423	FLJ12825	0.000955793	0.380953279
cg02392179	SORCS3	3.34E-05	0.380924934
cg01986123	ARL4A;ARL4A;ARL4A	0.003734675	0.380904368
cg26116950	PDZRN4	0.001147425	0.380899585
cg23368787	ATP4A	8.60E-06	0.380897543
cg01037823	ZG16B	1.96E-05	0.380895946
cg11113760	ZNF385B;MIR1258	0.000290372	0.380877109
cg10453365	RHCG	1.96E-05	0.380876146
cg02132284		7.23E-05	0.380868567
cg16402415	ZNF280D;ZNF280D;ZNF280D	0.001147425	0.380844574
cg00107682		0.000148457	0.380836577
cg11021940	LVRN	9.24E-05	0.380828006
cg19424531	CYP7B1	0.000233422	0.380827724
cg00177388		0.000290372	0.380827696
cg00153934	LCA5;LCA5	0.001376172	0.380819293
cg10168494		0.000233422	0.38080353
cg16732616	DMRTA2	0.000654035	0.380798181
cg02339682	DST;BEND6	0.000654035	0.380745172
cg12143789		0.000187194	0.380744422
cg24534566	CWH43	1.96E-05	0.380741152
cg09820011	KIAA1598;KIAA1598	2.58E-05	0.380737658
cg07832473	RXFP3	0.00043997	0.380725518
cg01580228	MIR1304;SNORA8;SNORD5;SNORA18	0.00043997	0.380714031
cg02744524		1.96E-05	0.380713208
cg14329860	NRXN1;NRXN1	5.59E-05	0.380706317

cg27304406	BNC1	2.58E-05	0.380697646
cg25400229	GNAL;GNAL;GNAL	0.000233422	0.380696407
cg09275956		0.000117986	0.38069292
cg22719623	OPRM1;OPRM1;OPRM1;OPRM1;OPRM1;OPRI	1.96E-05	0.380690035
cg23815900		8.60E-06	0.380686303
cg20266316	NPTX2	0.000148457	0.380685726
cg08393070	HBM	5.59E-05	0.380664487
cg11312408	PCDH17	1.50E-05	0.380659112
cg05633391	KCNF1	0.002714607	0.380658238
cg08757348	ZNF214;NLRP14;ZNF214	1.50E-05	0.380643094
cg25272655	HIST3H2BB	8.60E-06	0.380636424
cg05038216	CLIP4	0.000955793	0.380630226
cg08526074	SALL1;SALL1	0.001691157	0.380615753
cg07182756	MOCS1;MOCS1	0.000187194	0.380612395
cg05480594		0.004131428	0.380603545
cg15268191		4.34E-05	0.380600956
cg05339390	ARL4A;ARL4A;ARL4A	0.003189589	0.38059541
cg16094412	RNF207	0.000537905	0.380587102
cg13178361	UAP1	5.59E-05	0.380586598
cg17332338		0.00041966	0.380586546
cg26365925		0.000357885	0.380545467
cg17580935	MAGI1;MAGI1;MAGI1	1.50E-05	0.380533064
cg02211449		0.000791389	0.380511927
cg21264227		2.58E-05	0.380504539
cg04811512	PAX1	0.000148457	0.380467589
cg14266237	KLF1	1.11E-05	0.380466888
cg10000952		7.23E-05	0.380457113
cg05401965	PCDH10;PCDH10	0.000187194	0.380400158
cg07862488	SIX6	1.50E-05	0.380383387
cg10933959	FLJ42289;FLJ42289	5.59E-05	0.380375222
cg19854521	TFPI2	0.000537905	0.380373876
cg27182172	PPARGC1A	1.96E-05	0.380371104
cg05052969		1.96E-05	0.380360707
cg08399444	GSG1;GSG1;GSG1;GSG1;GSG1;GSG1	1.96E-05	0.380344066
cg05301470		0.000357885	0.380341458
cg17238065	CAPN1	5.59E-05	0.380336988
cg22555675	UNC13A	0.000117986	0.380310403
cg07481273	C14orf109;C14orf109	0.001639597	0.380299414
cg07482935	RELN;RELN	1.50E-05	0.380295465
cg00058449	ZBTB12	0.000187194	0.380276206
cg05157140	UNCX	0.000290372	0.380259545
cg08448812	PCDHA6;PCDHA2;PCDHA1;PCDHA1;PCDHA6	1.50E-05	0.380255356
cg16966315		0.000290372	0.380254444
cg09510559	NPHS1	8.60E-06	0.380227231
cg11236526		0.000654035	0.380199744
cg20312087	MGC70857	0.001147425	0.380183751
cg12091642		9.24E-05	0.380172716
cg18421949	MAP7	8.60E-06	0.380170157
cg08946575	DDAH1;DDAH1	5.59E-05	0.380158996
cg22662844	MAD1L1;MAD1L1;MAD1L1	1.96E-05	0.380155209
cg05207495	KCNH5;KCNH5;KCNH5	1.11E-05	0.380152514
cg22108374	CCDC33	7.23E-05	0.380133903
cg12969193	HOXD9	4.34E-05	0.380116209
cg21901153	TEKT3;TEKT3	5.59E-05	0.380089645
cg24419094	RRM2;RRM2	0.00043997	0.380082872
cg07699311	C1orf115	0.004357816	0.380080287
cg18575811	KCNK5	0.001381372	0.380054657
cg07027430	A2BP1;A2BP1;A2BP1	3.34E-05	0.380049851
cg07017477	THEM4	5.59E-05	0.380031518
cg00366818	ARMC4	0.001639597	0.380011495

cg25484904	CWH43	8.60E-06	0.379995853
cg09418283	PAWR;PAWR	0.000654035	0.379983695
cg16581347		9.24E-05	0.379983071
cg16635352	SLC6A5	0.000117986	0.379978675
cg25469714	CUBN	0.000117986	0.379960028
cg10893986	PRDM16;PRDM16	0.000148457	0.379950204
cg11154747	IL7;IL7	1.96E-05	0.379936681
cg16747109	PITX2;PITX2;PITX2	0.000148457	0.379933588
cg18390025	ELOVL3	0.000187194	0.379919159
cg14964336		0.000654035	0.379917979
cg22836400	TFAP2A;TFAP2A	0.000233422	0.379910768
cg02165099	FLRT2	2.58E-05	0.379908204
cg08550421	PCDHA7;PCDHAC1;PCDHA12;PCDHA6;PCDH	8.60E-06	0.379901473
cg14314674		1.11E-05	0.379896738
cg18565783		0.000357885	0.379895788
cg00721530		1.96E-05	0.379877438
cg02650401	SOX2OT	3.34E-05	0.379866396
cg15808943	HOXD8;HOXD8	0.001376172	0.379860555
cg08065657	HAS1	8.60E-06	0.379858821
cg09072162	PCSK9	0.000290372	0.379799864
cg27515369	SPSB4	0.000233422	0.379798726
cg20804191	GBX2	0.000233422	0.379783114
cg21406144		2.58E-05	0.379780757
cg10811045		0.002304201	0.379769419
cg08239858		0.000654035	0.379733453
cg01622437		1.96E-05	0.37969066
cg11814087	ZFR	8.60E-06	0.379644479
cg01434160		1.96E-05	0.379642979
cg08513253	SOBP	2.58E-05	0.379640668
cg23026554	LOC441666	1.11E-05	0.379640627
cg10838410	IFFO1;IFFO1;IFFO1	0.001147425	0.379637004
cg17707487	TFDP1;TFDP1	3.34E-05	0.379631954
cg08013557	ZIC4;ZIC4;ZIC4	2.58E-05	0.379625469
cg19156875	MAPT;MAPT;MAPT;MAPT;LOC100130148;MAP	2.58E-05	0.379603174
cg22898797		0.000791389	0.379598821
cg13920367	CDH1	8.60E-06	0.379565579
cg17240815	HCRTR2;HCRTR2	0.000187194	0.379539281
cg20115218	ANO5;ANO5;ANO5;ANO5	1.11E-05	0.379529305
cg24150172	SOX17	0.000357885	0.379501528
cg15919816		2.58E-05	0.379489256
cg19875547	AMPH;AMPH	0.000187194	0.379476975
cg11899535	WBSCR17	0.000187194	0.37947658
cg14618638	GALNT11	0.000537905	0.379473474
cg08668551		0.000117986	0.379458698
cg05571970		1.50E-05	0.379434419
cg02539083	TAC1;TAC1;TAC1;TAC1	1.11E-05	0.379427435
cg23625660	FBXL21	8.60E-06	0.379418139
cg07650252	KCTD8	5.59E-05	0.379416459
cg25044651	LVRN	1.50E-05	0.379406059
cg08855395		0.000187194	0.379402732
cg27255275	NFRKB	2.58E-05	0.37939059
cg24755910	C7orf63;C7orf63	7.23E-05	0.379384369
cg06674731	RPRM	0.000117986	0.379355853
cg19152508	MKX	1.50E-05	0.379343535
cg04859706	PCDH7;PCDH7;PCDH7	0.000357885	0.379328554
cg02140579	FAM198B;FAM198B;FAM198B	5.59E-05	0.379244628
cg00977827		0.001147425	0.379198824
cg07645844	ADRA1A;ADRA1A;ADRA1A;ADRA1A;ADRA1A;	1.50E-05	0.379195497
cg12678686	MCM2	0.000654035	0.379186086
cg20692998		4.34E-05	0.379174859

cg11769332	DLX4;DLX4	8.60E-06	0.379164054
cg12398124	GALNT13	3.34E-05	0.379164049
cg10020520	SETD1A	3.34E-05	0.379131658
cg02879423	WDFY1	7.23E-05	0.379127437
cg26789779	GDNF	0.00043997	0.379124814
cg04316624	HOXD3	3.34E-05	0.379123209
cg18672939	GFRA1;GFRA1;GFRA1;GFRA1	0.000791389	0.379122607
cg11026333	LRRC61;ACTR3C;LRRC61	0.000526175	0.379102169
cg24049616	IRX6	3.34E-05	0.379099282
cg22368262	C10orf35	0.000791389	0.379053731
cg04606861	GALNT9	0.000148457	0.379021412
cg03940966	THEM4	3.34E-05	0.379016256
cg21347353		3.34E-05	0.379015567
cg02505827	MARVELD2;MARVELD2	1.96E-05	0.379000559
cg19703610	DSCAML1	1.96E-05	0.378959923
cg14169158	FAM78B	5.59E-05	0.378944149
cg06951626		1.11E-05	0.378915278
cg25964728	TMEM22;TMEM22;TMEM22	0.000537905	0.378912841
cg13099890	LOC283392;LOC283392;TRHDE	9.24E-05	0.378910008
cg05065507	TACSTD2;TACSTD2	0.000148457	0.378874899
cg16182148	TMEM101	7.23E-05	0.378870432
cg17891759		2.58E-05	0.378868684
cg24939838	PRDM16;PRDM16	3.34E-05	0.378858475
cg13845982	GATA5	0.000955793	0.378844956
cg09644707		2.58E-05	0.378835492
cg07963234	CRMP1	0.000233422	0.378827813
cg12838192	C4orf31;C4orf31	1.11E-05	0.378809372
cg06494592		0.000955793	0.378799147
cg09220326	CDCP1;CDCP1	2.58E-05	0.378798833
cg07924892	MOCOS	3.34E-05	0.378798615
cg01938018	NPHS2	3.34E-05	0.378764251
cg00359365	MIR1976;RPS6KA1;RPS6KA1	0.001948248	0.378757959
cg06076277		8.60E-06	0.378752729
cg02179499	FGFR2;FGFR2;FGFR2;FGFR2;FGFR2	2.58E-05	0.378744475
cg03545227	PTPRN	1.11E-05	0.378741413
cg16145703	XKR6	0.001147425	0.378715304
cg05872073	PARD6G	0.00043997	0.37871125
cg25496181	KSR2	0.000791389	0.378699277
cg26023087		2.58E-05	0.378685293
cg05634149		0.000233422	0.378683059
cg27361914	KLK13	2.58E-05	0.378672303
cg25151919	ZNF502;ZNF502;ZNF502;ZNF502	0.000148457	0.37864216
cg00002810	DAB1	9.24E-05	0.37863745
cg25249539		0.000290372	0.378621844
cg03969305		2.58E-05	0.378611184
cg21683708		0.001147425	0.378556304
cg02533787	LYPD6B;LYPD6B	0.000148457	0.378546776
cg21646199	NHLH2;NHLH2	3.34E-05	0.378543206
cg20405893	CHAT;CHAT;CHAT;CHAT;CHAT;CHAT;CHAT;C	2.08E-05	0.378523928
cg16325502	CCDC140	1.50E-05	0.378523778
cg04677123		0.000233422	0.378522824
cg02664109		0.002304201	0.378516485
cg06917763		2.58E-05	0.378496884
cg03843000	TBX5;TBX5	1.96E-05	0.378478911
cg11667754	CDH1	4.34E-05	0.378472831
cg10863741	TMEM155;TMEM155;LOC100192379	0.000233422	0.378456217
cg08529049	CWH43	1.50E-05	0.378436587
cg03599078		0.000290372	0.37842707
cg08891110		2.58E-05	0.378416676
cg15647515	WNT2;WNT2	3.34E-05	0.378406712

cg26021627	FAM150A	1.50E-05	0.378405095
cg25637722	CRB3;CRB3	5.59E-05	0.378389518
cg03440125	TPBG;TPBG	0.000233422	0.378388697
cg14646075	WDR69	1.50E-05	0.378388393
cg02979001		0.00041966	0.378384625
cg20504791	FOXA2;FOXA2	0.001147425	0.378384538
cg19579167	EPB41L3	0.000148457	0.378373179
cg17408527	ZSCAN18;ZSCAN18	8.60E-06	0.378340352
cg16234557	EMX2OS	7.23E-05	0.378324027
cg15341350	SRRM4	9.24E-05	0.378322809
cg14059768		0.000148457	0.378303382
cg22957381	KCND3;KCND3	0.000233422	0.378263323
cg16729160	KCNB2	5.59E-05	0.378262977
cg26082257		2.58E-05	0.378253547
cg20978694	NAT8L	0.000148457	0.378249112
cg15624813		0.000791389	0.378231112
cg17303756		5.59E-05	0.378230525
cg13777717	CRHBP	0.000233422	0.378226072
cg21696012	HMGCL;HMGCL	0.000117986	0.378224909
cg18116154	NMNAT3	0.000654035	0.378220411
cg19780993		7.23E-05	0.37821515
cg09881545		0.000187194	0.378211362
cg11804724	DNAH14;DNAH14;DNAH14	1.96E-05	0.378197522
cg03325733	RAB32	0.000233422	0.378194088
cg14342850		0.000233422	0.378186291
cg17456678		3.34E-05	0.378181843
cg25352836	TSSK6;TSSK6	3.34E-05	0.378159367
cg09382096	PAX6;PAX6;PAX6	7.23E-05	0.378156728
cg13902210	KCNC4;KCNC4;KCNC4	7.23E-05	0.378151193
cg17071446		3.34E-05	0.378145211
cg07409471	TBCD	2.58E-05	0.378125181
cg12999636		0.000187194	0.378111296
cg09152582		8.60E-06	0.378087598
cg02435083		0.000233422	0.378077373
cg10325478	KCNK10;KCNK10	9.24E-05	0.37805543
cg20171396		1.96E-05	0.37804798
cg19229182		1.11E-05	0.378040941
cg09497644	WDR72	1.96E-05	0.378037217
cg16163847	WDFY2	0.000187194	0.378036841
cg18173058	TBX5;TBX5;TBX5;TBX5	0.000148457	0.377976034
cg05804863	KIAA1024	0.000117986	0.377948514
cg10858945		5.59E-05	0.377934554
cg07319315	MGAT5	7.23E-05	0.377934432
cg15410418	PDE4D	0.000117986	0.377910069
cg18044383		0.004357816	0.377906445
cg23679471		1.11E-05	0.37790325
cg10528576	DLK1	3.34E-05	0.377897091
cg12468478	TCF21;TCF21;TCF21;TCF21	0.000117986	0.377881579
cg01775414	PHF21B;PHF21B	2.58E-05	0.377874755
cg11723801	NES	0.000148457	0.377869667
cg10189695	GPR78;GPR78	1.50E-05	0.377868668
cg15044453	MTHFD1L	9.24E-05	0.377861939
cg14421860	GPR88	0.002714607	0.377856907
cg09550809	BHMT	2.58E-05	0.377850231
cg02248826	TJP1;TJP1	8.60E-06	0.37784241
cg11855325	SCRN1;SCRN1;SCRN1;SCRN1;SCRN1	9.24E-05	0.377837762
cg23687194	ID4	0.000187194	0.377825736
cg10541755	EIF5A2	5.59E-05	0.377804073
cg24738611	WDR43;SNORD53	1.50E-05	0.377785885
cg14026459	ELAVL2;ELAVL2;ELAVL2	1.96E-05	0.377771962

cg15479068	SYN3	1.96E-05	0.377762387
cg01140006	GPR97	2.08E-05	0.377761148
cg25437385	SLC35F3	1.96E-05	0.377749402
cg09763175	PRKAA2	0.000654035	0.377740392
cg11557382		9.24E-05	0.377736422
cg14270857	HPSE2;HPSE2;HPSE2;HPSE2	9.24E-05	0.377725456
cg12929755	CALCR;CALCR	4.34E-05	0.377705307
cg20910102	CA8	0.000791389	0.377696043
cg24792289		7.23E-05	0.377693979
cg17144108	GIPR	3.34E-05	0.377693282
cg21282630	CYYR1	0.000233422	0.377689675
cg04858164	TCF12;TCF12;TCF12;TCF12	7.23E-05	0.377683273
cg18268988	TIMM8B;SDHD;TIMM8B	1.11E-05	0.37767941
cg19389953		8.60E-06	0.377669963
cg03352287	CRCT1	0.000357885	0.377667142
cg06792417		1.11E-05	0.377663137
cg23083277	COL29A1;COL29A1	7.23E-05	0.377660433
cg17543296	FAM43B	0.000117986	0.377659254
cg23170439		8.60E-06	0.37765385
cg23686403	PARD6G	0.00043997	0.37762715
cg05448721		4.34E-05	0.377626744
cg07930539		0.000233422	0.377616109
cg00929635	DBNDD2;DBNDD2;DBNDD2;DBNDD2;SYS1-DE	0.000233422	0.377610876
cg00893493	MCOLN3	4.01E-05	0.37755795
cg15939347	TSNARE1	0.002304201	0.377547034
cg12387700	MCOLN3;MCOLN3	0.000148457	0.377539404
cg24446586	HOXA11AS;HOXA11	0.000357885	0.377539097
cg00581731	NPAS2	0.000290372	0.377537378
cg00214791		7.23E-05	0.377515269
cg02146001	GATA5	0.000148457	0.377509229
cg18252309	DPP6	1.11E-05	0.377502007
cg20467957		8.60E-06	0.377481028
cg12798157	AKR7L;AKR7L	0.000233422	0.377477602
cg02843349		1.96E-05	0.377460929
cg04756491	RBM23;RBM23;RBM23	0.00043997	0.377446837
cg13134916	IFLTD1;IFLTD1;IFLTD1;IFLTD1;IFLTD1	0.001639597	0.377438721
cg07033722	PPT1;PPT1	0.000357885	0.37739389
cg12063992	PTPN9	0.001948248	0.377390336
cg20864608		0.000290372	0.377388241
cg27308021	C16orf73;C16orf73	1.96E-05	0.377380462
cg06133110	SCRN1;SCRN1;SCRN1;SCRN1	5.59E-05	0.377375243
cg22121557		0.000233422	0.377362676
cg08211028		0.00043997	0.377356531
cg19730691	TBX15	3.34E-05	0.377351556
cg06966839	MSI2;MSI2	2.58E-05	0.377345165
cg17566735	PLD1;PLD1	0.001147425	0.377340284
cg01701649	N4BP1	8.60E-06	0.377333465
cg00745606		4.34E-05	0.377330375
cg22162281	ZDBF2	0.000117986	0.377324932
cg21091227		8.60E-06	0.377323367
cg26357587	POU2F3	0.000791389	0.377299243
cg25356504	SARM1	0.009066563	0.377290514
cg19054524	PAX1	7.23E-05	0.377288973
cg03087912		0.000791389	0.377264711
cg10349436	FHL2;FHL2;FHL2;FHL2	1.50E-05	0.377261036
cg22052659	CSMD3;CSMD3	0.000654035	0.377229968
cg03338754	F7;F7	2.58E-05	0.377205046
cg01571735	SP5	0.000357885	0.377204968
cg10769146		4.34E-05	0.377175127
cg04919592	IQCE;IQCE	1.50E-05	0.377175122

cg23568192		0.002304201	0.37717387
cg06575035	PCDHGA4;PCDHGA2;PCDHGA5;PCDHGB2;PC	8.60E-06	0.377154894
cg00741624	KIAA1409	0.000187194	0.377153676
cg13500720	AKAP12;AKAP12	0.000233422	0.377149347
cg04915300	GPD2;GPD2	3.21E-05	0.377130962
cg17932911	ERLIN1;ERLIN1	2.58E-05	0.377112091
cg17339147	WNT2;WNT2	8.60E-06	0.377101423
cg18440897	GABRA2;GABRA2	1.96E-05	0.377023651
cg11667258		0.000290372	0.377016992
cg13469851	PLAA	4.34E-05	0.376991608
cg17705041	PCGF3	1.11E-05	0.376965896
cg21167269		1.50E-05	0.376961383
cg04790084	TMEM132E;C17orf102	0.00043997	0.37693239
cg05965863		8.60E-06	0.376923385
cg09591286	ZNF804B	0.000654035	0.376918986
cg07339783		3.34E-05	0.376895861
cg19803052	TMEM196	4.34E-05	0.376887011
cg07935784		1.11E-05	0.376859285
cg02135245	ABR;ABR;ABR	1.96E-05	0.376859209
cg19897071	UGT3A1;UGT3A1	4.34E-05	0.376846621
cg18450420	FAM107B	4.34E-05	0.376839224
cg25340098		9.24E-05	0.376833187
cg12806613		5.59E-05	0.376827664
cg00376544	FBXO39	0.001147425	0.376813787
cg21189727	LRRC67	0.000290372	0.376796461
cg20296343	ZNF876P	1.50E-05	0.376795956
cg08231189	MKX	1.50E-05	0.376789275
cg01923724		1.50E-05	0.376789035
cg09481404		9.24E-05	0.376769539
cg07447922	EPHA10;EPHA10	5.59E-05	0.376733252
cg08739188	SV2C	1.11E-05	0.376733225
cg17777676	PCDHA2;PCDHA1;PCDHA1;PCDHA4;PCDHA3	2.58E-05	0.376712259
cg01450705	CPLX2;CPLX2	7.23E-05	0.376691389
cg15613048	KIF17;KIF17	9.24E-05	0.376677987
cg24632241	CTNNA2;LRRTM1;CTNNA2	0.000187194	0.376677795
cg20056634	MEIS1	0.000233422	0.376657871
cg23599224		1.11E-05	0.376647996
cg09238992		8.60E-06	0.3766383
cg05475524	ACTN3	0.000357885	0.37661398
cg07661818	CLDN1	0.000791389	0.376590152
cg01301637	WNT7A	0.000148457	0.376557437
cg26532358	SLC35F1;SLC35F1	1.11E-05	0.376542753
cg17871749	POU4F3;POU4F3	8.60E-06	0.376523004
cg19684783		0.000290372	0.37652066
cg21829923	SP5	4.34E-05	0.376514348
cg11151395	MPO	9.24E-05	0.376496038
cg20918128	ART5;ART5	0.000117986	0.376480547
cg10509254	NBLA00301;HAND2	8.60E-06	0.376477139
cg14562158	TNC	1.96E-05	0.376475957
cg21353911		1.96E-05	0.37646393
cg23206209	ONECUT1	1.11E-05	0.376462704
cg03133735		0.000654035	0.376415863
cg16132337		0.000117986	0.376412604
cg21882477	STK32A;STK32A	8.60E-06	0.376406364
cg22489153		8.60E-06	0.376390844
cg05563672	TMPRSS2;TMPRSS2;TMPRSS2	0.000955793	0.376382545
cg19523085	ADAMTS5	5.59E-05	0.376379761
cg14619949	F2RL1	0.0050758	0.376378556
cg21461649	CFTR	2.58E-05	0.376367829
cg04835297	TRPC1	0.000148457	0.376365923

cg01871214		0.000117986	0.376359674
cg26071526	TACC2;TACC2;TACC2;TACC2;TACC2;TACC2	5.59E-05	0.376327547
cg06580033	ZNF830;CCT6B	1.50E-05	0.376303513
cg17745088		0.00043997	0.376284003
cg02111748	PLEKHH2;LOC728819	0.000654035	0.37627088
cg06158650	TBX15	8.60E-06	0.376259356
cg09424759	SLC35F3;SLC35F3	1.11E-05	0.376257011
cg06365303		0.000117986	0.376252767
cg01471196	ZNF334;ZNF334;ZNF334;ZNF334	1.96E-05	0.376215765
cg09258813	ADRB3	0.000955793	0.376191622
cg18998138	GULP1	1.11E-05	0.376191557
cg08164556	CACNG4	5.59E-05	0.376169845
cg05919561	TTLL7	0.001948248	0.376161354
cg24818200	PCDHB4	1.50E-05	0.376149446
cg05628549	PRKCDBP	9.24E-05	0.37608641
cg01396176		8.60E-06	0.376081578
cg01076272		2.58E-05	0.376062141
cg11871421	FBXO39	5.59E-05	0.376057713
cg09254939	KLK10;KLK10;KLK10	4.34E-05	0.376044827
cg05803237	MTNR1B	1.11E-05	0.376041372
cg04595562		1.50E-05	0.376027968
cg24107021	DZIP1;DZIP1	1.50E-05	0.376019492
cg00134776	CLSTN2	2.58E-05	0.376017281
cg18624900	SLC16A12	0.000187194	0.376004939
cg06226283	RELN;RELN	8.60E-06	0.375999559
cg03748503		0.000117986	0.375989517
cg09701702		0.000187194	0.375974537
cg19356022	SHC1;SHC1;SHC1;SHC1	0.000187194	0.375968333
cg25814649	EFNB2	0.000290372	0.375947185
cg02388865	LIN28;LIN28	3.34E-05	0.375942115
cg18634506	BUD31;PDAP1	9.24E-05	0.375907871
cg23840223	NMU	0.000148457	0.375894874
cg00889398		8.60E-06	0.375887691
cg04761653	FAM46A	4.34E-05	0.375861288
cg27579609	HTR6	8.60E-06	0.375857223
cg22558291	LOC646405	0.000290372	0.375829296
cg05214685		2.58E-05	0.375820754
cg13652008	LTBP1;LTBP1;LTBP1;LTBP1;LTBP1	2.58E-05	0.375818758
cg02392124	WAC;WAC;WAC	7.23E-05	0.375810869
cg11044575		8.60E-06	0.375809431
cg02283366		0.000334462	0.375808381
cg26590003		0.00043997	0.375803833
cg11439596		0.002304201	0.375791743
cg17790605	KCTD8	0.002304201	0.375753121
cg16745596	SYCN	0.000148457	0.375741319
cg18596362	CBLN2	0.000290372	0.375717886
cg00060320	KY	5.59E-05	0.375717279
cg02365514	MAPK15;MAPK15	2.58E-05	0.375681069
cg13460556	ABHD2;ABHD2	4.34E-05	0.375673612
cg04851268	GHSR;GHSR	0.00043997	0.375670343
cg03414569	EHHADH;EHHADH;EHHADH	0.001376172	0.375666711
cg01960016	MSX2	8.60E-06	0.375656544
cg06145336	HOXD3	4.34E-05	0.375648227
cg01572513		0.000791389	0.375631428
cg18004847	SBNO2	3.34E-05	0.375619545
cg08414087		9.24E-05	0.375618927
cg04271218	C8orf85	8.60E-06	0.37560824
cg10035294	PAX3;PAX3;PAX3;PAX3;PAX3;CCDC140;PAX3	3.34E-05	0.375597708
cg13344169	SSTR2;SSTR2	1.96E-05	0.375591242
cg13885357	KRT3	0.003189589	0.375575965

cg12636435		4.34E-05	0.375575244
cg07110356	MPO	0.000357885	0.375565446
cg05706117	EGFLAM	4.34E-05	0.375558017
cg01450642	RGS20;RGS20;RGS20	0.000117986	0.375541212
cg03318469	LRRC47	0.000148457	0.375540098
cg15722404	PPARG;PPARG;PPARG	0.000290372	0.375527518
cg12058064	KLHL25;MIR1276	1.96E-05	0.375526514
cg22371492	PRRX1;PRRX1	9.24E-05	0.375524982
cg21348997	ARNT2;ARNT2	0.000537905	0.375511237
cg11654179	DTX3	0.000148457	0.375507461
cg14558431	C9orf170	0.000955793	0.375506616
cg18121355	GDNF	0.000537905	0.375503004
cg14907769		0.000187194	0.375492773
cg07557560	CXCL14;CXCL14	5.59E-05	0.37548793
cg01796438	ATG7;ATG7;ATG7	7.23E-05	0.375485036
cg25644243	WDR72	0.000233422	0.375425393
cg24748769	OTX2;OTX2	1.96E-05	0.375409769
cg09303701	WDR35;WDR35	0.000148457	0.375409285
cg15168727		2.58E-05	0.375401983
cg12396325	RIMS2;RIMS2	0.000148457	0.375372586
cg08721908	ANKRD20B	1.11E-05	0.37536311
cg26279070		1.96E-05	0.375326934
cg03896836	KIF1A	1.50E-05	0.375315996
cg22799499	COL9A2	1.96E-05	0.375249502
cg19389884		0.000148457	0.375248169
cg20599967		2.58E-05	0.375237284
cg06838985	TRPC6	8.60E-06	0.375234614
cg04573550	SSTR1	0.000148457	0.375217683
cg23528705	UNCX	4.34E-05	0.375215082
cg14742445	ASCC2	0.000290372	0.375196983
cg15409931	WTIP	0.000233422	0.375191877
cg08235161		0.000654035	0.375186339
cg00280177		0.000537905	0.375183725
cg12377139	SPAG6;SPAG6	7.23E-05	0.375176063
cg16051954	CABC1	0.000866489	0.375172976
cg24975222	CDCP1;CDCP1	0.000187194	0.375140276
cg05696969	SH3RF2	7.23E-05	0.375137703
cg13767940	MIR34B;BTG4;MIR34C	0.000537905	0.375134507
cg26688582		0.000148457	0.375132468
cg15191648	SALL3	1.50E-05	0.375131126
cg17293161		0.000148457	0.375128575
cg01962676	IGSF21	2.58E-05	0.375103558
cg24319381	CPEB1	9.24E-05	0.375099043
cg06489037		4.34E-05	0.37508401
cg08534016	SPEF2	3.34E-05	0.375063167
cg16396191	KIAA0564	0.000233422	0.375034551
cg23685712	CYP24A1;CYP24A1	5.59E-05	0.375032028
cg17279359	USH1C;USH1C	3.34E-05	0.375012939
cg26705960	PGR	0.000365373	0.375010387
cg00703513		1.96E-05	0.375001173
cg19310908	HIF3A	0.00043997	0.375000219
cg24054190	FGF12	3.34E-05	0.374994217
cg04427498		8.60E-06	0.374981108
cg20008332	SOX11	0.001948248	0.374979541
cg18358894		4.34E-05	0.374977204
cg08377331	GNA12	0.000187194	0.374963688
cg12658947	OPCML	0.000233422	0.374952822
cg11638181	MIR129-2	9.24E-05	0.374946182
cg02071243		2.58E-05	0.37494321
cg03858673		4.34E-05	0.374919123

cg27622506	THRB;THRB;THRB	0.000791389	0.37491683
cg14319235	ELTD1	0.000117986	0.374914375
cg09712683	MAL;MAL;MAL;MAL	1.11E-05	0.374900784
cg11849281		8.60E-06	0.374897129
cg19725903	PLBD1	2.58E-05	0.374886755
cg01568998	PREX2;PREX2	2.58E-05	0.374858236
cg21657087		4.34E-05	0.374856604
cg09273112	VPS24;VPS24	1.50E-05	0.374840769
cg25493589	CCK	8.60E-06	0.374811949
cg27436995	FBXL16	0.0050758	0.374805857
cg22041640	PCDHGA4;PCDHGA6;PCDHGA1;PCDHGA5;PC	5.59E-05	0.374803898
cg16925499	HSPA12A	9.24E-05	0.374800178
cg10725937	TREM2	0.000290372	0.37479509
cg26770907		2.58E-05	0.374786544
cg03722052	TPBG;TPBG	0.000290372	0.374775295
cg17406148	RUNDC3B;RUNDC3B;ABCB1;RUNDC3B	4.34E-05	0.374761553
cg23193365	EPHA5;EPHA5	9.24E-05	0.374737363
cg08323651	CHRM2;CHRM2;CHRM2;CHRM2;CHRM2;CHRI	4.34E-05	0.374724071
cg03536474	HOXC11	0.002714607	0.374704669
cg10068300	GNAL;GNAL;GNAL;GNAL	2.58E-05	0.374702103
cg17044159		2.58E-05	0.37468703
cg19802865	FOXA1	0.001948248	0.374685561
cg20509513	TACC2;TACC2;TACC2;TACC2	8.60E-06	0.374649339
cg18477204	C14orf162	7.23E-05	0.374633135
cg05376228	SBNO2;SBNO2	0.000148457	0.37462689
cg13900773	PIWIL1	0.002714607	0.374623296
cg02237629		9.24E-05	0.374620796
cg13011109	FBLL1	3.34E-05	0.374617989
cg02289992	FAM164A;FAM164A	0.000262362	0.374615491
cg11429969	PCDHA2;PCDHA1;PCDHA3;PCDHA3;PCDHA1	8.60E-06	0.374607595
cg11243196	ADAMTS3	0.000654035	0.374594777
cg01449136		8.60E-06	0.374591669
cg20464804	SYN3	2.58E-05	0.374588644
cg05398700	WDR20	3.34E-05	0.374532715
cg00295794		0.000187194	0.374529647
cg15482122	C14orf23;C14orf23	0.000357885	0.374525095
cg00861497		4.34E-05	0.374522642
cg10404336	LYNX1;LYNX1;LYNX1;LYNX1	5.59E-05	0.374491402
cg19396601	GPR123	3.34E-05	0.374490064
cg15527515	SLC7A8	0.00043997	0.374489067
cg09142313		0.001376172	0.374482482
cg17071639	FEZ1;FEZ1	0.000233422	0.374460884
cg16079958	MAP1B	0.000537905	0.374451064
cg10501704	PRKD1	3.34E-05	0.374440432
cg12136608	LRRN1	0.000187194	0.374439416
cg04407248	PRSS8	1.11E-05	0.374430685
cg23188704		3.34E-05	0.374401742
cg00011346	ERBB4;ERBB4	9.24E-05	0.374396178
cg05574357		8.60E-06	0.374385714
cg14603040		3.34E-05	0.374347692
cg06638795	KCNG3;KCNG3	9.24E-05	0.374338413
cg23697417	RSPO4;RSPO4	5.59E-05	0.374301978
cg19879906		1.11E-05	0.374289046
cg13435649		8.60E-06	0.374279568
cg07468327		3.34E-05	0.374278373
cg18867480	BDNF	5.59E-05	0.374264917
cg09465698	PCDHGA4;PCDHGA6;PCDHGB6;PCDHGA9;PC	7.23E-05	0.374255897
cg04910690	CD164L2	0.000357885	0.374236178
cg18689332	TBX5;TBX5;TBX5;TBX5	2.58E-05	0.374234026
cg11082362	INSM2	0.000955793	0.374232741

cg02021919	CCNH	0.000537905	0.374212778
cg07598549	DLX6AS;DLX6	0.001639597	0.374206356
cg09347413		0.000791389	0.374201004
cg23910786	MYO5B;MYO5B	9.24E-05	0.374168383
cg09699039	SLC35F1	8.60E-06	0.374147933
cg00998387		1.11E-05	0.374145997
cg18016194	RALYL;RALYL;RALYL;RALYL;RALYL	0.000117986	0.374142157
cg21117668	BOK;BOK	4.34E-05	0.374120494
cg24653263	EGFLAM	5.59E-05	0.374115073
cg26631984		5.59E-05	0.374103532
cg10947146	XKR6	0.000233422	0.374099499
cg02401556		8.60E-06	0.374094116
cg09949775	COMP;COMP	5.59E-05	0.374034079
cg25397054	GNA12	5.59E-05	0.37403019
cg05638174		1.50E-05	0.374025378
cg18307783		4.34E-05	0.374023135
cg15229124	RBP1;RBP1;RBP1	0.00043997	0.374022272
cg05847960		0.000233422	0.374012931
cg06293787	MTNR1A	1.50E-05	0.374012659
cg08767286	GBX2	8.60E-06	0.373983861
cg13886354		0.000187194	0.373972709
cg24943627		3.34E-05	0.373972193
cg02590345	LPPR1	1.50E-05	0.373962057
cg19192585		0.000357885	0.373961142
cg16490692	GGT7	1.50E-05	0.373930883
cg03694713	NKX2-6	0.000357885	0.373924201
cg19927885	LPIN3	0.001376172	0.373924014
cg03932308	ETV1;ETV1;ETV1;ETV1	7.23E-05	0.373900334
cg03115558	NODAL	8.60E-06	0.373873613
cg25486957		9.24E-05	0.373870659
cg05764628	SPTBN4	8.60E-06	0.373864546
cg12833207	STEAP4;STEAP4	0.000187194	0.373863923
cg12847554	SIM1	7.23E-05	0.373855225
cg07720188	TNFRSF19	0.003189589	0.373845501
cg15070848	WNT5A	0.000357885	0.373827135
cg11313468	HNRNPUL1;HNRNPUL1	9.24E-05	0.373819684
cg23756251	PEX5L	8.60E-06	0.373815519
cg15198235		0.000955793	0.373813164
cg00795024		3.34E-05	0.373803757
cg06100807	RHCG	4.34E-05	0.373802902
cg02099543	FBLN1;FBLN1;FBLN1;FBLN1	0.002304201	0.37379337
cg02039267	VAX1;VAX1	1.96E-05	0.373788549
cg14088357	HIF3A;HIF3A	0.000357885	0.373785951
cg15577178	CACNA2D3	0.000357885	0.373758924
cg00270789	GPT2;GPT2	8.60E-06	0.373741245
cg09772661	CLEC4G	0.000233422	0.373725509
cg11667451	TOX2	0.001147425	0.373679314
cg06068369	PREP	1.96E-05	0.373667235
cg25555059	SLC27A6;SLC27A6;SLC27A6	2.58E-05	0.373664955
cg13726218	DACH1;DACH1;DACH1	5.59E-05	0.373660482
cg01245966	ADCY8	1.50E-05	0.373656933
cg23578346		8.60E-06	0.373651016
cg03535659	CYP7B1	0.000290372	0.373650705
cg21197871	C8orf38	0.000117986	0.373648256
cg12671082	PID1;PID1	5.59E-05	0.373647955
cg11149930		0.000187194	0.373643605
cg04367351	PLA2R1;PLA2R1	5.59E-05	0.373633253
cg00547445	GPR120	5.59E-05	0.373629394
cg14159336	HIST3H2A;HIST3H2BB	8.60E-06	0.373623272
cg20100910		5.59E-05	0.373607032

cg23638252		7.23E-05	0.373541086
cg04038179		4.34E-05	0.373540495
cg14872657	HECW1	0.000537905	0.373533215
cg16650717	NBL1;NBL1	4.34E-05	0.373526214
cg16519587		0.000290372	0.373513447
cg12298745		0.000117986	0.373501608
cg13458645	PITX2;PITX2;PITX2	0.000117986	0.373492156
cg18629427	GRID2	1.50E-05	0.373474478
cg01187920	CARTPT	1.11E-05	0.373474033
cg01503065	DCHS2;DCHS2	2.58E-05	0.373470847
cg11586570		9.24E-05	0.373464793
cg00551647	CYYR1	0.000117986	0.373456451
cg09106624	HOXC13	1.11E-05	0.373449305
cg23037386	HCN1	1.11E-05	0.373448196
cg24081764	PRKCG	0.000148457	0.373446664
cg24542441	SEPT10;ANKRD57;SEPT10	5.59E-05	0.373445881
cg26081162	C6orf141;C6orf141	0.000148457	0.373445055
cg11333886	DSCAML1	0.000148457	0.373423247
cg15899948	SLC27A6;SLC27A6	4.34E-05	0.373400167
cg14266770	VAV2;VAV2	7.23E-05	0.373396455
cg11355135	SLC5A7	2.58E-05	0.373394814
cg02124514	LOC389332	5.59E-05	0.373330271
cg15703773		4.34E-05	0.373308966
cg22794304		4.34E-05	0.373297999
cg17547875		0.000290372	0.373279166
cg27085741	CLCN1	0.000955793	0.373278308
cg01088572	GPR126;GPR126;GPR126;GPR126	5.59E-05	0.373273926
cg08832220	WNT5A	5.11E-05	0.373240908
cg23782616	HMSD	0.004357816	0.373239493
cg08985029	TBX5;TBX5;TBX5	0.00043997	0.373238602
cg21602257	PLTP;PLTP	3.34E-05	0.373228735
cg13785123	ENO1	7.23E-05	0.373228146
cg15634398	LYPD5	9.24E-05	0.373225365
cg22892860	NRXN3;NRXN3;NRXN3;NRXN3;NRXN3	1.50E-05	0.373207667
cg14054928	CA10;CA10;CA10;CA10	1.50E-05	0.373198041
cg05397631		1.50E-05	0.373187144
cg26618041	CILP2	1.50E-05	0.373183773
cg16350446	DCAF17;DCAF17;DCAF17	0.000654035	0.373182849
cg13540171		0.000233422	0.373177491
cg26056277		1.50E-05	0.373104584
cg11554266	ANKS1B;ANKS1B;ANKS1B	3.34E-05	0.373094581
cg15170605	ADD2;ADD2;ADD2;ADD2;ADD2	0.0050758	0.373080141
cg14270687	TMEM132E	3.34E-05	0.373078654
cg15685268	IGSF9B	2.58E-05	0.373069913
cg20952286	CCDC37	2.58E-05	0.373061252
cg26962618		0.000654035	0.373049967
cg22829671	CXCL3	0.000791389	0.373039553
cg26875805	ILDR2	8.60E-06	0.373025975
cg00580978	LOC283392;LOC283392;TRHDE	0.000290372	0.373023967
cg03534453	PCDHB17	9.24E-05	0.373023625
cg12764034	LHX8	0.000357885	0.373003448
cg01544270	EXD3	5.59E-05	0.372989929
cg13547574	STEAP2;STEAP2	7.23E-05	0.372981545
cg22010052	LVRN	8.60E-06	0.372970723
cg12682382	UBE2W;UBE2W	0.000187194	0.372963857
cg17361803	C10orf41;C10orf41	1.50E-05	0.372941872
cg09439204		0.000654035	0.372911745
cg00423826		1.11E-05	0.372910979
cg08121755		4.34E-05	0.372910329
cg03613077	RGS7	2.58E-05	0.372903253

cg26002008	LPCAT1	0.000117986	0.372883656
cg24791428		8.60E-06	0.372871811
cg10273340	GNAO1;GNAO1;LOC283856	0.000117986	0.372855042
cg05667818	ST7;ST7;ST7OT2	0.000537905	0.372834835
cg08378830		0.001639597	0.372829044
cg22724500	RFX4	0.000187194	0.372827761
cg16061498		1.96E-05	0.372817529
cg13927566		9.24E-05	0.372796688
cg00937262	TMEM229A	0.000117986	0.372780728
cg20040743	NECAB1	8.60E-06	0.37277484
cg26554567	CACNA1A;CACNA1A	0.000290372	0.372771688
cg14650610	SPOCK1	0.000654035	0.372771207
cg13806939		0.001639597	0.37276531
cg20563468		1.96E-05	0.372762737
cg18325315	GTF2IRD1;GTF2IRD1	0.000357885	0.372759664
cg27274706	TMEM45B;TMEM45B	4.34E-05	0.372753232
cg07207220	LRAT	2.08E-05	0.372751279
cg22660207	SCRG1	1.96E-05	0.372742832
cg08865099	EVX1	0.000537905	0.372703023
cg24673742	GRIK3	0.000955793	0.372700954
cg01754525		1.96E-05	0.37268636
cg10583000	CNGA3;CNGA3	9.24E-05	0.372671029
cg09694051	MED21	5.59E-05	0.372667856
cg02511231	TLX3	0.001376172	0.372653291
cg05168033	EFEMP1;EFEMP1;EFEMP1	8.60E-06	0.372641906
cg24168914	SLC7A2	0.00043997	0.372599925
cg23615741		1.11E-05	0.372588608
cg23241637	ZNF804A	1.50E-05	0.372580906
cg23962250	C10orf93	9.24E-05	0.372567136
cg03058660	GRIN2D	0.000791389	0.372564537
cg09442828	ADRB3	1.11E-05	0.372552376
cg19315202	TMPRSS2;TMPRSS2	0.000955793	0.372537722
cg24359323	FGF3	0.000357885	0.372527059
cg16754467		0.000148457	0.372511833
cg19039028	COL25A1;COL25A1;COL25A1;COL25A1	8.60E-06	0.372506595
cg22315453	TFAP2A;TFAP2A;TFAP2A	5.59E-05	0.372502555
cg08729031		0.001948248	0.372498707
cg16033053	MKX	0.000117986	0.372472092
cg21694941	PENK;PENK;PENK	1.11E-05	0.372463418
cg27625055	PTPRT;PTPRT	5.59E-05	0.372437039
cg17439757	ADAMTS19	1.50E-05	0.372423569
cg15457217	WDFY1	3.34E-05	0.372419951
cg02601140		1.50E-05	0.372408337
cg17713539		0.000117986	0.37239563
cg26357833	C4orf31	0.000148457	0.372382844
cg27117792		0.00043997	0.372379426
cg06382344	TBR1	0.00041966	0.372366845
cg05238917		3.34E-05	0.372363457
cg00312553	C5orf52	3.34E-05	0.372362407
cg22891500		4.34E-05	0.372358084
cg18517369		5.59E-05	0.372357836
cg09135551	TMEM26;TMEM26	2.58E-05	0.372355639
cg18015746	LPL	0.000187194	0.372345844
cg17305266	FOXF2	0.000117986	0.372320694
cg11226708	MORN4;MORN4	2.58E-05	0.372293511
cg08175413	LNPEP;LNPEP;LNPEP	0.000955793	0.372293437
cg05555207	TBX5;TBX5	5.59E-05	0.372291625
cg21176643	RFX4	0.000537905	0.372276522
cg18290624	SLC6A11	7.23E-05	0.372257129
cg03356900	QRFPR	8.60E-06	0.372245927

cg01150683		3.34E-05	0.372192564
cg21215550		9.24E-05	0.372189365
cg04460364	NDEL1;NDEL1	3.34E-05	0.372189302
cg15317068		1.96E-05	0.372182421
cg27112666		1.11E-05	0.372153705
cg15212349	SLC6A3	8.60E-06	0.372146963
cg20068209	TMEM30A;TMEM30A	0.000537905	0.372141014
cg04932551	FOXA1	0.000117986	0.372131627
cg15595502		3.34E-05	0.372120006
cg14615559	LCN2	0.000233422	0.37211238
cg22570122	CBLN1	0.000290372	0.372095232
cg02677635	FAM125B;FAM125B	5.59E-05	0.372092516
cg27539746	KIAA1598;KIAA1598	8.60E-06	0.372092418
cg11990309	HTR1E	0.000233422	0.372069173
cg21082028		0.000537905	0.372065909
cg23813514	GRID1	0.000187194	0.372045134
cg26833652	MYBPC1;MYBPC1;MYBPC1;MYBPC1	3.34E-05	0.372019457
cg15710245	BDNF;BDNF;BDNF;BDNF;BDNF;BDNF;B	8.60E-06	0.372019192
cg20613972	PLAC8;PLAC8	3.34E-05	0.372010663
cg00221718		0.000537905	0.3719863
cg20514322	NCKAP5;NCKAP5	8.60E-06	0.371972459
cg11229862	CCDC37	4.34E-05	0.37197162
cg13713172		0.002714607	0.371955566
cg01187505	ANO3	3.34E-05	0.371944423
cg06290070	EYA1	0.000654035	0.371909535
cg21428681	NKX3-1	4.34E-05	0.371907439
cg24415066		9.24E-05	0.371853286
cg01568319	SMOC2;SMOC2;SMOC2;SMOC2	0.000233422	0.371843416
cg24664038		1.50E-05	0.371830197
cg15772216	SYNPR	1.96E-05	0.371794233
cg12374721	C17orf93;PRAC	0.000187194	0.371782742
cg17862113	MYOD1	1.11E-05	0.371781379
cg27606567		0.000955793	0.371769773
cg05608777	DOCK1	0.000290372	0.371751637
cg26348902		8.60E-06	0.371751484
cg25723459	ST3GAL4	0.000537905	0.371714078
cg27380915		0.000290372	0.371705529
cg09763162		1.11E-05	0.371683568
cg01672943	PAX9	0.000705485	0.371671579
cg09174233	SH3GL2	8.60E-06	0.371669599
cg10994430	PALM2;PALM2-AKAP2;PALM2;PALM2-AKAP2	8.60E-06	0.371662589
cg06900404		1.96E-05	0.37165121
cg07640648	DLL3;DLL3	4.34E-05	0.371627879
cg16763443		1.50E-05	0.371626754
cg04024095	VSTM2A;VSTM2A	8.60E-06	0.371625354
cg23205387		1.50E-05	0.371600958
cg08959396	C16orf73;C16orf73	3.34E-05	0.371572154
cg16055869	CXCL5	1.96E-05	0.371565896
cg23241456	CACNA1E	7.23E-05	0.371565236
cg22330512	WSCD2	5.59E-05	0.371546165
cg01392772	HLF;HLF	0.000357885	0.371543248
cg00473179		0.000148457	0.371539518
cg27100436	DPY19L2P2;DPY19L2P2	8.60E-06	0.371527093
cg07961512		3.34E-05	0.371518088
cg00748875	PCDHA1;PCDHA1;PCDHA2;PCDHA2	0.000537905	0.371516975
cg27045999	C15orf26	2.58E-05	0.371486178
cg07190535	PDE4D;PDE4D	0.000148457	0.371473445
cg06815950	CUX2	0.001376172	0.371471173
cg22491680	HAL	2.58E-05	0.371458376
cg18722790	BMPR1B	0.00043997	0.371457921

cg13460643	G0S2;G0S2	0.001887355	0.371430933
cg17222829	SHANK2;SHANK2	0.000117986	0.371414204
cg13303459	SH3BP4	7.23E-05	0.371411028
cg02263813	MT1A;MT1A	4.34E-05	0.371398586
cg15167959		1.96E-05	0.371389236
cg20881910	NTM;NTM;NTM;NTM	4.34E-05	0.371359031
cg01818121	GRAMD2	0.000334462	0.371340287
cg17852482	TBCD	4.34E-05	0.371339259
cg04193970		2.58E-05	0.371338418
cg18845189		0.000223017	0.371332623
cg01229567	MIB2;MIB2;MIB2;MIB2;MIB2;MIB2	0.000654035	0.3713248
cg12810701	GPR172B;GPR172B	0.000290372	0.371310837
cg15929797	NKX2-5;NKX2-5;NKX2-5	0.000791389	0.371299658
cg25096566	MAP7	0.004357816	0.371281549
cg14181409	RCVRN	7.23E-05	0.371277217
cg11155924	SHANK2;SHANK2	0.001376172	0.371269787
cg20082357	ATP6V0A1;ATP6V0A1;ATP6V0A1	1.96E-05	0.371267096
cg11241436	NMNAT2	0.000233422	0.371266234
cg02637078	SYT5	1.96E-05	0.371253004
cg14056110	CA10;CA10;CA10	4.34E-05	0.371246508
cg21717959	PACRG;PACRG;PARK2;PARK2;PACRG;PARK	0.000233422	0.371241345
cg15984718	UNC5C	2.58E-05	0.371226359
cg26666835		0.000510765	0.371221554
cg09180848	BNC1	2.58E-05	0.371179486
cg16364121		0.000233422	0.371172758
cg12337525		3.34E-05	0.371144206
cg02510267	NELL1;NELL1	0.001639597	0.371110737
cg26838949	KIF21B	0.001147425	0.371103172
cg24079702	FHL2;FHL2;FHL2;FHL2	8.60E-06	0.371089638
cg14324502	ISCA1	0.001147425	0.371087199
cg03313542	TMEM209	0.002714607	0.37108407
cg18729704	PDCD11	0.000148457	0.371071815
cg03238797	ADAMTS18;ADAMTS18	7.23E-05	0.371059335
cg16832648	CCNY;CCNY	5.59E-05	0.37105417
cg22608170	MAPK4	0.000357885	0.371034947
cg05412990	WDR35;WDR35	1.96E-05	0.371027083
cg15612947	TRIO	0.001639597	0.371024807
cg03036592		0.000357885	0.371023553
cg26578682	IRX2;IRX2	5.59E-05	0.37101261
cg09486567	OVOL2;OVOL2	4.34E-05	0.371007326
cg15589153		8.60E-06	0.370994177
cg26337277	TMPRSS2;TMPRSS2	0.001147425	0.370992318
cg15007156	KIAA1026;KIAA1026	1.11E-05	0.370985907
cg22081832	ADAMTS9	1.11E-05	0.370981726
cg24586758	PCDHB3	1.96E-05	0.370977593
cg26307218		8.60E-06	0.370958544
cg09578028	HOXD9	1.96E-05	0.370952088
cg06905692	ALK;ALK	0.000290372	0.370936594
cg09817427	TBX18	8.60E-06	0.370928324
cg16734795	GPR77	1.50E-05	0.370891315
cg03506979	FAM171B;FAM171B	9.24E-05	0.370883329
cg27341926	GFRA1;GFRA1;GFRA1;GFRA1	0.001376172	0.370880189
cg06639320	FHL2;FHL2;FHL2;FHL2	4.34E-05	0.370874239
cg05654364	CSNK1A1;CSNK1A1	0.006823935	0.370867093
cg07566169	HOXD11	0.000357885	0.370848895
cg12297590	ZDHHC14;ZDHHC14	0.004357816	0.370808506
cg08206959	GABRA2;GABRA2	4.34E-05	0.370806194
cg21510348		1.96E-05	0.370801275
cg23661721		0.000233422	0.370798577
cg14311320	GPR37	1.96E-05	0.370767667

cg14879760		4.34E-05	0.370764239
cg03983647	CDH6	5.59E-05	0.370760996
cg00399175	FAM59B	7.23E-05	0.370760189
cg07076175	FAM150A	1.96E-05	0.370746289
cg23779890	GDAP1;GDAP1	1.50E-05	0.370713624
cg24427660	PNPLA2	5.59E-05	0.370711574
cg10979547	VSNL1	1.50E-05	0.370708275
cg02213139	WDR63	1.11E-05	0.370700482
cg00950265		1.11E-05	0.370696216
cg17544952	MEOX2	1.50E-05	0.370691484
cg23616527		8.60E-06	0.370687938
cg03774803	BOLL;BOLL;BOLL	2.58E-05	0.370678674
cg21283680	SH3BP5	4.34E-05	0.370668436
cg18912484		0.000187194	0.3706664
cg24869535	NPNT	9.24E-05	0.370650235
cg03778029	CCDC68;CCDC68	7.23E-05	0.370626596
cg24503056		0.000117986	0.370592396
cg14339043	ANKRD24	5.59E-05	0.370587921
cg06390643	ETV5	0.000117986	0.370587895
cg00926502	BIVM;BIVM;KDELC1;BIVM;BIVM	4.34E-05	0.370586615
cg26267340	NRG1	1.50E-05	0.370581083
cg22044566	FAM84A	2.58E-05	0.370580454
cg22375763	LRG1	7.23E-05	0.370572926
cg13592931	PCDHA6;PCDHA2;PCDHA1;PCDHA9;PCDHA7	8.60E-06	0.370544056
cg19204131		3.34E-05	0.370537771
cg23956648	IGF2BP2;IGF2BP2	2.58E-05	0.370537462
cg06856840	FAH	0.000791389	0.370528033
cg10813544		5.78E-05	0.370480768
cg19786627	FOXG1	8.60E-06	0.37047492
cg10162522	KCNH5;KCNH5;KCNH5	7.23E-05	0.370436222
cg11104088	TAF3	0.001948248	0.370414869
cg08334278		8.60E-06	0.370408767
cg22458194	DRD2;DRD2	1.11E-05	0.370393533
cg22234080	NEFH	1.11E-05	0.370386105
cg13918640		5.59E-05	0.370382435
cg19674851		0.002714607	0.370379489
cg05141147	LHX5	5.59E-05	0.370375852
cg07854547	SNTG1	1.96E-05	0.370368766
cg25766271		8.60E-06	0.370366414
cg01644741	MIR1259;SNORD12B;C20orf199;C20orf199;SN	0.000148457	0.370359089
cg20010506	FAM19A2;FAM19A2	0.000290372	0.370358759
cg00752099	ATOH1	5.59E-05	0.370355378
cg21781157	ZNFX1	4.34E-05	0.370343963
cg18217235		1.11E-05	0.370337137
cg18192919	TMEM26;TMEM26	8.60E-06	0.370335794
cg03637878	JAM3	0.000187194	0.370325418
cg14081465	ASTN2;ASTN2;ASTN2	0.000654035	0.370298604
cg09472203	AP3B2;AP3B2	4.34E-05	0.370294657
cg05101674	DOK5	5.59E-05	0.370259123
cg09130963	DHCR24	1.96E-05	0.370258283
cg03236397	HPSE2;HPSE2;HPSE2;HPSE2	1.96E-05	0.370249307
cg13089335	POU2F1	4.34E-05	0.37024739
cg24471254	ACTL6B	8.60E-06	0.370245867
cg26526374		0.00043997	0.370244848
cg23777958	RALYL;RALYL;RALYL;RALYL;RALYL;RALYL;R	9.24E-05	0.370244123
cg12529672	NOTO	4.34E-05	0.370243098
cg07724670		2.58E-05	0.37021569
cg00688447		0.000537905	0.370214052
cg06737561	CHAT;CHAT;CHAT;CHAT;CHAT;CHAT;CHAT	7.23E-05	0.370209481
cg19608455	MAPK4;MAPK4	0.000654035	0.370185891

cg18535415	KIF21B	2.58E-05	0.37018257
cg12550055	C22orf45;C22orf45;UPB1	8.60E-06	0.37016575
cg01824410		0.001376172	0.370164802
cg20406460		0.000537905	0.370158119
cg02073887		0.000537905	0.370152026
cg10957242	STOX2	0.000158823	0.370150927
cg26344233	FHL2;FHL2;FHL2;FHL2	1.96E-05	0.370149224
cg06823060	CMIP;CMIP	2.58E-05	0.370104416
cg02172150	WBSCR17	0.000117986	0.370084794
cg02683197		0.000148457	0.370081809
cg26847100	SLC44A2;SLC44A2	0.000290372	0.370081288
cg00756058	DZIP1;DZIP1;DZIP1;DZIP1	0.000187194	0.370075331
cg20653075	MIR124-2	1.50E-05	0.370075218
cg00973653		7.23E-05	0.370070906
cg01572652	C12orf42;C12orf42	8.60E-06	0.370058146
cg18067134	FOXP1	0.000187194	0.370052793
cg06893389		0.000117986	0.370017164
cg10542584	FEZF2	0.000117986	0.370013049
cg21532659	NAV2;NAV2;NAV2	0.001639597	0.370010103
cg24041917		1.11E-05	0.370008288
cg27111463	FOXI3	9.24E-05	0.370006678
cg17208953		3.34E-05	0.370004291
cg27114120	PROKR2	0.000117986	0.369991549
cg18698788		0.003189589	0.369991158
cg13187764		0.000233422	0.369990717
cg12420104	DMRT3	1.11E-05	0.369981053
cg18162583		5.59E-05	0.369964865
cg14281591	KLF5	0.000290372	0.369935883
cg10330371	CADPS2;CADPS2;CADPS2	9.24E-05	0.369928698
cg19464804		1.96E-05	0.369926436
cg17038667	FGF3	0.000946184	0.369898443
cg07913294	AGBL4;AGBL4	8.60E-06	0.369894802
cg10491628		0.000290372	0.369894231
cg10967023		2.58E-05	0.369882151
cg14523804		1.50E-05	0.369874269
cg18621091		0.000233422	0.369856991
cg14584031		0.000117986	0.369856453
cg00980622		0.000148457	0.369834295
cg13057663		0.000290372	0.369822355
cg14249174	ARFIP1;ARFIP1;ARFIP1	0.000705485	0.369816754
cg15991405	HOXD9	0.000537905	0.369803579
cg22962883	NNMT	0.000187194	0.369797114
cg01914621		4.34E-05	0.369783035
cg11231701	CDK5RAP1;CDK5RAP1	0.000357885	0.369783004
cg27196745	PTPRO;PTPRO	1.96E-05	0.36977617
cg16532600	HAND1	3.34E-05	0.369772564
cg22082709	AGTR1;AGTR1;AGTR1;AGTR1	5.59E-05	0.369770678
cg04105416	RADIL	0.000148457	0.369755029
cg22353097	SLC22A15	0.000357885	0.369748348
cg11831981	SCG3;SCG3;SCG3;SCG3	0.000205664	0.369740018
cg02655630	SLC12A5;SLC12A5	1.50E-05	0.369722034
cg12817908	NMNAT3	0.001948248	0.369714138
cg03464847	DNAH9	8.60E-06	0.369708675
cg21058391		1.50E-05	0.369705634
cg09645336	C5orf43	4.34E-05	0.369628869
cg07850592	TRIM67	1.50E-05	0.369622116
cg07341378	TBC1D12;TBC1D12	2.58E-05	0.369618046
cg01556552		2.58E-05	0.369602173
cg23989004	ZNF709	0.000148457	0.36959818
cg10281977	TMEM179	2.08E-05	0.369577985

cg11142406	SOX2OT;SOX2	7.23E-05	0.369577217
cg10425827	FAM184A;FAM184A	5.59E-05	0.369562931
cg19032066	PAX9	2.58E-05	0.369561514
cg23044884	RBPMS;RBPMS;RBPMS;RBPMS	0.0050758	0.369556348
cg01655831	GREB1L	3.34E-05	0.369542235
cg13730105	TLR4;TLR4;TLR4	0.001147425	0.369530314
cg06187947	GULP1	8.60E-06	0.36952182
cg18710784	OPCML;OPCML	5.59E-05	0.369517292
cg17696377	ERN2	8.60E-06	0.36951507
cg01816304	PARVA	8.60E-06	0.369511203
cg26012941	WDR63	1.50E-05	0.369479623
cg26440289	SNX31	0.000357885	0.369475647
cg26001333	MAP1B	0.000117986	0.369474777
cg17012160	FMN2	5.59E-05	0.369472546
cg02547394	SOX1	0.000148457	0.369459519
cg01226811	KCNJ8;KCNJ8	8.60E-06	0.369459353
cg16865446	PAX6;PAX6;PAX6	9.24E-05	0.369445928
cg03573446	BMP7	0.000365373	0.369433989
cg14811102	ONECUT1	8.60E-06	0.369432036
cg07729059		0.000357885	0.369426356
cg06706156		0.000187194	0.369397644
cg06693487		4.34E-05	0.369381719
cg07108443		1.50E-05	0.369360431
cg23688510	T;T	1.96E-05	0.36935309
cg10899099		8.60E-06	0.369350676
cg12688240	NANOG	3.34E-05	0.369346196
cg23676151	NR2E1	7.23E-05	0.369342139
cg25556432		9.24E-05	0.369341328
cg26559209		0.000357885	0.369330425
cg02866628	VSTM2A	0.000187194	0.369306797
cg18541254		8.60E-06	0.369292315
cg14090923	ASTN2;ASTN2;ASTN2	0.000654035	0.369275475
cg14931230	LYPD5	0.000357885	0.369266747
cg03151187	BRUNOL4;BRUNOL4;BRUNOL4;BRUNOL4	4.34E-05	0.36926297
cg15129608	PRKCG	0.00043997	0.36924493
cg02885007	HOXD9	8.60E-06	0.369183
cg01247628	SEMA6D;SEMA6D;SEMA6D;SEMA6D;SEMA6D	0.000791389	0.369181916
cg02974928	CACNA1E	0.000537905	0.369164378
cg04792330		0.000233422	0.369159169
cg21041472	HIVEP1	3.34E-05	0.369155266
cg01419577		0.000117986	0.369134452
cg18075315	RFX4	0.001376172	0.369128935
cg10981651		3.34E-05	0.369126271
cg02150988	HOXD1	8.60E-06	0.369120836
cg02957057	NID1	0.000334462	0.369119829
cg18381051	PRDM16;PRDM16	9.24E-05	0.369100568
cg01429449		2.58E-05	0.369090114
cg22865720	PRKCZ	0.002714607	0.369087603
cg08850461		0.000791389	0.369081027
cg10756127	TLL1	0.000148457	0.3690752
cg18276112	FOXK1	0.001948248	0.369070313
cg02355420	EPHA5;EPHA5	5.59E-05	0.369066745
cg03234732	ZNF454	0.000187194	0.369062044
cg09251429	ROBO3	0.000233422	0.369061032
cg18860954	ZNF814	1.96E-05	0.369047443
cg05394500	ADRA2C	0.002927025	0.36904674
cg19456953	PCSK1	0.000955793	0.369037777
cg04684656		3.34E-05	0.369026455
cg12903638	UNC80;UNC80	0.000357885	0.369014635
cg17331494		1.50E-05	0.36901194

cg14919250	XKR6	5.59E-05	0.368992625
cg06230847	TRIM39;TRIM39	3.34E-05	0.368981755
cg27297088	FAM163A	1.50E-05	0.368974192
cg09749813	TBX18	2.58E-05	0.368973888
cg03474903	KIAA1755	7.23E-05	0.368953931
cg02311193		8.60E-06	0.368952379
cg11689407		0.000187194	0.368946099
cg26736540	TFAP2C	5.59E-05	0.368945813
cg20218614	FAM83A;FAM83A;FAM83A;FAM83A	0.000290372	0.368918505
cg01440934	MKNK1;MKNK1;MKNK1;MKNK1;MKNK1	0.000233422	0.368905235
cg20547653	SLC5A7	1.96E-05	0.368897164
cg25130385	C9orf98	7.23E-05	0.368892544
cg11368643	PCDHB15	8.60E-06	0.368882287
cg06779110	C14orf23;C14orf23	4.34E-05	0.368876007
cg03933572	LYPD6	8.60E-06	0.368875217
cg15235999	ZNF578	9.24E-05	0.368795683
cg15012484		1.96E-05	0.368774876
cg24846199		1.11E-05	0.368772664
cg23575688		0.0050758	0.368758914
cg21224380	SSH1;SSH1	9.24E-05	0.368758079
cg19324633		5.59E-05	0.368756713
cg01843946		4.34E-05	0.368746173
cg05155965	NR2F2;NR2F2	1.50E-05	0.368723947
cg08017928	MNX1;MNX1;MNX1	1.50E-05	0.368686199
cg10444382	RFX4	8.60E-06	0.368672214
cg22517656		0.000233422	0.368669547
cg07814567	DAPP1	3.34E-05	0.368581888
cg23133011		0.000117986	0.368580378
cg04525757	FOXG1	0.000233422	0.368573492
cg09041678	PAX6	1.50E-05	0.368551559
cg03611555	SLC25A21;SLC25A21;SLC25A21;LOC1001297	1.11E-05	0.368533151
cg00116554	SNAI2;SNAI2	2.58E-05	0.368504587
cg14574996	ZNF414;ZNF414	0.001948248	0.368499621
cg06672560	CBLN4	1.96E-05	0.368498237
cg21319323		0.001291055	0.368497333
cg04115680	SRRM3	0.000791389	0.368494336
cg02229261	HAS1	8.60E-06	0.368446547
cg27430561		0.000187194	0.368436106
cg10313337	CDH1	9.24E-05	0.36835491
cg23911433		0.000187194	0.368316965
cg04670857	CTNNA2;CTNNA2;CTNNA2;CTNNA2	0.000357885	0.36831586
cg04425551	TRIM39;TRIM39	0.000117986	0.368310853
cg02919422	SOX17;SOX17	2.58E-05	0.368297951
cg01535698	FMN2	0.00041966	0.368276174
cg16650529		0.000537905	0.368274938
cg26750893		4.34E-05	0.368262083
cg27555382	EIF5A2	0.000187194	0.368239691
cg22763680	SLFN12L	2.58E-05	0.368226131
cg13594903	STOML2	1.96E-05	0.368149903
cg17000199	FUT9	0.000357885	0.368143479
cg05930881	GATA4	0.000357885	0.368141723
cg00866976	GNAO1;GNAO1;LOC283856	0.000205664	0.36813292
cg12934884		0.00043997	0.368130869
cg25869196	IL1RL1;IL1RL1	0.000117986	0.368125396
cg00436496		7.23E-05	0.36811294
cg18459489	VAX1	1.11E-05	0.368112568
cg17255712	SOX9	0.000117986	0.368103807
cg19206040	GRIK3	0.001376172	0.368102603
cg26212180	VAX1;VAX1	1.96E-05	0.368090984
cg07461772	SMPD3	9.24E-05	0.368087678

cg19748424	LOC255167;LOC255167	1.96E-05	0.368036103
cg09066326	PCDHGA4;PCDHGA6;PCDHGA1;PCDHGA5;PC	2.58E-05	0.368026225
cg02519037	TACC2;TACC2	3.34E-05	0.367986205
cg17704189	FEZF1;FEZF1	1.50E-05	0.367984794
cg09759458		4.34E-05	0.367979624
cg04365481	MST1P9	1.50E-05	0.367963416
cg15333318	NECAB1	4.34E-05	0.367961925
cg27343150	ANO5;ANO5	1.96E-05	0.367954072
cg14324370		0.000791389	0.367903707
cg23767994	DLX6AS	9.24E-05	0.367866302
cg08967802	C17orf102;TMEM132E	0.000148457	0.3678582
cg08418670	SH3GL1	2.58E-05	0.367843818
cg02605370	RTBDN	0.000654035	0.367828395
cg02983163		0.000187194	0.367796683
cg09481483		0.001376172	0.367772808
cg21852408	C11orf87	1.50E-05	0.367768527
cg05388492	ESRRG	0.000233422	0.367763202
cg14537533	KRT7	0.000955793	0.367738052
cg25755575	ESRRG	8.60E-06	0.367731095
cg24926276	LRG1	0.000117986	0.367730926
cg10005978	DMRT3	2.58E-05	0.367714207
cg00099976	SHISA2	1.96E-05	0.367708141
cg16000637	UNCX	0.000791389	0.367690775
cg00772000	NHLRC1	1.96E-05	0.367679264
cg13259925	LRAT	8.60E-06	0.367633978
cg01892516	FOXA1	0.002040485	0.367631537
cg18295183	GABRG2;GABRG2;GABRG2;GABRG2;GABRG	2.58E-05	0.367623607
cg22501604		0.003189589	0.367622159
cg25449484	NR2F1	5.59E-05	0.367620278
cg22487414	NPHS1	5.59E-05	0.367617814
cg08044097	SOX17	0.000537905	0.367585083
cg18415791	OSBPL5;OSBPL5;OSBPL5	2.58E-05	0.367578018
cg20018912	INSM2	0.000290372	0.367576081
cg08526991	SOX11	0.004357816	0.367575231
cg12212555	KCNK13	4.34E-05	0.367562558
cg01915516	TP73	0.001948248	0.367528603
cg08857797	VPS25	4.34E-05	0.367519717
cg24080008		0.000654035	0.367503501
cg21158075	PRR4;PRH1	0.000187194	0.367500328
cg12230289		0.002304201	0.367459429
cg19047292	DKFZP434H168;GNAO1;GNAO1	0.000654035	0.367447545
cg14546394	MSC	8.60E-06	0.367444751
cg09851545		0.000791389	0.367429162
cg25915838	PGR	0.000117986	0.367405366
cg18421529	MRAS;MRAS	0.000187194	0.36740419
cg10274453		1.50E-05	0.367374153
cg06470822		8.60E-06	0.367368799
cg24381412		0.000290372	0.367343608
cg20273670		8.60E-06	0.367342595
cg06857116	ZSWIM7;ZSWIM7	0.000537905	0.367328574
cg18324707	DGKI	0.000187194	0.367326146
cg02575948		7.23E-05	0.36729247
cg00698685	DRD1	2.58E-05	0.367286153
cg19671120	CNGA3;CNGA3;CNGA3;CNGA3	7.23E-05	0.367284539
cg02063759		0.000117986	0.367276896
cg11677857	MMEL1	0.000537905	0.367255398
cg00232735	OPCML	0.000117986	0.367243438
cg07316846		0.000117986	0.367238013
cg14123232		1.11E-05	0.367226883
cg00799984	NDRG1;NDRG1	1.11E-05	0.36721305

cg07853758	HECW2	0.000233422	0.367206145
cg23572908	VIPR2	0.001639597	0.367187228
cg12136772	SMPD3	8.60E-06	0.367181564
cg01566592	RIMS2	0.001948248	0.367180126
cg12164472	RGS20;RGS20	0.000654035	0.367176298
cg09923293		7.23E-05	0.367173913
cg22466995	MFAP3L	0.000791389	0.367160444
cg27167224		9.24E-05	0.367151061
cg02009585	PEX5L	1.11E-05	0.367150458
cg02184772		7.23E-05	0.367123337
cg15950068	SNAP91;SNAP91	0.000654035	0.367085462
cg12804010	SYT6	0.000117986	0.367082201
cg06722407	LOC100101938	0.000148457	0.367078147
cg14092626		9.24E-05	0.367074973
cg04094148	BARX2	0.000791389	0.3670658
cg08460041	LMX1A	0.00043997	0.367046935
cg19656974		1.96E-05	0.367045965
cg19607845	FBXO9;FBXO9;FBXO9	0.000654035	0.367028439
cg18839750	PCDHGA2;PCDHGA3;PCDHGA1;PCDHGA3	4.34E-05	0.367027773
cg20707323		9.24E-05	0.367026317
cg14915878	MATN4;MATN4;RBPJL;MATN4	3.34E-05	0.367015023
cg27235148		5.59E-05	0.367014241
cg13537353	ATP8A2	0.001147425	0.366991242
cg07806886	STXBP5L	9.24E-05	0.366988447
cg08734600	NKX1-2	7.23E-05	0.366977533
cg10868817	ZFPM2	0.00043997	0.366977268
cg19430967	TMEM108;TMEM108	5.59E-05	0.366954669
cg22461835	ADRA1A;ADRA1A;ADRA1A;ADRA1A	0.000187194	0.366934854
cg13415073		4.34E-05	0.366906802
cg21535462	KRR1	0.000187194	0.36689643
cg09317884	LMX1A	0.000117986	0.366875817
cg22335490	EPB41L3	0.000205664	0.366871912
cg27175093		1.96E-05	0.366863952
cg05131696		0.001639597	0.366862924
cg09792008	FGF10	0.000187194	0.366862546
cg10460820	ATOH1	1.96E-05	0.366860649
cg19239848	SNAP25;SNAP25	2.58E-05	0.366850032
cg00810208	IDUA	7.23E-05	0.366835877
cg25853622	LPP;LPP;LPP	0.00043997	0.366835709
cg07745624	KCNIP4	0.000117986	0.366819058
cg22840076	HNRNPUL1;HNRNPUL1	1.11E-05	0.366777053
cg07544837	FAM13C;FAM13C;FAM13C;FAM13C;FAM13C;F	1.96E-05	0.366769608
cg13197216		3.34E-05	0.366762455
cg14769207	SIM2;SIM2	0.000791389	0.366760678
cg19850080	SLC19A3	8.60E-06	0.366755689
cg06745030	B3GNT7	0.001147425	0.366754617
cg23065100	MLF1;MLF1;MLF1	0.000357885	0.366751371
cg11839815	MGLL;MGLL	0.000290372	0.366717208
cg02421985		7.23E-05	0.366686493
cg01850449	LYPD1;LYPD1	3.34E-05	0.366683705
cg12449916		9.24E-05	0.366676801
cg23979412		1.11E-05	0.366656172
cg08780398	LOC100192378	1.11E-05	0.366645429
cg15565065	HOXB4	0.000654035	0.366631247
cg04084354		0.000117986	0.366622834
cg27059140		9.24E-05	0.366581057
cg04702396	FAM18B2;FAM18B2	0.000791389	0.366563871
cg20982735	PSKH2	8.60E-06	0.366561807
cg19462523	SOX2;SOX2OT;SOX2	0.000117986	0.366555067
cg15852312		1.50E-05	0.366523758

cg15812753	HOXD3	2.58E-05	0.366516022
cg09411999	HOXA10;HOXA10	0.000187194	0.366514075
cg19144866	GOLSYN;GOLSYN;GOLSYN;GOLSYN;GOLSYN	0.000357885	0.366512202
cg15177613	TSKS	9.04E-05	0.36650476
cg17030233		8.60E-06	0.366492408
cg25999722	ALK	0.000290372	0.366482342
cg08104960	NXN	7.23E-05	0.366459151
cg18877252	SV2C	0.000290372	0.366455225
cg02081266		0.004357816	0.366453639
cg00453717	POLD3	0.001639597	0.366450195
cg06899551	FILIP1	8.60E-06	0.366443034
cg15987088	GHSR;GHSR	1.50E-05	0.36643862
cg18122419		1.11E-05	0.366429412
cg17137424	TRIM67	1.11E-05	0.366408209
cg06333800	PRKAG2;PRKAG2	8.60E-06	0.366408001
cg02735446	VASH1	8.60E-06	0.366389242
cg02192746	PILRA;PILRA;PILRA	1.11E-05	0.366355129
cg17065772	NR2C2	0.000233422	0.366353584
cg10746542	SSTR1	1.50E-05	0.366346579
cg01449144		0.000357885	0.366346253
cg21871952		1.50E-05	0.366321853
cg24341611	CA10;CA10;CA10;CA10	9.24E-05	0.366313221
cg09073398	SLIT3;SLIT3	2.58E-05	0.366279429
cg04569615	SLC6A5	5.59E-05	0.366275389
cg26623444		3.34E-05	0.366272928
cg21266559	CHST8;CHST8	1.96E-05	0.366271115
cg16294013	PDE4B;PDE4B	8.60E-06	0.366251119
cg01432663		3.34E-05	0.366234906
cg04205034		0.000290372	0.36622782
cg05671350	P4HA3	1.11E-05	0.366177243
cg05492904	CYP19A1;CYP19A1	0.000148457	0.366155381
cg14835517		9.24E-05	0.366147523
cg16217610	SGMS2	7.23E-05	0.366141559
cg19084726	PDE4DIP	0.000233422	0.366121018
cg05164666	VAX2	0.000117986	0.366113305
cg06980654		0.0050758	0.366087185
cg21074260	SYT13	0.000148457	0.366085543
cg24976385	TBC1D12	0.000117986	0.36608387
cg07860213	PRDM14	0.000537905	0.366082151
cg18318878		9.24E-05	0.366079366
cg23896164	SIM2	3.34E-05	0.366063406
cg09294077	GPHN;GPHN	0.000791389	0.366061085
cg23473285	RIMS1;RIMS1	9.24E-05	0.366047517
cg09166556		0.000290372	0.36604097
cg00644823		8.60E-06	0.366034613
cg25924096	PRDM13	8.60E-06	0.366029799
cg15822765		0.002304201	0.366027291
cg19759671	MGC45800	5.59E-05	0.36601985
cg17211582	IGSF21	2.58E-05	0.366014646
cg02774439	HAND2;NBLA00301;HAND2	1.11E-05	0.365983028
cg08036309		4.34E-05	0.365966013
cg04485065	FBLN5	5.11E-05	0.365957306
cg11898009		4.34E-05	0.36594598
cg27392804		4.34E-05	0.365913072
cg20800086	ISL1	3.34E-05	0.365902831
cg17518931	MPPED2	0.00043997	0.365887134
cg03696599	CNTNAP5	9.24E-05	0.365876227
cg07813031		0.001948248	0.365874989
cg02655972	CDH6	1.96E-05	0.365874955
cg21001198	LRRN4	0.000654035	0.365870869

cg20238412		3.34E-05	0.365870515
cg14913969	GATA6	0.000537905	0.365866065
cg07633851	RPL26L1;LOC100268168;LOC100268168	8.60E-06	0.365841165
cg21031345		2.58E-05	0.365839438
cg11153071	RPTOR;RPTOR	1.50E-05	0.36583797
cg03347018	MSC	1.96E-05	0.365837459
cg11186405		0.000357885	0.365826809
cg02775804		8.60E-06	0.365820013
cg22630755	NEUROG1	0.000233422	0.365815748
cg12140144	FLJ42875;PRDM16;FLJ42875;PRDM16	0.000148457	0.365812896
cg26489413	AMPD3;AMPD3;AMPD3;AMPD3	0.000187194	0.365776903
cg21519701	TEX2	0.001376172	0.365775225
cg19726179	H2AFY2	0.002304201	0.36576155
cg07152894		0.001376172	0.365733225
cg09213102	CALB2;CALB2;CALB2	9.24E-05	0.365728482
cg23845009	ABTB2	0.000187194	0.365725411
cg24277791	LHFPL3	2.58E-05	0.365697906
cg04457979	KCNQ1DN	0.000148457	0.365681938
cg06440519	SYT3;SYT3;SYT3	4.34E-05	0.365679446
cg02105519	PCDHA7;PCDHA12;PCDHA6;PCDHAC1;PCDH	0.000148457	0.365676838
cg16024318	SLC6A7;SLC6A7	0.000148457	0.365672192
cg26215967	SH3GL3;SH3GL3	0.000117986	0.365661471
cg11142981	BICC1	8.60E-06	0.365655428
cg18921954		8.60E-06	0.365650321
cg10655021		0.001639597	0.365646953
cg05568490		8.60E-06	0.365645944
cg14566959	PCDHGA4;PCDHGA6;PCDHGA1;PCDHGA5;PC	0.000117986	0.3656425
cg19947463	C7orf50;C7orf50;C7orf50	0.000148457	0.365635362
cg23817893	CCDC81;CCDC81;CCDC81;CCDC81	0.002714607	0.365618616
cg24595152	ABHD5	3.34E-05	0.365617239
cg17933583		8.60E-06	0.365617061
cg02273041	PRKCDBP	0.000233422	0.365584624
cg07345734	NKX2-1;NKX2-1	9.24E-05	0.365578293
cg25186143	FAM57A	0.000357885	0.365566897
cg13139972	DKK2;DKK2	0.000537905	0.365546328
cg15672768	PCDHGA4;PCDHGA2;PCDHGB2;PCDHGA1;PC	3.34E-05	0.365543703
cg24766229	C17orf99	2.58E-05	0.365538794
cg14514813	PCDHA6;PCDHA2;PCDHA1;PCDHA9;PCDHA7	8.60E-06	0.365529159
cg00691830	MEIS2;MEIS2;MEIS2;MEIS2;MEIS2;MEIS2;MEI	1.96E-05	0.365521547
cg07085827	LOC150786;LOC150786	4.34E-05	0.365517316
cg06211872	TBX3;TBX3	0.000117986	0.365496363
cg03844506		4.34E-05	0.365490904
cg16043144	CPEB1	1.11E-05	0.365473954
cg08616061	PCDHGA4;PCDHGA1;PCDHGA6;PCDHGA5;PC	8.60E-06	0.365465399
cg01261013	MORC3	1.50E-05	0.365460878
cg10751070		5.59E-05	0.36543982
cg03940684	CHL1	0.000955793	0.365437665
cg10591607	GRIK2;GRIK2;GRIK2;GRIK2;GRIK2;GRIK2	1.11E-05	0.365431229
cg20110349	LOC222699	0.000187194	0.365405582
cg01601050	STXBP6	0.000117986	0.365401774
cg13472192		0.000148457	0.365399141
cg16330450	NPBWR1	2.58E-05	0.365394684
cg00884606	NOL4	0.000117986	0.365393897
cg27132391	PRKCDBP	5.59E-05	0.365391236
cg11699845	DPY19L2P4	1.11E-05	0.365375538
cg04188862		0.000537905	0.365375239
cg16179952	SNTG2	1.50E-05	0.365369592
cg21443659		0.000117986	0.365367272
cg16776981	BAHCC1	5.59E-05	0.365366908
cg12059112		0.000537905	0.365363122

cg03405515	MAP9	0.001948248	0.365359012
cg00014998	HOXD4;MIR10B	0.000290372	0.365352811
cg24073122	TP73;WDR8	0.000187194	0.365340225
cg27040468	BICC1	0.000791389	0.365329811
cg09069886		3.34E-05	0.365314374
cg06178563	NKX2-2	7.23E-05	0.36531388
cg03893271	WBSCR17	0.000357885	0.365313495
cg04922029	DARC;DARC;DARC	0.000233422	0.365306167
cg23901852	FAT4	9.24E-05	0.365304274
cg05581878		0.001948248	0.365296524
cg11485152	TRNT1	0.000117986	0.365286952
cg08999807	GPC5	5.59E-05	0.365279388
cg13200848	GLP1R	0.000654035	0.365271932
cg10575261	ZNF454	0.000187194	0.36526204
cg08548498	SLPI	0.000537905	0.365259723
cg00752195		8.60E-06	0.365242669
cg15183258	MGA;MGA	0.000233422	0.365242127
cg11687036		4.34E-05	0.365239404
cg07212852		1.96E-05	0.365239197
cg14345012	MIR663	1.50E-05	0.365219709
cg22558871	NSUN5;NSUN5;NSUN5;NSUN5	0.000122184	0.365216257
cg01765403	SHISA4;SHISA4;SHISA4	5.59E-05	0.365211724
cg20267441	VAX2	0.000537905	0.365203136
cg10308906		0.000205664	0.365195544
cg00405198	TFAP2A	5.59E-05	0.365190291
cg23542968	SFTA3	8.60E-06	0.365176452
cg24352971		7.23E-05	0.365160839
cg19034038	DBX2	3.34E-05	0.365152769
cg00083018		1.96E-05	0.365150986
cg16801887		0.000187194	0.365137783
cg18325192	CSGALNACT1;CSGALNACT1	5.59E-05	0.365117206
cg06971129	EDNRB;EDNRB;EDNRB	0.001147425	0.365117074
cg17040471	CHODL	4.34E-05	0.365116995
cg12066624	LGI2	3.34E-05	0.365093069
cg14957547	CYP1B1	0.000791389	0.365084189
cg19806849	TRIM67	2.58E-05	0.36508201
cg22758714		0.000290372	0.365072956
cg09563922		8.60E-06	0.365069602
cg18036763	PHF21B;PHF21B	3.34E-05	0.365061249
cg18763165		0.000117986	0.365044296
cg17816394	GNG4;GNG4;GNG4;GNG4;GNG4	0.000290372	0.365030278
cg26994526	MYO5B	1.96E-05	0.365010071
cg13581015		0.000791389	0.365009612
cg16790847	ZIC4;ZIC4;ZIC4	2.58E-05	0.364982091
cg00548268	NPTX2	8.60E-06	0.364969134
cg12355110		1.11E-05	0.36495073
cg02018465	MAPK15	3.34E-05	0.364947816
cg17336584	PHACTR1	1.50E-05	0.364947493
cg20807545	ADAMTS18	7.23E-05	0.364928297
cg23262897	PCDHGA2;PCDHGA1;PCDHGA2	7.23E-05	0.364923865
cg11474182	CNTNAP3	1.50E-05	0.364916451
cg14553243	CYP27A1;CYP27A1	3.34E-05	0.364906037
cg11184748		1.11E-05	0.364893635
cg21184415	EMILIN3	1.50E-05	0.364892467
cg01699630	ARG1	4.34E-05	0.364891153
cg08522340	CTSC;CTSC;CTSC	9.24E-05	0.364842579
cg04874782		0.000654035	0.364822365
cg08997628	SOX21	0.00041966	0.364800987
cg22752008	COL4A3;COL4A3;COL4A3;COL4A3;COL4A4	8.60E-06	0.364799351
cg14889768	RAB32	4.34E-05	0.364779229

cg01115380		0.000290372	0.364764024
cg04008252	NPFFR2	0.00043997	0.364760606
cg07609862	MTNR1B	1.11E-05	0.364759687
cg05488770	LHFPL3	7.23E-05	0.364746026
cg09559021	CSMD2	8.60E-06	0.364745296
cg14138540		9.24E-05	0.364738805
cg06484100		4.34E-05	0.364733576
cg12549600		0.000148457	0.364727416
cg00832329	PACS2;PACS2	9.24E-05	0.364723553
cg12323063	MAP2K6	1.96E-05	0.364719546
cg13518604	NFIB;NFIB	7.23E-05	0.364690111
cg10967350	CNTN4	1.96E-05	0.364684357
cg15347189	SST	4.34E-05	0.364683108
cg01070209	SRRM4	0.001147425	0.364666975
cg24488059	PPAPDC1A	0.000148457	0.364658607
cg13060157		4.34E-05	0.364654389
cg17986181		5.59E-05	0.364634177
cg04712949	CALCR;CALCR	1.50E-05	0.364595352
cg05294112		5.59E-05	0.364592827
cg11618529	ZNF132	1.11E-05	0.364578649
cg00913954	STK40	0.000290372	0.36456145
cg14435109	SGPP2	5.59E-05	0.364555468
cg17327401	CPE	0.000148457	0.364548333
cg01616682	CALCB	0.00043997	0.364539435
cg04932544	SYT14;SYT14;SYT14;SYT14;SYT14;SYT14	4.34E-05	0.364526754
cg15105326	ZIC4;ZIC4;ZIC4;ZIC4;ZIC4	2.58E-05	0.364523088
cg04056384	PCDHB17	0.000233422	0.364522829
cg01574663	HS3ST2	1.50E-05	0.364517393
cg18956481	CYP24A1;CYP24A1;CYP24A1;CYP24A1	0.000537905	0.364514062
cg13480357	CCDC108;CCDC108	2.58E-05	0.36450432
cg22434409	KCNIP4	9.24E-05	0.364496032
cg06102775		8.60E-06	0.36447634
cg17990197	KIAA1755	3.34E-05	0.364474379
cg08858210	TSPYL5	0.002304201	0.364472706
cg14086364	SLC36A3;SLC36A3	5.59E-05	0.364426499
cg05683630		1.50E-05	0.364414691
cg21208104	PRSS12	1.50E-05	0.364409339
cg19586288	RELN;RELN	8.60E-06	0.364396453
cg24089600	COL4A1;COL4A2	0.00043997	0.364389855
cg15405439	EPHA6	1.11E-05	0.364389781
cg19398269		8.60E-06	0.364364987
cg13722700	GPRIN2	0.000233422	0.364357147
cg09747169		0.000117986	0.364331982
cg26136772	FOXK1	3.34E-05	0.364320472
cg02022808	PCDHGA4;PCDHGA2;PCDHGA5;PCDHGB2;PC	0.000537905	0.364310985
cg12782933		0.000233422	0.364309596
cg10943359	MARCH1	1.50E-05	0.364283365
cg05954830		0.000955793	0.36427503
cg05369857		0.000187194	0.364255834
cg16834823	TNXB	0.000357885	0.364243315
cg23395449		4.34E-05	0.364232297
cg00292986	SLC9A2	0.00043997	0.364224228
cg17586860	SSTR4	2.58E-05	0.364217532
cg13923018	PDZD2	3.34E-05	0.364214961
cg17441062	GRM5;GRM5	0.000117986	0.364212346
cg14871932	GCK;GCK;GCK	0.000357885	0.364208181
cg20435474		0.000148457	0.364201208
cg08519575	FAM164A	0.000148457	0.364158813
cg19907776		0.000187194	0.364150077
cg22371972	SLC17A6	0.000233422	0.364146493

cg15400238	RPRM	7.23E-05	0.364128763
cg02076785	CNGA3;CNGA3	0.000233422	0.364127908
cg02865818	WWC1;WWC1;WWC1	3.34E-05	0.364125019
cg22523050	CREB3L2	7.23E-05	0.364123195
cg22797270	NRXN2;NRXN2;NRXN2	8.60E-06	0.364119826
cg13740815	SHANK1	0.000148457	0.364118579
cg14542583	SPRED3	2.58E-05	0.364109551
cg09355771		5.59E-05	0.364101063
cg25771013	MIR148A	2.58E-05	0.364094449
cg23894086		2.58E-05	0.364090537
cg05312353		0.000290372	0.364065501
cg09371047	ZNF214;NLRP14	0.000187194	0.364052207
cg24931242		3.34E-05	0.364046474
cg16656979		0.000117986	0.364017349
cg10739556		1.96E-05	0.364011715
cg07881365		2.58E-05	0.364009662
cg21870668	SNRPN;SNRPN;SNRPN;SNRPN	1.96E-05	0.363996917
cg03966785	PDGFRA	0.000791389	0.363987403
cg25794153		1.11E-05	0.363981633
cg01984802		9.24E-05	0.363981171
cg08089301	HOXB4	0.000233422	0.36397328
cg26717983	RIMS1	0.000148457	0.3639381
cg13464915	CDH2;CDH2	0.000654035	0.363930539
cg03810428		1.50E-05	0.363923666
cg08688551	KCNJ8	0.000654035	0.363921316
cg21825879		0.000117986	0.363913883
cg22328256		1.50E-05	0.363889751
cg18812353	RFX7	4.34E-05	0.363882496
cg18947801	LPPR4;LPPR4	8.60E-06	0.363867659
cg04912999	PAQR9	0.000654035	0.363842492
cg06102330	MSC	2.58E-05	0.363842391
cg16926102		2.58E-05	0.363832814
cg12033822	SLC35C2;SLC35C2;SLC35C2	5.59E-05	0.363829994
cg22624022		0.000187194	0.363810066
cg01855272	EPHA10	0.000537905	0.363790626
cg19351604	ARHGEF10	0.000537905	0.363777112
cg06341054	SPATA18	0.000148457	0.36377063
cg17451941		0.00043997	0.363769745
cg08681924	FEZF2	0.000357885	0.3637691
cg19037350	PCDHGA4;PCDHGA4;PCDHGA2;PCDHGA1;PC	1.96E-05	0.363758329
cg07825294	DAZAP1;DAZAP1	5.59E-05	0.363757978
cg13677149	EVX1	0.000955793	0.36375237
cg06865642	BRD1;LOC90834	3.34E-05	0.36373189
cg04914946	METTL9;IGSF6;IGSF6;METTL9	0.000955793	0.363731219
cg02057782	ATP8A2	1.11E-05	0.363726736
cg04328162	HS6ST3;HS6ST3	1.50E-05	0.363722098
cg24386894	ERI1	3.34E-05	0.363720156
cg15061981	UGT8	8.60E-06	0.363711085
cg20124587	HOXD11	1.50E-05	0.363702354
cg14670974	C5orf52	0.000290372	0.363655466
cg27547442		3.34E-05	0.363644462
cg02165355	ZNF454	9.24E-05	0.363636412
cg22178613	SFRP2	0.000187194	0.363631917
cg08905487	LAIR2;LAIR2	0.002714607	0.363615341
cg01042246	SP5	0.000233422	0.363614344
cg06675190	ACAN;ACAN	5.59E-05	0.363603954
cg21947094	FAM189A1	3.34E-05	0.363587348
cg17457560	NRG1;NRG1;NRG1;NRG1;NRG1;NRG1;NRG1;	0.00043997	0.36358352
cg26612727	ZPBP2;ZPBP2	2.58E-05	0.363578884
cg14430542		1.96E-05	0.363569567

cg11402363	ADCYAP1;ADCYAP1	3.34E-05	0.363540969
cg04089901	COL28A1	0.000117986	0.363528724
cg03869608	PDZRN3	2.58E-05	0.363514207
cg16096172		1.50E-05	0.363498642
cg05342515		4.34E-05	0.363498178
cg02305723	SNRPN;SNRPN;SNRPN	8.60E-06	0.363486939
cg15669183	FAM155A	9.24E-05	0.363451685
cg07437373		5.59E-05	0.363441399
cg11168614		0.000233422	0.36342987
cg13279673		7.23E-05	0.363422337
cg02721902		3.34E-05	0.363408239
cg11302943	CRTAC1	9.24E-05	0.363398544
cg19384289	HOXD8	0.000148457	0.36338729
cg12798118	PMP22;PMP22	1.50E-05	0.3633866
cg21729992		9.24E-05	0.36336771
cg01511356		1.96E-05	0.363360252
cg21632975	NOVA2	1.96E-05	0.363358917
cg09020199	TRIM39;TRIM39	1.96E-05	0.363352525
cg14142283	ISL1	8.60E-06	0.363351169
cg10730174	PRRX1;PRRX1	0.000148457	0.363342985
cg17019053	C2orf65	3.34E-05	0.363329953
cg04464276	SMPD3	0.000187194	0.363310853
cg15586392	SLC45A4	0.000148457	0.363307216
cg17932662	PTPN7;PTPN7	4.34E-05	0.363302272
cg00127167	ADAMTS16	3.34E-05	0.363292238
cg07905808	TRIM39;TRIM39	1.96E-05	0.363278679
cg18049638		4.34E-05	0.36326887
cg10710218	ACOT11;ACOT11	8.60E-06	0.363245608
cg18189994		2.58E-05	0.3632414
cg07283114	LOC401463	0.000187194	0.363193744
cg10941566	CABP7	0.00043997	0.363186556
cg00824767		0.000148457	0.363185268
cg26149275	EVX2	7.23E-05	0.363178807
cg23125492	PAX6;PAX6;PAX6	2.58E-05	0.363165475
cg02907098	ANO2	1.11E-05	0.363158016
cg08665243	PCDHB16	4.34E-05	0.363154571
cg14504259		5.59E-05	0.363141697
cg19871394	TMEM170B	8.60E-06	0.363096761
cg25061843		1.96E-05	0.363088058
cg14081218	CTNND2;CTNND2	1.96E-05	0.363064182
cg08318726	TBX5;TBX5;TBX5	0.000537905	0.363059113
cg05044185	GABRA5;GABRA5;GABRA5	7.23E-05	0.363048259
cg22906273	PAX1	4.34E-05	0.363046313
cg02386403	FOXF2	8.60E-06	0.363037147
cg00361495	TRIL	0.001948248	0.363031334
cg17763354	RGR;RGR;RGR	1.96E-05	0.363030683
cg00157199		0.000357885	0.363026212
cg13077519	HTR1B	2.58E-05	0.363012647
cg05374412	CDH13	0.000187194	0.362995531
cg16967583	AGXT	1.50E-05	0.362989073
cg15829088		2.58E-05	0.362982685
cg26708220	MEIS2;MEIS2;MEIS2;MEIS2;MEIS2;MEIS2;MEI	0.000357885	0.362976245
cg21500166	KCNC2;KCNC2;KCNC2;KCNC2;KCNC2;KCNC2	2.58E-05	0.362974488
cg20414996	ANKRD34B;ANKRD34B	1.96E-05	0.362972923
cg27304110	SYT6	0.000357885	0.36295656
cg11471772	SIM1	0.000654035	0.362947528
cg13950829	CUGBP2;CUGBP2	2.58E-05	0.362946236
cg04527363	GRM7;GRM7	2.58E-05	0.362941141
cg15311814		0.00043997	0.362940589
cg16151977	OCLN	5.59E-05	0.362929895

cg11800620	ESRRG	9.24E-05	0.362923164
cg01500945	SALL1;SALL1	0.001147425	0.362922102
cg24116870	ADAMTS20	1.50E-05	0.362897511
cg02227879	CAMK2G;CAMK2G;CAMK2G;CAMK2G;CAMK2	0.002714607	0.362890803
cg10425861		5.59E-05	0.362883559
cg10711209	NPHS2	7.23E-05	0.362882511
cg04452311		2.58E-05	0.362882253
cg23173688		8.60E-06	0.362866187
cg17943279		4.34E-05	0.362860631
cg07688988	ACOXL;ACOXL;ACOXL;ACOXL;ACOXL;ACOXL	3.34E-05	0.36284276
cg25730685		0.000187194	0.362840017
cg04079248	ARL4A;ARL4A;ARL4A	0.004131428	0.362835064
cg14543285	RCOR1	8.60E-06	0.362833828
cg11679177	RGS7	0.000654035	0.362818403
cg16652303		0.000117986	0.362816424
cg26059468	SOX17;SOX17	0.000537905	0.362815389
cg16943529	EOMES	0.000148457	0.362808107
cg00478884	RNASE10	5.59E-05	0.362786143
cg02861504	FCAMR;FCAMR;FCAMR;FCAMR	0.000148457	0.362782038
cg03833880		3.34E-05	0.362777571
cg20673829	VIPR2	0.0050758	0.362767062
cg11997899	DLX5	0.002714607	0.362765015
cg01410359	CYP1B1	8.60E-06	0.362757274
cg04264299	SCARA3;SCARA3	0.007873393	0.362737962
cg20896728		8.60E-06	0.362718895
cg01717727	ADAMTS16	4.34E-05	0.362713792
cg07274716	PITX1	0.000117986	0.362713298
cg17560332	BOLL;BOLL	1.11E-05	0.362700458
cg23634124	DLX4;DLX4	0.000117986	0.362691426
cg01373292	CAMTA1	0.004357816	0.362686902
cg04205107	PLD5	0.000233422	0.362673378
cg13302823	SCRT1	1.50E-05	0.362641402
cg11841771		7.23E-05	0.362624728
cg21327694	CCK	0.000290372	0.362615545
cg13529755		5.59E-05	0.362568584
cg14138235		0.007873393	0.362546457
cg08730245	NPFF	2.58E-05	0.362543197
cg02031245	CSMD2	0.000187194	0.362527121
cg22968936	PAX9	5.59E-05	0.362496483
cg19443257	SPSB4	0.003734675	0.36248567
cg17331920	ANKFY1	3.34E-05	0.362475491
cg03880355	FAM169A	0.000233422	0.362473687
cg09848789	MCOLN2	0.000791389	0.362437052
cg05255275	C14orf39	7.23E-05	0.362433631
cg15698842	CDH2	7.23E-05	0.362411759
cg15450160	POP1;POP1;HRSP12;POP1	0.000654035	0.362408087
cg01664666	PLD5	0.000233422	0.362407382
cg23253569	OLIG2	0.000148457	0.362406231
cg12730468		0.000233422	0.362389167
cg03774463	DLX6AS;DLX6	0.000955793	0.36237169
cg25033364	KCNC1;KCNC1	0.000117986	0.362357164
cg08183317	TFDP1;TFDP1	0.001147425	0.362353359
cg22692549		5.59E-05	0.362344031
cg03088791	PLCE1	5.59E-05	0.36233629
cg09535924	MEIS1	0.000233422	0.36229283
cg13696609	FOXE1;FOXE1	1.50E-05	0.362289008
cg25344503	PPYR1	0.000233422	0.362286267
cg11854981	TRERF1	0.000290372	0.362278182
cg01676182		1.11E-05	0.362272206
cg18882407	MAP1LC3C	3.34E-05	0.362266401

cg13266435	ANKRD30B	0.002714607	0.362239746
cg14741153	PLCE1	0.000117986	0.362231645
cg24101578	CDH22;CDH22	8.60E-06	0.362229736
cg25535027	SLC39A7;SLC39A7;RXRB;SLC39A7	2.58E-05	0.362222086
cg10937591	KCND3;KCND3	5.59E-05	0.362220037
cg25773262	BCAN;BCAN	9.24E-05	0.362209831
cg24881834	ME1	1.50E-05	0.362186053
cg01204927		7.23E-05	0.362173589
cg07126263	CCBE1	0.000187194	0.362136657
cg15928093	FZD10;FZD10	0.000654035	0.362134822
cg21495349	C7orf50;C7orf50;C7orf50	4.34E-05	0.362097749
cg14325153	PRR23C	8.60E-06	0.362084946
cg07967679	KRT7	3.34E-05	0.362068129
cg23614791	ACSS3	8.60E-06	0.362052944
cg21726079		0.000148457	0.362015754
cg08376864	MGC2889;HRASLS	5.59E-05	0.362015257
cg00266322	FADS6	0.000654035	0.362013643
cg00347369	STMN2	1.11E-05	0.362003967
cg18095675	BDH1;BDH1;BDH1	1.11E-05	0.362001711
cg03213352	KCNC3	5.59E-05	0.361994002
cg10440877		2.58E-05	0.361987974
cg16792302	MAP1D	0.008944659	0.36196541
cg16759888	CYP39A1;SLC25A27	0.000233422	0.361964686
cg08016383	ADAMTS17	0.001376172	0.361964642
cg03989260	TBX15	8.60E-06	0.361948723
cg19040483	DLC1;DLC1	1.50E-05	0.361930343
cg10040836	NR2F2;NR2F2;NR2F2;MIR1469;NR2F2	7.23E-05	0.361893489
cg15167202	SYNPO;SYNPO	7.23E-05	0.361887805
cg10397678		3.34E-05	0.36188543
cg07959771		1.96E-05	0.361875908
cg20532999	MIR548H4;NOX5	1.96E-05	0.361872746
cg13951074		0.000117986	0.361872322
cg08558873	ZNF214;NLRP14;ZNF214	8.60E-06	0.36186372
cg00193200	STXBP5L	9.24E-05	0.361836796
cg17695462	DPYSL4	2.58E-05	0.361835439
cg19793697	RTBDN	0.000187194	0.361804588
cg13412550	ASTN2;ASTN2;ASTN2	1.96E-05	0.361794963
cg11932158	PLCH1	9.24E-05	0.361791947
cg22274234	KCTD10	1.96E-05	0.361745752
cg08815081	MKX	7.23E-05	0.361738881
cg20629239	DRD2;DRD2	9.24E-05	0.361738511
cg14016177		0.000791389	0.361722838
cg27010159	SRRM4	0.000654035	0.361717965
cg06563089	KCNC2;KCNC2;KCNC2	8.60E-06	0.361694997
cg27569300	SYNM;SYNM	0.002714607	0.361681037
cg06609197	STMN2	1.96E-05	0.361679637
cg16238918	PARD6G	0.000233422	0.361652911
cg03191889	PARM1	0.006823935	0.361651239
cg10272601	WNK2	0.001948248	0.36164908
cg14291919	C11orf90	0.000654035	0.361636673
cg15048991	ADAMTS16	0.000357885	0.361626974
cg06899044		4.34E-05	0.361624777
cg11576834	OGDHL;OGDHL;OGDHL	7.23E-05	0.361620789
cg22231400	SLC17A7	0.000537905	0.361610507
cg09948350	ASPRV1;ASPRV1	1.96E-05	0.361606457
cg24955204		4.34E-05	0.361570037
cg01688936	C16orf93	1.11E-05	0.361563701
cg24454932		0.001147425	0.361552639
cg25724201	PKIB;PKIB;PKIB	9.24E-05	0.361530018
cg16498194	FZD10	3.34E-05	0.361492857

cg24595580	FLJ42289;FLJ42289	0.000791389	0.361478037
cg05052450		9.24E-05	0.361473478
cg01013590	SCTR	5.59E-05	0.361459186
cg22333960	IGSF21	0.000148457	0.361444691
cg02701625	VWC2L	7.23E-05	0.361397582
cg14066298	TRIM39;TRIM39	5.59E-05	0.361397553
cg07078225		0.000290372	0.361385584
cg04408595	EIF4E3;EIF4E3;GPR27	9.24E-05	0.361375314
cg00642749	SEMA5A	0.00043997	0.361366928
cg05542262	RASGRF2	0.000537905	0.361366502
cg06654079	DNAH3;TMEM159	7.23E-05	0.36134905
cg13788515	ZDBF2	0.000148457	0.361337081
cg24271863		1.11E-05	0.361334757
cg16199881	FAM149A	0.000187194	0.361325917
cg23208590	CARD10	7.23E-05	0.361325577
cg14024242		3.34E-05	0.361298503
cg27438889	TACC2;TACC2;TACC2;TACC2	4.34E-05	0.361293829
cg12050497	FAM84A	8.60E-06	0.361292626
cg05457273	KCNN2	9.24E-05	0.361278925
cg24642320		9.24E-05	0.361245322
cg12215739	HOXD4;MIR10B	7.23E-05	0.361242072
cg08182501		3.21E-05	0.361234998
cg14377681	ERBB2;ERBB2	5.59E-05	0.361229432
cg05201477	ARNT2	0.000117986	0.361225551
cg27388911	THRB;THRB;THRB	0.000187194	0.361206856
cg25367558	USP49	4.34E-05	0.361202443
cg24634420	PDGFC	0.001393182	0.361201486
cg11948341	DHCR24	1.96E-05	0.361186695
cg04178316	TLX3	8.60E-06	0.36118525
cg24222995	TRIL	0.000357885	0.361183115
cg20718350	ASCL1	0.000654035	0.361168779
cg09123368	VAX1;VAX1	1.50E-05	0.361158638
cg16777618		0.000117986	0.361158439
cg01287514		0.000117986	0.361156724
cg20784768	SLC35F3	8.60E-06	0.361139303
cg09086201	TFAP2D	0.000148457	0.36113808
cg12755298	CYP7B1	0.00043997	0.361125461
cg24087681		0.000955793	0.361117014
cg25017620		0.000187194	0.361114882
cg22752533	SLC12A5;SLC12A5;SLC12A5	2.58E-05	0.361114249
cg21635203	SOX2;SOX2OT;SOX2	7.23E-05	0.361111868
cg16708981	ZNF677	3.34E-05	0.361085294
cg23883393	FGF14	7.23E-05	0.361083615
cg14210726	TMC8	4.34E-05	0.361070257
cg00539976	IRX4	2.58E-05	0.361069143
cg02736420	SOX21	0.000955793	0.361058626
cg10959668		1.96E-05	0.361020707
cg21521683	ST8SIA1	0.000357885	0.361010317
cg11732619	SLIT3;SLIT3	7.23E-05	0.361010141
cg05996795	MGC34034;MGC34034	0.000357885	0.36100761
cg12836863	BRCA2	0.000233422	0.361001878
cg24510700	ENPP5	1.50E-05	0.360992262
cg17881660	SLC22A15;SLC22A15	0.002304201	0.360991215
cg12235459		1.96E-05	0.360990047
cg16786178		0.000290372	0.360978314
cg18675097	NKAPL;NKAPL	8.60E-06	0.360974174
cg17886678	UACA	5.59E-05	0.360965057
cg00230502	CCDC60	9.24E-05	0.360950335
cg03399905	ANKRD34C	4.34E-05	0.360944858
cg14883916	ZNF215	7.23E-05	0.360911728

cg20403938	HOXC10	0.000537905	0.36089828
cg17730484	DTX3	0.000233422	0.36089577
cg24725576		0.000187194	0.360894055
cg05674150	BNC2	0.000955793	0.360888687
cg14584448	PLA2R1;PLA2R1	0.000148457	0.360888383
cg09112081		0.000148457	0.360887932
cg04786857	SPDYA;SPDYA	0.000117986	0.360865966
cg21640587	DSCAML1	0.00041966	0.360862765
cg05975727	KIF5C	3.34E-05	0.360842969
cg12279419		8.60E-06	0.360830109
cg26755141	SIM1	0.000148457	0.360829474
cg09746736	SLC6A2	0.000148457	0.360821581
cg20670923	VAPA;VAPA	0.002304201	0.360805213
cg16987900	PCDHA2;PCDHA1;PCDHA1;PCDHA6;PCDHA5	4.34E-05	0.360801805
cg06854666	LIMCH1;LIMCH1;LIMCH1	0.004131428	0.360789395
cg00206356		8.60E-06	0.360782821
cg14514569		1.50E-05	0.360777112
cg20814202	AATK	0.000357885	0.360775561
cg04602284	C3orf15	2.58E-05	0.360760888
cg02525108		3.34E-05	0.360737592
cg01882870	MIR9-3	1.96E-05	0.360726239
cg08390877		0.000187194	0.360680898
cg17838734	TPBG;TPBG	0.002714607	0.360680786
cg03092191	UNCX	9.24E-05	0.360671976
cg09685182		4.34E-05	0.360666274
cg01423335	GPC6	0.000148457	0.360652839
cg18546689	TNFSF11;TNFSF11;TNFSF11	0.001948248	0.360651791
cg19935471	MATN2;MATN2	1.96E-05	0.360647008
cg23302603	KIF1A	0.000148457	0.360634406
cg09169255	SPR	0.001376172	0.360628599
cg24042339	MYO5B	8.60E-06	0.360595871
cg25767345		4.34E-05	0.360590813
cg14958635	NEUROG1	9.24E-05	0.360585429
cg07895169	TTC23L	0.000117986	0.360580793
cg01274643		0.000233422	0.360572614
cg09159022	CSMD1	0.000148457	0.360562821
cg06481122		9.24E-05	0.360560951
cg19290410	TBX5;TBX5	1.50E-05	0.360558386
cg11125805	SLC36A3;SLC36A3	0.001948248	0.360542042
cg03572260	STARD3;STARD3;STARD3	8.60E-06	0.360541319
cg08213337	PDE11A;PDE11A	0.000537905	0.360540039
cg21464115	ESYT3	8.60E-06	0.360536626
cg13300301	FUT4;FUT4	0.000791389	0.360516446
cg13817046		0.00043997	0.360514967
cg21469278	GRB14	4.34E-05	0.360510788
cg11430245	SV2C	0.000537905	0.360503815
cg17806623	KL	0.002266401	0.360479967
cg14750277		1.96E-05	0.360458717
cg21916108	ZFPM2	0.000148457	0.360457695
cg25015038		0.000158823	0.360454848
cg13607230	MFSD9	2.58E-05	0.36044309
cg04978078		0.000187194	0.360433519
cg23817096		2.58E-05	0.360430206
cg10539861	ENTPD7	5.59E-05	0.360426669
cg11176493	WNT3A	9.24E-05	0.360386266
cg14898416	PLA2R1;PLA2R1	0.003189589	0.360385676
cg06446668	FAM155A	5.59E-05	0.360382036
cg03027179		1.96E-05	0.360359505
cg13631916	FAM162B	7.23E-05	0.360351526
cg10726475	WWTR1;WWTR1;WWTR1	7.23E-05	0.360330954

cg00371453	CLEC4GP1	0.000357885	0.36033016
cg13634602	SALL3	2.58E-05	0.360320198
cg20488280	GPR126;GPR126;GPR126;GPR126	0.000117986	0.360294529
cg13455410		7.23E-05	0.360289824
cg13962664		8.60E-06	0.360283229
cg06633061		1.96E-05	0.360266998
cg04059773		4.34E-05	0.360260506
cg14836555		9.24E-05	0.360221013
cg18650670	IGDCC4	7.23E-05	0.360212769
cg07282889		7.23E-05	0.360194978
cg00301660		1.11E-05	0.36017444
cg11770663		0.00043997	0.360171206
cg04011228	RPRM;RPRM	2.58E-05	0.360170781
cg06792358	NRSN1	3.34E-05	0.360164237
cg22070406	ST8SIA3	3.34E-05	0.360160879
cg04349243	SLC16A12	0.000357885	0.360137042
cg08289140		0.000233422	0.36012726
cg24794531	TRPC1	5.59E-05	0.360096117
cg10446179	GJA1	1.96E-05	0.360092135
cg07300060		4.34E-05	0.360078492
cg07110659	LPAR3	5.59E-05	0.360072411
cg08286738	CYP26B1	0.000187194	0.360066241
cg08053904	GPR139	0.000187194	0.360047059
cg09009074	LMCD1	0.000357885	0.360035513
cg13627491	LPPR1	0.000233422	0.360035324
cg21189054	NCAM1;NCAM1;NCAM1	4.34E-05	0.360034981
cg14600987	TNNT3;TNNT3;TNNT3;TNNT3	0.000117986	0.360031473
cg08832624		3.34E-05	0.360028894
cg06922606	GRIN2A;GRIN2A;GRIN2A	1.11E-05	0.360020382
cg17745697		0.000290372	0.360005839
cg02920216	CHODL	8.60E-06	0.359997483
cg08092930	PPFIA1;PPFIA1	0.001639597	0.359997265
cg14700304		0.000955793	0.359985965
cg18161327		0.000187194	0.359971512
cg12355180	SLC30A10	3.34E-05	0.359970887
cg26956874	RYR2	3.34E-05	0.359965889
cg04009492	MAL2	1.50E-05	0.359963102
cg13935437	AKR7L;AKR7L	0.000117986	0.359958864
cg00005619	FAM180B	1.96E-05	0.359924793
cg02012576		0.000791389	0.359921833
cg22047703	BMP7	0.000290372	0.359909349
cg07643225	C10orf107	5.59E-05	0.359905069
cg06212175	A2BP1;A2BP1;A2BP1	3.34E-05	0.359893194
cg25484990	FOXA2;FOXA2	7.23E-05	0.359885332
cg09505788		7.23E-05	0.359882274
cg24769846		2.58E-05	0.359876438
cg24213719		0.001639597	0.359869363
cg10498524	PDE11A;PDE11A	0.000290372	0.359848081
cg17647904	NCOR2;NCOR2	2.58E-05	0.359844175
cg24666702		0.000187194	0.359821186
cg21233517	ACOT4	7.23E-05	0.35981592
cg02423064	ACCN1	7.23E-05	0.359789211
cg17788682	ARNT2	0.000187194	0.359774511
cg18646207	VAX1;VAX1	4.34E-05	0.359774329
cg12164321	CPXM2	2.58E-05	0.359765374
cg11915650	INS-IGF2;IGF2AS;IGF2;IGF2;IGF2AS;IGF2	0.000187194	0.359764382
cg15381098	AP3B2	2.58E-05	0.359760712
cg15692992		4.34E-05	0.359755628
cg20219035	PLBD1	7.23E-05	0.359754602
cg25366582		0.000537905	0.359748325

cg00272200	FGF10	7.23E-05	0.359733316
cg17173498	FLJ42875;PRDM16;PRDM16;FLJ42875	0.000187194	0.35972779
cg05124441	SYN2;SYN2;SYN2;SYN2	7.23E-05	0.359726909
cg02741229	PCDHGA4;PCDHGA6;PCDHGA1;PCDHGA5;PC	1.96E-05	0.359720923
cg03970319	UNCX	8.60E-06	0.359712501
cg23098529	PPAN;PPAN-P2RY11	0.000187194	0.359689603
cg27029821	PRDM14	2.58E-05	0.359686465
cg00472277	TSPAN32	0.006823935	0.359674157
cg19880942	C8orf34	4.34E-05	0.359673383
cg24413662		5.59E-05	0.359672609
cg21907579	TBX5;TBX5;TBX5;TBX5	2.58E-05	0.359665695
cg04250847	PPFIA2	1.50E-05	0.359663372
cg11717564	RXRG;RXRG	0.000290372	0.359658193
cg05126887	COL21A1	1.50E-05	0.359639229
cg12530503	MIR9-3	1.96E-05	0.359636284
cg00662556	GALR1	8.60E-06	0.359622979
cg08106973		0.001948248	0.359587545
cg12782180	LEP	4.34E-05	0.359579005
cg21549294		2.58E-05	0.359569225
cg04056343	A2BP1;A2BP1;A2BP1	4.34E-05	0.35954383
cg22631938	EIF4E3;EIF4E3;GPR27	1.50E-05	0.359542152
cg23906261	GFRA1;GFRA1;GFRA1	9.24E-05	0.359521559
cg21912162	MAP1B	3.34E-05	0.359514319
cg12043818		0.000187194	0.359509321
cg10947408		4.34E-05	0.359501224
cg04330449	NEUROG1	9.24E-05	0.359491671
cg25035988		0.000526175	0.359491188
cg27467803		0.000205664	0.359474368
cg24394856		0.000357885	0.359441064
cg11692124		0.002714607	0.359438192
cg23458558	RALYL;RALYL;RALYL;RALYL	1.11E-05	0.359437024
cg20128928	TACR3	3.34E-05	0.359416701
cg20008101	LGALS3	0.00043997	0.359380935
cg20386487		0.000537905	0.359363331
cg25105522	MAP3K14	0.00043997	0.359360115
cg12986271	TSHZ3	0.004357816	0.359350866
cg07892597	TBX15;TBX15	1.96E-05	0.359338958
cg05048377		0.000357885	0.359331873
cg04570735	PDE4C;PDE4C;PDE4C	0.000955793	0.359327831
cg10056132	C10orf53;C10orf53;C10orf53;C10orf53	0.000187194	0.359324563
cg21907107	OTP	0.000148457	0.359279717
cg18987410	HEPACAM	0.000233422	0.35924454
cg11385338		9.24E-05	0.359242396
cg19011001	ITPK1;ITPK1;ITPK1	2.58E-05	0.359236473
cg22930650	CWH43	0.00156564	0.359229247
cg21088438	SLC10A1	0.000357885	0.359219354
cg08944026	ANKRD33B	0.000537905	0.359212725
cg15281774	HCN4	0.000233422	0.359209997
cg21215899	LOX	3.34E-05	0.35920666
cg20806175	FCER1G	0.000290372	0.359195267
cg03002352	RAD51AP2;RAD51AP2	2.58E-05	0.359184321
cg05825420	EMX2OS;EMX2;EMX2	8.60E-06	0.359183195
cg03759824		1.96E-05	0.35918221
cg03698948	DAAM2	0.000955793	0.359167808
cg14466269	SLC25A21;SLC25A21;LOC100129794	1.11E-05	0.359164288
cg21235151		0.00043997	0.359157256
cg26262840	C14orf102;C14orf102	5.59E-05	0.359154
cg20540566	TTPA	0.000262362	0.359132464
cg22324022	NRXN1;NRXN1	2.58E-05	0.359120367
cg12833465	SFTA3	0.000117986	0.359096969

cg14747563	NEUROG1	0.000148457	0.359071134
cg23244488		0.001147425	0.359057789
cg05060607	C3AR1	0.000148457	0.359057142
cg27027827		2.58E-05	0.359032727
cg25436886		3.34E-05	0.359032701
cg26508200	SSH1;SSH1	0.000357885	0.359015024
cg22091110		7.23E-05	0.359011156
cg27047406	SLC6A2	4.34E-05	0.359008359
cg07513791		0.000187194	0.358999848
cg14012546	INPP5D;INPP5D	0.000233422	0.358991899
cg00606739	LOC440040	1.50E-05	0.358974978
cg08247450	AADAT;AADAT;AADAT	4.34E-05	0.358953073
cg18349835	VIPR2	0.003734675	0.358930206
cg25509184	CFTR	9.24E-05	0.358929623
cg24396691	SLC30A10	2.58E-05	0.358884039
cg20870512	UNCX	0.001061731	0.358882339
cg12057242	CPSF3	3.34E-05	0.358856214
cg27648946	RHOBTB1;RHOBTB1	8.60E-06	0.358852719
cg03595533		2.80E-05	0.35884874
cg08039116	HECW1;HECW1	5.59E-05	0.358825576
cg05998607		3.34E-05	0.358825355
cg10643691		4.34E-05	0.35880409
cg26731008	FAM24A	2.58E-05	0.358800947
cg08870743	OLIG2	0.000148457	0.35878222
cg18569734	NR2E1	1.96E-05	0.358780294
cg20576393	LMO3;LMO3	8.60E-06	0.35877332
cg11258943	FOXE1	0.000290372	0.35872945
cg12864721	C10orf41;C10orf41	9.24E-05	0.358726386
cg24690231	IGSF21	9.24E-05	0.358722895
cg17010895		0.000117986	0.358709369
cg25806701	LRAT	0.000233422	0.358704482
cg05036656		7.23E-05	0.35869886
cg14312649		0.000148457	0.358689431
cg15623175		1.50E-05	0.358665357
cg06535308	ABCC8	1.50E-05	0.358655341
cg02745847	IGF2BP1;IGF2BP1	9.24E-05	0.358653352
cg18934187	STARD6	1.11E-05	0.358646173
cg04226724	LPHN2	0.000233422	0.358636117
cg18687675	GSC	0.003734675	0.358623842
cg16973520		7.23E-05	0.358619853
cg26843711	PCDHGA4;PCDHGA6;PCDHGA1;PCDHGA5;PC	5.59E-05	0.358616012
cg14348439	LONRF2	0.000233422	0.358615207
cg00450784	RFX4	2.58E-05	0.358612551
cg15208375	TSHZ3	0.000654035	0.358592792
cg03160883		0.001639597	0.358592483
cg19137348		0.000290372	0.358588398
cg22967396	OTX2OS1	8.60E-06	0.358577292
cg00701692	NKX6-2	1.50E-05	0.358571882
cg02743222	TLX3;TLX3	0.000233422	0.358537847
cg27138901	CLEC2D;CLEC2D	0.00043997	0.35852805
cg11758945		0.000233422	0.358519924
cg22115076		2.58E-05	0.35851642
cg08920032	IGF1R	9.24E-05	0.358508429
cg24258699	MSC	0.000233422	0.358500239
cg02611848	C2orf65	0.000233422	0.358482927
cg01566555		0.000537905	0.358462546
cg02146929	PDE10A;PDE10A	0.000187194	0.35843836
cg02268748		0.000290372	0.3584357
cg04123776		7.23E-05	0.358427326
cg00564163	STEAP4	0.000290372	0.358410539

cg16204509	ALDH1L2;ALDH1L2	4.34E-05	0.358406797
cg00879003		1.96E-05	0.358387742
cg10416963	PRDM5	2.58E-05	0.358382399
cg05330360	ZPBP2;ZPBP2	3.34E-05	0.358377157
cg02896696	DHCR24	2.58E-05	0.358343168
cg22736850	OVOL2	7.23E-05	0.358327713
cg23018092	MEGF10	0.002714607	0.358325555
cg06734406	AQP2	0.000357885	0.358312855
cg09165842	PCSK2	0.000187194	0.358312714
cg22884656		2.58E-05	0.358312122
cg01232748	CHIC2	7.23E-05	0.358282281
cg02819231	FBXL2	0.000290372	0.358267009
cg26517584	SBNO2	2.58E-05	0.358238694
cg20611911	TP73;WDR8	0.001061731	0.358209203
cg03898631		1.50E-05	0.358207648
cg02175321	PDGFD;PDGFD	4.34E-05	0.358196073
cg05344747	CD59;CD59;CD59;CD59	0.001147425	0.358183194
cg16053580	KLK13	0.000290372	0.358175173
cg01926238		5.59E-05	0.358171833
cg06159896		1.50E-05	0.358160559
cg03371199	OXGR1	0.000148457	0.358145043
cg11825621	MKX	0.000148457	0.358141363
cg04605532		0.000148457	0.358124017
cg05457768		1.96E-05	0.358074911
cg01777397	CLIP4	0.002304201	0.358065651
cg01033463	PHF21B;PHF21B	0.000290372	0.358048091
cg07416237	CLYBL	0.000233422	0.358047965
cg04920358		0.000654035	0.358034523
cg22894805	MGA;MGA;MIR626	7.23E-05	0.358024173
cg11970349	GPR78	2.58E-05	0.358000789
cg09235539	DNAJC6;DNAJC6	1.96E-05	0.357998415
cg08534628		8.60E-06	0.357991222
cg04898695	CHD5	0.000537905	0.357988091
cg20312205	SLITRK1;SLITRK1	0.000290372	0.357987599
cg14639897	MATN2;MATN2	5.59E-05	0.357980946
cg14834925		7.23E-05	0.357977067
cg05364759	ALPL;ALPL	0.000357885	0.357971712
cg09722742	SPOCK3;SPOCK3;SPOCK3;SPOCK3	0.001639597	0.357962502
cg11058942		0.000290372	0.357962351
cg12152566	FAM193B;FAM193B	9.24E-05	0.357951319
cg26809635		0.000654035	0.357928025
cg07161721		4.34E-05	0.357924262
cg07357987	SGPP2	0.000187194	0.357916544
cg05822532	ELN;ELN;ELN;ELN;ELN	0.000365373	0.357906443
cg00792513		1.50E-05	0.357896007
cg05952572	PDE2A;PDE2A;PDE2A;PDE2A	8.60E-06	0.357889522
cg18315963	PCDH7;PCDH7;PCDH7	5.59E-05	0.357882695
cg13995854	ST7OT2;ST7;ST7	0.000537905	0.357848406
cg23277376	PLA2G7;PLA2G7	0.000117986	0.357830142
cg16182986	MARCH11	4.34E-05	0.357822
cg06851941	FBXL2	0.000187194	0.357818201
cg24277715		0.001147425	0.357758804
cg11028872		2.58E-05	0.357756261
cg10615842		3.34E-05	0.357743384
cg21946299	PPARG;PPARG;PPARG	9.24E-05	0.357733876
cg04461802	GPR126;GPR126;GPR126;GPR126;GPR126;G	5.59E-05	0.357707346
cg08571883		0.000117986	0.357704447
cg08214808	MAPK8IP1	0.000955793	0.357697109
cg09434193	HBA1	0.000148457	0.357695645
cg04425005	ZNF827	1.96E-05	0.35765939

cg26052635	CLIP4	0.006823935	0.357647775
cg14933494		4.34E-05	0.357630377
cg22320365	SRCIN1	0.000117986	0.357624659
cg24433287	MANEAL;MANEAL;MANEAL	7.23E-05	0.357604161
cg26153410		0.000117986	0.357601084
cg14993491	PCSK9	1.50E-05	0.357584588
cg19016332	NPHS2	1.11E-05	0.357583996
cg00336320	PNPLA1;PNPLA1;PNPLA1	7.23E-05	0.357581603
cg15907944		9.24E-05	0.357548352
cg10135260		0.000357885	0.357546149
cg13802457	GLIS3	1.11E-05	0.357533421
cg01764020	TRPC4;TRPC4;TRPC4;TRPC4;TRPC4;TRPC4	0.000117986	0.357508919
cg01363714		0.000233422	0.357508096
cg13303534		1.96E-05	0.357495178
cg07877559	CTNND2	0.000148457	0.357480531
cg19075787		5.59E-05	0.357461677
cg19384905	FBXO5;FBXO5	0.001639597	0.357459005
cg13927387	YAP1;YAP1	0.000233422	0.3574577
cg03259243		1.50E-05	0.35745538
cg27111678	CHST8;CHST8;CHST8	2.58E-05	0.357448048
cg05666120	EGR4	0.000233422	0.357446487
cg07467520	ROBO1;ROBO1;ROBO1;ROBO1;ROBO1;ROBO	5.59E-05	0.357444392
cg06580371	PKIB;PKIB;PKIB	0.000148457	0.357433788
cg15885337	OPCML;OPCML	0.000654035	0.357422038
cg00653387	PTN;PTN	1.11E-05	0.357416983
cg11014124	PCDHGA4;PCDHGA6;PCDHGA1;PCDHGA8;PC	0.00043997	0.35741452
cg17974780	CLDN3	0.000117986	0.357414376
cg02969141	MEOX2	0.004357816	0.357378106
cg26029736		0.000187194	0.357376745
cg20762480		8.60E-06	0.357335127
cg02356551		0.002714607	0.357330026
cg20183802	PIP4K2C;PIP4K2C;PIP4K2C;PIP4K2C	9.24E-05	0.357326067
cg05049361	GALNTL1;GALNTL1	0.001147425	0.357323402
cg23906060	ANKRD34C	1.50E-05	0.357292054
cg12353688		0.000290372	0.357291347
cg16857801	LOC440040	2.58E-05	0.357265544
cg25842285	CNNM1	0.000187194	0.357260604
cg07831417		1.50E-05	0.357259248
cg04748988	PPARG;PPARG;PPARG	0.000791389	0.357249535
cg20269359	C21orf88;C21orf88	4.34E-05	0.357213015
cg18806378		4.34E-05	0.357201333
cg12539415	HOXD4;HOXD4	0.000117986	0.357190171
cg18729308	AADAT;AADAT	3.34E-05	0.357164756
cg13732582	KLB	0.009066563	0.357162616
cg03237606	TMEM26	3.34E-05	0.357133441
cg24042242	C1QL1	0.000474381	0.357132483
cg02243785	LPHN2	0.000187194	0.357128375
cg18804985	CXCL2	0.000654035	0.357123162
cg18788994		5.59E-05	0.357119933
cg12581627	PCDH20	5.59E-05	0.357118439
cg05630016	IGLON5	4.34E-05	0.357109848
cg24427504		0.001147425	0.357107485
cg21135135	WBSCR17	0.000233422	0.357074638
cg15775921	PAX3;PAX3;PAX3;PAX3;PAX3;PAX3	1.50E-05	0.357070579
cg00459623		0.000233422	0.357067471
cg16341836	STAMBPL1	0.000290372	0.357060455
cg02508651		0.000117986	0.357059236
cg16512661		0.000117986	0.357042518
cg14472366		0.001639597	0.357037851
cg21501724	PAK7;PAK7	2.58E-05	0.357037232

cg07988820	PPFIA2	2.58E-05	0.357035818
cg09555736	RAB11FIP3	2.58E-05	0.357026321
cg14399851	HLF;HLF	0.000117986	0.35701849
cg16954385		0.000654035	0.357008315
cg22376706	PTPRR	7.23E-05	0.357007295
cg25174591	DNAH9	8.60E-06	0.35700157
cg02570079		3.34E-05	0.356995644
cg08927145		0.000233422	0.356982183
cg25732028		1.50E-05	0.356969338
cg06492708	SASH1	1.50E-05	0.356968736
cg18483908		0.000654035	0.356948968
cg02840025		1.96E-05	0.35694821
cg14611767		4.34E-05	0.356941187
cg10129391		0.00043997	0.356924885
cg23422268		4.34E-05	0.356924501
cg01634458		5.59E-05	0.356904185
cg24924936	PTGFR;PTGFR	0.000148457	0.356900958
cg14628914	TTRAP	2.58E-05	0.356893268
cg26074603	KCNC2;KCNC2;KCNC2;KCNC2;KCNC2;KCNC2	0.000791389	0.356892603
cg14803765	CREB5;CREB5	0.001147425	0.356892551
cg07008213	WWC2;C4orf38	4.34E-05	0.356824654
cg08887961	RHOBTB1;RHOBTB1	1.50E-05	0.356814459
cg25689193		9.24E-05	0.356813048
cg04131583		0.000148457	0.356792455
cg12602409		0.005897668	0.356784194
cg05875410		0.000187194	0.356783377
cg16034881	LRRC49;THAP10	1.11E-05	0.356783061
cg26577201	CRYBA2;CRYBA2;CRYBA2	0.000654035	0.356745828
cg20740029	SLC5A8;SLC5A8	1.50E-05	0.356745014
cg16524928	NKX2-2	1.11E-05	0.356738937
cg24305584	SLC16A12	0.000537905	0.356734382
cg08522426		4.34E-05	0.356733985
cg20541456	CYFIP2;CYFIP2;CYFIP2;CYFIP2	0.002304201	0.356730688
cg00829961		0.000233422	0.35672443
cg20000718		0.000117986	0.356673028
cg02106941	NEFM;NEFM	9.24E-05	0.356665928
cg18780257	FGF12;FGF12;FGF12	0.000187194	0.356656765
cg15183083	KCNA4;KCNA4	1.50E-05	0.356644812
cg18876990	NGDN;NGDN	0.003189589	0.356624573
cg21540621		7.23E-05	0.356624136
cg05471523		0.000955793	0.356609669
cg04166042		0.000148457	0.356605514
cg04880091		8.60E-06	0.356580312
cg17338208	TFPI2;TFPI2	0.000233422	0.356552247
cg24340081	NKAIN3	0.000537905	0.356545177
cg01162672	EYA4;EYA4;EYA4	0.00043997	0.356524684
cg01047555	C6orf174	0.001948248	0.356521125
cg02246609		9.24E-05	0.356520361
cg15343119	GALR1	2.58E-05	0.356510472
cg04134305	C7orf13;RNF32	0.00041966	0.356508074
cg00830817	C11orf87	4.34E-05	0.35649332
cg18172877	IRX4	5.59E-05	0.356479587
cg03287340		0.000290372	0.35647147
cg26950557	EGFLAM	0.000233422	0.356471244
cg05172912	MOGAT1	0.000233422	0.356464079
cg22059733	MAPK4	0.000537905	0.356452461
cg06279276	B3GNT9	0.000791389	0.356447528
cg14183540	OSBPL5;OSBPL5;OSBPL5	9.24E-05	0.356445679
cg24990212	CYP7B1	2.58E-05	0.356427657
cg00586644	CHAT;CHAT;CHAT;CHAT;CHAT;CHAT;CHAT;C	5.59E-05	0.356407145

cg09971646	DLK1	4.34E-05	0.356400423
cg13088432	TNKS	0.000148457	0.356392725
cg17931661	GULP1	8.60E-06	0.356387473
cg13294849	SOX2OT	0.003486652	0.356365866
cg06233376	A2BP1;A2BP1	1.11E-05	0.356346446
cg03760839	TBX15	3.34E-05	0.356341033
cg01937793	FAM189A2	2.58E-05	0.356308537
cg12113984		9.24E-05	0.356306666
cg00498604	TLL1	3.34E-05	0.356301758
cg25056460	PPAPDC1A	0.000955793	0.356277723
cg00032805	MARCH1	8.60E-06	0.356271288
cg25006077	MBNL1;MBNL1;MBNL1;MBNL1;MBNL1;MBNL1	2.58E-05	0.356265377
cg19426522		0.000233422	0.35626512
cg06983174	XKR4	0.001147425	0.356264552
cg13679804		0.006823935	0.356246031
cg19111459		1.96E-05	0.356235408
cg00355992	ZNF214;NLRP14;ZNF214	1.96E-05	0.35622988
cg16530437		0.00043997	0.356224356
cg17004208	MPPED2	0.000357885	0.356220877
cg08431704	LOX	5.59E-05	0.356220125
cg22830707	HOXC13	1.11E-05	0.356217652
cg18358020		7.23E-05	0.356198538
cg00488829		2.58E-05	0.356194528
cg20287234	GPR55	4.34E-05	0.356187242
cg02128244		1.50E-05	0.356181243
cg11621911	NALCN	0.001147425	0.356179427
cg19317413	LMX1A	3.34E-05	0.356178631
cg16910251		9.24E-05	0.356176112
cg23584144	DACT2	3.34E-05	0.356122238
cg09416188	DAG1;DAG1	1.96E-05	0.356120451
cg13912545	EYA4;EYA4;EYA4	0.000148457	0.356109627
cg08917174	MARCH4;MARCH4	0.000117986	0.356108358
cg16581884	PGR;PGR	4.34E-05	0.356107531
cg26509715	EN1	0.000117986	0.356089792
cg00434010	SIM1	7.23E-05	0.356072102
cg25250358	PLOD2;PLOD2;PLOD2;PLOD2	1.50E-05	0.356071311
cg01498832	RPTOR;RPTOR	2.58E-05	0.356058432
cg08146323	ZNF835	4.34E-05	0.356049384
cg20792735	CTNNA2;LRRTM1;LRRTM1;CTNNA2	1.50E-05	0.356049098
cg21849844		0.00043997	0.356041076
cg12510708	NFE2L3	0.002714607	0.356024284
cg23986620		7.23E-05	0.356023386
cg21320567	CSPP1;CSPP1;COPS5	7.23E-05	0.356020646
cg13409248	ENTPD3	0.000117986	0.356015215
cg16326242		4.34E-05	0.356001739
cg01530101	KCNQ1DN	0.000148457	0.355998728
cg19942083		0.000148457	0.355993108
cg09681089		0.00043997	0.355983367
cg05996052	C8orf85;C8orf85	2.58E-05	0.355983265
cg18977541		7.23E-05	0.355978201
cg14426525	SMPDL3A	0.000654035	0.355975614
cg24011260	DPP6;DPP6	4.34E-05	0.355971227
cg00008036	FAM163A	1.11E-05	0.355963356
cg06873316	NELL1;NELL1	0.000955793	0.355962283
cg07465627	STXBP4	0.000233422	0.355957707
cg05673882	POLK	0.000117986	0.355951975
cg21300373	MARCH1	0.000233422	0.355939126
cg09523275	NKAPL	0.000148457	0.355924395
cg22386583	RPTOR;RPTOR	5.59E-05	0.355898214
cg04092800	PTGER4	0.004357816	0.355870667

cg25464921		0.000654035	0.355838123
cg04776597		2.58E-05	0.355824961
cg26986937	ETV1;ETV1;ETV1;ETV1;ETV1;ETV1	0.000187194	0.35582257
cg21865845	ROBO1	0.000117986	0.355813654
cg16292768	CLU;CLU;CLU	5.59E-05	0.355813572
cg01893322		7.23E-05	0.355806679
cg01074767	C1RL;LOC283314	0.000233422	0.355800932
cg05438320	FAM123A;FAM123A	0.000117986	0.355798543
cg06992688	OTUB2	5.59E-05	0.355785606
cg01166145		0.00156564	0.355765193
cg08386692	SHISA2	0.000290372	0.355755115
cg15699693	MYOZ3;MYOZ3	0.000187194	0.35575441
cg13783152	SH3GL2;SH3GL2	7.23E-05	0.355734666
cg05250458	ZNF177	1.11E-05	0.355733048
cg13366774	C12orf59	0.000290372	0.355699564
cg05324194		1.50E-05	0.355692575
cg14663914	AZU1	0.00043997	0.35569155
cg10887021	PCDHB2	0.000117986	0.355688288
cg09141662	TDH	3.34E-05	0.355684625
cg10050661	KRT222	3.34E-05	0.355674202
cg16219810	FRMD5;FRMD5	4.34E-05	0.355665903
cg26718433	CXCL12;CXCL12;CXCL12	0.002714607	0.355653346
cg00654888	EN1	8.60E-06	0.355624884
cg02368812	NQO2	1.11E-05	0.355614226
cg04839289	JPH4;JPH4	3.34E-05	0.355611631
cg13736376	PITX2;PITX2;PITX2	1.50E-05	0.355601816
cg11118962		7.23E-05	0.35560176
cg04421973	ADAMTS9	3.34E-05	0.355591116
cg26246127		5.59E-05	0.355580951
cg01715248		0.000187194	0.355575062
cg06578434	SBNO2	1.96E-05	0.355565741
cg27367615		0.000117986	0.355563548
cg21514612		1.96E-05	0.355541892
cg26395694	ITPR1;ITPR1;ITPR1	0.001147425	0.355517259
cg16175599		2.58E-05	0.355506743
cg16674484	NDST1	0.00043997	0.355499443
cg26390889	RXFP3	2.58E-05	0.35549271
cg18187581		5.59E-05	0.355469667
cg19867917	COLEC11;COLEC11	9.24E-05	0.355462372
cg01031441	C12orf56;C12orf56	0.000654035	0.355451367
cg08754967	DIP2C	0.001639597	0.355419791
cg01361499		0.001948248	0.355418631
cg16584393	ANKRD30B	0.004357816	0.355407809
cg11075230	MGC70857;KIAA1688	8.60E-06	0.355393704
cg14937343		5.59E-05	0.355393698
cg01176516	ZNF727	0.001376172	0.355388896
cg01281157	GRM6	0.000290372	0.35536812
cg13398192	FAM155A	1.50E-05	0.355367742
cg04510788	GRIK3	0.000791389	0.355367563
cg18213472	SNAP91;SNAP91	0.003189589	0.355366692
cg21530453	LOC283856;GNAO1;GNAO1	0.000148457	0.355362956
cg17717259		0.000654035	0.355362599
cg23372684	COL23A1	9.24E-05	0.355340253
cg05396897	NLRP3;NLRP3;NLRP3;NLRP3;NLRP3	0.000357885	0.355322536
cg15248035	CCIN	4.34E-05	0.355292498
cg02363526	KCNH2;KCNH2	1.50E-05	0.355287481
cg21002528	CRY2;CRY2	5.59E-05	0.355282129
cg08965888		3.34E-05	0.355279297
cg11694519	CRYAB;HSPB2	0.000654035	0.35527861
cg26132084	CXCL3	0.000290372	0.355265194

cg08673054	PDE4A;PDE4A;PDE4A	0.00043997	0.355263913
cg10537555	CHD5	0.000187194	0.355227999
cg06816235	BDNF;BDNF;BDNF;BDNF;BDNF;BDNF;BDNF;B	7.23E-05	0.355216716
cg08109815	NMBR;NMBR	3.34E-05	0.355213348
cg07691897		3.34E-05	0.355212512
cg21963643	CACNA1E	0.00043997	0.355202432
cg17133183	CRABP1	0.000148457	0.355196176
cg04353095	SPSB4	0.000654035	0.355166757
cg17971936	AP3B2	5.59E-05	0.355141939
cg07852825	GHSR;GHSR	1.50E-05	0.355137349
cg22549558	SEMA5A	0.003486652	0.355120918
cg03043296	AP3B2	0.000117986	0.355119077
cg10659886	ZSCAN18;ZSCAN18	3.34E-05	0.355115394
cg22176895	PROX1;PROX1	0.000233422	0.355111883
cg09163720	INPP5A	0.000357885	0.355109996
cg06214770	MIR375	3.34E-05	0.355109041
cg04988098	GRID1	0.000233422	0.355103715
cg02664349	FAM38B	3.34E-05	0.355102909
cg10530169	DNAH12;DNAH12;DNAH12;DNAH12	1.96E-05	0.355098094
cg27313642	MIR124-2	8.60E-06	0.355096506
cg23302989	C7orf31	0.000187194	0.355095294
cg12457238		7.23E-05	0.35509036
cg15611413	HOXC13	0.000233422	0.355081601
cg13786801	TACSTD2	8.60E-06	0.355079499
cg18210226	CLVS2;CLVS2	7.23E-05	0.355068611
cg07303912	LRRN4	0.000187194	0.355066566
cg24995657	PLEK2	0.000654035	0.355038004
cg17394649	HCG4	0.000791389	0.35503722
cg08455099		0.000187194	0.355030123
cg22130262	MOS	3.34E-05	0.355018912
cg24267699	ABO	2.58E-05	0.355003195
cg15146720	GALNT11	4.34E-05	0.355000388
cg17660384	C11orf70	0.000148457	0.354987199
cg13031611		5.59E-05	0.354981121
cg03905413		7.23E-05	0.354957412
cg00476317	DLC1;DLC1	7.23E-05	0.354956656
cg08550523		0.001639597	0.35495102
cg13331200	CADM2;CADM2;CADM2;CADM2	5.59E-05	0.35493771
cg19930288	WWC2;C4orf38	1.50E-05	0.354887687
cg24347838	CCDC39	9.24E-05	0.354868125
cg15818671		0.000537905	0.354864262
cg03080985	SH3BGRL2	1.96E-05	0.354813442
cg07105285	MTNR1B	8.60E-06	0.354813285
cg24757160	NDUFS8	1.11E-05	0.354803401
cg01663016	SLIT3;SLIT3	8.60E-06	0.35479549
cg12828656	FSTL4	0.000187194	0.354794773
cg08245317		4.34E-05	0.354793665
cg20332645		5.59E-05	0.354790164
cg13722619	CDCP1;CDCP1	9.24E-05	0.354784043
cg12165782		0.000187194	0.354771478
cg11120913	C3orf14	7.23E-05	0.354760422
cg02276807		0.001948248	0.354752283
cg20482698	ACTN2	0.000233422	0.354739303
cg09586924	ST14	0.000537905	0.354736505
cg03835609	PCDHGA4;PCDHGA6;PCDHGA1;PCDHGA5;PC	5.59E-05	0.354733533
cg12804278	C10orf67	0.001147425	0.354733479
cg00569447		1.11E-05	0.354732739
cg14255256	FAM110C	0.003189589	0.354712437
cg14625175	HOXA10;HOXA10	0.000654035	0.35466987
cg06504526	SIM1	0.000357885	0.354668091

cg03441409	STEAP2;STEAP2	3.34E-05	0.354663681
cg14158073		0.000537905	0.354660209
cg27067781	PRRT1	1.96E-05	0.354633192
cg00065935	STXBP5L;STXBP5L	0.000233422	0.354593508
cg01687680	CBLN1	0.000233422	0.354589168
cg26747293	EGFLAM;EGFLAM	0.000537905	0.35457381
cg03032098	TUSC3;TUSC3;TUSC3;TUSC3	0.009066563	0.35456404
cg16167240	BSX	2.58E-05	0.354556722
cg03424342	FSTL1;FSTL1	0.000290372	0.354551539
cg22459630	LBXCOR1	0.000148457	0.354540507
cg26878949	PTCH1;PTCH1	8.60E-06	0.354531609
cg13449295	HIST3H2A	7.23E-05	0.354514155
cg21391741	MMEL1	1.96E-05	0.35450967
cg10857167		1.96E-05	0.354507814
cg10502121		1.50E-05	0.354488007
cg03363161		9.24E-05	0.354470612
cg05310486		9.24E-05	0.354447182
cg01697902	CTSG	0.000117986	0.354435032
cg01599167		0.000187194	0.354424398
cg05337137		8.60E-06	0.354415276
cg09072859	OPCML	9.24E-05	0.354407356
cg04761722	GRIN2A;GRIN2A;GRIN2A	1.11E-05	0.354405242
cg00820718	C1QL2	1.50E-05	0.354357596
cg02229993	FAM78B	0.000117986	0.354347003
cg19981568	SIX6	9.24E-05	0.354315878
cg18617589	CA8	0.00043997	0.354306998
cg05815255	CBLN1	0.000233422	0.354286877
cg18086761	TDRKH;TDRKH;TDRKH;TDRKH	0.000791389	0.354285026
cg04958236		1.50E-05	0.354283751
cg14056357	ZNF229	0.000526175	0.354283097
cg18207741		0.000955793	0.354253108
cg13661740		0.000290372	0.354239979
cg17446739	AIFM2	0.001376172	0.354236441
cg03654433		9.24E-05	0.354226388
cg09596234	LMO3;LMO3	3.34E-05	0.354222076
cg11787160	DTX1	0.000117986	0.354201779
cg27207796	FAM184B	0.000290372	0.354201415
cg06441668	PCSK2	1.50E-05	0.354198632
cg16590190	PDPN;PDPN	7.23E-05	0.354189864
cg19975346	FAM53B	7.23E-05	0.354184988
cg01051310	WNT3A	0.000148457	0.354164958
cg15145341	LOC646405	9.24E-05	0.354164268
cg27314569	BSX	9.24E-05	0.354157289
cg13610014		1.50E-05	0.354154177
cg00660167		5.59E-05	0.354148245
cg24061141	TBX20;TBX20	7.23E-05	0.354122813
cg27541691	TUBG2	0.000791389	0.354122618
cg13878010	ADCY5	5.59E-05	0.354114805
cg19103169		2.58E-05	0.354107544
cg22720041	ZNF229	0.000290372	0.354085851
cg08975720	FAM83H	0.000148457	0.354082582
cg26751356		0.001639597	0.354081712
cg13778723	PRTG	5.59E-05	0.354065947
cg22074950	FAM47E	5.59E-05	0.354062872
cg01040749	INPP5A	0.000233422	0.354059456
cg02954056		4.34E-05	0.354048517
cg23242898	DCC	3.34E-05	0.35403655
cg17265829	FAT4	0.000537905	0.35403367
cg01610488	TRPA1	1.96E-05	0.354021118
cg03080142		2.80E-05	0.353996883

cg16562275		0.002304201	0.353973264
cg02548238	LOX	8.60E-06	0.353972227
cg20956594		0.001639597	0.353964125
cg12691572	VTI1A	0.000654035	0.35395857
cg20271517	NPY2R;NPY2R	7.23E-05	0.353952178
cg26828017		0.000791389	0.353946617
cg21538208	MSX1	0.001948248	0.353916446
cg01513392		9.24E-05	0.35391636
cg05371552	VIPR2	8.60E-06	0.353916356
cg12653917		0.000233422	0.353915055
cg11435506	PON3	0.000955793	0.353871134
cg06008480	TTC12	1.96E-05	0.35387113
cg04548856	OTX2OS1	0.001147425	0.353869012
cg24109612	ID4	0.000233422	0.353868234
cg21070161	TMEM132E	1.11E-05	0.353866816
cg00750523	RGS7BP	1.11E-05	0.353858934
cg24944109	SLC1A2	4.34E-05	0.35382559
cg09595202		0.000357885	0.353820276
cg15798385	EVX1	0.000233422	0.353800938
cg21946824	NLGN1	0.000187194	0.353766804
cg08167951	PON3	9.24E-05	0.353763106
cg26541218	PKD1L1	0.000654035	0.353750795
cg10051615	C4orf38;WWC2	0.000117986	0.353744682
cg05688651		0.000955793	0.353740247
cg13491584	FOXI3	5.59E-05	0.353730778
cg05492442	MAPK4	8.60E-06	0.353716915
cg03181524	RYR1;RYR1	5.59E-05	0.353686393
cg12085977		5.59E-05	0.353684782
cg16778156		8.60E-06	0.353671241
cg05382097	JAKMIP1;JAKMIP1	8.60E-06	0.353655479
cg06603113	INSM2;INSM2	0.000187194	0.35364718
cg01271812	MEIS1	5.59E-05	0.353625682
cg17771605	LOC283392;TRHDE;LOC283392	0.000187194	0.353624592
cg26026615		1.11E-05	0.353615528
cg05878337	CTNND2	5.59E-05	0.353608413
cg07568194	ADAMTS18;ADAMTS18	3.34E-05	0.353597581
cg00455439	FAM13C;FAM13C;FAM13C;FAM13C;FAM13C	7.23E-05	0.353593241
cg25444208	SOX2OT;SOX2	0.000117986	0.353578051
cg07883457		1.11E-05	0.353577444
cg18013519	PPP2R2C;PPP2R2C	0.000187194	0.353576443
cg23428738		4.34E-05	0.353570255
cg09232478		0.000187194	0.353560646
cg02372145		8.60E-06	0.353541757
cg02262056		0.000233422	0.353535861
cg04969688	TJP1;TJP1	1.96E-05	0.353517838
cg08668316		7.23E-05	0.35350674
cg05505872	NR2F2;NR2F2	0.000233422	0.353506292
cg15825027		1.96E-05	0.353490422
cg04514644	CHST8;CHST8	1.96E-05	0.353487403
cg18058747	BHMT	0.000117986	0.353485737
cg06857614	MOGAT1	7.23E-05	0.353474812
cg22856324	CYP26B1	0.001639597	0.353470996
cg02838178	LAD1;LAD1	4.34E-05	0.353466748
cg24833739		1.96E-05	0.353464235
cg08501426		0.00043997	0.353461877
cg23754772	CIDEA;CIDEA	0.000148457	0.353458804
cg25833672		9.24E-05	0.353422902
cg23930711	PCDHGA4;PCDHGA4;PCDHGA2;PCDHGA1;PC	5.59E-05	0.353421357
cg01444716	BTBD3;BTBD3	0.000791389	0.353417391
cg16259229		9.24E-05	0.353400556

cg11359133	EVX2	0.000654035	0.353397627
cg18202449	ELAVL4	3.34E-05	0.353387505
cg22560214		3.34E-05	0.353380583
cg12572625	PCDHB1;PCDHB1	9.24E-05	0.353378305
cg02117721	CXCL6;CXCL6	8.60E-06	0.353375577
cg00629514		8.60E-06	0.353368869
cg23110109	MORN3	0.000117986	0.353366349
cg27106959	SRRM4	1.50E-05	0.353359618
cg10139846	INADL	9.24E-05	0.353355452
cg19060383	NPHS1	8.60E-06	0.353340921
cg06419432		0.001147425	0.353317171
cg20094085	PHYHIPL;PHYHIPL	0.000117986	0.353294791
cg01257828	FGF12;FGF12	8.60E-06	0.353292982
cg04175027		8.60E-06	0.353286188
cg09597312		8.60E-06	0.353253206
cg17797848	MYBPHL	0.000117986	0.353246891
cg04969067	LYNX1;LYNX1;LYNX1;LYNX1	0.000537905	0.353238311
cg05134015	SFTA3	5.59E-05	0.353234421
cg26053864	ACTC1	9.24E-05	0.353224669
cg04686354	MRPS7	4.34E-05	0.353223299
cg26367591	BRSK2	0.000187194	0.353217203
cg06436905	GLRB;GLRB;GLRB	0.000117986	0.353211328
cg02066682	FOXB2	1.11E-05	0.353177541
cg19485539	GFRA1;GFRA1;GFRA1	0.000654035	0.353173889
cg03712816	SALL3	1.11E-05	0.353144813
cg25778535	RIC3;RIC3;RIC3;RIC3	7.23E-05	0.353142578
cg00043819		2.58E-05	0.353141127
cg14068328	TBX18	1.96E-05	0.353140356
cg06957447		0.000187194	0.353138848
cg12177743	TTC12	3.34E-05	0.353124445
cg12745335	MYST2	0.001639597	0.353121226
cg01676795	POR	1.11E-05	0.353106384
cg03559235	LONRF2	0.000233422	0.353085307
cg01866606		2.58E-05	0.353069496
cg16558770	ZNF710	0.000537905	0.353061215
cg10280223	AP3B2	3.34E-05	0.353044606
cg07021644	UPK3A;UPK3A;UPK3A;UPK3A	0.000654035	0.353042896
cg09420637		0.000233422	0.353036771
cg27302539		0.000357885	0.353031974
cg00027232	TGOLN2	2.58E-05	0.353030946
cg11770080	BHMT	0.000955793	0.353001412
cg13674271		0.000187194	0.353000739
cg10606834		5.59E-05	0.352992344
cg02295078	ZNF287	7.23E-05	0.352989411
cg25693769	SUSD5	1.96E-05	0.352984742
cg11326968	FAM13C;FAM13C;FAM13C;FAM13C;FAM13C	0.000262362	0.352961507
cg10284973	C10orf41;C10orf41	0.000233422	0.352960109
cg16178603	MEIS1	3.34E-05	0.352951018
cg01838004	KIAA1529	8.60E-06	0.352927067
cg17507952	MARCH11	2.08E-05	0.352920448
cg19308222	EREG	5.59E-05	0.352917468
cg27312388	OPCML	0.000187194	0.352910963
cg19762657	MAL;MAL;MAL;MAL	8.60E-06	0.352898717
cg06275059	PCDP1	4.34E-05	0.352881881
cg02508664	HTR1E	8.60E-06	0.352881244
cg21775570	SKI	0.000205664	0.352859988
cg07477602	PPAP2B;PPAP2B	0.000117986	0.352857782
cg03411507	TRPC6;TRPC6	1.11E-05	0.352855552
cg06064179	PLEKHM1P	3.34E-05	0.352849176
cg10151741	PTPRS;PTPRS;PTPRS;PTPRS;PTPRS;PTPRS	0.00043997	0.352848107

cg11537367		0.000357885	0.352831493
cg16420798	STK32A;STK32A	4.34E-05	0.352829988
cg11645631	FAM159B	4.34E-05	0.352818732
cg00181125		8.60E-06	0.352809082
cg04386206	SLC7A14	7.23E-05	0.352798224
cg04079301	ZIC4;ZIC4;ZIC4;ZIC4;ZIC4	8.60E-06	0.352768185
cg16596523	PKP2;PKP2	1.11E-05	0.352765872
cg20180370	TFAP2D	7.23E-05	0.352757774
cg20962117		0.000117986	0.352752142
cg07539798	PCDHGA4;PCDHGA11;PCDHGA11;PCDHGA9;	1.96E-05	0.352749017
cg15549927	DPP6;DPP6	1.11E-05	0.352713649
cg04286455	LHFPL2	0.002714607	0.352704907
cg04231085		3.34E-05	0.352695859
cg12002303		4.34E-05	0.352691854
cg00152946		0.001376172	0.352678848
cg16956426	SGCE;SGCE;PEG10;SGCE;PEG10	1.96E-05	0.352671921
cg07964216	LPL;LPL	7.23E-05	0.352668832
cg03985727	MEOX2	9.24E-05	0.352666497
cg09284708	MOCS1;MOCS1	1.50E-05	0.352664984
cg17222645	LOC348840	0.006823935	0.352663213
cg15206171	KIFC1	3.34E-05	0.352641588
cg24291087	PCDH7;PCDH7;PCDH7;PCDH7;PCDH7;PCDH7	0.000233422	0.352638055
cg01899542	ZNF423	0.003189589	0.352634666
cg03140968	PAX1	1.50E-05	0.3526323
cg03553587		2.58E-05	0.352632049
cg26845297		1.50E-05	0.352613428
cg16536824	PLA2G7;PLA2G7	8.60E-06	0.352609123
cg17833106	PVRL4	0.000148457	0.352602584
cg25790531		0.000117986	0.352549431
cg20371765	ICA1;ICA1;ICA1	9.24E-05	0.352535147
cg17535647	PDE3A	0.00043997	0.352506858
cg12806353	PLA1A	4.34E-05	0.352506511
cg11469061	PAX6;PAX6;PAX6	3.34E-05	0.352486594
cg11372422	UGT8;UGT8	1.96E-05	0.352450813
cg06808498	SIX6	4.34E-05	0.352450356
cg11718030	BDNF	0.000117986	0.352445101
cg23193639	IGFBP3;IGFBP3	0.001639597	0.35243144
cg17508591	PCDHA6;PCDHA2;PCDHA1;PCDHA7;PCDHA1	2.58E-05	0.352413187
cg04640920		0.000148457	0.352396654
cg09509183	IRF6	0.000117986	0.352373447
cg18309752	SUMO1;SUMO1;SUMO1	1.50E-05	0.352372271
cg11453343	FAM176A;FAM176A	3.34E-05	0.352366295
cg06059360	NKTR	0.000187194	0.352358259
cg23994043	SATB2	0.000955793	0.352351084
cg02935338	CDH2	5.59E-05	0.352346752
cg12493075		3.34E-05	0.352335864
cg19250799		4.34E-05	0.352331637
cg22474886	SASH1	9.24E-05	0.35232264
cg25185173		0.00043997	0.352312371
cg16419629	NLGN1	7.23E-05	0.35230625
cg08368934	GPR97	7.23E-05	0.352302637
cg01186457	NPHS1	1.11E-05	0.352292217
cg21931419	TMEM90A	0.000233422	0.352286565
cg24111955		0.000357885	0.352278756
cg13297960	NCAM2	4.34E-05	0.352238845
cg20488281	CCDC60	9.24E-05	0.352232582
cg00183186	HTR1B	1.96E-05	0.352193211
cg23332582	SPSB4	0.004357816	0.352192236
cg11851456	NGDN;NGDN	0.005897668	0.352190816
cg01522826	TSPAN19;LRRIQ1;LRRIQ1;LRRIQ1;LRRIQ1	9.24E-05	0.352172548

cg20041802	RORA	3.34E-05	0.352170257
cg05547200		0.000233422	0.352165241
cg17482089	PCDHA6;PCDHA2;PCDHA1;PCDHA9;PCDHA7	4.34E-05	0.352155933
cg15057061	SOX2OT	0.000290372	0.352153958
cg00935653		2.58E-05	0.352151234
cg16767801	PAX1	0.000117986	0.352149264
cg03302259	ACP5;ACP5;ACP5;ACP5	0.000537905	0.352142677
cg12765064		0.000117986	0.352139014
cg12167167	LOC441177;C6orf176;C6orf176	0.000357885	0.352136009
cg02832512	FLJ22536	9.24E-05	0.352123159
cg13738144	PRDM13;PRDM13	1.50E-05	0.35212297
cg25012739		2.58E-05	0.352121526
cg15193782	ARMC3;ARMC3	1.50E-05	0.352112944
cg16823535		1.96E-05	0.352108575
cg22055427	P2RY1	0.000148457	0.352100816
cg06679720	DLEU7	0.000148457	0.352086671
cg18023065	FUT4	7.23E-05	0.352083813
cg22678398	RAP2B;RAP2B	0.003734675	0.352079297
cg04048259	EDN3;EDN3;EDN3;EDN3	0.006823935	0.35207546
cg10994148	C6orf168	0.000117986	0.352025874
cg14873600		8.60E-06	0.352019851
cg24922143		0.00043997	0.352012975
cg15627078	TNFRSF19;TNFRSF19	0.005897668	0.352006817
cg20381975		2.39E-05	0.35200411
cg01048372	C8orf73	8.60E-06	0.35200182
cg14184873	RND3	4.34E-05	0.351977163
cg21126487		2.80E-05	0.351961184
cg20410810	CACNG4	0.000955793	0.351959324
cg20931033	AGBL4;AGBL4	8.60E-06	0.351948105
cg23421262	SNCAIP	0.000357885	0.35194453
cg00822399		1.50E-05	0.351925171
cg14535980	C2orf40	0.000187194	0.351920555
cg27062759		0.001376172	0.351915213
cg10275315	BNC1	5.59E-05	0.351905623
cg07901138	TDRD5	1.50E-05	0.351893037
cg20578175	ADAMTS17	0.002450672	0.351889404
cg08143343	SLC6A15;SLC6A15;SLC6A15	0.000187194	0.351882698
cg02859837	PTGFR;PTGFR	2.58E-05	0.35187333
cg04603976	ZBTB7A	3.34E-05	0.35187063
cg00254133	NTSR1;NTSR1	0.000187194	0.351862179
cg11597277	SUMO1P1	2.58E-05	0.351851478
cg13571358	TMEM171;TMEM171	1.96E-05	0.351824933
cg01758805	STOX2;STOX2	2.58E-05	0.35181855
cg18032969		1.96E-05	0.351817155
cg09994553	TAF3	0.001195582	0.351813537
cg23778841	TFDP1;TFDP1	0.000148457	0.351805158
cg11558731	MYH14;MYH14;MYH14	4.34E-05	0.351792889
cg25835226		3.34E-05	0.351787942
cg02960853	GPR37	0.000233422	0.351782358
cg03442378	ALX4	0.000791389	0.351770704
cg21923442	MPPED2	0.000537905	0.351751379
cg05787556	TLX3	8.60E-06	0.351711845
cg07033624		2.58E-05	0.351707907
cg03606772	CRCT1	0.000233422	0.351694232
cg02756683		0.004357816	0.351667362
cg01632240	AP3M2;AP3M2	1.96E-05	0.351659352
cg25523509	PAX2;PAX2;PAX2;PAX2;PAX2	0.000290372	0.351656031
cg05398580	C7orf52	0.000233422	0.351652408
cg21129615	PCOLCE2;PCOLCE2	0.000791389	0.351630979
cg14033514		8.60E-06	0.351620438

cg18336674	TLX3	2.58E-05	0.351611558
cg17807479	SHISA6	1.96E-05	0.351608569
cg20620326		4.34E-05	0.351599867
cg24277313	PRKAA2	0.000148457	0.351596902
cg24919972	FYCO1	3.34E-05	0.351575307
cg10708905	LRIG3;LRIG3	5.59E-05	0.351573796
cg18546236		1.50E-05	0.351554802
cg26799209	MSC	8.60E-06	0.351542292
cg26989374	ZNF830;CCT6B	0.000187194	0.351530145
cg08363794	HOXD9	1.50E-05	0.351527894
cg25920792	HTRA1	7.23E-05	0.351504707
cg10692363		9.24E-05	0.351502022
cg08927739	ASCL1	3.34E-05	0.351478891
cg26693467	EMX2OS;EMX2;EMX2	3.34E-05	0.351472354
cg00246932	DKFZP434H168;GNAO1;GNAO1	0.002304201	0.351466769
cg14554491	C19orf30	9.24E-05	0.351436486
cg23508813		1.11E-05	0.351435554
cg15042811	FREM3	0.001639597	0.351432322
cg00511674		5.59E-05	0.351427124
cg08722774		0.000117986	0.35142264
cg16151503	CCNY;CCNY	0.000117986	0.351417843
cg18077304	SLC4A8;SLC4A8	0.000187194	0.351417569
cg15179113	C7orf13;RNF32	0.007869847	0.351413944
cg13686044		0.000262362	0.351413127
cg11905488	CLEC12A;CLEC12A	0.000791389	0.35139771
cg26019112	PAX6;PAX6;PAX6	7.23E-05	0.351387883
cg04401986	FGF5;FGF5	2.58E-05	0.351383024
cg23462514	RNF212;RNF212	0.000290372	0.351375289
cg14799457		0.000148457	0.351357164
cg24188073	PARM1	0.005897668	0.35135205
cg23424273	HTR1B	1.96E-05	0.351346183
cg02902975	LRRC36;KCTD19	0.001376172	0.351327307
cg16970851	ST8SIA1	0.000205664	0.35132113
cg17357285		0.000233422	0.351317391
cg03780851	GRHL2	1.50E-05	0.351281261
cg02936872	CTSL2	0.000117986	0.351268689
cg08101303	DLX5	0.000791389	0.351257577
cg15501281	SLC24A3	0.000357885	0.35124909
cg27075187	TEAD1;TEAD1	0.000654035	0.351235472
cg16206504		7.23E-05	0.351226302
cg22612764	FAM3B;FAM3B	0.000233422	0.351215787
cg20710519		0.001147425	0.351196996
cg18830442	SAMD5	1.11E-05	0.351192583
cg05234035		0.001948248	0.351191535
cg08430329	NOTO	7.23E-05	0.351187971
cg14196840	CTTNBP2	7.23E-05	0.351176736
cg09892203	CACNG4	4.34E-05	0.351152995
cg26896762	ISL1;ISL1	8.60E-06	0.351152158
cg22605919	C17orf104	0.000117986	0.351151349
cg04226002	ZBTB16;ZBTB16	0.001147425	0.351150855
cg23158731	COL14A1	1.96E-05	0.351141582
cg20973720	RASGRF1;RASGRF1;RASGRF1;RASGRF1	0.000654035	0.351127448
cg01866431	GLT1D1	4.34E-05	0.351114986
cg04836428	DTNA;DTNA;DTNA	3.34E-05	0.351109404
cg17507573	RASGRF1;RASGRF1;RASGRF1;RASGRF1	9.24E-05	0.351108677
cg02083559	SOX5;SOX5	0.000187194	0.351106098
cg24690314	PHACTR2;PHACTR2;PHACTR2;PHACTR2	4.34E-05	0.351103482
cg02477557		0.000955793	0.351095115
cg20989855	KCNIP4;KCNIP4;KCNIP4;KCNIP4	7.23E-05	0.351086294
cg24228819	CHRM2;CHRM2;CHRM2;CHRM2;CHRM2;CHRI	1.50E-05	0.351077791

cg00821098	ENTPD7	0.000148457	0.351061723
cg13623495		8.60E-06	0.351036668
cg15677916		1.96E-05	0.351032779
cg01972751	OLIG3	0.000537905	0.351012133
cg04719819	KCNA4	1.50E-05	0.351001065
cg09447675		0.00043997	0.350999475
cg22400015	RESP18	1.96E-05	0.350991518
cg27013696	TWIST1	0.000117986	0.350989305
cg16400999	ZNF385B;MIR1258	0.000357885	0.350979735
cg21488279	PRDM6	1.96E-05	0.350971951
cg07927379	C7orf13;RNF32	0.000654035	0.350965971
cg09126273	PTPRO;PTPRO	0.000148457	0.350961538
cg00861009	HOXD11	0.002927025	0.350961385
cg02291164		5.59E-05	0.350958224
cg07757861	PTPRZ1	1.11E-05	0.350955544
cg24874350		1.11E-05	0.350953209
cg02060689		5.59E-05	0.350946427
cg23363832	RBP1;RBP1;RBP1	0.002304201	0.350934703
cg19367540	KCND3;KCND3	0.000117986	0.350922136
cg12480034	DAB1	3.34E-05	0.350921482
cg11676902	PRDM13	5.59E-05	0.350916582
cg01572696	IDUA	4.34E-05	0.350905424
cg12422450	CHGA	7.23E-05	0.350903815
cg26371320		0.000117986	0.350897194
cg13381984	LEP;LEP	2.58E-05	0.350884316
cg26352006	RAI14;RAI14;RAI14;RAI14;RAI14;RAI14	0.001147425	0.350884274
cg04943741	VPS28;VPS28	1.96E-05	0.350880157
cg02026062	FAM162B	4.34E-05	0.350864903
cg13218903	RPRM	7.23E-05	0.350859458
cg27431596	ZNF697	0.001639597	0.350851935
cg26926612		9.24E-05	0.350846608
cg01046309	TMC7;TMC7	0.000187194	0.350811589
cg06779449	TOX2;TOX2;TOX2	0.000117986	0.350762947
cg03094134	TRIM39;TRIM39	4.34E-05	0.350761519
cg15811668	MEG8	0.0050758	0.350736615
cg01311151		0.000233422	0.350736395
cg22367678		3.34E-05	0.350709202
cg01498829	EVL	1.50E-05	0.350702889
cg04839259	TACC2;TACC2	1.11E-05	0.350700468
cg11752100	POU6F2;POU6F2	0.000357885	0.350697151
cg25344401	FOXK1	0.001948248	0.350692146
cg19425332	VAX1;VAX1	2.58E-05	0.350678576
cg23325963		2.58E-05	0.350669775
cg11163901	NKX6-2	1.50E-05	0.350663109
cg14186066	SIX6;SIX6	1.11E-05	0.350657109
cg21780711		1.11E-05	0.350657008
cg13723118	NTRK2;NTRK2;NTRK2;NTRK2;NTRK2;NTRK2;I	0.000117986	0.350640605
cg26551026	CABLES1;CABLES1	4.34E-05	0.350636217
cg24928333		4.34E-05	0.35060518
cg14489366	PRUNE2	9.24E-05	0.350604778
cg17160751	TACR3	8.60E-06	0.350600072
cg02527112	HOXD11	8.60E-06	0.350583226
cg22355517	PDE3A	3.34E-05	0.350581447
cg05304531	DHDDS;HMGN2;DHDDS	0.000791389	0.350579357
cg17199800	DLX6AS	1.11E-05	0.350575008
cg15684962		8.60E-06	0.350569433
cg06163371	EPHA10;EPHA10	0.000187194	0.350554568
cg17051321	BNC1	1.50E-05	0.350525071
cg21214293		1.11E-05	0.350524938
cg05059605	BARX2	0.006823935	0.350521864

cg00760935	DCHS2;DCHS2	2.58E-05	0.350518298
cg24387472		9.24E-05	0.350492244
cg12298268	RBP1;RBP1;RBP1	0.000537905	0.350482698
cg12818557	MMP16;MMP16	0.000290372	0.350477668
cg20789700	RBM47	0.000537905	0.350476718
cg13019143	DLX6AS	0.000117986	0.350475025
cg05210904		8.60E-06	0.35047381
cg08697689	SLITRK1	8.60E-06	0.350468412
cg17607298		0.000233422	0.350458256
cg14252279	DAB2IP	0.000955793	0.350452315
cg17444738	NKX6-1	8.60E-06	0.350449321
cg16196812	PRLHR;PRLHR	8.60E-06	0.350436784
cg15281710		1.50E-05	0.350407929
cg04851712		4.34E-05	0.350393475
cg14288330	INSM2	0.000955793	0.350382727
cg16357388	CRMP1	7.23E-05	0.350346332
cg11521965	PTN	0.000187194	0.350345005
cg04892170	ADAM12;ADAM12;ADAM12;ADAM12	1.11E-05	0.350343206
cg12052661	CACNA1B	1.50E-05	0.35033965
cg10805039	NXN	0.00043997	0.350330451
cg08330224	SHISA9;SHISA9	0.000654035	0.350284667
cg18071147	FAM189A1	0.000148457	0.350254876
cg09214243		0.000117986	0.350250331
cg08196553	SOX2OT	5.59E-05	0.350245132
cg05006067	DOC2B	0.000148457	0.350241035
cg17592231		1.50E-05	0.350218989
cg00094898		0.000357885	0.350216045
cg21858485		0.000233422	0.350211524
cg24322398	FADS6	0.000537905	0.350208422
cg13292042		0.000117986	0.350181611
cg21429745	LBX2	7.23E-05	0.350170635
cg06959053	RASGRF1;RASGRF1	0.000365373	0.350170101
cg13392022	ADAR	8.60E-06	0.350168384
cg14576896	GJD2	1.50E-05	0.350129131
cg20043105	C14orf23;C14orf23	1.96E-05	0.350128019
cg08115833		1.50E-05	0.350126784
cg12825804	VASH1	9.24E-05	0.350098971
cg15779716	CDCP1;CDCP1	5.59E-05	0.350097578
cg24370698	PGM1	0.000955793	0.350094558
cg00586817		2.58E-05	0.350092587
cg15162330	LIMCH1;LIMCH1;LIMCH1	4.34E-05	0.350088944
cg18587806	LIMCH1;LIMCH1;LIMCH1;LIMCH1;LIMCH1;LIM	0.002040485	0.350076468
cg03120185	PRDM13	4.34E-05	0.35004454
cg25287268	DLEU2	0.000955793	0.350040129
cg20969194	PAX3;PAX3;PAX3;CCDC140;PAX3;PAX3;PAX3	1.50E-05	0.350028414
cg05960938		0.000233422	0.350021908
cg10743390	VSTM2B	1.11E-05	0.350021724
cg11529819	FCN3;FCN3	0.000187194	0.350018695
cg24453699		0.00043997	0.349987162
cg00557360	C20orf95	0.000187194	0.349974804
cg06335343	TMEM20;TMEM20	0.000148457	0.349971439
cg02976719	PPAPDC1A	0.000537905	0.349951749
cg20510207	EPHA10;EPHA10;EPHA10;EPHA10	0.001376172	0.349909308
cg12124386	FAM5C	3.34E-05	0.349902504
cg26194477	FOXG1	2.58E-05	0.349897167
cg09165004		0.000187194	0.349891067
cg24012880	TSPAN18	0.001948248	0.349890079
cg07980216	OSBP2	1.11E-05	0.349822155
cg07922443	HAND1	5.59E-05	0.349811084
cg01075271		4.34E-05	0.34981047

cg14748795		3.34E-05	0.349808873
cg20387341	FGF10	3.34E-05	0.349805141
cg13016524		8.60E-06	0.349782671
cg06346138	MST1P9	8.60E-06	0.349780238
cg04908625	ADCY5	8.60E-06	0.349774877
cg07413251		0.001147425	0.349770903
cg14795968	ACADL	8.60E-06	0.349766532
cg18175808		3.21E-05	0.349757848
cg01945562	PLOD2;PLOD2;PLOD2;PLOD2	5.59E-05	0.349749136
cg13095704	CALCR;CALCR	7.23E-05	0.34971205
cg18802998		0.000537905	0.349709639
cg02591356		0.000233422	0.349692287
cg09538995	SLC27A6;SLC27A6	2.58E-05	0.349685376
cg10422777		2.58E-05	0.349682274
cg07687119	HOXC4;HOXC6;HOXC5;HOXC6	0.001147425	0.349681996
cg23951961	BEND4;BEND4	0.000262362	0.349656763
cg15852572	PAQR9	0.000122184	0.349653494
cg16707405	CDO1	0.000148457	0.349636447
cg01168235		1.96E-05	0.349628022
cg14648916	CPLX2	5.59E-05	0.349624313
cg06433879	SNAP25;SNAP25	4.34E-05	0.349603511
cg03465947		0.000117986	0.349581522
cg19671533	SNAP91;SNAP91	5.59E-05	0.349535752
cg09607276	DRD4	0.002714607	0.349528103
cg20649017	HOXD10	0.000117986	0.349525233
cg19304273		8.60E-06	0.349522771
cg10457563	KIF6;KIF6	0.001147425	0.349520558
cg08449389		1.11E-05	0.349519442
cg07037371		0.00043997	0.349507799
cg16712637	ASCL1;ASCL1	0.000148457	0.3494976
cg01241721		7.23E-05	0.349483682
cg27423760	SALL1	2.58E-05	0.349468318
cg25116388	HOXB8	1.50E-05	0.349464076
cg17940821		2.58E-05	0.349452736
cg00485194	ZNF385D;ZNF385D	8.60E-06	0.349451644
cg01188592	RSPO4;RSPO4;RSPO4;RSPO4	5.59E-05	0.349435724
cg04175111		4.34E-05	0.349418955
cg00397673	RAX	4.34E-05	0.349395451
cg00083188	KCNMA1;KCNMA1;KCNMA1;KCNMA1	0.001376172	0.349373941
cg10150592		5.59E-05	0.349372392
cg14225168	NOTCH1	0.000187194	0.349361756
cg03917138	SOX9	5.59E-05	0.349353379
cg04673937	MIR548H4	9.24E-05	0.34934997
cg23390619	ARHGAP22	0.000187194	0.349335838
cg17385088		2.58E-05	0.349327571
cg04490714	SLC6A2;SLC6A2	4.34E-05	0.349325863
cg13941682	PCGF3	2.58E-05	0.349325659
cg24114154	HOXB4	0.000187194	0.349322396
cg03983543	C7orf57	1.11E-05	0.349317019
cg09897374		0.000148457	0.349309537
cg18856632		8.60E-06	0.349302679
cg14908255	IQSEC3;IQSEC3	0.000233422	0.349298748
cg00567190	C1orf97	0.00043997	0.349289308
cg26601317	KCNQ1DN	1.50E-05	0.349279293
cg12537382		0.000117986	0.349264331
cg11826104	UBE3A;UBE3A;UBE3A;UBE3A	0.000117986	0.349257764
cg16608018		2.58E-05	0.349253816
cg14664621	TACR3	0.000405139	0.349227554
cg26036993	CYP1B1	0.000148457	0.349227392
cg18026631		5.59E-05	0.349216166

cg15904283		9.24E-05	0.349204422
cg20947082	KCNK1	0.000148457	0.349196431
cg02968429	NR2F2;NR2F2	5.59E-05	0.349183848
cg20440901	SLC19A3	1.96E-05	0.349167919
cg01932823	GPR97	9.24E-05	0.349151519
cg10486998	GALR1	2.58E-05	0.34913332
cg19694010		0.000290372	0.349131499
cg05789595	DST;DST;DST;DST	0.000791389	0.349105228
cg18714767	FAM18B2;FAM18B2;FAM18B2;FAM18B2	0.000233422	0.349101318
cg24818566	DBC1	8.60E-06	0.349096055
cg16004055	SCARA5	3.34E-05	0.349083296
cg06889108	CPEB4	7.23E-05	0.349081299
cg04633225	PDX1	0.000117986	0.349067834
cg17630392	NPR3	0.000955793	0.349067276
cg14306819	ERCC3	0.000187194	0.349060826
cg20192387	RPS6KA2;RPS6KA2	1.96E-05	0.349028618
cg13766864	KCNE3	1.50E-05	0.34902529
cg03086707	PCDHGA4;PCDHGA2;PCDHGB2;PCDHGA1;PC	0.000357885	0.349022163
cg17267805	CXCL12;CXCL12;CXCL12	0.009066563	0.349018509
cg23013850	FAM124B;FAM124B	0.0050758	0.348997647
cg00998438	ILDR2	0.000101153	0.348997333
cg11583475	CCDC149;CCDC149	0.001639597	0.348985929
cg25959239	LARP6;LARP6	0.000654035	0.348984988
cg13054224	IGDCC3	8.60E-06	0.348971061
cg15513743	TBX15	0.000955793	0.348966272
cg12629325	PCDHAC1;PCDHA7;PCDHAC1;PCDHA12;PCD	3.34E-05	0.348961315
cg02179652	BSX	0.000357885	0.348951857
cg16847428		1.50E-05	0.348951665
cg13909214	ESRP1;ESRP1;ESRP1;ESRP1;ESRP1	9.24E-05	0.348946564
cg05178576	ADAMTS18	0.000148457	0.348939328
cg03295083	NRXN1;NRXN1;NRXN1	0.00043997	0.348922652
cg15801340		4.34E-05	0.348917082
cg01031101	NKAPL	0.001948248	0.348905101
cg15912800	MIR196B	0.000290372	0.348888594
cg10281002	TBX5;TBX5	0.000187194	0.34888434
cg13448816		4.34E-05	0.348883059
cg02161478	OTX2OS1	1.11E-05	0.348862417
cg04481212	BDNF;BDNF;BDNF;BDNF;BDNF	0.000233422	0.348857923
cg21136104		0.000148457	0.348842375
cg22847221	CXCL2;CXCL2	9.24E-05	0.348811947
cg10742957	FBXO17	0.000791389	0.34880717
cg03277049		0.000654035	0.348802258
cg09881510	PPFIA2;PPFIA2	8.60E-06	0.348795055
cg22152728		0.002714607	0.348770976
cg02189888		5.59E-05	0.348760383
cg04880732	SLC24A4;SLC24A4;SLC24A4	0.000233422	0.348758083
cg05161082	C12orf39	0.000290372	0.348744835
cg03217897	AGBL4	2.58E-05	0.348737323
cg17922851	DZIP1;DZIP1	4.34E-05	0.348733512
cg08118344	RAP1GAP;RAP1GAP;RAP1GAP	0.000290372	0.348716716
cg09140778		1.96E-05	0.348716258
cg03573529		8.60E-06	0.348713291
cg00656387	ENTPD3	3.34E-05	0.348706708
cg11440030		0.000187194	0.348704037
cg08388041		0.000654035	0.348702893
cg21956337	NTRK3;NTRK3;NTRK3	0.000117986	0.348643348
cg25934680	MYO1D	8.60E-06	0.348635499
cg21075680		0.001147425	0.348573691
cg07068191	HRASLS;HRASLS;MGC2889	1.96E-05	0.348573644
cg04064160		4.34E-05	0.348568026

cg25353171		1.96E-05	0.348554888
cg02150654	PPAPDC1A	5.59E-05	0.348552478
cg09384610	RPH3A;RPH3A	2.58E-05	0.348542622
cg09628962	CSPG4;CSPG4	0.001147425	0.348536375
cg04104463	MIR124-2	0.000187194	0.348529234
cg21097036	C6orf174	1.96E-05	0.348515093
cg13575139		0.000187194	0.34851274
cg12329178	SHISA2	4.34E-05	0.348502993
cg07642043	GRIN2A;GRIN2A;GRIN2A	0.001948248	0.348488736
cg07098391	KDR	2.58E-05	0.348476155
cg14018735	C9orf96	5.59E-05	0.34846987
cg08186924	NPHS2	1.50E-05	0.348466912
cg18692678	TBX5;TBX5;TBX5;TBX5	0.000654035	0.34845839
cg21392341	TBX15	0.000955793	0.348448672
cg27428551	TMEM120B	1.11E-05	0.348446982
cg17368808	C20orf103	2.58E-05	0.348405265
cg10385303	SFTA3	1.11E-05	0.348391372
cg18839609	PHYHIPL;PHYHIPL	2.58E-05	0.348388985
cg10384245	ADCYAP1;ADCYAP1	0.000290372	0.348373066
cg18506390	EBF2	0.000187194	0.34837283
cg06962944	TOX2;TOX2;TOX2	0.002714607	0.348365401
cg25887069	GALR3	2.58E-05	0.348358756
cg15906794	YBX2	0.000187194	0.348350073
cg23690866	SOX9	0.001376172	0.348343434
cg02004845	CDH6	2.58E-05	0.348328062
cg24401219	RIMS1	8.60E-06	0.348305916
cg22797735	TBX3;TBX3	5.59E-05	0.348298418
cg11090352	EPHA6	0.000537905	0.348286369
cg26312807		0.000117986	0.348282914
cg05216056	TRIM27	1.96E-05	0.348278689
cg16626012	SLC47A1;SLC47A1	0.000187194	0.348266753
cg12997720	HOXA11AS;HOXA11	0.000570759	0.348266145
cg03464573	HOXA9	5.59E-05	0.348253619
cg02241330	CCDC105	1.11E-05	0.348241653
cg12116137	PRPF8	4.34E-05	0.348236022
cg09191036	DRD5;DRD5	0.000233422	0.348222408
cg24795958	ACTC1	0.000187194	0.348209149
cg00421139	GDF6;GDF6	0.000537905	0.348208657
cg18319921	ADAMTS19	9.24E-05	0.348207347
cg18391758	GRIN2A;GRIN2A;GRIN2A	8.60E-06	0.348191839
cg08431899	EVX2	3.34E-05	0.348190263
cg03202077	ADAMTS5;ADAMTS5	4.34E-05	0.348173174
cg10844844	KCNK5	9.24E-05	0.34815488
cg08340737		2.58E-05	0.348146862
cg02059626		1.96E-05	0.348086743
cg18291941	MIR663	3.34E-05	0.348073359
cg12026749	NEFM;NEFM	1.11E-05	0.348067308
cg17105755	C2orf65	8.60E-06	0.348027592
cg15215348	PDE4C	0.000654035	0.348023087
cg13583934	KCNK5	0.00043997	0.3480141
cg05335955	AP3B2	0.00043997	0.348013239
cg06699564		0.000148457	0.34800254
cg13217064	SOX14	0.000148457	0.347985222
cg25975712	FAM19A5;FAM19A5	0.004357816	0.347984939
cg09141953		0.000357885	0.347978893
cg23922739	HOXC13	0.00043997	0.347978784
cg03358769	C3orf55;C3orf55;C3orf55;C3orf55	0.000148457	0.34797449
cg04550737	TBX15	0.000654035	0.347972087
cg08782002	CCDC47	1.50E-05	0.34796963
cg27218796	HAND2	7.23E-05	0.347957239

cg20306837	GRIA4;GRIA4;GRIA4;GRIA4	1.96E-05	0.347955382
cg09069499	PPP2R2A	9.24E-05	0.347950863
cg27316886	PRMT8	9.24E-05	0.34794806
cg25241102	HNMT;HNMT;HNMT;HNMT;HNMT;HNMT	0.001147425	0.347939949
cg16404040		1.50E-05	0.347930397
cg04003615	RNF130	0.000148457	0.347913724
cg10748160	JAKMIP1;JAKMIP1	0.000290372	0.347903438
cg25947544	CADPS;CADPS;CADPS	0.001147425	0.347896342
cg11009245	ALDH1L1	7.23E-05	0.347861704
cg26985666	SLC1A2;SLC1A2	0.001639597	0.347861221
cg23936023	VCAN;VCAN;VCAN;VCAN	0.003734675	0.347847648
cg09904383	FCHSD1	0.001948248	0.347844456
cg12587766	LIFR;LIFR;LIFR	0.002714607	0.347844224
cg04728429		9.24E-05	0.347822998
cg06525651	FAM196A;FAM196A;DOCK1	0.000537905	0.347815965
cg07404921	LHX8	1.96E-05	0.347815826
cg06668194		5.59E-05	0.347812429
cg02174225	JAM3	9.24E-05	0.347793164
cg23316253		0.000290372	0.347791713
cg20027296	TLX3	0.000148457	0.347771523
cg21772773	CAMTA1	4.34E-05	0.347768337
cg01269620	PDE10A;PDE10A	4.34E-05	0.3477661
cg08078028	EIF3A	1.11E-05	0.347756129
cg11324504	SLC16A8	8.60E-06	0.347750944
cg23300372	CARTPT	4.34E-05	0.347736898
cg00036788		7.23E-05	0.347727045
cg17076890	PTF1A	8.60E-06	0.347720659
cg13964030		7.23E-05	0.347707682
cg26734888	HKR1	0.000117986	0.347694222
cg24440088		0.000148457	0.347683469
cg08269974	ELANE	0.000233422	0.347681477
cg20119148	PDE4C	0.000187194	0.347676672
cg01568492		8.60E-06	0.347667832
cg21870884	GPR25	8.60E-06	0.347640452
cg07502389	NEFM;NEFM	8.60E-06	0.347639787
cg21952901	MAP1B	0.001147425	0.347639239
cg07734159	VCL;VCL	0.002304201	0.347628201
cg26032119	ZNF204P	2.58E-05	0.347567696
cg10499176		0.000955793	0.347565793
cg11213383	SH3GL2	5.59E-05	0.347564622
cg23034922		0.000233422	0.347552679
cg03238298	DTNA;DTNA;DTNA	9.24E-05	0.347552056
cg20954977	B3GNT7	0.000233422	0.34754927
cg02232704	MYO3A	0.000654035	0.347540805
cg24298421	CASZ1;CASZ1	5.59E-05	0.347522518
cg22093640		0.000290372	0.34751819
cg07960624	SAMD12	0.001147425	0.34751351
cg17723958	CCDC92	2.58E-05	0.347511581
cg03292388	CPZ;CPZ;CPZ;CPZ;CPZ;CPZ	0.000654035	0.347507708
cg18283342	PCDHA6;PCDHA2;PCDHA1;PCDHA9;PCDHA7	0.000791389	0.347503046
cg01463154	BHLHE22	0.000334462	0.347499193
cg18405727	TRIM71	9.24E-05	0.347487273
cg09744766		3.34E-05	0.347485669
cg12285988		9.24E-05	0.347475423
cg23448348	RBP1;RBP1;RBP1	9.24E-05	0.347428856
cg12626956	FOXB2	8.60E-06	0.347421796
cg26060489		8.60E-06	0.347417313
cg21165221		8.60E-06	0.347414596
cg15658793	NFIC;NFIC	2.58E-05	0.347369414
cg13549719	TRPC4;TRPC4;TRPC4;TRPC4;TRPC4;TRPC4	2.58E-05	0.347360754

cg08018585	SIAH3	0.004357816	0.347355302
cg02000882		0.000233422	0.347346305
cg08194313	GFRA1;GFRA1;GFRA1;GFRA1	0.000537905	0.347330834
cg09926486	FRMD5	4.34E-05	0.347320758
cg18944924	FAM82A1	0.00043997	0.347316832
cg15711268		0.001376172	0.347310459
cg04751149	MEIS1	3.34E-05	0.347309155
cg23693485	GFOD1	7.23E-05	0.347305947
cg14222229		7.23E-05	0.347305498
cg10934870	PCYOX1	0.000187194	0.347272754
cg02671880		2.58E-05	0.347250076
cg27506082	ZDBF2	0.000148457	0.347244049
cg19568544	PCDHB5;PCDHB5	0.000117986	0.347240079
cg01243371	FOXF1	5.59E-05	0.347231925
cg13047308	RAPSN;RAPSN	3.34E-05	0.347203057
cg12167830	PRKAA2	0.000117986	0.347195785
cg17507671	PITX2;PITX2;PITX2	3.34E-05	0.34719243
cg08864944	PBX1	7.23E-05	0.347188136
cg19949955		0.000187194	0.347186798
cg18534345	PRLHR	1.50E-05	0.34717256
cg08195176	SIK3	1.50E-05	0.347169416
cg07235053	FUT4	0.000148457	0.347144313
cg08920610	PCSK9;PCSK9	9.24E-05	0.347137291
cg03039218	KIF18B	0.000187194	0.347133224
cg08866608		4.34E-05	0.347103678
cg07935012	DCC	1.11E-05	0.347100974
cg22336669		0.000233422	0.347099724
cg07002711	TTPA	0.000117986	0.347098801
cg01584932	TTC31;TTC31;CCDC142	0.000233422	0.347089366
cg07514505	HS6ST3	3.34E-05	0.347050963
cg03041738		1.50E-05	0.347048699
cg11323198	CDH8	1.50E-05	0.347047709
cg03294066	C8orf42	0.001948248	0.347040237
cg18560204	BNC1	1.50E-05	0.347037444
cg08876932	PHOX2A	0.000290372	0.347012322
cg15087347	CNTN1;CNTN1	0.000290372	0.3470043
cg18011401	CACNA1A;CACNA1A	0.00043997	0.346993193
cg09803262	DLX6AS	0.000290372	0.346989162
cg01546729	ZFPM2	0.000537905	0.34697519
cg24108286	TFDP1;TFDP1	2.58E-05	0.346971531
cg21011139	CYB5R2	0.000233422	0.346965749
cg09484039	FRZB;FRZB	7.23E-05	0.346961431
cg04550052	SALL1;SALL1;SALL1	0.004385436	0.346958338
cg19848629		0.000187194	0.346957928
cg07142797		0.000233422	0.346957366
cg13331300	KIAA1598;KIAA1598	5.59E-05	0.346949274
cg14262681		0.009456257	0.346948102
cg26963356	HMGCLL1;HMGCLL1	2.58E-05	0.346938514
cg17621718	CCK	0.000955793	0.346937367
cg27469154	HSPB8;HSPB8	0.004357816	0.346927408
cg06298740		0.004357816	0.346916595
cg08287724	KCNE1;KCNE1;KCNE1	1.11E-05	0.346890347
cg06184647		0.000791389	0.346886383
cg25537434		1.50E-05	0.346885673
cg24650785	BDNF;BDNF;BDNF;BDNF;BDNF;BDNF	4.34E-05	0.34687735
cg04873188	MXD1	0.004357816	0.346858703
cg18395636	RAB38	0.000955793	0.346857853
cg19969606	LHX5	4.34E-05	0.346855116
cg09378756	PCK2;PCK2	0.000365373	0.34685452
cg19592921	BMP7;BMP7	0.000117986	0.346847428

cg01195276		0.00043997	0.346837897
cg17663463	PEG3;PEG3;ZIM2;PEG3;PEG3;ZIM2;ZIM2	8.60E-06	0.346829509
cg02998333		2.58E-05	0.346810567
cg10903903		0.000187194	0.346805397
cg07178006		0.000117986	0.34679916
cg20916535	VGLL2;VGLL2	0.000357885	0.346793515
cg14380111	C15orf26	0.000357885	0.346792777
cg10159215	LPPR5;LPPR5	5.59E-05	0.346763406
cg24163242		0.000537905	0.346760534
cg03585447		2.58E-05	0.346750649
cg07363697		0.000791389	0.346744125
cg00253658		0.004357816	0.346742112
cg25707767	RSPH10B;RSPH10B2	8.60E-06	0.346732833
cg07702424		2.58E-05	0.346730142
cg08832227	KCNA1	0.000148457	0.346719685
cg18574157	CADPS2;CADPS2;CADPS2	0.000117986	0.346707313
cg06335143	ZYG11A	8.60E-06	0.346701532
cg27074174	GRIK2;GRIK2;GRIK2	0.000117986	0.346699953
cg13713523	RGS7BP	2.58E-05	0.346689075
cg08284213		0.000357885	0.34668639
cg02778418	ATOH1	1.11E-05	0.346684934
cg03723247		9.24E-05	0.346671261
cg21106486	CR1L	7.23E-05	0.346662199
cg13601427	SUSD5	0.000357885	0.346642418
cg03774026	FAM184A;FAM184A;FAM184A	0.000233422	0.346639309
cg03456968	WNT9B	0.001147425	0.346632834
cg10316635	INADL	0.000233422	0.346621851
cg03449175	HIST3H2BB;HIST3H2A;HIST3H2A	0.002304201	0.3466154
cg14526718		4.34E-05	0.346599806
cg08203253		9.24E-05	0.346594038
cg11273478	GABRB3;GABRB3	2.58E-05	0.346590525
cg00220721	PRR5L;PRR5L;PRR5L;PRR5L	0.000148457	0.346564102
cg25033393		4.34E-05	0.346563952
cg01469864	CPLX2;CPLX2	4.34E-05	0.346538895
cg26708235	ATP8A2	2.58E-05	0.346527256
cg18875371	C14orf23;C14orf23	2.58E-05	0.346519559
cg23693169		0.000117986	0.346518325
cg24424545	CNNM1	7.23E-05	0.346510119
cg11084334	LHFPL4	2.58E-05	0.346499466
cg16436762	PIWIL4	0.002304201	0.346477404
cg08474859		0.000233422	0.346471386
cg10996596		7.23E-05	0.346471166
cg00067018	DBX2	1.50E-05	0.346467878
cg13720108		1.50E-05	0.346462878
cg15493780	PRLHR	5.59E-05	0.346453257
cg17100943	SLC25A23	0.000791389	0.346450896
cg21809447	OLFM3;OLFM3	0.000110797	0.346436445
cg23974944	NOVA2	1.50E-05	0.346433717
cg08333333	ZNF835	5.59E-05	0.34642322
cg01957732	EYA4;EYA4;EYA4	0.000187194	0.346420142
cg03648789	CYP1B1	0.000955793	0.346415915
cg24512138	TF	1.96E-05	0.346414673
cg26161329	PPM1E	5.59E-05	0.346406166
cg24768649	ERBB4;ERBB4	1.11E-05	0.346395004
cg02071076	STOX2;STOX2	2.58E-05	0.346390958
cg11074047	NFATC2;NFATC2;NFATC2	0.009066563	0.346373674
cg23026675	LOC100192378	1.96E-05	0.346364977
cg01673674	GDF6	4.34E-05	0.346361474
cg23696618	SERPINB10	0.001639597	0.34635803
cg25734726	SERTAD3;SERTAD3;SERTAD3	0.000117986	0.34634429

cg05237436	SIL1;SIL1	0.000357885	0.346321318
cg20837557	RAB32	0.000148457	0.346315247
cg07622996	FAM84A	7.23E-05	0.346314455
cg20203469	ACAD8	2.58E-05	0.34630629
cg15473155	PEG3;PEG3;ZIM2;PEG3;PEG3;ZIM2;ZIM2	8.60E-06	0.346303754
cg13215970	CXCL5	3.34E-05	0.346303293
cg14761318	OCLN;OCLN	0.000290372	0.346297035
cg23667734		0.000233422	0.346286203
cg02788090	SOX9	0.000233422	0.346285219
cg06883496		1.96E-05	0.346267523
cg17356733	IFNGR2	0.000791389	0.346263377
cg19544372	ZNF560	5.59E-05	0.346222964
cg17900495	NHLH2;NHLH2	4.34E-05	0.346211831
cg07079231	DNAH3;TMEM159	7.23E-05	0.346188889
cg14319409	GLRA1;GLRA1	0.000148457	0.346180676
cg07102705	HTR4;HTR4;HTR4;HTR4;HTR4;HTR4;HTR4	0.000233422	0.346180072
cg15606473		0.000791389	0.346172683
cg11705975	PRLHR	1.96E-05	0.346145287
cg01202197	FADS6	2.58E-05	0.346143724
cg02136596	ATAD3C	1.96E-05	0.346136881
cg10521638	CXCL1	8.60E-06	0.346082363
cg23237183	NOTO;NOTO	0.000187194	0.346081978
cg24228329	CHST9	8.60E-06	0.346078991
cg12026729	LOC100192426;PTPRM;PTPRM	0.000357885	0.346066757
cg01085837		0.000654035	0.346014369
cg26381364	C12orf42;C12orf42	0.001948248	0.345994043
cg20691140		0.00043997	0.345993727
cg07923686	BHLHE22;LOC401463	3.34E-05	0.345980159
cg22574802	FGF10	0.000148457	0.345952894
cg10283958	KDELC1;BIVM;KDELC1;BIVM	7.23E-05	0.345946225
cg06353428	MARVELD3;MARVELD3;MARVELD3;MARVELD	9.24E-05	0.345945747
cg22755445	IL1R1	2.58E-05	0.345941752
cg01535080		8.60E-06	0.345939851
cg12864853	TPBG;TPBG;TPBG	0.000148457	0.345939689
cg15270892	EOMES	0.000148457	0.34592128
cg08235864	IRX2;IRX2;C5orf38	0.003734675	0.345909836
cg01737026	GRIK2;GRIK2;GRIK2	0.000148457	0.345905339
cg06593258	OSBPL1A;OSBPL1A	0.0050758	0.345903898
cg08582701	A2BP1;A2BP1	8.60E-06	0.345881219
cg12610207	TBX5;TBX5;TBX5;TBX5	0.000357885	0.345878879
cg08664487	BEND6;DST	0.000290372	0.345863627
cg01461824		2.58E-05	0.345859322
cg10762882		0.000357885	0.345856198
cg01428589	RGS9BP	0.000955793	0.345824798
cg07809484		5.59E-05	0.345821088
cg04361551		0.001376172	0.34578528
cg17310600	MARCH1	1.11E-05	0.345780464
cg21296676	EYA4;EYA4;EYA4	0.000148457	0.345775176
cg05707714		0.000117986	0.345762757
cg16620526	LVRN	9.24E-05	0.345762662
cg10529075	NPAS2	0.00043997	0.345762304
cg14445366		4.34E-05	0.345761815
cg11537406	SLC37A2;SLC37A2	1.96E-05	0.34574592
cg01479187		0.000791389	0.34573322
cg16922058	RAD21L1	9.24E-05	0.345724089
cg06018401	MAP1B	0.000187194	0.345722449
cg13419390	FAM123C;FAM123C;FAM123C;FAM123C	4.34E-05	0.345708746
cg27619291	TRPA1	9.24E-05	0.345688227
cg00053393	MGC45800	8.60E-06	0.345686931
cg16173191		4.34E-05	0.345674427

cg16452678	ANKRD5;ANKRD5	0.001147425	0.345668157
cg19761480	PPP2R3A	4.34E-05	0.345650507
cg05062612	WSCD2	0.000654035	0.345648265
cg16256390	BCL11B;BCL11B	0.003189589	0.345640894
cg11848173	CD63;CD63	0.000357885	0.345640414
cg18624636	SLC16A12	0.000791389	0.345638862
cg04157263		3.34E-05	0.345636556
cg02816003	RFX6	1.96E-05	0.345631039
cg10874314	PTPRE	3.34E-05	0.345619428
cg16999254	RGR;RGR;RGR	9.24E-05	0.345605167
cg06870213	HMGCLL1;HMGCLL1	5.59E-05	0.34557991
cg13276306	ANGPT1	4.34E-05	0.345576954
cg05499148		2.58E-05	0.345548634
cg00840960	HTR4;HTR4;HTR4;HTR4;HTR4;HTR4;HTR4	0.000654035	0.345529236
cg04622802	FIBIN;FIBIN	2.58E-05	0.345527053
cg20038996		1.50E-05	0.345510109
cg11146691	SLC38A4;SLC38A4;SLC38A4;SLC38A4	0.001147425	0.345493434
cg03669797		0.000290372	0.345493265
cg19744587	IL17RB;CHDH;CHDH	0.000117986	0.345481614
cg21735068	PGCP	4.34E-05	0.345468491
cg01172640		0.000117986	0.345465376
cg27146050	HIF3A;HIF3A	1.96E-05	0.345447226
cg08023265		1.11E-05	0.345445081
cg02273846	C14orf23;C14orf23	0.000148457	0.345441879
cg21144928		3.34E-05	0.345437291
cg05644602	C2orf65	1.96E-05	0.345428189
cg00192026		1.18E-05	0.345426048
cg06154313	RBP4	1.11E-05	0.345417447
cg09885622	LPCAT1	0.003734675	0.345403125
cg12457773	NRSN1;NRSN1	8.60E-06	0.345397214
cg01014615	HOXD3	2.58E-05	0.34538682
cg06558014		0.00043997	0.345384365
cg13729210		0.002304201	0.3453467
cg16197717	CPXM2	8.60E-06	0.345340016
cg08322034	ELOVL4	3.34E-05	0.345331029
cg14901671		8.60E-06	0.345322797
cg04832767	DLX6AS	0.000791389	0.345319981
cg09721739	NMNAT3	0.002714607	0.345312447
cg01394781	ABCC1;ABCC1;ABCC1;ABCC1;ABCC1	0.000290372	0.345300368
cg24100636	PRMT8	0.000290372	0.345297014
cg09663111	C20orf85	4.34E-05	0.34527416
cg18371769	PCDHGB4;PCDHGA4;PCDHGA6;PCDHGA1;PC	0.000537905	0.34523325
cg15997906	FAM183A	7.23E-05	0.345226135
cg06166242	SASH1	4.34E-05	0.345225702
cg02386859	CPE	8.60E-06	0.345225047
cg05613447	GPR120	2.58E-05	0.345198007
cg00048832	GABRA1;GABRA1;GABRA1;GABRA1;GABRA1	4.34E-05	0.345183783
cg11542086	IL7	5.59E-05	0.345166391
cg01968493	ZNF804A	5.59E-05	0.345165027
cg27174108	C19orf33;YIF1B;YIF1B;YIF1B;YIF1B;YIF1B;YIF1	1.50E-05	0.345156181
cg12560527	LOC222699	9.24E-05	0.345136496
cg04101351	PDE3A	3.34E-05	0.345135697
cg05954120	TMEM79;TMEM79	0.000148457	0.345132467
cg08557970	RPS6KA2;RPS6KA2	0.000357885	0.345097838
cg08628635	E2F3	0.001948248	0.345089163
cg00725089		2.58E-05	0.345083924
cg25741452	KITLG;KITLG;KITLG;KITLG	2.58E-05	0.345068536
cg20557801	LBXCOR1	4.34E-05	0.345062548
cg19155932	C20orf85	3.34E-05	0.345050227
cg08737836	DPYSL3	7.23E-05	0.345042553

cg21071896	AP3B2	4.34E-05	0.345012383
cg26000554	MOSC2	0.0050758	0.344999654
cg24697460	NCOR2;NCOR2	1.50E-05	0.344996638
cg19005485	BDH1;BDH1;BDH1	0.000117986	0.344988244
cg13848598	ADRB1	1.11E-05	0.344982853
cg01759136		1.96E-05	0.344955332
cg00673286	AP3B2	2.58E-05	0.344937033
cg19593767	TOX2;TOX2;TOX2	4.34E-05	0.344926819
cg14542554		7.23E-05	0.344916757
cg01872077	CYP27A1	0.000187194	0.344916361
cg26647139	CADM1;CADM1	1.96E-05	0.344901712
cg15345206		1.11E-05	0.34489983
cg25043129	BAIAP2;BAIAP2;BAIAP2;BAIAP2	2.58E-05	0.34489962
cg08952844		1.11E-05	0.344899207
cg01066472		0.000233422	0.344886513
cg00155310	TFAP2B	0.000148457	0.344871433
cg14236735		0.000187194	0.34486765
cg10367023		0.00043997	0.344839165
cg07104652	LPHN2	7.23E-05	0.344819666
cg11881754	SRRM3	7.23E-05	0.344805368
cg14921939	MATN4;MATN4;RBPJL;MATN4	1.96E-05	0.344804165
cg17381377	ABCC8	9.24E-05	0.344780142
cg04002794	FNDC3B;FNDC3B	0.000654035	0.344777627
cg17609931		0.000148457	0.344747879
cg19708803		7.23E-05	0.34474482
cg21406075		3.34E-05	0.344740757
cg09130288	PMP22;PMP22	1.96E-05	0.344730111
cg00419314	CXCL1	2.80E-05	0.344719707
cg14311670	SOX2OT	0.000290372	0.344717139
cg00030774	FAM189A1	3.34E-05	0.344715968
cg04105282	CRTAC1	0.000290372	0.344712588
cg05255811	KCNK5	0.000357885	0.344700975
cg11626496		1.11E-05	0.344688581
cg02067405	SCNN1G	0.000654035	0.344683958
cg20925233	NXPH3	0.002304201	0.344681724
cg13321166	MS4A3;MS4A3;MS4A3	0.000117986	0.344667395
cg16406892	TBX3;TBX3	0.002714607	0.344659074
cg23500059	CNTN1;CNTN1	0.000148457	0.344649707
cg12942414	EPCAM	0.000148457	0.344648033
cg18366209	CLVS2	1.11E-05	0.344640146
cg04066885	SH3BP4	2.58E-05	0.34464008
cg05118482	SCNN1G	0.000537905	0.344630042
cg18446336	GNA12	0.00043997	0.344619972
cg17266282		0.006823935	0.344619104
cg23478547		2.58E-05	0.344615325
cg09746494		8.60E-06	0.344598759
cg16583552	PCDHB7	1.50E-05	0.344592331
cg00736299	MGRN1;MGRN1;MGRN1;MGRN1	1.96E-05	0.344581425
cg04897804	SOX30;SOX30	8.60E-06	0.344565027
cg22199216		0.000537905	0.344561843
cg14214262	TBX5;TBX5;TBX5;TBX5	0.000148457	0.344559944
cg03771840	TRIM15	0.000187194	0.344557127
cg11511443	LMO3	3.34E-05	0.344550767
cg05485137	MKX	0.000654035	0.344542349
cg10722226		1.50E-05	0.344534476
cg04955573	GFI1;GFI1;GFI1	5.59E-05	0.344510869
cg23123250	SPNS2	0.000654035	0.344490392
cg15184602	FEZ1;FEZ1	0.000955793	0.344489391
cg24882875	HS6ST3	4.34E-05	0.344481604
cg03224572	GDF6	0.000148457	0.344474956

cg26933866		4.34E-05	0.34447147
cg22065923		8.60E-06	0.344462441
cg06119894	GRIA4;GRIA4;GRIA4;GRIA4	0.000791389	0.344449407
cg05213896	IL4I1;IL4I1	0.000955793	0.344445215
cg10126181	FLJ22536	0.002714607	0.344428148
cg21471199	ALDH4A1;ALDH4A1;ALDH4A1	0.002714607	0.344420616
cg04064644		5.59E-05	0.344412534
cg08159291	AJAP1;AJAP1	0.000955793	0.344408465
cg18161890	KRT72;KRT72;KRT72;KRT72;KRT72;KRT72	3.34E-05	0.344384916
cg06423617		1.50E-05	0.344365768
cg07003920		0.000290372	0.344359941
cg01435344	GOLGA7B	0.000357885	0.344321694
cg26584545	NTN4	2.58E-05	0.34431192
cg03309393		0.000233422	0.34430813
cg18092314	ROR1;ROR1	0.001376172	0.344279533
cg02807859		0.000187194	0.344254335
cg11869862		8.60E-06	0.344245286
cg03865648		0.000705485	0.344229056
cg05669418		3.34E-05	0.344225855
cg13984330		2.58E-05	0.344209552
cg16102040	ZNF827	3.34E-05	0.344204953
cg24873464		1.50E-05	0.344200475
cg24694675		0.000117986	0.344199671
cg06241792	C12orf39	0.003189589	0.344173069
cg26534938		5.59E-05	0.344169206
cg05477834	PCDHB17	7.23E-05	0.344157742
cg26886462		1.50E-05	0.344149844
cg07067241	TRPC1	0.000791389	0.344135298
cg18661205	PCDHB18	0.000791389	0.344130539
cg05684528		0.001147425	0.344110953
cg22322562	NRXN1;NRXN1;NRXN1	7.23E-05	0.344098608
cg23114616	TNFRSF25;PLEKHG5;TNFRSF25;PLEKHG5;PL	0.000290372	0.344095523
cg19448837	MAPK4	0.000290372	0.344082104
cg03970229	MTNR1B	4.34E-05	0.344066735
cg07094915		0.002714607	0.344058309
cg02895639	FAM19A5;FAM19A5	0.007873393	0.344052187
cg24531255	TFPI2;TFPI2	0.000357885	0.344046789
cg22751696	C3orf14	0.000117986	0.344032861
cg10989634		1.11E-05	0.344023545
cg18007463	TLL1	5.59E-05	0.344016618
cg04508482	IDUA	0.000537905	0.344002214
cg23374700	PARD6G	0.000791389	0.343998043
cg02818189		0.000290372	0.343977273
cg22346476		0.000233422	0.343973968
cg15278109		5.59E-05	0.343972313
cg04553838	TRPC6	0.000117986	0.343940394
cg00347620	TBX3;TBX3	8.60E-06	0.343937714
cg09285795		1.11E-05	0.343924022
cg18566984	PACRG;PACRG;PARK2;PARK2;PACRG;PARK	0.000233422	0.343919125
cg26233331	S100P;S100P	0.000187194	0.343907385
cg06369992		1.50E-05	0.343889469
cg00360414	TMEM26	5.59E-05	0.343886099
cg16783744	DPYS	0.000290372	0.343878114
cg20232102	PPP1R14A	1.11E-05	0.343849134
cg21477176	CACNG7	8.60E-06	0.343834444
cg23267550	RAB32	0.000233422	0.343823026
cg02239862	SUSD5	0.000654035	0.343822556
cg23839136	WBSCR17	7.23E-05	0.34382226
cg07241568	ABO	3.34E-05	0.3438137
cg05490712	PAX6	0.000117986	0.343778041

cg20498859	GPR149	7.23E-05	0.343775947
cg11509341	FAM176A;FAM176A;FAM176A	0.000290372	0.343757714
cg03960951	RFX4	3.34E-05	0.343742744
cg13974394	IRX4	4.34E-05	0.343730304
cg12796028		0.000148457	0.343726716
cg12994228		0.00043997	0.343711485
cg13740339	FBP1;FBP1	5.59E-05	0.343674408
cg24926361	GSC2	8.60E-06	0.343673102
cg10780164	CALY	9.24E-05	0.343669167
cg16714055	GATA5	0.00156564	0.343639779
cg04739123		0.000187194	0.343638526
cg03614916	IMPACT	0.00043997	0.343636641
cg05885936		9.24E-05	0.343630619
cg07953890	MACROD2	0.00043997	0.343630411
cg08217024	MDGA2	0.0050758	0.343630349
cg00065215	PAX7;PAX7;PAX7	0.000866489	0.343629315
cg07194093	PTPRM;PTPRM	0.000357885	0.343626931
cg13729816		1.50E-05	0.343626331
cg15000232	PNPLA3	4.34E-05	0.343620595
cg07701191	RORA	0.000117986	0.343614868
cg21586152	ELANE	4.34E-05	0.343611087
cg15353031	C10orf53;C10orf53	8.60E-06	0.343606662
cg16585651		0.001376172	0.343599305
cg12129117	CAPN2;CAPN2	0.000117986	0.343596829
cg00114029	DLGAP3	0.000158823	0.343592177
cg09643312	CD302	0.004357816	0.343576099
cg02291010	CAST;CAST;CAST;CAST;CAST;CAST;CAST;C/	2.58E-05	0.343550015
cg24921943	SH3PXD2B	0.002304201	0.343548637
cg00707317	NMNAT2;NMNAT2	9.24E-05	0.343535998
cg14415816	RFX4	1.50E-05	0.343503341
cg17873451	LOC440925;SP5	0.000187194	0.343484004
cg06995920	DPY19L2	0.000233422	0.343462571
cg03875330	TMEM205;TMEM205;CCDC159;TMEM205	0.000148457	0.343460744
cg26278103	GPR37	1.96E-05	0.34346057
cg14475702	KLF4	7.23E-05	0.343448866
cg23514532	SLC2A10	0.000187194	0.343404631
cg24374583	MOSC1	0.000233422	0.343393408
cg26356061	ZNF418	8.60E-06	0.343386754
cg17307442	EMX2;EMX2;EMX2OS;EMX2;EMX2	0.001393182	0.343356112
cg10767045	MINPP1	9.24E-05	0.343349682
cg02605760	NSUN5;NSUN5;NSUN5;NSUN5	7.23E-05	0.343346858
cg19513582	UBE2H;UBE2H	7.23E-05	0.343346031
cg07234958	GPC6	0.000148457	0.3433365
cg14176752		8.60E-06	0.343334124
cg00960305	C20orf56	0.002714607	0.343315686
cg08691235	TTN;TTN;TTN;MIR548N;TTN	3.34E-05	0.343311893
cg09745243	CXCL2	0.001948248	0.343298257
cg16260421	AGBL4	8.60E-06	0.34329757
cg14470895	CDO1	4.34E-05	0.343293018
cg03203197		0.000117986	0.343290422
cg18297437	ZIC1	0.00043997	0.343288271
cg25137687	CXCL3	0.000537905	0.343279818
cg16146177		0.000148457	0.343253757
cg02288301	TMEFF2	7.23E-05	0.343250837
cg16422316	AP1G1;AP1G1	0.000117986	0.34323733
cg13427361		9.24E-05	0.343234729
cg07202413		2.58E-05	0.343234378
cg20386580	NEURL	8.60E-06	0.343229946
cg09698548	CA8	0.000955793	0.343227806
cg01272400	GPR120	0.000357885	0.343222568

cg11166759		0.000357885	0.343194773
cg17097923	FBXO39	5.59E-05	0.343191045
cg26934243	LRIG3;LRIG3	8.60E-06	0.343184585
cg08805144	CACNA2D3	0.001147425	0.3431797
cg02044223		0.000148457	0.343160618
cg04209913	GDNF;GDNF;GDNF	0.006738759	0.343156586
cg04882233		0.000955793	0.343153932
cg16844053	CUL1	0.000187194	0.343153191
cg05463966	NDUFS8	4.34E-05	0.343126179
cg02793099	PEG3;PEG3;ZIM2;PEG3;PEG3;ZIM2;ZIM2	1.11E-05	0.343125398
cg01719210	VAX2	0.000187194	0.343119933
cg22793735	PLOD2;PLOD2	0.000357885	0.343117453
cg18010131	AMPD3;AMPD3;AMPD3	4.34E-05	0.343104885
cg21806015	NXPH1	4.34E-05	0.34310429
cg08292467	PCDHGA4;PCDHGA6;PCDHGA9;PCDHGA1;PC	4.34E-05	0.343091312
cg21390624	EYA4;EYA4;EYA4	0.000233422	0.343091022
cg14795750	PCDH10;PCDH10;PCDH10;PCDH10	1.11E-05	0.343087666
cg17977173	HS6ST1	8.60E-06	0.343058195
cg12281565	ERBB4;ERBB4	1.50E-05	0.343048402
cg22488158	WDR63;WDR63	1.96E-05	0.343042452
cg04831327	RBM19;RBM19;RBM19	0.000233422	0.34299397
cg02705835	CCDC71	0.007873393	0.342989305
cg04239994	HRNBP3	0.000233422	0.342988109
cg00105628		2.58E-05	0.342968097
cg15250797	SCTR	1.11E-05	0.342964435
cg09571713	SPOCK3;SPOCK3	1.50E-05	0.342951079
cg02112168	PRPF39;SNORD127	4.34E-05	0.342947156
cg02304370	PHRF1	7.23E-05	0.342938644
cg22459739	PCDHA2;PCDHA1;PCDHA1;PCDHA3;PCDHA4	7.23E-05	0.342930812
cg18026588	GABRB3;GABRB3	4.34E-05	0.342929603
cg26220018		0.002714607	0.342929399
cg02583151	SIX1	1.11E-05	0.342923302
cg13875133	PAPPA	4.34E-05	0.342912061
cg25632105	CHRM2;CHRM2;CHRM2;CHRM2;CHRM2;CHRI	9.24E-05	0.342904631
cg18929903	ENTPD7	0.000187194	0.342883229
cg12746356	HTRA1	0.000537905	0.342832246
cg13675859	GPR110;GPR110	0.001948248	0.342823166
cg20541656	LAPTM4B	2.58E-05	0.34281586
cg04906989	C17orf102;TMEM132E	0.000654035	0.342813118
cg03104428	OLFM3	0.001376172	0.342799372
cg10899768	GRHL2	0.000654035	0.342790237
cg13544851	DPP6;DPP6;DPP6;DPP6	8.60E-06	0.342787454
cg05722504	PHOX2B	1.96E-05	0.342774725
cg10111115	PDZRN3	3.34E-05	0.342767759
cg18397975	AMACR;AMACR;AMACR;AMACR;AMACR;AMA	1.50E-05	0.342766521
cg11808581	GABRD	0.001948248	0.342741421
cg06410191	LVRN	1.50E-05	0.342736117
cg17496659	TP73	0.000233422	0.342728879
cg26351229	ADCY8	1.11E-05	0.342720654
cg10364040	HOXD10	0.000654035	0.342705092
cg00714740		0.000187194	0.342690361
cg27106513	RGS20	0.000187194	0.342683466
cg14234426		4.34E-05	0.342671457
cg15120341	TFDP1;TFDP1	4.34E-05	0.342660672
cg18587476	C2orf84	4.34E-05	0.342659469
cg06740950	FNDC3B;FNDC3B	0.000187194	0.342648696
cg19676182	CCDC149;CCDC149	0.003189589	0.3426251
cg19496491	TEAD1	0.002304201	0.342618039
cg08317058		2.58E-05	0.342616924
cg18261205		0.000187194	0.342590515

cg01405761	CLVS1;CLVS1	1.50E-05	0.342573088
cg16945312	FOXF2	0.000187194	0.342572516
cg24874180	DKK2	2.58E-05	0.342571506
cg22758454		5.59E-05	0.342568504
cg03352189	CCDC36;CCDC36	2.58E-05	0.342552034
cg21454485	CD84	0.009066563	0.342543099
cg06271970		7.23E-05	0.342529335
cg27107171	PRR16	0.004357816	0.342525171
cg21733531		4.34E-05	0.342515754
cg16976662	PLCE1	1.50E-05	0.342476336
cg22511947	FN1;FN1;FN1;FN1;FN1;FN1;FN1	1.96E-05	0.342465994
cg14866863		6.94E-05	0.34245809
cg00735923	CFTR	0.000122184	0.342456976
cg25005894	ERC2	0.000187194	0.342455217
cg18834712		1.11E-05	0.34244956
cg15373239	EMX2OS;EMX2;EMX2	1.96E-05	0.342442684
cg26928418	ZNF578	0.000233422	0.34243691
cg12078031	ADAMTS5	8.60E-06	0.342386481
cg19300568	NAT8L	1.50E-05	0.342385701
cg01787285	SKI	0.0050758	0.342383088
cg13794530	VIPR2;VIPR2	0.004357816	0.342378855
cg08151596		2.58E-05	0.342375516
cg06319822	HBM	0.000290372	0.342339794
cg25605545		9.24E-05	0.342331108
cg17154729	UTP23	0.000537905	0.342319336
cg27595860	SCRT1	7.23E-05	0.342316784
cg08708747	EPHA6	0.000187194	0.342312925
cg13582072	FAM155A	1.96E-05	0.342303185
cg03113285	ENPP4	0.009066563	0.342283357
cg01992382	TNXB	0.000117986	0.342281072
cg03807298		0.000187194	0.342276875
cg16672562	HIF3A;HIF3A;HIF3A	3.92E-05	0.342272938
cg19716238	FAM83B	4.34E-05	0.342272009
cg06750410	ANKRD55;ANKRD55	4.34E-05	0.342264893
cg15879154		0.001147425	0.342262736
cg08876441	FAM5C	0.001376172	0.342259275
cg17583413	NEUROG3	4.34E-05	0.342258902
cg02976699		8.60E-06	0.342248317
cg06880612		0.000117986	0.342247947
cg13722123	GRM1;GRM1	8.60E-06	0.342229575
cg08384637	FOXC2	0.000148457	0.342228002
cg09588271	KANK4	0.000148457	0.342211835
cg00195796	SOX2OT	7.23E-05	0.342201646
cg18250846		4.34E-05	0.342191053
cg05635730		1.50E-05	0.342182854
cg27589088		0.000946184	0.34217512
cg08621473		3.34E-05	0.342156837
cg00856157	UGT8;UGT8	4.34E-05	0.342146113
cg02158486	MAPK4	1.11E-05	0.342134342
cg04213390		0.001147425	0.342131198
cg01660999	ANGPT1	0.000233422	0.342129882
cg00257047		0.000233422	0.342123217
cg16989059	ROBO1	0.000117986	0.342114262
cg08424188	BCAR1;BCAR1;BCAR1;BCAR1;BCAR1;BCAR1	1.50E-05	0.342094083
cg11543397	MAL2	0.000233422	0.342085446
cg04748834	CHL1	0.001639597	0.342080976
cg03782202	HOXD11	5.59E-05	0.342080947
cg08829841	FOXI2	1.11E-05	0.342060123
cg11037576		0.000187194	0.342056609
cg23276120	MEGF11	8.60E-06	0.342037421

cg21685427	SGK2	1.96E-05	0.342009229
cg08364137		4.34E-05	0.342005722
cg26083180		2.58E-05	0.341999593
cg03430923	C10orf107	7.23E-05	0.341995325
cg22007163		7.23E-05	0.3419791
cg01428678	GPHN;GPHN	0.000290372	0.341965585
cg13265789	UNC5C	0.000955793	0.341953341
cg22158650		0.002714607	0.341948054
cg12297221	ART5;ART5;ART5	9.24E-05	0.341941468
cg14714629	TRIM4;TRIM4	0.00043997	0.34191721
cg02639634	RGS20;RGS20	5.59E-05	0.341887578
cg21005412	WDR43;SNORD53	7.23E-05	0.341886869
cg27220070	EP400	0.001639597	0.34187748
cg10490742		8.60E-06	0.341855613
cg20109856	DLX6AS	0.000654035	0.341826274
cg01554410	IRX6	8.60E-06	0.341824829
cg14642432	A2BP1;A2BP1	7.23E-05	0.34181475
cg04779631	SH3GL3;SH3GL3	7.23E-05	0.341813289
cg13891220	PITX2;PITX2	0.000148457	0.341790959
cg15824707	DUOX2	1.96E-05	0.341788275
cg21875330	PAX9	2.58E-05	0.341782176
cg15406010		1.96E-05	0.341775561
cg00340850		1.96E-05	0.341764287
cg09310891	CLEC4GP1	0.000233422	0.341757695
cg07879785	CRYL1	9.24E-05	0.341756201
cg05124129	CPNE8	0.001147425	0.341734851
cg17278864	RICH2	0.000148457	0.341716738
cg18910324	FAM46A	0.001147425	0.34171362
cg16964946	LGR4	5.59E-05	0.341701405
cg01446894	PAX1	1.11E-05	0.341700577
cg14186641	CNR1;CNR1;CNR1;CNR1	0.003189589	0.341691943
cg02334926	RGS7BP	5.59E-05	0.341682929
cg15638055	KCNV1	3.34E-05	0.341678856
cg06095270	PRDM13	8.60E-06	0.341669652
cg04265797	SSTR1	1.11E-05	0.341646567
cg05861971	NPAS2	0.000537905	0.34162413
cg15084803	CHCHD4;CHCHD4	0.001147425	0.341615694
cg11665991		7.23E-05	0.341579553
cg21701379	TSPAN11	8.60E-06	0.341573848
cg14928902	PRDM14;PRDM14	7.23E-05	0.341557901
cg12695586	OXTR	3.34E-05	0.341542242
cg00789960	MNAT1	0.00043997	0.341525082
cg03905758	HAND2;NBLA00301	7.23E-05	0.34151195
cg06294475	KCNQ1;KCNQ1OT1;KCNQ1	0.00043997	0.341460358
cg00377344		0.001639597	0.341439927
cg03766264	STK38L	0.000537905	0.341434263
cg22282410	PTPRN2;PTPRN2;PTPRN2	4.34E-05	0.341432957
cg26387689	PDE3A	0.000148457	0.341431781
cg02441647	COL8A1;COL8A1;MIR548G;COL8A1;COL8A1	2.58E-05	0.341406637
cg08554257	NOD2	0.009066563	0.341402257
cg26702958	FLJ41350	3.34E-05	0.341379511
cg04562909	TBX5;TBX5;TBX5;TBX5	1.50E-05	0.341379319
cg07392385	SORCS1;SORCS1	8.60E-06	0.341366546
cg14306650	RALGPS1	0.000187194	0.341354301
cg12978105		0.00043997	0.341349579
cg01100175	KIF5C	5.59E-05	0.341346702
cg05905988	FAM155A	8.60E-06	0.341344943
cg00710862	TGOLN2	9.24E-05	0.341309946
cg14980983	GATA5	0.001639597	0.341298628
cg18267374	NEFM;NEFM;NEFM	8.60E-06	0.341290284

cg08252579	FEZF2	0.001147425	0.341285573
cg17627654	SHANK2;SHANK2	0.000290372	0.341275587
cg15426956	RGS7BP	5.59E-05	0.341275529
cg22185451	C7orf13;RNF32	0.000654035	0.341269332
cg12258161	MAGI2	8.60E-06	0.341267566
cg24720355	TBX15	3.34E-05	0.341266804
cg23260578	RNASE10	7.23E-05	0.341263934
cg17708016		0.000205664	0.34124128
cg04013024		1.96E-05	0.341235516
cg03580065		0.00043997	0.341235109
cg26820037	FGF8;FGF8;FGF8;FGF8	9.24E-05	0.341230346
cg08413427	DBF4B	1.96E-05	0.341226297
cg14347088	LRFN5	9.24E-05	0.341208562
cg06552356	ALX1	1.11E-05	0.341208353
cg23550826	PDGFRA;PDGFRA	0.000187194	0.34118976
cg04982401	DLC1;DLC1	0.000187194	0.341167188
cg21737226		1.96E-05	0.34114942
cg10171125	SCARA5	4.34E-05	0.341141491
cg13366352	GREB1L	0.000148457	0.341140968
cg08690634		0.000148457	0.341134731
cg05701403	PLEKHA7	0.000117986	0.341133594
cg18421710		2.80E-05	0.341129613
cg15725584		1.11E-05	0.341128327
cg00221745	FBXL15;PSD	0.000654035	0.34112071
cg12492094	PDE11A;PDE11A	0.000654035	0.341114157
cg12481212		0.000955793	0.341103934
cg24461964	KIF5C;KIF5C	3.34E-05	0.341083841
cg20337996	FAM101A	0.000955793	0.341082558
cg00574530	NEUROG1	0.000357885	0.341076112
cg17146570	EDN3;EDN3;EDN3;EDN3	0.005897668	0.341074796
cg22855933		0.000537905	0.341063742
cg24595261	RAB32	0.000233422	0.341063111
cg11870037	SASH1	1.50E-05	0.341034449
cg07052231	PEX5;PEX5;PEX5;PEX5;PEX5	2.58E-05	0.341028198
cg12082609	MEIS1	0.000290372	0.341025139
cg00009088	VPS37C	0.001376172	0.341023987
cg04464122		0.000357885	0.341007405
cg12253830	ST8SIA1	0.000148457	0.341005322
cg26667720		0.000187194	0.340968977
cg21319932		1.96E-05	0.340965168
cg11752769	CR1L	0.000187194	0.340955207
cg14140379	S100P	1.96E-05	0.340949996
cg08084860		0.000187194	0.340946538
cg25461801		0.002304201	0.34093773
cg17865533	MAPK4	9.24E-05	0.340933493
cg05484788	PHACTR3	7.23E-05	0.340925725
cg04093633	EFTUD1;EFTUD1	0.00043997	0.340915473
cg13622546	ASB6;ASB6	7.23E-05	0.340912338
cg18301891	DIO3;MIR1247	0.007869847	0.340890214
cg05779148	LIMCH1;LIMCH1;LIMCH1;LIMCH1;LIMCH1	3.21E-05	0.340883821
cg11011938	SEMA5A;SEMA5A	0.000654035	0.340863653
cg23644960	TRPC6	0.000654035	0.340861714
cg03836615	ITPR1;ITPR1;ITPR1	7.23E-05	0.340842937
cg13329912		3.34E-05	0.34083675
cg14307693		0.002304201	0.340836301
cg14490882		0.000233422	0.34082787
cg16909408		0.002714607	0.340825251
cg18087477	SYCP1;SYCP1	5.11E-05	0.340811704
cg12534424	PRRT4	1.96E-05	0.340810514
cg10397440	PENK;PENK;PENK	3.34E-05	0.340804617

cg12806763	HOXB4	0.000233422	0.340800725
cg25400396	ZNF814	4.34E-05	0.340784186
cg23196831	COL14A1;COL14A1	0.000117986	0.340781481
cg15493618		8.60E-06	0.340769029
cg02969426	KCTD4;GTF2F2;KCTD4	1.11E-05	0.34074486
cg11052143	ALS2CR11;ALS2CR11;ALS2CR11;ALS2CR11	1.50E-05	0.340744011
cg04395689	ATP6V1B2	0.00043997	0.34073752
cg24136205	ZIC5	5.59E-05	0.340734156
cg14742716	C19orf41	5.59E-05	0.340718906
cg25034395	HPSE2;HPSE2;HPSE2;HPSE2	5.59E-05	0.340708199
cg08614290	VIPR2	0.00043997	0.340707138
cg11345918	HCCA2;LOC338651	2.58E-05	0.340703393
cg12103152	HTR1B	0.000233422	0.340702949
cg25678088	DLX6AS	1.50E-05	0.340673879
cg22108469		0.000117986	0.340647996
cg12894449	EMX2OS;EMX2;EMX2	0.000117986	0.340637791
cg05710753	AGTR1;AGTR1;AGTR1;AGTR1	0.000117986	0.340637596
cg07921712		2.58E-05	0.340617544
cg17980508	IFI44L	0.000537905	0.340617415
cg04858155	ALX4	1.50E-05	0.340581377
cg23300659	CHRM2;CHRM2;CHRM2;CHRM2;CHRM2;CHRI	2.58E-05	0.340577897
cg24310461	C1orf115	0.003189589	0.340575957
cg24043916		7.23E-05	0.340563453
cg08886973	LOC441177;C6orf176;C6orf176	5.59E-05	0.340551058
cg15739581	GALNT3	0.000357885	0.340531009
cg27499860	MAPK15;MAPK15	1.11E-05	0.340530243
cg04987608	BCAR1;BCAR1;BCAR1;BCAR1;BCAR1	4.34E-05	0.34051309
cg15959252	C12orf68;C12orf68	0.000187194	0.340496855
cg23677657	DLX6AS	4.34E-05	0.34049009
cg24295381	EBF2	7.23E-05	0.340483161
cg06776588		0.000955793	0.340482747
cg03829464		1.11E-05	0.340479347
cg18944752	RGS12;RGS12;RGS12	0.000290372	0.340478523
cg07625194	TFAP2D	4.34E-05	0.340477081
cg08638184		5.59E-05	0.340453052
cg20991421	HTR1E	8.60E-06	0.340445181
cg02361458	PRKAA2	8.60E-06	0.340421485
cg21158633	UNCX	0.00043997	0.340392843
cg01098320		2.58E-05	0.340385926
cg07072366	LPL	3.34E-05	0.340382678
cg01044849	NCRNA00171	1.96E-05	0.340365291
cg05858126	MIR146B	0.000654035	0.340338496
cg04837280	TMED9	0.001639597	0.340331571
cg21780859	MTUS2;MTUS2;MTUS2	4.34E-05	0.340322083
cg12062220		0.000233422	0.34031304
cg06917617	CR1L	5.59E-05	0.34030743
cg00782708	C2orf34	3.34E-05	0.340306004
cg02343823	ZNF300	0.002304201	0.340303312
cg01505987		2.58E-05	0.340280832
cg03911494	MARVELD3;MARVELD3	0.001393182	0.340279984
cg19594666	LEP	0.000148457	0.340278161
cg09059267		2.58E-05	0.340271915
cg24800880	ANK1	0.000233422	0.340265957
cg01241390	RNU5E;RNU5D;ZCCHC9;ZCCHC9;ZCCHC9	0.000233422	0.340239992
cg05715532	IHH	2.58E-05	0.340233209
cg14670435	MIR1258;ZNF385B	3.34E-05	0.340220371
cg24913349	LIFR;LIFR	2.58E-05	0.340194479
cg17432620		2.58E-05	0.340192805
cg09693811		0.001376172	0.340180533
cg13394305	SLC40A1	0.000955793	0.340170319

cg18026225	PLCD3	4.34E-05	0.340165726
cg05709770		0.000233422	0.340156083
cg24182831	TSNARE1	0.001639597	0.340144957
cg04257984	LOC440925	7.23E-05	0.340128322
cg19598584	HOXA2	0.001376172	0.340121694
cg15709235	ROBO1;ROBO1;ROBO1;ROBO1;ROBO1;ROBO	9.24E-05	0.340097353
cg00087368	SIM1	2.58E-05	0.340090692
cg19990182	WDR35;WDR35	0.000148457	0.340084532
cg08874035	KIAA1598;KIAA1598	3.34E-05	0.340082679
cg20571592		8.60E-06	0.340069324
cg04128045	IGSF21	9.24E-05	0.340064426
cg09160776		8.60E-06	0.34005663
cg16574134	PAX2;PAX2;PAX2;PAX2;PAX2	7.23E-05	0.340055265
cg20467168		0.003734675	0.340038514
cg14876117		0.000654035	0.34003555
cg17384889	NKAPL	0.000233422	0.340024082
cg16324121	IL17RE;IL17RE;IL17RE	0.001147425	0.340016661
cg07035165	KCNN2	1.11E-05	0.339987799
cg14045860		4.34E-05	0.339987756
cg15100426	PNKD;PNKD	5.59E-05	0.339986806
cg26295057	GDNF	0.002714607	0.339984914
cg01026458	NECAB1	8.60E-06	0.339981272
cg23986671	ADAMTS5;ADAMTS5	4.34E-05	0.339966682
cg22867063	ZNF214;NLRP14;ZNF214	0.000233422	0.339930525
cg06390651		0.000955793	0.339929615
cg14793406		1.96E-05	0.339929339
cg11960629	SLC16A12	0.000187194	0.339927828
cg26406563	C1orf175;C1orf175	3.34E-05	0.339913496
cg22991320	C20orf85	4.34E-05	0.339912351
cg12967001	EPB41L3	0.000158823	0.339888668
cg25033990	ACCN1	0.000357885	0.339878262
cg22187251	GCNT2	0.009066563	0.339840043
cg08044427		1.96E-05	0.339837043
cg09187338		5.59E-05	0.339834796
cg27649073	PITX3	0.000290372	0.339816518
cg01616926	PITX2;PITX2	0.000791389	0.339791868
cg14782015	CRB3;CRB3	2.58E-05	0.339790233
cg19585597	LOC134466	1.96E-05	0.339777512
cg00314003		8.60E-06	0.339770842
cg25536676	DHCR24	1.11E-05	0.339766465
cg02487453	OSBP2	0.000654035	0.339761387
cg01503348	SVEP1	9.24E-05	0.339755959
cg07267782	FAM83B	0.001291055	0.339749025
cg23302570	NXPH2	0.000290372	0.339743348
cg22029275	FAM123A;FAM123A;FAM123A;FAM123A	0.000955793	0.339729563
cg15491247	PRDM16;PRDM16	9.24E-05	0.339712282
cg15681577		1.11E-05	0.339690738
cg00004996	MTMR7	2.58E-05	0.339655523
cg16431436	VCAN;VCAN;VCAN;VCAN	0.003734675	0.33965547
cg17235702		2.58E-05	0.339647485
cg16514543		0.000148457	0.339635029
cg11145160	CLDN11	0.001887355	0.339620852
cg16306900	ZNF648	0.000290372	0.339603811
cg01181227		0.000117986	0.339599607
cg14713146		7.23E-05	0.339596886
cg03032497		0.000357885	0.339585345
cg09363735	TFAP2B	2.58E-05	0.339583037
cg08747377	CDH13;CDH13	5.59E-05	0.339581553
cg21771250	FAM83F	0.000290372	0.339572384
cg08012521		5.59E-05	0.339569664

cg15041550	GLP1R;GLP1R	0.000290372	0.339559915
cg12238343	RXFP3	7.23E-05	0.339556261
cg23685965	CYB5A;CYB5A	3.34E-05	0.339549055
cg18532076	FAM83B	9.24E-05	0.339548049
cg21216477	HOXC4	0.000148457	0.339545866
cg25307902	SNAP25;SNAP25	0.000537905	0.339542946
cg05674784	TBX18	2.58E-05	0.339513984
cg07143462		7.23E-05	0.339508726
cg18533386	CACNA1E	0.000791389	0.339507116
cg02264990	HOXC4;HOXC4	0.000233422	0.339491611
cg21293934	ANKRD30B	0.0050758	0.339483808
cg07318417	NMU	0.000148457	0.339479806
cg14188639	INS-IGF2;IGF2AS;IGF2;IGF2;IGF2AS;IGF2	2.58E-05	0.339476624
cg12070987	NDUFS8	3.34E-05	0.339467489
cg02901723	FAM190A	4.34E-05	0.339440268
cg09557556	KCNC1;KCNC1	0.000955793	0.339435402
cg01627252	CAPN14	0.000791389	0.339430763
cg25798409	ERBB4;ERBB4	1.11E-05	0.339414427
cg22851420	HPCAL4	4.34E-05	0.339405098
cg19235974		0.00043997	0.339392943
cg06821999		1.50E-05	0.339392792
cg04582294	GRID1	0.000233422	0.339388645
cg16915821	DKK3;DKK3;DKK3	0.000791389	0.339387855
cg00279001		1.96E-05	0.339386386
cg09191731	HS3ST2	1.50E-05	0.339367706
cg19498794	RASAL2;RASAL2	7.23E-05	0.339359539
cg26492368	SPAG6;SPAG6	3.34E-05	0.339355496
cg26913248	TWIST2	0.000357885	0.339350565
cg20283498	CDH23;CDH23	7.23E-05	0.339345776
cg20646280	KCTD8	1.11E-05	0.339334391
cg23587176	FOXK1	3.34E-05	0.339310569
cg06691542		0.000117986	0.339310076
cg06276708	AP3B2	2.58E-05	0.339305008
cg08064068		0.000187194	0.339301093
cg08424167		0.000290372	0.339288159
cg10243398	ROBO1	4.34E-05	0.339286763
cg09808235	MMEL1	0.000654035	0.339284515
cg14031402		3.34E-05	0.339268579
cg01176947		1.50E-05	0.339265802
cg20626645	CACNA1D;CACNA1D;CACNA1D	0.000791389	0.339265389
cg24364535	OPCML	4.34E-05	0.339262873
cg04355791	MIR129-2	0.000791389	0.339256115
cg14013195	C2orf39	2.58E-05	0.339250828
cg14427437		3.34E-05	0.339246351
cg24306982		0.000148457	0.339237214
cg01321673	IL17REL	2.58E-05	0.339231111
cg01263386	SLC8A2	0.000187194	0.339227017
cg05117208	STAT5A	0.000117986	0.33922506
cg02661802	HTRA1;HTRA1	0.000187194	0.339222191
cg14164099		0.000148457	0.339218446
cg17283453	ZNF177	0.000187194	0.339211589
cg19360330		7.23E-05	0.339205287
cg15391574	GLB1L2	1.96E-05	0.339202412
cg20183094		8.60E-06	0.339198336
cg24891539	SOX17	0.000357885	0.339193479
cg06288696	CRYGN;CRYGN	0.003734675	0.339187906
cg10725720	HCRTR2;HCRTR2	0.000187194	0.339182317
cg18616418		4.34E-05	0.339170414
cg12922751		4.34E-05	0.339159636
cg11463113	CRYGD	0.000537905	0.339159349

cg26766005	SP5;LOC440925;SP5	0.000233422	0.339158719
cg04743945	GRM3	0.000357885	0.339154287
cg20037575		9.24E-05	0.339152461
cg23257935	SPAG17;SPAG17	2.58E-05	0.339136818
cg05545441		0.000290372	0.33913325
cg24654525	GEFT;GEFT	5.59E-05	0.339124346
cg00548708		5.59E-05	0.339114714
cg10095016	DNAJC30;WBSCR22	1.50E-05	0.339103211
cg07732115	DYNLRB2	0.000537905	0.339099682
cg20720686	POR	0.000187194	0.339094649
cg17704343	KIF18B	9.24E-05	0.339081536
cg21611810	TBX5;TBX5;TBX5;TBX5	0.000290372	0.339080409
cg26375057	ZIC1;ZIC1	1.96E-05	0.339073714
cg22154024	ASCL1	0.000357885	0.339060919
cg25285090		5.59E-05	0.339047088
cg02367736	MOSC2	0.003189589	0.339046889
cg03285577		1.50E-05	0.339041704
cg23563008	DLX6AS;DLX6	0.009066563	0.339019091
cg14709292	NUDT16P;NUDT16P	1.96E-05	0.338984369
cg12005098	SLC16A12	0.000537905	0.338976364
cg09323727		0.002304201	0.338973784
cg00744413		1.11E-05	0.338936034
cg13628577	PRDM6	5.59E-05	0.338934249
cg09366312	TMEM200C	0.000290372	0.3388996
cg08523865	CNTNAP5	7.23E-05	0.338892116
cg24833674	ILDR2	0.000955793	0.33888161
cg01258200	ANKRD56	5.59E-05	0.338880403
cg04020984	LOC100270746;C6orf41	4.34E-05	0.33887638
cg09339194	GATA5	0.000654035	0.338870114
cg14737994	ZNF177	1.11E-05	0.338863848
cg00109356	SHISA2	9.24E-05	0.338848875
cg03023152	GRHL2	0.000233422	0.33884859
cg03209854	ADAMTS16	2.58E-05	0.338793331
cg22763649	PAX1	1.96E-05	0.338788971
cg12678667	MOGAT1	1.96E-05	0.338777972
cg17496887	S100A7A	4.34E-05	0.338776245
cg01283289	ACSS3	1.50E-05	0.338771644
cg22297146	MYEF2;MYEF2	0.001147425	0.338757153
cg02232208		4.34E-05	0.338745926
cg13361703	TMEM20;TMEM20	5.59E-05	0.338736446
cg23780110	ASXL2	0.005897668	0.338720507
cg02799411	ITPR1;ITPR1;ITPR1;EGOT	0.003734675	0.338706571
cg20470857	C1QL2	8.60E-06	0.338694012
cg08194009		0.00043997	0.338691343
cg17912370	C3orf55;C3orf55;C3orf55;C3orf55	0.000187194	0.338677934
cg05514256		0.000148457	0.338677425
cg02214623	GNB2L1	2.58E-05	0.338674358
cg27126555		1.11E-05	0.338671552
cg23825057	RILPL1	0.000117986	0.338664003
cg17343563	TFAP2B	0.000955793	0.338653218
cg27045356	NDFIP2;NDFIP2	0.000148457	0.338636412
cg15480095	IGSF9B	3.34E-05	0.338624775
cg08425678	PRDM2	0.000148457	0.338624105
cg04007303	FOXE1;FOXE1	4.34E-05	0.338615044
cg03764259		0.001147425	0.338610264
cg16276063	SOX2OT	0.000866489	0.338602345
cg00555036		1.50E-05	0.338578804
cg01247426	NELL2;NELL2;NELL2;NELL2;NELL2	0.000290372	0.338577106
cg07920503	FAM123A;FAM123A	0.000233422	0.338571793
cg25255850		2.58E-05	0.338553961

cg19589811	FAM181B	7.23E-05	0.338545689
cg00431549	MGP	0.004357816	0.338527983
cg07057579		1.50E-05	0.338492887
cg01656470	PEG3;PEG3;ZIM2;PEG3;PEG3;ZIM2;ZIM2	8.60E-06	0.338475497
cg27434954	GALNT11	1.96E-05	0.338466337
cg11760198	BRUNOL4;BRUNOL4;BRUNOL4;BRUNOL4	1.96E-05	0.338451748
cg14598976		5.59E-05	0.338444505
cg27586417		0.000233422	0.338434848
cg12877251	LOC157627	0.000654035	0.338428629
cg23022785		0.000537905	0.338417128
cg08022436	KCNJ3	8.60E-06	0.338416092
cg15059608	C3orf14	8.60E-06	0.338396868
cg13390630	CDH22	0.000233422	0.338385734
cg25149751	PPP2R2B;PPP2R2B;PPP2R2B;PPP2R2B;PPP2	0.000187194	0.338383236
cg08210322	C2orf72	4.34E-05	0.338378712
cg05694921	PTPRU;PTPRU;PTPRU	3.34E-05	0.338371471
cg25897519		0.000357885	0.338370619
cg08034077	CA10;CA10;CA10;CA10	5.59E-05	0.338369356
cg16234966	FAM84A	1.96E-05	0.338352209
cg19382919	LOC440925;SP5	0.000357885	0.338339794
cg10112407	AGAP1;AGAP1	0.000791389	0.338313451
cg07904452		0.004357816	0.338311818
cg23093609		1.96E-05	0.338293982
cg12610471	SPAG6;SPAG6	0.000148457	0.338264807
cg17944161	NOTCH3	0.000955793	0.338233175
cg06951565	GRM6	0.002304201	0.338229404
cg04988206	CT62;CT62	0.00043997	0.338229037
cg17959183	RAB10	0.000955793	0.338227591
cg23407265	RHOBTB1;RHOBTB1	0.000537905	0.338221859
cg00154119		1.50E-05	0.338216703
cg14200569	PRDM16;PRDM16	0.000654035	0.33821247
cg26896771		5.59E-05	0.338195743
cg26685195	SLC30A10	2.80E-05	0.338192427
cg14273450	C2orf39	0.000117986	0.338161542
cg04774711		3.34E-05	0.338147543
cg15070718	NR2F2;NR2F2;NR2F2;NR2F2;MIR1469	1.11E-05	0.338140619
cg12487540	PCDHGA2;PCDHGA4;PCDHGA1;PCDHGB1;PC	0.000233422	0.33812165
cg10557578	RAB11FIP1;RAB11FIP1	0.00043997	0.338116246
cg21356631		0.001376172	0.33810147
cg06833823	LOC441666	0.000262362	0.338098345
cg19117365	CLSTN2	2.58E-05	0.338083668
cg14448169	WNT16;WNT16	0.00043997	0.338073174
cg12154418	THBS1	0.000148457	0.338071528
cg25848398	C2orf72	7.23E-05	0.338070929
cg05368033	FGFR2;FGFR2;FGFR2;FGFR2;FGFR2;FGFR2	2.58E-05	0.338064971
cg26807939	SOX14	8.60E-06	0.338051524
cg02286506		7.23E-05	0.338017483
cg10576280	PLEKHA1	9.24E-05	0.338011185
cg22946159		1.50E-05	0.3380099
cg19688403		7.23E-05	0.338002073
cg12449685	ROBO4	4.34E-05	0.337996954
cg08504812	C3orf55;C3orf55;C3orf55;C3orf55	0.000290372	0.337993454
cg16489809	TCTN3;TCTN3	4.34E-05	0.337980304
cg02138331	AHCY	1.50E-05	0.337969992
cg13216331	ROBLD3;ROBLD3	0.001639597	0.337953951
cg08522176	ROBO3;ROBO3	3.34E-05	0.337943959
cg21243944		0.000187194	0.337930256
cg14073722	CA10;CA10;CA10;CA10	7.23E-05	0.337921102
cg08662757	SSH3;SSH3	0.009066563	0.337917672
cg14585235		0.000187194	0.337900534

cg07291439	ZIC1	7.23E-05	0.337898998
cg03029734		8.60E-06	0.337896552
cg25826457	HLF;HLF	0.000290372	0.337895126
cg22131691	PDE1C;PDE1C	7.23E-05	0.337883257
cg13832669		0.000570759	0.337858097
cg11956761	LPPR4;LPPR4	1.11E-05	0.337835806
cg12978433	CYP24A1;CYP24A1	0.000117986	0.337830865
cg18688704	PDGFC	6.94E-05	0.337806991
cg04718414	CDC16;CDC16	0.000955793	0.337798938
cg22418829	BTNL9	0.000148457	0.337790118
cg18863333	SOX11	0.002714607	0.337758386
cg11827101		1.11E-05	0.337755544
cg01592801	KCNS2	5.59E-05	0.337754937
cg15608277	KRT3	0.001147425	0.337742725
cg16080781	DBX2	1.96E-05	0.337728519
cg18313899	SLC35F1	8.60E-06	0.337712982
cg06707544	KLK7;KLK7	0.000233422	0.337695592
cg08828819	PON3	0.001147425	0.337692168
cg24743156	CTAGE5;CTAGE5;CTAGE5;CTAGE5	0.002714607	0.337685046
cg25474372		0.000537905	0.337681503
cg26938364	FLJ42875;PRDM16;PRDM16;FLJ42875	0.000654035	0.337667263
cg12052765	SLC18A3;CHAT	1.96E-05	0.337656607
cg03346079	WNT7A	0.000233422	0.337654848
cg08165971	NGEF;NGEF	0.001376172	0.337650287
cg01574364	SLC36A1	4.34E-05	0.337648154
cg11994674		0.000290372	0.337632741
cg17803175	PCSK1	2.58E-05	0.337632569
cg08226111	INSM2	0.000955793	0.337623914
cg19194098	ARMC4	0.006738759	0.337623183
cg08937380	TMEM61	1.11E-05	0.337620745
cg06122230	ZNFX1	0.000791389	0.337578144
cg26266842	LPPR5;LPPR5	4.34E-05	0.337576967
cg23973429	GRHL2;GRHL2	0.000357885	0.337549856
cg21700532	SDR16C5	0.000654035	0.337545557
cg10956096		1.96E-05	0.337544763
cg15956237	ADAMTS9	0.000705485	0.33754168
cg11731671	PRDM16;PRDM16	2.58E-05	0.337529471
cg13738615	ZNF462	0.000357885	0.337502805
cg18358723	LOC645323	0.000654035	0.337481539
cg07268332	AMPD3;AMPD3;AMPD3	0.000148457	0.337474395
cg01070794		9.24E-05	0.337456633
cg07640800	LRRTM1;CTNNA2;CTNNA2	8.60E-06	0.33744855
cg20156450	SLC16A9	0.00043997	0.337440162
cg19427746	CAMTA2;CAMTA2;CAMTA2;CAMTA2	0.000537905	0.337432245
cg04399899	RAPGEF2;RAPGEF2	0.000537905	0.337429899
cg16648062	RIMS2	0.00043997	0.337423733
cg03251287		2.58E-05	0.337422612
cg09048530	FZD10	0.000357885	0.337412541
cg18623216	PLCH1;PLCH1	0.000537905	0.337367339
cg26619908		1.96E-05	0.337363984
cg00033127	SNX7;SNX7;SNX7	0.002040485	0.337350427
cg00674004		0.000357885	0.337322996
cg13985485		0.001147425	0.337316143
cg25287474	ZDHHC21	0.0050758	0.337315359
cg22511808	GFM1	7.23E-05	0.337299537
cg07171496	TDRD5	1.96E-05	0.337299303
cg05398036		0.000955793	0.337298005
cg21762820	FAM78B	8.60E-06	0.337285287
cg22153181	GALNTL6	0.001639597	0.337285136
cg08506931	CCDC140	0.000187194	0.337284049

cg13713293	DMRT1	0.000117986	0.337279132
cg11324953	HTT	2.58E-05	0.337261251
cg20536469	NRXN1;NRXN1;NRXN1	4.34E-05	0.33725893
cg17546366		0.000357885	0.337251551
cg14494620		0.000148457	0.337244014
cg09619146	CPXM2	4.34E-05	0.33721191
cg02471132		9.24E-05	0.337201626
cg05062333	EYA4;EYA4;EYA4	0.001147425	0.337198282
cg14272822	PTCH1;PTCH1;PTCH1;PTCH1	0.000233422	0.337186982
cg01783662	SOX2OT	2.58E-05	0.337184381
cg18984452		1.96E-05	0.337176937
cg05093169	PHOX2A	4.34E-05	0.337175441
cg23697278	GLRA3;GLRA3	7.23E-05	0.337174979
cg15736524		7.23E-05	0.337169576
cg09072120	PRTFDC1	0.000357885	0.337168375
cg15056288		2.58E-05	0.337152469
cg01682111	UNKL	6.94E-05	0.337151158
cg08081323	RTN4RL2	4.34E-05	0.337100815
cg26949694	BDNF;BDNF;BDNF;BDNF;BDNF;BDNF;BDNF	9.24E-05	0.337097038
cg00909926	ESRRG;ESRRG;ESRRG;ESRRG;ESRRG	0.000187194	0.337092148
cg18821485	ARNT2	0.000955793	0.337077611
cg24238409	CPEB3	9.24E-05	0.337072181
cg19908485		3.34E-05	0.33706775
cg07059360	POLR3D	1.11E-05	0.337062785
cg02257750	HSPA12A	0.000117986	0.33705409
cg11750592		1.50E-05	0.337045707
cg23705224	CTXN2;CTXN2	1.96E-05	0.33704275
cg01677601	PARVA	0.000117986	0.337032821
cg11079048	KIAA1598;KIAA1598	0.000357885	0.337031421
cg01286655	DKK3;DKK3;DKK3	1.50E-05	0.336993138
cg18334637	C10orf41;ZNF503	0.000117986	0.336992222
cg27100236	GATA4	7.23E-05	0.33698068
cg18641151	ARHGEF10L	4.34E-05	0.336975277
cg22762091	ADCY8	1.11E-05	0.33695793
cg12508451	LOC145845	2.58E-05	0.336956008
cg23689712	LMX1A	4.34E-05	0.336955465
cg05366118		0.000117986	0.336947825
cg13832670	CREB3L2	7.23E-05	0.336937532
cg01496199		0.000357885	0.33693193
cg05142211	SOX2OT;SOX2	0.000290372	0.336927174
cg16146033	SLC22A8	0.000290372	0.336895371
cg01837362		2.58E-05	0.33688956
cg02850815	SCRIB;SCRIB;MIR937	2.58E-05	0.336874095
cg13912858	SORT1	0.000148457	0.336864353
cg07188523		0.000117986	0.336855508
cg23954268		0.000117986	0.336850305
cg05853159		4.34E-05	0.336841109
cg12213414	LYG2	0.000537905	0.336827864
cg03611868		8.60E-06	0.336822959
cg00705280	MIR375	0.000357885	0.336813455
cg23891273	MYBPC1;MYBPC1;MYBPC1;MYBPC1	0.000791389	0.336808279
cg00625653	WNT7A	7.23E-05	0.336803156
cg27554973		0.000357885	0.336791462
cg04455058		0.000233422	0.336789433
cg02194223	EVX1	0.000148457	0.336786819
cg10313385		9.24E-05	0.336782487
cg12385599	LOC389493	9.24E-05	0.336781698
cg07015911	HOXB4	0.000148457	0.336765494
cg13983063	PPP2R2B;PPP2R2B;PPP2R2B;PPP2R2B;PPP2	3.34E-05	0.336745887
cg24293044	C6orf97	4.34E-05	0.336745852

cg10576051	HCN4	0.000955793	0.33673322
cg14515996		0.000187194	0.336708463
cg09854003		4.34E-05	0.33670533
cg16321280	ALX4	0.000290372	0.336702738
cg15135286	LTBP1;LTBP1;LTBP1;LTBP1;LTBP1	2.58E-05	0.336696952
cg06570224		4.34E-05	0.336688256
cg11800635	DOK1	0.000117986	0.336675286
cg23251248	TBPL2	1.50E-05	0.336646489
cg21644856	FN3K	0.000654035	0.336641767
cg25013011	TMEM132D	5.59E-05	0.336613376
cg13612083	C6orf174	0.000955793	0.336601628
cg25553665	BMPR1B	0.000117986	0.336589285
cg21634365	GALR1	0.000117986	0.336587275
cg20422099	ADAMTS2;ADAMTS2	0.001376172	0.336571344
cg06664254		7.23E-05	0.336568767
cg26816707		1.50E-05	0.336544171
cg12802310	CYP1B1	0.000233422	0.336540615
cg03595755		0.000955793	0.336538599
cg02610222	POU4F2;POU4F2	8.60E-06	0.336524859
cg13928649	PRDM12	1.11E-05	0.33652356
cg24336089	PRDM13	3.34E-05	0.336512531
cg23659250	BRD1;LOC90834	0.000187194	0.336493883
cg11855022	PLSCR4;PLSCR4;PLSCR4;PLSCR4	0.002304201	0.336471548
cg25753024	SSH3	0.009066563	0.336460229
cg09533178	PPP1R12C	3.34E-05	0.336456388
cg17102963	TRIM67	2.58E-05	0.336443198
cg11416811	C10orf107	0.000233422	0.336438908
cg14926196	MDGA1	0.000187194	0.336433593
cg17735593	PCDHB7	4.34E-05	0.336425451
cg06237774	DPY19L2P4	9.24E-05	0.33640914
cg05256605	LOX	0.00043997	0.336398948
cg08012278		1.50E-05	0.336378813
cg05202204		7.23E-05	0.336377758
cg02285812	RND3	8.60E-06	0.336376458
cg12599673	CT62	5.59E-05	0.336368807
cg17540545	FXYD1;FXYD1	0.000654035	0.336366846
cg21074827	MLF1;MLF1;MLF1	0.000117986	0.33635548
cg10594090	HIF3A;HIF3A	0.000537905	0.336349888
cg05327789	SLCO4A1	0.001639597	0.336337486
cg13358873	ANTXR1;ANTXR1;ANTXR1	1.50E-05	0.33632711
cg24475782	TBX15	0.00043997	0.33630026
cg25248750	C8orf47;C8orf47	9.24E-05	0.336275586
cg07303968	HSPA12A	0.001147425	0.336254463
cg26345888	DAB1	0.000233422	0.336253658
cg06899985	IGDCC4	0.00043997	0.33623488
cg17959970		0.000117986	0.336228537
cg13033338	OPCML	3.34E-05	0.336226929
cg07520649	PCDHGA5;PCDHGA4;PCDHGA2;PCDHGB2;PC	0.001147425	0.336217664
cg21762695		0.000187194	0.336215722
cg22283959	NR2F2;NR2F2;NR2F2;NR2F2	1.50E-05	0.336214267
cg23357981	GRP;GRP;GRP	1.96E-05	0.336193596
cg12176249		0.001147425	0.336180814
cg17040303	SIM2;SIM2	0.000654035	0.336173997
cg12356107	FOXA2	0.000654035	0.336171214
cg26468336		8.60E-06	0.336153908
cg00338080	SRRM3	0.000357885	0.336152777
cg15920906	CUX2	0.000537905	0.336141167
cg26680520		0.000537905	0.336131012
cg03860890	EGFR;EGFR;EGFR;EGFR	8.60E-06	0.336110717
cg17446956	SSH1;SSH1	7.23E-05	0.336099539

cg00714725	CYP26C1	0.002714607	0.336095873
cg12432010	IQSEC3	1.50E-05	0.336094476
cg08681432	SCNN1G	0.001948248	0.336060512
cg27099274	C9orf125	1.50E-05	0.336058221
cg12661206	DYNC1I1;DYNC1I1;DYNC1I1	0.001639597	0.336054488
cg11427855		0.000233422	0.33604705
cg20203352		0.000290372	0.336013453
cg19776201	ZNF132;ZNF132	7.23E-05	0.336007511
cg09295081	NTSR2	0.000117986	0.336004301
cg01481544		1.50E-05	0.335990565
cg26094984	HECW2	9.24E-05	0.335986669
cg17510957	SORL1	1.96E-05	0.335986075
cg13491481	CADPS;CADPS;CADPS	0.000654035	0.335960666
cg08470180	C5orf24;C5orf24	0.000187194	0.335948233
cg13316854	HOXD3	1.50E-05	0.335937419
cg16961816	DLX6AS	0.000705485	0.335931332
cg18913890	CTNNA2;CTNNA2	9.24E-05	0.335920868
cg08091192	ZIC4;ZIC4;ZIC4	5.59E-05	0.335901461
cg04541699		1.11E-05	0.335898787
cg17078883		0.000357885	0.335883515
cg09765089		0.003189589	0.335848349
cg09794131	HYDIN;HYDIN	3.34E-05	0.33584603
cg12065579	NEUROG1	4.34E-05	0.335826396
cg08650890	TNXB	0.001376172	0.335774767
cg24697184	TTC22;TTC22	9.24E-05	0.335772933
cg03120091	ADCY8;ADCY8	1.11E-05	0.335763473
cg24491784		0.000148457	0.335760191
cg05906092	SLC37A3;SLC37A3	7.23E-05	0.335749842
cg17832991	OVOL2	3.34E-05	0.335748735
cg00048759	STAG3;GPC2	8.60E-06	0.335729687
cg01560972	RIC3;RIC3	5.59E-05	0.33571712
cg12898019	ST6GALNAC1	0.000955793	0.335682963
cg10548102	MGST1;MGST1;MGST1	4.34E-05	0.335682622
cg08362738	BDNF;BDNF;BDNF;BDNF;BDNF;BDNF;BDNF;B	8.60E-06	0.335672852
cg13686615		0.0050758	0.335665968
cg18519308	ADAMTS12	0.000117986	0.335634522
cg18532215	PLOD2;PLOD2	1.11E-05	0.335622802
cg19623624	LOC619207	5.59E-05	0.335622559
cg23971170	LMX1A	0.001639597	0.335622387
cg08091050		0.000955793	0.335595992
cg26597982	TSNARE1	0.004357816	0.335591559
cg00116699	HDAC4	1.50E-05	0.335579308
cg24664689	XKR6	0.000187194	0.335571335
cg02394955	PPYR1	0.000148457	0.33556815
cg13488811		0.000117986	0.335566552
cg05131623	PEX5L	4.34E-05	0.335565341
cg01292810		0.001948248	0.335553836
cg11228250	PLA2R1;PLA2R1	0.000233422	0.335551573
cg27227742	MATN4;MATN4;RBPJL;MATN4	1.96E-05	0.335513111
cg00841141	SULT6B1	1.96E-05	0.335510055
cg07681935	NBLA00301;HAND2	0.000791389	0.335503234
cg03653541	PDE4D;PDE4D	0.00043997	0.335500898
cg20044860		2.58E-05	0.335488749
cg17307479	BHLHE22	0.000955793	0.335486405
cg19635571		8.60E-06	0.335486058
cg03638905		0.000148457	0.335462932
cg10251896	CRABP1;CRABP1	0.000290372	0.335456635
cg00862116	ABCB1	4.34E-05	0.33544042
cg06252564		8.60E-06	0.335415765
cg22978087	NEFL;NEFL	0.000537905	0.335415247

cg02245612	PTPRS;PTPRS;PTPRS;PTPRS	0.000357885	0.335413392
cg01793593	ROBO1;ROBO1;ROBO1;ROBO1	7.23E-05	0.335411303
cg25711575	PDE11A;PDE11A;PDE11A	0.000233422	0.335406941
cg16584573	FGF8;FGF8;FGF8;FGF8	2.58E-05	0.335386059
cg01834210	HAND1	0.000233422	0.335340516
cg13067553		0.000187194	0.335330173
cg01427458	OPCML	5.59E-05	0.335320027
cg15074033		0.000117986	0.335317056
cg06873880		1.50E-05	0.33531387
cg24532471	FAM181B	9.24E-05	0.335307352
cg25557835	SLC7A2;SLC7A2	0.000537905	0.335300035
cg24376266	FAM3B;FAM3B	0.000537905	0.335297859
cg18115507	NALCN	0.00043997	0.335296012
cg23142799	SHISA2	8.60E-06	0.335292031
cg04557544	SHISA9;SHISA9	0.002266401	0.335290121
cg20597403	SDK1;SDK1	0.00043997	0.335275812
cg10055566	NKAPL	8.60E-06	0.335268937
cg03538436	NOS1;NOS1	2.58E-05	0.335258253
cg18691434	STAG3;GPC2	1.96E-05	0.335251023
cg01715455	VSX1;VSX1	0.000148457	0.335249054
cg02114924	PCDH10;PCDH10	7.23E-05	0.335243435
cg22387369	ALPK3	0.000955793	0.335243163
cg26955835	DKK2;DKK2	0.000187194	0.335241762
cg11500797	DLX5	0.000955793	0.335226362
cg21384203	GSC2	3.34E-05	0.335220666
cg15980539	ESR1;ESR1;ESR1;ESR1;ESR1	0.001147425	0.335218383
cg06991510	BDNF;BDNF;BDNF;BDNF;BDNF;BDNF;BDNF;B	4.34E-05	0.335207971
cg19497501	ZMAT4;ZMAT4	1.96E-05	0.335177173
cg16375290		9.24E-05	0.33517501
cg15680755		7.23E-05	0.335172075
cg25180228	LOC440905	1.50E-05	0.335157753
cg08622198	CHRM3	0.000791389	0.335141048
cg12040846	RUNDC3B;ABCB1;RUNDC3B;RUNDC3B	2.58E-05	0.335123576
cg09053907	BTC	0.000654035	0.335099186
cg15736169		0.000117986	0.335090811
cg23231515	TEAD1	0.000955793	0.335088161
cg02681733	GOLGA7B	0.000290372	0.335081315
cg26554353		0.000537905	0.335065318
cg06346335		0.003734675	0.335055661
cg03018496	NR2F2	0.000357885	0.335053079
cg01627405	MIR1227;PLEKHJ1	0.001061731	0.335052733
cg03445694	LHX8	4.34E-05	0.335030082
cg16462237	ULK2;ULK2;ULK2;ULK2	0.00043997	0.335012644
cg07775371	C16orf73;C16orf73;C16orf73	1.11E-05	0.33500073
cg15980656	SDC2	0.002714607	0.33499255
cg14631053	SSTR4	2.58E-05	0.334991344
cg13999106	NRXN1;NRXN1	0.000233422	0.334988393
cg13381486	FAM123C;FAM123C;FAM123C;FAM123C	7.23E-05	0.334984901
cg08575049	FAT4	8.60E-06	0.334972149
cg20959866	AJAP1;AJAP1	1.50E-05	0.334967984
cg19035107		1.11E-05	0.334945233
cg08215532	DPY19L2P4	9.24E-05	0.334944346
cg11840125	EFS;EFS	0.000122184	0.334937829
cg16717713	CCDC85C	0.00041966	0.334930028
cg14165051	PID1;PID1;PID1;PID1	0.001147425	0.334926023
cg07062043	PHYHIPL;PHYHIPL	2.58E-05	0.334921322
cg01688688	PCDHB18	0.000233422	0.334901836
cg00478246	FAM84A	1.11E-05	0.334887128
cg17634781	SORBS2;SORBS2;SORBS2;SORBS2;SORBS2	2.58E-05	0.334854744
cg03400421		9.24E-05	0.334854588

cg04865691	SOX1;SOX1	0.000654035	0.334828059
cg07390122	RSPO2	5.59E-05	0.334822781
cg22352772		0.000654035	0.334812612
cg00532474	PSTPIP2	0.001147425	0.334796221
cg06298519	GFRA1;GFRA1;GFRA1	0.000955793	0.334778925
cg04390523	EDNRB;EDNRB;EDNRB	0.001147425	0.334755535
cg06148736		0.000117986	0.334729447
cg02757432	GPR26	8.60E-06	0.334722083
cg24355907		1.11E-05	0.334711816
cg15961744	PCDHA6;PCDHA2;PCDHA1;PCDHA7;PCDHA1	1.11E-05	0.33470921
cg15267010	TOX2;TOX2;TOX2	0.005742788	0.334705072
cg01414464		0.000357885	0.334704339
cg06029846	PRMT8	0.000791389	0.334697908
cg08085357	SOX2OT	0.000290372	0.334691528
cg14741939	ZIC1	2.58E-05	0.334682322
cg20058043	FZD10	0.001147425	0.334674462
cg02658730		4.34E-05	0.334670525
cg22890950	PPAPDC1A	5.59E-05	0.33465643
cg23549358	NELL1;NELL1	3.34E-05	0.334650199
cg04098052		9.24E-05	0.334602141
cg16115841	ST6GAL2;ST6GAL2;ST6GAL2	3.21E-05	0.334599858
cg22373221	KIRREL3;KIRREL3;KIRREL3;KIRREL3	1.96E-05	0.334599832
cg25634666	FOLR3;FOLR3	5.59E-05	0.3345966
cg22275864	ADRB3	0.000866489	0.334583505
cg25131922	GPR177;GPR177	1.11E-05	0.334576152
cg14900984	TDH	0.000117986	0.334561762
cg20165381	CD164L2	0.002304201	0.334551029
cg00980319	MMEL1	7.23E-05	0.334548341
cg05873889	SERP2;SERP2	1.11E-05	0.334518135
cg10327400	LRFN5	0.000148457	0.334512313
cg10341556	RGS20;RGS20	9.24E-05	0.33449903
cg25226247	TFAP2B	7.23E-05	0.33448725
cg25900280	TBX4	0.000187194	0.33448055
cg24304249	CACNA1E	8.60E-06	0.334458918
cg17657179	HAS1	0.000233422	0.334446962
cg01047904	PDE3A	0.000290372	0.334439894
cg23296792	FBXO38;FBXO38	0.001948248	0.334434116
cg09493505	VWC2	0.005897668	0.334429369
cg08599259	POU3F1;POU3F1	0.000290372	0.334427118
cg18498593	HOXB13	4.34E-05	0.334424006
cg08280468	SHISA9;SHISA9	7.23E-05	0.334417853
cg02688118		2.58E-05	0.334416305
cg09308336	ACADL	8.60E-06	0.334398708
cg00955307	ILDR2	4.34E-05	0.334396232
cg09295573	KIAA1239	0.000290372	0.334378803
cg08499861		1.96E-05	0.334376332
cg06362543		4.34E-05	0.334365001
cg03169508		5.59E-05	0.334355645
cg15946653	ANKK1	1.96E-05	0.33434646
cg00750428		8.60E-06	0.334344677
cg22526531	KIAA0319L	1.50E-05	0.334342951
cg00945293	FLJ42875;PRDM16;PRDM16;FLJ42875	0.000537905	0.334308748
cg03529568	ZNF207;MIR632;ZNF207;ZNF207	5.59E-05	0.334306622
cg23556533	FAM83H	1.96E-05	0.334303133
cg19047804	TRIM39;TRIM39	4.34E-05	0.334300839
cg07621104	DSCAML1	0.000148457	0.334298523
cg05740045		4.34E-05	0.334283971
cg20920097		4.34E-05	0.334276565
cg17171539		0.001376172	0.33427108
cg09714778		9.24E-05	0.334262248

cg18006637	FAM13A;FAM13A	0.000233422	0.33425649
cg13844341	MAD1L1;MAD1L1;MAD1L1	2.58E-05	0.334249356
cg17830985	SHISA2	4.34E-05	0.334220752
cg09177131	SYT6;SYT6	0.000262362	0.334200306
cg21915313	PCDHGA4;PCDHGA6;PCDHGA1;PCDHGA5;PC	5.59E-05	0.334194341
cg27262735	TLE6;TLE6	4.34E-05	0.334186457
cg20116199	C12orf56;C12orf56	0.000955793	0.334186285
cg19267252		3.34E-05	0.334175082
cg17262810	CCNY;CCNY	0.00043997	0.334163967
cg12213062	SPEG	0.000187194	0.334162588
cg09193347	RASGRF1;RASGRF1	0.000654035	0.334159667
cg00112685	ELAVL3;ELAVL3	0.00043997	0.334159252
cg00528572	MTNR1B	0.000357885	0.334158521
cg14650577	TACR1;TACR1	0.000791389	0.334140654
cg27572072	ADAP1	0.000357885	0.334124524
cg21179457	INSM2	0.000148457	0.334122975
cg13733410	VWDE;VWDE	9.24E-05	0.334114905
cg27520776	ESRRG	0.000570759	0.334108897
cg20335425	S100A8	1.96E-05	0.334099931
cg22125805		0.001376172	0.334094564
cg13216057	DKK3;DKK3;DKK3	0.000117986	0.334093529
cg04853843	IRX6	2.58E-05	0.334085553
cg04349839	SEL1L3	0.000187194	0.334082524
cg14249348	ZAR1	0.00043997	0.334071157
cg19106932		0.006409488	0.334055531
cg03497399	SUSD5	0.00043997	0.334045384
cg04615460		7.23E-05	0.334026573
cg10237044	LHX8	8.60E-06	0.334006105
cg22400166	C14orf23;C14orf23	7.23E-05	0.334003764
cg02326566	PCDHA6;PCDHA2;PCDHA1;PCDHA7;PCDHA1	0.000117986	0.334001106
cg26880055	RIPPLY2;RIPPLY2	8.60E-06	0.333992987
cg10526374	ASCL2	0.002450672	0.333992637
cg10838789	LRRC67	0.000791389	0.333992461
cg13004927	SLC47A1	0.000290372	0.333978779
cg17383727	GDNF;GDNF;GDNF	0.001147425	0.333973625
cg16344234	SEC14L5	0.000955793	0.333966624
cg22409727		0.000290372	0.333942571
cg01287132		0.000290372	0.333942194
cg02085507	TRIP10	0.000148457	0.333928428
cg01953658		0.000122184	0.333927085
cg17789273		1.50E-05	0.333893414
cg16656895	EMX2;EMX2;EMX2OS	1.11E-05	0.333856042
cg20647467	FEZF1;FEZF1	1.11E-05	0.333844807
cg10764541	INSM2	0.000117986	0.333843806
cg05569086	LYNX1;LYNX1;LYNX1;LYNX1;LYNX1;LYNX1	4.34E-05	0.333835708
cg04330683		1.96E-05	0.333828285
cg14765959		0.003734675	0.333821183
cg09644153		8.60E-06	0.333799966
cg24504014		9.24E-05	0.333792534
cg14492800	GDNF	0.002304201	0.3337904
cg13300273	GPR25	0.007873393	0.33377878
cg18825419	ABI1;ABI1;ABI1;ABI1	0.002714607	0.333768018
cg25536373	DPP10;DPP10	0.000357885	0.333762896
cg07139301	SLC2A2	4.34E-05	0.333760739
cg16864658	CCK	0.001147425	0.33376025
cg04731544	LMTK3	0.000117986	0.333756996
cg04894537	KCNQ1;KCNQ1	0.000233422	0.333755419
cg20541178	LYNX1;LYNX1;LYNX1;LYNX1;LYNX1	4.34E-05	0.333728695
cg18734842	FAM176A;FAM176A	7.23E-05	0.333724989
cg25332377	CCDC108;CCDC108	5.59E-05	0.33371365

cg02005873		0.000357885	0.333713591
cg16879574	NHLRC1	2.58E-05	0.333679798
cg26313511	ZNF148	0.000187194	0.333675642
cg20283477		7.23E-05	0.333663518
cg14219290	EGLN3	0.007873393	0.333655527
cg22232500	NCKAP5;NCKAP5	2.58E-05	0.333654621
cg05824050		0.000537905	0.333644911
cg01935149		0.000233422	0.333619959
cg06881398	USP24	0.000290372	0.333616286
cg18111500		0.000233422	0.33361295
cg20806345	NEUROD1	8.60E-06	0.333598502
cg03878133	FIGN	5.59E-05	0.333584631
cg12467264		0.000187194	0.333582735
cg02366534	ME1	4.34E-05	0.333570388
cg20048037	ENPP2;ENPP2;ENPP2	0.000654035	0.333558828
cg17170568	C7orf13;RNF32;RNF32	7.23E-05	0.333554888
cg03663215	ADCY2	2.58E-05	0.333549145
cg18575770	TBX3;TBX3;TBX3;TBX3	0.000357885	0.33354558
cg26495109	LOC283856;GNAO1;GNAO1;GNAO1;GNAO1	0.000187194	0.333537621
cg21113773		8.60E-06	0.333535038
cg16899179	NCOA2	0.000148457	0.333533181
cg05722981		8.60E-06	0.333532224
cg22374057	FAM38B;FAM38B	2.58E-05	0.333506777
cg14598373		0.000357885	0.333493451
cg18537222	PPARG;PPARG;PPARG;PPARG	0.001948248	0.333488571
cg11821817	KIAA1024	9.24E-05	0.333475698
cg07243784	WWC1;WWC1;WWC1	0.000654035	0.333471076
cg04361126	SLC40A1	0.000290372	0.333466322
cg20819482	TNKS	0.000148457	0.33346071
cg23303408	POU4F3;POU4F3	3.34E-05	0.333454482
cg05060949	MNX1;MNX1	0.00043997	0.333450246
cg08287265	NKX2-3;NKX2-3	3.34E-05	0.33345011
cg27353143		0.000187194	0.333422385
cg13474450	SLC45A4	4.34E-05	0.333381807
cg17416793	KCNQ1;KCNQ1	3.34E-05	0.333376926
cg11322464	TPD52L1;TPD52L1;TPD52L1;TPD52L1	7.23E-05	0.333358669
cg03593550	CACNG8;MIR935	0.001948248	0.333336986
cg25537669	NPHS1	0.000290372	0.333330232
cg01129459		1.50E-05	0.333294437
cg26521404	HOXA9	0.00043997	0.333288732
cg14762973	ZSWIM2	9.24E-05	0.333286984
cg19710326	DTNB;DTNB;DTNB;DTNB;DTNB	2.58E-05	0.333272042
cg11833968		4.34E-05	0.333266563
cg14515812	RAP1GAP;RAP1GAP;RAP1GAP	0.000866489	0.333252787
cg24876786	ZFR2;ZFR2;ZFR2	3.34E-05	0.333252422
cg22587602	EGR4;EGR4	0.001376172	0.333242939
cg23154059	WNT6	0.000537905	0.333239194
cg00691123	VGLL4;VGLL4	0.000117986	0.333231635
cg18703601	ROBO1	2.58E-05	0.333217531
cg23637314	ESRRG;ESRRG;ESRRG;ESRRG;ESRRG	7.23E-05	0.333215768
cg08066875		2.58E-05	0.333208396
cg25431220	TOM1L1	7.23E-05	0.333204948
cg04116766		0.000290372	0.333181185
cg23089838	C8orf48;C8orf48	1.11E-05	0.333171027
cg04277560	CADPS2;CADPS2;CADPS2	5.59E-05	0.333163078
cg00903099	HTR5A	0.000537905	0.33315723
cg10298052		0.001376172	0.333143372
cg10539507	NKX6-1	0.000357885	0.333134799
cg10148764	RPL31;RPL31;RPL31	3.34E-05	0.333127739
cg09413950	LOC440040	7.23E-05	0.333098089

cg13588023	RIPK4;RIPK4	5.59E-05	0.333096367
cg26535158	NKIRAS2;NKIRAS2;NKIRAS2;NKIRAS2;NKIRAS	0.000117986	0.333095731
cg15448975	CRMP1	9.24E-05	0.333095667
cg20643029	CLDN14;CLDN14	0.001639597	0.333086535
cg14345128	ONECUT2	0.003734675	0.33305703
cg02434314		0.000117986	0.333043358
cg05585303	RPH3A;RPH3A;RPH3A;RPH3A	3.34E-05	0.333034993
cg05491001		0.000955793	0.333029382
cg07472704	F7;F7	1.11E-05	0.333028439
cg04910921		0.000233422	0.333028397
cg14277923	LHX3;LHX3	7.23E-05	0.333020615
cg08545129		5.59E-05	0.333019237
cg23024047		7.23E-05	0.333005264
cg06630413	HOXC11	0.00043997	0.332977921
cg13413384	RXRA	5.59E-05	0.332966992
cg17006136	SORBS2;SORBS2;SORBS2;SORBS2;SORBS2	0.000654035	0.332955459
cg01084154		7.23E-05	0.332945701
cg05783854	PCDHAC2;PCDHA7;PCDHA12;PCDHA6;PCDH	2.58E-05	0.332941043
cg18065811	HELT	3.34E-05	0.332935366
cg24767956		8.60E-06	0.332929956
cg02694099	CT62	1.11E-05	0.332927046
cg13610307	CACNA1B	0.000290372	0.332897294
cg02162286		0.000117986	0.332895306
cg06620896		5.59E-05	0.332885611
cg03678062	ZC3H12D	9.24E-05	0.332879049
cg06794000	LMO3;LMO3;LMO3	5.59E-05	0.332875251
cg27657429	ARHGAP22;ARHGAP22	0.000537905	0.332871671
cg24138857	TNXB	1.50E-05	0.332871654
cg27252164	ASCL2	0.000290372	0.332864828
cg19800670		8.60E-06	0.332848559
cg16087447	PRICKLE1	0.000654035	0.332837404
cg13222915		8.60E-06	0.332835134
cg23261443	CUX1;CUX1;CUX1	0.000187194	0.332834972
cg14099514	PLCXD3	1.96E-05	0.33282576
cg06341749	PCOLCE2	0.001376172	0.332806233
cg18926108	PCDHGA4;PCDHGA6;PCDHGA1;PCDHGA5;PC	8.60E-06	0.332806146
cg15370180		9.24E-05	0.332799806
cg19355186		4.34E-05	0.332798376
cg22253838	CT62	0.000654035	0.332796444
cg03387135	MIR124-3	0.0050758	0.332790674
cg23976652		4.34E-05	0.332787585
cg07976644	TTC22;TTC22;TTC22;TTC22	0.000526175	0.332784894
cg12346592		0.000290372	0.332767162
cg01458605	ZIC1	0.00043997	0.332764851
cg22750155	STMN2	7.23E-05	0.332762788
cg17787084	LMX1A	1.96E-05	0.332746939
cg08973191	SCRIB;SCRIB	9.24E-05	0.332734085
cg18096722		9.24E-05	0.332728656
cg05473871	ADAMTSL5	0.007873393	0.33272088
cg13917662	CLYBL	0.001639597	0.332707278
cg16571983	EDNRB;EDNRB;EDNRB;EDNRB;EDNRB	0.000791389	0.332703927
cg10514097	FOXF1	0.000148457	0.332691664
cg26067760	HPSE2;HPSE2;HPSE2;HPSE2	0.000290372	0.332688425
cg23866916	SBNO2	1.11E-05	0.332675712
cg20748065	POR	9.24E-05	0.332675545
cg11384848	SPSB4	0.000357885	0.332667153
cg17670496	HCG4	3.34E-05	0.332663664
cg11908751	SYT12	0.000791389	0.332647922
cg09105881	ABCB1	1.11E-05	0.332644012
cg07902789	PRDM16;PRDM16	4.34E-05	0.33263153

cg12369353		0.000117986	0.332626157
cg09271157	PPAPDC1A	5.59E-05	0.332624681
cg03052004		4.34E-05	0.332610511
cg04466549		1.11E-05	0.332587501
cg11368617		1.96E-05	0.332582596
cg20464246	ESRRG;ESRRG;ESRRG;ESRRG;ESRRG	0.000148457	0.33258097
cg08080396		0.000357885	0.332576962
cg09326345	MARVELD3;MARVELD3	1.96E-05	0.332565757
cg08832603	DLX5	0.000791389	0.332563343
cg02747950	RAB8B	0.001376172	0.332563241
cg00730653	WNT7A	0.00043997	0.332548688
cg15105359	TNS3	0.003189589	0.332532645
cg14135814	SRRM4	0.000537905	0.332526852
cg07436152		1.96E-05	0.332520044
cg12951296		1.96E-05	0.332499959
cg24841257	SNX7;SNX7	0.000233422	0.332491643
cg04753583		0.000117986	0.332473591
cg02760031	TCF15	0.000148457	0.332471182
cg26599006	GSC2	0.000290372	0.332455606
cg08164191		0.000791389	0.332427245
cg08248516	HOXA7	0.0050758	0.332402497
cg07760204		8.60E-06	0.332399176
cg10113101	SCRN1;SCRN1;SCRN1;SCRN1	3.34E-05	0.332398193
cg11016563	TRPC6;TRPC6	1.11E-05	0.332389161
cg00916536	CD8A;CD8A;CD8A;CD8A	7.23E-05	0.332388238
cg01064918	SLC24A4;SLC24A4;SLC24A4;SLC24A4	0.000537905	0.332376826
cg21838979	C2orf40	7.23E-05	0.332366734
cg22287014		4.34E-05	0.332354822
cg17404534	DAGLB;DAGLB	4.34E-05	0.332351933
cg09195098	GCNT2	0.006823935	0.332349702
cg18533201	GDF6	0.000357885	0.332346034
cg27139419	IGF1R	0.00043997	0.332328727
cg19063972	SOX21	4.34E-05	0.33232474
cg07835051	RAX	1.96E-05	0.332309063
cg20129213	RIMS2	0.000526175	0.332290789
cg18193094	GRIK2;GRIK2;GRIK2;GRIK2;GRIK2;GRIK2	8.60E-06	0.332290714
cg25912580		0.000117986	0.332288263
cg24159514	ZNF833	0.00043997	0.33227375
cg16206838	SLC25A26	2.58E-05	0.332268071
cg21912653	NDFIP2;NDFIP2	3.34E-05	0.332267176
cg26514552	C4BPA	5.59E-05	0.332259716
cg19768329	ZNF697;ZNF697	0.000654035	0.332255996
cg11362010	SOX2OT	2.58E-05	0.332249039
cg00505045	POU4F2	5.59E-05	0.332240127
cg21051972		4.34E-05	0.332237171
cg01798589	STAC	0.000187194	0.332236655
cg08863777	FUT4	0.000187194	0.332213155
cg10144400		1.50E-05	0.332198836
cg23562261	EFCAB1;EFCAB1;EFCAB1;EFCAB1;EFCAB1	4.34E-05	0.332198227
cg19899882	FOXQ1	8.60E-06	0.332196474
cg00719651		8.60E-06	0.332192532
cg04768935		1.96E-05	0.332157322
cg01371799		2.58E-05	0.332154244
cg24986840		0.000148457	0.332136797
cg15046877	OPCML	0.00043997	0.332120321
cg12544974	SNCAIP	0.00043997	0.332111578
cg03781931	ALX1	8.60E-06	0.332103312
cg14346243	SNCA;SNCA;SNCA;SNCA	4.34E-05	0.332092275
cg18331412	TGFBI	2.58E-05	0.332078495
cg09150076	TRIM67	5.59E-05	0.33207427

cg04095724	SLITRK1;SLITRK1	3.21E-05	0.332068779
cg25326048		1.96E-05	0.332066643
cg00225858	ADAD2;ADAD2	9.24E-05	0.332052128
cg14420245	ACVR2A	0.001147425	0.332043791
cg20098659	CLEC9A;CLEC9A	3.21E-05	0.332042508
cg17394623	CARS2	0.000791389	0.332039899
cg26164577	SNRPF	0.002714607	0.332036628
cg25627263	OSBPL5;OSBPL5;OSBPL5	0.000290372	0.332008326
cg25188032	SLITRK1	1.11E-05	0.332003275
cg12745764	ZNF804B	0.000396647	0.331980199
cg18468354		9.24E-05	0.331972203
cg17449954	HEYL	0.004357816	0.331969668
cg08640046		4.34E-05	0.331945755
cg12697442	YAP1;YAP1	0.001639597	0.331922296
cg01072639	CHGB	5.59E-05	0.331917417
cg00971695		1.96E-05	0.331913324
cg00450824	STK33	0.00043997	0.331907054
cg00777627	PDE10A;PDE10A	0.00043997	0.331905431
cg03291835	COL4A1	8.60E-06	0.331902713
cg02825171	DACT2	1.96E-05	0.331890733
cg09572169	PLEKHA7	1.11E-05	0.331882097
cg07095530	ZNHIT1;PLOD3	5.59E-05	0.331881528
cg24139737	C10orf72;C10orf72	5.59E-05	0.331870087
cg27203560		0.000148457	0.33186547
cg15379858	CSGALNACT1;CSGALNACT1	1.50E-05	0.331856659
cg12422688		0.000117986	0.331846845
cg01821022	PPHLN1;PPHLN1;PPHLN1;PPHLN1;PPHLN1;P	0.007873393	0.331846685
cg26982279	ZNF470;ZNF470	0.000148457	0.331842101
cg13870990	DSC2;DSC2	0.001639597	0.331838125
cg00883212	RAPGEF4;RAPGEF4	0.000117986	0.331835759
cg01287000		8.60E-06	0.331812319
cg02403883	PCDHB6	2.58E-05	0.331805439
cg18474742	SAMD5	9.24E-05	0.331799249
cg13079123		0.004357816	0.331794002
cg04169021		0.000117986	0.331790807
cg13884903	C20orf56	0.001147425	0.331789657
cg00630724	PITX3	9.24E-05	0.331788564
cg11282657	TSPAN10	3.34E-05	0.331785476
cg00950846	CDH8	9.24E-05	0.331785165
cg16301890	EMX2;EMX2OS;EMX2	4.34E-05	0.331782152
cg09988118	TBX18	0.000117986	0.331767809
cg01541645		2.58E-05	0.331759169
cg00876757		0.000117986	0.331738414
cg14472390	PCDHGA4;PCDHGA2;PCDHGB2;PCDHGA1;PC	5.59E-05	0.331735817
cg16997364		5.11E-05	0.331735163
cg01299579	NOL10	0.001948248	0.331714142
cg07434402	DLG2;DLG2	0.002714607	0.331707564
cg26547816	ARHGAP26;ARHGAP26	0.000148457	0.331704673
cg03789420	CADM3;CADM3	5.59E-05	0.331700945
cg11235498	SPSB4	0.000654035	0.331700437
cg09803407	KIRREL3;KIRREL3	0.001376172	0.331698448
cg09076788	ITPRIPL2;ITPRIPL2	0.001639597	0.331684561
cg01407406	ADAMTS18	0.000117986	0.331683602
cg08899523	FGFR2;FGFR2;FGFR2;FGFR2;FGFR2;FGFR2;F	8.60E-06	0.331680839
cg06493386	TRPA1;TRPA1	4.34E-05	0.331667545
cg25181710	ASXL2	0.003189589	0.331646237
cg11965370	NTM;NTM;NTM;NTM;NTM;NTM;NTM	7.23E-05	0.331639276
cg04603391		5.59E-05	0.331623932
cg01663603	KCNB1	0.000187194	0.331618332
cg20979799	RFX6	1.96E-05	0.331615807

cg20775959	ARNT2	7.23E-05	0.331591038
cg18099189		7.23E-05	0.331577675
cg24890104		0.000148457	0.331562339
cg18597883	C10orf41;ZNF503;C10orf41	9.24E-05	0.331557103
cg27641532		4.34E-05	0.331547427
cg06110728	NELL2;NELL2;NELL2;NELL2;NELL2	0.000537905	0.331543354
cg15703632	PAX3;PAX3;PAX3;CCDC140;PAX3;PAX3;PAX3	5.59E-05	0.331543007
cg18443629	GALR1	4.34E-05	0.331522713
cg03109101	EMX2;EMX2;EMX2OS	0.000187194	0.331506632
cg01658421	EVX2	0.000148457	0.331502037
cg13728797		2.58E-05	0.331495889
cg22287067	NOL4	3.34E-05	0.331490088
cg05241355	OTX2	0.000290372	0.331487299
cg06234167	PHF21B;PHF21B	3.34E-05	0.331483988
cg20241876	NOTCH4	0.000117986	0.331483891
cg10126409	PRRX1;PRRX1	9.24E-05	0.331480165
cg08354889	DEPDC7;DEPDC7	0.000654035	0.331471524
cg22356339	ASCL1	0.000233422	0.331465644
cg25101396	ABHD1	9.24E-05	0.331464664
cg25399352	TBX5;TBX5;TBX5;TBX5	0.001376172	0.331453951
cg12062099	WDR63	0.000791389	0.331438244
cg01288724	ARHGAP26;ARHGAP26	0.002714607	0.331419417
cg10323552	PCDH8;PCDH8	0.000654035	0.331419033
cg05596265	CNTN4	1.11E-05	0.331411666
cg12146864	LRP1	0.001948248	0.331404738
cg26248284	LIMCH1;LIMCH1;LIMCH1;LIMCH1;LIMCH1;LIM	0.00043997	0.331402403
cg16203125	ONECUT1	0.000117986	0.331401419
cg00333226	VWC2	0.001376172	0.331390743
cg25063151	MXRA8	0.004357816	0.331387721
cg05923226	CCDC105	8.60E-06	0.331383538
cg25724895	C20orf197	0.000233422	0.331382434
cg09678615	TOX3;TOX3	3.34E-05	0.331377363
cg06616081	TSPAN3;TSPAN3;TSPAN3	1.50E-05	0.331371982
cg07151747	UPP2;UPP2	0.000148457	0.331369213
cg10715265	DAB1	0.000357885	0.331361402
cg25443851		0.002304201	0.331356014
cg10582968	CCDC33	0.000955793	0.331355569
cg23677243	MEIS2;MEIS2;MEIS2;MEIS2;MEIS2;MEIS2;MEI	4.34E-05	0.331353764
cg12215340	ADRA1D;ADRA1D	0.000187194	0.331350232
cg25456849	ANKRD34C;ANKRD34C	0.000290372	0.331344858
cg20585038		4.34E-05	0.331344382
cg05604874		0.000357885	0.331332281
cg01817029	LOC283392;TRHDE;LOC283392	5.59E-05	0.331325168
cg16772035	ABCB1	1.11E-05	0.331306812
cg05876918	PIP4K2C;PIP4K2C;PIP4K2C;PIP4K2C	5.59E-05	0.331271435
cg12212311		0.000654035	0.331267084
cg12648520		0.000117986	0.3312624
cg04373435	CTNNA2;CTNNA2	0.000233422	0.331231847
cg02526986	RFX6	1.11E-05	0.331209331
cg00745231	PRIMA1	5.59E-05	0.331198134
cg12009759	HHIPL1;HHIPL1	0.000290372	0.331191803
cg06188670	DKK3;DKK3;DKK3	0.002304201	0.331164658
cg25626427		0.000187194	0.331149839
cg25961205	GRTP1	7.23E-05	0.331146861
cg05758246	TFAP2A;TFAP2A;TFAP2A	1.50E-05	0.331136863
cg17418743		0.000955793	0.33113012
cg25674286	ADORA3;ADORA3;ADORA3	7.23E-05	0.331119474
cg00616687	SIM2;SIM2	0.003734675	0.331115423
cg05236879		7.23E-05	0.331101701
cg03817939	FAM176A;FAM176A	0.0050758	0.331100276

cg14609025		0.000187194	0.331078754
cg02509086		0.000148457	0.331065161
cg24155384		3.34E-05	0.331064933
cg11092616	RIMS1	0.000357885	0.33106478
cg04891053	PVRL4	0.000117986	0.331056411
cg02400433	PRDM13	1.96E-05	0.331055595
cg25106913		2.58E-05	0.331042364
cg05433490		0.000187194	0.331032946
cg19694200	TFAP2C	8.60E-06	0.331032027
cg18090004		1.50E-05	0.33100351
cg08616516	GRM5	7.23E-05	0.33098524
cg05842855		1.96E-05	0.330979503
cg02102075	RAB11FIP3	0.000187194	0.330921718
cg09844907	MPV17L;MPV17L;MPV17L;MPV17L	0.000955793	0.330901511
cg15724534	C2orf67	4.34E-05	0.330899727
cg00683156	OGDHL;OGDHL;OGDHL	3.34E-05	0.330894196
cg18562243	SOX2OT	0.000148457	0.330892085
cg14780466	GDF7	1.50E-05	0.330875861
cg13481359	EGR4	2.58E-05	0.330861949
cg00500705		2.58E-05	0.330859039
cg17874478	SPATA6	0.001147425	0.330836744
cg19021383	MAPK8IP1	7.23E-05	0.330832956
cg10245879	NPTX2	4.34E-05	0.330828022
cg15448829	SAMD12;SAMD12	3.34E-05	0.330812656
cg22539420		0.000233422	0.330804949
cg07362168	ISL1	0.000148457	0.330788048
cg15063116	CIDEA;CIDEA;CIDEA	0.000148457	0.330778943
cg05579030	PCDHB5	0.000187194	0.330752139
cg19877683	B3GNTL1	9.24E-05	0.330738342
cg03978579	C10orf53;C10orf53	1.11E-05	0.330731471
cg19535607	CHAT;CHAT;CHAT;CHAT;CHAT;CHAT;CHAT	1.11E-05	0.330729261
cg20596493		0.000791389	0.330718407
cg02444987	RND3	4.34E-05	0.330716119
cg20710709	PAX9	0.000148457	0.33071377
cg23049307	DHCR24	3.34E-05	0.330706863
cg06328855	RBM20	0.009066563	0.330706009
cg13893555	FBXO10	8.60E-06	0.330701725
cg10508760		4.34E-05	0.33070079
cg06974592		0.000233422	0.330667796
cg20229496		0.000148457	0.330666419
cg05878558	PVR;PVR;PVR;PVR;PVR;PVR;PVR;PVR	0.000148457	0.3306663
cg04264638	CLOCK	0.002304201	0.330663454
cg12919006	CNTNAP5	9.24E-05	0.330654141
cg26830291		0.000148457	0.330645857
cg14057383	AFF3;AFF3	7.23E-05	0.330634799
cg02169487		5.59E-05	0.330626402
cg19964641	SEC14L4;SEC14L4	1.11E-05	0.330618453
cg26419728		3.34E-05	0.330618221
cg23444468	ERC2	2.58E-05	0.330608499
cg18406033	PAX3;PAX3;PAX3;PAX3;PAX3;PAX3;PAX	0.001147425	0.3306033
cg27110994	PDGFC;PDGFC	9.24E-05	0.330597521
cg19519310	FOXF2	0.000233422	0.330585973
cg06197377	PCSK9	8.60E-06	0.330541651
cg16592832	C22orf45;C22orf45;UPB1	0.000117986	0.330536898
cg09248345	LMX1A	0.000526175	0.33053446
cg26436330	COL11A1;COL11A1;COL11A1;COL11A1	0.000117986	0.330530814
cg01009697	NTRK2;NTRK2;NTRK2;NTRK2;NTRK2;NTRK2;[illegible]	0.002714607	0.330528466
cg06508320	PEX5L	7.23E-05	0.330527912
cg13059719	FAM163A	4.34E-05	0.330527363
cg23907053	RAB3IP;RAB3IP;RAB3IP;RAB3IP;RAB3IP	0.005897668	0.33052689

cg02018902	ANKRD34C	5.59E-05	0.330491169
cg04438558	PCDHGA2;PCDHGA3;PCDHGA3;PCDHGA1	0.000357885	0.330489902
cg16254962	EGFLAM	0.000117986	0.330486792
cg09561830	UBAC2;MIR623;UBAC2;UBAC2	0.002714607	0.330484782
cg09553242		4.34E-05	0.330479529
cg18342279	ZAR1	0.000654035	0.330461542
cg13653144	LMX1B	7.23E-05	0.330460109
cg22485350	DAB1	4.34E-05	0.330451676
cg24924958	NMBR	0.00043997	0.3304392
cg02592727	RDH13;RDH13;RDH13;RDH13	3.34E-05	0.330434547
cg17460441		0.000570759	0.330415891
cg15976763	AQP4;AQP4	1.50E-05	0.330412373
cg03764518		0.000187194	0.33040617
cg20145426		0.003189589	0.330391798
cg24900370	SAMD5	0.000117986	0.330368652
cg25705486	LOC100192426;PTPRM;PTPRM	0.000357885	0.330362352
cg22082397	DKK3;DKK3;DKK3;DKK3	0.000233422	0.330345215
cg14070647	RSPO2	5.59E-05	0.330330344
cg24286765		1.50E-05	0.330320096
cg12991125	PLA2R1;PLA2R1	0.000654035	0.330319148
cg21253130	FRY	4.34E-05	0.330303062
cg19210770	ACCN4;ACCN4	9.24E-05	0.330296249
cg10575075	ZNF438;ZNF438;ZNF438;ZNF438;ZNF438;ZNF4	0.000654035	0.33028685
cg18081498		4.34E-05	0.330282626
cg26239984		0.000233422	0.330282518
cg23129170	CCDC108;CCDC108	3.34E-05	0.330259443
cg25511332		1.11E-05	0.33024991
cg08836481	LARP4B	9.24E-05	0.330246063
cg22478591	GPM6A;GPM6A;GPM6A;GPM6A	0.000290372	0.330242678
cg11284842		0.001147425	0.330237547
cg25984400		0.00041966	0.330235683
cg01850269	SIM1	4.34E-05	0.330231933
cg19401340	PPM1E	1.96E-05	0.330230201
cg00072720	CLDN7	1.96E-05	0.330226244
cg18247042	SIX1	2.58E-05	0.330221211
cg24079361	SERP2;SERP2	0.000233422	0.330218188
cg16711612	NRXN3;NRXN3;NRXN3	9.24E-05	0.330214283
cg04427852	NMU	4.34E-05	0.330208294
cg18031850	SLC7A10	0.000290372	0.330200237
cg01591938	APBA2;APBA2	0.000187194	0.330200086
cg02074956	MGRN1;MGRN1;MGRN1;MGRN1	1.50E-05	0.330193769
cg16394551	PRDM16;PRDM16	1.50E-05	0.330185135
cg22958047		4.34E-05	0.330161387
cg24235882	CHIC2	0.000290372	0.330133948
cg16766249	NRXN3;NRXN3;NRXN3	0.000233422	0.330125475
cg25763393	ZNF578;ZNF578	0.002714607	0.330123872
cg14533390	MAGI1;MAGI1	0.001948248	0.33011875
cg03618113		4.34E-05	0.330114154
cg25993718	CBLN4	2.58E-05	0.33008049
cg05578989	CA8	0.001147425	0.330075425
cg03816707	KCNS1	0.000654035	0.330052809
cg21995068	LOC100134868	4.34E-05	0.330042133
cg01472538	SETD6;SETD6	0.002266401	0.3300393
cg07425885		0.001639597	0.330037915
cg04751631		0.000117986	0.329997924
cg05240479	MLPH;MLPH;MLPH;MLPH	0.000117986	0.329990777
cg25433267	C10orf41;ZNF503	0.000123078	0.329951698
cg04113094		0.00043997	0.329944613
cg13518327	THRB;THRB;THRB	0.000654035	0.329941527
cg19416570	ZNF274;ZNF274;ZNF274	4.34E-05	0.329936401

cg03044239	MRAS;MRAS;MRAS	9.24E-05	0.32989254
cg02057211	OSBPL10	0.000357885	0.329886039
cg09169617	LOC254559	3.34E-05	0.329880797
cg25382751	TSPAN16;TSPAN16	3.34E-05	0.329874301
cg03048029	SYNJ1;SYNJ1;SYNJ1;SYNJ1	0.000233422	0.329841447
cg04507775	NID1	0.000654035	0.329838204
cg19003884	PRDM13	8.60E-06	0.329820969
cg12425057	MRPS25	0.000233422	0.329817934
cg15393937	INS-IGF2;IGF2AS;IGF2;IGF2;IGF2AS;IGF2	0.000357885	0.329799511
cg26814100	MAP7	9.24E-05	0.329798909
cg25074493		8.60E-06	0.329798255
cg01815567		0.004357816	0.329797611
cg15683970	FAM165B	1.96E-05	0.329792233
cg14830601	VAX1;VAX1	4.34E-05	0.329789495
cg16964389		0.0050758	0.329788056
cg14956197		4.34E-05	0.329781035
cg21348210		7.23E-05	0.329778805
cg04044720		7.23E-05	0.329746438
cg17525406	AJAP1;AJAP1	0.000357885	0.329746307
cg13423075	LHCGR	0.000233422	0.329719281
cg18428265	NPAS2	0.000117986	0.32970915
cg18025136	C1orf159	0.000357885	0.329707082
cg13744194	OCLN	0.000148457	0.329674783
cg27511599		0.000233422	0.329673459
cg23326228	CLVS1	0.000205664	0.329668882
cg26976732	HBM	0.000290372	0.329656805
cg18461021	GALR1	0.000148457	0.329653871
cg07394719	C10orf90	0.000357885	0.329653417
cg14101494		0.000955793	0.329628819
cg18538340	TMEM108;TMEM108	0.000233422	0.329624906
cg23093589	SP8;SP8	0.000187194	0.329599876
cg23092040		8.60E-06	0.329593365
cg09074113	GDF7	1.11E-05	0.329592463
cg21484213	LRP1B;LRP1B	0.000187194	0.329583708
cg03912954	CACNA1B	1.11E-05	0.329575843
cg13633497	MADCAM1;MADCAM1	0.000654035	0.329573513
cg24916013	WTIP	9.24E-05	0.329567224
cg13482485	SNX7;SNX7	7.23E-05	0.329567072
cg14145801	ZSWIM2	0.00043997	0.329559846
cg07572341	FOXA2;FOXA2	1.11E-05	0.329559542
cg12157673	ZNF578	8.60E-06	0.329554368
cg26588061	UPF2;UPF2	0.001147425	0.32955423
cg09446136	KITLG;KITLG	8.60E-06	0.329551733
cg12221475	FOXF2	3.34E-05	0.3295506
cg01454215	UCN	0.000955793	0.329550219
cg26112871	FBXW11;FBXW11;FBXW11	9.24E-05	0.329537251
cg07943461		1.50E-05	0.329529359
cg27440715	LMX1A	3.34E-05	0.329526885
cg22747076	HOXC4;HOXC4	0.001376172	0.329523661
cg21700663		1.96E-05	0.329518605
cg18416950	HOXB2	9.24E-05	0.32950045
cg00009292		7.23E-05	0.329493076
cg08879910	HLA-J;NCRNA00171	0.000654035	0.329490405
cg04373334	FAT4	0.000117986	0.329488949
cg16419441	DPYS	0.0050758	0.32948232
cg16061012	ARL4A;ARL4A;ARL4A	0.005897668	0.329479475
cg07247419	NKX2-4	9.24E-05	0.329475291
cg06382526		0.000148457	0.329474832
cg07552803	NEFM;NEFM	0.000290372	0.329469563
cg22604636	ATOH1	1.50E-05	0.329468907

cg17464383	PRDM6	0.001639597	0.329468415
cg07503059	EPB41L4A;FLJ11235	0.001639597	0.329449499
cg02710296	C1orf14	0.004357816	0.329449053
cg05347845	ACTA1	0.000955793	0.329443175
cg16241421	CUX2	0.000357885	0.329439947
cg09508016		3.34E-05	0.329438009
cg00838898		0.000233422	0.329434683
cg03549146	MIR140;WWP2;WWP2	0.000117986	0.329429194
cg19181162		1.11E-05	0.329412895
cg17147045	PREX2;PREX2	0.000537905	0.329412512
cg18833573		0.000117986	0.329399647
cg09096555	GRIN2C	8.60E-06	0.329399247
cg13305823	MAS1L	0.000357885	0.329384098
cg12007399	ZFR2;ZFR2;ZFR2	1.50E-05	0.329368318
cg10445453	ADAMTS14;ADAMTS14	0.002714607	0.329366337
cg26561681	EIF4G3	0.0050758	0.329335226
cg03600259		1.96E-05	0.32933016
cg01877450	BRI3;BRI3	0.000187194	0.329319848
cg17541528	COL4A1;COL4A2;COL4A2	0.007873393	0.3293071
cg22538959		8.60E-06	0.329297578
cg17605235	ANKRD18A;C9orf122	2.58E-05	0.329273645
cg13933734		0.000187194	0.329272797
cg17650274	VSX1;VSX1	8.60E-06	0.32926828
cg04557057		7.23E-05	0.329260991
cg03149699	PRICKLE1	0.001376172	0.329209648
cg05328197	MAPK4	0.000233422	0.329201404
cg23757446	ONECUT2	9.24E-05	0.329189385
cg23381749		3.34E-05	0.329169421
cg25556204	UGGT2	0.000233422	0.329160163
cg03340398	SLC7A2	7.23E-05	0.329154459
cg24071726	LOC440925;SP5	0.000290372	0.329151093
cg08912317	SCIN	0.002714607	0.329137175
cg01087254		0.005897668	0.329114927
cg08938906	RFX4	1.11E-05	0.329105103
cg07167168	PTPRT;PTPRT	0.00043997	0.329104587
cg05143887	ACSS3	0.000187194	0.329096906
cg17517865	KCND3;KCND3	0.001948248	0.329090459
cg26316946	GRIK2;GRIK2;GRIK2;GRIK2;GRIK2;GRIK2	1.50E-05	0.329089961
cg18900649	PRDM13	2.58E-05	0.329079406
cg11199869	FMO1	0.000791389	0.329078642
cg13762060	DMRT1	9.24E-05	0.329054369
cg22977892		0.000791389	0.329048008
cg05437823	TFAP2B	0.000187194	0.329039974
cg00501765	C1orf105	3.34E-05	0.329039111
cg21068911	ELMOD1;LOC643923;ELMOD1	0.000791389	0.329032628
cg21092808	HOXD3	5.59E-05	0.329030319
cg26095658	FIGLA	2.80E-05	0.329024198
cg23097006	VSX1;VSX1	9.24E-05	0.329004015
cg01832892	ANO3	9.24E-05	0.328998831
cg26248173		0.002714607	0.328998573
cg03629151	SDR42E1	1.11E-05	0.328993822
cg21570988	PRRT1	4.34E-05	0.328987854
cg08160091		8.60E-06	0.328986145
cg26620655	C7orf50;C7orf50;C7orf50	4.34E-05	0.328985462
cg15219811	ACTR1A	0.001376172	0.328982008
cg11006995	EBF2	4.34E-05	0.328978199
cg04415176	HOXD13	0.000955793	0.328972968
cg00488091		1.96E-05	0.328962015
cg01853561	SIM2	0.000148457	0.328959694
cg20080624	DLX5	0.000117986	0.328942991

cg01268901	WNT5B;WNT5B	4.34E-05	0.32893924
cg02699332	ARHGEF4;ARHGEF4	9.24E-05	0.328928572
cg10280347	PPAPDC1A	8.60E-06	0.328907235
cg26220673		0.000537905	0.328885908
cg20771178	STK33	9.24E-05	0.328871492
cg15997429	DACT2	7.23E-05	0.328870403
cg27213509	EVX2	8.60E-06	0.32886131
cg22396959	PPAP2B;PPAP2B	0.000233422	0.328855512
cg26228266	DLGAP3	0.002266401	0.328854092
cg11325267		8.60E-06	0.328834123
cg13057857		4.34E-05	0.328833412
cg05229355	ADCY8;ADCY8	1.11E-05	0.32882596
cg19134568	POU4F2	1.50E-05	0.328803914
cg08478447	CACNA1D;CACNA1D;CACNA1D	0.000357885	0.328784341
cg19698403		1.11E-05	0.328775137
cg21653826	EFHA2	2.58E-05	0.328774146
cg02431597	CACNA1E	0.000117986	0.328772687
cg15609237	TTN;TTN;TTN;MIR548N;TTN	2.58E-05	0.328748682
cg07204353	OSBP2	0.000955793	0.328727265
cg15713630	LMX1A	1.50E-05	0.328721876
cg22614142	KIFC2	0.001639597	0.328718693
cg20464368	HEATR1	4.34E-05	0.328709677
cg03546806	RBM19;RBM19;RBM19	8.60E-06	0.328700227
cg18049167	PPT2;PPT2	0.007873393	0.328691266
cg05855039	FLRT2;FLRT2	5.59E-05	0.328662247
cg17538572	CYP26A1;CYP26A1	0.000357885	0.328645915
cg10287137	P2RY2;P2RY2;P2RY2	3.34E-05	0.328624144
cg14556683	EPHX3;EPHX3	0.000290372	0.328619409
cg24457126	CYP39A1;SLC25A27	0.000187194	0.328604427
cg17627617	PAQR9	0.001376172	0.328600539
cg01468309	PARVA	1.96E-05	0.328597195
cg03769383	PDZRN4	0.000537905	0.328596446
cg24497186		0.000187194	0.328594654
cg14775114	TFPI2;TFPI2	0.000290372	0.32857963
cg07040303		8.60E-06	0.328567872
cg07057636	CYP1B1	0.000290372	0.328562319
cg23418645	GPHN;GPHN	0.000148457	0.328555077
cg22827063		2.58E-05	0.328535778
cg25717239	HECW1	0.001147425	0.328518937
cg07850418	ZIC4;ZIC4;ZIC4;ZIC4;ZIC4	9.24E-05	0.328516822
cg26686361		9.24E-05	0.328513597
cg20510724	ACAN;ACAN;ACAN;ACAN	7.23E-05	0.328510004
cg16519192		5.59E-05	0.328507797
cg10146203	GABRA5;GABRA5	0.000187194	0.328493597
cg03693911	PPP2R2B;PPP2R2B;PPP2R2B;PPP2R2B;PPP2	5.59E-05	0.328488951
cg24717325		2.58E-05	0.328484899
cg26384430	NPY2R	1.50E-05	0.328475963
cg24581326	TFAP2C	2.58E-05	0.328469893
cg00660105		1.11E-05	0.328456821
cg14844194		0.000187194	0.328453019
cg04180462	ACAT1	5.59E-05	0.328445874
cg11881313	LHFPL3	1.96E-05	0.328443758
cg24451800	PTPRM;PTPRM	0.000357885	0.32844086
cg18032891	C8orf48;C8orf48	7.23E-05	0.328438827
cg08614481	HTR1B	8.60E-06	0.328432673
cg20871586		0.000122184	0.328408881
cg12406559	ZNF532;ZNF532	3.34E-05	0.328402622
cg24435401	NPAS4	7.23E-05	0.328397721
cg18583378	ATG9B	0.000290372	0.328393662
cg11002686		2.58E-05	0.32838777

cg15579817	VAX2	0.002714607	0.328380032
cg25161129	WIPI1	0.004357816	0.328371861
cg07769790	CPEB1	1.50E-05	0.328354345
cg07739113		0.00043997	0.328353951
cg25313204	SLC22A3	9.24E-05	0.328347344
cg07690768	BICD1;BICD1	1.96E-05	0.32834381
cg22993195	AMIGO3;RNF123	1.96E-05	0.328335975
cg05694021		0.0050758	0.328331133
cg19416417	SGPP2	3.34E-05	0.328321945
cg08152546		0.000233422	0.328298255
cg09188868	PIP4K2C;PIP4K2C;PIP4K2C;PIP4K2C	1.50E-05	0.328289971
cg04159901		0.001147425	0.328279614
cg02631462	LYPD5	5.59E-05	0.328273238
cg22717109	REEP1;REEP1;REEP1;REEP1	4.34E-05	0.328271017
cg20272619	DAB1	1.96E-05	0.328236762
cg05834899	SART3;ISCU;ISCU	3.34E-05	0.328236048
cg18788741	FAM150A	0.002304201	0.328225362
cg16461251	TACR3;TACR3	3.34E-05	0.328215285
cg07356745	FAM190B	0.001147425	0.328208388
cg19730972		0.000357885	0.32820338
cg22058990		4.34E-05	0.328183732
cg01329687	SNN	0.000357885	0.328170006
cg23739994	GPR83	7.23E-05	0.328161238
cg19267325	NELL2;NELL2;NELL2;NELL2;NELL2;NELL2;NE	0.009066563	0.328158804
cg15948567	AKR7L;AKR7L	0.000148457	0.328148901
cg22539069	ONECUT1	4.34E-05	0.328147845
cg24634422	SLC1A2	0.000187194	0.328140061
cg04936009		0.000117986	0.328132339
cg10543574		0.000117986	0.328128406
cg22179082	GPR12	0.000290372	0.328118665
cg23978504	SOX2OT	0.000148457	0.328112246
cg17172851	PAX7;PAX7;PAX7	0.006823935	0.328097053
cg04899492	NRSN1	5.59E-05	0.328077951
cg17093795	GRP;GRP;GRP;GRP;GRP;GRP	9.24E-05	0.328077426
cg01101663	PCDHB16	8.60E-06	0.32807319
cg04418492	CYP7B1;CYP7B1	0.000117986	0.328068792
cg06738040		0.00043997	0.328063824
cg19516404	ZIC4;ZIC4;ZIC4	2.58E-05	0.328052724
cg20012308		0.000233422	0.328047592
cg19567689		3.34E-05	0.328046549
cg05846716	PCSK2	1.50E-05	0.328041258
cg11111225	DPYSL5	4.34E-05	0.328040143
cg13319711		5.59E-05	0.328034537
cg07800320	ALDH1L1	9.24E-05	0.328034057
cg01486146		9.04E-05	0.328029281
cg11654011	CEACAM8	0.000148457	0.328019922
cg23593167	MOSC2;MOSC2	0.001639597	0.328011475
cg11445094		0.00746304	0.328006188
cg17833476	TLX3	1.50E-05	0.327997668
cg07586272	GPR83;GPR83	0.001639597	0.327981022
cg17953577	DNAH9	8.60E-06	0.327974625
cg14124894	VASH1	3.34E-05	0.327972551
cg10022526	BDNF	7.23E-05	0.327928474
cg24410535	FAT1	0.003734675	0.327910725
cg13929566	SYNE1;SYNE1	3.34E-05	0.327909726
cg12805420	CLDN7	1.11E-05	0.327901886
cg00412772	YIF1B;YIF1B;C19orf33;YIF1B;YIF1B;YIF1B;YIF1	0.000148457	0.327894692
cg24419324		0.000537905	0.32788491
cg18279004		0.00043997	0.327882227
cg17353412	HOXA2	9.24E-05	0.327862408

cg12487655		0.000187194	0.327842678
cg12925682	OSMR;OSMR	5.59E-05	0.327819841
cg14189391	DNMT3A;DNMT3A;DNMT3A	0.000654035	0.32779946
cg21880476		0.000955793	0.327796084
cg18848688	LIFR;LIFR	0.000537905	0.327784637
cg04962621	MGRN1;MGRN1;MGRN1;MGRN1	0.000791389	0.327777197
cg00758915	PCDHGA4;PCDHGA6;PCDHGA1;PCDHGA5;PC	0.000537905	0.32777599
cg27166577	ELOVL4	1.50E-05	0.327774108
cg20533957	FUT4	0.000148457	0.327766151
cg19532307	SLITRK3	0.000117986	0.327757557
cg11702896		0.000187194	0.327754287
cg08874747	FAT1	0.003189589	0.327734686
cg17854471	GABRD	1.50E-05	0.327730752
cg06235653	DNASE1L2	3.34E-05	0.327721137
cg10679266	DEFA4	1.96E-05	0.327710283
cg13892954	PRKAA2	0.000654035	0.327704281
cg19453991		5.59E-05	0.327702237
cg03529189	SRGAP1	0.000290372	0.327665005
cg20622089	CNR1;CNR1;CNR1;CNR1	0.002714607	0.327629574
cg01106338		1.11E-05	0.327626576
cg14274989	EN1	7.23E-05	0.327612386
cg16151538	TRPC4AP;TRPC4AP	2.58E-05	0.327612255
cg01601658		7.23E-05	0.327612014
cg15388804		0.000187194	0.327604545
cg15748490	FMN2	4.34E-05	0.327591961
cg04352304		0.000955793	0.327582732
cg19812826	FGF14	5.59E-05	0.327557114
cg22071224	LOC440925;SP5	5.59E-05	0.327540658
cg07808348	GREM2	0.001147425	0.327534171
cg00261569	C3orf1	0.000654035	0.32752685
cg21297704	MTUS2;MTUS2	0.00043997	0.327526085
cg08661007	ROBO1	0.000187194	0.327525139
cg09896900	ZNF229	0.001147425	0.32752332
cg05271255		0.000187194	0.327519395
cg26501369	EYA4;EYA4;EYA4	5.59E-05	0.327503942
cg17795240	GATA4	0.001479724	0.327495567
cg25260683	GRP;GRP;GRP	1.50E-05	0.327494357
cg02272576	LOC100132354	5.59E-05	0.327493587
cg10039299		1.50E-05	0.32747669
cg15175129	MSRB3;MSRB3	0.001948248	0.327471793
cg04836851	PCDHB16	0.000117986	0.327471521
cg10576257	TACR1;TACR1	0.000537905	0.327464165
cg14619064	MPO	4.34E-05	0.327461979
cg04272632	CSMD2	9.24E-05	0.32746094
cg23208176		1.11E-05	0.327460696
cg08173915	IFNGR2	0.000955793	0.327454428
cg09924064	ACCN1;ACCN1	0.0050758	0.327449616
cg24452591		1.11E-05	0.327447333
cg03319479		2.58E-05	0.327441263
cg25444002	FGF14;FGF14	4.34E-05	0.327439136
cg16697438		0.001147425	0.327436716
cg18506678		0.000148457	0.327424744
cg06943420	MMP23A;MMP23B	0.000290372	0.327412181
cg07317551	HPCAL4	9.24E-05	0.327378305
cg08677617		0.000187194	0.327365877
cg14004073	FAM159B	0.000654035	0.327351002
cg16735881	NBEA	2.58E-05	0.327341277
cg01526217		0.0050758	0.327311534
cg08823027	KCNE1;KCNE1;KCNE1	0.000791389	0.327299549
cg12516270	LYNX1;LYNX1;LYNX1;LYNX1;LYNX1;LYNX1	0.001376172	0.327295415

cg17183770	SKIL;SKIL;SKIL	0.000955793	0.327293104
cg18961944	GALR1	7.38E-05	0.327293045
cg03576469	CCDC8	0.000187194	0.327267058
cg15356923	FAM19A4;FAM19A4	3.34E-05	0.327266981
cg08999915		9.24E-05	0.327261828
cg14868703	LHX5	0.000187194	0.327237661
cg01197871		7.23E-05	0.327228078
cg13964068		7.23E-05	0.327216261
cg12044685	CYP26B1	0.000148457	0.327215646
cg17722002		9.24E-05	0.327213041
cg27125044		9.24E-05	0.327193378
cg23985374	PCDHA6;PCDHA2;PCDHA1;PCDHA9;PCDHA7	1.50E-05	0.327187906
cg10012059	STAP2;STAP2	0.000791389	0.327180844
cg14611683	EIF2B3;EIF2B3	2.58E-05	0.327177093
cg21226442	C12orf11	1.50E-05	0.327171084
cg02051616	NKX2-5;NKX2-5;NKX2-5	5.59E-05	0.327157423
cg05928849	ASAM	0.000791389	0.327143662
cg17430979	JAKMIP1	1.96E-05	0.327143211
cg25010752		9.24E-05	0.327135375
cg13891181		3.21E-05	0.327105814
cg16646616		0.000290372	0.327099197
cg14620572	GPR144	8.60E-06	0.327085177
cg11838299	NLRP14;ZNF214;NLRP14	2.58E-05	0.327073075
cg16736896		0.000187194	0.327046589
cg25905812	DMRT1	1.96E-05	0.327031268
cg14934413		5.59E-05	0.327029686
cg08830758	RERG;RERG	0.000187194	0.327016756
cg13882309	PCDHB13	8.60E-06	0.327007338
cg16509658	GABRB2;GABRB2	0.000334462	0.326995807
cg02579377		0.000654035	0.326992143
cg05855588	KL	0.002304201	0.326991601
cg15229275	HIF3A	0.001376172	0.326985667
cg11252792		5.59E-05	0.326981044
cg03199745	FLJ12825	4.34E-05	0.326962509
cg07139284		0.000187194	0.326957949
cg26275470		2.58E-05	0.326942925
cg23414595	ZMAT5;ZMAT5	3.34E-05	0.326940848
cg20242210	PKIB;PKIB;PKIB	0.000233422	0.326927715
cg06198892		5.59E-05	0.326924712
cg07599881		9.24E-05	0.32692031
cg08064488		0.000148457	0.326913922
cg01229658		0.000357885	0.326902278
cg15644413	C1orf216	2.58E-05	0.326890718
cg22869030		0.000148457	0.326888775
cg02707307		0.000233422	0.326888194
cg24127719		0.000148457	0.326878106
cg26226802	GLRA3;GLRA3	0.000233422	0.326877696
cg27138281	ME1	0.000365373	0.326860141
cg16658618	MYBPC1;MYBPC1;MYBPC1;MYBPC1	4.34E-05	0.326860101
cg14497054		8.60E-06	0.326857052
cg11399409	GBA;GBA;GBA;GBA;GBA	0.000148457	0.32685248
cg21928095		0.001376172	0.326841151
cg17811778	NKX2-3	1.50E-05	0.326818618
cg16601569	ZNF528	9.24E-05	0.326815084
cg20060685	NUAK1	0.000187194	0.32681071
cg14770293		0.001948248	0.326809616
cg19439399	ELOVL4	1.50E-05	0.326792994
cg18107232		0.000357885	0.326790457
cg10030921	MORN4;MORN4	3.34E-05	0.326786409
cg07203423	TLX2	0.000955793	0.326772792

cg20479774	DLX6AS;DLX6	0.0050758	0.326714534
cg09558547		0.000290372	0.326704463
cg27274542	NPEPL1	0.00043997	0.326704317
cg15717183	SOX2OT	8.60E-06	0.326700984
cg20061010		0.000117986	0.326690219
cg07388347		0.005897668	0.326674192
cg11599680		5.59E-05	0.326672704
cg07696699	FAM184B	0.003189589	0.32666709
cg09342940		3.34E-05	0.326662284
cg21599215	LOC150786	3.34E-05	0.326658147
cg20927119	ACTR3C;ACTR3C;ACTR3C	0.001376172	0.32665552
cg20942162		4.34E-05	0.326645287
cg09014354	NEUROD4;NEUROD4	5.59E-05	0.326644506
cg27615578	LRRC8E	7.23E-05	0.326643916
cg24882324	TNXB	0.000654035	0.326635432
cg12348588	BTBD17	0.000654035	0.326605391
cg24076884	PCDHAC2;PCDHA7;PCDHA12;PCDHA6;PCDH	4.34E-05	0.326594548
cg07248310	HOOK1;HOOK1	1.96E-05	0.326589761
cg19795292	IL1RL1;IL1RL1	2.58E-05	0.32658683
cg24848035	RGS22	0.000290372	0.326583011
cg07590961	DPP6;DPP6;DPP6	8.60E-06	0.326580556
cg07007400	KRT7	4.34E-05	0.326568514
cg23547017	HOXD3	9.24E-05	0.326568293
cg24098603	C10orf79	0.007869847	0.326561621
cg08675869	NR2F2;NR2F2;NR2F2;NR2F2;NR2F2	0.00043997	0.326557397
cg10208610	TRNP1	0.000187194	0.326555107
cg06286962	FAT1	0.000233422	0.326544549
cg15511381		2.58E-05	0.326532321
cg09415754		8.60E-06	0.326531655
cg21234265		0.000148457	0.326527764
cg06562865	GALR1	0.000537905	0.326518674
cg15972331	FGF14;FGF14	0.000148457	0.32651403
cg02767771	UTF1	0.001147425	0.32650707
cg09086835	LOC254559	3.34E-05	0.326507032
cg01070355	NRSN1	7.23E-05	0.326506337
cg21121136		0.001147425	0.3265027
cg27009703	HOXA9	0.003189589	0.326500117
cg09476130	CCDC19	4.34E-05	0.326497955
cg19613432	EN1	3.34E-05	0.326497754
cg00567749	VCAN;VCAN;VCAN;VCAN	0.003734675	0.326491955
cg20907614		0.000148457	0.326483651
cg12503575	OGDHL;OGDHL;OGDHL	5.59E-05	0.326482199
cg10133935	DAZAP1;DAZAP1	2.58E-05	0.326478379
cg20309121		0.001376172	0.326476911
cg26147845	EP400	0.006823935	0.326473153
cg23024158	C10orf11	0.000148457	0.326445743
cg04424443	DST;DST;DST;DST;DST	0.001376172	0.326423967
cg14291663		0.000357885	0.326407165
cg25221235		4.34E-05	0.326406364
cg05967001	RPH3A;RPH3A;RPH3A;RPH3A	4.34E-05	0.326346998
cg27283345	TFAP2A;TFAP2A	2.58E-05	0.326343135
cg14283758		0.000537905	0.326338496
cg26972272	OTX2OS1	4.34E-05	0.3263378
cg22963554	KIAA1024	7.23E-05	0.326324642
cg24996161	PCDHGA2;PCDHGA4;PCDHGA1;PCDHGB1;PC	1.50E-05	0.326316246
cg16395183	HHAT;HHAT;HHAT;HHAT;HHAT;HHAT	0.000117986	0.326306557
cg24174665	XKR4	0.000187194	0.326306492
cg20633875	KPNA6	0.000537905	0.326287077
cg10523903		1.50E-05	0.326280349
cg12664940	PCDHB1	0.002450672	0.32626734

cg07916589		8.60E-06	0.326250441
cg09797577		0.00043997	0.326237372
cg07762181		0.000148457	0.326227276
cg08864344		0.000187194	0.326215364
cg03805492		2.58E-05	0.326201729
cg04565261		0.003189589	0.326191576
cg18537001	EPHA10;EPHA10	0.000148457	0.326174878
cg14042396		1.11E-05	0.326158005
cg00472814	ADAMTS1;ADAMTS1	0.000187194	0.326155008
cg02805920	FLRT2	1.96E-05	0.326154447
cg12973591	TFPI2	0.00043997	0.326152309
cg20910807	EDN3;EDN3;EDN3;EDN3	0.000955793	0.326151136
cg12471986	ZNF214;NLRP14;ZNF214	1.50E-05	0.326148437
cg19524362	CALCR;CALCR	0.000290372	0.32614026
cg20634738	ANO5;ANO5	4.34E-05	0.326138081
cg13426307	KIF12	0.000290372	0.326119718
cg06086731		3.34E-05	0.326107386
cg22003366	PRR16;PRR16	0.001376172	0.326063711
cg10660916	SLC5A5	9.24E-05	0.326057535
cg19246007	COL21A1;COL21A1	1.50E-05	0.326038324
cg21607030	EYA4;EYA4;EYA4	0.001639597	0.32601194
cg17588578	PCDHGA2;PCDHGA3;PCDHGA1;PCDHGA3	0.000117986	0.326001965
cg13264399	EVX2	8.60E-06	0.325996969
cg10833037	LOC100128811;GPR158	2.58E-05	0.325996711
cg15397448	MAP9	0.003189589	0.325992307
cg02625902	MAST4;MAST4	0.004357816	0.325992216
cg00541476	SULF1;SULF1;SULF1;SULF1	3.34E-05	0.325990438
cg06746829	PADI2	0.006823935	0.325990262
cg09522056		5.59E-05	0.325981491
cg11320910		5.59E-05	0.32595456
cg11772171	ASCL2	0.003734675	0.325943587
cg22070855	GHSR;GHSR	0.000187194	0.325914566
cg21790851	KLF5	0.000233422	0.325911105
cg06967120	LOC100128811;GPR158	8.60E-06	0.325908309
cg10008589		0.003189589	0.325885828
cg22757824	COL4A1;COL4A2	0.002304201	0.325880623
cg20101352	CPVL;CPVL	0.00043997	0.325880139
cg27564108		0.001147425	0.325878594
cg17344091	TRIM27	0.003734675	0.325876874
cg24176563	EYA4;EYA4;EYA4;EYA4;EYA4;EYA4	0.001147425	0.325860833
cg20772101	EGFLAM;EGFLAM	0.001639597	0.325836256
cg09772792		0.000233422	0.325817194
cg10239388	WBSCR17	0.004357816	0.325816073
cg18725867	GDNF;GDNF;GDNF	0.003734675	0.325806937
cg12345526		0.000187194	0.325803597
cg13763503	CTSL2;CTSL2	0.000148457	0.32580204
cg22859727	FLRT2	0.001376172	0.325799417
cg03370738	GRID1	0.000187194	0.325781804
cg04536807	FKBP9	0.003189589	0.325777899
cg03593823	GREM2	0.001147425	0.325773916
cg10500219	MED8;C1orf84;MED8	0.000537905	0.325772392
cg26099134	SIM1	8.60E-06	0.325771048
cg05385434	EPHX1	0.000117986	0.325762656
cg25987744	CCDC8;CCDC8	0.00043997	0.325738838
cg21567504	OCA2	0.000117986	0.325729359
cg05871997	BEND6;DST	0.004357816	0.325728901
cg22161476	FSTL5;FSTL5;FSTL5;FSTL5;FSTL5;FSTL5	1.96E-05	0.325720033
cg07891473	ADAMTS9	0.000290372	0.325713364
cg11985360	GSC2	0.000187194	0.32571038
cg19867991	CALR	5.59E-05	0.325698966

cg16976870		0.000654035	0.325697162
cg01790346	SAMD12;SAMD12	0.000233422	0.325683038
cg25655234	SORCS2	0.00041966	0.325678899
cg04087740	GPR27;EIF4E3;EIF4E3	0.000233422	0.325661364
cg15968604	C5orf52	0.000233422	0.325658673
cg13698691	TNXB	0.000654035	0.325657047
cg09396865		9.24E-05	0.325640116
cg20937016	GALNTL1;GALNTL1	0.000117986	0.325624686
cg26958615		1.96E-05	0.325624149
cg06804921	SLC6A5	4.34E-05	0.325624057
cg00303982	SCRT1	0.000117986	0.325622048
cg04949206	HOXD11	1.96E-05	0.325613917
cg14238959		0.000148457	0.325612235
cg27141474	C2orf58	0.000117986	0.325612024
cg19656577	PRMT8	0.002927025	0.325604399
cg01708273	HOXD11	0.002714607	0.325602193
cg08426444		1.11E-05	0.325597408
cg26692294	UBE2QL1;UBE2QL1	0.001639597	0.325591067
cg23030278	PYGO1	1.11E-05	0.325543299
cg22353329	CBX4	0.000117986	0.325530594
cg13661968	SLC6A5	0.000187194	0.325528711
cg15266705		0.000290372	0.325517476
cg20432960	TFAP2B	0.000117986	0.325513701
cg22899100		1.96E-05	0.325513591
cg08663552		9.24E-05	0.325497961
cg04550697	EXOC6B	2.58E-05	0.325488564
cg21047695	NKAIN3	0.000117986	0.325480707
cg14329157	WDR69	8.60E-06	0.325480497
cg01357892	ZXDC;ZXDC	0.000537905	0.325459338
cg05575505		7.23E-05	0.325454629
cg03297593	MYEF2	0.001147425	0.32545145
cg04374393	SOX14	1.96E-05	0.325439222
cg03521696	HLA-G	0.000187194	0.325434075
cg15788369		1.50E-05	0.325429993
cg14443519	HCG4	0.00043997	0.325422102
cg02260587	PCDHB2;PCDHB2	2.58E-05	0.325406576
cg16439198	CYP1B1	4.34E-05	0.325397249
cg01052879	MIR124-3	0.000955793	0.325389387
cg03580568	EDNRA;EDNRA;EDNRA	9.04E-05	0.325381533
cg01450522		0.000148457	0.325366598
cg10736454		0.000791389	0.325360004
cg10156366		0.005897668	0.325359201
cg22451233	PDGFC	0.00043997	0.325351074
cg09307788		8.60E-06	0.325347087
cg07125112	FRMD5	5.59E-05	0.325338697
cg00979288	PCDHB2	1.11E-05	0.325331404
cg10899301	TFAP2A;TFAP2A	0.000233422	0.325320441
cg17438849	HECW1;HECW1	0.000537905	0.325306978
cg14289429	FAM78A	0.000148457	0.325299728
cg08553437	TMEM155;LOC100192379	0.000654035	0.32527594
cg04282138		2.58E-05	0.325275694
cg01446217	ERMP1	0.00043997	0.325272023
cg04174180	ZNF23	9.24E-05	0.325261848
cg06388730		4.34E-05	0.325251617
cg14326671	LOC100192378	0.000117986	0.325247905
cg00946992		2.58E-05	0.325237248
cg02763800		1.50E-05	0.325230036
cg16483916	PLAC2	0.000290372	0.325219927
cg23497217	BDNF;BDNF;BDNF;BDNF;BDNF;BDNF;BDNF;B	0.000117986	0.325219207
cg15475851	INA	0.000187194	0.325219084

cg16105558	KCND2	0.00043997	0.325208136
cg23143978	RBM33	0.000955793	0.325200797
cg05408649	HOXC4	0.003734675	0.325187917
cg01534145	CREB5	0.004357816	0.325175355
cg11674865		0.000955793	0.325162646
cg02054108	USP34	1.11E-05	0.325154712
cg16435779	GRHL2	7.23E-05	0.325150101
cg01146063	CTNNA2;CTNNA2	5.59E-05	0.325146763
cg22030890	RAB32	0.000117986	0.32514558
cg05352087	LHX8	5.59E-05	0.325145448
cg24172509	PCDHGA4;PCDHGB5;PCDHGA6;PCDHGA1;PC	0.001639597	0.325138859
cg13496323	HMCN1	0.000290372	0.325131202
cg00142402		4.34E-05	0.325125272
cg24406240		0.001376172	0.325112631
cg22636108		5.59E-05	0.3251106
cg01044025		0.000233422	0.325093321
cg14054620	C9orf122	0.002304201	0.325080434
cg15385562	FBN1	0.002304201	0.325079801
cg19431274		0.000357885	0.325078495
cg22508969	HMGCLL1;HMGCLL1	0.000187194	0.325076344
cg12640000	L1TD1;L1TD1	8.60E-06	0.325070333
cg06733794	TACC2;TACC2;TACC2;TACC2	0.000117986	0.325057891
cg16713889	FAM161B	0.004357816	0.325052595
cg24502330	CDA	0.000148457	0.325048094
cg18731327	ZIC4;ZIC4;ZIC4	2.58E-05	0.325042306
cg15971010	SLC47A1	0.00043997	0.325030496
cg12095167	RAB2A	1.96E-05	0.32502209
cg00126320		0.000290372	0.325020205
cg21354781		0.000117986	0.325012826
cg07193041	ERC2	0.00043997	0.32500921
cg15855970		1.50E-05	0.32500789
cg20531020	SLC16A3;SLC16A3	2.58E-05	0.325004471
cg11070172	CEBPE	0.000233422	0.325000085
cg01369406	ANKRD5;ANKRD5	0.00043997	0.324992676
cg00037441	RFX6;RFX6	1.96E-05	0.324987143
cg12749559	BRP44;BRP44;BRP44	0.000791389	0.324986306
cg25602684	GDNF	0.001147425	0.324985613
cg22153407	GALNT2	0.000290372	0.324967579
cg16108059	TSHR;TSHR;TSHR;TSHR;TSHR;TSHR	0.001639597	0.324960235
cg03009871	MAGI2	2.58E-05	0.324958994
cg06132727		0.000654035	0.324946575
cg19370684	PRKCZ	0.006823935	0.324942855
cg05317851		4.34E-05	0.324939815
cg21188710		0.001639597	0.324929248
cg14845216	RASGEF1A	2.58E-05	0.324923343
cg21990700	LOC283314;C1RL	0.000955793	0.324923294
cg14221171	TAC1;TAC1;TAC1;TAC1	4.34E-05	0.324917434
cg07861790		1.50E-05	0.32491365
cg09556138	EPHA4	0.000791389	0.32490924
cg00180447	CHST13	2.58E-05	0.324905951
cg15444947	TRIL	0.002304201	0.324892159
cg19640130	RTKN2	0.000290372	0.324876666
cg27340506	PSKH2	5.59E-05	0.324868318
cg10425624	TTC8;TTC8;TTC8	0.000537905	0.324864128
cg10171347	NKX6-2	1.50E-05	0.32486059
cg02058108	SSPN;SSPN	0.000290372	0.324856708
cg02639108		8.60E-06	0.324855276
cg21615583	MIR375	0.001147425	0.324851934
cg11398020	KLF5	0.000148457	0.324833019
cg25713185	SLC19A3	0.00043997	0.324816818

cg07290552	FAM164A	0.000654035	0.324813936
cg17095728	TRIM67	3.34E-05	0.324811653
cg10265570	RTBDN	9.24E-05	0.324811461
cg03471150	IPO9	4.34E-05	0.324807755
cg18759102	PRDM16;PRDM16	0.00043997	0.324801345
cg09418063	ZNF208	0.000537905	0.324800183
cg24775496	FLRT2	0.001376172	0.324795266
cg02622052	CNTN1;CNTN1	7.23E-05	0.324771098
cg15552461	RDH13	0.000233422	0.324768353
cg11104510	ETV1;ETV1;ETV1;ETV1;ETV1;ETV1	0.000537905	0.324767537
cg00235367	ZIC4;ZIC4;ZIC4;ZIC4;ZIC4	1.11E-05	0.324758673
cg13804575	ZNF462	0.001639597	0.324756383
cg01194057	UNC5C	0.001948248	0.324755533
cg04299135	BARX2	0.003189589	0.324738357
cg06497198	NHLRC1	0.000537905	0.324723755
cg00353773	CXCL12;CXCL12;CXCL12	0.003734675	0.324719407
cg17304646		3.34E-05	0.324706546
cg24682551	SLC22A15	0.00043997	0.324686261
cg07917609	RXFP3	1.11E-05	0.324682227
cg11518846	EYA4;EYA4;EYA4	0.000654035	0.324679997
cg10323921		1.11E-05	0.324664518
cg17510653	ENTPD7	4.34E-05	0.324660279
cg26468007	MMD2;MMD2;MMD2;MMD2	7.23E-05	0.324656316
cg13371951	GRM1;GRM1	0.000117986	0.324650721
cg16510548	LOC401463	0.000290372	0.324646299
cg26610624	PRKCZ	0.002304201	0.32464202
cg11670519	BMP7;BMP7	0.000334462	0.324640687
cg16015712	ZYG11A	0.000537905	0.324635114
cg01371155	ESRRG;ESRRG;ESRRG;ESRRG;ESRRG	0.000148457	0.32462948
cg13470069	RAB5C;RAB5C	0.001376172	0.324620646
cg13770461	PHF21A;PHF21A	9.24E-05	0.32461821
cg17171215		5.59E-05	0.324613447
cg15108641	UBTD1	0.00043997	0.324607372
cg04106782		0.000122184	0.324606575
cg07634645	PLBD1	4.34E-05	0.324586203
cg17691292	ESRP1;ESRP1;ESRP1;ESRP1;ESRP1	0.000187194	0.324562868
cg23444265	GRIN2D	9.24E-05	0.32455977
cg12196232	NRG1;NRG1;NRG1;NRG1;NRG1;NRG1;NRG1;	8.60E-06	0.324559188
cg15905435		0.000148457	0.324552466
cg19538655	PAX9	0.000187194	0.324520659
cg18482268	POU4F3	0.000290372	0.324519967
cg16373229	FEZF1;FEZF1	8.60E-06	0.324518311
cg14251622	INSM2	0.00043997	0.324500458
cg08318212	EDN3;EDN3;EDN3;EDN3	0.003189589	0.324480779
cg27517681	TBX15	0.000148457	0.324478824
cg25936147	HOXC13	1.11E-05	0.324475098
cg15433534		0.003734675	0.324465087
cg17617843	C1QTNF3;C1QTNF3	0.001639597	0.324452493
cg05947443	PLA2G7;PLA2G7	5.59E-05	0.324448783
cg01774159	STMN2	0.000148457	0.324440626
cg08488841		7.23E-05	0.324432762
cg10245910	FAM155A	2.58E-05	0.324429895
cg23950724	SYT10	1.11E-05	0.324429754
cg13466002	ARRB2;ARRB2	0.000654035	0.324426011
cg15396686	HMX3	0.000117986	0.324424348
cg10580056	ACTC1	0.000122184	0.324392083
cg16063747	EMX2OS	0.000117986	0.324386488
cg16840358		3.34E-05	0.324386181
cg13776340	ELOVL4	4.34E-05	0.324358706
cg10334354	EPHA5;EPHA5	7.23E-05	0.324357418

cg04603419	PYY	7.23E-05	0.32435129
cg26668837	STK33;STK33	8.60E-06	0.32434988
cg13564825	PPP1R14A	3.34E-05	0.324347319
cg21639114		0.000148457	0.324344979
cg08145590	C3orf15	0.000187194	0.324341641
cg27382568		5.59E-05	0.32434045
cg16919517	GATA5	0.000654035	0.324331148
cg27170985		1.50E-05	0.324305244
cg03388789	ZIC4;ZIC4;ZIC4	5.59E-05	0.324296117
cg13035910		0.000290372	0.32429342
cg20732703	GRB10;GRB10;GRB10;GRB10	0.000357885	0.324289144
cg04986504	DKK3;DKK3;DKK3	0.001948248	0.324287171
cg23967169	FAM19A4;FAM19A4;FAM19A4;FAM19A4	5.59E-05	0.324267523
cg05059304	SYCP2L	0.000148457	0.324265418
cg21081803		7.23E-05	0.324260981
cg02512226	ARMC4	0.002714607	0.324259647
cg17852032	ZDHHC14;ZDHHC14	0.000290372	0.324253551
cg04982308	BTBD17	0.000357885	0.324250411
cg25213546	SH3GL3;SH3GL3	8.60E-06	0.324248406
cg24650501	LMX1A;LMX1A	1.50E-05	0.324224852
cg18532727	C17orf51;C17orf51	0.002714607	0.32422411
cg15086439	EDARADD;EDARADD	0.001376172	0.324199722
cg04484947	ZBTB8B	0.000148457	0.324186882
cg14041338	ENOX1;ENOX1	0.000187194	0.324184439
cg05143123	SPOCK1;SPOCK1	0.000791389	0.32417832
cg26757221		1.96E-05	0.324164603
cg03839794	NPAS3;NPAS3;NPAS3;NPAS3	1.50E-05	0.324129616
cg00315391	SCNN1G	0.000654035	0.324128614
cg26591696	COL5A3	4.34E-05	0.324128376
cg06671450	PAX5	0.000148457	0.324117294
cg23211240	MIR34B;BTG4;MIR34C	0.000537905	0.324117241
cg21527621	ACOT11;ACOT11	0.000357885	0.324110296
cg02994956	NEFH	2.39E-05	0.324107483
cg24748191		3.34E-05	0.324099152
cg15423872	FAM110B	9.24E-05	0.324099119
cg04470072	PSKH2	0.000117986	0.324078096
cg10197432	C6orf103	0.000791389	0.324075818
cg10423771		0.000187194	0.324062229
cg08141553	GALNT2	1.11E-05	0.32406174
cg14932016	ACTN2	0.000187194	0.324053792
cg06528267	VWC2	0.000955793	0.324037964
cg18342026		1.50E-05	0.324025114
cg04087271	CDA	0.000791389	0.324022199
cg09970569	RSPO2	1.50E-05	0.324020075
cg07935500	ARNT2	0.000791389	0.324012578
cg13948585	CACNA1E	0.000148457	0.323999568
cg02340541	GAL	0.001639597	0.323975874
cg21029612	LOC254559	2.58E-05	0.323970622
cg04295372	VCAM1;VCAM1	0.000148457	0.323962051
cg26776269		4.34E-05	0.323938783
cg10556349		0.000187194	0.323922848
cg12765028		1.50E-05	0.323918405
cg12173409		7.23E-05	0.323915628
cg05059291		0.000262362	0.323906732
cg00673191	DOPEY2	9.24E-05	0.323905697
cg14811608	NPY2R	2.80E-05	0.323903049
cg07160932	SEZ6L2;SEZ6L2;SEZ6L2;SEZ6L2	0.000537905	0.32389472
cg08032135	NRG1;NRG1;NRG1;NRG1;NRG1;NRG1;NRG1;	9.24E-05	0.323894525
cg10554264	PTPRN2;PTPRN2;PTPRN2	0.000187194	0.323888985
cg24115032	APCDD1L	8.60E-06	0.323868718

cg08577743	C9orf122;ANKRD18A	0.000117986	0.323864538
cg23733133		3.34E-05	0.323858915
cg04455164	DPY19L2	0.000654035	0.32385379
cg22855255	CA10;CA10;CA10	0.000117986	0.323849682
cg16745446	FAM189A1	0.000233422	0.323835458
cg24541426	HOXD3	0.000117986	0.323828214
cg14582763	ZNF578	0.000357885	0.323821111
cg17130457		0.006823935	0.323809722
cg22271921	ZMAT4;ZMAT4	1.50E-05	0.323798831
cg11418595	HOXD1	0.001948248	0.323776486
cg18440199	LOC283392;TRHDE;LOC283392	0.000357885	0.323768469
cg19677302	PKP2;PKP2	9.24E-05	0.323743856
cg21656751	HECW1	0.000117986	0.32374155
cg25203481	KCNQ1DN	0.000955793	0.323737205
cg19935951	LRBA	7.23E-05	0.32373424
cg15948088	C11orf87	7.23E-05	0.32372895
cg06152533	LRRN1	9.24E-05	0.323725845
cg17862332	ARMC4	5.59E-05	0.323707925
cg05256504		7.23E-05	0.323698522
cg25237016	SPTBN4	1.50E-05	0.323697398
cg09734761		0.000357885	0.323691174
cg00922781	ADAM12;ADAM12;ADAM12;ADAM12	8.60E-06	0.323689606
cg03744383	CES1;CES1;CES1;CES1;CES1;CES1	4.34E-05	0.323689099
cg22334880	RPP21	1.96E-05	0.323684517
cg02867763	PCDHGA4;PCDHGA4;PCDHGA2;PCDHGA1;PC	1.96E-05	0.323673119
cg22127848		7.23E-05	0.323641869
cg19509715		0.000537905	0.323641708
cg25338454	ITPR2	0.001639597	0.323621382
cg07820868	TBX15	0.003189589	0.323592707
cg07278054		1.96E-05	0.32358641
cg06048524	CXCL12;CXCL12;CXCL12	0.005897668	0.32358345
cg21921460	CTNND2	4.34E-05	0.323569726
cg05396212	KRT19	9.24E-05	0.323560027
cg13687570	HKR1	0.000117986	0.323557196
cg10043253	SLC13A5;SLC13A5	0.003734675	0.323551715
cg07712198		0.00043997	0.323549598
cg06749715	PAX9	9.24E-05	0.323535849
cg15842276	MTNR1B	1.50E-05	0.32353046
cg12363682	GRM6	0.001147425	0.323520871
cg13686115	PHYHIPL;PHYHIPL	1.50E-05	0.323511179
cg18739675		0.001147425	0.323505093
cg17029354		1.50E-05	0.323504035
cg12530050	TBX3;TBX3;TBX3;TBX3	0.000233422	0.323492799
cg22435791		0.000187194	0.323484004
cg00455327	CABLES1	0.001147425	0.32347565
cg21635854		0.000290372	0.32346565
cg27262041	NAV2	7.23E-05	0.323442325
cg20132590	PDZRN3	1.50E-05	0.323421601
cg18206027	VWC2;VWC2	0.001376172	0.32342121
cg03984209	ERC2	0.001393182	0.323420287
cg03339065	EMX2;EMX2OS;EMX2	0.00043997	0.323414694
cg14152139		5.11E-05	0.323398445
cg08632909	FADD	0.000357885	0.323398394
cg07201475		0.000357885	0.323398049
cg10371414		0.00043997	0.323393763
cg13000912	PRDM13	1.11E-05	0.323364792
cg06859084		4.34E-05	0.323364392
cg17191462	TFAP2D	3.34E-05	0.323364272
cg18222653		0.000537905	0.323364129
cg26814075	LEP	3.34E-05	0.323361791

cg18702820	SSPN;SSPN	0.000233422	0.323338733
cg13509363	C9orf172	1.11E-05	0.323338514
cg03164275	CDK5R2;CDK5R2	9.24E-05	0.323338186
cg22799321	TFPI2	5.59E-05	0.323325317
cg21972382	CLIP4	0.001147425	0.32329078
cg05756489	HTR7;HTR7;HTR7	9.24E-05	0.323288654
cg04937144		3.34E-05	0.323288215
cg08291024	LMX1A	9.24E-05	0.323281658
cg02032558		0.000654035	0.323274923
cg04168853		9.24E-05	0.323259156
cg03556669	TNXB	0.001061731	0.323249462
cg06838365	LHFPL4	2.58E-05	0.323245141
cg23031493	LHX5	0.000290372	0.323239251
cg12720643	FAM163A	1.11E-05	0.323237832
cg12853184	SLC30A9	0.001639597	0.323237186
cg14508391		7.23E-05	0.32322674
cg13059411	KCND3;KCND3	3.34E-05	0.323219358
cg13504245	NTRK2;NTRK2	0.001376172	0.32320866
cg04106092		5.59E-05	0.323198084
cg20576153	SIX6	4.34E-05	0.323183528
cg01316476		0.000791389	0.323182399
cg25979559	NHLH2;NHLH2	0.002714607	0.32315088
cg17894318	SOX2OT	4.34E-05	0.323112883
cg05477024	KIAA1804	1.96E-05	0.323099591
cg22190023	PCDHB7;PCDHB7	0.000357885	0.323097194
cg04457114		1.11E-05	0.323093838
cg11511795		0.003734675	0.323082423
cg04641171	RASGRF1;RASGRF1	7.23E-05	0.323058847
cg21867345	ZNF167;ZNF167	0.000290372	0.323039389
cg06262436	ISM1	0.001948248	0.323037854
cg26689483	QDPR	0.007873393	0.323028882
cg10112391	DPP4	0.000526175	0.323021945
cg01912921	FOXC1	0.000187194	0.323004664
cg10838385		1.11E-05	0.322995269
cg10726816		0.000117986	0.322991643
cg26998537	OTX2	0.000117986	0.322989399
cg12210040	LMO3;LMO3;LMO3	8.60E-06	0.322985039
cg14161359	KCNA4	0.000187194	0.322975603
cg13175739	STYK1	1.50E-05	0.322966157
cg01012879	ADSS	3.34E-05	0.322964955
cg26877720	FAM107B	0.000117986	0.322964731
cg01286685	RNF39;RNF39	0.000290372	0.322960842
cg26335633		0.000233422	0.322954005
cg05617469	ZIC5	5.59E-05	0.322950839
cg18371475	IRX2;IRX2;C5orf38;IRX2;IRX2	0.001147425	0.322933085
cg18138206	TCERG1L	4.34E-05	0.322928433
cg10261191	FEZ1;FEZ1	9.24E-05	0.322914083
cg06664357	DLL3;DLL3	0.000117986	0.322904919
cg11789820	SLC35F4	9.24E-05	0.322889724
cg03669782		2.58E-05	0.322889062
cg26143045	TMEM108;TMEM108	0.000357885	0.322886656
cg16994041	PRKAG2;PRKAG2	0.004357816	0.322883522
cg23726537	CRCT1	9.24E-05	0.322877244
cg20880339		1.96E-05	0.322852522
cg14783581	SOX14	1.96E-05	0.32284998
cg19802138	SOX1	0.000537905	0.322845321
cg25215292	FBXL21	0.000187194	0.322839152
cg17263974	PCSK6;PCSK6;PCSK6;PCSK6;PCSK6;PCSK6;I	0.000117986	0.322835617
cg14701867	ZNF365;ZNF365	0.000537905	0.322831318
cg24320612	GATA5	0.00043997	0.322824287

cg04745673		0.000233422	0.322794881
cg20640433	LAMA2;LAMA2	0.000187194	0.322781361
cg12538248	CA8	0.000955793	0.3227764
cg19356825	FLJ42875;PRDM16;PRDM16;FLJ42875	0.001376172	0.322760979
cg03700287	PCDHB19P	1.11E-05	0.322732087
cg17983957	AMICA1;AMICA1;AMICA1	0.000148457	0.322724723
cg15188182	C2CD4B	8.60E-06	0.322723643
cg14244013		4.34E-05	0.322722309
cg21196747		7.23E-05	0.322719089
cg09986292		9.24E-05	0.322716673
cg01229865	ATP11A;ATP11A	0.002714607	0.322716497
cg10148473	KCNC2;KCNC2;KCNC2	0.000233422	0.322715767
cg20996682	MAGI2	2.58E-05	0.322708107
cg13411784	WDR85	0.001376172	0.322701343
cg03365437	ALDH1A2;ALDH1A2;ALDH1A2;ALDH1A2	8.60E-06	0.32268051
cg15894431	YAP1;YAP1	0.003734675	0.3226789
cg27407935	SREBF1;SREBF1	0.000537905	0.322649913
cg06072036	PNPLA2	0.00043997	0.322637589
cg14304817	NAALAD2;NAALAD2	0.001061731	0.32262853
cg15829006	OLFM1;OLFM1	8.60E-06	0.322626164
cg01718139	VSTM1	0.002714607	0.322612281
cg05568930		0.000187194	0.322604133
cg02895156		8.60E-06	0.322579718
cg02558364	VAX2	0.001147425	0.322570857
cg14478163	ANKRD34B	5.59E-05	0.322570179
cg20130213	FRMD4A	1.50E-05	0.322561804
cg14372701	FAM181B	0.000233422	0.322559718
cg06617341		0.000357885	0.322556171
cg15623612	LSAMP	0.00043997	0.32254447
cg24617723	ZSWIM2	0.000117986	0.322542321
cg17178336	IHH	0.000357885	0.322516978
cg18174928	LIFR;LIFR	0.001376172	0.322476231
cg05928342	ZNF177	9.24E-05	0.322473617
cg26656658	P2RX7	3.34E-05	0.322465942
cg14531530	LIMCH1;LIMCH1;LIMCH1	0.000148457	0.322462093
cg23782083	ASH2L;ASH2L	0.000187194	0.322455676
cg14810013	EPHA5;EPHA5	1.50E-05	0.322453888
cg03478689	EDIL3	0.000290372	0.32242857
cg09313439	CDH2;CDH2	0.001376172	0.322410023
cg25781755		0.000955793	0.322403578
cg23795893	PGR	0.000233422	0.322369354
cg19342109	SYNE1;SYNE1	0.000148457	0.322359833
cg20995055	NELL2;NELL2;NELL2;NELL2;NELL2	0.00043997	0.322353996
cg23462956		0.000290372	0.322340832
cg21453451	SOX2OT	0.000148457	0.32233961
cg19605250	RAD21L1;RAD21L1	0.000233422	0.322334239
cg26435698		4.34E-05	0.322332883
cg17713376		7.23E-05	0.322330378
cg26820055	ARHGEF4;ARHGEF4	0.000187194	0.322320443
cg03609614	ZNF300;ZNF300	0.007873393	0.322305071
cg21638161	MAFA	0.000148457	0.322303393
cg16924616	DLX5	0.001639597	0.322299019
cg23489627	PDZRN3	8.60E-06	0.322282299
cg07859640	PCDHB3	9.24E-05	0.322276035
cg24333621	SLC35B4	5.59E-05	0.32227459
cg05680531	CHRM2;CHRM2;CHRM2;CHRM2;CHRM2;CHR[illegible]	3.34E-05	0.322264853
cg03304528	JAM3	0.000148457	0.322263138
cg00686880	RIPK4	1.11E-05	0.322262631
cg07577997	C1orf161	0.000357885	0.322260765
cg02169734	ELOVL4	1.50E-05	0.322242435

cg23889772	COL5A3	1.96E-05	0.322226307
cg20512711		0.001147425	0.322222993
cg10345326	LUZP2	0.001376172	0.322220047
cg17462793	KCNV1	1.50E-05	0.32221988
cg18199666	CHAT;CHAT;CHAT;CHAT;CHAT;CHAT;CHAT	2.58E-05	0.322217254
cg02790177	PPAPDC1A	9.24E-05	0.322214013
cg09792881	DMRTA2	0.000290372	0.322204493
cg01445809	KCNB1	0.000357885	0.322193946
cg05235392	HCN4	0.000233422	0.32217812
cg06270661	DNAH11	3.34E-05	0.322174338
cg02049955	SCN2B	0.000117986	0.322172242
cg06655623	RASGEF1A	0.000148457	0.322165581
cg00386408	TGFBI	0.00043997	0.322162285
cg02996413	LOC100192426;PTPRM;PTPRM	0.000791389	0.322160856
cg23213887	FBN2	0.000187194	0.322158776
cg03617826	PCDHGA2;PCDHGA2;PCDHGA2;PCDHGA1;PC	0.000148457	0.32215336
cg20209956	CCKBR	3.34E-05	0.322137757
cg25856888		0.000187194	0.322132363
cg14618996	ADAM32	1.50E-05	0.322128631
cg04583232	SLC17A6	4.34E-05	0.322109583
cg14579239	PCDHA6;PCDHA2;PCDHA1;PCDHA8;PCDHA7	0.000117986	0.322081355
cg02798621	FAM46A	0.001147425	0.322066506
cg15742412	CACNA1E	0.000148457	0.322065192
cg02893180	ADM2	0.006823935	0.322061091
cg15396570	DDAH1;DDAH1	4.34E-05	0.322051718
cg17720087		1.11E-05	0.322049897
cg09676860	RAB6C;RAB6C	1.11E-05	0.322047661
cg19902394	PPP1R3C	0.001948248	0.322044212
cg19829847	PITX2;PITX2	0.0050758	0.322030993
cg16309595		9.04E-05	0.322016399
cg13945578	NPY5R	3.34E-05	0.322014018
cg12718339	TMTC1	8.60E-06	0.322001029
cg26917540	ULK2;ULK2	0.00043997	0.321998361
cg00891541	SMPD3	3.34E-05	0.321995142
cg08690094		1.11E-05	0.321992067
cg01765529	CD164L2	0.001376172	0.321990246
cg02938414		8.60E-06	0.321986437
cg02801359		5.59E-05	0.321985583
cg22932815	NELL2;NELL2;NELL2;NELL2;NELL2	0.007873393	0.321976793
cg02486086		0.000791389	0.321959234
cg01654582	AADAT;AADAT	0.000117986	0.321954208
cg10264012		8.60E-06	0.321944886
cg06110297		5.59E-05	0.321942461
cg04675542	ZNF300	0.001948248	0.321935805
cg01902845	PROX1	5.59E-05	0.321930391
cg06743703		0.000187194	0.321883235
cg27562682		0.000117986	0.321860712
cg16415870	GRM7;GRM7	1.96E-05	0.321859899
cg26728709	ARSJ	0.000791389	0.321837279
cg07902749		0.007873393	0.321828612
cg18268562	FOXR1	1.96E-05	0.321817001
cg12782294	PLD1;PLD1	0.002714607	0.321791631
cg16051361	RAB21	0.000654035	0.321786565
cg02047300	PCDHA6;PCDHA2;PCDHA1;PCDHA1;PCDHA6	0.000357885	0.32178203
cg05190176	VDR;VDR	0.000148457	0.321778696
cg02919615	EFNA2	0.000117986	0.321774255
cg12214908	MEIS3;MEIS3	0.000233422	0.321764402
cg22736787	PTGFRN	0.004357816	0.321729751
cg11600734	ITPR1;ITPR1;ITPR1;EGOT	0.0050758	0.321726493
cg11715789	RUFY1;RUFY1;RUFY1	0.000148457	0.321724901

cg17071063	TRPC4;TRPC4;TRPC4;TRPC4;TRPC4;TRPC4	1.96E-05	0.321722156
cg14775296	MEIS1	5.59E-05	0.32171952
cg11288641	FAM3B;FAM3B	0.001147425	0.321719375
cg05065572	TBX5;TBX5;TBX5;TBX5	5.11E-05	0.321714214
cg11251858	ESR1;ESR1;ESR1;ESR1;ESR1	0.00043997	0.321711859
cg11275630	SLC8A2	0.000187194	0.3216965
cg10477905		0.003189589	0.321690744
cg14862395	CDH22	8.60E-06	0.321687105
cg16235748	ZC3H12D	0.000187194	0.321683873
cg08203352		7.23E-05	0.321681101
cg03955483	LYPD5	0.000290372	0.32166797
cg06664085	ARMC4;ARMC4	0.001147425	0.321661512
cg16544169	NKX2-6	4.34E-05	0.321661382
cg26504021	IRX2;IRX2;C5orf38	0.004357816	0.321653307
cg21975834		1.50E-05	0.321650557
cg02177231	TBX15	0.000791389	0.321647977
cg12479507		0.000955793	0.321646817
cg13253980	RBP4	0.000290372	0.321632917
cg25561913	ZNF503;C10orf41	0.000290372	0.321626447
cg04844267		8.60E-06	0.321621197
cg10574494	SPATA18	0.00043997	0.321620945
cg05258261	SPSB4	0.000117986	0.321594903
cg10152131		0.000955793	0.321585043
cg13890379	DOK6	5.59E-05	0.321577535
cg05263113		0.000187194	0.321564077
cg24647724	DNAH9	1.50E-05	0.321524183
cg24698655		7.23E-05	0.321522188
cg23657179	C10orf41;C10orf41	9.24E-05	0.321517822
cg05355225	CA3	3.34E-05	0.321512219
cg22579028	MIAT	1.96E-05	0.321504593
cg07877145		4.34E-05	0.321500185
cg09674215	TWIST1	1.11E-05	0.321490222
cg12627844	VPS54;VPS54	0.00043997	0.321490061
cg25584930	AP3B2	7.23E-05	0.321469678
cg26841967		4.34E-05	0.32146706
cg16589135		0.000290372	0.32146673
cg01891818	ICT1	1.96E-05	0.321465091
cg11054816	BACE1;BACE1;BACE1;BACE1	0.002714607	0.321421932
cg07883600	CACNA1D;CACNA1D;CACNA1D	0.001948248	0.321419908
cg19645221		0.000187194	0.321412568
cg08239804	SGK1;SGK1;SGK1	0.000117986	0.32139685
cg10644361	MIPOL1;MIPOL1	1.11E-05	0.321395041
cg03979241	EPB49;EPB49;EPB49;EPB49;EPB49;EPB49;EP	0.000148457	0.321393759
cg04991805	CRTAC1	0.000117986	0.321390055
cg05073035	ZIC1;ZIC1	0.000148457	0.321385544
cg00123055	SOX17	0.000357885	0.321351161
cg07613945	PCDHGA4;PCDHGA6;PCDHGA9;PCDHGA1;PC	0.00043997	0.321351095
cg05654711	DDAH1;DDAH1;DDAH1	4.34E-05	0.321325806
cg00545804	TSHZ3	0.001061731	0.321323613
cg22231902	EN1;EN1	7.23E-05	0.321311812
cg16516691	ZSWIM2	0.000187194	0.321310136
cg11754206	KCNB2	1.96E-05	0.32130422
cg16800708	DLK1	9.24E-05	0.321300161
cg02110182	SIX1	7.23E-05	0.321278468
cg16643151	GRID1	0.000187194	0.321276181
cg02723856		4.34E-05	0.321274862
cg24580066	OVOL2	0.001376172	0.321273902
cg17487784	CSGALNACT1;CSGALNACT1;CSGALNACT1	0.000654035	0.321266106
cg04392469	MOSC2	0.001376172	0.321253289
cg04095732		8.60E-06	0.321247907

cg08195279	NELL2;NELL2;NELL2;NELL2;NELL2	0.000262362	0.321247068
cg17163760	CPVL;CPVL	0.000791389	0.321237518
cg24410546	PNMA2	0.003734675	0.321234307
cg19495308	TRIM10;TRIM10	0.007873393	0.321232901
cg09038794	MOCS1;MOCS1	0.000187194	0.32122916
cg23681017	MFSD7	1.96E-05	0.321208965
cg02718825		8.60E-06	0.321205968
cg08248297	AP1M1;AP1M1	7.23E-05	0.321192274
cg17239558	PRDM16;PRDM16	0.000396647	0.321185925
cg03321003	EPHA3;EPHA3	0.003734675	0.321185755
cg18646851		0.000537905	0.321168405
cg15887459	LOC494141;LOC494141;LOC494141	9.24E-05	0.321168207
cg23664459	INSM2	0.000117986	0.321148989
cg13416310	GULP1	8.60E-06	0.321116329
cg05332960	C18orf34;C18orf34	0.000187194	0.321109499
cg15056556	RAB32	0.000233422	0.321108137
cg26492514	SLIT3	1.96E-05	0.321091984
cg07254032	HCN1	1.11E-05	0.321082427
cg22706610	PDE4D;PDE4D	0.000290372	0.321082377
cg11501236	LOC134466	3.34E-05	0.321057747
cg20809087	BRUNOL6;BRUNOL6	0.000955793	0.321053952
cg16368763		1.50E-05	0.321053625
cg25457832	EFCAB1;EFCAB1;EFCAB1	7.23E-05	0.321043025
cg18565355	ESRP1;ESRP1;ESRP1;ESRP1;ESRP1	7.23E-05	0.321030566
cg10124710		0.000187194	0.32101679
cg16530429	LIN28	9.24E-05	0.321014603
cg15988350	PDE1C	0.000654035	0.321012789
cg04871778	TTC22;TTC22	0.000187194	0.321007632
cg24903144	PAX2;PAX2;PAX2;PAX2;PAX2	2.58E-05	0.321000753
cg14049990	LPCAT1	0.000537905	0.32098408
cg04423976	PARM1	7.23E-05	0.320965819
cg22374861	PTPRO;PTPRO	0.000654035	0.320958168
cg23089840	LRRC3	0.000233422	0.320950952
cg09253581	LYPD5;LYPD5	0.000117986	0.320941916
cg20364632		0.004357816	0.320936662
cg23818870	C1orf114	0.000148457	0.320934584
cg10638780	ZNF560	1.50E-05	0.320915469
cg06391199	PTH2R;PTH2R	2.58E-05	0.320913375
cg18892446	ENC1	0.000955793	0.320897379
cg04991036	C14orf23;C14orf23	8.60E-06	0.32088633
cg20648847	ACTN3	0.00043997	0.320871472
cg12286573	C17orf104	1.11E-05	0.320855178
cg27449864	STX1A;STX1A;STX1A;STX1A	0.000117986	0.320846622
cg14042504	FOXA2;FOXA2	0.000233422	0.320844515
cg11685510		0.00043997	0.320839153
cg17215863	RALYL;RALYL;RALYL;RALYL	4.34E-05	0.320838569
cg13008174	NEIL2;NEIL2;NEIL2;NEIL2	0.000187194	0.320828315
cg16921083	SOX14	4.34E-05	0.320816018
cg27569863		5.59E-05	0.320798761
cg01796166		0.000654035	0.32079736
cg18164784	PCDHGA4;PCDHGA6;PCDHGA1;PCDHGA5;P(	0.009066563	0.320796619
cg16586442	GALNT11	5.59E-05	0.32079457
cg05636681	TBC1D12	0.000357885	0.320785952
cg06821199	CPLX1	0.001195582	0.320782362
cg15536490	TFAP2C	0.000117986	0.320782095
cg00384701		0.000654035	0.320774316
cg26802830	MUC12	2.08E-05	0.320765326
cg24051481	SOX21	3.34E-05	0.320752937
cg10511904	FGF12;FGF12	2.58E-05	0.320752463
cg18074184	SLC10A4	8.60E-06	0.320750683

cg09381701	SASH1;SASH1	2.58E-05	0.320746049
cg03301582	PON3	0.000148457	0.320732585
cg15399466		9.04E-05	0.320719903
cg12309653	LOC145845	1.50E-05	0.320701032
cg23042796	FLJ40330	0.000537905	0.320677476
cg17334018	UNC5C	0.000290372	0.320676572
cg08245702	VAX2	0.000955793	0.320674616
cg06817917	BMPR1A	0.000654035	0.32065865
cg24438644	ADRB3;ADRB3	1.11E-05	0.320642875
cg02008651	C14orf23;C14orf23	5.59E-05	0.320637132
cg23202979	CADM1;CADM1	4.34E-05	0.320625955
cg02783918	FOXF1	0.000233422	0.320625409
cg26311610	EIF4H;EIF4H	0.000654035	0.320608935
cg14308082		0.000955793	0.320608725
cg13642537		9.24E-05	0.32060531
cg01383911	TRIM39;TRIM39	5.59E-05	0.320600806
cg08404028	CHP2;CHP2	0.002304201	0.320579368
cg04971534	SEZ6;SEZ6	1.11E-05	0.320574258
cg13869872	HECW2	9.24E-05	0.320546842
cg11796455		9.24E-05	0.320516921
cg02778237		4.34E-05	0.320505641
cg23166773	SDCCAG8;AKT3	0.000148457	0.32049646
cg01246835		4.34E-05	0.320488879
cg05395302	VSTM2B	3.34E-05	0.320483342
cg00196671	DSC2;DSC2;DSC2;DSC2	0.003734675	0.320467504
cg21782409	UNC5C	0.000955793	0.320464145
cg17692125	BARX2	0.004357816	0.320463791
cg19357416	LAPTM4B	3.34E-05	0.320461908
cg06893379		3.34E-05	0.320456182
cg21082557	SCTR	0.000290372	0.320454923
cg06497668		0.000955793	0.32044641
cg09353063	OXTR;OXTR	4.34E-05	0.320440681
cg03126821	C8orf47;C8orf47	5.59E-05	0.320438223
cg18206952	DZIP1;DZIP1	9.24E-05	0.320433461
cg00446046		0.007873393	0.320429674
cg05140069	PDPN;PDPN;PDPN;PDPN	7.23E-05	0.32041309
cg02861380	LHX8	0.000148457	0.320407499
cg11649795	C11orf87	4.34E-05	0.320407062
cg05892030		0.000290372	0.320404859
cg15662768	HRH3	0.001147425	0.320401821
cg13816321		0.000654035	0.320385215
cg04281019		0.000233422	0.320384575
cg26445561	SLC36A4	7.23E-05	0.32037534
cg16570133		0.001147425	0.320371771
cg27416489	KIF5C	0.004357816	0.320363418
cg26529044	C13orf30	7.23E-05	0.320347362
cg07363137		0.000110797	0.320314274
cg18425434	C10orf82	0.000117986	0.32030282
cg09648702		0.000233422	0.320290481
cg24221541	PNMAL1;PNMAL1;PNMAL1;PNMAL1	0.007873393	0.320250763
cg01172965	KCNK13	0.000148457	0.320250088
cg07372602	PDK4;PDK4	0.003734675	0.320243673
cg08857144	ADD2;ADD2;ADD2;ADD2;ADD2	0.004357816	0.320235209
cg18784558		0.000148457	0.320234149
cg15830864	RNF220	2.58E-05	0.320215984
cg22235417	ZNF214;NLRP14	0.000233422	0.320210038
cg24002887		0.002714607	0.320199946
cg13969001	PAX7;PAX7;PAX7;PAX7;PAX7;PAX7	0.001948248	0.320199575
cg20312026	NPY1R	1.50E-05	0.320196288
cg13885201	ZNF23	0.000187194	0.320188458

cg01820539	SPHKAP;SPHKAP	1.96E-05	0.320186655
cg15964611	OPCML	0.001147425	0.320159224
cg09868780	LVRN	0.000148457	0.320157651
cg27030482		0.000290372	0.320155892
cg07919443		0.000357885	0.320154076
cg18579879	WNT16;WNT16	1.50E-05	0.320151059
cg13078563	GABRB3;GABRB3	5.59E-05	0.32014919
cg11554335	UBE2L6	0.000148457	0.32014535
cg16015423	SYDE1	0.002450672	0.320131301
cg24347663	ADD2;ADD2;ADD2;ADD2;ADD2	0.003734675	0.320121539
cg25800638	ITSN2;ITSN2;ITSN2	1.96E-05	0.320118097
cg07957995		2.58E-05	0.320100667
cg10997627	TAC1;TAC1;TAC1;TAC1	3.34E-05	0.320095353
cg18181196	ESRRG	4.34E-05	0.320094396
cg19358307		3.34E-05	0.32009404
cg14627743	FEZF1;FEZF1	1.96E-05	0.320086965
cg00336164	CCDC108;CCDC108	4.34E-05	0.320074651
cg09628838	C17orf102;TMEM132E	0.000357885	0.320062308
cg16315058	TBX5;TBX5;TBX5;TBX5	3.34E-05	0.320043146
cg14397318		2.58E-05	0.320040202
cg05351257	CCKBR	0.000654035	0.320039873
cg00546491	PDE1C	3.34E-05	0.320029398
cg13810562	MGC2889;HRASLS	7.23E-05	0.320029091
cg03348461	CAMTA1	0.000233422	0.320014697
cg14662355	LMX1A;LMX1A	0.000117986	0.319991309
cg01225004		0.00043997	0.319987809
cg00758296	PHF21B;PHF21B	7.23E-05	0.319987426
cg14681055	PITX3	0.00043997	0.319986402
cg22528044	SCN9A	0.004878663	0.319984434
cg21985590	RIPPLY2	8.60E-06	0.319974785
cg10290504		0.001376172	0.319973363
cg12927498	FCHSD1	0.00156564	0.319963118
cg00765830		2.58E-05	0.319926086
cg11002986		7.23E-05	0.319913943
cg05210412	CA4;CA4	0.000148457	0.319902458
cg00397635	ZYG11A	0.000233422	0.319883128
cg02841912	SYNE1;SYNE1	0.000955793	0.319880529
cg15174564	GRIK4	0.000537905	0.319877814
cg04711162	VSTM2B	0.001147425	0.319876183
cg18443378	WDR17;WDR17	7.23E-05	0.319835633
cg04474085		4.34E-05	0.319833494
cg22526139	RHCG	0.000117986	0.319830915
cg11277662	TSNARE1	0.000290372	0.319830047
cg25856090	CLCN1	9.24E-05	0.319823458
cg12668309	CACNA1D;CACNA1D;CACNA1D	0.001639597	0.319817748
cg05301866	TBR1	0.000290372	0.31981223
cg06528306	NPBWR1	0.000537905	0.319810994
cg23998119	ZDHHC22	0.002714607	0.319799663
cg05392364	DNAJA4;DNAJA4	0.005897668	0.319792356
cg26334507	ZBTB17	4.34E-05	0.319776537
cg12645220	PAK7;PAK7	1.11E-05	0.319769062
cg26948907	FOXC2;FOXC2	3.34E-05	0.319759916
cg18191162	KIF6	0.00043997	0.319755055
cg10850791	PABPC4L	4.34E-05	0.319750874
cg10363975	GPR126;GPR126;GPR126;GPR126	0.000290372	0.319745362
cg04575395	CRMP1	0.000187194	0.319737515
cg22883472	EBF2	2.58E-05	0.319732873
cg17241776	DLEU7	0.002927025	0.319732338
cg10135640	FAM176A;FAM176A	0.009066563	0.319718345
cg07103493	SLC27A6;SLC27A6;SLC27A6;SLC27A6	9.24E-05	0.319714793

cg22910449	SYT9	1.50E-05	0.319705228
cg00475820		8.60E-06	0.31970022
cg21127068	ZIC4;ZIC4	7.23E-05	0.319693862
cg17679453	WNT5A	3.34E-05	0.319687438
cg18335796	CALB1	5.59E-05	0.319682442
cg14807248	EPB41L4A;FLJ11235	0.000290372	0.319675494
cg12681948		7.23E-05	0.319656571
cg18482164	NEUROD2;NEUROD2	0.000117986	0.319655968
cg01056135	SERPINB13	0.002714607	0.319650757
cg18867004		9.24E-05	0.319644642
cg20575761	OTX2	3.34E-05	0.31964158
cg02307823	PTEN	0.001948248	0.319636955
cg11752275	GNLY;GNLY	0.000148457	0.319603073
cg05100070	SIM1	7.23E-05	0.319599197
cg16708451		2.58E-05	0.319592355
cg09528265	PPP2R2B;PPP2R2B;PPP2R2B;PPP2R2B;PPP2	0.00043997	0.319589043
cg19094243	TMEFF2	8.60E-06	0.319578911
cg06264984	CYP1B1	0.000187194	0.319572698
cg20157281	PCSK2	0.000233422	0.319570408
cg19165854	KIAA1024	0.00043997	0.319551421
cg13080379	TBX15	9.24E-05	0.319547421
cg26537209		0.001376172	0.319542958
cg02987482	MLLT1	0.000187194	0.319540532
cg18396987	SYCP1	0.000537905	0.319509364
cg20740903	TK1	0.000117986	0.319492851
cg20031656	FEZ1;FEZ1	0.000654035	0.3194909
cg14017655	EYA4;EYA4;EYA4	0.003734675	0.319489694
cg04878701		1.50E-05	0.319447754
cg22278296	RPRM;RPRM	0.000148457	0.319426654
cg20601919	CYP27A1;CYP27A1	0.000791389	0.319414939
cg12718440	MORN4;MORN4	2.58E-05	0.319410893
cg01618130	HS3ST6	9.24E-05	0.319405713
cg16959758		0.000117986	0.319397846
cg13907959	HIST3H2BB;HIST3H2A;HIST3H2A	7.23E-05	0.319396865
cg10501976		0.001147425	0.319393875
cg03062454	RNF160	5.59E-05	0.319390744
cg10647465	KIRREL3;KIRREL3	0.000955793	0.319389451
cg04095339	IRX4	8.60E-06	0.319376623
cg19170807		5.59E-05	0.319372427
cg18627360	MIR124-3	0.004357816	0.319368928
cg13890451		0.000187194	0.319365874
cg23641852	TSHZ3	0.00043997	0.319346495
cg22147084	GFPT2	0.00043997	0.319346115
cg25364343	CABYR;CABYR;CABYR;CABYR;CABYR;CABY	0.004357816	0.31934417
cg22667178	SERP2	1.11E-05	0.31934314
cg18693412	TOX2	0.000187194	0.319341689
cg26306909	C4orf19	0.000262362	0.319335738
cg08130988	EFEMP1;EFEMP1;EFEMP1;EFEMP1;EFEMP1	1.50E-05	0.319332421
cg13304825	TMEM105	0.000290372	0.319319677
cg06615678	LHX8	1.50E-05	0.319314526
cg07379434	VWC2	0.000357885	0.319309329
cg17307558	DKK2	5.59E-05	0.319308888
cg11228876	NID1	0.000537905	0.319301833
cg25787499	TMEM151A	2.58E-05	0.31929689
cg26196480	TBX5;TBX5	1.96E-05	0.319292975
cg00629427		7.23E-05	0.319288036
cg17109042	ANKS1B;ANKS1B;ANKS1B	2.58E-05	0.31928661
cg13782301	PRRT1	5.59E-05	0.319280892
cg17982102	RAB31	5.59E-05	0.31926488
cg07477009	UGGT2	1.50E-05	0.319264292

cg16789104	SKAP1;SKAP1	0.000357885	0.319254133
cg07341624	TMC2	1.96E-05	0.319250943
cg14738170	PCDHGA4;PCDHGA2;PCDHGB2;PCDHGA5;PC	0.000290372	0.319248726
cg06886153		0.000117986	0.319240344
cg26538140		0.001948248	0.319237236
cg02471897		0.000290372	0.319235874
cg22759823		0.000570759	0.319231238
cg27294678	TMEM45B	0.000290372	0.319194953
cg06219660	NADK	1.50E-05	0.319164365
cg24887140	PTGS2	7.23E-05	0.319158479
cg14081924	PAQR9	7.23E-05	0.31915716
cg03780545	LRRC67	0.000357885	0.31915003
cg00833352	NEBL	0.000187194	0.319141679
cg14633398		0.000791389	0.319128641
cg24882551		1.11E-05	0.319119287
cg16232504	SNX7;SNX7	0.000290372	0.319111964
cg12920180	COCH;COCH	0.000233422	0.319072578
cg26056703		8.60E-06	0.319072091
cg01468621	BRSK2	5.59E-05	0.319056542
cg25774643	SCT	0.003189589	0.319051343
cg18930354	ZIC4;ZIC4;ZIC4;ZIC4;ZIC4	8.60E-06	0.319029631
cg17466795	C20orf103	5.59E-05	0.319008065
cg09680447		1.50E-05	0.319003125
cg00172812		0.000187194	0.318997723
cg26416887	CUX2	0.001147425	0.318992709
cg27119456	LOC441666	0.000290372	0.318984632
cg25396728	IQSEC3;IQSEC3	0.000791389	0.318982936
cg18481230	KCNJ3	8.60E-06	0.318981743
cg17964432	WWC2;C4orf38	0.002304201	0.318975078
cg11165905	ATP8A2	0.002304201	0.318951689
cg01791587	SLC6A17	3.34E-05	0.318944167
cg06943667	DKK3;DKK3;DKK3	0.002304201	0.318931535
cg26118906		1.11E-05	0.318927461
cg01716975		0.000148457	0.318909952
cg11742688	ABCB10	0.000791389	0.318900631
cg16027761	PCDH8;PCDH8	0.000537905	0.318893486
cg12189835	SYT7	0.000290372	0.318883915
cg24594454		0.000654035	0.318882588
cg04071270		1.11E-05	0.318875503
cg11377047	MIR1976;RPS6KA1;RPS6KA1	0.001376172	0.318868483
cg02699218	ANKRD43	4.34E-05	0.318866547
cg21561970	ISM1	0.000187194	0.318861511
cg04219099	MANEAL;MANEAL;MANEAL	0.000357885	0.31884704
cg17082803	VAX2	0.000955793	0.318846502
cg06151243		1.50E-05	0.318830755
cg11412466		0.000955793	0.318824855
cg09461185	PTGS2	7.23E-05	0.318819291
cg10634551	KCNMB2;KCNMB2	0.005897668	0.318817409
cg13548361	PSD;FBXL15	1.96E-05	0.31881591
cg07989851		0.000365373	0.318809819
cg03171795		0.000187194	0.318803412
cg26870584	COL12A1;COL12A1	5.59E-05	0.318800117
cg15914828		7.23E-05	0.318789552
cg24347994		0.000233422	0.318783196
cg15196197	TNXB	0.001948248	0.318775818
cg26485937	PCDHGA4;PCDHGA9;PCDHGA1;PCDHGB1;PC	4.34E-05	0.318771937
cg18064025	EYA1	0.000357885	0.318759276
cg26123605		5.59E-05	0.31873068
cg26332310	BARHL2	0.000117986	0.318722559
cg19414591		9.24E-05	0.318722494

cg20217257	CCDC88B	2.58E-05	0.318714906
cg05655837	T	4.34E-05	0.318710649
cg08015447	A2BP1;A2BP1	7.23E-05	0.318702718
cg00249621	TSPYL5	0.002304201	0.318690241
cg20930114	SEPT10;SEPT10;ANKRD57	8.60E-06	0.318681147
cg11816600	KIF21A	0.001639597	0.318680393
cg23511285	GLTSCR1	3.34E-05	0.318671446
cg19273773	NAPEPLD;NAPEPLD	0.005897668	0.318655086
cg05341815		0.000357885	0.318645525
cg11831043	CREB5	0.004357816	0.318638932
cg23260190	ENTPD7;ENTPD7	0.000233422	0.318626919
cg16778107	AXIN1;AXIN1	0.000357885	0.318624385
cg09601912		4.34E-05	0.318609475
cg15962861		4.34E-05	0.318604313
cg02520104	NDFIP2;NDFIP2	0.000148457	0.318601408
cg24318763	CLEC2L	0.000290372	0.318594173
cg14797198	PHF21B;PHF21B	8.60E-06	0.318587531
cg27295373	SLC30A10	0.000290372	0.318582231
cg10679156		0.000654035	0.31857751
cg08919494	CA8	0.000955793	0.318572534
cg26590537	KCNA1	4.34E-05	0.318569119
cg24990317	NCOR2;NCOR2	9.24E-05	0.318568545
cg05186666	FLJ44817	0.000357885	0.318564183
cg03679521	NEUROD2;NEUROD2	5.59E-05	0.31854371
cg02543772	TMEM132C	7.23E-05	0.318539507
cg08141165	DAB1	2.58E-05	0.318539194
cg07903918	GABBR2	4.34E-05	0.318537104
cg04902302		0.002304201	0.318525768
cg09601175		1.96E-05	0.318525242
cg20499290		7.23E-05	0.318512896
cg06526620	FUT4	9.24E-05	0.31848891
cg08974450	ATP2C2;ATP2C2	0.001376172	0.318487967
cg14065115		0.000791389	0.318484685
cg07511564	NXPH1	0.000791389	0.3184835
cg10476773		0.001376172	0.318478548
cg04608900	VAX2	0.000791389	0.318476039
cg19542238	SEMA3E;SEMA3E	0.000233422	0.318457137
cg18468219	MARVELD3;MARVELD3	1.11E-05	0.318455904
cg01511828	CCBE1	0.000357885	0.318452896
cg06630799	TFAP2C	5.59E-05	0.318451464
cg25387636	SLC47A1	7.23E-05	0.318446189
cg16426459	MLPH;MLPH	8.60E-06	0.318443113
cg11624233	SIGIRR;SIGIRR	5.59E-05	0.318428148
cg03408135	CR1L	0.000537905	0.318419625
cg18298920		0.002304201	0.318417832
cg09638668	RFX4	0.000357885	0.318408039
cg20004910	CYP1A1	0.000233422	0.318406796
cg19223782	RNH1;RNH1;RNH1;RNH1;RNH1;RNH1;RNH1;R	4.34E-05	0.318398863
cg18649319		0.000357885	0.318388518
cg25273602	YTHDF2	0.001147425	0.318383653
cg27007937	PCDHB3	5.59E-05	0.318382481
cg23232188	EAF2	0.000117986	0.318357985
cg03717315	SEMA4F	0.000537905	0.318354564
cg12844784	FREM2	3.34E-05	0.318349326
cg27256528	A2BP1;A2BP1	1.96E-05	0.318348502
cg02154345		3.34E-05	0.318331526
cg25697556	WASF3	5.59E-05	0.318324887
cg26482052		2.58E-05	0.318322958
cg15618978	TRIM59	0.001376172	0.318321084
cg08592707	PPM1E	1.96E-05	0.318320803

cg14431361		0.000233422	0.318320007
cg02640041	RXFP3;RXFP3	0.000117986	0.318317499
cg05317275	COL5A3	0.000791389	0.318315122
cg15731815	RNF207	0.000654035	0.318303439
cg03943839	TMC7;TMC7	0.000117986	0.318299167
cg08881796	VPS53;VPS53	5.59E-05	0.318296807
cg04692403	TCF21;TCF21	4.34E-05	0.318296337
cg00532449		0.000537905	0.318294138
cg02772430	SLITRK1	3.34E-05	0.318288935
cg02370605	CDH7;CDH7	1.50E-05	0.318266063
cg26669793	PRRX1;PRRX1	0.002304201	0.31825704
cg18774195	SLC7A14;SLC7A14	2.58E-05	0.318240999
cg10074775	ZFHX4;LOC100192378	1.96E-05	0.318229319
cg04704053		0.000290372	0.318224763
cg01269718		0.00043997	0.318222006
cg14458619	PTPRD;PTPRD	0.002714607	0.318220211
cg06912966		0.001147425	0.31821149
cg01143309	FADS6	0.001147425	0.318207285
cg15254559		0.000148457	0.318186926
cg27061249	PHACTR2;PHACTR2;PHACTR2;PHACTR2	9.24E-05	0.318185004
cg08602500	PTPRN2;PTPRN2;PTPRN2	7.23E-05	0.318176266
cg05264870	NRP2;NRP2;NRP2;NRP2;NRP2;NRP2	0.000955793	0.318172389
cg21702506	SLC6A15;SLC6A15;SLC6A15	1.50E-05	0.318168686
cg01426713		7.23E-05	0.318161926
cg14576825		0.001376172	0.318161619
cg26290543		0.000117986	0.318157489
cg11222948	FAM83H	3.34E-05	0.318155531
cg23529896		0.000290372	0.318149452
cg13846270	DLEU7	0.001376172	0.318147108
cg08887694	IL17RB;CHDH;CHDH	0.000233422	0.318114622
cg01727145	SPEG	0.002304201	0.318110978
cg05921905	HOXA2	5.59E-05	0.318108905
cg26630735	CCDC68	0.000233422	0.318100231
cg12018969		7.23E-05	0.318100216
cg13806741	FAM181B;FAM181B	1.50E-05	0.318098831
cg00852924	TOR3A	0.000290372	0.318084123
cg24351167	PHACTR1	1.96E-05	0.318082106
cg16358679	DNAJA4;DNAJA4	0.0050758	0.318081647
cg09668400	SHISA2	4.34E-05	0.318045528
cg23586322	COL14A1	0.000262362	0.318034113
cg13342435	MPPED2	0.003189589	0.31801652
cg27512205	KITLG;KITLG	1.11E-05	0.318012718
cg21318213	NR2F2;NR2F2;NR2F2;MIR1469;NR2F2	9.24E-05	0.318011266
cg20353489		1.50E-05	0.318000717
cg09772121	CADPS2;CADPS2;CADPS2	0.000148457	0.317999827
cg10824063	NELL1;NELL1	0.001147425	0.317996113
cg25442600		0.005897668	0.317995888
cg11678896		0.000233422	0.317994404
cg10329173	PCDH7;PCDH7;PCDH7	8.60E-06	0.31799369
cg17460447	EVC;EVC	0.002714607	0.31798227
cg15384598	JPH4;JPH4	0.000233422	0.317970502
cg17530231		0.000148457	0.317940507
cg03286235	TACR1;TACR1	0.0050758	0.317913422
cg25618271		0.000357885	0.31791195
cg14171414	PCDH8;PCDH8	2.58E-05	0.317893762
cg18285309	HPCAL4	0.00043997	0.317887715
cg25965774	CTBP2;CTBP2	0.000290372	0.317877635
cg22082462	CPNE6	0.000117986	0.317869873
cg08939373	C11orf41	0.000537905	0.317865562
cg27344859		0.005897668	0.317864857

cg27492749	SGCE;SGCE;PEG10;SGCE;PEG10	7.23E-05	0.317860072
cg09710586	PPP2R2C	5.59E-05	0.317849195
cg20357910	PLA2R1;PLA2R1	0.000955793	0.317842594
cg20310894	HHIPL1;HHIPL1	0.000117986	0.317838238
cg19477977		0.000187194	0.317818148
cg00064284	YAP1;YAP1	0.001376172	0.317803936
cg02756106	NELL2;NELL2;NELL2;NELL2;NELL2	0.000955793	0.317801031
cg11264499	MTX3;MTX3	0.009066563	0.317800398
cg23496400	VSNL1	2.58E-05	0.317794381
cg26311734		0.000357885	0.317793144
cg09840840	CACNG4	4.34E-05	0.317790508
cg12959558		2.58E-05	0.317790125
cg22678136	SNRPN;SNRPN;SNRPN	1.96E-05	0.317775801
cg21667878	INS-IGF2;IGF2AS;IGF2;IGF2;IGF2AS;IGF2	0.002304201	0.317767358
cg10742917		5.59E-05	0.317766383
cg11092486		0.000233422	0.317763066
cg05281894	PDPN;PDPN;PDPN;PDPN	3.34E-05	0.31776152
cg17451176	PCDHGA4;PCDHGA2;PCDHGB2;PCDHGA5;PC	0.000148457	0.317753262
cg27316369		4.34E-05	0.317749375
cg02860732	SHISA9;SHISA9	0.000654035	0.317728639
cg03098159		9.24E-05	0.317725098
cg05184456	SCRT1	5.59E-05	0.317721841
cg11284797		0.000148457	0.317720958
cg13242070		4.34E-05	0.317712141
cg09698471	HTR1A	7.23E-05	0.317710117
cg13589463	WDR25;WDR25	0.000233422	0.317709058
cg26620157	PAX9	3.34E-05	0.31770245
cg06248560	BRUNOL4;BRUNOL4;BRUNOL4;BRUNOL4	0.000955793	0.317699344
cg18724565	FSCN1	8.60E-06	0.317697013
cg22029297	LIN28	1.11E-05	0.317690437
cg06353425	ROBO2	0.000148457	0.317652073
cg26919149		9.24E-05	0.31763415
cg23695504	C1orf229	0.000791389	0.317612812
cg16049600	PCDHB11	0.000290372	0.317612451
cg03663462		0.000233422	0.317607564
cg11920122	GNAL;GNAL;GNAL	4.34E-05	0.317606761
cg11859607	ADCYAP1;ADCYAP1	0.000233422	0.317596101
cg22787719		0.000357885	0.317589079
cg10108372		1.50E-05	0.317563084
cg03351460	GDF1;LASS1;LASS1	0.003189589	0.317561205
cg26140688	HUNK	0.001948248	0.317538407
cg05719140		3.34E-05	0.317534531
cg04049253	ZBTB16;ZBTB16	7.23E-05	0.31752697
cg06805348	KIAA1239	1.50E-05	0.317512694
cg18038894	ACSF2	0.000357885	0.317505023
cg15591678	ZNF365;ZNF365;ZNF365;ZNF365;ZNF365;ZNF	0.000148457	0.317500357
cg14367995		0.001291055	0.317494709
cg12627071		8.60E-06	0.317494649
cg23588217	TBX15	0.000233422	0.317475345
cg15699085		0.000955793	0.317474314
cg00499475	TBX3;TBX3	3.34E-05	0.317442744
cg24558425	CSMD2;C1orf94	8.60E-06	0.317441816
cg02981731	OGDHL;OGDHL;OGDHL	1.96E-05	0.317425651
cg11942936		0.000187194	0.317403873
cg02495823		8.60E-06	0.317403262
cg05406943	GRIN2A;GRIN2A;GRIN2A;GRIN2A	0.006823935	0.317401651
cg20813589	RNH1;RNH1;RNH1;RNH1;RNH1;RNH1;RNH1;R	0.007873393	0.317401225
cg14081270		0.001376172	0.317400837
cg22717227	C10orf53;C10orf53	1.96E-05	0.317398967
cg15672853		8.60E-06	0.317378177

cg13629999	UCK1;UCK1	0.000187194	0.317360949
cg24349886	PLAC2	4.34E-05	0.317353601
cg15829642	PCDHGA4;PCDHGA2;PCDHGA5;PCDHGB2;PC	2.58E-05	0.317347014
cg15325658	ROBO1	0.000117986	0.317310918
cg07691152		0.000233422	0.317287722
cg02663821	FAM46A	5.59E-05	0.31728718
cg21202716	SHANK2;SHANK2	9.24E-05	0.317269134
cg03947464	UPK3A;UPK3A	0.004357816	0.317263778
cg17242351	ACTC1	0.001639597	0.317259955
cg20274462		0.000290372	0.317253523
cg05528102	MAP1B	0.000537905	0.31725192
cg05390694	KIAA1024	0.000158823	0.317251588
cg01919632	FLRT2	7.23E-05	0.317249683
cg21773142	VSTM2A	1.11E-05	0.317248275
cg01869896	MFSD2A;MFSD2A	0.000117986	0.317222161
cg08793792		3.34E-05	0.317220426
cg16095551		1.96E-05	0.317218669
cg13327545		1.11E-05	0.317211131
cg23303074	LRAT	9.24E-05	0.317210833
cg12078155		1.96E-05	0.317204688
cg18003762	SCRN1;SCRN1;SCRN1;SCRN1;SCRN1;SCRN1	0.003189589	0.317190497
cg08276295	GABRB3;GABRB3	0.000357885	0.317181089
cg20779917		3.34E-05	0.317172338
cg00717482	KCND3;KCND3	0.000187194	0.31717059
cg07406191	ZIC4;ZIC4;ZIC4;ZIC4;ZIC4	7.23E-05	0.31716412
cg00883831		0.002714607	0.317162912
cg23942980	TPBG;TPBG	0.002304201	0.317162281
cg05890377		0.009066563	0.317156513
cg22666828	BRUNOL4;BRUNOL4;BRUNOL4;BRUNOL4	0.000148457	0.317145124
cg25894334	FAM47E	0.000955793	0.317143912
cg13209481	SFTA3	0.000570759	0.317143521
cg02627227	AGPAT4	7.23E-05	0.317140792
cg14514032	LBX2	0.000117986	0.317137885
cg20773479	CEP72	3.34E-05	0.317117828
cg21727532	CLVS2	4.34E-05	0.31711451
cg25638611	SHANK2;SHANK2	0.000955793	0.31710985
cg11166303	TSSC1	4.34E-05	0.317095717
cg00617305	SHISA4;SHISA4;SHISA4	0.00043997	0.317089847
cg05685587	FOXQ1	8.60E-06	0.317075007
cg12236164		1.96E-05	0.317067797
cg26309194	TMPRSS2;TMPRSS2	0.001948248	0.317064192
cg11773720	ADCYAP1;ADCYAP1	4.34E-05	0.31703398
cg18043828	C9orf150	0.003189589	0.317005281
cg16002507	KIF1A	4.34E-05	0.317000578
cg05476568	SGEF	0.000357885	0.316992905
cg27200630		0.002714607	0.316988151
cg09306675	EYA2;EYA2	7.23E-05	0.316982417
cg16545105	CRHBP;CRHBP	7.23E-05	0.316967993
cg23262134	OVOL2	0.000148457	0.316945654
cg22723675		0.003189589	0.316938208
cg02802029	PLOD2;PLOD2	1.11E-05	0.316936814
cg18267506	SPATA6	0.001639597	0.316913932
cg25714392	C3orf59	0.0050758	0.316908721
cg14677681	KCTD8	0.000537905	0.316887041
cg14597545	ADPGK;ADPGK;ADPGK	9.24E-05	0.316886631
cg13640297	WDR27	7.23E-05	0.316874262
cg06846752	ZBTB8B	1.96E-05	0.316872423
cg04350202	XPNPEP1;XPNPEP1;XPNPEP1	7.23E-05	0.316871649
cg14192291	BARHL1	2.58E-05	0.316857517
cg03369269	FAM123A;FAM123A	0.000187194	0.316852882

cg24032249		9.24E-05	0.31684929
cg09632976		3.34E-05	0.31684809
cg03349184		7.23E-05	0.316838863
cg26002512	KCND2;KCND2	0.000148457	0.316827739
cg18544329	DLX5	0.001147425	0.31682338
cg00788461	SPATA6;SPATA6	0.003189589	0.316821197
cg08360511	SYT7	1.11E-05	0.316818505
cg00441573		0.000955793	0.31681575
cg17243193		0.000791389	0.316810814
cg04694812	HTR1A	1.50E-05	0.316778471
cg17774851	NR2F1	0.000357885	0.316777162
cg07260592		1.11E-05	0.316735326
cg16904960		0.000187194	0.316730588
cg08011119	C1QL2	2.58E-05	0.316717611
cg18338775		0.000117986	0.316715339
cg21883598	DUOX2	0.000654035	0.316704037
cg16458596		8.60E-06	0.316696575
cg10825876	PLA2R1;PLA2R1	0.000654035	0.31669328
cg13702996	IQSEC3	4.34E-05	0.316679027
cg17211404	PLA2G7;PLA2G7;PLA2G7	5.59E-05	0.316675246
cg11907797	PCDHGA4;PCDHGA7;PCDHGA6;PCDHGA1;PC	0.000117986	0.316674769
cg14571669	MSRB3;MSRB3	1.96E-05	0.316665016
cg03287940	THRB;THRB;THRB	0.003189589	0.316662503
cg26298967		2.58E-05	0.316655738
cg03370752	TMEM138;TMEM138	3.34E-05	0.316655287
cg03258472	CRB3;CRB3	2.58E-05	0.31665464
cg06567290	SLC37A4;SLC37A4;SLC37A4;SLC37A4;SLC37A	0.001948248	0.316652483
cg09960171	KCNB2	0.000148457	0.316647476
cg23602533	TTPA	1.11E-05	0.316646778
cg21727359	FAM46A	0.002304201	0.316644897
cg17086773		0.000117986	0.31664264
cg01099876	SEPT10;ANKRD57;SEPT10	0.000117986	0.316628709
cg10386016	RELN;RELN	5.59E-05	0.316625987
cg16134686	PCSK2	0.000187194	0.316625819
cg00840990		0.002714607	0.316606305
cg13372456	GALNT10	0.002927025	0.316595895
cg25060172	CDKL2	0.000955793	0.316594822
cg24259244	ZIC5	1.50E-05	0.316583639
cg14848594	SLC30A10	3.34E-05	0.316580573
cg13398291	SFRP1	0.000117986	0.316578389
cg10236239	SULT1C4;SULT1C4	0.000117986	0.31657837
cg04457196	FIGN	0.00043997	0.316574574
cg13891768		0.000290372	0.316573738
cg25146557	LOC200726	1.11E-05	0.316565969
cg14342707		5.59E-05	0.316555421
cg17124224	BNC1	0.000357885	0.316544336
cg21742923	KIF5C	0.000158823	0.316525875
cg21439672	C1RL;LOC283314	0.002304201	0.316517877
cg08332990	IDUA	1.96E-05	0.316504407
cg10297473	SALL1;SALL1	0.000357885	0.31650395
cg26315263	MYO1E	0.000791389	0.31649791
cg18107425	MESP2	1.96E-05	0.316495449
cg20348858	EMX2;EMX2;EMX2OS	8.60E-06	0.316493347
cg08844701		1.11E-05	0.316491592
cg10885338	C2orf40	1.96E-05	0.316491327
cg13928709	UNC5A	0.002304201	0.316481737
cg15515258		0.000357885	0.316470146
cg13851843	ABCC8;ABCC8	4.34E-05	0.316449751
cg05919238	SLC38A10;SLC38A10	3.34E-05	0.316445099
cg22540115	CYP26B1	0.000148457	0.316438428

cg02808240	C6orf10	9.24E-05	0.316437659
cg03843972		2.58E-05	0.316427398
cg02357541		2.58E-05	0.316410767
cg23625388	RIN3	0.002714607	0.316403894
cg11846112		0.000187194	0.316403403
cg04032066	SLC6A5	2.58E-05	0.316402852
cg04835811		8.60E-06	0.316399323
cg13534734		2.58E-05	0.316366651
cg01394819	PODN	0.000187194	0.316363901
cg04147027	FAM46A	0.000791389	0.316357758
cg05367661	C7orf57;C7orf57	0.001147425	0.316334645
cg02920218	SMPDL3A	2.58E-05	0.316333189
cg06508879	IGSF9B	0.000187194	0.316328509
cg23060047		0.000233422	0.316322157
cg14045872	VWC2	0.002304201	0.316319402
cg14596967	ZBTB16;ZBTB16	0.000187194	0.316317628
cg06680243		1.96E-05	0.316308887
cg18153869	MAGOH	0.001147425	0.316308747
cg00338702	CHFR;CHFR;CHFR;CHFR;CHFR	0.00043997	0.316308218
cg11178293	FEZF2	0.004357816	0.316286015
cg18798922	SP5	0.000187194	0.31626942
cg17883458		0.000117986	0.316261702
cg09407232		2.58E-05	0.316249469
cg01719995	USP38	9.24E-05	0.316237181
cg18702689	COL25A1;COL25A1	0.000357885	0.316233119
cg20548888	SFTA3	0.000148457	0.316222587
cg12189137	FBXO21;FBXO21	0.000187194	0.3162207
cg22585595	LOC401463	4.34E-05	0.316218363
cg12418190		1.96E-05	0.316214742
cg20537640		1.96E-05	0.316214561
cg11088672		0.000654035	0.316212542
cg14583999	TMEM111	0.000233422	0.316207937
cg22369787		1.96E-05	0.316202454
cg01451391	HLF	0.001147425	0.316197041
cg02593884	FLYWCH1;FLYWCH1	0.000187194	0.316193176
cg17901584	DHCR24	0.000357885	0.316165554
cg05928053		1.96E-05	0.316162193
cg15681626		0.000357885	0.316150071
cg04275490	GSX2	4.34E-05	0.316142876
cg13561409	PIP5K1C	9.24E-05	0.31613143
cg09557462	ADRA1A;ADRA1A;ADRA1A;ADRA1A	0.000233422	0.316124477
cg06999323	NEFL	0.000955793	0.316111119
cg13132370		0.000791389	0.316095493
cg18988498	PAX6;PAX6;PAX6	0.000233422	0.316095201
cg09447621	B3GAT1;B3GAT1	0.006823935	0.316090897
cg25731807	TMEM171;TMEM171	8.60E-06	0.316087206
cg18235937	RNF207	0.000537905	0.316075283
cg01332882	ZNFX1	1.96E-05	0.316065633
cg08939394		0.000654035	0.316033252
cg26365998	CCDC60	0.000187194	0.316007471
cg19392831	PRLHR	1.11E-05	0.316003406
cg05892674	C11orf70;C11orf70	4.34E-05	0.315996734
cg14163665	ALK	7.23E-05	0.315960482
cg05516537	TFDP1;TFDP1	7.23E-05	0.315945254
cg12437013	TMCO3	7.23E-05	0.315941046
cg27341128	DOK5	0.000570759	0.315936088
cg25565383	RAD51AP2	2.58E-05	0.315922825
cg01874697	SORCS3	3.34E-05	0.315912079
cg19623418		0.000187194	0.315907938
cg04987053	SEC14L5	0.000117986	0.315901314

cg25574175	NR1I2;NR1I2;NR1I2;NR1I2;NR1I2	5.59E-05	0.315894755
cg05969591	C2orf88;C2orf88;C2orf88;C2orf88	3.34E-05	0.315894136
cg12918473	WNT2;WNT2	0.000233422	0.315891733
cg26577169	LOC441177;C6orf176;C6orf176	0.000117986	0.315888253
cg00382138	CFI;CFI	0.000955793	0.31587074
cg05406635	CCDC85C	5.59E-05	0.315858936
cg25139649	SKI	0.002714607	0.315851402
cg02145932	ISM1	0.000233422	0.315846866
cg09681877		9.24E-05	0.315837401
cg27076139	ADCYAP1R1	0.000117986	0.315808977
cg02221471		8.60E-06	0.315803141
cg14374829	HUNK	0.000148457	0.315801729
cg04011995		9.24E-05	0.315796348
cg23702046	TSPAN9;TSPAN9	2.58E-05	0.315791193
cg09293603		3.34E-05	0.315767413
cg11457640		7.23E-05	0.315764734
cg02331561	ABCA17P;ABCA3	2.58E-05	0.31576023
cg25930591	DLX6AS	0.000791389	0.315758579
cg20879085	TES	0.006823935	0.315755309
cg21796225		7.23E-05	0.315740108
cg00157477	HLA-H	0.000187194	0.315726828
cg10480584	PRDM6	3.34E-05	0.315719451
cg00858840	SP5	5.59E-05	0.315712357
cg11214140	SOX17	0.00043997	0.315709608
cg02788102	CADM1;CADM1	0.000187194	0.315704456
cg03741619	TRPV3	1.50E-05	0.315699611
cg17775765		3.34E-05	0.315695272
cg04384626		0.000117986	0.315687965
cg02348449	ZSCAN18;ZSCAN18	0.000148457	0.315686124
cg02481642	WISP2	0.009066563	0.31567978
cg04105342	PCDH7;PCDH7;PCDH7	8.60E-06	0.3156645
cg26816688	C7orf13;RNF32	0.006823935	0.315658223
cg00525277	TNXB	0.000537905	0.315644449
cg08303612	SLC22A3	0.000117986	0.315641711
cg00852622	KCND3;KCND3;KCND3;KCND3	0.002714607	0.315634336
cg21178978	TRIM67	5.59E-05	0.3156295
cg02666610		0.005897668	0.315626991
cg18500988		1.96E-05	0.315620549
cg25244722		4.34E-05	0.315605775
cg11362013		0.000290372	0.315604806
cg09241332	ZIC1	1.50E-05	0.315603077
cg04539203	H2AFY2	0.000654035	0.315603054
cg05121006		3.34E-05	0.315594658
cg06633438	MLLT1	0.001948248	0.315549521
cg14375531	C9orf170	0.003189589	0.315537613
cg04845060		0.000117986	0.315528829
cg22428147	DGKI	0.000117986	0.315465876
cg12689285	GDAP1L1	0.000148457	0.315435025
cg06169746		7.23E-05	0.315421266
cg05499367	TMEM26	5.59E-05	0.315404106
cg11856078		0.004357816	0.315399715
cg10536067	FADS6	4.34E-05	0.315398247
cg14008883	SLC18A3;CHAT	8.60E-06	0.315391586
cg27485921	ATP6V1E2	0.000537905	0.315390934
cg09965733	LRFN5	4.34E-05	0.315370991
cg23165899	ID4	0.000955793	0.315365077
cg24786875	ANKRD32	0.000955793	0.315342893
cg00181439	HTR1B	2.58E-05	0.315336544
cg20804831	NUDT16P;NUDT16P	1.11E-05	0.315318781
cg11072882	FAM47E	9.24E-05	0.315312477

cg17810609		0.001376172	0.315287275
cg23500537		1.96E-05	0.315286434
cg03440762		0.000791389	0.315276614
cg26530498	C1QL2;C1QL2	9.24E-05	0.315272947
cg03445516		4.34E-05	0.31526671
cg15501219		0.000233422	0.315259002
cg11971771		0.000233422	0.315251594
cg08014499	IGF2AS;INS-IGF2;IGF2;IGF2;IGF2AS	0.000654035	0.315236493
cg11241627	FERD3L	1.50E-05	0.315231512
cg24310133	SLC37A3;SLC37A3	4.34E-05	0.315222813
cg15848986	LOC100190940	9.24E-05	0.31521742
cg26895804		7.23E-05	0.315212236
cg22886275		1.96E-05	0.315185613
cg14336011	LHX3	0.000290372	0.315185601
cg27564792	FEZF2	0.000187194	0.315172811
cg21159128	SSBP3;SSBP3;SSBP3	0.001639597	0.315169783
cg14148055		0.000187194	0.315167452
cg24570042	GLDN	2.58E-05	0.315162402
cg06836736	ME1	3.34E-05	0.315141507
cg09305883		3.34E-05	0.315139317
cg19723805		0.000955793	0.315130981
cg24837149	CCNL2;CCNL2;CCNL2;LOC148413;CCNL2	0.000791389	0.315128682
cg06570358	ARHGAP10	0.000117986	0.315128474
cg26115531		1.50E-05	0.31511541
cg27038197	LCA5;LCA5	0.000537905	0.315103902
cg04259977	HCN1	5.59E-05	0.315097734
cg03352181	HSPA12A	0.000955793	0.315084057
cg07480647	GPR153	0.001376172	0.315079867
cg01577751	HOXC13	9.24E-05	0.315077205
cg16276982		0.000654035	0.315076313
cg05084391	LOC283856;GNAO1;GNAO1;GNAO1;GNAO1	0.002714607	0.315075617
cg16390060	NKX2-2	0.000290372	0.315065801
cg00259404	TRIM27	0.001639597	0.31506266
cg03415617		3.34E-05	0.315041438
cg23906459		9.24E-05	0.315039622
cg24669528	C13orf36	9.24E-05	0.315027174
cg24094706	FAM19A5;FAM19A5	0.003189589	0.315021451
cg16863829	DUPD1	9.24E-05	0.315010461
cg27282530	ALDH1L1	9.24E-05	0.315004136
cg13085627	LRRK1	3.34E-05	0.315003061
cg25516803	CCK	5.59E-05	0.314984732
cg14202146	FBXL15	0.000955793	0.314979867
cg23952644	WASF3	0.003189589	0.31496244
cg04742719	SLC12A9;TRIP6	0.001147425	0.314958803
cg00129196	WDR35;WDR35	0.000148457	0.314958301
cg00680572	NKX2-8	3.34E-05	0.314957442
cg21349625		3.34E-05	0.314940945
cg11741189		0.000187194	0.314935652
cg05108031		0.000357885	0.314920999
cg02379107	KIAA1755	0.000357885	0.314912437
cg10023530	FAM38B	7.23E-05	0.314890722
cg05211662	ADAMTS19	1.96E-05	0.314872824
cg09837656	PLEKHO2	0.000290372	0.314870486
cg24113409		0.000537905	0.314867261
cg10588034	ESYT3	0.001291055	0.314866247
cg15305538		0.000396647	0.314862046
cg21239901		5.59E-05	0.314861694
cg03746482		1.11E-05	0.314854692
cg05144889	GPR148	0.000187194	0.314854342
cg14394550	EGR3	0.006823935	0.31484151

cg15034135	ADAMTS19	4.34E-05	0.3148334
cg19985870		0.000233422	0.314828197
cg27639457	CTNND2	8.60E-06	0.314819399
cg16922039		9.24E-05	0.314814967
cg02303941		1.96E-05	0.314810456
cg04552451		0.000791389	0.314808355
cg12847240		1.11E-05	0.314805458
cg06499368		0.000654035	0.314805286
cg26931990	IFT140	0.000654035	0.31479902
cg02229095	ZNF34	9.24E-05	0.314798843
cg14859460	GRM6	1.96E-05	0.314793354
cg14118850	HPCAL1;HPCAL1	0.001376172	0.314789512
cg22491058	C4BPA	0.000654035	0.314786841
cg24658517		2.58E-05	0.314780001
cg27204739	ANKRD5;ANKRD5;ANKRD5;ANKRD5	0.004357816	0.31477833
cg18120259	LOC100132354	9.24E-05	0.314763243
cg04223420	PITX1	0.001376172	0.314755484
cg04422150		0.000233422	0.31474337
cg24511738		0.000654035	0.314742471
cg10168722		4.34E-05	0.314738017
cg23884076	CXCL10;ART3	0.000290372	0.314733257
cg04771100	HTRA4	0.005897668	0.314720197
cg06861044	UMOD;UMOD	0.00043997	0.314702914
cg07964848	MKX	2.58E-05	0.314700059
cg27638196	CDK5R2;CDK5R2	0.000357885	0.314693276
cg03452895	GCA	0.000791389	0.314692472
cg08073591	FAM47E	0.002040485	0.314690096
cg14753599	C10orf116	3.34E-05	0.314683331
cg05481929	POLR2C	0.000187194	0.314670319
cg02120658	PAX3;PAX3;PAX3;PAX3;PAX3;PAX3;PAX3;PAX	4.34E-05	0.314629138
cg05890484	BHMT	0.000654035	0.314613364
cg14373456	PDGFRA	0.000187194	0.314602341
cg21505925	DYRK1A;DYRK1A;DYRK1A;DYRK1A;DYRK1A	0.000791389	0.314589916
cg25771271		0.000117986	0.314587287
cg13210260	DMRTA2	2.58E-05	0.314586412
cg10184740	TMEM196	1.11E-05	0.314584741
cg10996058	C1QL2	4.34E-05	0.314549729
cg15408073	RXFP3	0.000955793	0.314549674
cg12011136	PAPOLB;RADIL	1.11E-05	0.314544912
cg07673740		0.001376172	0.314539439
cg03626734	PRSS16	1.96E-05	0.314524267
cg22734397	NEUROG1	0.000233422	0.314515759
cg00383598	HSPBP1;HSPBP1	1.96E-05	0.314495159
cg03600687	BOK	0.000290372	0.314492694
cg23401251	IFT172	1.50E-05	0.31448112
cg05424071	GLRA3;GLRA3;GLRA3;GLRA3	7.23E-05	0.314463507
cg27039898		3.34E-05	0.314462919
cg03192020	TDH	1.50E-05	0.314455439
cg06861880	CYP1B1	0.000537905	0.31444974
cg11946503	NEUROG1;NEUROG1	0.001376172	0.31444127
cg11187796	ADRA2C	0.004357816	0.314440941
cg11141380	BSX	0.000791389	0.314436086
cg26770475	ZNF498	5.59E-05	0.31443464
cg18556976		0.001147425	0.314430226
cg09631043	TACC2;TACC2	1.11E-05	0.314422182
cg07593390	IL20RB	0.000290372	0.314415096
cg20314038	CSMD2	9.24E-05	0.314401581
cg16987754		0.000290372	0.314392931
cg14166009	HKR1	0.000117986	0.314390293
cg04121624	RHOBTB1;RHOBTB1	4.34E-05	0.314378099

cg07647822	THRB;THRB;THRB	0.000357885	0.314366005
cg02017257		8.60E-06	0.314343412
cg03186975		1.50E-05	0.314340974
cg18168663	REC8;REC8	1.96E-05	0.314334828
cg18414021	CCDC85C	0.000187194	0.314334691
cg03877267		5.59E-05	0.314319283
cg26531700		7.23E-05	0.314315339
cg24408656		9.24E-05	0.314298954
cg10724086	FOXA1	0.000233422	0.314293471
cg01285926		2.80E-05	0.314267404
cg05533124	AQP4;AQP4	4.34E-05	0.314260164
cg13936863	VAX2	0.000148457	0.314247591
cg08310116	NEK9	0.001948248	0.314228648
cg19620724	TFAP2B	4.34E-05	0.314218547
cg11043002	COL23A1	0.000357885	0.314210526
cg19511045	SPR	0.000791389	0.314208114
cg27215033		3.34E-05	0.314205429
cg02860602		0.000357885	0.314203075
cg22043588	USH1C;USH1C	4.34E-05	0.314188001
cg22669136		8.60E-06	0.314179647
cg03388409	TP53BP2;TP53BP2	0.004357816	0.314172273
cg07690882		0.000187194	0.314166705
cg18315960		0.000537905	0.314147728
cg00255821	CYP4X1;CYP4X1	0.00043997	0.31413558
cg09984545	SAMD5	7.23E-05	0.31411203
cg08761102	CYP1B1	0.003189589	0.314097426
cg23928824	GALNTL1;GALNTL1	0.000526175	0.314084678
cg21781828	MTX3;MTX3	0.006823935	0.314076256
cg18902742	FEZF2	0.000654035	0.314072454
cg01775260	GABRB3;GABRB3	0.000187194	0.314052974
cg19422030	NMU	1.96E-05	0.314032232
cg19672997	MDGA2	0.005897668	0.314029544
cg17165841	LOC200726	2.58E-05	0.31402525
cg24126698	RASA3	0.000233422	0.314024533
cg05355757		0.001147425	0.314023229
cg07397399	MAGI1;MAGI1;MAGI1	1.50E-05	0.31401863
cg23690528	SGCE;SGCE;PEG10;SGCE;PEG10	0.000187194	0.314009616
cg23429903	EPHA3;EPHA3	0.000791389	0.314002126
cg07067171	FKBP9	0.002714607	0.313993002
cg15846434		5.59E-05	0.313990831
cg27136844		0.000187194	0.313987701
cg06705017		0.000791389	0.313971163
cg08863459	CYP26B1	0.000357885	0.313965164
cg07483989	PRSS3	4.34E-05	0.313952907
cg00870662	PARD6G	0.000955793	0.313946147
cg25513776	MYLK4	0.00043997	0.313929056
cg24117150	KCNK10;KCNK10	0.0050758	0.313920982
cg26394220	MIR375;CCDC108	4.34E-05	0.313917722
cg02891686	SOD3	2.58E-05	0.313910835
cg24376434	PRRX1;PRRX1	7.23E-05	0.313906363
cg26292150	ZDHHC19	0.000791389	0.313905492
cg22259797	C2CD2L	7.23E-05	0.313898952
cg07657465	ZNF208	3.34E-05	0.313891717
cg00156336	FAM176A;FAM176A	0.000955793	0.313890168
cg04780572		0.003734675	0.313887003
cg06459443	TMEM132E;C17orf102	1.96E-05	0.31388522
cg08671267		4.34E-05	0.313879892
cg17923377	BRD1;LOC90834	5.59E-05	0.313844119
cg07377178		0.000537905	0.313837979
cg14993283	SIM1	7.23E-05	0.313837913

cg19295314	GMDS	0.000187194	0.313822066
cg03681335	SULT1C2;SULT1C2	7.23E-05	0.313805726
cg17485681	CDH23	4.34E-05	0.313773099
cg08685096	GRIK1;GRIK1	1.96E-05	0.313770225
cg01143068	STARD9	0.001376172	0.313761206
cg12608145	PCDHGA2;PCDHGA3;PCDHGA1;PCDHGA3	0.000357885	0.313755868
cg11510586		0.009066563	0.313747209
cg00650176	C9orf135	3.34E-05	0.313742958
cg03478969	ANKRD34B	2.58E-05	0.313741275
cg04094548		7.23E-05	0.313721581
cg03355526	ZNF454	1.50E-05	0.31370941
cg01755562	CLSTN2	9.24E-05	0.31370004
cg23576807		8.60E-06	0.313697371
cg01577475	PAX8;PAX8;PAX8;PAX8;PAX8;LOC440839	0.00043997	0.313693797
cg07753583	LRRC61;ACTR3C;LRRC61	3.34E-05	0.313681325
cg12227907	PRDM13	8.60E-06	0.313680925
cg11200929	CRABP1;CRABP1	0.000955793	0.313677997
cg13109216		9.24E-05	0.313672421
cg01337391		0.001147425	0.313668907
cg23384093		2.58E-05	0.313663662
cg24319143	DUSP22	1.96E-05	0.313662975
cg01839657	HOXC13	1.11E-05	0.313646397
cg11751707	CYP1B1	7.23E-05	0.313645688
cg27379109		0.000187194	0.313641694
cg04744409		5.59E-05	0.313639761
cg18447876		4.34E-05	0.313628224
cg02797930	SGEF;SGEF	3.34E-05	0.313606012
cg19460836	BAIAP2;BAIAP2;BAIAP2;BAIAP2	0.000955793	0.313601435
cg18313182	PNPLA2;PNPLA2	7.23E-05	0.313580991
cg13461118	VAPA;VAPA	0.003734675	0.313580617
cg23374964	VAX1;VAX1	0.000233422	0.313580059
cg26667946	SIAH3	0.005897668	0.31357725
cg16072304		0.00043997	0.313568681
cg11763094	ADAMTSL3	8.60E-06	0.313567656
cg23023466	KCNK1	0.000233422	0.313565452
cg07589612	GDF6	3.34E-05	0.313545193
cg20317748	C15orf52	0.009066563	0.313541352
cg12130328	LPHN2	0.000117986	0.313536092
cg17131030	TSPAN2	3.34E-05	0.31351372
cg03349922	H2AFY2	0.0050758	0.313488088
cg20769926		3.34E-05	0.313484778
cg00548098		2.58E-05	0.313479795
cg18980148	ST3GAL1;ST3GAL1	0.000955793	0.313473007
cg22571530	NFASC;NFASC;NFASC;NFASC;NFASC	3.34E-05	0.313470579
cg17039645	C6orf10	0.001948248	0.313468043
cg20634514	ERI3	0.000117986	0.313461844
cg06113354		0.000233422	0.313449352
cg17602126	HEYL	0.004357816	0.313448961
cg17386093	SLIT3	0.000357885	0.313443167
cg21012455	WDR8	8.60E-06	0.313418951
cg14669524	SPATS1	0.000537905	0.31341873
cg18603028	SPOCK1	0.000117986	0.313418626
cg02614660	CDK5R2;CDK5R2	7.23E-05	0.313408227
cg17683110		0.000791389	0.313403921
cg18469270		0.00043997	0.313396371
cg27079680	LOC134466	8.60E-06	0.313387712
cg00584000	TCHH	0.0050758	0.313386889
cg06583576		5.59E-05	0.313375012
cg05740567	SNORA22;CCT6P1	1.96E-05	0.31336859
cg02488942		0.000117986	0.313367884

cg04425632	DGKI	0.00043997	0.313358781
cg16326674	QRFPR;QRFPR	0.000290372	0.313352868
cg26548834	FOXK1	0.003189589	0.313349866
cg05100419		1.96E-05	0.313345035
cg25637894		4.34E-05	0.313334508
cg05495011		0.001639597	0.313318384
cg13184736	GNG12	0.0050758	0.313289331
cg05583681	HSDL1;LRRC50;HSDL1	7.23E-05	0.313287255
cg03048314	NBL1;NBL1	1.50E-05	0.313275632
cg26309457		1.96E-05	0.313270091
cg02676375	TLX3	4.34E-05	0.313267122
cg21187669	ATPAF2	0.000290372	0.313262958
cg21401647	HIST3H2A;HIST3H2BB	1.11E-05	0.313260719
cg09659661	IRX6	5.59E-05	0.313250002
cg02989669	GUCY1A2	0.000357885	0.313246194
cg04938315	TCHH	3.34E-05	0.313215198
cg09239744	GRIN2A;GRIN2A;GRIN2A;GRIN2A	0.001376172	0.313214941
cg14522803	PNPLA2	4.34E-05	0.313199233
cg07624600	ART5;ART5	0.000117986	0.313195869
cg09667001		4.34E-05	0.313190136
cg16691033	PPAP2C;PPAP2C;PPAP2C	4.34E-05	0.313179822
cg22944499	LOC200726;LOC200726	0.000117986	0.31317789
cg04016086	COBL	0.000537905	0.31317449
cg04024799	LOC646627	0.000117986	0.313161621
cg27362525	ZNF232	0.001639597	0.313102418
cg10823322	CRYGD	0.001639597	0.313094343
cg23747904	PCDP1	2.58E-05	0.313090339
cg06660332		2.58E-05	0.313073688
cg05011258	LRRC36;KCTD19	0.004357816	0.313063987
cg08883066	SYT6	4.34E-05	0.313061716
cg07105596	GNAS;GNAS;GNAS	1.50E-05	0.313049706
cg01436998		9.24E-05	0.313020392
cg18898440	WSCD1	0.000537905	0.3130138
cg19029904		0.000357885	0.31301123
cg19572637		0.000187194	0.312998624
cg07369190	PCP4L1	0.000791389	0.312996261
cg10608596	ZNF833	0.000955793	0.312992917
cg26644059	CRB3;CRB3	8.60E-06	0.312981358
cg22370006	OPRM1;OPRM1;OPRM1;OPRM1;OPRM1;OPRI	2.58E-05	0.312955372
cg12253819	GNAL	0.001147425	0.312953711
cg09622982	LYPD1;LYPD1	4.34E-05	0.312948354
cg04843813	FNDC3B	0.00043997	0.312931688
cg22058708		0.00043997	0.312916545
cg13223682		0.000117986	0.312901352
cg20998319		5.59E-05	0.312901068
cg25046074	EFEMP1;EFEMP1;EFEMP1	3.34E-05	0.312897555
cg24903183	SNX31	0.000537905	0.31288633
cg12630714		4.34E-05	0.312885214
cg04222728	KIAA1549;KIAA1549	0.000187194	0.312854885
cg12101586	CYP1A1	4.34E-05	0.312852308
cg07037251	SHISA6	0.000187194	0.312850451
cg08383399	CSMD1	0.000537905	0.312828913
cg01610857	TFAP2B	2.80E-05	0.312827929
cg23790942		8.60E-06	0.312815175
cg01485117	TNXB	0.00043997	0.312809778
cg03537386	COL16A1	4.34E-05	0.31279429
cg14774364	HTR1E;HTR1E	0.000654035	0.312779237
cg03181582	ABCC8	0.003189589	0.312772592
cg05494069	PRKAA2	1.50E-05	0.312764055
cg11670211	NOX4;NOX4;NOX4;NOX4	1.96E-05	0.312762513

cg19865133	MLF1;MLF1;MLF1	0.00043997	0.312759813
cg16005494	WBSCR17	0.000148457	0.312738247
cg16430072	KCNN2	0.000290372	0.312738127
cg03098643	FAT1	0.004357816	0.312729516
cg22221870	FAM3B;FAM3B;FAM3B;FAM3B	0.001639597	0.312721127
cg01908395		7.23E-05	0.312720198
cg25573227		0.001639597	0.312665354
cg09012125	KCNJ5	0.001948248	0.312665062
cg27254852	LOC100306951;INPP5K;INPP5K;INPP5K	0.000791389	0.312653745
cg10635895	CACNA1C;CACNA1C;CACNA1C;CACNA1C;CA	0.000148457	0.312653548
cg06067842	TMEM26	1.11E-05	0.312638059
cg11219917	CPLX1	0.000791389	0.312636898
cg20113732	NELL1;NELL1;NELL1;NELL1	0.001639597	0.312624775
cg14239346	WDR93;PEX11A;PEX11A	0.000357885	0.312601658
cg20076442		0.000117986	0.31258348
cg13124863	SCARA5;SCARA5	0.001376172	0.312568221
cg27103296		5.59E-05	0.312559448
cg18612627		0.000290372	0.312557976
cg05657618	NGEF;NGEF;NGEF	0.003734675	0.312542972
cg13364903	SCUBE3	0.000117986	0.312541219
cg21228005	ZNF300	0.002714607	0.312523246
cg07492240	C4orf31	1.50E-05	0.312516117
cg26450866	KCNA7	7.23E-05	0.312515101
cg18734433	ZNF775	7.23E-05	0.312492417
cg15092168	HTR1A	7.23E-05	0.312473735
cg16158681	MT3	0.000117986	0.312465463
cg10213542	ADAMTS2;ADAMTS2	0.000290372	0.312457803
cg11699925	KLF5	0.000187194	0.312446017
cg12980128	ONECUT2	0.001948248	0.312435774
cg17284779	CHAT;SLC18A3	1.96E-05	0.312428072
cg18755296	NPBWR1	0.00043997	0.312409432
cg00584422	ERN2;ERN2	0.000233422	0.312401675
cg20432350	KCND3;KCND3	8.60E-06	0.312398569
cg25007283	ZIC4;ZIC4;ZIC4	0.000357885	0.312390247
cg18443571	ZNF710	0.000187194	0.312384421
cg10037494	INS-IGF2;IGF2AS;IGF2;IGF2;IGF2AS;IGF2	0.00043997	0.312374099
cg27254482	OLIG2	0.001147425	0.312369959
cg03600318	SFTPD	0.000290372	0.312368053
cg20801753		2.58E-05	0.312354686
cg19714132	FOXG1	3.34E-05	0.312352319
cg05087842	C8orf47;C8orf47	1.96E-05	0.312335993
cg02007844	KCNJ8	9.24E-05	0.312334157
cg25228737		9.24E-05	0.312330403
cg23357250	DNAH9	1.50E-05	0.312315901
cg16546442	PITX2;PITX2;PITX2	8.60E-06	0.312296943
cg19592637	VSX1;VSX1	8.60E-06	0.312283487
cg07709606	RAD51AP2	0.000290372	0.312271091
cg05151185	SLC35C1;SLC35C1;SLC35C1;SLC35C1	2.58E-05	0.312265204
cg12485185		0.000290372	0.312262866
cg05624445	CHST9	7.23E-05	0.312249065
cg00344308		0.006823935	0.312239777
cg06202276		8.60E-06	0.312238863
cg17231690	KRT19	9.24E-05	0.312238496
cg01196858	SCRN1;SCRN1;SCRN1;SCRN1	9.24E-05	0.312229641
cg24333629	SPHKAP;SPHKAP	1.11E-05	0.312227805
cg07329251	AMPD3;AMPD3;AMPD3	0.002714607	0.312220583
cg22134162	THOC7	0.001639597	0.312216479
cg02096793	MFSD7	5.59E-05	0.312212387
cg15000279		0.00043997	0.312210374
cg09516476	GCM2	5.59E-05	0.312207466

cg01534871	C1orf151	1.11E-05	0.312197093
cg25803632	HECW1	4.34E-05	0.312189299
cg23254393		0.000357885	0.312157457
cg11322252		7.23E-05	0.312133755
cg08066943		0.000117986	0.312111593
cg17241310	BARHL2	0.000357885	0.312107887
cg19010054		0.001948248	0.312103551
cg04138502	ADCY5	0.000357885	0.312102253
cg20464360	HSF5	1.96E-05	0.312095507
cg27417469	ADAM32	8.60E-06	0.312092159
cg13067714		0.000117986	0.312089484
cg26400885	ZIC1	0.000148457	0.312087302
cg08843850		0.001147425	0.312074063
cg18521914	C10orf53;C10orf53	1.11E-05	0.312061375
cg05977669	HOXA11AS;HOXA11	0.000187194	0.312054563
cg15603311	CACNA2D3	2.58E-05	0.312046062
cg17781313		8.60E-06	0.312042565
cg22033476	THADA;THADA	0.003734675	0.312033997
cg16419009	EN1	0.000187194	0.312031856
cg15804782	ARNT2;ARNT2	0.000791389	0.312023117
cg05953100		1.50E-05	0.312008837
cg18313221	MET;MET	0.001948248	0.311987936
cg13572145	CTNNA2;LRRTM1;CTNNA2	1.96E-05	0.311983203
cg03989480	DMRTA2	2.58E-05	0.311982772
cg27352639	PCDHA7;PCDHA12;PCDHA6;PCDHA10;PCDH/	2.58E-05	0.311980225
cg15732230	KLK7;KLK7	0.000148457	0.311976791
cg07685786	S100B	0.000148457	0.311970109
cg03550384	NECAB1	5.59E-05	0.311952231
cg27659787	PITX2;PITX2;PITX2	7.23E-05	0.311949382
cg06268921		0.000117986	0.311946311
cg06394247	PAX5	0.000187194	0.311944677
cg08968222	FAM13C;FAM13C;FAM13C;FAM13C	0.000187194	0.311943989
cg01082843	UNC80;UNC80	9.24E-05	0.311943203
cg12995090	LOC100192426;PTPRM;PTPRM	0.000233422	0.311934104
cg23732483	ARIH2	0.009066563	0.311931606
cg17567562	SMARCC1	3.34E-05	0.311928094
cg10239163	DNMT3A;DNMT3A;DNMT3A	0.001948248	0.311923215
cg15096085	C15orf38	0.000117986	0.311921533
cg08694544	RTBDN;RTBDN	0.000148457	0.31190815
cg15852963	SOX2OT	1.11E-05	0.311878714
cg15897209		0.001147425	0.311877691
cg08125821	CSMD3;CSMD3	4.34E-05	0.311867683
cg19967554	UGGT2	0.000187194	0.311862995
cg05050944		5.59E-05	0.311858751
cg16366843		0.001639597	0.311832697
cg01329756		7.23E-05	0.311816593
cg09994356	GDAP1L1	0.000654035	0.31181568
cg13408430	SV2C	5.59E-05	0.311800205
cg14939652	NPAS3;NPAS3;NPAS3;NPAS3	0.000187194	0.311798711
cg08327106	RALYL;RALYL;RALYL	1.50E-05	0.311781216
cg02634272		0.002304201	0.311778731
cg00287096		2.58E-05	0.311776501
cg02147126	AZU1	0.00043997	0.311765719
cg16553435		9.24E-05	0.311762733
cg04829946	PTF1A	1.11E-05	0.311760547
cg07566707		5.59E-05	0.311742882
cg06082548	NKX6-2	8.60E-06	0.311730347
cg06000994	ZNF536	0.000233422	0.311728879
cg26431163		1.96E-05	0.311728863
cg09816471	SNN	0.000654035	0.311722317

cg01852585	NPNT	3.34E-05	0.31171506
cg03192775	GPR12	0.002040485	0.311703913
cg15122993	PCDHA7;PCDHA6;PCDHA10;PCDHA4;PCDHA	7.23E-05	0.311696277
cg03459073		0.000117986	0.311691082
cg18030774	SLC46A1	3.34E-05	0.311687076
cg14282904	KCNG1	0.000955793	0.311679759
cg22753548	TNNI3	0.000955793	0.311672633
cg03152353	NOTCH1	2.58E-05	0.311668529
cg00508024	CTXN2	1.11E-05	0.311660187
cg02052265	C1orf150	0.000187194	0.311649791
cg11847808	EPHA8;EPHA8	0.00043997	0.311647873
cg22876092	GRID1	0.000233422	0.311647061
cg25664938	CDGAP	0.000187194	0.311643515
cg05565239	RTN1;RTN1;RTN1	8.60E-06	0.311629887
cg04950301	PHACTR1	5.59E-05	0.311626434
cg18641486	SLC25A21;SLC25A21;SLC25A21;LOC1001297	4.34E-05	0.311624188
cg07255674	TRIL	0.000654035	0.311617945
cg06400704	FMN2	2.58E-05	0.311603866
cg24182470	CPNE8	0.003734675	0.311600074
cg01576559	FAM46A	0.000654035	0.311598812
cg07839533	HTR1A	0.000187194	0.311584372
cg03390636	ARNT2	0.001639597	0.311576291
cg01747796		9.24E-05	0.311574533
cg16134323	PCDHB16;PCDHB16	1.96E-05	0.311569965
cg04350913	FGF12;FGF12;FGF12	5.59E-05	0.311560906
cg04180046	MYO1G	8.60E-06	0.31155731
cg24795903		0.002714607	0.311542154
cg02060682		3.34E-05	0.311533851
cg08987251	GOT2	0.001147425	0.311518522
cg04939673	C4orf38;WWC2	5.59E-05	0.311505969
cg03850057	SPAG17	0.000357885	0.31150559
cg10583485	DOCK7	5.59E-05	0.311498632
cg05940452	FOXR1;FOXR1	3.21E-05	0.311470958
cg09227621	WNT3A	0.000537905	0.311470288
cg07777790	ACTA1	9.24E-05	0.311441077
cg14783814	MIR137	9.24E-05	0.311426577
cg15122171	PRLR	0.00043997	0.311409301
cg08091147		0.000148457	0.311404123
cg16998150	C1orf114	0.00043997	0.311399521
cg04710764	HOXC10	0.000290372	0.311396818
cg09601629	CCDC105	0.001376172	0.311379914
cg23218760	EFHA2	2.58E-05	0.311368811
cg14830002	OR2B11	0.003189589	0.311367974
cg21166092		8.60E-06	0.311367553
cg11119767	ZAK;ZAK	0.005897668	0.311362079
cg00748599	SEMA3C	2.58E-05	0.311357859
cg20461188		0.0050758	0.311355193
cg02718516	C8orf48	4.34E-05	0.311342859
cg07409200	C13orf30	0.003189589	0.311336813
cg17153727	TFAP2B	1.96E-05	0.311313126
cg27212729	LOC440925;SP5	0.000290372	0.311284877
cg10316490	FAM5C	0.000187194	0.311281724
cg05033803		1.96E-05	0.311280405
cg15164958	VSTM2A	9.24E-05	0.311275904
cg00512279	SLC18A2	0.001376172	0.311275672
cg09975850	CYP7B1	9.24E-05	0.31127067
cg12771281	SLC38A6;TRMT5	0.003734675	0.311269845
cg04221886	ABCG5	0.000117986	0.311268381
cg03483150	PENK;PENK	5.59E-05	0.311265427
cg02150867	LRFN5	0.000537905	0.311263404

cg12749246		8.60E-06	0.311262007
cg02380802		1.96E-05	0.311220536
cg01470340	C10orf105;CDH23	0.000233422	0.311216337
cg12351587		9.24E-05	0.311203781
cg21211882	EPB41L1;EPB41L1	0.000357885	0.311196984
cg00860379		4.34E-05	0.311188777
cg02365780	NPAS2	0.000233422	0.311188668
cg14938677	ARF5	0.000148457	0.311184122
cg15518950	TMEM171;TMEM171	1.50E-05	0.311182748
cg13423098		8.60E-06	0.311182654
cg15309006	CHP2;CHP2	0.000654035	0.311180344
cg27095527	PPARG;PPARG;PPARG	0.001948248	0.311156407
cg20842253	TRIM54;TRIM54	0.000148457	0.311143336
cg20294486		9.24E-05	0.311139071
cg21530280	HAND2;NBLA00301	2.58E-05	0.311134535
cg21618730		0.001376172	0.311133562
cg27629977	CTNNA2;LRRTM1;CTNNA2	0.000205664	0.311127025
cg04888241	ADRA2C	7.23E-05	0.311123605
cg27597956	PAX6;PAX6	5.59E-05	0.311114158
cg01059398	TNFSF10	7.23E-05	0.311105351
cg26388816	B4GALNT3	0.0050758	0.311100424
cg27517968		0.000233422	0.311094828
cg11827910	PAX6;PAX6;PAX6	0.004357816	0.311091881
cg26343001		0.000148457	0.311087601
cg03001942		0.001639597	0.311087111
cg24507762	KCNB1	9.24E-05	0.311086579
cg22387323	CCDC149	0.000357885	0.311072377
cg20889818		0.000148457	0.311053611
cg20076842		0.000148457	0.311050207
cg16590189	CPLX2;CPLX2	1.50E-05	0.311047547
cg22040202		8.60E-06	0.311038922
cg06139341		8.60E-06	0.311030527
cg14359824	C9orf135	0.001948248	0.311023215
cg09401099		0.000654035	0.311014072
cg26195178		0.000955793	0.311007954
cg26841048	HOXB2	0.000955793	0.311007121
cg10343447		0.000537905	0.311006018
cg03166566	RHOD	0.000233422	0.310994391
cg01460227	CALCR;CALCR	1.11E-05	0.310987159
cg06829969	PGD	0.001639597	0.310984398
cg11768647		2.58E-05	0.310981657
cg14249520	CPT1A;CPT1A	2.58E-05	0.310980444
cg25721451	MEIS1;MEIS1	0.000117986	0.310979262
cg04041669	RIC3;RIC3	1.96E-05	0.310977124
cg04561389	FGF14	0.000117986	0.310971496
cg08971771	DLX4;DLX4	6.94E-05	0.310924242
cg08316825	GLRA1;GLRA1	5.59E-05	0.310910116
cg26237512		0.002304201	0.310905453
cg16104915	HOXA9	0.000290372	0.310903951
cg14481208	RTKN	0.003189589	0.310903284
cg15129823	GALR1	0.000290372	0.310898526
cg02229433	MNX1;MNX1	0.000357885	0.310895387
cg13249591	S100A10	0.000233422	0.310888758
cg01690182	GABRB3;GABRB3;GABRB3	0.000187194	0.31088753
cg05089897	DYRK1A;DYRK1A;DYRK1A;DYRK1A;DYRK1A	2.58E-05	0.310882482
cg11089489		5.59E-05	0.310880979
cg06006071		0.001376172	0.310870551
cg18593081		9.24E-05	0.310863432
cg26727768	LOC145845	8.60E-06	0.310858967
cg15081220	KCNG1	0.001887355	0.310853015

cg05417162		0.000791389	0.310851093
cg16312514	SHANK2	0.000233422	0.310850101
cg27093143	APLP1;APLP1	0.000791389	0.310845608
cg16791619		0.000791389	0.310838926
cg00960552	NHLH2;NHLH2	7.23E-05	0.310837342
cg08309529	MNX1;MNX1	8.60E-06	0.310836419
cg27025752	XKR6	4.34E-05	0.310819243
cg07906855		5.59E-05	0.310817611
cg00546005	KCNG1	9.24E-05	0.310817184
cg00503840	DLX5	5.59E-05	0.310810415
cg19507527	FGF12;FGF12	0.000117986	0.310809794
cg17826168	CRB3;CRB3	7.23E-05	0.310807965
cg08383338	PLA2G7;PLA2G7	0.000187194	0.310793983
cg02226672	SMPD3	5.59E-05	0.310787454
cg13804058	NGF	0.000290372	0.31077556
cg18834729	NOS1	9.24E-05	0.310770438
cg03330485	GFRA2;GFRA2;GFRA2	1.11E-05	0.310766303
cg07466705	SLC6A1	0.000537905	0.310749459
cg20850023	IGFBP3;IGFBP3	0.000537905	0.310726042
cg04330513	SFTA3	8.60E-06	0.310714593
cg11982072	GATA5	0.001639597	0.310708756
cg08505883		0.000290372	0.310708026
cg00663972	SOX1	0.001147425	0.310688935
cg17210126	ZFAT;ZFAT	1.96E-05	0.310684416
cg09570974		7.23E-05	0.310683204
cg01612140		0.000148457	0.310656901
cg17200768		2.58E-05	0.310651466
cg20980783		0.000187194	0.310651384
cg13059459		1.50E-05	0.310635038
cg14245102	MEG3;MEG3;MEG3	0.000148457	0.310631446
cg00699219		1.50E-05	0.310627273
cg03388025	SPIRE2	3.34E-05	0.310622087
cg24875854		0.003189589	0.310621768
cg02584459	SLC16A12	0.006823935	0.310616323
cg10488267		8.60E-06	0.310606959
cg20121142	TWIST1	0.00043997	0.310595504
cg14012082	EPX	0.000537905	0.310591067
cg18490616	THNSL2	8.60E-06	0.310585655
cg24358846		1.50E-05	0.310584365
cg02841941	P2RY1	0.000791389	0.310563042
cg00924143		0.00043997	0.310530442
cg01028849	LOC401463	7.23E-05	0.310518546
cg10424681	C6orf201;C6orf146;C6orf146	8.60E-06	0.310517112
cg12616174	MIR124-1;LOC157627	0.004357816	0.3105048
cg14400886	PCDH10;PCDH10	0.000537905	0.31050242
cg09704159	MYEF2	0.001948248	0.310493266
cg16911495		0.000654035	0.310492333
cg02228111	GALNT9;GALNT9	0.000955793	0.31048913
cg06039392	MTNR1A	9.24E-05	0.310477825
cg23676302		9.24E-05	0.310474841
cg16489895	C1orf92	0.000233422	0.310471151
cg02325324		0.001376172	0.310467989
cg23448139	ZFP112;ZFP112	5.59E-05	0.310464045
cg08082810		0.000187194	0.310459364
cg05692123		7.23E-05	0.310458367
cg21252523		0.000233422	0.310454264
cg17500686		7.23E-05	0.310400249
cg00931558	ZFHX4;LOC100192378	3.34E-05	0.310369413
cg12341314	ACTA1	2.58E-05	0.310368854
cg19626725	RUFY1;RUFY1;RUFY1	0.000537905	0.310368132

cg03186486	FAM19A4;FAM19A4	0.000117986	0.310357962
cg23500396	TSPAN18	8.60E-06	0.310354039
cg19499754		0.000290372	0.310353572
cg26780998	PLA2G7;PLA2G7	4.34E-05	0.310346168
cg01734112	C2orf39	5.59E-05	0.310344598
cg03262554		5.59E-05	0.310328618
cg24753662	TBX1;TBX1;TBX1	0.001376172	0.310321182
cg04737991	CHST11	0.000654035	0.310317295
cg11321433		0.001639597	0.310306286
cg02137956		7.23E-05	0.310284183
cg21707187		0.000357885	0.310282762
cg09012471	ARMC8;ARMC8;ARMC8	0.001639597	0.310278644
cg05386493	FOXL2;C3orf72;FOXL2	0.000205664	0.310255222
cg24720192	CYP1B1	0.001639597	0.310245652
cg11600596		0.00043997	0.31023154
cg20259256	TRPC1;TRPC1	0.00043997	0.310230752
cg06196379	TREM1	0.000290372	0.310219453
cg26805749		0.000357885	0.310213602
cg11295178		0.000187194	0.31020381
cg22133562		2.58E-05	0.310202012
cg22615158	WNT5A	0.007869847	0.310186878
cg27483342		1.96E-05	0.310181566
cg01405004	FAM19A4;FAM19A4	3.34E-05	0.310180513
cg13491462		8.60E-06	0.310178042
cg05329888	TYMP;TYMP;TYMP	0.000148457	0.310176593
cg01899797	DAB1	4.34E-05	0.310169009
cg12136088	ASNS;ASNS;ASNS	0.000537905	0.310165697
cg14202850	MAPT;MAPT;MAPT;MAPT;LOC100130148;MAP	0.000290372	0.310157535
cg04062391	ZNF560	7.23E-05	0.310156201
cg00762160	PAX9	0.000537905	0.310155006
cg03571927	DPY19L2P4	0.000233422	0.310144003
cg06213060		0.001639597	0.310143948
cg12156512	FKRP;FKRP	0.000148457	0.310135389
cg04238348	KCNK2;KCNK2;KCNK2	0.000148457	0.310133787
cg05451210		0.000955793	0.310121923
cg12342334	SFRP5	0.000117986	0.310119798
cg18717423		0.000791389	0.310115882
cg24453580	PRPH	0.001376172	0.310111915
cg16335643	ANKRD46	1.96E-05	0.310094959
cg00704633	MFAP3L	0.000290372	0.310093615
cg19974854	POU4F3	0.000187194	0.310091198
cg20014974		9.24E-05	0.310089548
cg23827572	TBX5;TBX5	0.000187194	0.310070478
cg03303774		7.23E-05	0.310066455
cg19726712	LAPTM4B	0.000148457	0.310061521
cg01920578	NELL2;NELL2;NELL2;NELL2;NELL2;NELL2	0.001147425	0.310046379
cg25253705	SLAIN1;SLAIN1	5.59E-05	0.310037514
cg10989897	ELAVL4	0.000148457	0.310027474
cg10115894	SIRPA;SIRPA;SIRPA	8.60E-06	0.310022874
cg05771369	B4GALNT1	0.000187194	0.310004284
cg15409013	ADAMTS16	0.000290372	0.310003906
cg14772925	PGR	4.34E-05	0.309995537
cg20521863		7.23E-05	0.309977668
cg16548780	KIAA0895L;EXOC3L	0.005897668	0.309969327
cg03303585		0.000537905	0.309963712
cg15967709		5.59E-05	0.309930721
cg09412728	HIPK4	1.50E-05	0.309928273
cg01933836		0.004357816	0.309918112
cg07009823	KIF6	0.001639597	0.309917879
cg14826226	CRABP1	5.59E-05	0.309912596

cg08289782	POLR3A	0.001948248	0.309908454
cg22837726	C19orf45	0.000537905	0.309878262
cg18117228	DNAJC6	0.000148457	0.309873464
cg05500015	NAALAD2;NAALAD2	0.002304201	0.309870781
cg20387429		0.00043997	0.309862437
cg23739862	PRMT8;PRMT8	7.23E-05	0.309828249
cg02707869		0.000187194	0.309827666
cg26818735	TWIST1	0.006823935	0.309823975
cg20908936		0.000955793	0.309819279
cg19738283		0.001147425	0.309798888
cg01261351	GFRA1;GFRA1;GFRA1	0.0050758	0.309796478
cg11314471		0.000290372	0.309795629
cg16490906	C5orf36	0.001639597	0.309787474
cg11501438		0.000357885	0.309786599
cg13180315		1.96E-05	0.309785137
cg15996342		0.000290372	0.309775128
cg15697849	DBX2	8.60E-06	0.309770315
cg26049726	EGR4	0.000290372	0.309757406
cg03398865	IRX4	1.50E-05	0.309755937
cg09195234		7.23E-05	0.309748276
cg17330672	OSBP2	0.000654035	0.309743768
cg15144793	SH3BP4	1.96E-05	0.309741221
cg10409302		3.34E-05	0.309737377
cg05928649	CBLN2	0.000187194	0.309733739
cg17222452	MLF1;MLF1;MLF1;MLF1;MLF1	0.000357885	0.309724747
cg04927889	FEZF2	0.000187194	0.309724317
cg22729438		0.000233422	0.309723437
cg24161652	TFAP2B	1.11E-05	0.309705922
cg19925035		2.58E-05	0.309700771
cg06069616	C21orf63	0.000233422	0.309700105
cg25505880	NXF1;NXF1	0.001948248	0.309691162
cg23535596	TFAP2A;TFAP2A	0.000187194	0.309687996
cg26926521	ADRA2A;ADRA2A	0.000233422	0.309668875
cg12565585	RIMS2;RIMS2	0.001639597	0.309663366
cg05707458		0.000233422	0.30965913
cg27444994	CDH8	0.000357885	0.309653365
cg14733720	LYPD5	1.96E-05	0.309630852
cg27454064		0.000654035	0.309623608
cg25531497	GPR158;LOC100128811	4.34E-05	0.309623319
cg07594831	RNF19A;RNF19A	0.000122184	0.309622365
cg02566775	PLAGL1;PLAGL1	0.00043997	0.309621758
cg16622920		0.006823935	0.309617977
cg23318812		9.24E-05	0.309602043
cg10480343	FBN1	0.000791389	0.309594818
cg04144521	MFSD6	0.001639597	0.309589712
cg17338430		0.000148457	0.309555956
cg18419358		0.000290372	0.309547243
cg25417766		4.34E-05	0.309527153
cg15477500	ZDHHC3;ZDHHC3;ZDHHC3	0.000117986	0.309524056
cg04180890	STK32A;STK32A	0.000654035	0.309517605
cg19279257	SLC12A9;TRIP6	0.002304201	0.309515026
cg01097180		0.000148457	0.309493154
cg00219816	C8orf37	9.24E-05	0.309449385
cg13603508	CPNE8	0.009066563	0.30944733
cg00996758		0.000187194	0.309429902
cg27622679	SOX2OT	7.23E-05	0.309429696
cg19624873	SHISA4;SHISA4;SHISA4	0.00043997	0.309427025
cg10779423	C3orf18	0.002714607	0.309424078
cg23877720	VWC2	0.00043997	0.309404991
cg02347527		1.11E-05	0.309387385

cg09134726	PRTN3	0.000654035	0.309381617
cg11853788	TDRD5	7.23E-05	0.309378682
cg09542111	GRIK1;GRIK1;GRIK1;GRIK1	1.11E-05	0.309360715
cg08891559		0.001147425	0.309355611
cg22788065		0.000537905	0.309351002
cg16488737	OPCML	0.000537905	0.30934412
cg01541178		4.34E-05	0.309332159
cg02721000	HOXC4	0.000357885	0.30932806
cg14458068	PCDHGA4;PCDHGA6;PCDHGA1;PCDHGA5;PC	0.000955793	0.309327354
cg26435928		2.58E-05	0.309319247
cg06151165	VSX1;VSX1	0.000187194	0.309314058
cg11468233	DPY19L2P2;DPY19L2P2	8.60E-06	0.3092914
cg10765471		4.34E-05	0.309289588
cg24399337	LOC84856	8.60E-06	0.309287828
cg10249375		0.00043997	0.309287036
cg10453977	EXOC6B	1.50E-05	0.309255723
cg27301230	TPST1	0.000148457	0.309238292
cg15967278		0.006823935	0.309227786
cg16206138	B4GALNT2;B4GALNT2;B4GALNT2	7.23E-05	0.309225388
cg26876974	MSC	1.96E-05	0.309217107
cg07777008	FIBIN;FIBIN	4.34E-05	0.3092165
cg01442799	YAP1;YAP1	0.000791389	0.309214756
cg16145324	MAPK14;MAPK14;MAPK14;MAPK14	0.000866489	0.309206537
cg12935979	POU4F2	3.34E-05	0.309203458
cg18275316		0.001147425	0.309201592
cg18716979	AADAT;AADAT	7.23E-05	0.309190425
cg14661959	TCHH	0.000537905	0.30918745
cg00747922	RPH3AL	0.000148457	0.309183406
cg07073964		0.000148457	0.309171958
cg27465717	LRRC67;LRRC67	0.000955793	0.309161669
cg24842760	EYA4;EYA4;EYA4	0.000791389	0.309159484
cg01728493	ARHGEF10L	0.000357885	0.309149363
cg02836325	PGS1	0.000148457	0.309144854
cg00230700	AGPAT9	0.00043997	0.309143773
cg17226042	MEAF6	0.000117986	0.309121901
cg20260127		4.34E-05	0.309121449
cg25197500	WDR45L	0.005897668	0.309118785
cg04947680		8.60E-06	0.30911295
cg13486532		0.000537905	0.309107889
cg01476003		1.11E-05	0.309104044
cg11586330	LHX5	0.000187194	0.309101332
cg13912964	ZNHIT1	0.000233422	0.309099956
cg17104824	HOXD4;MIR10B	0.000537905	0.309091587
cg09967877	SYT12	0.001948248	0.309088869
cg20216139		7.23E-05	0.309062042
cg08719486	DAPK1	0.003734675	0.309051808
cg09802389	ANXA5	0.001376172	0.309051086
cg07478468	MCOLN2	0.000290372	0.309047237
cg09149149	LOC441177;C6orf176;C6orf176	0.000537905	0.309034055
cg02715546	PYGO1	3.34E-05	0.309028187
cg26057780	BDNF;BDNF;BDNF;BDNF;BDNF;BDNF;BDNF;B	0.000117986	0.30902716
cg25307074	ASPG	0.000117986	0.309016811
cg22354595	MIMT1;PEG3;PEG3;ZIM2;PEG3;PEG3;ZIM2;ZIM	8.60E-06	0.309014492
cg01068808	GLT25D2	0.001147425	0.309000294
cg00085438	MBNL1;MBNL1;MBNL1;MBNL1;MBNL1;MBNL1	0.000187194	0.308994067
cg23204296	SEMA5A	0.001393182	0.308991524
cg08606911	LMX1A	5.59E-05	0.308982477
cg24422027		3.34E-05	0.308979715
cg04451433		7.23E-05	0.308957085
cg25938646	SLITRK1	5.59E-05	0.308952533

cg14051264		2.58E-05	0.308945967
cg17198929	GRID2	0.000233422	0.308934679
cg13478928	LHX8	7.23E-05	0.308931775
cg02331772	SNX7;SNX7	0.000148457	0.308918965
cg20640281	HLF	0.000233422	0.308900653
cg11671598		3.34E-05	0.30888385
cg02498983	CNR1;CNR1;CNR1;CNR1	7.23E-05	0.308880505
cg08726801	CACNA2D4	0.000117986	0.308867254
cg10019916	WNT3A	5.59E-05	0.308867077
cg17617930	ADCYAP1R1	0.000537905	0.308866581
cg03440757	MOSC2	0.001376172	0.308860374
cg04881125	PTGS2	1.96E-05	0.308847486
cg15175143	NXPH2	0.000290372	0.308817743
cg00733005	SIX1	0.000537905	0.308812898
cg14839404	BMP7	0.000357885	0.308803643
cg20034202	B3GNT7	0.000955793	0.30879782
cg20768043	ISL1	0.00043997	0.308797476
cg08933615		0.000654035	0.308785892
cg03578926	SHANK2;SHANK2	5.59E-05	0.308782885
cg19987665	PAX3;PAX3;PAX3;PAX3;PAX3;PAX3;PAX3;PAX	0.000148457	0.3087787
cg23398076	MEIS1	0.000537905	0.308777823
cg06003296	PTPRB;PTPRB	0.002714607	0.308764401
cg27604702	NGEF;NGEF	0.000955793	0.308756582
cg00377074	MGC34034;MGC34034	2.58E-05	0.308752298
cg17074339	GALNTL4	5.59E-05	0.308730917
cg25506726	RASGRF1;RASGRF1	0.000654035	0.308720246
cg07548367	DLX5	0.000791389	0.30870105
cg20937139	PDGFC	0.000791389	0.308684902
cg10004780	ARHGEF4;ARHGEF4	0.000357885	0.308678434
cg22027435		1.96E-05	0.308676911
cg13892295	ARMC4	4.34E-05	0.308648007
cg14096353	PAX2;PAX2;PAX2;PAX2;PAX2	0.00043997	0.308644801
cg10105681		0.000955793	0.308631888
cg20003494	SNCA;SNCA;SNCA;SNCA	0.001147425	0.308629937
cg01277155	SLC26A5;SLC26A5;SLC26A5;SLC26A5;SLC26/	7.23E-05	0.308610137
cg09994418		5.59E-05	0.308604536
cg06879152	PHYHIPL;PHYHIPL	1.50E-05	0.308592305
cg01617695		1.50E-05	0.308587039
cg13024368	DKK3;DKK3;DKK3	7.23E-05	0.308569287
cg10426464	C2CD4B	0.000955793	0.308544229
cg03350439	TRIM67	1.11E-05	0.308541204
cg02989521	ELMOD1;LOC643923;ELMOD1	0.001948248	0.308536213
cg01157070	DKFZP434H168;GNAO1;GNAO1	0.00043997	0.308530498
cg13302154	MGP	0.000791389	0.308518511
cg22715837		0.00043997	0.308514288
cg10172669	BEND4;BEND4;BEND4;BEND4	0.000233422	0.308510552
cg26430305	C4BPA	0.000654035	0.308509702
cg19453997	WWC2;C4orf38	3.34E-05	0.308503046
cg20026798	STOX2	3.34E-05	0.308503023
cg18782991	PCDHB3	0.000148457	0.308502621
cg19653594	SPTBN4;BLVRB	0.001061731	0.308500445
cg19999645	GJD2	4.34E-05	0.308490839
cg26377000	SLFN12L	0.000654035	0.308489176
cg03503087	GFRA1;GFRA1;GFRA1	0.000187194	0.308463109
cg04242021	COL14A1	0.000791389	0.308459045
cg02503874	FERD3L;FERD3L	1.11E-05	0.308454855
cg02909379		1.96E-05	0.30845042
cg18829827		0.000187194	0.308449567
cg11189532	HPCA	7.23E-05	0.308443113
cg14918548		0.000537905	0.308427661

cg21816292		7.23E-05	0.308416774
cg00997079	CTNNA2;LRRTM1;CTNNA2	4.34E-05	0.3083981
cg23731826		0.000117986	0.30839119
cg12079303	NFIA;NFIA;NFIA;NFIA	0.000791389	0.308386688
cg23207990	SFRP2	0.001376172	0.308386501
cg08681855	ZNF578	0.000187194	0.308376305
cg01695643	GSC	0.000148457	0.308373294
cg00107772		1.96E-05	0.308363628
cg03307401	KLK13	0.002450672	0.308332123
cg14455366	NFIB	0.000791389	0.308319088
cg06274396	HELT	0.000187194	0.308318101
cg11781421	HAPLN4	0.000654035	0.308316401
cg18270687		0.001639597	0.308310885
cg07113230		0.000654035	0.30830585
cg20611276	EYA4;EYA4;EYA4	0.000148457	0.308289666
cg02657654		0.005897668	0.3082727
cg14215702	EN2	9.24E-05	0.308269464
cg07214164		5.59E-05	0.308246229
cg21004042		9.24E-05	0.308238303
cg02849695	CCDC19	0.00169692	0.308235384
cg08726248	DRD4	0.000233422	0.308232281
cg26386624	HOXC11	0.000955793	0.308214813
cg22079747	WDR69	0.000187194	0.308208792
cg00517787		2.80E-05	0.308205268
cg03004350	PRTN3	0.000290372	0.308188561
cg08575537	EPO	0.001948248	0.308155268
cg23713079		0.000357885	0.308151366
cg03147002	CADM2;CADM2	9.24E-05	0.308144558
cg26647135	PILRB;PILRB;PILRB	7.23E-05	0.308140979
cg04306507	LGALS3	2.58E-05	0.308122018
cg07012926	GLRA1;GLRA1	3.34E-05	0.308102936
cg23486345	C6orf168;C6orf168	0.000290372	0.308100874
cg14500258	PCDHB2	8.60E-06	0.308094863
cg20049415	NKX2-4	0.001376172	0.308094768
cg10049708	JAKMIP1;JAKMIP1	1.50E-05	0.308079591
cg21777188	PCDHB17	0.001376172	0.308079426
cg05176991	KCTD1;KCTD1;KCTD1	0.00043997	0.308077827
cg01016191		0.001147425	0.308073658
cg17295834		0.000233422	0.308047805
cg26848086	PAX6;PAX6;PAX6	0.000791389	0.308040739
cg24066316	PAX9	0.000117986	0.308034452
cg24351410	DAB1;DAB1	3.34E-05	0.308029364
cg22215631		5.59E-05	0.308027161
cg06734271	NPY1R	5.59E-05	0.308016312
cg20926035	SIX2	9.24E-05	0.30801153
cg25257903	KIAA1755	9.24E-05	0.308004688
cg00719668		0.001376172	0.308003944
cg10654496		1.50E-05	0.307997352
cg26964907	VGLL2;VGLL2	8.60E-06	0.30798702
cg19361865	MOSC2	0.000233422	0.30798646
cg01238669	DNAH11	0.001376172	0.307968252
cg26843324	LHX5	2.58E-05	0.307961569
cg07135446	DLC1;DLC1	4.34E-05	0.307959765
cg08653692	CACNG8;CACNG8	0.000233422	0.307950825
cg04992150	NUP210	0.000117986	0.307942911
cg22975434	KIAA1598;KIAA1598;KIAA1598;KIAA1598	0.000122184	0.307937085
cg26165081	PSMA1	7.23E-05	0.307926347
cg10211298	TMEM108;TMEM108	0.000117986	0.307921961
cg23290121		8.60E-06	0.307914948
cg11706467	KCNJ3	7.23E-05	0.307901744

cg14813603	FGF14	0.000148457	0.307898842
cg02959108		0.000955793	0.307886654
cg02927747	PHLPP1	0.001376172	0.307875777
cg01474257	PYY	8.60E-06	0.307858351
cg27585074	CSNK1G2	0.000148457	0.307857874
cg10675138	PDE10A;PDE10A;PDE10A;PDE10A	0.000117986	0.307850413
cg12575928	GAS2	8.60E-06	0.307836492
cg07994661		2.58E-05	0.307831911
cg25649895	TMEM92;TMEM92	4.34E-05	0.307822689
cg03399933	TDH	1.96E-05	0.307817518
cg25232795	SLC36A1	1.50E-05	0.307817386
cg19586698	OTUD6B	8.60E-06	0.307807945
cg24772240	IGFBP3;IGFBP3	4.34E-05	0.307806628
cg22987447	C3orf55;C3orf55;C3orf55;C3orf55	7.23E-05	0.307805455
cg10689404		0.000357885	0.307803769
cg04904331	VWC2	0.001147425	0.307793671
cg18841159	COL9A1;COL9A1	0.00043997	0.307782775
cg02087931	CALD1;CALD1;CALD1;CALD1;CALD1;C	0.003734675	0.307779784
cg07639520		4.34E-05	0.307779439
cg04222933	LY9	0.001639597	0.307764949
cg14422827	DMRT2;DMRT2;DMRT2	0.001376172	0.30775363
cg19560758	ERRFI1	1.50E-05	0.307739093
cg27279620	MMP2	0.000148457	0.307737838
cg22443982	NLRP14;ZNF214;NLRP14	3.34E-05	0.307732725
cg08799727	SEMA3C	0.000791389	0.307726286
cg08363042	CLIC6	8.60E-06	0.307710513
cg02043329	FNDC3A;FNDC3A	1.96E-05	0.307704843
cg06623560		0.001948248	0.307699037
cg21074347	FLJ46111	0.000955793	0.307698426
cg07573727	VSX2;VSX2	0.000791389	0.307678752
cg12121660	HOXB2	0.000233422	0.307677741
cg18244487	MFSD7	7.23E-05	0.307651341
cg10453425	JPH3;JPH3	0.000117986	0.307650467
cg14902356	ANKRD5;ANKRD5	0.003189589	0.307650192
cg22634891	UNC5C	0.000117986	0.307606424
cg14217069	ONECUT1	0.000148457	0.307586274
cg19491035	MANSC1	0.000187194	0.307564732
cg22809683	LAMC1	0.000148457	0.307563394
cg14649140	HOXA10;HOXA10	0.000290372	0.307557174
cg21317965	TFAP2B	0.000117986	0.307554423
cg24865270		9.24E-05	0.307550834
cg00273449	C2orf65	5.59E-05	0.307543994
cg16405019	PAX7;PAX7;PAX7	4.34E-05	0.307520445
cg22894301		0.000187194	0.307512025
cg15174906		0.000357885	0.307511705
cg19570640	VSNL1	5.59E-05	0.307492306
cg14402591	SEMA6B	0.000148457	0.307491498
cg12874181	LOC401463	1.96E-05	0.307489874
cg14413904		0.002304201	0.307479873
cg26158897	ONECUT1	2.58E-05	0.30747869
cg00591421	SORCS3	2.58E-05	0.307471835
cg21800309	C6orf168	0.000117986	0.307467853
cg22620274	UGGT2	0.001147425	0.307466659
cg26682270		8.60E-06	0.307465232
cg15941948	IRX2;IRX2;C5orf38;IRX2;IRX2	0.001376172	0.307461126
cg27309276	POU4F3	5.59E-05	0.307452814
cg07702750	TRIL	0.000357885	0.307451204
cg10765212	COL5A2	7.23E-05	0.307451058
cg11376198	AKR7L;AKR7L	0.000117986	0.307438666
cg04842157	ADAMTS19	1.50E-05	0.307423094

cg05452899	INS-IGF2;IGF2AS;IGF2;IGF2;IGF2AS;IGF2	0.000233422	0.307414
cg24671344	PDPN;PDPN;PDPN;PDPN	0.000233422	0.307411607
cg18159860		0.00043997	0.307400427
cg26279021	TWIST1	0.000187194	0.307395102
cg05656486	NDUFS2;NDUFS2	0.000290372	0.307378939
cg24390913		0.000537905	0.307378262
cg03675047		2.58E-05	0.30735525
cg08386091	DZIP1;DZIP1;DZIP1;DZIP1	0.000148457	0.307353787
cg14309283	ZBTB22;TAPBP;TAPBP;TAPBP;ZBTB22	0.004357816	0.30734831
cg11324957		0.000187194	0.307347474
cg06211550		0.004357816	0.307340639
cg08454053	RTN4RL1	0.002714607	0.307335719
cg01156834	SRCIN1	0.000955793	0.307320057
cg13286281	SLC7A4	3.34E-05	0.307317674
cg18221429	LRRC61;ACTR3C;LRRC61	0.000233422	0.307289636
cg05929019	LAMC2;LAMC2	7.23E-05	0.307281014
cg25951210	HOXD4	0.000148457	0.307263525
cg13462082	HBP1	4.34E-05	0.307258673
cg06118384	KIF5C	0.000148457	0.307254061
cg01700290		7.23E-05	0.307245131
cg15916646	HOXA11AS;HOXA11	1.96E-05	0.307233192
cg11857320		0.000148457	0.307229742
cg22812955		8.60E-06	0.30722288
cg09032973	PCDHA1;PCDHA1;PCDHA1	8.60E-06	0.307189437
cg12201779	DSCAM	2.58E-05	0.307180533
cg12816979		0.006823935	0.307173555
cg18280362	CWH43	0.00041966	0.307170324
cg00890363		0.000233422	0.30717031
cg08047907	C1orf114	0.000187194	0.307132848
cg17245188	KIF5C	0.00043997	0.307132824
cg04654288		8.60E-06	0.307127712
cg09310966	TREM1	5.59E-05	0.307126696
cg25264081		0.001147425	0.307115438
cg17846334	KCNG1	0.001147425	0.307113572
cg09854626	SOX8	9.24E-05	0.307112455
cg03591753	FKBP5	8.60E-06	0.307109309
cg00564051		1.50E-05	0.307102895
cg23595636	MGC45800	4.34E-05	0.307097768
cg11064255		0.001376172	0.307094074
cg21845953	LOC100192378;ZFHX4	4.34E-05	0.307070822
cg27507591	TTPA	0.000187194	0.307049392
cg26831415	EFHA2	7.23E-05	0.307043584
cg15553418	C1orf144;C1orf144	0.000955793	0.307043483
cg10684547		0.000187194	0.307029429
cg04723534	C2CD2	0.000357885	0.307025411
cg20636352		4.34E-05	0.307013499
cg24267283	SOX2OT	2.58E-05	0.307000617
cg00116946	RPL26L1;LOC100268168;LOC100268168	5.59E-05	0.306994944
cg19599611	AQR	0.000290372	0.306991426
cg12456681	DPY19L2P4	2.58E-05	0.306984677
cg13991908	FAM46A;FAM46A	0.009066563	0.306979002
cg17898823	LGI2	0.0050758	0.306978761
cg25600766	ST8SIA1	0.000117986	0.306977579
cg14400354	GALNT12	0.000654035	0.306961141
cg15524336	RIC3;RIC3	1.96E-05	0.306948441
cg10899637	TFCP2L1;TFCP2L1	0.001376172	0.306934604
cg09956302	HHIPL1;HHIPL1	0.000955793	0.306927833
cg06603997	PAQR5	0.000187194	0.306923651
cg02495518	NRXN1;NRXN1;NRXN1	4.34E-05	0.306912534
cg01431340	C6orf186	0.005093617	0.306895828

cg09985260		3.34E-05	0.306886179
cg00172597	SUSD4;SUSD4	0.000955793	0.306862967
cg22670147	DPP10	0.000290372	0.306859571
cg20895877	PRIMA1	0.00043997	0.306844253
cg01574481	REC8;REC8	0.003734675	0.306827874
cg12738347		0.000117986	0.30681992
cg15489005		0.00043997	0.306818212
cg22920665	EDIL3	0.000290372	0.306811076
cg26294667	FAM110B	0.009066563	0.306798515
cg23815246	FAM46A	0.000357885	0.306784955
cg08205700		9.24E-05	0.306777421
cg14936269	HS3ST6	0.000233422	0.306772287
cg20503109	ST6GALNAC2	7.23E-05	0.306759642
cg25234170	FAM18B2;FAM18B2	0.000791389	0.306726985
cg25806655	SEMA6B	7.23E-05	0.306726562
cg17345081	NMBR	1.96E-05	0.306725646
cg26100099	SPAG17	9.24E-05	0.306719542
cg26727435		0.000357885	0.306719498
cg21768566		2.58E-05	0.306708927
cg12713060		1.50E-05	0.306700619
cg00646347	UTF1	3.34E-05	0.306699498
cg14216285		7.23E-05	0.306697923
cg05523056	ADAMTS18	9.24E-05	0.306687212
cg05205647		0.000233422	0.306680508
cg05929882	TBX5;TBX5	7.23E-05	0.306667437
cg18515591	BTG4;MIR34C;MIR34B	9.24E-05	0.306663235
cg05467160	EVX2	8.60E-06	0.306657228
cg20799917	GALM	0.00043997	0.306655971
cg04959480	SFRP2	0.000537905	0.306621861
cg07588113	C7orf63;C7orf63	0.000357885	0.306606437
cg10189962		9.24E-05	0.306601679
cg03903246	FMN2	5.59E-05	0.306595027
cg09748975	MSX1	0.003734675	0.306582625
cg18238236		5.59E-05	0.306564085
cg09178678		3.34E-05	0.306561429
cg20461912	FOXL2;FOXL2	8.60E-06	0.306553048
cg13452162	CLDN10;CLDN10;CLDN10	1.50E-05	0.306550521
cg15148683		8.60E-06	0.306537302
cg12363472		5.59E-05	0.306518884
cg01234044		8.60E-06	0.306501501
cg19616230	SLC34A2	0.000290372	0.306495817
cg14296561	BIRC3;BIRC3	0.000187194	0.306486584
cg10693058	SPTBN4	0.000148457	0.30646611
cg26305174	SLC12A9;TRIP6	0.001147425	0.30646449
cg24643102	GRM7;GRM7	5.59E-05	0.306459492
cg03876413		0.000357885	0.306449023
cg16458494		9.24E-05	0.306446538
cg11358945	NR4A2	0.000654035	0.306431814
cg20066792		0.000233422	0.306431683
cg05265484	TOM1L1	0.000148457	0.306421507
cg05511722		9.24E-05	0.306417203
cg05374654	GPR83	0.000148457	0.306415086
cg26097011	FGF14	0.001376172	0.306411989
cg19428336	FAT1	0.000791389	0.306411421
cg19041006	PEG3;PEG3;ZIM2;PEG3;PEG3;ZIM2;ZIM2	8.60E-06	0.306407114
cg14114673	GLIS3;GLIS3	0.000537905	0.306392619
cg27350402		2.58E-05	0.306382414
cg21039380		7.23E-05	0.306371142
cg09202227	SMPD3	0.000654035	0.306361063
cg15812348	FEZF2	0.001147425	0.306355703

cg05654290	GDA	1.50E-05	0.306353678
cg01506917	TRERF1	0.000117986	0.306336549
cg08894362	CXCL1	9.24E-05	0.306326834
cg08183724		0.000654035	0.306317928
cg13441156	GHRL;GHRL;GHRL;GHRL;GHRL;GHRL;GHRL;	0.007873393	0.306317776
cg18262616	GJB3;GJB3	0.000187194	0.306314196
cg07334912	DTD1	8.60E-06	0.306304902
cg02849507		0.00043997	0.306291595
cg02192673	NPFFR2;NPFFR2	5.59E-05	0.306256588
cg17451609	EBF2	0.000357885	0.306255789
cg08195247		0.000148457	0.306254624
cg24799909		0.00043997	0.306252734
cg23319477	TAPBP;TAPBP;RGL2;RGL2	0.00043997	0.306251179
cg15591803	DENND2D	1.50E-05	0.306243187
cg05238375	ADRA2A	7.23E-05	0.306225749
cg12721068	HHIPL2	3.34E-05	0.306220288
cg20299697	MRAS;MRAS	3.92E-05	0.306206243
cg23512231		1.96E-05	0.306204457
cg20876760		0.000187194	0.306195548
cg07654012		0.00043997	0.306192116
cg10271819	HIPK2;HIPK2	0.000791389	0.30618302
cg17480760	CA3	0.000262362	0.306178555
cg13862384		0.000791389	0.306172038
cg15668468	VAX1;VAX1	0.000148457	0.306165308
cg11787167	NPAS3;NPAS3;NPAS3;NPAS3	0.001147425	0.306163881
cg02448922		7.23E-05	0.306162691
cg14951193	PLIN5	0.001147425	0.306157382
cg21834061		9.24E-05	0.306143766
cg07382943	IGLON5	5.59E-05	0.306141197
cg08950161		0.000537905	0.306138705
cg14567424	GSTP1	0.000357885	0.306123685
cg23039807		0.000187194	0.306111733
cg03355213	CHMP4B	0.000117986	0.30610555
cg08683938	OPCML	2.58E-05	0.306093687
cg15749148	C10orf79	0.000290372	0.30607687
cg06001419		0.000537905	0.306057379
cg14118997		0.000187194	0.306054327
cg16400631		0.000537905	0.306036043
cg17787159	PNMAL1;PNMAL1	0.003189589	0.30603311
cg13441058		0.000526175	0.306024827
cg06738242	TWIST2	0.000955793	0.306023879
cg18602913		3.34E-05	0.306022115
cg17332198	AVIL	0.000148457	0.30602054
cg06919440	CBLN1	1.96E-05	0.306006201
cg11703632		0.000117986	0.306004937
cg10186400	ENPP5	1.50E-05	0.306004934
cg09511896	WNT3A	0.000365373	0.305996917
cg20489909		5.59E-05	0.305987912
cg21158502		0.001639597	0.305985181
cg03496114		0.000233422	0.305979256
cg02621130		0.000187194	0.305971825
cg25688568		0.000187194	0.305961083
cg18462898	LOC729991-MEF2B;LOC729991-MEF2B;MEF2I	8.60E-06	0.305950659
cg12074545	ZNF35	0.009066563	0.305949984
cg02088996	LOC285954	0.003734675	0.305940492
cg11453719		4.34E-05	0.305919022
cg25844590	PPFIBP2	9.24E-05	0.305917171
cg24218295		8.60E-06	0.305910847
cg02174359	MRPS18A	0.000187194	0.305898899
cg11018769		0.00043997	0.305891306

cg05409340		9.24E-05	0.305877427
cg15885628	OCA2	5.59E-05	0.305873972
cg09156233	BMPR1B	0.0050758	0.305873859
cg01966465	RUNDC3B;RUNDC3B;ABCB1;RUNDC3B	0.000148457	0.305862162
cg05902522	WWTR1;WWTR1;WWTR1	0.000117986	0.305861663
cg25551714		9.24E-05	0.305858578
cg23908228	KCNE1;KCNE1;KCNE1	0.000233422	0.305852075
cg07220939	SLC22A12;SLC22A12;SLC22A12;SLC22A12	1.50E-05	0.305849881
cg03717750	PAWR	0.001376172	0.305829675
cg04127342	PENK;PENK	9.24E-05	0.305826332
cg01777861		2.80E-05	0.305817192
cg22286764		8.60E-06	0.305805272
cg23148467	MSX2	0.000117986	0.305805066
cg07587893		1.11E-05	0.305799628
cg12989851		7.23E-05	0.305788414
cg24424381	WDR93;PEX11A	0.000187194	0.305776137
cg10897223	TRIM40	8.60E-06	0.305770408
cg13832441	PLEKHA9	1.96E-05	0.305754957
cg15870818	TTC14;TTC14	5.59E-05	0.305744716
cg23822879	PDZRN3	0.000187194	0.305727634
cg00460235	ADRA1A;ADRA1A;ADRA1A;ADRA1A	7.23E-05	0.305726875
cg16267322	SLC24A4;SLC24A4;SLC24A4	0.000290372	0.305720325
cg27338487	GRP;GRP;GRP;GRP;GRP;GRP	0.000290372	0.305712946
cg08190291	ADAMTS5	9.24E-05	0.305681999
cg04996873	KIAA1026;KIAA1026	1.96E-05	0.305668067
cg04685170	PON3	0.00043997	0.305667265
cg23378989		0.000233422	0.305656992
cg11945167	CSMD1	0.003189589	0.305655351
cg19907305	COMP	0.000187194	0.305649337
cg07341290		0.000290372	0.305638819
cg21642103	TNXB	0.0059649	0.305635662
cg08278554	C15orf48;C15orf48;C15orf48	0.000537905	0.305635168
cg07420362	TRPC2	0.002714607	0.305634332
cg16508480	ADAMTS16	4.34E-05	0.305626116
cg21146184		0.000537905	0.305619778
cg24169486		0.000117986	0.305615212
cg12894711	BLVRA	0.000233422	0.305614539
cg26172504	KLK7;KLK7	4.34E-05	0.305605903
cg16023415		0.001948248	0.305579241
cg12256206	DACT2	5.59E-05	0.305578002
cg26374305	MEG3;MEG3;MEG3	2.58E-05	0.305569332
cg16099023		4.34E-05	0.305560337
cg07855083	SATB2	0.000205664	0.305558714
cg09736162	CELSR3	3.34E-05	0.305548794
cg21963854	MIR548H4	0.000290372	0.305534223
cg06367693	STOX2;STOX2	4.34E-05	0.305527715
cg15186181	SOX17	0.003189589	0.305517397
cg03363565	RAB11FIP3	0.001948248	0.305493661
cg22897615	PRRT1	0.000955793	0.305491181
cg17389504	MKX	8.60E-06	0.305483411
cg17062279	ITGAE	0.002714607	0.305479475
cg23596620	RPH3A;RPH3A	5.59E-05	0.305472828
cg20870885	TFCP2L1	0.000791389	0.30547091
cg15489979	REM1	1.96E-05	0.305461374
cg10070185	SERPINA1;SERPINA1;SERPINA1;SERPINA1;S	0.000117986	0.305448072
cg09125812	ANK1;ANK1;ANK1;ANK1;ANK1	0.000654035	0.305442819
cg03216529	TFAP2C	0.000117986	0.3054385
cg00500564	FAM196A;DOCK1	0.000233422	0.305435597
cg04187935		0.000117986	0.305434052
cg27332878	ARNT2	0.000148457	0.305429057

cg06974428	PIP4K2A	9.24E-05	0.305417133
cg20730770	PCDHB16	5.59E-05	0.305415677
cg26337312	LMX1A	0.000791389	0.305406951
cg07545037	HOXC4;HOXC5;HOXC6;HOXC6	8.60E-06	0.305404459
cg03642463	HRNBP3	0.000537905	0.305392119
cg23885415	VAX2	0.000791389	0.305376043
cg09258804	TBX4	1.50E-05	0.305375964
cg02976109	GTF2H3;EIF2B1	7.23E-05	0.305374687
cg14214706	FGF14	0.000233422	0.305371274
cg25026992	PCDHB7;PCDHB7	0.003189589	0.305369286
cg00294109	CRBN	0.000290372	0.305357508
cg19203333		0.000187194	0.305350783
cg02906238	TFCP2L1;TFCP2L1	0.000955793	0.305346525
cg24316515	NKAIN3	0.000187194	0.30534194
cg17003736	ZIC4;ZIC4;ZIC4;ZIC4;ZIC4	0.000117986	0.305338776
cg01856162	ECEL1	9.04E-05	0.305335477
cg24733530		0.000233422	0.305333717
cg22265644	NKX2-2	0.000117986	0.305326547
cg02956542	PGLYRP4	0.000148457	0.305309954
cg00513220	MOSC2	3.34E-05	0.305299995
cg25313172	KRT7	9.24E-05	0.305268186
cg04225089	TRIM47	9.24E-05	0.305263175
cg00484711		0.001376172	0.305252584
cg19411008		4.34E-05	0.305225362
cg25784280	DTNA;DTNA;DTNA;DTNA;DTNA	0.003734675	0.305206819
cg13353699	WASF3	0.000654035	0.305204672
cg21069005		0.001639597	0.305202986
cg24884703		0.000187194	0.305197988
cg14927519	CREB5;CREB5	0.003189589	0.305175296
cg13683827	FAM113B	7.23E-05	0.305166717
cg03381237	RBM33	4.34E-05	0.305153375
cg08153621		0.000233422	0.305150951
cg17080163		0.000357885	0.305139508
cg17679781		1.96E-05	0.305136706
cg02075820	NUCB2	0.000955793	0.305125377
cg08278892	RDH13;RDH13;RDH13;RDH13	1.96E-05	0.305120527
cg13325154	PITX3	0.000955793	0.305100017
cg01613010		9.24E-05	0.305091844
cg24685998	LRIG3;LRIG3;LRIG3	2.58E-05	0.305090397
cg23006497		0.000117986	0.305088652
cg19418458		0.001147425	0.305079902
cg01524278		1.11E-05	0.305071717
cg04340729		3.08E-05	0.305071192
cg13027965	PHACTR2;PHACTR2;PHACTR2;PHACTR2	0.001948248	0.305067451
cg06383163	FAM135B	0.000290372	0.305064126
cg10635194	KCND3;KCND3	0.000187194	0.305063713
cg01416712	PHOX2B;PHOX2B	0.000537905	0.305063203
cg10435849	COL6A2;COL6A2;COL6A2	0.001639597	0.305044688
cg04326198		0.00045851	0.305041745
cg26116742	GABRG2;GABRG2;GABRG2	0.000117986	0.305039564
cg25021259	PPP2R2B;PPP2R2B;PPP2R2B;PPP2R2B;PPP2	7.23E-05	0.305034618
cg08943494	PRR5L;PRR5L;PRR5L;PRR5L;PRR5L	0.001376172	0.305021164
cg06571387	HOXD12	1.11E-05	0.305013038
cg18852765	BAIAP2L1	0.000955793	0.304983167
cg16909733	ANKMY1;ANKMY1	0.000233422	0.304974359
cg00508543	ADAMTS9	0.000233422	0.304950944
cg13690178	SLC2A10;SLC2A10	0.000537905	0.304950524
cg05937194	PCOLCE2	0.000791389	0.304942175
cg08175609		8.60E-06	0.304934536
cg14120703	NOTCH1	8.60E-06	0.304922752

cg05563515	RNF39;RNF39	0.00043997	0.304917339
cg22410057	C15orf38	7.23E-05	0.304907984
cg08295543	IRX6	1.96E-05	0.304899402
cg06832008		5.59E-05	0.304895993
cg12949975	GDF7	0.000866489	0.304888186
cg26499055	SIM1	9.24E-05	0.304873816
cg09761352	CRB3;CRB3;CRB3;CRB3	0.000791389	0.304873015
cg19346371	TBX3;TBX3	7.23E-05	0.304866002
cg07920525		0.001639597	0.304859913
cg00919411		4.34E-05	0.304842859
cg00842325		7.23E-05	0.30482789
cg18534992		5.59E-05	0.304819889
cg25929533	C6orf174	2.58E-05	0.304787607
cg24002167	EDIL3	0.000148457	0.304787427
cg12405599	RPN1	0.001147425	0.304783786
cg00184732	GLDN;GLDN	1.96E-05	0.304781084
cg10043037	FERD3L	3.34E-05	0.304773932
cg23501406	DRD4	0.00043997	0.304772822
cg13643796	ADAMTS3	0.00043997	0.304759881
cg12188268	HHIPL1;HHIPL1	0.000290372	0.30475919
cg00974864	FCGR3B;FCGR3B	0.000654035	0.304746192
cg11950360	BRUNOL4;BRUNOL4;BRUNOL4;BRUNOL4	5.59E-05	0.304742542
cg14037837	PAX6	7.23E-05	0.304735368
cg10599770	FAM3B;FAM3B	0.001147425	0.304733684
cg04750910		4.34E-05	0.304723827
cg26104475	LCP2	2.58E-05	0.304705602
cg14410589		2.58E-05	0.304701835
cg19705910		2.58E-05	0.304696993
cg00886554	NMU	0.000955793	0.304690348
cg24156796		1.50E-05	0.304686888
cg01499172	DAAM2	0.000791389	0.304677251
cg24216596	WNT5A	0.005897668	0.304675967
cg24288310	ST8SIA2	2.80E-05	0.304675967
cg16485682	GATA6;GATA6	0.006823935	0.304670982
cg26696162	ZBTB2	0.000791389	0.304661785
cg09177106		7.23E-05	0.30465306
cg18010752	WNT5A	4.34E-05	0.30463469
cg12031275		0.000537905	0.304634143
cg12398330	ROBO3	0.000148457	0.304625998
cg03246914	TUBB1	0.000290372	0.304623059
cg05110943	DSC2;DSC2	0.007873393	0.304620514
cg19815574	DACT3	8.60E-06	0.304617982
cg10533990	FOLR3	0.00043997	0.304610793
cg09799571	LIN28	0.000791389	0.304598956
cg22213821	TMEM98;TMEM98	2.58E-05	0.304566831
cg08813325	SORCS2	5.59E-05	0.30456059
cg24853724	TRIL	9.24E-05	0.304544416
cg01435574	BARX2	0.005897668	0.304526245
cg20992114	PITX2;PITX2;PITX2	7.23E-05	0.304491234
cg15778745	SLITRK5	0.003189589	0.304477479
cg25771096	C11orf87	3.34E-05	0.304470592
cg24033871		0.000791389	0.304467579
cg21619814	PCDHA2;PCDHA1;PCDHA1;PCDHA2;PCDHA2	4.34E-05	0.304458372
cg16268165	UNC5A	0.000117986	0.304452331
cg10687644	ATG16L2	0.000187194	0.304444489
cg11204139		3.34E-05	0.304431386
cg03183058		0.000233422	0.304413729
cg27085869	TMEM171;TMEM171	1.96E-05	0.304401411
cg21449150	SPATA18	0.000654035	0.304398576
cg04806942		0.000357885	0.304388879

cg25352328	GLRA1;GLRA1	1.50E-05	0.304379577
cg17495715		0.001376172	0.304374047
cg22473637	MCHR2;MCHR2	0.003734675	0.304373818
cg05626128	IFFO2	0.000148457	0.304373581
cg26986911	RXFP3	0.001948248	0.304353809
cg03350186	PRRX1;PRRX1	5.59E-05	0.304334289
cg26021714	RNASE2	2.58E-05	0.304323315
cg02132560	MT1M	0.000357885	0.304309488
cg16541852		0.00043997	0.304305065
cg14422498		0.000187194	0.304291981
cg23262036		0.0050758	0.30427936
cg01412469	INHBA;LOC285954;LOC285954	0.000654035	0.304273647
cg25326952	PDIA4	4.34E-05	0.304269449
cg20390045	RAB3C	0.000357885	0.304248444
cg01413809	MCOLN2	7.23E-05	0.304245374
cg08476244	EPB41L5	0.003734675	0.304232468
cg15375469	CPVL;CPVL	0.00043997	0.304232207
cg22057720	SLITRK1;SLITRK1	7.23E-05	0.304222659
cg13944018		0.001639597	0.304221287
cg02339211		0.001376172	0.304215477
cg05290300		0.003734675	0.304199934
cg20026178	WRNIP1;WRNIP1	0.001147425	0.304165125
cg26942250	SNRPA1	1.50E-05	0.304164732
cg14507533		7.23E-05	0.304162995
cg03671052		0.0050758	0.304148355
cg04638468	TMEM155;LOC100192379	0.001948248	0.304140753
cg14904464	NELL2;NELL2;NELL2;NELL2;NELL2;NELL2	0.001376172	0.304136316
cg06076122	CDH18;CDH18;CDH18;CDH18	7.23E-05	0.304132498
cg13211683	GDF6	0.000148457	0.304115386
cg23603891	HES2	0.00043997	0.304086293
cg17878972	HRASLS;MGC2889	0.000791389	0.30407532
cg03804272	PDE4D;PDE4D	2.58E-05	0.304074709
cg06269115	GAL	0.003734675	0.304067258
cg13388983	RHOD	0.000117986	0.30406413
cg13037201	PTPN21	2.58E-05	0.304061793
cg05536800	HAND2;NBLA00301	0.000148457	0.304061443
cg04052466	AMH	0.006823935	0.304050234
cg21112168		0.000290372	0.304043291
cg03604367	SCIN	0.000148457	0.304034841
cg19732566		0.000148457	0.304021681
cg02100819		9.24E-05	0.304007924
cg16413030		0.000654035	0.304005001
cg24600895	NLGN1	8.60E-06	0.304002218
cg14111928	MYST4	0.000117986	0.303992376
cg26784011		7.23E-05	0.303983582
cg06253058	WWTR1;WWTR1;WWTR1	5.59E-05	0.303982846
cg13607226	GGTA1	0.000117986	0.303976141
cg10738239	SOX21;SOX21	1.96E-05	0.303972705
cg27482605	C19orf51	0.00043997	0.303969851
cg08703231	C16orf68	0.000654035	0.303968705
cg08368973	C11orf9	0.004357816	0.303966183
cg27603283	MMD2;MMD2;MMD2;MMD2	9.24E-05	0.30394736
cg22084410		0.000290372	0.303944223
cg23527621	ECE2;CAMK2N2	4.34E-05	0.303940739
cg21712322		2.58E-05	0.303936521
cg04884011	PRKG1;PRKG1	0.001147425	0.303926313
cg17421623	KTELC1;KTELC1	0.000117986	0.303918596
cg26146542	DTNA;DTNA;DTNA	7.23E-05	0.303918532
cg14896134		9.24E-05	0.303917826
cg16329784	PITX3	0.000537905	0.303913758

cg17084653		1.11E-05	0.303907647
cg19192280	PRRT1	0.000368365	0.303905876
cg05791173		0.000955793	0.303896923
cg17907003		1.96E-05	0.303896263
cg14838433	TMEM61	0.000357885	0.303895083
cg07717903	PCDHGA4;PCDHGA1;PCDHGA6;PCDHGA5;PC	0.000187194	0.303886521
cg09326843		8.60E-06	0.303880707
cg01897498	ANKRD19	1.50E-05	0.303865062
cg00975624		0.001147425	0.303858048
cg16236766	NOTO	0.000654035	0.30385042
cg22389871	FAM46A	0.001147425	0.303847104
cg05321361	IRX2;IRX2	0.00043997	0.303838878
cg15058253		1.11E-05	0.303829058
cg02541031	STK33	5.59E-05	0.303817944
cg02856190	FUT4;FUT4	0.001639597	0.303809826
cg14826456	ADRB1	7.23E-05	0.303781207
cg05874732	TMC2	4.34E-05	0.303769115
cg10931792		9.24E-05	0.303747097
cg14362312	ADAMTS13;ADAMTS13;ADAMTS13;ADAMTS1	0.000290372	0.303725788
cg10655144	TAAR3	0.000537905	0.303725482
cg15765724	GBX2	0.001639597	0.303711313
cg10573018	RSPO1	0.003189589	0.303701035
cg06527213	KLHL25;MIR1276	1.50E-05	0.303694945
cg23689615	LOXL1	0.000148457	0.303690343
cg07283833	RIPPLY2	2.58E-05	0.303664747
cg01037890	PHYHIPL;PHYHIPL	1.96E-05	0.303662185
cg27410337	NMU	4.34E-05	0.303660867
cg21616935	CCDC11	0.000148457	0.303649258
cg14614211	MKX	1.11E-05	0.303623969
cg09643544	ZNF177;ZNF177	1.96E-05	0.303618238
cg08898653	CAMK2N1	0.000290372	0.303593703
cg15687855	ZNF502;ZNF502;ZNF502;ZNF502	3.34E-05	0.303590931
cg14818701	CSGALNACT1;CSGALNACT1	0.000290372	0.303590581
cg16370398	HOXC4;HOXC4	0.000791389	0.303582243
cg05007997	SLC11A1;SLC11A1	7.23E-05	0.303580664
cg06817516	DUOX2	0.000233422	0.303580113
cg13624385	PCDH7;PCDH7;PCDH7	0.000233422	0.303574073
cg14515453	TWIST1	9.24E-05	0.303565209
cg05574272	RXFP3	1.96E-05	0.303563141
cg21809624		7.23E-05	0.303547552
cg16907934	FAM50B	1.96E-05	0.303545432
cg07204747	FCHSD1	0.000187194	0.303532799
cg05994974	PARP12	0.005897668	0.30352904
cg06188545	PEAR1	0.001948248	0.303515561
cg16300565	ZFR	0.000537905	0.303502398
cg16361302		0.000791389	0.303499014
cg02572463	SEMA3E;SEMA3E	5.59E-05	0.303492295
cg12400956		0.003734675	0.303485145
cg14431006	BNC1	0.000357885	0.303478596
cg25581222	CSNK1G1	4.34E-05	0.303465576
cg18657094		0.000537905	0.303445486
cg27343616	PCSK2;PCSK2	8.60E-06	0.303442358
cg19279310		0.000955793	0.303432618
cg22853064	ICAM5	0.000357885	0.303431168
cg13910860	PAX5	0.000233422	0.303407358
cg27120934	LAMA2;LAMA2	0.001639597	0.303395834
cg13099330	RBP1;RBP1;RBP1	7.23E-05	0.303394756
cg02056062	WWTR1;WWTR1;WWTR1	1.11E-05	0.303375928
cg24594997	RBP1;RBP1;RBP1	0.000357885	0.303359322
cg14246335	SLITRK1	0.000148457	0.303358074

cg12018098	SPRED3	0.000290372	0.303353407
cg22091421		0.000117986	0.303328937
cg14092045		0.003734675	0.303323139
cg15627089		0.0050758	0.303310731
cg21903395	TMEM132D	0.000290372	0.303310499
cg17285931	HDAC4	1.11E-05	0.303308841
cg19521832	KCNE1;KCNE1;KCNE1	9.24E-05	0.303305768
cg04324615	FBLN7;FBLN7	0.000233422	0.303291421
cg21187554	ANKRD18A;ANKRD18A;C9orf122	0.000233422	0.303283929
cg03724964	FBXO17	5.11E-05	0.303280996
cg11341144	TRA2B	0.000955793	0.303279706
cg18350895	PRKD1	0.000357885	0.303277412
cg16562776		4.34E-05	0.303273344
cg27420236	RPRM;RPRM	0.000117986	0.303271986
cg07253829	DPPA4	7.23E-05	0.303262819
cg08493063		0.000955793	0.303259995
cg21019253	OVOL2	5.59E-05	0.303253081
cg25699034	C17orf95;JMJD6;JMJD6	0.001639597	0.303246884
cg09071155	EVX1	0.000233422	0.303240805
cg14767442		9.24E-05	0.303232259
cg07678904	FSD1	0.000148457	0.303231081
cg13567403		0.000117986	0.303217529
cg14005246		0.000537905	0.303210095
cg10095020	ADAMTSL5;ADAMTSL5	0.002714607	0.303201897
cg04272086	DCC	1.11E-05	0.303198058
cg02059637	GRB14	8.60E-06	0.303197719
cg26005232	NUDC	4.34E-05	0.303194584
cg23352712	LOX	0.000233422	0.303154043
cg12420755		0.000791389	0.30315011
cg22633280		0.001147425	0.303146103
cg14419435		0.000117986	0.303141622
cg07379055		0.000955793	0.303138758
cg17716453	CYP4F22;CYP4F22	0.000654035	0.303136282
cg15724256	EN1	0.000233422	0.303133871
cg09095551	SEC14L5	0.000537905	0.303127685
cg07140792		1.50E-05	0.303127446
cg16605327	TBX5;TBX5	5.59E-05	0.303113904
cg15412772	MED24;MED24	5.59E-05	0.303112103
cg11464895	UTF1	1.96E-05	0.303108806
cg14221550	MAL2	0.001147425	0.303096397
cg17498773	RAB39	0.000117986	0.303089724
cg17168157	RGS22	0.009066563	0.303084145
cg01261007	GPR6	1.50E-05	0.303073857
cg01400884		1.11E-05	0.303069177
cg06609489	NOL4	0.000791389	0.303060241
cg13601595	RP1L1	0.00043997	0.303053036
cg14187844		0.000187194	0.303052313
cg19284211	INSM1	7.23E-05	0.303050336
cg21405799	NECAB2	4.34E-05	0.303044391
cg17769793	FAM13A	0.002714607	0.303007996
cg14588078	LOC401463	5.59E-05	0.303000754
cg10058779	SLC22A17;SLC22A17;SLC22A17	7.23E-05	0.302977788
cg20168837	C1orf83	3.34E-05	0.302958544
cg19094438	BCAR1;BCAR1;BCAR1;BCAR1;BCAR1;BCAR1	0.00043997	0.302943121
cg06062084	ACADL	0.00043997	0.302941681
cg14559259	CCDC140	0.00043997	0.302936292
cg10900696		0.000117986	0.30293393
cg09393619		9.24E-05	0.302927375
cg05155704	FAM83B	0.000148457	0.302926655
cg15628917	C1orf87	0.000955793	0.302905351

cg14696334	RCCD1;RCCD1	0.000537905	0.302904916
cg24056365		0.001948248	0.302889999
cg16291589	LRRN4	1.96E-05	0.302881917
cg04682905	FBXL18	9.24E-05	0.302865419
cg19430577	STEAP4	0.000233422	0.302851418
cg13492364	FMN2	0.000233422	0.302846493
cg19224201		0.000117986	0.302839388
cg00592870		0.000187194	0.302837841
cg26516935		0.000290372	0.302835611
cg00699986	VSX1	0.000537905	0.30283483
cg04968806	SLC6A5	1.50E-05	0.302821735
cg14101302	RIMS1	0.000791389	0.302821318
cg14125604	FLRT2	0.000148457	0.302819962
cg11557636	KIAA1239	0.000290372	0.30279459
cg19981991		2.58E-05	0.302783435
cg02768694	C2orf65	8.60E-06	0.302771534
cg00818198		0.000357885	0.3027578
cg13541353	ACO1	0.000187194	0.302740893
cg23805360	DMRT3	3.34E-05	0.30272036
cg24596116		0.000955793	0.302716481
cg01065806		4.34E-05	0.302707341
cg09639931	ZPBP2;ZPBP2	8.60E-06	0.302706887
cg08241330	ABAT;ABAT	1.50E-05	0.302702002
cg26988406	BHLHE22	0.000117986	0.302700149
cg27486427	RARB;RARB;RARB;RARB	9.04E-05	0.302687748
cg10796899	DIP2C	0.004357816	0.302687196
cg25234732	LOC389493	8.60E-06	0.302675789
cg09823288		1.96E-05	0.302672063
cg19040077	D2HGDH	0.000955793	0.302667855
cg27634020	AGAP1;AGAP1	0.000187194	0.302635174
cg09797463	LIPA;LIPA	0.000187194	0.302632211
cg23054181	FAM169B	0.003734675	0.302625597
cg11438755	TMEM20;TMEM20	3.34E-05	0.302619702
cg00382944	PCSK9	0.000117986	0.302590468
cg20704159	RPH3A;RPH3A;RPH3A;RPH3A	2.58E-05	0.302581885
cg01524174	SOX9	0.001376172	0.302556098
cg18491375	PLA2G7;PLA2G7	5.59E-05	0.302554087
cg24880056	ASXL3	0.002304201	0.302541679
cg15762032	GAD2;GAD2	0.000357885	0.302540518
cg16621364	PCDHGA4;PCDHGA2;PCDHGA5;PCDHGB2;PC	4.34E-05	0.302532742
cg20064455	WNT2;WNT2	4.34E-05	0.302531116
cg19184934	SOX21	4.34E-05	0.302524672
cg14405924	EBF3	2.58E-05	0.302516928
cg24092914	VHL;VHL	0.000233422	0.302516285
cg08883213		0.000187194	0.302516179
cg11480762	SLC29A3	2.58E-05	0.302486866
cg21814870	LPHN3	0.000117986	0.302476539
cg25439973	RAD21L1	0.000290372	0.302473425
cg05970811	SRGAP1	1.50E-05	0.302466616
cg16629158	TFCP2L1	0.000654035	0.302464973
cg11533098	FAM84A	7.23E-05	0.302443584
cg12964144	WSCD1	4.34E-05	0.302439726
cg21381065	DCC	9.24E-05	0.302420032
cg16065899	ALX4	0.000117986	0.302419149
cg09272849	UACA;UACA	0.000537905	0.302401
cg09233651	TBX5;TBX5;TBX5;TBX5	0.000791389	0.302400289
cg02119348	A2BP1;A2BP1;A2BP1;A2BP1	8.60E-06	0.30239593
cg00352218		5.59E-05	0.302374609
cg07570570	PRIMA1	0.000290372	0.30235221
cg08536228	SYNPR;SYNPR	0.00043997	0.302349792

cg25109019	SOX2OT	1.60E-05	0.302344687
cg02700894	SYN2;SYN2	0.000654035	0.302305272
cg07217924	PTPN5;PTPN5;PTPN5	5.59E-05	0.30226033
cg19102771	CCDC140	8.60E-06	0.30225135
cg16415058	SORCS1;SORCS1	5.59E-05	0.302250591
cg13424029		0.000537905	0.30224541
cg24456654		0.000148457	0.30223895
cg12484370	NFIB	0.001376172	0.302236401
cg04128746	FAM196A;DOCK1	0.001639597	0.302227349
cg14320054		3.34E-05	0.302225861
cg21051580	MMD2;MMD2	0.000117986	0.302215569
cg04101194	SLC34A2	0.000187194	0.302183662
cg02264082		4.34E-05	0.302183658
cg21155316	LGTN	4.34E-05	0.302183419
cg08170911	PRKCD;PRKCD	3.34E-05	0.302155746
cg12169536	CDH2	0.000955793	0.302151203
cg12659883		0.001376172	0.302148164
cg24899806	KCND2;KCND2	8.60E-06	0.30214466
cg07464571	LEP	1.96E-05	0.302135509
cg18290233		0.000148457	0.302131061
cg19990022	PCDHB1	0.000955793	0.302121796
cg13552201	APCDD1L	2.58E-05	0.302103935
cg04476070	ZC3H18	3.34E-05	0.302092084
cg09950916		0.007873393	0.302090366
cg23472708	MNX1;MNX1	0.003189589	0.30207278
cg16621833	VGLL2;VGLL2	0.001147425	0.302066526
cg02346737	ARHGAP26;ARHGAP26	0.000290372	0.302062685
cg20812988		1.96E-05	0.302050908
cg16581536	FOXA1	0.002714607	0.302046739
cg01579765	HSF2BP	0.005897668	0.302030739
cg07477922	NANOG	0.000357885	0.302024369
cg23799901	GPR37	0.000187194	0.302024043
cg18694169	NKAPL	0.000955793	0.302004311
cg21883293		1.11E-05	0.302003347
cg21095561	C17orf104	9.24E-05	0.301993084
cg20457732		0.000537905	0.301989964
cg03919488	PPM1H	0.00043997	0.301985097
cg09326204	CASR	0.000290372	0.301963946
cg10830145		0.006823935	0.301962516
cg18276638		0.006738759	0.30196212
cg04905434	MAFA	0.000117986	0.301954866
cg07979236		0.001948248	0.301943892
cg11943209	OSR1	2.58E-05	0.301936675
cg19476053	CCDC140	1.11E-05	0.301917903
cg20329085	ASXL3	0.001948248	0.301904489
cg26398656	OSBPL1A;OSBPL1A	0.007873393	0.301898292
cg07711192		0.000117986	0.30189506
cg22619824	ST7;ST7;ST7OT2	5.59E-05	0.301888676
cg00455526	TRIL	0.0050758	0.301887784
cg13874425		0.001639597	0.301887526
cg06049781	CYP26B1	0.001948248	0.301885122
cg13314310	WDR35;WDR35	9.24E-05	0.301883472
cg01196322	PRDM6	9.24E-05	0.301874505
cg24871887		5.59E-05	0.301873921
cg08427067		0.000187194	0.30187297
cg21117330	PCDHGA4;PCDHGA6;PCDHGA1;PCDHGA5;PC	0.000357885	0.301868241
cg12486537	PPP1R3C	0.003734675	0.301867774
cg22829917	HOTAIR	3.34E-05	0.30186692
cg23596233	B3GAT1;B3GAT1	0.001147425	0.301862093
cg26416811	RFX6	8.60E-06	0.301860864

cg09078517	KCND2	9.24E-05	0.301859708
cg26076905	PIK3R1	5.59E-05	0.301844743
cg08132815	UHRF1;UHRF1	3.34E-05	0.301842053
cg01557989	SLC24A3	0.000148457	0.301841229
cg23357708	ZNF414;ZNF414	0.0050758	0.301821932
cg21350697	LRRC27;LRRC27;LRRC27;LRRC27;LRRC27	0.000955793	0.301818583
cg14505704	CDH6	3.34E-05	0.301817176
cg19147415	RAB30	0.000654035	0.30178445
cg26021960		4.34E-05	0.301776621
cg03505117		0.000187194	0.30177523
cg07323825		0.000233422	0.301772849
cg16587952		0.001948248	0.301766232
cg19024969	C17orf102	0.000187194	0.30176563
cg04362887		1.96E-05	0.301762054
cg04483289	SKAP1;SKAP1	0.002304201	0.301751217
cg24621972	PAX3;PAX3;PAX3;PAX3;PAX3;PAX3;PAX3;PAX	0.000233422	0.301741775
cg14119337	MEG3;MEG3;MEG3	8.60E-06	0.301738876
cg01920232	RFX4	3.34E-05	0.301735037
cg22676693	DAB1	7.23E-05	0.301727281
cg01348584	RADIL	0.001639597	0.301724603
cg16670554	LBXCOR1	0.000791389	0.301721776
cg03504078	PCDHB3	4.34E-05	0.301718942
cg19594218	L1TD1;L1TD1;L1TD1;L1TD1	1.11E-05	0.301715586
cg26298409	MKX	0.000117986	0.301708761
cg05742247	RSPO2	1.96E-05	0.301700435
cg13706544	LHFPL3	4.34E-05	0.301694359
cg08454687		0.000117986	0.30168842
cg13655674	TBX15	0.000187194	0.301683426
cg25705508	CHIT1	0.000290372	0.30168082
cg19407095	SOX1	0.000537905	0.301680105
cg12134633	SCG5;SCG5	0.000537905	0.301673544
cg01596292	RNF19A;RNF19A	0.000187194	0.301661812
cg15646817	RASGRF2	0.000791389	0.301660476
cg04097639	NR5A2;NR5A2	0.006823935	0.301643997
cg23370213	SLC7A2	0.000357885	0.30164349
cg26727693	AVPR1A	4.34E-05	0.301626417
cg21389309	DPP6;DPP6;DPP6	0.000148457	0.301616968
cg18535011	CDH6	0.000187194	0.30160811
cg03529432	HOXA6	0.001376172	0.301602424
cg04088697	DLX6AS	9.24E-05	0.301597168
cg19727439	GRIK3	0.003189589	0.301570568
cg03670381		0.000117986	0.301565426
cg20155875	WIPI1	0.000654035	0.301562885
cg06897628		2.58E-05	0.301557849
cg25301406	DNER	0.001147425	0.301548957
cg09987740	GULP1	2.80E-05	0.301544145
cg21306006	LOC441177;C6orf176;C6orf176	4.34E-05	0.301539464
cg09100271		0.000148457	0.301519549
cg16474170	PTPRM;PTPRM	0.005897668	0.301517318
cg22258315		4.34E-05	0.30151719
cg00685291	ASPG	0.000537905	0.301508374
cg02851062	BSX	3.34E-05	0.301489714
cg24644551		2.58E-05	0.301469904
cg12800028	GPR6	2.58E-05	0.301467164
cg07598952	NMBR	3.34E-05	0.301464817
cg06076323	ARHGEF10	0.000357885	0.301460686
cg23260993	EFEMP1;EFEMP1;EFEMP1	0.003189589	0.301458745
cg03716999	RNF207	0.006823935	0.301454613
cg08148553	AP1M2	0.002304201	0.30145215
cg03882242	C12orf42;C12orf42	0.002304201	0.301450465

cg23845889	MTMR7	8.60E-06	0.30144712
cg21223983		0.002304201	0.301432043
cg09247619	PTPRC;PTPRC;PTPRC;PTPRC	0.000233422	0.301431796
cg03772350	FOXA1	8.60E-06	0.301430319
cg12670477	IGSF22	7.23E-05	0.301423186
cg14565725	TBX15;TBX15	0.00045851	0.301422721
cg07392724	GPR37	4.34E-05	0.301419624
cg14282221		1.96E-05	0.301417136
cg14239811	AADAT;AADAT	0.000654035	0.301410907
cg17651959		9.24E-05	0.301396862
cg19419146	STK32A;STK32A	4.34E-05	0.301393008
cg01308258	CNTNAP5	2.58E-05	0.30138343
cg09657265	SMPDL3A	0.000357885	0.301376114
cg26332740	WNT3A	0.000187194	0.301368282
cg04036920	C11orf41	3.34E-05	0.301344203
cg20104341	HOXC13	7.23E-05	0.301336255
cg13998223		1.11E-05	0.301335251
cg22306928	MIR375	0.000791389	0.301332064
cg00357532	NCKAP1;NCKAP1	5.59E-05	0.301316139
cg07162571	ANK1;ANK1;ANK1;ANK1;ANK1	0.002304201	0.301309912
cg19499361	C12orf39	7.23E-05	0.301308561
cg15912390	C3orf21	1.11E-05	0.301305933
cg14880499	FAM123A;FAM123A;FAM123A;FAM123A	0.000357885	0.30130165
cg18233405	TSPYL5;TSPYL5	0.006823935	0.3012975
cg20977448	GRPEL2	9.24E-05	0.301296002
cg16270526	ISL1	8.60E-06	0.301281781
cg07348009	PRDM16;FLJ42875;PRDM16;FLJ42875	0.000357885	0.3012728
cg08392199	LIFR;LIFR	0.000537905	0.301263129
cg10367244		0.000187194	0.30124665
cg19344013	MYO5B	1.96E-05	0.301237087
cg25304107	HOXD4	3.34E-05	0.301223879
cg20561716	CAMK2B;CAMK2B;CAMK2B;CAMK2B;CAMK2B	0.001639597	0.30120993
cg03504245		0.000148457	0.301191758
cg14944514	CADM1;CADM1;CADM1;CADM1	9.24E-05	0.301169449
cg13845439	CDH2	9.24E-05	0.301167098
cg02008154	TBX20;TBX20;TBX20;TBX20	2.58E-05	0.301151554
cg25828963	FNDC3A	0.00043997	0.301147972
cg04420889	SGK2	2.58E-05	0.301134738
cg10656742	TNFRSF19;TNFRSF19	0.000955793	0.301131903
cg01649773	FIBIN;FIBIN	0.000117986	0.301101369
cg19770281		0.003189589	0.30108285
cg12176783	TCEA2;TCEA2	0.00043997	0.301080199
cg18725076	CTNND2	0.001376172	0.301067482
cg12424088		1.11E-05	0.301062969
cg15778232	PHB2;PHB2;EMG1	0.000791389	0.301059181
cg13775005	INSM2	3.34E-05	0.301057051
cg01645998	SDR42E1	1.96E-05	0.301053019
cg00073837		0.000955793	0.301046683
cg09100013	UNC5C	0.000148457	0.301039083
cg25523538	MKX	0.000117986	0.301028492
cg11965311	LOC145845	0.00043997	0.301027665
cg20497803	FAM83B	7.23E-05	0.301013277
cg15990972		1.96E-05	0.300980965
cg19360104	TDRD10;TDRD10;TDRD10;TDRD10;SHE	0.000537905	0.300954925
cg21184256		0.00043997	0.30095294
cg09484032		0.000357885	0.300933947
cg06958563	LOC645323	0.0059649	0.300924925
cg01362243		3.34E-05	0.300922484
cg05895618	CSRP3;CSRP3	0.000290372	0.300907207
cg24546155		0.001376172	0.300896496

cg00325531		0.000537905	0.30086786
cg01590338		5.59E-05	0.300856002
cg08224785	CYFIP2;CYFIP2;CYFIP2	0.000187194	0.300850395
cg11125104	NCRNA00164;MIR663B	0.00043997	0.300842191
cg01559726	CACNA2D3	0.000290372	0.300838502
cg11900393	NINJ2	4.34E-05	0.300832501
cg13774792	IGSF9B	9.24E-05	0.300829708
cg22040627	SLC13A5;SLC13A5	3.34E-05	0.300829231
cg23053525	DNAH17	0.001147425	0.300825563
cg04464946	PTPRR;PTPRR	3.34E-05	0.300821401
cg02922035		0.000233422	0.300820197
cg14355134		5.59E-05	0.300799916
cg03052128		7.23E-05	0.300782176
cg10185424	CD180	0.001376172	0.300752598
cg09586183	TLX1;TLX1NB	4.34E-05	0.30075221
cg11191210	VGLL2;VGLL2	4.34E-05	0.300729286
cg12681727	COL6A3;COL6A3;COL6A3	0.000187194	0.300704836
cg06257708	FADS6	0.000654035	0.300698718
cg03670452		0.001376172	0.300694453
cg00292135	C7orf13;RNF32	0.001639597	0.300688713
cg06722216	NOL4	3.34E-05	0.300683025
cg02978184	ADCY5	0.000334462	0.300679582
cg03566418	C8orf48	0.000148457	0.300678275
cg26518861		1.50E-05	0.300661034
cg03513163	PCDHB1	0.000187194	0.300642158
cg07167192	CDC42BPB	0.000148457	0.300639319
cg11721610	KIF14	0.001948248	0.30058626
cg13185413	RNF39;RNF39	0.009066563	0.300582581
cg05361489		0.003189589	0.300577017
cg12457040	KCNIP3;KCNIP3	0.002714607	0.300576261
cg20207911	RCVRN	0.000148457	0.300557554
cg09362335		0.000537905	0.300529653
cg02570354	OSBPL10	0.000791389	0.300525659
cg07520752	SAMD5	5.59E-05	0.300518514
cg21154627	SLC7A14	3.34E-05	0.300510255
cg01870826	LOC389458	4.34E-05	0.300504508
cg05048976	WNT5A	0.000148457	0.300497989
cg19342782	HHLA3;ANKRD13C;HHLA3;HHLA3;HHLA3	0.001376172	0.300493522
cg17815049	CNST;TFB2M;CNST	0.000791389	0.300492587
cg14317639		0.001147425	0.300489547
cg05600740	B4GALNT1	0.00043997	0.300483697
cg07291445		5.59E-05	0.300475557
cg03942265	CSPG4	0.000233422	0.30046467
cg26929536	SCG3;SCG3	0.000148457	0.300390108
cg22022798		0.000357885	0.300388577
cg02005147	HHIPL1;HHIPL1	0.000537905	0.300387713
cg18447727	SLITRK3	0.000187194	0.300387256
cg13285174	OXT	0.000357885	0.300371199
cg09200738	FAM7A2;FAM7A1	0.000117986	0.300369797
cg26653360	WDR93;PEX11A	0.000357885	0.300368326
cg14677983	CARS2	1.50E-05	0.300362844
cg00063773	PLS1	0.000187194	0.300352334
cg10034364		0.000357885	0.300333343
cg01366419	WBSCR17	0.001147425	0.300314599
cg02340083		0.000117986	0.300303976
cg24202381		0.000290372	0.300298135
cg11702639		0.002714607	0.300273123
cg11632238		7.23E-05	0.300270137
cg17481903		0.000187194	0.300269792
cg13718729	GRIN1;GRIN1;GRIN1	0.000791389	0.300255632

cg00255368	ESRRG	1.50E-05	0.300245995
cg13036855	CHDH;IL17RB	0.000955793	0.30023343
cg23663760	WNT3A	7.23E-05	0.300217068
cg06271087	MAGI1;MAGI1	1.11E-05	0.300216576
cg12856183		9.24E-05	0.300215629
cg02185666	NRP2;NRP2;NRP2;NRP2;NRP2;NRP2	7.23E-05	0.300208975
cg24674368		0.000148457	0.300205475
cg18722841	PHOX2A	0.000148457	0.300198076
cg20800509	RIMS2;RIMS2	0.000791389	0.300195091
cg26828839	ANO2	0.000117986	0.300189408
cg15681239	DLEC1;DLEC1	0.001948248	0.300180641
cg13306164	HPCA	5.11E-05	0.30016653
cg12063580	PACS2;PACS2	2.58E-05	0.300153725
cg10066188	NAT8B;NAT8B	0.009066563	0.300140353
cg09540612	C20orf135	0.004357816	0.300120123
cg08920068	LOX	0.000233422	0.30011789
cg19025461	FLJ37453	0.001639597	0.30010588
cg17758363		0.000791389	0.300073965
cg04180086	IRX4	0.000117986	0.300065687
cg26779676		3.34E-05	0.300045713
cg24371033	AGXT	0.000290372	0.300045097
cg21624359	FFAR3	4.34E-05	0.300031811
cg02913882		0.001376172	0.300028348
cg25973534	ADAMTS16	0.000537905	0.300026086
cg26438325	JAZF1	5.59E-05	0.300025179
cg08538258	POFUT2;POFUT2;LOC642852;POFUT2	0.000654035	0.300020316
cg00074184	SLC22A15	0.000791389	0.300010355
cg03006077	TGFBR3	0.000290372	-0.300060759
cg08495433	CLIP2;CLIP2	0.000357885	-0.300130601
cg18564075	MED13L	0.005897668	-0.300202856
cg24460268	INTS6;INTS6	0.009066563	-0.300250379
cg03818303	KCNMB3;KCNMB3	0.000148457	-0.300255386
cg11606444	SORL1	1.96E-05	-0.300310675
cg24102222	SLC15A4	0.000357885	-0.30031323
cg14962007		0.000187194	-0.30038628
cg17855390		0.006823935	-0.300393356
cg06800849	ACSF3;ACSF3;ACSF3	0.001376172	-0.300399528
cg21399778	PASK	0.000290372	-0.300417153
cg12906963		0.002304201	-0.300456798
cg19438750	SV2B;SV2B	0.00043997	-0.300613999
cg18275512	ATP2B2;ATP2B2	0.000117986	-0.3007299
cg13876325	PIGL	9.24E-05	-0.300757338
cg14575854	GRB7;GRB7	0.000791389	-0.300797802
cg07855575		0.000357885	-0.300823624
cg13571479	MAD1L1;MAD1L1;MAD1L1	7.23E-05	-0.300843119
cg12477050	ATP10B	0.000955793	-0.300905654
cg26452771	ODZ4	0.0050758	-0.300910953
cg17349406	ADIG;ADIG	8.60E-06	-0.300931747
cg14613228		0.001376172	-0.300949577
cg02692177		0.000290372	-0.30101827
cg17220749	GALNT2	0.000290372	-0.301029436
cg04554195	FPR2;FPR2	0.00043997	-0.301042006
cg10853543	BCL11A;BCL11A;BCL11A	0.0050758	-0.30105399
cg15407213	COL11A2;COL11A2;COL11A2	0.0059649	-0.301070837
cg16124188		3.34E-05	-0.301097628
cg06621027	CTNNA2;CTNNA2	1.50E-05	-0.30120396
cg19262563	SEMA3C	0.001691157	-0.301267892
cg07826859	MYO1G	8.60E-06	-0.301282236
cg18265249	TCF7L2;TCF7L2;TCF7L2;TCF7L2;TCF7L2;TCF	0.007873393	-0.301302787
cg27301488		0.000654035	-0.301303456

cg02397552	CLN5	0.001376172	-0.301309086
cg17224596		7.23E-05	-0.301356906
cg19264571	APCDD1	0.007873393	-0.301417319
cg24581650	ERGIC1	0.003734675	-0.30142701
cg19346632		0.000955793	-0.301454564
cg19060120	RPTOR;RPTOR	0.000117986	-0.301495686
cg10304534	ABCC4;ABCC4	0.000357885	-0.301615439
cg17475733		0.000791389	-0.301722908
cg01716474	DST;DST;DST;DST	0.000537905	-0.301750581
cg24166100		9.24E-05	-0.301771342
cg00786237		0.003189589	-0.301816252
cg13879523	PDE9A;PDE9A;PDE9A;PDE9A;PDE9A;PDE9A;I	0.001948248	-0.301836767
cg23295629	PVT1	1.96E-05	-0.301873778
cg11145776	SLC6A12;SLC6A12;SLC6A12	0.003734675	-0.302022668
cg14168713	BICC1	0.00043997	-0.302178835
cg00438026	ATP6V1G2;NFKBIL1;NFKBIL1;ATP6V1G2	0.000290372	-0.302179934
cg01148127	SLC43A2	0.002714607	-0.302263468
cg02379856	CAMK2D;CAMK2D;CAMK2D;CAMK2D;CAMK2I	0.003189589	-0.302363712
cg10068572	CCNY;CCNY	0.000187194	-0.302398836
cg24076588	KLHDC7A	0.003734675	-0.302475932
cg17380943	FARP1	0.003734675	-0.302479837
cg26117521	EBF1	0.000233422	-0.302508293
cg27380758	SORBS3	9.24E-05	-0.302554357
cg03586240	MAD1L1;MAD1L1;MAD1L1	4.34E-05	-0.302569048
cg19009471		7.23E-05	-0.302643355
cg01179256	CHST11	4.34E-05	-0.302661864
cg13506158	UTS2D;CCDC50;CCDC50	0.00043997	-0.302669829
cg26048923	TRAPPC9;TRAPPC9	0.000148457	-0.302716541
cg00074771	WDR76;WDR76	0.005897668	-0.302721591
cg07401516		0.004357816	-0.302916945
cg06848801		0.000654035	-0.302919944
cg19835128		0.000233422	-0.302940195
cg15159987	CPAMD8	0.00043997	-0.303004696
cg25302603	PCNXL3	0.000233422	-0.303046684
cg15213650	KDM4B	0.007873393	-0.303046702
cg00812770		0.000290372	-0.303052108
cg12620645		4.34E-05	-0.303065827
cg06869454		0.000654035	-0.303082403
cg03548512	C10orf104	3.34E-05	-0.30313153
cg10722267		0.001147425	-0.303146245
cg24897506		0.001639597	-0.303252272
cg19375114		3.34E-05	-0.303291946
cg26236922	LAMA5	4.34E-05	-0.303322944
cg14435659		0.003189589	-0.303341238
cg20820876		2.58E-05	-0.303343213
cg24214471	BLCAP;BLCAP;BLCAP;BLCAP;BLCAP	0.001639597	-0.303346826
cg04095257	C7orf50;MIR339;C7orf50;C7orf50	0.003189589	-0.303395455
cg19081470		0.000955793	-0.303402812
cg03625953	SMG6;SMG6	0.001639597	-0.303448863
cg20731819	ADARB2	0.00156564	-0.303486159
cg13629783		0.000148457	-0.303546826
cg02449762		0.000117986	-0.303584068
cg08219486	RPTOR;RPTOR	0.003189589	-0.303596526
cg01838965		0.001147425	-0.303609153
cg10859192	ATP5G2	0.00043997	-0.303613777
cg01598733	ILDR1	0.009066563	-0.303622671
cg09271709	NRXN1;NRXN1	0.00043997	-0.303623197
cg16732367	RPTOR;RPTOR	0.0050758	-0.303678956
cg26331247	LOC284805	0.000148457	-0.303721774
cg00855461	SIPA1L2	0.009066563	-0.303751216

cg07266910	ZMAT3;ZMAT3	5.59E-05	-0.303772874
cg21052932	ABHD12B;ABHD12B	1.96E-05	-0.303795116
cg07701567	GABRD	0.000654035	-0.303858853
cg26106921		0.000148457	-0.303948208
cg11067179	CD248	0.003734675	-0.30404708
cg11838898	ARHGEF4;ARHGEF4	0.002304201	-0.304101358
cg16545743	IL3	3.34E-05	-0.304156607
cg13254586		0.002714607	-0.30424207
cg11564805		0.004357816	-0.304250923
cg26603047	FAM38A	0.000187194	-0.304273036
cg19750643	TSPAN18	0.000955793	-0.304353419
cg00621943	LY6G5C	0.001948248	-0.304381581
cg04160876	MYH11;MYH11;MYH11;MYH11	0.002304201	-0.30442374
cg18998365	NR3C1;NR3C1;NR3C1;NR3C1;NR3C1;NR3C1;	0.000791389	-0.304428808
cg07833467	KLHDC7B;KLHDC7B	0.000187194	-0.304446603
cg07938480	OR1F2P	0.000187194	-0.304466358
cg19326100	TMCO3	0.001639597	-0.304574823
cg14116129		9.24E-05	-0.304587275
cg14136214	DAB2IP	0.0050758	-0.30459402
cg06755296		0.002714607	-0.304708373
cg18184935	STAC2	0.009066563	-0.304712114
cg15751131	SLC37A3;SLC37A3	3.92E-05	-0.304727396
cg09019347	TRPV1;TRPV1;TRPV1;TRPV1	0.000955793	-0.304736946
cg08699270	LRP1	0.000290372	-0.304764189
cg12377401		0.003734675	-0.304771296
cg02167757	SIAH1;SIAH1	0.001376172	-0.304773944
cg00359395	HSPG2	0.003734675	-0.304784889
cg16051561	CTNND2	0.002304201	-0.304822237
cg03737424		0.001639597	-0.304822269
cg01403532	LOC652276	9.24E-05	-0.304826835
cg19426625	ASAP2;ASAP2	0.003189589	-0.304834803
cg23433118		0.009066563	-0.304879521
cg11203293		0.001147425	-0.304972643
cg04355222	CD81	0.005897668	-0.304996254
cg18825221	RAD51L1;RAD51L1;RAD51L1	3.34E-05	-0.304998802
cg24074448	KLHL26	0.000654035	-0.305002247
cg18291664	PRKAR1B;PRKAR1B;PRKAR1B;PRKAR1B;PRK	0.001639597	-0.305046406
cg05075308	IRF2	0.002304201	-0.305052946
cg22546748		4.34E-05	-0.305086908
cg17419731	MIR589;FBXL18	0.00043997	-0.305097874
cg27285056	NAPSA;NAPSA	0.002304201	-0.305110245
cg08753560		0.003734675	-0.305163441
cg23679982	SMYD4	0.000357885	-0.305176341
cg07103517	IKZF1	0.000148457	-0.305178167
cg07893474	C3orf43	0.000148457	-0.30523413
cg14391240		0.000537905	-0.30527117
cg17088007	KCTD17	0.000654035	-0.305284137
cg09577144	DNAH17	4.34E-05	-0.305320929
cg27462475	DOCK9;DOCK9	0.002714607	-0.305337506
cg26910870	PRKCZ;PRKCZ;PRKCZ	0.000791389	-0.305452095
cg06720722	TP63;TP63;TP63	0.003486652	-0.305511807
cg05725940	GSDMB;GSDMB;GSDMB;GSDMB	4.34E-05	-0.305513202
cg07570113	LOC644649	0.000148457	-0.305514133
cg12041266	ARRB1;ARRB1	0.000233422	-0.305525701
cg14969094		1.96E-05	-0.305529209
cg04624413	NKX6-3	0.00043997	-0.305596942
cg12571629		0.000290372	-0.30563581
cg17545652	PFKFB3;PFKFB3	1.50E-05	-0.305691618
cg03974193	MAP4K4;MAP4K4;MAP4K4	0.003734675	-0.305771117
cg08489478	ZFYVE27;ZFYVE27;ZFYVE27	0.002304201	-0.305793855

cg27457191	PHTF2;PHTF2	0.001639597	-0.305810651
cg22311403	MLLT4;MLLT4;MLLT4	0.005897668	-0.305814629
cg02082273	AMPD3	7.23E-05	-0.305964364
cg04872675		0.000187194	-0.305972804
cg06889348		0.000187194	-0.306003317
cg07431973	FRMD6;FRMD6	0.000654035	-0.306076755
cg13691961	DUSP6;DUSP6	9.24E-05	-0.306115073
cg20460514	LPIN1	0.000117986	-0.306158354
cg06538771	TRABD	0.005897668	-0.306214586
cg12355172	ACY3	0.000187194	-0.306263387
cg27223728		0.001639597	-0.306354821
cg18690282	TRIO	0.000955793	-0.306355529
cg18515868	MAEA;MAEA	0.001376172	-0.306370834
cg22717593		0.000117986	-0.306394175
cg02343604	ID3	7.23E-05	-0.306571257
cg19103609	PKN1	0.001147425	-0.3065821
cg17501982	NCK2;NCK2	0.000791389	-0.306614537
cg23804842	ZNF705A;ZNF705A	0.006823935	-0.306631776
cg09199562		0.001376172	-0.306682802
cg09100014	IRF8	0.000148457	-0.306695614
cg11267546	HOXA3	0.004357816	-0.306751027
cg18773597		1.50E-05	-0.306828637
cg07974504	SPEG	0.001376172	-0.306850644
cg17934775	CHD2;CHD2	3.34E-05	-0.306893732
cg05213414		0.000955793	-0.306958173
cg15806569		0.000791389	-0.307024059
cg11042320	PDGFRB	0.000791389	-0.307027702
cg15055553	FBXL14	0.003734675	-0.307144453
cg03309308		4.34E-05	-0.30727165
cg14943796	BAHCC1	0.001147425	-0.307286881
cg10356204	JSRP1	0.000117986	-0.307295301
cg07219303	ADH6;ADH6	0.005897668	-0.30735576
cg21111416	HLTF;HLTF	0.005897668	-0.307356179
cg00018184	S100A3	0.000148457	-0.307369645
cg23627828		2.58E-05	-0.307374975
cg16774528	NIN;NIN;NIN;NIN	0.0050758	-0.30737535
cg18406924	RPTOR;RPTOR	0.000357885	-0.307379037
cg21055910	ELFN2	0.000290372	-0.307491711
cg14554017	CCDC40	0.001376172	-0.30755871
cg00181849	LMF1	0.005897668	-0.307612181
cg06330289		0.004357816	-0.307653419
cg24255327	ARHGEF10	0.00043997	-0.307670634
cg06710694	AP1B1;AP1B1;SNORD125;AP1B1	0.000955793	-0.307717796
cg20655334		0.006823935	-0.307815835
cg10246448	ATP8B2;ATP8B2	0.00043997	-0.307860916
cg15059065	NR2F6	0.003189589	-0.307868932
cg04085571	LSP1	0.009066563	-0.307899706
cg06398881		0.000290372	-0.308014982
cg04233537		0.00043997	-0.308083167
cg10825847	CASP5;CASP5;CASP5;CASP5;CASP5;CASP5	0.000654035	-0.308087497
cg02484343	E4F1	0.007873393	-0.308126398
cg17658568	LOC399959	0.001948248	-0.308154531
cg10582860		4.34E-05	-0.30840433
cg27175112		0.002714607	-0.308561794
cg01837846	KLHL29	1.50E-05	-0.308608961
cg17269581	TNFRSF13B	0.000654035	-0.308831656
cg07757358	LPP;FLJ42393	0.003189589	-0.308843614
cg17320378	FAM129C	2.58E-05	-0.308865378
cg02208504	ATP10A	0.000233422	-0.308900617
cg26564960		0.000791389	-0.308959821

cg08092966		0.009066563	-0.308994601
cg24141382	CTPS	9.24E-05	-0.309069178
cg06767142	MACROD1	0.005897668	-0.309110157
cg24372256	UMODL1;UMODL1;C21orf128	0.000187194	-0.309147265
cg03109660	RELL1;RELL1	0.000187194	-0.309147519
cg25279613	OSBPL3;OSBPL3;OSBPL3;OSBPL3	0.000290372	-0.309156395
cg26169081	CAMK1D;CAMK1D	5.59E-05	-0.309184083
cg15137213	VPS53;VPS53	9.24E-05	-0.309315205
cg13305415	SPRY4;SPRY4	0.000148457	-0.309327754
cg19046189	HSD17B14	5.59E-05	-0.309365364
cg21747070		0.000148457	-0.309378997
cg16144314	ADARB2	0.000791389	-0.30940734
cg01028142	CMPK2	0.000537905	-0.309452342
cg22876356	ELK3	0.002304201	-0.309483038
cg09463220		0.000955793	-0.309506198
cg04020713	TENC1;TENC1;TENC1	0.000233422	-0.309509094
cg10169812	CD38	0.003189589	-0.309525271
cg07876162	PALM;PALM	0.000148457	-0.309567032
cg01104590	CARS;CARS;CARS;CARS	1.50E-05	-0.30960667
cg11950754	LRP8;LRP8;LRP8;LRP8	0.001147425	-0.309639378
cg03797660	CBFA2T3	0.000148457	-0.309645852
cg17994788	C1GALT1	0.002714607	-0.309669752
cg15345743	RASSF3	0.002304201	-0.309746566
cg00987918	TRIM40	3.34E-05	-0.309762496
cg04714939	ZMIZ1	0.002714607	-0.309785801
cg10231179	DENND3	0.000791389	-0.30984967
cg09946870	DENND3	0.0050758	-0.309889691
cg10730435		9.24E-05	-0.309895879
cg25332502	MBP;MBP	0.000117986	-0.30991173
cg03670238	WISP1;WISP1	0.000955793	-0.309956234
cg23732024	LY96	9.24E-05	-0.309990213
cg09026568	TRABD	0.000187194	-0.309997809
cg13461482	TCIRG1;TCIRG1	7.23E-05	-0.310024197
cg12936469	C6orf59;AGPAT4	0.000654035	-0.310071634
cg27175380		4.34E-05	-0.310112111
cg04719721		0.000148457	-0.31015534
cg26974444	SNX29	0.001376172	-0.31017955
cg03661324		0.000537905	-0.310209061
cg01113811	MAML2	0.00043997	-0.310209373
cg17516997		0.000290372	-0.310246854
cg05321594		0.001639597	-0.310253526
cg25916172		2.58E-05	-0.310268992
cg05223100	LHCGR	0.000791389	-0.310297254
cg26394380	SFTPB;SFTPB	4.34E-05	-0.31031293
cg00779313	WDR37	0.002714607	-0.310341767
cg08730070	COMT;COMT;COMT	0.004357816	-0.310375077
cg00511632	PXDN	0.003189589	-0.310403771
cg06649165	MAP1S	0.000117986	-0.310406301
cg07437546		5.59E-05	-0.310446094
cg16782719	SLC15A4	0.003734675	-0.310501659
cg04670451	LOC100129637	0.009066563	-0.310504809
cg17884674	CSNK1G2	8.60E-06	-0.310505447
cg15773261	GPR133	0.000357885	-0.31052929
cg16201957	MAML3	1.11E-05	-0.310567788
cg19903805	TC2N;TC2N	4.34E-05	-0.310580497
cg18651347	AUTS2;AUTS2	0.005897668	-0.310585678
cg13366849	DNM2;DNM2;DNM2;DNM2	0.001639597	-0.310644102
cg21964662	RNF219	0.003734675	-0.310658106
cg00562504	DIP2C	0.000537905	-0.310673104
cg22439922		0.001376172	-0.310679137

cg14592928	ANGPT4	0.001376172	-0.31068823
cg04685632	KCNQ2;KCNQ2;KCNQ2;KCNQ2;KCNQ2	0.000791389	-0.310815119
cg04372535	AP2A2	0.007873393	-0.310825778
cg25775449	LTB4R;LTB4R;CIDEB	7.23E-05	-0.310854582
cg13601957		0.000117986	-0.310981539
cg09918634		0.000187194	-0.311145376
cg24448870	CHCHD6	0.000290372	-0.311150482
cg18659586	ASGR2;ASGR2;ASGR2;ASGR2	2.58E-05	-0.311205482
cg21320573	UNC5D	0.001639597	-0.311216069
cg04225551	GRHL2	0.000654035	-0.311288664
cg20145598	SGTA	0.00043997	-0.311297269
cg21172497	PCSK5	0.000148457	-0.311331174
cg00271311	CNTF;ZFP91-CNTF	4.34E-05	-0.311340731
cg05861879	NUDT1;NUDT1;NUDT1;NUDT1;NUDT1;NUDT1	0.000233422	-0.311343474
cg23811811	BMP2K;BMP2K	0.00043997	-0.311349334
cg00566158	PTPRN2;PTPRN2;PTPRN2	4.34E-05	-0.311401766
cg23280720	HECA	4.34E-05	-0.311477332
cg00860359	PANX2;PANX2;PANX2	0.00043997	-0.31148096
cg11076814		0.000537905	-0.311507203
cg26192520	YWHAZ;YWHAZ;YWHAZ;YWHAZ;YWHAZ;YWH	0.003189589	-0.311522577
cg02450785		0.001639597	-0.311548688
cg27550441	PARN;PARN	5.59E-05	-0.311611206
cg04887078	ADARB2	0.001639597	-0.311611522
cg26785823	LOC115110	4.34E-05	-0.311680434
cg12002047	FCER2	9.24E-05	-0.311697153
cg08367573		0.000187194	-0.31173788
cg24919348	MIR599;VPS13B;VPS13B;MIR875	0.000148457	-0.311751794
cg20702417	DNMT3A;DNMT3A;DNMT3A	0.000148457	-0.311844213
cg16453673	APPL2	0.006823935	-0.311867992
cg01503450		0.002304201	-0.31186943
cg17018896	MAD1L1;MAD1L1;MAD1L1	0.001376172	-0.311871655
cg08320989		0.001376172	-0.311881659
cg20914792		2.08E-05	-0.311901954
cg08949143	LOC100188949	2.58E-05	-0.311980984
cg04398180	ADPRHL1;ADPRHL1;ADPRHL1	9.24E-05	-0.312021958
cg17396638	FURIN	0.00043997	-0.312079333
cg20495365	RGS12;RGS12;RGS12	7.23E-05	-0.312104318
cg22634633	SUNC1;SUNC1	0.006823935	-0.312140448
cg16616918	SMYD4	0.000187194	-0.312178768
cg04609875	KCNMB4	5.59E-05	-0.312188686
cg27551910	BAIAP2;BAIAP2;BAIAP2;BAIAP2	4.34E-05	-0.312261679
cg10039504	TSPAN9;TSPAN9	0.001948248	-0.312277869
cg00113315	SLC2A5;SLC2A5	0.000955793	-0.312357528
cg01484915	RASSF4	0.000290372	-0.312455143
cg04195684	MLC1;MLC1	0.00043997	-0.312459098
cg24581795	TNFRSF10B;TNFRSF10B;TNFRSF10B	0.000654035	-0.312546276
cg25551622	CACNG1	0.001147425	-0.312616351
cg00864684		0.000537905	-0.312808949
cg10753966	ERC2	0.004357816	-0.312818039
cg14711743	SERINC5	0.000537905	-0.312822423
cg19274618		0.0050758	-0.312866842
cg04861974		0.000357885	-0.312880002
cg00648660	RPTOR;RPTOR	0.0050758	-0.312913517
cg00478326		7.23E-05	-0.312991686
cg26415789	SEPT9;SEPT9;SEPT9;SEPT9;SEPT9;SEPT9;SI	9.24E-05	-0.313035714
cg04466743	RIN2	0.000187194	-0.313070236
cg04677846	C21orf33;C21orf33	0.000537905	-0.313122107
cg06617335	LOC91450	0.000187194	-0.313146196
cg02211735	OR52A4	0.000290372	-0.31315112
cg09323092	LMF1	0.007873393	-0.313177868

cg14600368		0.000537905	-0.313184959
cg16318349	PBXIP1	0.001948248	-0.313215071
cg07155381	RPH3AL	0.006823935	-0.313255015
cg04339790	EMP2	0.001639597	-0.313255304
cg18300008		0.000537905	-0.313280456
cg00706994	OR5A2	6.94E-05	-0.313280593
cg07376029	GC	9.24E-05	-0.313285769
cg14537247	DENND1A;DENND1A	0.005897668	-0.313321638
cg09197783	SLC43A3;SLC43A3;SLC43A3	0.001948248	-0.313331583
cg23786545		0.009066563	-0.313423531
cg09947611	HCCA2	0.004357816	-0.313425576
cg22361816	COL11A2;COL11A2;COL11A2	4.34E-05	-0.31345121
cg06601266	C10orf119	1.50E-05	-0.313477746
cg11456838	OSTCL	0.000357885	-0.313523002
cg11955768		7.23E-05	-0.313575911
cg26244225	APOLD1;APOLD1	2.58E-05	-0.313609592
cg25256099		3.34E-05	-0.313616476
cg17168630	PLAUR;PLAUR;PLAUR	0.000791389	-0.313627125
cg16067628	AATK	0.006823935	-0.313679597
cg20748242	RNF44	0.005897668	-0.313698539
cg00391031	KIAA1274	0.000654035	-0.313710156
cg09811510	SCHIP1	0.001948248	-0.313725018
cg12325605	ARHGEF3;ARHGEF3;ARHGEF3	2.58E-05	-0.313800559
cg08796342	TC2N	0.000791389	-0.313860339
cg06640997		0.006823935	-0.313887607
cg13361558	PLEKHB1;PLEKHB1;PLEKHB1;PLEKHB1;PLEK	7.23E-05	-0.313998356
cg08289350	CYGB	1.96E-05	-0.314099059
cg06935608		0.000233422	-0.314118432
cg12491115	APLP2;APLP2;APLP2;APLP2;APLP2;APLP2	7.23E-05	-0.314129636
cg09166898	RHOV	0.004357816	-0.314145018
cg00334274		0.003189589	-0.314173008
cg16834011	COMT	0.001948248	-0.314216846
cg21959598	VOPP1	0.003734675	-0.314233926
cg05995576		0.000654035	-0.314251995
cg11706849	MGAT5	0.000537905	-0.314408557
cg15903956		0.000233422	-0.314422476
cg02640147	KCNIP1;KCNIP1;KCNIP1	0.001376172	-0.314472736
cg10842314	SH3TC1	9.24E-05	-0.314475584
cg14381712	VPS13D;VPS13D	0.006823935	-0.31454433
cg14149774	FAM69B	0.001376172	-0.314564611
cg21940655	MAD1L1;MAD1L1;MAD1L1	0.00043997	-0.314638244
cg09221159		0.000148457	-0.314656962
cg25913761	SEMA4B	0.000148457	-0.314708585
cg03602500	KIR3DX1	0.001147425	-0.31482979
cg05831188	SDK1	0.000187194	-0.314833386
cg05992079	SIT1	1.96E-05	-0.314841864
cg25548350		0.003189589	-0.315002021
cg15549075	PRKCE	0.000654035	-0.315003015
cg06097077	NLGN1	0.001147425	-0.315020942
cg20000908	MEGF6	0.000654035	-0.31502937
cg25654695	OAZ1	0.003189589	-0.31505371
cg27124512	TRIM72;TRIM72	0.000187194	-0.315091954
cg18173263	PEMT;PEMT;PEMT	1.96E-05	-0.315128795
cg20728490	DNTT;DNTT;DNTT;DNTT	2.58E-05	-0.315192713
cg12229364	WWC2	0.000148457	-0.315248588
cg18915456		0.001147425	-0.315264252
cg13641082	SNX8	0.000654035	-0.315286425
cg04091927		0.000187194	-0.315331735
cg01092108		0.000187194	-0.315366856
cg26021007		3.34E-05	-0.315403248

cg14344864		0.000148457	-0.31542141
cg06855137	FAM38A	0.00043997	-0.315446089
cg08177126	DUSP22	0.000791389	-0.315477661
cg07433178	SYNPO;SYNPO	0.000791389	-0.315528224
cg02319782		3.34E-05	-0.315534384
cg03631294	CD79B;CD79B;CD79B	4.34E-05	-0.315556426
cg04409030	SOCS2	0.000365373	-0.315592488
cg09158821	SLC43A2	0.000187194	-0.315610499
cg11580897		0.000955793	-0.315651587
cg00471368	PLCG2	0.004357816	-0.315786469
cg00073460	ZC3H12D	3.34E-05	-0.31582099
cg03033975	GTPBP5	9.24E-05	-0.315867244
cg01615861		4.34E-05	-0.315896629
cg00870449	SLC8A1;SLC8A1;SLC8A1;SLC8A1	0.003734675	-0.315982625
cg26654771	SORBS3	0.000458355	-0.316011285
cg08287903	UGT8	0.000233422	-0.316022936
cg06517940	LCK	0.000158823	-0.316032948
cg07813254	FAM43B	0.000117986	-0.316049261
cg13300744	CUX2	0.000233422	-0.316120235
cg12551957	FBXL18	3.34E-05	-0.316147804
cg24405716	MTMR10	0.001639597	-0.316178378
cg06677021	SERINC5	4.34E-05	-0.316270809
cg14465801		5.59E-05	-0.316300565
cg00533183	PSMB8;PSMB8	0.000537905	-0.316302607
cg27043531	FLJ22536	1.50E-05	-0.316304224
cg05815436		0.000117986	-0.316321987
cg00546248	PLCH2	0.000233422	-0.31632784
cg15636295	HBEGF	0.000187194	-0.316372133
cg22478679	ADPRHL1;ADPRHL1	0.000357885	-0.316412564
cg05859076	RAB43	0.002227374	-0.316440005
cg26757820	ADK;ADK	0.000233422	-0.316525814
cg06539006	CPO	0.001639597	-0.316571668
cg18885162	FOXP4;FOXP4;FOXP4	0.000117986	-0.316635464
cg25958911	BOP1	0.003734675	-0.316889012
cg26687670	OPN3	0.003189589	-0.316904597
cg06959205	CBFA2T3;CBFA2T3	4.34E-05	-0.316908028
cg00797286		0.000791389	-0.316948608
cg23533000		1.96E-05	-0.317028161
cg08854834	C21orf7	0.001948248	-0.317054726
cg12157387	PARD3B;PARD3B;PARD3B	0.001376172	-0.317086072
cg25308562		9.24E-05	-0.317130469
cg03095773	RFX8	0.000233422	-0.317143643
cg14043104	KLF12	0.000357885	-0.317183864
cg07457727		0.003734675	-0.317222223
cg16646298	FLOT1	0.000955793	-0.317244137
cg27443224	CCL21;CCL21	0.000117986	-0.317300652
cg27557782		9.24E-05	-0.317331172
cg18941322		5.59E-05	-0.317354044
cg23413497		0.000233422	-0.317356577
cg14275281	PLCD3	4.34E-05	-0.317424748
cg08246318		0.000791389	-0.31744151
cg04641860	HNRNPF;HNRNPF;HNRNPF;HNRNPF;HNRNPI	0.003189589	-0.317460567
cg11036672	C4orf23	0.000290372	-0.317478925
cg26687842	LOC646982;LOC646982;LOC646982	3.34E-05	-0.31748004
cg21708145	LOC154449	0.004357816	-0.317510932
cg20821314	PDIA6	0.000117986	-0.317522426
cg05647602	BID;BID	0.000654035	-0.317570176
cg05892024	PDGFA;PDGFA	0.003734675	-0.317620679
cg08669954	IFT140	0.009066563	-0.31764099
cg24710671	C21orf2	0.000791389	-0.317643009

cg16150435	C6orf15	7.23E-05	-0.31764553
cg01632562		0.000791389	-0.3177404
cg05799596		0.001639597	-0.317835464
cg21901847	NOS3	0.001639597	-0.31786325
cg04637506	LRRC43;LRRC43	0.002304201	-0.317892872
cg05754179		0.004357816	-0.317927173
cg11870455		1.96E-05	-0.317937315
cg17169037	KDM2B;KDM2B	0.0050758	-0.317993301
cg02736966		0.000955793	-0.318069486
cg21484631	UHRF1;UHRF1	3.34E-05	-0.318091308
cg09989938	CD19	0.003734675	-0.318114594
cg08268075	INSC	0.000537905	-0.318117235
cg04511619	ZNF423	0.001948248	-0.318169841
cg18897335	OSBPL5;OSBPL5;OSBPL5	0.001147425	-0.318170874
cg01495363	LOC100287216;SH3RF3	0.001376172	-0.318257692
cg21969795	TMEM88	9.24E-05	-0.318261253
cg08279097	DCLRE1B	0.002304201	-0.318270105
cg09071834		0.000955793	-0.318290723
cg11408632		0.000357885	-0.318361861
cg06705947	CUX2	2.58E-05	-0.318408784
cg21448167	TSPAN9;TSPAN9	0.0050758	-0.318517744
cg06448249	CD79A;CD79A	0.001376172	-0.31869766
cg01493678	HLA-DOB	0.002714607	-0.318782399
cg19480385		4.34E-05	-0.318805839
cg11434671	CYGB	3.34E-05	-0.318838999
cg22623319	RBM20	0.000537905	-0.318889059
cg25474235	PRAGMIN	3.34E-05	-0.318969287
cg05970307	KIAA1191;KIAA1191;KIAA1191	0.000357885	-0.319077058
cg09730735	LRRC2;LRRC2	0.001639597	-0.319080224
cg02084553		0.002714607	-0.31911217
cg27067040	C6orf59;AGPAT4	0.000955793	-0.319132125
cg01099825	COBRA1	4.34E-05	-0.319169335
cg07057617		0.005897668	-0.31922397
cg13842421	GPR133	0.001376172	-0.319235455
cg07241457		0.002714607	-0.319245605
cg20592017		0.004357816	-0.319389912
cg06549275	ASAP2;ASAP2	4.34E-05	-0.319423627
cg22898082	SMARCA4;SMARCA4;SMARCA4	5.59E-05	-0.319439972
cg11659652	PTPRE	0.000955793	-0.319448299
cg05057352	XYLT1	0.001948248	-0.319518015
cg09698826	HIVEP1	0.002714607	-0.319553715
cg20698113	PIM3	0.001147425	-0.319664931
cg07213487	TRRAP	0.007873393	-0.319673646
cg16098340	MIR548C;RASSF3	0.00043997	-0.319719791
cg00599809	POM121	0.002714607	-0.319729736
cg01577029		0.000654035	-0.319813595
cg25916135		0.001147425	-0.319853088
cg07992659	BMPER	0.000791389	-0.319854133
cg19622623	RASSF9	0.000148457	-0.319886168
cg07420333	LGR5	0.000187194	-0.319900406
cg00962740	SDCCAG8	0.000537905	-0.319990977
cg00525508	ADARB2	0.00043997	-0.320032199
cg00115458	COL11A2;COL11A2;COL11A2	7.23E-05	-0.320100271
cg12421110	C13orf15	0.001376172	-0.320187533
cg24060317	CLSTN1;CLSTN1	0.002304201	-0.320208401
cg21097504		7.23E-05	-0.320237519
cg16947583	GULP1	3.34E-05	-0.320288864
cg22614400	GIGYF1	0.006823935	-0.32033866
cg21486834	RHBDF2;RHBDF2	0.0050758	-0.320387758
cg22485298	DDR1;DDR1	5.59E-05	-0.320419485

cg21627202	PLXNB2	2.58E-05	-0.320446095
cg27123351	ALDH3B2;ALDH3B2	0.002714607	-0.320522474
cg22372194		0.000290372	-0.320568099
cg25198661	ODZ4	0.000955793	-0.320570842
cg15582102		0.009066563	-0.320591454
cg06612130		4.34E-05	-0.320645398
cg06762457	ZC3H12D	7.23E-05	-0.320736748
cg26686009	C10orf25;ZNF22	4.34E-05	-0.320743466
cg00071012		0.006823935	-0.320766773
cg15775674	CAPN3;CAPN3;CAPN3;CAPN3;CAPN3;CAPN3	0.002304201	-0.320797669
cg01420564	OSBPL6	0.000290372	-0.320807802
cg22724943	MAN1C1	9.24E-05	-0.320837094
cg26341831	TMEM63A	0.00043997	-0.320843993
cg25206071	IL19;IL19	0.000187194	-0.320883746
cg18007757		0.002304201	-0.320892156
cg02796939	FOXK2	0.000187194	-0.320909848
cg01808849	MYO10	0.003734675	-0.320928579
cg18121565	RMND1	0.007873393	-0.320954632
cg03512076		0.0050758	-0.321062293
cg07906046	ADCY9	5.59E-05	-0.32106427
cg06513247	SEPT9;SEPT9;SEPT9;SEPT9;SEPT9;SEPT9;SI	1.96E-05	-0.321076044
cg10426318	KIAA1949;KIAA1949	2.58E-05	-0.321116168
cg09208331		4.34E-05	-0.321315973
cg20283221	FYTTD1;KIAA0226;FYTTD1	0.001376172	-0.321337199
cg20376123	CELSR1	2.58E-05	-0.321380368
cg01801603	ZHX2	5.59E-05	-0.321424311
cg26747517	ANKRD11	0.000654035	-0.321472148
cg22137572	CDH18;CDH18	0.000148457	-0.321546836
cg15549637	ATXN7L1;ATXN7L1	0.000955793	-0.321674399
cg26307814	PIK3CD	0.003734675	-0.321685279
cg10270895	PARD3	0.006823935	-0.321711762
cg05924882		0.000357885	-0.321792897
cg25838060		0.00043997	-0.32190377
cg05211356	ACER2	0.000290372	-0.321939095
cg19273668		0.002714607	-0.321991019
cg11479568	ANK1;ANK1;ANK1;ANK1;ANK1;ANK1;ANK1;AN	0.000233422	-0.322013431
cg20856497	CSNK1D;CSNK1D	4.34E-05	-0.322020767
cg06453088	CRMP1;CRMP1	0.000117986	-0.322055517
cg06810986		0.000654035	-0.32209961
cg04023226	CYGB;PRCD	0.000955793	-0.322134489
cg05480110	MUC4;MUC4;MUC4	0.006823935	-0.322246073
cg21476000	HOXC4	0.000955793	-0.322252562
cg11873854	LCN6	5.59E-05	-0.322256138
cg26706520		0.0050758	-0.322288706
cg22354363	FGF19	0.007873393	-0.322308561
cg13801057		0.002714607	-0.322311707
cg08679800		0.002714607	-0.322317553
cg03655684	CLCN7;CLCN7	0.009066563	-0.322423569
cg07322003		0.000654035	-0.322429409
cg27595127		0.000791389	-0.322574984
cg18814227	SESTD1	0.003189589	-0.322621438
cg07696514	IKBKB	0.000233422	-0.322702063
cg09985802	LHPP;LHPP	2.58E-05	-0.322711801
cg04503912	DSPP	0.001376172	-0.322750531
cg06343686	KLHL30	0.001147425	-0.32277472
cg24049888	POU2AF1;POU2AF1	8.60E-06	-0.322874089
cg10547908	FAM174B	0.000187194	-0.322890998
cg20594765	GALNT2	5.59E-05	-0.322900796
cg20682563	TCF7;TCF7;TCF7;TCF7;TCF7;TCF7;TCF7	9.24E-05	-0.323046153
cg24093176	PAPLN	0.000117986	-0.323159824

cg11182965	TXNRD2	4.34E-05	-0.323192296
cg23009067	GNA12	0.001147425	-0.323207612
cg23314514		0.001376172	-0.323219652
cg18158438	SLC22A11	1.96E-05	-0.323235685
cg16618789		0.000117986	-0.323525606
cg21142904	KIRREL	0.000955793	-0.323663025
cg03361664	OSBPL5;OSBPL5;OSBPL5	0.00043997	-0.323677368
cg00540464		0.000791389	-0.323681015
cg15174682	KDM4B	0.000357885	-0.323851406
cg09373727	PTP4A2;PTP4A2	0.000148457	-0.323901151
cg16652347	PLXNA4;PLXNA4;PLXNA4	0.005897668	-0.323943435
cg26973018	ZNRF1	0.000148457	-0.323991639
cg04820679	COL11A2;COL11A2;COL11A2	0.000233422	-0.324076663
cg08623942	SMURF1;SMURF1	0.001376172	-0.324139167
cg15816503	C8orf74	7.23E-05	-0.32415859
cg25597976	UBE2W;UBE2W	0.00043997	-0.324168337
cg06071246	SLC7A5	0.000290372	-0.324222544
cg01616628	BCL11A;BCL11A;BCL11A	0.000537905	-0.32428036
cg15995075	TRPC1	0.001147425	-0.324316826
cg19750657	UFM1	0.000537905	-0.324327896
cg00617975	C22orf40	0.00043997	-0.32439401
cg16432031	DHRS3	0.009066563	-0.324409043
cg15900221	ARHGEF16	0.000955793	-0.324409345
cg09432758	TCF3;TCF3	0.000148457	-0.324421623
cg06720713		0.000955793	-0.324463637
cg24863175	PLA2G4A	0.000290372	-0.324486309
cg08754149	C10orf11	0.000654035	-0.324494492
cg15487646		5.59E-05	-0.324530459
cg14341467	GRK5	0.000537905	-0.32454749
cg21908828	KIAA0922;KIAA0922	0.000537905	-0.324563523
cg07986058	ATP10A	5.59E-05	-0.324570671
cg23251296	CD79B;CD79B;CD79B	0.002266401	-0.32464857
cg01279933	ATG4D;KRI1	1.96E-05	-0.324659013
cg06021990	FLT1	9.24E-05	-0.324682243
cg15038286	GATA2;GATA2;GATA2	0.002304201	-0.324715352
cg27244242	LY6G5C	0.003189589	-0.324747041
cg17701942	STX5	0.000357885	-0.324751608
cg21831463		5.59E-05	-0.324783058
cg26471398	TRAPPC9;TRAPPC9	0.000148457	-0.324786673
cg21578050	SEMA6A	0.006823935	-0.324814269
cg27616666		0.005897668	-0.324820277
cg25136988	LY6G5C	0.001147425	-0.324857899
cg13213814	MYEOV	0.000290372	-0.324921633
cg08901339	BLNK;BLNK;BLNK;BLNK	3.34E-05	-0.324956037
cg05163268		0.004357816	-0.324976015
cg03160526	B3GNTL1	2.58E-05	-0.325016253
cg22199118	C8orf34;C8orf34	4.34E-05	-0.325056285
cg07620544	TRIM72;TRIM72	0.000233422	-0.325083413
cg24721309	ATP11A;ATP11A	0.001147425	-0.325085706
cg24033471	CACNA1C;CACNA1C;CACNA1C;CACNA1C;CA	9.24E-05	-0.325111625
cg27427054	SUV420H2	0.000654035	-0.325199437
cg21705505	C21orf33;C21orf33	0.000955793	-0.32534297
cg10829004	RTN1;RTN1	0.002714607	-0.325363053
cg24556382	GALNT7	7.23E-05	-0.325387378
cg09408937	CYFIP1	0.007873393	-0.325462947
cg18809947	PBX2	0.001639597	-0.325524338
cg26256901		9.24E-05	-0.325559022
cg23751110		0.003734675	-0.32560163
cg07324172	FBXW7;FBXW7;FBXW7	0.007873393	-0.325627876
cg02162605	C2orf85	5.59E-05	-0.325687821

cg20150640	C1orf213;C1orf213;C1orf213;ZNF436	0.000187194	-0.325730674
cg25952596		0.001948248	-0.325740037
cg16549994		0.000357885	-0.325760523
cg14569771		5.59E-05	-0.325805485
cg09314803	AZI1;AZI1	0.005897668	-0.325816437
cg20837332	KCNK7;KCNK7;KCNK7;KCNK7	0.000290372	-0.325841203
cg18551531	LSM7	0.000148457	-0.325921283
cg07262457		0.000148457	-0.325925056
cg08428949	TTBK2	4.34E-05	-0.325925603
cg05358758	KRT71	0.000290372	-0.325947909
cg15685223		0.007873393	-0.326009241
cg26808154	MORN1;LOC100129534	0.000233422	-0.32604546
cg25865542	CMTM7;CMTM7	0.000148457	-0.326075339
cg01008894	RGS12;RGS12	0.000117986	-0.326100324
cg11746924	CD79B;CD79B;CD79B	3.34E-05	-0.32621394
cg13109410	GPR44	0.001376172	-0.326225586
cg11075561		0.000791389	-0.326237457
cg19445335	ADAP1	0.001639597	-0.326356135
cg27660099	EEPD1	0.005897668	-0.326395267
cg12701674		0.000290372	-0.326564495
cg23729763	APLP2;APLP2;APLP2;APLP2;APLP2;APLP2	7.23E-05	-0.326575113
cg02947125		4.34E-05	-0.326579288
cg07803420	DDR1;DDR1	4.34E-05	-0.326652871
cg07064537	PLD1;PLD1	0.001147425	-0.326698339
cg19913465	FCER2	0.000117986	-0.326722459
cg16946840	MED12L	0.001376172	-0.326735818
cg26738160	RAPSN;RAPSN	0.002304201	-0.326858397
cg14223485	C9orf25	0.003734675	-0.326979047
cg13502395	KIAA1026;KIAA1026	0.000955793	-0.327049044
cg00061805	SQSTM1;SQSTM1;SQSTM1	0.001147425	-0.327055855
cg24947371	GPR133	0.000233422	-0.32719239
cg03048947	LBR;LBR	0.002304201	-0.32726668
cg06568490	HDAC11;HDAC11	0.000290372	-0.327323468
cg21169779	NAIF1	0.004357816	-0.327351967
cg04176122	BCL9L	2.58E-05	-0.32737302
cg26841040	GUK1;GUK1;GUK1	9.24E-05	-0.327407043
cg06156640	ALLC	0.001948248	-0.327434266
cg20739984		7.23E-05	-0.327454201
cg20595271	SH2B3	0.000791389	-0.327480974
cg01636910	BCL10	0.000654035	-0.327490609
cg19491207	SNTG2	0.000233422	-0.327511765
cg25117092	MED12L;P2RY14;P2RY14	0.002304201	-0.327513317
cg04537282		1.50E-05	-0.327516655
cg03157862		0.000290372	-0.327520215
cg08126223	RND1	0.000117986	-0.327542872
cg01239922	LRRC14B	0.00043997	-0.327561758
cg18920397	LY9;LY9	2.58E-05	-0.327566399
cg02857726	TM4SF1	0.000537905	-0.32757807
cg16014060	RAD51L1	0.000187194	-0.327677355
cg18755226	MXRA7;MXRA7;MXRA7	0.001147425	-0.327698118
cg06595162	NCRNA00114;NCRNA00114	0.003734675	-0.327841816
cg14821245	CTNNA2;CTNNA2	0.000187194	-0.327852826
cg07215697	TULP4;TULP4	0.000187194	-0.328147597
cg02003183	CDC42BPB	9.24E-05	-0.328153085
cg25152942	MIA	0.001376172	-0.328186518
cg21195376	PIK3C2B	2.58E-05	-0.328209942
cg09208942	PTGER2	0.000654035	-0.328259066
cg04704634	IL3	1.96E-05	-0.328329157
cg26733897	RPTOR;RPTOR	4.34E-05	-0.328340998
cg05932360	JARID2	1.96E-05	-0.328348196

cg09445967	ZP1	5.59E-05	-0.328435599
cg04912778	KCNMA1;KCNMA1;KCNMA1;KCNMA1	0.000290372	-0.328438942
cg13869484	SLC2A5	0.000654035	-0.328535708
cg13601739	SLCO2B1;SLCO2B1;SLCO2B1	0.000357885	-0.328543613
cg02311864	TNFRSF21	0.000357885	-0.328573887
cg03556480	IGFBP2	0.0050758	-0.328603779
cg25350722		0.001639597	-0.328606971
cg07877964	USP7	0.001376172	-0.328618894
cg26256521		2.02E-05	-0.328622616
cg11654325	C2orf85	0.000357885	-0.328627941
cg03994959	LIG4;LIG4;LIG4;LIG4	0.0050758	-0.328702503
cg15731920	LASP1	0.000290372	-0.328717394
cg09105687	MMEL1	1.96E-05	-0.32877716
cg04103532	HIVEP2	1.96E-05	-0.328819305
cg17359975	CTGF	0.007873393	-0.328856144
cg10464130	KALRN;KALRN	5.59E-05	-0.328863524
cg07996532	ECE1	2.58E-05	-0.328930362
cg15474337	NAPSB	1.96E-05	-0.329000515
cg04266474	EEPD1	0.002714607	-0.329057547
cg07236127	TNNI1	0.000955793	-0.329058259
cg26244575		0.000654035	-0.329125506
cg01205058		4.34E-05	-0.32913986
cg16530881		5.59E-05	-0.329182951
cg08830576	C16orf91	0.000187194	-0.329218453
cg27635976	CBFA2T3;CBFA2T3	0.007873393	-0.329220852
cg10481072	CDK6;CDK6	0.001948248	-0.329255588
cg01471006	FAM190B	0.001147425	-0.329495893
cg05124117		0.003189589	-0.329504156
cg13628057	EHMT1;EHMT1	0.004357816	-0.329534711
cg26003455		4.34E-05	-0.32960769
cg12781678		0.002304201	-0.329668658
cg00792968		0.009066563	-0.329679003
cg07800837		0.001948248	-0.32978521
cg13020868		0.000290372	-0.329790142
cg15630458		0.000357885	-0.329835849
cg16100721	TMEM123	2.08E-05	-0.32986595
cg14443182	DNAJB5;DNAJB5;DNAJB5	9.24E-05	-0.329868653
cg10445988	MAD1L1;MAD1L1;MAD1L1	0.000357885	-0.329884758
cg15694146		2.58E-05	-0.329906789
cg24112097	BRD1	0.001147425	-0.329953071
cg08859278	ZAP70	0.000233422	-0.329992627
cg00624799	ZNF710	0.000955793	-0.330040658
cg17004353	CARS;CARS;CARS;CARS	2.58E-05	-0.330110802
cg22526990		0.000791389	-0.330121459
cg04219504	MEF2A;MEF2A;MEF2A	0.000148457	-0.330130026
cg17815933	FOXJ2	3.34E-05	-0.330144403
cg07826255	SGCA;SGCA	7.23E-05	-0.330160445
cg20379919	THPO	5.59E-05	-0.330167633
cg21435957	FLOT1	0.00043997	-0.330218397
cg08842021	RUNX2;RUNX2;RUNX2	0.003189589	-0.330233062
cg02073763	PRKCE	5.59E-05	-0.330329279
cg14694744	XIRP1	0.006823935	-0.330329626
cg01176694		0.000187194	-0.330351427
cg14174320	PC;PC;PC	0.000365373	-0.330355149
cg12577411	DTNBP1;DTNBP1;DTNBP1	4.34E-05	-0.330361928
cg26479022		5.59E-05	-0.330379849
cg13586425	GPR133	0.000148457	-0.330470284
cg06678005		0.000117986	-0.330526736
cg10239022	GPR133	9.24E-05	-0.330539469
cg11601443	OAS2;OAS2;OAS2	9.24E-05	-0.330642046

cg17701035	CLEC17A	0.000955793	-0.330704767
cg06770731	SLC7A5	0.000537905	-0.330739897
cg03270969	C2orf85	4.34E-05	-0.330750039
cg05921138	SORL1	0.001147425	-0.330760804
cg06821871	CBFA2T3;CBFA2T3	0.001147425	-0.330782092
cg17638507		3.34E-05	-0.330789695
cg04776489	VPREB1	5.59E-05	-0.330873944
cg07918080		0.000357885	-0.330939791
cg03372385	LRRC16A	0.000955793	-0.331036014
cg03990819		0.001948248	-0.331045912
cg02703190	FAM172A;FAM172A;FAM172A;FAM172A	0.007873393	-0.331133046
cg25315503		0.000187194	-0.331135744
cg10985158	NAV1	0.00043997	-0.331252204
cg09526469	TRIO	0.005897668	-0.331365251
cg20277740	NPHP4	0.001147425	-0.331376829
cg01192051	POLG;POLG	0.000357885	-0.331392685
cg00912580	MGAT5	1.96E-05	-0.331467046
cg19478079		0.000148457	-0.331497715
cg15602677	SNTG2	0.000290372	-0.331519619
cg18880986	FBXL18	0.000233422	-0.331542079
cg21028142	NPLOC4	0.003189589	-0.331880145
cg01373166	ADORA2A	8.60E-06	-0.331910439
cg00461022	FAM134B	0.000537905	-0.331976107
cg05703230		0.000290372	-0.332067017
cg07403350	LIG4;LIG4;LIG4;LIG4	0.005897668	-0.332080349
cg11944933	FAM167A	0.001639597	-0.332080394
cg13861536	KIAA1614	1.96E-05	-0.332093881
cg13271206	RPS3	0.002304201	-0.332156954
cg19222784	NAV2;NAV2;NAV2;NAV2	0.000233422	-0.332201234
cg00499539	PSD4;LOC440839	9.24E-05	-0.332208072
cg01764370		0.005897668	-0.332308556
cg23793965	NECAB1	4.34E-05	-0.332450362
cg01964170		0.002450672	-0.332481811
cg17742617	PPM1H	0.00043997	-0.332528743
cg24153714	C10orf11	0.000290372	-0.332539577
cg05515866	FARP2	0.000537905	-0.332625131
cg06559756	PITPNC1;PITPNC1	5.59E-05	-0.332824335
cg18499396		0.000537905	-0.332870188
cg08364772		0.000955793	-0.332910566
cg01400685	FADS2	0.002304201	-0.332961685
cg00323915	GIMAP4	0.000791389	-0.333078978
cg01741999	PNKD	4.34E-05	-0.333110856
cg08626131	SEPP1;SEPP1;SEPP1	0.003189589	-0.333205348
cg07975634	GJA5;GJA5;GJA5	0.000148457	-0.333306337
cg13365436	AP2A2	0.001948248	-0.333308356
cg07422329	PTGS2	0.000187194	-0.333320673
cg24494102	CNGA1	0.000357885	-0.333334784
cg05021589	LY86;LOC285780	1.50E-05	-0.333400017
cg21855816	C7orf50;C7orf50;C7orf50	0.0050758	-0.333404448
cg18103836		0.000148457	-0.333425075
cg24770256	FOXK2	0.003734675	-0.333531132
cg03722295		0.000290372	-0.333542525
cg17999642	LY6G5C	0.006823935	-0.333575465
cg25817639	ODZ3	0.000233422	-0.333601682
cg01523027	ZNF799	0.002304201	-0.333620116
cg04530852	GTPBP5	4.34E-05	-0.333628973
cg11224946	MEGF6	3.34E-05	-0.333667437
cg01147055	RASA3	0.0050758	-0.333672933
cg04447756	MATR3;MATR3	0.001639597	-0.333741487
cg25320816	ADPRHL1;ADPRHL1	0.000187194	-0.333776896

cg07710335	PMP22;PMP22;PMP22	0.002304201	-0.333792416
cg12496975	RAPGEF3;RAPGEF3;RAPGEF3	0.009066563	-0.333816125
cg20956114	PTPRN2;PTPRN2;PTPRN2	0.000791389	-0.333851276
cg12877853	PTPRN2;PTPRN2;MIR595;PTPRN2	0.000148457	-0.333871983
cg01597784		0.002304201	-0.333991857
cg03347934	C5orf56	7.23E-05	-0.334078528
cg02471848		0.003734675	-0.33408243
cg10767662	NFIX	0.0050758	-0.334235914
cg03605761	RNF126	0.000187194	-0.334286968
cg04398156		0.004357816	-0.334302626
cg03313126	ALLC	0.003189589	-0.334306145
cg25673241	RPTOR;RPTOR	0.001147425	-0.334313645
cg08850729	FGGY;FGGY	3.34E-05	-0.334319734
cg10558740	FLJ22536	0.000791389	-0.334410884
cg09179248	PRKCZ;PRKCZ;PRKCZ	0.002304201	-0.33442021
cg02921257	XIRP1	0.000537905	-0.334444659
cg12078154	RPTOR;RPTOR	0.00043997	-0.334471505
cg10035831	RPTOR;RPTOR	0.000365373	-0.334480265
cg10278213	CBFA2T3;CBFA2T3	0.002714607	-0.334492866
cg22321036		0.000148457	-0.334547514
cg21128953	MTUS2;MTUS2	0.001639597	-0.334557304
cg11362935	POU2AF1	8.60E-06	-0.334567017
cg00130947	LAMA2;LAMA2	9.24E-05	-0.334584648
cg00129811	DSE;DSE	3.34E-05	-0.33460069
cg00543073	BCL11A;BCL11A;BCL11A	0.001147425	-0.334611196
cg03358588	KCNT1	0.002714607	-0.334646289
cg24307601		0.000117986	-0.33473099
cg03787282		4.34E-05	-0.334748157
cg07004075	REC8;REC8	0.001376172	-0.334793471
cg05079191	ZNF804B;MGC26647	0.003734675	-0.334817953
cg00741986	TNIP2;TNIP2	1.96E-05	-0.33482763
cg16706546	IGF2BP2;IGF2BP2	0.004131428	-0.334839887
cg15123742	CSGALNACT1;CSGALNACT1;CSGALNACT1	0.000148457	-0.334958372
cg02948862	CBFA2T3	0.001639597	-0.334970381
cg24442766	INADL	0.001948248	-0.335019064
cg04477962	METTL7A	0.001376172	-0.335041083
cg06524846		0.00043997	-0.335046887
cg15468423	GNG7	0.000357885	-0.335149922
cg10816378		0.000148457	-0.335170244
cg10552473		0.003189589	-0.335341037
cg00033909	PER4	4.34E-05	-0.335370792
cg06267718	ELFN2	0.001147425	-0.335385795
cg27064845	GLTSCR2	0.000357885	-0.335389404
cg27457201	RPTOR;RPTOR	0.000654035	-0.335436187
cg01692626	DYNC1H1	0.002304201	-0.335440929
cg27627493		0.000187194	-0.335446236
cg00981070	PRKCZ;PRKCZ;PRKCZ	0.00043997	-0.335583985
cg07698793		0.002714607	-0.335591659
cg04781080	CORO2B	2.58E-05	-0.335594389
cg01297020	DAOA;DAOA;DAOA	4.34E-05	-0.335604443
cg07172334	KRTAP24-1;KRTAP24-1	4.34E-05	-0.335606298
cg19131667	TBC1D5;TBC1D5;TBC1D5	0.000187194	-0.335614769
cg01513063	SEPT9;SEPT9;SEPT9;SEPT9;SEPT9;SEPT9;S	0.000187194	-0.335643938
cg10247711	WDR25;WDR25	0.000148457	-0.335682891
cg27494470		0.00043997	-0.335691171
cg03737367	MIR150	0.00043997	-0.335711999
cg10959672	KIAA0319L	0.000791389	-0.335823908
cg09464728	CYGB	0.000955793	-0.335867274
cg20892919		0.002714607	-0.335910531
cg26960322	CXCR7	0.0050758	-0.335915598

cg26226408	LOC646982;LOC646982;LOC646982	0.000233422	-0.33593141
cg03805182	ZFHX3;ZFHX3	0.000117986	-0.335950801
cg06487369	SLC38A10;SLC38A10	2.58E-05	-0.336044114
cg14651650	OPA1;OPA1;OPA1;OPA1;OPA1;OPA1;OPA1;O	3.34E-05	-0.336101664
cg00861207	ABLIM2;ABLIM2;ABLIM2;ABLIM2;ABLIM2;ABLI	0.003189589	-0.336130893
cg02046475	ELP3	0.000187194	-0.336169741
cg12661092	SLIT1	0.000187194	-0.336190993
cg20146241	RCAN3	1.11E-05	-0.336239263
cg08856268	RPRD2	0.000955793	-0.336264438
cg24486958		0.000537905	-0.336340955
cg11108991	KCNMB3;KCNMB3	9.24E-05	-0.336370574
cg06242243	NCOR2;NCOR2	0.0050758	-0.336398976
cg00856825	GHRL;GHRL;GHRL;GHRL;GHRL;GHRL;GHRL(	0.009066563	-0.336430673
cg21518332		0.000290372	-0.336438519
cg16284674	ZNF511;TUBGCP2	0.004357816	-0.336505649
cg11177526	PYHIN1;PYHIN1;PYHIN1;PYHIN1	0.001639597	-0.336526046
cg15375424	IRF1	0.000654035	-0.336539188
cg10719144		0.002714607	-0.336547297
cg22680073		1.96E-05	-0.336629398
cg20901032	CSNK1A1L	7.23E-05	-0.336878851
cg17156862	KLHDC7A	0.000187194	-0.336972453
cg04929543	OSBPL6;OSBPL6	0.006823935	-0.337008427
cg15168958	FARP1	0.000955793	-0.337037469
cg19611013		0.003189589	-0.337047169
cg19519593	PDE7A	0.000357885	-0.337105869
cg22926923		0.003734675	-0.337129403
cg11497377	SFRS12;SFRS12	7.23E-05	-0.337171705
cg02057305		0.000654035	-0.337291369
cg08217910	TMEM37	9.24E-05	-0.337317576
cg26270263		0.001376172	-0.337656272
cg00443981	C17orf64	0.002304201	-0.337678704
cg08460812	LOC641518;LEF1;LEF1;LEF1;LEF1	2.58E-05	-0.337751657
cg26797124		8.60E-06	-0.337807772
cg10426370	ESM1;ESM1	0.000233422	-0.337811589
cg03538922	LOC100129637	0.000233422	-0.337852018
cg08628269		0.00043997	-0.337858094
cg02690117		0.000537905	-0.337905701
cg26544722		5.59E-05	-0.337945582
cg25607670	MFI2;MFI2	1.96E-05	-0.338141568
cg21739584	BCL6;BCL6;BCL6	0.000187194	-0.338142405
cg17298005	ELK3	0.004357816	-0.33815823
cg26969933	C6orf47;BAT4	0.000233422	-0.338197491
cg05246645		0.000187194	-0.33819911
cg27217350	C2orf85	1.50E-05	-0.338227
cg18788890	OR7C2	0.000117986	-0.338264969
cg22851561	C14orf43;C14orf43	0.000187194	-0.338278391
cg10491546	CTBP2;CTBP2	0.007873393	-0.338294519
cg17581870	C7orf50;C7orf50;MIR339;C7orf50	0.003734675	-0.338341311
cg04918831	CHML;CHML;OPN3	0.009066563	-0.338347772
cg04583842	BANP;BANP	7.23E-05	-0.338375211
cg17442852	SLC43A2	0.000233422	-0.338423415
cg16522993		0.000233422	-0.338521598
cg14341131	KCNT1	0.001147425	-0.33857202
cg13849419	TJP2;TJP2;TJP2;TJP2	0.001376172	-0.338575772
cg04462378	NUMA1	0.001376172	-0.338643414
cg16578549	CTNNBIP1;CTNNBIP1	0.000187194	-0.338669729
cg16828576		3.34E-05	-0.338684218
cg12434889	PTPRN2;PTPRN2;PTPRN2	0.000290372	-0.338722628
cg13388769		0.0050758	-0.338732478
cg13808842		0.000654035	-0.338895106

cg16171213		0.001376172	-0.338907166
cg13522882	MAP4K4;MAP4K4;MAP4K4	0.003734675	-0.338919337
cg03251852	PRX;PRX	0.003734675	-0.338940146
cg20645966		6.94E-05	-0.339012878
cg17401720	PTPRN2;PTPRN2;PTPRN2	0.002714607	-0.339165868
cg10030250	MMP2;MMP2	0.002304201	-0.339259572
cg18847118		4.34E-05	-0.339308553
cg03774957		0.000233422	-0.339359892
cg22319052	SNX8	0.000233422	-0.339378916
cg07910529	MAD1L1;MAD1L1;MAD1L1	0.000187194	-0.339381384
cg14202937	AMPD2;AMPD2;AMPD2	0.009066563	-0.339406085
cg25318211	MICAL1;MICAL1	0.000117986	-0.339424261
cg07017242	IMPDH1;IMPDH1;IMPDH1;IMPDH1;IMPDH1;IM	0.000148457	-0.339528704
cg13621396	STIM2;STIM2;STIM2	0.0050758	-0.339650306
cg05982473	OSBPL5;OSBPL5;OSBPL5	0.000537905	-0.339659021
cg17298510	LRFN2	0.002304201	-0.339680887
cg26102517		0.000955793	-0.339681517
cg25422880	TMEM163	0.000117986	-0.33974382
cg05900530	WDR49	5.11E-05	-0.339811174
cg03318573	NAA30	0.000117986	-0.339820429
cg05957567	BCL7A;BCL7A	3.34E-05	-0.339846202
cg13935127	RIMS3	0.002714607	-0.339847724
cg10997248	FXYD2	1.96E-05	-0.339927839
cg13458384	SARDH;SARDH	0.000654035	-0.339967811
cg18745507	ZGLP1	9.24E-05	-0.34003107
cg01113530		0.007873393	-0.340050414
cg20687098	WIPF1;WIPF1	9.24E-05	-0.340063748
cg03283990		3.34E-05	-0.34014799
cg23047992	CUX1;CUX1;CUX1	0.000187194	-0.340149256
cg16462701	CD81	0.003734675	-0.340195461
cg19851560	SMYD1	0.000537905	-0.340214365
cg06771256	CBFA2T3;CBFA2T3	0.001376172	-0.340247011
cg04561791		3.34E-05	-0.340277184
cg23074762	CHSY1	0.0050758	-0.340316131
cg04847110		0.000654035	-0.340333824
cg05720721		7.23E-05	-0.340409234
cg01935086	IQCC;IQCC;DCDC2B	0.000148457	-0.340420386
cg14765206	DNMBP	0.000955793	-0.340473287
cg10451425	ARMC5;ARMC5	5.59E-05	-0.340523382
cg18202741	AQP6	0.000233422	-0.340529799
cg00511027	C7orf50;C7orf50;MIR339;C7orf50	0.002714607	-0.340568759
cg04400533	ETV5	7.23E-05	-0.340583933
cg20957428	APOC3	5.59E-05	-0.340584609
cg24055836	FAM190A;FAM190A	0.000537905	-0.340632315
cg04731810	HLTF;HLTF	0.000791389	-0.340654507
cg14170999	BAHCC1	0.005897668	-0.340665712
cg06499213	TRAF3IP2;TRAF3IP2;TRAF3IP2;TRAF3IP2;TRA	0.005897668	-0.34066606
cg00889627	GABRD	0.000654035	-0.340672062
cg09103979	INPP5A	0.00043997	-0.340674149
cg14465207	PDIA6	5.59E-05	-0.340743133
cg15704408	NFATC1;NFATC1;NFATC1;NFATC1	0.0050758	-0.340768824
cg14162361		0.001376172	-0.340786568
cg25546253		0.001376172	-0.340827044
cg03512414	RIN2	0.004357816	-0.340971086
cg05809437	DLGAP2	0.000654035	-0.340985465
cg02122920	PTCRA	0.007873393	-0.341025924
cg13630239	RRP12;RRP12	0.000233422	-0.341065861
cg05673966		0.000148457	-0.341088142
cg03345925	ZC3H3	0.000290372	-0.341142119
cg06567829	ANKRD11	0.002304201	-0.341283363

cg27485075	LOC100134259	7.23E-05	-0.341336255
cg14505616	C18orf1;C18orf1	0.000262362	-0.341361153
cg18482303	MGAT5	0.000791389	-0.341427145
cg06484432	ADAMTS19	0.000654035	-0.341437533
cg21204860	SEPT9;SEPT9;SEPT9;SEPT9;SEPT9;SEPT9	3.34E-05	-0.341458367
cg08384322	C16orf46;C16orf46	0.003189589	-0.341463397
cg08823240	MAP3K14	7.23E-05	-0.341556155
cg13535736	C9orf5	0.002304201	-0.341581225
cg05542646		0.003189589	-0.341602548
cg22372096	ZFR2	2.58E-05	-0.34171259
cg01936091	ABLIM2;ABLIM2;ABLIM2;ABLIM2;ABLIM2;ABLI	0.005897668	-0.341773635
cg05626616	SEPT9;SEPT9;SEPT9;SEPT9;SEPT9;SEPT9;SI	0.000537905	-0.341870771
cg10089657	C7orf50;MIR339;C7orf50;C7orf50	2.58E-05	-0.341870852
cg05863755	TSPAN9;TSPAN9	0.004357816	-0.341937046
cg22315903	C21orf2	5.59E-05	-0.342001958
cg26692003	IQSEC1	0.001147425	-0.342081897
cg09655520	PRKCA	0.000357885	-0.34209987
cg18505871	RNF44	5.59E-05	-0.342122638
cg13308743		0.000791389	-0.342262432
cg01830053		0.004357816	-0.342263395
cg21305315	LRPAP1	0.000117986	-0.34230739
cg04623297	RFX8	0.003189589	-0.342316245
cg00295485	UXS1	4.34E-05	-0.342327765
cg03965138	SIGLEC5	0.000187194	-0.342360936
cg09140232		0.000654035	-0.342419997
cg01966334	MYO7B	4.34E-05	-0.342435374
cg17184479	EXOC6;EXOC6	0.000791389	-0.342524017
cg01643090	CAMTA1	0.000791389	-0.342560065
cg26895622		0.006823935	-0.342643642
cg00088797	IQSEC1;IQSEC1	0.000117986	-0.342659397
cg16624891	CTSB;CTSB;CTSB;CTSB;CTSB	0.001147425	-0.342660129
cg06070269	GOT2	0.000148457	-0.342660486
cg19589800	UBE2N	7.23E-05	-0.342686631
cg05567440		0.001948248	-0.342724119
cg26658743		9.24E-05	-0.342792235
cg11961495	ZMIZ1	0.000233422	-0.342826197
cg16754216		0.004357816	-0.34284862
cg16715687		0.005897668	-0.343030729
cg18825594	KLF11	0.001147425	-0.343055925
cg18165707	DENND3	0.003189589	-0.34308467
cg10354512	WISP1;WISP1	0.003060839	-0.343098723
cg05386726		0.000357885	-0.343126101
cg19145398	FOXS1	0.000233422	-0.343139784
cg08867471		0.00043997	-0.343186145
cg19641315	P2RY6;P2RY6;P2RY6	0.000187194	-0.343201362
cg14925764		0.002714607	-0.343280814
cg13488011		3.34E-05	-0.343289858
cg22020227	OLFML3	0.000955793	-0.343400714
cg25644380	BCL3	0.0050758	-0.343411447
cg06099971	PLA2G4D	5.59E-05	-0.343417767
cg02294870		0.007873393	-0.343476882
cg23014759	BCL9	0.000537905	-0.343478017
cg03064100		0.002266401	-0.343571115
cg22888023	RPTOR;RPTOR	0.004357816	-0.343607172
cg13647973		0.000290372	-0.343621537
cg14091154	FRAS1;FRAS1	0.000187194	-0.343631143
cg17494034	VPS13D;VPS13D	9.24E-05	-0.343686875
cg09940188	SMYD4	0.00043997	-0.343689555
cg25643229		0.000148457	-0.343747166
cg04440811	PRDM10;PRDM10;PRDM10;PRDM10	0.0050758	-0.343794728

cg22799757	SCRN1;SCRN1;SCRN1;SCRN1	0.000117986	-0.343908301
cg17256234		0.000791389	-0.343989432
cg24101039	SP4	0.002304201	-0.344118922
cg18406852	CHST10	0.007873393	-0.344167639
cg14477471		0.000148457	-0.344216671
cg20511548	OSBPL5;OSBPL5;OSBPL5	0.007873393	-0.344253383
cg18232841	FXYD3;FXYD3;FXYD3;FXYD3;FXYD3	0.000654035	-0.344304631
cg14850660	HEBP1	0.000537905	-0.344325837
cg27025738	SIPA1L1	0.000233422	-0.344342359
cg08741688	RGS12;RGS12;RGS12	9.24E-05	-0.344379052
cg21057323	CDC42BPB	1.11E-05	-0.344393754
cg00848594	C10orf25;ZNF22	7.23E-05	-0.344421331
cg15599668	SYNPO;SYNPO	0.003189589	-0.344471585
cg16049597		0.003189589	-0.344496537
cg15673994	TNRC4	0.000148457	-0.344520716
cg26555531	ADARB2	0.001948248	-0.344530954
cg01117384	PMEPA1;PMEPA1;PMEPA1;PMEPA1	0.000117986	-0.34461649
cg01827012	TRRAP	0.007873393	-0.344619525
cg22386008		0.000791389	-0.344674023
cg26609398	KCTD5	4.34E-05	-0.344708919
cg03324138		2.58E-05	-0.344756795
cg25578476		0.001948248	-0.344771688
cg11142556	TBCD	0.000654035	-0.344849273
cg04275362	RCSD1	5.59E-05	-0.344896956
cg18287768	TNFAIP3	0.000955793	-0.344965293
cg16513459	KCNAB3	0.001948248	-0.344971716
cg07585928	DUSP15;TTLL9;DUSP15;DUSP15	0.000357885	-0.344981908
cg27059970		0.002304201	-0.344993996
cg21966764	OR52B2	0.000654035	-0.345020493
cg14724265	PPEF2;PPEF2	4.34E-05	-0.345026976
cg13375463	AHDC1	0.000955793	-0.345123474
cg04597312	IRF8	0.004357816	-0.345144579
cg24554439	TMCO3	0.000233422	-0.345158077
cg14753070	IL27RA	0.002304201	-0.34520934
cg12950829		3.34E-05	-0.345284017
cg00446697	HCCA2	0.000791389	-0.345369095
cg15704872	SLC9A2	0.001948248	-0.345417122
cg03249047	TBCD	9.24E-05	-0.345546651
cg04168494	UBASH3B	0.003189589	-0.345570101
cg20722088	DUSP6;DUSP6	0.000654035	-0.345680724
cg04856396	P2RX5;P2RX5	3.92E-05	-0.345693161
cg23619936		4.34E-05	-0.345717563
cg00663986	TBCD	0.000290372	-0.345745683
cg03879320	SLC7A5	0.000357885	-0.345774535
cg26904702	SLC27A3	0.000117986	-0.34577818
cg03366884	PIK3R5	1.96E-05	-0.345796119
cg05816398		0.001948248	-0.345807882
cg03366574		0.001147425	-0.345820838
cg05347794	FOXN3;FOXN3	0.000537905	-0.345967921
cg18479255	FAM124A	0.000148457	-0.346011711
cg14457610	LMOD3;LMOD3	0.000654035	-0.346022028
cg05328461	EIF3G	0.006823935	-0.346035787
cg02858625	BOC	0.002714607	-0.346110775
cg06719042		4.34E-05	-0.346131316
cg10614445	DNMT3A;DNMT3A;DNMT3A	5.59E-05	-0.346139331
cg14507658		0.001376172	-0.346199429
cg08564487	SEPT9;SEPT9;SEPT9	0.002304201	-0.34624906
cg20504474		0.000537905	-0.346287558
cg09081994	CDGAP	0.000654035	-0.346299301
cg18362538	KDM4B	0.001376172	-0.346370316

cg11360546	C7orf50;C7orf50;C7orf50	0.000117986	-0.346447255
cg02586712	EHD4	7.23E-05	-0.346479883
cg06686156	ACSM5	2.58E-05	-0.346494407
cg21230021	MIR412;MIR410;MIR409;MIR369;MIR541	0.003189589	-0.346504852
cg08441850	IKZF3;IKZF3;IKZF3;IKZF3;IKZF3;IKZF3	1.50E-05	-0.346521968
cg10416668	IL21R;IL21R;IL21R	0.000290372	-0.346524467
cg02729344		0.000654035	-0.34656165
cg20318272	PODXL;PODXL	7.23E-05	-0.346568228
cg02525435	CBFA2T3;CBFA2T3	0.000233422	-0.346573712
cg20091384	GNG7	0.001376172	-0.34660229
cg01792749	SCN10A	0.001376172	-0.346626829
cg27353085	PLXND1	0.001948248	-0.346646975
cg23859051	SLC43A2	0.002714607	-0.346676158
cg26797722	JARID2	5.59E-05	-0.346684631
cg03626672	CXCR7	7.23E-05	-0.346719561
cg03025825	SMG6;SMG6	0.001376172	-0.346766142
cg14096899		2.58E-05	-0.346800933
cg27205928	C7orf50;C7orf50;C7orf50;MIR339	0.003189589	-0.346861724
cg15756359		0.000117986	-0.346907722
cg18417061	PRKCH	0.002714607	-0.346926764
cg09169516	MAPKAPK2;MAPKAPK2	0.002714607	-0.346928806
cg18366956	SDK2	5.59E-05	-0.346964061
cg05819223	ROR1;ROR1	7.23E-05	-0.346973045
cg14473838		4.34E-05	-0.346975786
cg14343017		0.006823935	-0.346989626
cg11356375	CDYL;CDYL;CDYL;CDYL	0.000791389	-0.3470216
cg13654573	DDHD1;DDHD1;DDHD1	0.000148457	-0.347059079
cg24998197		0.000148457	-0.347072158
cg18536994	LOC283999	0.004357816	-0.347191726
cg05191839		0.000537905	-0.347247599
cg13814351	FAM163B	0.000148457	-0.347339731
cg08360638		7.23E-05	-0.347383731
cg11683191		0.000357885	-0.347395927
cg18316498	POU2AF1	8.60E-06	-0.347398816
cg08705382	GALNT2	0.005897668	-0.347430571
cg03040848	CBFA2T3;CBFA2T3	0.000791389	-0.347459683
cg01468567	TEAD2	0.000122184	-0.347571055
cg16079645	C7orf50;C7orf50;C7orf50	4.34E-05	-0.34769257
cg10310700	TBCD	7.23E-05	-0.347711672
cg09334629		4.34E-05	-0.347760666
cg17164016		0.001948248	-0.347763629
cg23418201	MBOAT2	0.000791389	-0.347854032
cg12432568	MCF2L;MCF2L	0.000537905	-0.347972243
cg18802332	WISP1;WISP1	0.001147425	-0.347979356
cg14547801	PPFIA3	0.000148457	-0.348024102
cg01623771	KLHL30	4.34E-05	-0.348115202
cg26275799		5.59E-05	-0.348151921
cg09611620	FOXP1;FOXP1	0.000117986	-0.348176949
cg09802610	NPHP4	0.003734675	-0.348348026
cg07621239		0.000791389	-0.348447414
cg03923676	STT3B	0.000791389	-0.348489769
cg09059767	GAS8;GAS8	0.000233422	-0.348500183
cg22668906		9.24E-05	-0.348514943
cg02445907	C6orf222	2.58E-05	-0.348590805
cg04716447		0.000117986	-0.348600071
cg03653817		4.34E-05	-0.34865569
cg17102910	GPER;C7orf50;C7orf50;GPER;C7orf50;GPER	4.34E-05	-0.348755443
cg01843272	SBF1	2.58E-05	-0.348764519
cg00697658	KIAA1688	0.000654035	-0.348769823
cg13780428		0.005897668	-0.348824961

cg09595079	SPPL3	3.34E-05	-0.348944332
cg10775195	C14orf166B	0.0050758	-0.348947888
cg00302763		0.004357816	-0.348970808
cg18052528	TNIK;TNIK;TNIK;TNIK;TNIK;TNIK;TNIK;TNIK	0.000187194	-0.349002862
cg04792777		0.001639597	-0.349033669
cg12253071		0.000791389	-0.349043894
cg03364781	ALPK1;ALPK1	5.59E-05	-0.349045746
cg24126567	CYGB	0.000654035	-0.349062795
cg06747087	MIR595;PTPRN2;PTPRN2;PTPRN2	0.001376172	-0.349080151
cg23316807	ZNF787	0.000233422	-0.349219456
cg13479215	TMTC2	0.003189589	-0.349287974
cg10427868	ZMIZ1	9.24E-05	-0.34928869
cg02960418	PDGFA;PDGFA	0.00043997	-0.349383263
cg22353828	OSBPL5;OSBPL5;OSBPL5	9.24E-05	-0.349424925
cg13804478	MUC1;MUC1;MUC1;MUC1;MUC1;MUC1;MUC1	7.23E-05	-0.349495258
cg27300619		0.000233422	-0.349498858
cg18265297		4.34E-05	-0.34951438
cg12643293		3.34E-05	-0.349524121
cg04593460	MAD1L1;MAD1L1;MAD1L1	0.001639597	-0.349528703
cg26359730	TNR	7.23E-05	-0.349548584
cg00573857		0.001376172	-0.349672609
cg19972822	ZC3HAV1;ZC3HAV1	7.23E-05	-0.349692856
cg00401433	ZFPM2	7.23E-05	-0.34972367
cg02765962	HRH2;HRH2	0.000955793	-0.349802047
cg00321234	SCOC	0.000654035	-0.349806622
cg03179291	CAMKK1;CAMKK1;CAMKK1	0.001639597	-0.34988257
cg06423425	LFNG	0.003189589	-0.349909728
cg12159992	MYBPC1;MYBPC1;MYBPC1;MYBPC1	0.002304201	-0.349988831
cg12476455	NASP;NASP;NASP	0.000148457	-0.349992135
cg01408363		4.34E-05	-0.350013877
cg14405813	HIPK2;HIPK2	7.23E-05	-0.350403216
cg09791743	CNPY3	0.000791389	-0.350426676
cg10603136		1.50E-05	-0.350493345
cg05025332		1.11E-05	-0.350519141
cg19823847	KIAA1244;PBOV1	0.000791389	-0.350548332
cg12592276		4.34E-05	-0.350548978
cg08655953	PLCH2	0.003734675	-0.350614442
cg05242915		0.001376172	-0.350707691
cg24722198		0.000537905	-0.35078625
cg13492133	SMG7;SMG7;SMG7	0.006823935	-0.350865444
cg21330727	RAPGEFL1	0.000233422	-0.350882998
cg20570464	HIVEP3;HIVEP3	0.000537905	-0.350940421
cg05531174	CBFA2T3;CBFA2T3	0.003189589	-0.350971467
cg05350607	LINGO4	4.34E-05	-0.351009015
cg09926649	COMT;COMT;COMT	0.002927025	-0.351151207
cg01108697	MAPK8IP3;MAPK8IP3	0.002304201	-0.351186584
cg26495867		0.001948248	-0.35124385
cg15344504	STT3B	0.000791389	-0.351245581
cg18533546	ARHGEF10	0.002714607	-0.351278521
cg16664617	SLC27A1	0.000187194	-0.351535475
cg02857074	CACNA2D1	0.000357885	-0.351615636
cg22014289	SH3RF3	5.59E-05	-0.351676626
cg00937012	CPEB1;CPEB1;CPEB1;CPEB1	0.000290372	-0.351680036
cg03135983	ZFAT	0.000357885	-0.351764461
cg08426823	MAML2	0.000290372	-0.351839132
cg15651928	PXMP4;PXMP4	3.34E-05	-0.351887249
cg17988320	ZNRF2	5.59E-05	-0.3519604
cg16214653		0.006823935	-0.351969645
cg09249101		7.23E-05	-0.352029915
cg00483459	ALS2CL	0.004357816	-0.352032947

cg16674047		0.00043997	-0.352099178
cg23261073	CLSTN1;CLSTN1	0.000955793	-0.352101289
cg09863413		0.003734675	-0.352107304
cg19497709	COPG	0.003734675	-0.352152018
cg01360627	TNF	0.001147425	-0.352160322
cg08526209		0.000117986	-0.352187955
cg01727408		0.001376172	-0.352258421
cg10696062	WNT5B	0.003189589	-0.352267406
cg20666438		0.000791389	-0.352344666
cg24973755	MAEA;MAEA	0.001147425	-0.352382483
cg22146593		0.000187194	-0.352414802
cg11015768	ST3GAL5	4.34E-05	-0.352469156
cg14007090	LAMA5	0.000955793	-0.352501115
cg03730622	CELSR1	2.58E-05	-0.35250733
cg17023034	CACNB4;CACNB4;CACNB4	9.24E-05	-0.352521591
cg14228933		7.23E-05	-0.352574169
cg02006142	NSMCE2	0.0050758	-0.352659102
cg07165029	MAGI1;MAGI1;MAGI1	3.34E-05	-0.352663603
cg02835462	MAST2	0.000233422	-0.352678586
cg02823132	FAM38A	9.24E-05	-0.35276521
cg04781339		4.34E-05	-0.352772147
cg04664897		3.34E-05	-0.352867147
cg04977922	UHRF1	7.23E-05	-0.352869479
cg09292202	CACNA2D4	0.003734675	-0.353050371
cg01116491	BANK1;BANK1	0.006823935	-0.353100528
cg16745104	MIR941-1;MIR941-3;MIR941-2;MIR941-2;MIR94	0.000290372	-0.353148347
cg22014983	MAP4K3	5.59E-05	-0.353185811
cg24787238	MAD1L1;MAD1L1;MAD1L1	9.24E-05	-0.353268355
cg21865762	SCN8A	0.00043997	-0.35327848
cg07272042		0.001376172	-0.353287748
cg18041814	CPNE5	0.005897668	-0.35332205
cg01640684	TBCD	0.001393182	-0.353330876
cg01861740		0.006738759	-0.353360313
cg10274029		1.50E-05	-0.353409614
cg20491695		3.34E-05	-0.353458926
cg15548859		7.23E-05	-0.353496287
cg20458044	TMEM49	7.23E-05	-0.353571556
cg08119434		0.003189589	-0.353611615
cg03892714	SH3PXD2B	0.000791389	-0.353616292
cg10205310		0.001147425	-0.353633663
cg23593387	MAD1L1;MAD1L1;MAD1L1	7.23E-05	-0.353660999
cg22189286	HSPB8	0.000290372	-0.353711931
cg11134246	CCDC24	0.001147425	-0.353723442
cg03857384	OSBPL5;OSBPL5;OSBPL5	0.001147425	-0.353730941
cg26919780	HMHB1	0.002304201	-0.353743485
cg09526022	NRXN2;NRXN2	0.001376172	-0.353747953
cg00550955		0.00043997	-0.353868418
cg10022702	CBFA2T3;CBFA2T3	0.000955793	-0.35393898
cg01791634	TMC8	2.58E-05	-0.353944037
cg05894462	PTPRN2;PTPRN2;PTPRN2	9.24E-05	-0.353952774
cg16726039		0.002714607	-0.35395818
cg05112120		9.24E-05	-0.354009983
cg25338994		0.001948248	-0.354061729
cg04581293		0.000148457	-0.354098447
cg15718663	OSBPL5;OSBPL5;OSBPL5	0.000955793	-0.354119072
cg17093855	TMEM104	0.000290372	-0.354123086
cg06205746	ADPRHL1;ADPRHL1	0.000290372	-0.354237821
cg15078958		4.34E-05	-0.354271343
cg20210051	PDZRN4;PDZRN4	0.000187194	-0.354408458
cg14568203		0.001147425	-0.354428731

cg00930515		0.006823935	-0.35453137
cg04858631	DDIT4	1.96E-05	-0.354539651
cg08124000	OSBPL5;OSBPL5;OSBPL5	0.000148457	-0.354601142
cg03921696		0.000148457	-0.354631314
cg02689514	CBFA2T3;CBFA2T3	0.000537905	-0.354652079
cg03289764	LOC374443	0.000187194	-0.354689005
cg03585084	LHPP;LHPP	7.23E-05	-0.354690541
cg22900075	CUEDC1	2.58E-05	-0.354770984
cg21220462	SEMA4D;SEMA4D	0.001376172	-0.354799483
cg11554650	KIAA1949;KIAA1949	1.96E-05	-0.354872867
cg06396119		0.002304201	-0.354908629
cg03771580	OR5A2	0.000117986	-0.355058524
cg08616585	CDH1	5.59E-05	-0.355070321
cg18777299	STIM2;STIM2;STIM2	3.34E-05	-0.355091171
cg07426848	S100A3;S100A3	0.000117986	-0.355214422
cg17755321	TNF	0.006823935	-0.355222218
cg23088126	FAIM3;FAIM3;FAIM3	0.000955793	-0.355228004
cg27384918		1.60E-05	-0.355246988
cg20171453	RHOH	0.000654035	-0.355247946
cg08447200	PANX2;PANX2;PANX2	0.004357816	-0.355269799
cg04193065		7.23E-05	-0.355342094
cg24545100	LINGO4;RORC;RORC	0.000233422	-0.355379903
cg26075639		0.001393182	-0.35556545
cg09977718	RPTOR;RPTOR	0.003189589	-0.355599632
cg26438284	CD81	0.000148457	-0.355738006
cg03270204	DDR1;DDR1	0.005897668	-0.355787071
cg24394336	ARHGEF10L;ARHGEF10L	0.001147425	-0.355852963
cg23022999	RINL	0.002714607	-0.355933952
cg08823209	FXYD3;FXYD3;FXYD3;FXYD3;FXYD3;FXYD3;F	0.000117986	-0.355955946
cg13618880		0.000537905	-0.355969606
cg03657064	GPR160	0.000537905	-0.356052992
cg08825200	KCNMB3;KCNMB3	0.000117986	-0.356116607
cg08772206	SH2D4B;SH2D4B	0.0050758	-0.356140369
cg06935361	BRCA2	4.34E-05	-0.356253832
cg13683301		0.002304201	-0.356260643
cg08766211		0.000537905	-0.356275152
cg06789500	MAD1L1;MAD1L1;MAD1L1	0.000955793	-0.356275647
cg26576937	EBF4	7.23E-05	-0.356369337
cg12011522	OSBPL6;OSBPL6	2.58E-05	-0.356418605
cg18131582	GLT25D2	3.34E-05	-0.356445034
cg09791746	ADAMTSL5	0.000290372	-0.356598968
cg23716690	KIF1B;KIF1B	0.00043997	-0.356624319
cg23326607		3.34E-05	-0.356731048
cg04250930	ADORA2A	9.24E-05	-0.356749815
cg12216435	COPG	1.96E-05	-0.356753269
cg15006828		2.58E-05	-0.356822624
cg23217983	SGIP1	0.000148457	-0.35699369
cg19734164	C5orf56	0.000117986	-0.357054972
cg20018425	PANX2;PANX2;PANX2	5.59E-05	-0.357075409
cg06272045		9.24E-05	-0.35722668
cg27592424	C16orf58	0.004357816	-0.35726449
cg14071925	LILRA1	2.58E-05	-0.357272726
cg14182461	RNF157;FOXJ1	0.000290372	-0.357288857
cg07127456	TK2	5.59E-05	-0.357389776
cg05242065	C2orf89	0.000955793	-0.357409956
cg16686279		0.000117986	-0.357442404
cg00986598	TMCC1;TMCC1;TMCC1	0.00043997	-0.357540516
cg11701615	C20orf166	4.34E-05	-0.357653006
cg04444104		9.24E-05	-0.357689535
cg26146569	KLF13	0.002304201	-0.357714053

cg25934954	MAP4K2	7.23E-05	-0.357724504
cg08189139		9.24E-05	-0.357763168
cg14656948		0.000654035	-0.357800517
cg20125501		0.000290372	-0.357814806
cg01546472	ODZ3	0.000233422	-0.357843818
cg07668558	GPR179	0.000955793	-0.357855791
cg11418725		0.000357885	-0.357869197
cg16391727	PRKAG2;PRKAG2	0.000148457	-0.357955497
cg20146177	HAS2	0.000233422	-0.358083989
cg18601229	EHD4	0.001147425	-0.358091859
cg15415194		7.23E-05	-0.358091992
cg09174653	C7orf50;C7orf50;MIR339;C7orf50	0.000791389	-0.358170622
cg21243597	C18orf1;C18orf1	0.000654035	-0.358189435
cg10877430	DTX1	0.000148457	-0.358229543
cg02319972		4.34E-05	-0.358242628
cg08612271	AIG1	0.000654035	-0.358295878
cg04656831		5.59E-05	-0.358317678
cg13356324	GUK1;GUK1;GUK1	0.001147425	-0.358367153
cg10511902	CCDC88C	0.001376172	-0.358532207
cg02251850	RPTOR;RPTOR	0.001376172	-0.358633887
cg10283844	HPCAL1;HPCAL1	0.002714607	-0.358847413
cg06573088	CAP2	9.24E-05	-0.358867234
cg15247329		4.34E-05	-0.358896353
cg08488915	SLC43A2	0.006738759	-0.358979455
cg06691250		0.000187194	-0.35898673
cg26643870		0.000357885	-0.359002566
cg27388962	LOC100216001	9.24E-05	-0.359093166
cg08263416	RGS6	4.34E-05	-0.359137421
cg11465630	C21orf33;C21orf33	0.001376172	-0.359153005
cg06559878	ITK	3.34E-05	-0.35919846
cg20300911		1.96E-05	-0.359209293
cg14220262	TTLL10;TTLL10	4.34E-05	-0.359211754
cg07298347	FMNL2	0.007873393	-0.35924696
cg01613294	APOL3;APOL3;APOL3;APOL3;APOL3;APOL3	0.001147425	-0.359278616
cg01245155	PARD3B;PARD3B;PARD3B	0.000290372	-0.359281072
cg02662417	ARID1B;ARID1B;ARID1B	0.000187194	-0.359314972
cg14248717	C9orf171	0.008829128	-0.359353034
cg10853416	MS4A7;MS4A7;MS4A7;MS4A7	4.34E-05	-0.359403797
cg24520862		1.96E-05	-0.359433894
cg10359807	USP7	0.00043997	-0.359439901
cg12991306	C8orf80	4.34E-05	-0.35950684
cg02784232	PHC3	0.000148457	-0.359543544
cg10456541		3.34E-05	-0.359544041
cg18741277	ZGLP1	8.60E-06	-0.359552519
cg26955540	SEMA4B	0.000148457	-0.359624483
cg18973238		0.003734675	-0.359674676
cg13559409	ABCA1	0.002304201	-0.35969034
cg26534812		0.00043997	-0.359707356
cg03017850	SPTBN1;SPTBN1	0.000955793	-0.3597193
cg04406229		0.001376172	-0.359810393
cg25337513	RPTOR;RPTOR	2.58E-05	-0.359811873
cg26448137		0.000148457	-0.359839391
cg26649096		1.50E-05	-0.359864176
cg09644974		0.003734675	-0.359868247
cg18943588		0.000148457	-0.359902145
cg15316716	DOK2	2.58E-05	-0.359931023
cg09863136	SH2B2	0.000357885	-0.359940911
cg15246989	PLXDC1	9.24E-05	-0.359952848
cg15931673	TMCO4	0.00043997	-0.359983455
cg04134235	FAM49A	0.000233422	-0.360046492

cg27635453		0.003734675	-0.360090137
cg15208525	CNTNAP2	9.24E-05	-0.360122206
cg08241841	LSM7	0.001376172	-0.360123819
cg00039016	FBXO31	0.000955793	-0.360240786
cg09664479	CD82;CD82	2.58E-05	-0.36025162
cg21913301	PDE3A	2.58E-05	-0.36029583
cg04931913	ATP2B2;ATP2B2	4.34E-05	-0.36034596
cg21674927	IL1R2;IL1R2	0.000791389	-0.360408354
cg14286456	RERE;RERE	4.34E-05	-0.360452218
cg02781105	KIF19	1.96E-05	-0.360462931
cg24924502	AATK	0.001147425	-0.360509407
cg09129334	ARHGEF7;ARHGEF7;ARHGEF7;ARHGEF7;AR	5.59E-05	-0.360519727
cg15174117	CORO2B	0.004357816	-0.360574553
cg19649018		0.000290372	-0.360652292
cg11874976		0.000233422	-0.360725358
cg19816586	SYT1;SYT1;SYT1	0.003734675	-0.360829041
cg23714707	PEMT;PEMT;PEMT	0.001639597	-0.360873046
cg03695260		0.001639597	-0.360917443
cg17525436		1.96E-05	-0.36091822
cg15471388		0.000357885	-0.360927013
cg01404750	ATP11A;ATP11A	0.000233422	-0.360934125
cg09234973	PAX8;PAX8;PAX8;PAX8;LOC440839;PAX8	0.000537905	-0.360935158
cg21350909		9.24E-05	-0.360938652
cg15507449		0.002304201	-0.360969146
cg13977835		0.001147425	-0.361039385
cg13168221	C1QA	0.000117986	-0.36106101
cg01445100	BANP;BANP	4.34E-05	-0.361064941
cg08651003	GNB1	0.006823935	-0.361111151
cg10039047	B3GNTL1	0.000117986	-0.361214725
cg23863328		0.000187194	-0.361275943
cg21582611		7.23E-05	-0.361349489
cg19935065	DNTT;DNTT	4.34E-05	-0.36136704
cg26141291	DENND3	0.000290372	-0.361375522
cg26230851	PCP4L1	0.006823935	-0.361472059
cg17890667	PHTF2;PHTF2;PHTF2;PHTF2;PHTF2	0.001948248	-0.361504699
cg13810695		9.24E-05	-0.361577839
cg02113385	NFATC1;NFATC1;NFATC1;NFATC1;NFATC1	5.59E-05	-0.361618551
cg16988611	TSPAN14;TSPAN14	5.59E-05	-0.361629091
cg25246082	TMEM156	0.000290372	-0.361785766
cg23712594	PARP1	0.000187194	-0.361812299
cg09087961	IGF2BP3	0.000117986	-0.361827414
cg02127689		0.000233422	-0.361847635
cg07568430	CSF1;CSF1;CSF1;CSF1	7.23E-05	-0.361864232
cg03408354	SLC7A5	0.001147425	-0.361869534
cg05927789	TTC7A	4.34E-05	-0.361899081
cg21121609	FCER2;FCER2	0.000148457	-0.362003924
cg19384448		1.96E-05	-0.362006651
cg24469742	STARD13;STARD13	0.000654035	-0.362157011
cg25962657		0.006823935	-0.362190512
cg27470486	ACLY;ACLY	1.50E-05	-0.362220725
cg13613180	XRRA1	0.000357885	-0.362252371
cg16774946	SPATA13;SPATA13	7.23E-05	-0.362377825
cg13407335	KCNAB3	0.002714607	-0.36238976
cg14087413	TMEM8B;TMEM8B	4.34E-05	-0.362397592
cg02378006	UNC5B	0.000955793	-0.362647526
cg26662512	MAD1L1;MAD1L1;MAD1L1	4.34E-05	-0.362678255
cg02487823	KIAA0226;KIAA0226	5.59E-05	-0.362717831
cg02212836	LY86;LOC285780	1.50E-05	-0.36274629
cg01800148	SLC2A5;SLC2A5;SLC2A5	0.000233422	-0.362861162
cg16471612		0.000233422	-0.362868201

cg05020759		0.001948248	-0.362896705
cg25745642	EFNA3	7.23E-05	-0.362911001
cg21185255		0.001147425	-0.362937696
cg12754854	PHYHD1;PHYHD1	0.001639597	-0.36299966
cg01709312	LOC282997;PDCD4;PDCD4	1.96E-05	-0.363013406
cg12348511	OSBPL5;OSBPL5;OSBPL5	0.000117986	-0.363106299
cg01866518	PXN;PXN;PXN	5.59E-05	-0.363228672
cg05303899		7.23E-05	-0.363314938
cg12534645	CAMK2A;CAMK2A	9.24E-05	-0.363436079
cg01807946	CSNK1D;CSNK1D	7.23E-05	-0.363451383
cg21428954	CELSR1	0.000537905	-0.363466789
cg25306932	LYSMD1;LYSMD1	2.58E-05	-0.363474433
cg08145839	LMF1	0.001948248	-0.363515435
cg13232176	KIAA1671	0.001639597	-0.363533716
cg13519194		9.24E-05	-0.363645907
cg06546787	DEFB112	5.59E-05	-0.363654467
cg19082416	DIP2C	0.001948248	-0.363708792
cg04416111		0.000357885	-0.363783802
cg21080533	PGK2	7.23E-05	-0.363803818
cg12152540	MKX	0.004357816	-0.363810419
cg25735823	TSSC1	9.24E-05	-0.363839871
cg01701303		0.003189589	-0.363907408
cg17813891	EVL	0.002304201	-0.363917366
cg11804940	WDR66	0.000233422	-0.363923913
cg13002189		0.000791389	-0.36396065
cg27296341	IPCEF1;OPRM1;IPCEF1;IPCEF1	0.000791389	-0.363963819
cg14471645	RADIL	0.000290372	-0.364047959
cg14136042	ATP6V1G2;NFKBIL1;NFKBIL1;ATP6V1G2	0.001147425	-0.364052164
cg12067421		0.000148457	-0.364115424
cg00001269		0.001948248	-0.364154834
cg02416333		5.59E-05	-0.364189025
cg00706536	ATP6V0A1;ATP6V0A1;ATP6V0A1	7.23E-05	-0.364196326
cg10938221	KIAA0564	0.002714607	-0.364224842
cg02852419	LMF1	0.005897668	-0.364382391
cg08553274	KIAA1530	0.001639597	-0.364458693
cg14267222		0.002714607	-0.364462801
cg01140247	C10orf11	7.23E-05	-0.36446623
cg09976287		4.34E-05	-0.364488458
cg13906362	LAT2;LAT2;LAT2	5.59E-05	-0.364617933
cg08145373	CD81	0.002304201	-0.364680286
cg02259723	ADPGK;ADPGK;ADPGK	0.000117986	-0.364709874
cg14192130	CCR6;CCR6	2.58E-05	-0.364766622
cg03713668	INO80	0.009066563	-0.364864133
cg17981790	FAM49A	4.34E-05	-0.364890256
cg13688202	SPTB	9.24E-05	-0.364897989
cg18151635		0.000233422	-0.364937318
cg09245302		0.009066563	-0.365055276
cg13782919	SYK;SYK;SYK;SYK	4.34E-05	-0.365073494
cg17608587	NCRNA00171	0.00043997	-0.365145034
cg04823738		1.11E-05	-0.365166594
cg03015498		0.000233422	-0.365193569
cg01514033		9.24E-05	-0.365204948
cg18013792	MAD1L1;MAD1L1;MAD1L1	0.001948248	-0.365244683
cg23579537	RNASEH2B;RNASEH2B	5.59E-05	-0.365254545
cg06819373	PTPRN2;PTPRN2;PTPRN2	0.007873393	-0.365301049
cg17806069		0.000148457	-0.365321018
cg07522372	C18orf1;C18orf1	9.24E-05	-0.365332816
cg22593342	TSPAN14;TSPAN14	7.23E-05	-0.365503169
cg21373806	BAHCC1	2.58E-05	-0.365590899
cg15778089	ARPP-21;ARPP-21;ARPP-21;ARPP-21	0.0050758	-0.365621789

cg22528123	SMAD3;SMAD3	0.000791389	-0.365652329
cg02981163		0.000654035	-0.365700731
cg01678292	ANK1;ANK1;ANK1;ANK1;ANK1;ANK1;ANK1;AN	0.000955793	-0.365735681
cg16098726	GP9	3.34E-05	-0.365743966
cg09800748	KIF21A	0.000537905	-0.365840907
cg05386769	TNS1	0.000148457	-0.365845884
cg04537602	CXCR5;CXCR5	1.50E-05	-0.36592899
cg21579975	FADS6	2.58E-05	-0.365967592
cg13924015		4.34E-05	-0.366014515
cg15122966		0.004357816	-0.36606386
cg00659129	ASAP1	4.34E-05	-0.36617074
cg05141988	SCML4	0.001147425	-0.366376803
cg01170124	ISCU;ISCU	0.001948248	-0.366382771
cg23991482	LSM7	0.00043997	-0.366385934
cg22700246		1.96E-05	-0.366395578
cg19682835	FAM116B	0.001376172	-0.366426912
cg07267067	SOAT2	0.00043997	-0.366509598
cg12715235	PAG1	0.006823935	-0.366520663
cg17831694	RPTOR;RPTOR	7.23E-05	-0.366551558
cg01852186	LIN7A	9.24E-05	-0.366612023
cg07921759	SAMD4A;SAMD4A	2.58E-05	-0.366618863
cg17729668		0.000537905	-0.366642645
cg05710479	CAMTA1	0.000654035	-0.366646986
cg07012823	NOX3	5.59E-05	-0.366689598
cg07708521		0.000233422	-0.366690967
cg16254267		0.001147425	-0.366739333
cg19002591		5.59E-05	-0.366871479
cg27627876	BANP;BANP	0.000233422	-0.366933694
cg10967866	INPP5A	0.000290372	-0.367071325
cg02550151	BRD1;BRD1	0.000148457	-0.367092694
cg02956499	TET3	0.000117986	-0.367158666
cg25366639		0.003734675	-0.36717609
cg18182844	ITIH3	0.001948248	-0.367243198
cg25315819		0.002304201	-0.367256859
cg08316831	HMG20A	0.004357816	-0.367298711
cg04404982	ITGAL;ITGAL	0.000791389	-0.367335205
cg07728579	FSD2	4.34E-05	-0.367344196
cg14630748	TNXB;TNXB	9.24E-05	-0.367400867
cg08258506	C14orf43	0.001639597	-0.367403978
cg12897164	FBXO32;FBXO32	2.58E-05	-0.367417332
cg24726137	CYGB	1.96E-05	-0.367425594
cg07532353		0.000570759	-0.367427787
cg13403369		0.001376172	-0.367495333
cg24027780	LOC728743	0.002304201	-0.367590328
cg11811559	TCL1A;TCL1A	0.000791389	-0.3676108
cg07935357	PEMT;PEMT;PEMT	0.000654035	-0.367655669
cg25268422	VAV2;VAV2	0.00043997	-0.367729148
cg21268658	PTPRF;PTPRF	0.002714607	-0.367756313
cg02939781	KLHL6	5.59E-05	-0.367765061
cg21877355	DYNC1H1	0.000357885	-0.367784058
cg01618697		0.000357885	-0.367814352
cg12224388		7.23E-05	-0.367819575
cg19501902	CD96;CD96	3.92E-05	-0.367840707
cg26686647	RGS12;RGS12;RGS12	4.34E-05	-0.367883806
cg16396223	PRKAG2;PRKAG2	0.002714607	-0.367924618
cg13765778	DAPK1	7.23E-05	-0.367929843
cg06825478	TNF	0.002706259	-0.368019035
cg19306970	HLX	8.60E-06	-0.368197342
cg08640804		0.000791389	-0.368207383
cg18114313		7.23E-05	-0.36829248

cg13280056	LOC728743	1.96E-05	-0.368334243
cg19346786		2.58E-05	-0.368503097
cg01143547	CCND3;CCND3;CCND3;CCND3	1.96E-05	-0.368505345
cg13753527		0.000290372	-0.36857432
cg10319629		0.000233422	-0.368576681
cg21526778	SDCCAG8	0.004357816	-0.368711287
cg07029002	ROR1;ROR1	5.59E-05	-0.368737493
cg01831771	ATP11A;ATP11A	0.004357816	-0.368773532
cg09174162	MAD1L1;MAD1L1;MAD1L1	0.000537905	-0.368786147
cg13475995		5.59E-05	-0.368836471
cg25311466	WFS1;WFS1	7.23E-05	-0.368860553
cg03517614		4.34E-05	-0.36889957
cg07324245	SEPT9;SEPT9;SEPT9;SEPT9;SEPT9;SEPT9	0.000117986	-0.368926292
cg12886494	ARPP-21;ARPP-21;ARPP-21	0.007873393	-0.369068698
cg16661609	LILRB4;LILRB4	0.000654035	-0.369095824
cg00735329	MBP;MBP	0.001376172	-0.369115032
cg18677148	CLTC	0.001639597	-0.36913973
cg02002247	SLC27A1	0.000654035	-0.369184919
cg09588284	ACRV1;ACRV1;ACRV1;ACRV1;ACRV1;ACRV1	0.000537905	-0.369227131
cg17163729		5.59E-05	-0.36926389
cg13621113	SFRS8	0.001147425	-0.369284176
cg16370778	ARPP-21;ARPP-21;ARPP-21;ARPP-21	0.007873393	-0.369332839
cg15473481	ITGA7	4.34E-05	-0.369395986
cg08888553	C16orf91	0.000117986	-0.369564564
cg06159562	ANKRD11	3.34E-05	-0.369584154
cg12964697	MAD1L1;MAD1L1;MAD1L1	5.59E-05	-0.369634452
cg03620886	ZGLP1	8.60E-06	-0.369655352
cg00862290	KCNMB3;KCNMB3	5.59E-05	-0.369771828
cg14982987	PPP2R5C;PPP2R5C;PPP2R5C;PPP2R5C;PPP2	0.000955793	-0.369838831
cg25193073	MAD1L1;MAD1L1;MAD1L1	5.59E-05	-0.369985899
cg10594837	DENND3	0.001147425	-0.370103241
cg13276502		4.34E-05	-0.370146282
cg19118072	PLXNC1	0.001147425	-0.370152096
cg09971102	MEG3;MEG3;MEG3	0.001376172	-0.370202371
cg22902799	CLEC14A	0.000357885	-0.370286781
cg22233020		3.34E-05	-0.370349936
cg13918228	CD69;CD69;CD69	0.000654035	-0.37037976
cg04257752	C21orf29;KRTAP12-3	0.000357885	-0.370431923
cg03708045	KIAA0146	0.000233422	-0.370527064
cg22376214		0.000357885	-0.37053937
cg09833475	PTPRN2;PTPRN2;PTPRN2	0.000233422	-0.370613962
cg13717350	SARDH;SARDH	4.34E-05	-0.370643374
cg05493344	SARDH;SARDH	3.34E-05	-0.37070175
cg16353350	C10orf55	0.000233422	-0.370740793
cg21188858	EBF1	0.001147425	-0.370770258
cg19364276		0.0050758	-0.37082994
cg02385728	FIBCD1;FIBCD1	0.007873393	-0.370864343
cg26768584	SATB1;SATB1	0.000290372	-0.370923037
cg18484299	GORASP2	0.000654035	-0.370925343
cg12111500		4.34E-05	-0.370971809
cg16173529	MSRA;MSRA;MSRA	0.000148457	-0.371050992
cg17829017	ZFATAS;ZFAT;ZFAT;ZFAT	4.34E-05	-0.371184508
cg06144110		1.11E-05	-0.371209983
cg17136255		3.34E-05	-0.371220617
cg14254562	TLE1	0.001948248	-0.371229165
cg15015996	ARHGEF16	3.34E-05	-0.371269162
cg22618337	C10orf81	0.000117986	-0.371360091
cg23112464	TMCO3	4.34E-05	-0.371372081
cg03625292	ABI1;ABI1;ABI1;ABI1	0.000187194	-0.371403303
cg08002981	BCAS3;BCAS3	0.007873393	-0.371460267

cg11688093	RAB20	2.58E-05	-0.371556471
cg21768702	C7orf50;C7orf50;C7orf50	0.000148457	-0.371613194
cg24264042		0.000187194	-0.371674627
cg26610739	CDH13	0.000955793	-0.371686941
cg17181543	AK5	0.003734675	-0.371734935
cg01758575	CD19;CD19	7.23E-05	-0.371908341
cg09773897	NFIC	5.59E-05	-0.371928575
cg17851868		0.000233422	-0.371970446
cg12743248	EFCAB1;EFCAB1;EFCAB1	9.24E-05	-0.372042767
cg22586996	EBF1	0.001948248	-0.372156387
cg02370232	SORBS1;SORBS1;SORBS1;SORBS1;SORBS1	0.000233422	-0.372161287
cg02713832	CD6	2.58E-05	-0.372249481
cg22870373	MAD1L1;MAD1L1;MAD1L1	0.000117986	-0.372298538
cg05050341	ENG;ENG	9.24E-05	-0.37236462
cg14997711	PTPRJ;PTPRJ	0.001147425	-0.372404844
cg23347250	BTBD6;BRF1;BRF1;BRF1	0.002714607	-0.372587541
cg13702949		0.000117986	-0.372684377
cg21828517	ZBTB46	0.001639597	-0.372732567
cg25611723	APCDD1	0.000187194	-0.372781124
cg16108580		3.34E-05	-0.3727944
cg20203400	TMEM182	0.000187194	-0.372819803
cg03742629		0.000117986	-0.372856436
cg10963218	S100A13;S100A13;S100A13;S100A1;S100A13;	0.001147425	-0.372860058
cg06879681	ARHGEF10	0.000290372	-0.372901612
cg02664177	PDGFA;PDGFA	0.000654035	-0.372966197
cg20203592	PHLDB2;PHLDB2;PHLDB2;PHLDB2	0.000117986	-0.37312199
cg15822656		0.001376172	-0.37316037
cg14649095		0.000654035	-0.373178631
cg14827643	RGS12;RGS12	1.50E-05	-0.373306322
cg18128887	RCAN3	1.50E-05	-0.37334563
cg06527166	HIVEP3;HIVEP3	0.00043997	-0.373506811
cg00707814	KDM2B;KDM2B	9.24E-05	-0.37352403
cg23230158	GALNT2	0.003734675	-0.373570521
cg10687217	ABR	3.34E-05	-0.373576958
cg07434082	ARHGEF7;ARHGEF7;ARHGEF7;ARHGEF7;AR	5.59E-05	-0.373605517
cg03786808	C17orf88	0.0050758	-0.37376093
cg23953820	DDR1;DDR1	3.34E-05	-0.373934054
cg22255288	RPTOR;RPTOR	0.001948248	-0.37396102
cg20095669	PPFIBP1;PPFIBP1	0.005897668	-0.373976371
cg22250556		0.000233422	-0.374010448
cg19359550		0.000654035	-0.374037255
cg13852084	HLA-DMA	0.000187194	-0.374138992
cg23893629	LAX1;LAX1	0.002304201	-0.374163806
cg03209332		0.00043997	-0.374165696
cg15775914	CHML;OPN3;CHML	4.34E-05	-0.374189298
cg00862307		0.001639597	-0.374194158
cg04760708	CBFA2T3;CBFA2T3	7.23E-05	-0.374255784
cg00031162	TNFSF12;TNFSF12-TNFSF13	1.96E-05	-0.374558692
cg10394757	CA7;CA7	0.000148457	-0.37472333
cg00316478	ACSF3	0.000187194	-0.374777474
cg14139311		1.96E-05	-0.37480483
cg01437221	MAD1L1;MAD1L1;MAD1L1	0.000791389	-0.374806284
cg13503476		4.34E-05	-0.374819752
cg02217184	GPR18;GPR18;UBAC2;UBAC2;UBAC2	0.000233422	-0.374865847
cg06060703	CCDC78	0.000187194	-0.375004261
cg18072687	CD81	0.001376172	-0.375042725
cg03841638		0.000233422	-0.375064519
cg09075743	MAD1L1;MAD1L1;MAD1L1	0.001639597	-0.375067326
cg02093062	ZFYVE27;ZFYVE27;ZFYVE27	0.000357885	-0.375104341
cg24065957	ARL15	0.006823935	-0.375159316

cg12103897	SNRNP70	0.001147425	-0.375173403
cg10296718	ARHGEF10	0.000233422	-0.375208198
cg24091819	GPR133	0.000357885	-0.375222664
cg07039378		5.59E-05	-0.375250551
cg13665684	FAM188B	7.23E-05	-0.375289685
cg02299020		0.005897668	-0.375300411
cg02410870	TRABD	0.000233422	-0.375581602
cg07448742	NRM	5.59E-05	-0.375727081
cg01439248	MYOM2	0.002714607	-0.375729453
cg06308720	ZMIZ1	0.000148457	-0.37575832
cg09372614		0.000117986	-0.375807623
cg16133703		0.000233422	-0.375838255
cg22979041	TIFAB;TIFAB	0.000148457	-0.375865992
cg25133951		3.92E-05	-0.37590903
cg08162476	IQSEC1	0.000148457	-0.375922621
cg03738656		0.000187194	-0.375994743
cg24368031	MRC2	0.001376172	-0.376048558
cg14712058	SIN3B	9.24E-05	-0.376064484
cg02192490		0.001147425	-0.376118035
cg19126910	POU2AF1	2.58E-05	-0.376145156
cg11970797	CRYL1	0.000148457	-0.37617339
cg07349815		0.001147425	-0.376299897
cg25510823		0.000290372	-0.376319642
cg16997486	NFATC1;NFATC1;NFATC1;NFATC1;NFATC1	0.000791389	-0.376383388
cg22688656	MYO16	0.000117986	-0.376440035
cg07207669	EFNA1;EFNA1	4.34E-05	-0.376464832
cg18566313	SORBS2;SORBS2;SORBS2;SORBS2;SORBS2	0.007873393	-0.376488995
cg03382805		0.002304201	-0.376569914
cg27190654		3.34E-05	-0.376576352
cg10211062		9.24E-05	-0.376627847
cg23830540	CRMP1;CRMP1	0.000791389	-0.376691683
cg24678438		3.34E-05	-0.376726468
cg04917258	PC;PC;PC	0.000148457	-0.376854573
cg14331899	CYBASC3;CYBASC3;CYBASC3	0.000148457	-0.376874882
cg14935025	CARS;CARS;CARS;CARS	1.96E-05	-0.376874996
cg12689752	IQGAP3	0.000117986	-0.376977167
cg05889171	GRAMD1B	0.000148457	-0.377007649
cg04930661	ADARB2	0.000187194	-0.377105894
cg08549806		0.000233422	-0.377124522
cg26040809	ADARB2	0.001376172	-0.377189912
cg20086579	ELOF1	0.009066563	-0.377234895
cg07438246	SLC6A6;SLC6A6;SLC6A6	0.000187194	-0.377235762
cg13712012		0.000537905	-0.377338975
cg09727148	UMODL1;UMODL1	5.59E-05	-0.377369701
cg17758673	RGS12;RGS12	0.00043997	-0.377391049
cg18899797		0.000205664	-0.377400942
cg21008363	POM121C	9.24E-05	-0.377406694
cg22487391		0.001887355	-0.377407164
cg22153312		0.002304201	-0.377495054
cg08498833	TG	0.000117986	-0.377514416
cg06880420	NR5A2;NR5A2	0.002304201	-0.377538411
cg11195707		0.001639597	-0.377585822
cg25330422	STAT3;STAT3;STAT3	0.000187194	-0.377600333
cg11183935	TBCD	9.24E-05	-0.377603453
cg19788186	KCNJ15;KCNJ15;KCNJ15	0.005897668	-0.377638307
cg12550597	FAM129C	4.34E-05	-0.377792032
cg21642988	DNAJA4;DNAJA4;DNAJA4	5.59E-05	-0.377959754
cg21617903	VGLL4;VGLL4;VGLL4;VGLL4	0.001639597	-0.378162198
cg27305939	MORN1;LOC100129534	3.34E-05	-0.378201296
cg19134130	TMEM104	2.58E-05	-0.378207853

cg00754552	NAPSB	1.50E-05	-0.378231506
cg08250921		0.000233422	-0.378352574
cg17426184	MAPKBP1;MAPKBP1	5.59E-05	-0.378539735
cg15438314	MYO9B;MYO9B	1.96E-05	-0.378566887
cg19478371	PPP2R5C;PPP2R5C;PPP2R5C;PPP2R5C;PPP	0.000537905	-0.37869159
cg25724842	PACRG;PACRG;PACRG	4.34E-05	-0.378722788
cg23867673	CDH23;CDH23	0.000290372	-0.378759949
cg16372051	HSPA1B	0.001376172	-0.378772558
cg26294551	GP5	0.000233422	-0.378797779
cg26120073	CCDC88C	4.34E-05	-0.378873845
cg07402396	INPP5A	0.006823935	-0.378897029
cg02433266		0.000187194	-0.378924188
cg22153994	ARHGAP27;ARHGAP27	0.000187194	-0.378931311
cg00432328	MYST4	0.00813378	-0.379061902
cg06452129		0.0050758	-0.379103111
cg18428825		0.000791389	-0.379253553
cg14688299		9.24E-05	-0.379304214
cg27304328	CD84	0.000148457	-0.37931588
cg25957332	DIDO1;DIDO1;DIDO1	0.000233422	-0.379365798
cg20275211	PPP2R2C;PPP2R2C	0.000148457	-0.379431444
cg26421805		0.000955793	-0.37945214
cg13499923		5.59E-05	-0.379565617
cg14326743	ERP44	0.001376172	-0.379648292
cg03110921	DLG5	2.58E-05	-0.379669073
cg23325384	ACSL3;ACSL3	9.24E-05	-0.379699174
cg27007060	RTN4IP1	0.001639597	-0.379722403
cg20429981	CDK11B;CDK11B;CDK11B;CDK11B;CDK11B;L	2.58E-05	-0.379756963
cg22164207	CRNN	7.23E-05	-0.379848362
cg17884698		0.0050758	-0.37997778
cg05897699	SCAMP2	4.34E-05	-0.380112981
cg19036372	PTPRN2;PTPRN2;PTPRN2	7.23E-05	-0.380124728
cg19014792	CARD11	1.11E-05	-0.380171187
cg22951556	LINGO3	0.009066563	-0.380195985
cg04270013		0.000791389	-0.380225771
cg13504055		7.23E-05	-0.380233066
cg04264560		9.24E-05	-0.380252989
cg11308319	HDAC4	4.34E-05	-0.380466473
cg24733614	CHST11	7.23E-05	-0.380509629
cg13758310	LETM1	0.000955793	-0.380533904
cg07616791		0.000117986	-0.380566392
cg27466999	LMF1	0.000290372	-0.380572037
cg19510565	LAPTM5	4.34E-05	-0.380579616
cg25940202		9.24E-05	-0.380663984
cg00353330		0.000791389	-0.380687073
cg16385865		0.00043997	-0.380690031
cg17656763		0.0050758	-0.380706654
cg08043592	GALP;GALP;GALP;GALP	0.001147425	-0.380777727
cg07585502		0.007873393	-0.380882071
cg25863735	ADAL	4.34E-05	-0.38096243
cg25344194		4.34E-05	-0.38096542
cg12128262	IER3IP1	0.000654035	-0.381006458
cg00059089	FMNL2	5.59E-05	-0.381106754
cg24918715	TRIM72	0.000791389	-0.381126812
cg16320419	BHLHE40	5.59E-05	-0.381129519
cg21143086		4.34E-05	-0.381211209
cg13298528	CXCR5;CXCR5	4.34E-05	-0.3812278
cg18741439	CSGALNACT1;CSGALNACT1;CSGALNACT1	7.23E-05	-0.381272826
cg01538731	KLHDC4	0.000148457	-0.381313659
cg27587780	IRF1	0.000117986	-0.381348226
cg25379762	GAS7	2.58E-05	-0.381350013

cg11507187	CAMK2A;CAMK2A	0.000233422	-0.381472642
cg12229979	MYO9B;MYO9B	3.34E-05	-0.381714327
cg07433769	C18orf1;C18orf1;C18orf1;C18orf1;C18orf1;C18o	4.34E-05	-0.381719883
cg24527636		0.000357885	-0.381732381
cg21192698	NFATC1;NFATC1;NFATC1;NFATC1	0.000654035	-0.381755113
cg08314949	RPTOR;RPTOR	7.23E-05	-0.381820828
cg22226904		0.000955793	-0.381858173
cg03553826		0.00043997	-0.382016258
cg00669330	C2orf85	0.003734675	-0.382060847
cg07311363		0.0050758	-0.382196413
cg22059469		0.000117986	-0.382212012
cg23815312	ARHGAP22	7.23E-05	-0.382246691
cg06048193		0.004357816	-0.382271746
cg05903720		0.000117986	-0.382278331
cg00175150	ECM1;ECM1	0.000117986	-0.382355115
cg02135427		0.004357816	-0.382402843
cg19115204	C12orf56;C12orf56	3.34E-05	-0.382500781
cg07328664	DIS3L2	5.59E-05	-0.382508152
cg24441810	TMEM177;TMEM177;TMEM177	0.006823935	-0.382555177
cg10054332	PVT1	0.000537905	-0.382590407
cg01713348	C16orf91	0.000187194	-0.382677048
cg18960324		3.34E-05	-0.382716631
cg16640008		0.000148457	-0.382864745
cg22946147	ZNF804B;MGC26647	0.000187194	-0.382902254
cg10809134		0.002714607	-0.38292817
cg23445604	ALS2CL	0.003734675	-0.382991324
cg27159096	CMTM2	0.000654035	-0.383166025
cg21122693	RNF4	0.00043997	-0.383181161
cg01476222	TRAF6;TRAF6	0.000117986	-0.383214957
cg16386293	ECM1;ECM1	0.001147425	-0.383325387
cg14491284		0.000148457	-0.383325869
cg13448775		5.59E-05	-0.383356214
cg06748146	HK1;HK1;HK1;HK1;HK1	3.34E-05	-0.383440697
cg27009704		9.24E-05	-0.383497649
cg07548313	CD1C	0.002714607	-0.383655466
cg16734875	GRHL3;GRHL3;GRHL3	0.000654035	-0.383745121
cg19654743	SEPT9;SEPT9;SEPT9;SEPT9;SEPT9;SEPT9	7.23E-05	-0.383807701
cg11747820	PTPRU;PTPRU;PTPRU	0.000290372	-0.38381514
cg24756642		0.000187194	-0.383886004
cg04986899	XYLT1	0.000955793	-0.383904822
cg22124590	HEY2	4.34E-05	-0.38396705
cg08548882	PLEKHG1	4.34E-05	-0.383983686
cg09464061	MGC2752	0.001948248	-0.383984067
cg12924120	C3orf37;C3orf37	0.000148457	-0.384015085
cg26574777	PCCA;PCCA	7.23E-05	-0.384022962
cg04884313	DYNC1H1	9.24E-05	-0.384117135
cg11231069	HDAC4	0.003189589	-0.384159636
cg04875041	VPS13D;VPS13D	0.000290372	-0.384227255
cg26881863	TRAPPC9;TRAPPC9	0.000648283	-0.38423236
cg02368508	TNFRSF17	4.34E-05	-0.384281362
cg03327570		7.23E-05	-0.38431251
cg10517535	GAS7;GAS7;GAS7	8.60E-06	-0.384504984
cg01053714	SLC12A6	0.001376172	-0.384508699
cg20582941	ARPP-21;ARPP-21;ARPP-21;ARPP-21;ARPP-2	0.006823935	-0.384535636
cg08743794	KIAA1949;NRM;KIAA1949	4.34E-05	-0.384598723
cg14804706	CD81	0.000648283	-0.384674021
cg12240358	HOMER2;HOMER2;HOMER2;HOMER2	0.003189589	-0.384873914
cg06657721		3.34E-05	-0.384927032
cg13808641	WNK2	4.34E-05	-0.384958511
cg05259303		9.24E-05	-0.385000415

cg20640246		0.001147425	-0.385081757
cg09262100	CDC42BPB	0.000117986	-0.385099138
cg11194613	CTTN;CTTN	0.000955793	-0.385219086
cg01716252	ADARB1;ADARB1;ADARB1;ADARB1;ADARB1;	9.24E-05	-0.385307238
cg07076850		5.59E-05	-0.385364623
cg14373410	TSHR;TSHR;TSHR	0.001639597	-0.385381239
cg16785077	MX1	0.000148457	-0.385461291
cg23465650	EBF1	0.003734675	-0.385470884
cg07206827	LINGO4	5.59E-05	-0.385477097
cg11812071	TNFAIP3	0.009066563	-0.38565932
cg04731448	SCT	0.000791389	-0.385674775
cg25251562	ALLC	0.001948248	-0.385790634
cg08880369	IFT140;TMEM204	0.000187194	-0.385859955
cg14608550	SEPT9;SEPT9;SEPT9;SEPT9;SEPT9	7.23E-05	-0.385943669
cg27017580	BRF1;BTBD6	0.000290372	-0.386076005
cg01715572	ARMC9	1.96E-05	-0.386212356
cg23344241	SPTBN2	0.001147425	-0.386214665
cg13943333	AFF1;AFF1	0.000187194	-0.386248378
cg10952171		0.000290372	-0.386265063
cg22542139	PEMT;PEMT;PEMT	2.58E-05	-0.386282855
cg25597952	MAD1L1;MAD1L1;MAD1L1	0.002304201	-0.386306465
cg01809639	RCSD1	3.34E-05	-0.386327554
cg23863157		1.50E-05	-0.386336789
cg16873863	SLC22A18AS;SLC22A18	9.24E-05	-0.386371491
cg15094117		0.002450672	-0.386390895
cg08945443	ZMYND17	0.000233422	-0.386575638
cg07608867		0.000148457	-0.386582829
cg20560283		0.000791389	-0.386588845
cg16969872	RBM26	2.58E-05	-0.386673612
cg18687314		9.24E-05	-0.386675311
cg01331275		9.24E-05	-0.386721232
cg17565299	TMEM129;TMEM129	5.59E-05	-0.386833195
cg26375587	BOC	0.001147425	-0.386911771
cg22110158	ST14	0.000148457	-0.386928443
cg23124325		0.000117986	-0.386948868
cg16584662	PML;PML;PML;PML;PML;PML;PML;PML;PML	0.000290372	-0.386971764
cg11014810	SH3PXD2A	0.000233422	-0.387039354
cg11555873		0.00043997	-0.387077459
cg03622263		2.58E-05	-0.387120681
cg08780166	SLC8A1;SLC8A1;SLC8A1;SLC8A1	0.002040485	-0.387144333
cg08449164	SPTBN1;SPTBN1	9.24E-05	-0.387162541
cg03505866	KIAA0247	0.000654035	-0.38718324
cg26217846	DIAPH1;DIAPH1	1.96E-05	-0.387193314
cg12221864	WDR43	0.002304201	-0.387222284
cg01980810	SPTBN2	0.001147425	-0.387261277
cg03647778	ADAM19	0.000187194	-0.387357337
cg15700989		0.001147425	-0.387521352
cg20630690	ZGLP1	8.60E-06	-0.387539809
cg02931058	KLHL30	5.11E-05	-0.387608308
cg05054910		0.000148457	-0.387647375
cg05132782		3.34E-05	-0.387737457
cg16535080	HLA-E	0.000955793	-0.387912925
cg10376360		0.00043997	-0.388040416
cg02309230		0.000537905	-0.388041774
cg23048001	MAD1L1;MAD1L1;MAD1L1	0.001147425	-0.388079827
cg10500570	MIR599;VPS13B;VPS13B;MIR875	0.001639597	-0.388242299
cg03617572	OSBPL5;OSBPL5;OSBPL5	3.34E-05	-0.388295445
cg03004102	CD34;CD34	4.34E-05	-0.388311614
cg14930864	YWHAQ	0.000233422	-0.38837833
cg15128555	IGFBP2	0.000537905	-0.388413324

cg02286717	SYNPO2L;SYNPO2L	7.23E-05	-0.388431509
cg07784959	CXCR4;CXCR4	0.000117986	-0.388504506
cg07578772	FAM194A	0.000148457	-0.388524553
cg24361098	BCL11A;BCL11A;BCL11A	4.34E-05	-0.388618255
cg02817932	KIAA0182;KIAA0182	0.000955793	-0.388620162
cg21218687	KIF21B	0.001291055	-0.388660371
cg15584954	DIP2C	0.000187194	-0.388688305
cg01042641	IFT140	0.000148457	-0.388711019
cg25772418	GPR133	0.000537905	-0.38873613
cg14467066	BAHCC1	4.34E-05	-0.388755082
cg12223258	DCAKD;DCAKD	1.96E-05	-0.388786365
cg18783047	ZGLP1	1.11E-05	-0.388884416
cg20745620		0.000791389	-0.388885525
cg06820006	GPR133	0.000233422	-0.388888386
cg22450342	MR1	1.96E-05	-0.388934909
cg07613391	TGFBR2;TGFBR2	2.58E-05	-0.389014849
cg17400366	ZGLP1	8.60E-06	-0.389032009
cg06113708	COMTD1	0.00043997	-0.389036332
cg03999941		0.000791389	-0.389077201
cg13354414	ZNF804B;MGC26647	0.000148457	-0.389083195
cg12136950	ITPKB	3.34E-05	-0.389135927
cg06443678		0.001948248	-0.389220933
cg02671711	RPTOR;RPTOR	0.000148457	-0.389249034
cg05166473	BANP;BANP	0.000233422	-0.389252071
cg07956000		0.000148457	-0.389283542
cg23184226	TECPR1	9.24E-05	-0.389290472
cg17123334		0.003734675	-0.389408873
cg12977548	DHX15	4.34E-05	-0.389462541
cg01005441	ARHGEF10	0.001376172	-0.389467884
cg05295930	ACSF3;ACSF3;ACSF3	0.000117986	-0.389503933
cg21965980	FXYD3;FXYD3;FXYD3;FXYD3;FXYD3	0.00043997	-0.389538546
cg02989448	SLC45A1	4.34E-05	-0.389631229
cg00827581	RAVER1	1.50E-05	-0.389655372
cg20546215		0.005897668	-0.389691879
cg01803461		0.001376172	-0.389721041
cg20555462	UBASH3B	0.001639597	-0.389793998
cg26593521	FAM71B	0.002714607	-0.389799662
cg18851795		0.000148457	-0.389830369
cg16161418	KIAA1949;KIAA1949	0.000357885	-0.389835126
cg00405484	EIF2C2;EIF2C2	0.000654035	-0.389990045
cg21515305	ELFN2	0.000791389	-0.389995646
cg04848693	MAD1L1;MAD1L1;MAD1L1	0.007873393	-0.389996499
cg23201527		0.000357885	-0.390016779
cg17341502	BRF1;BRF1	0.000955793	-0.390024162
cg02376282	HCCA2	0.001147425	-0.390051249
cg02236941		0.003734675	-0.390169097
cg04141975		0.004357816	-0.390236469
cg01243072		5.59E-05	-0.390327943
cg15625636	GPR65	0.003189589	-0.390343751
cg00924622	CROCCL2	9.24E-05	-0.390361031
cg00393373	ZNF518B	0.00043997	-0.390423812
cg24933856	LOC100188949	4.34E-05	-0.39045087
cg25354657	APLP2;APLP2;APLP2;APLP2;APLP2;APLP2	4.34E-05	-0.390485588
cg01894985	MYLK;MYLK;MYLK;MYLK	2.58E-05	-0.390515946
cg02167837		0.000537905	-0.390537128
cg18516609	ZEB1;ZEB1;ZEB1;ZEB1;ZEB1	0.000791389	-0.390565649
cg02676052	LCP2	9.24E-05	-0.390623249
cg04554272	LMF1	0.000955793	-0.390677356
cg05872570		0.000290372	-0.390770957
cg09437994	CBFA2T3	1.96E-05	-0.390848678

cg17491300	ARHGEF10	0.001639597	-0.390904387
cg02403929	RPTOR;RPTOR	0.000654035	-0.390915957
cg10661263		5.59E-05	-0.390972207
cg23237276		3.34E-05	-0.391059297
cg19365614	TTC24	0.000654035	-0.391068207
cg03663984	LMOD3	4.34E-05	-0.3910865
cg03281139	NFIC;NFIC	0.000117986	-0.3911803
cg15235922	MTSS1	0.001147425	-0.391226037
cg20647799		5.11E-05	-0.391353987
cg06224893	DNMT3A;DNMT3A;DNMT3A	0.001376172	-0.391384942
cg05215277	ZNF434	0.001948248	-0.391457943
cg15829622	PFKL;PFKL	0.000290372	-0.391466898
cg03386536		0.000290372	-0.391483394
cg03565153	PHACTR1	0.001376172	-0.391532462
cg11086127	PTPRA;PTPRA;PTPRA	0.002714607	-0.391546024
cg01259220	C7orf20	0.0050758	-0.391584521
cg00661777	PIK3CG	1.11E-05	-0.391650302
cg19052272	ALLC	0.000117986	-0.391659695
cg04510420	BANP;BANP	0.000955793	-0.391739063
cg20496896	LRRC2;LRRC2	0.000537905	-0.391762358
cg13443938		0.000117986	-0.391802277
cg21089050	GAS7	0.000791389	-0.391867602
cg01869288		0.002714607	-0.391890354
cg08059719		0.000117986	-0.391896725
cg13823415	TBXAS1;TBXAS1	0.003189589	-0.391910755
cg12700039	TBC1D16	0.000357885	-0.391938451
cg08102294	IQSEC1	5.59E-05	-0.392187493
cg07426634		0.000117986	-0.392223549
cg03199996	FAM65C	2.58E-05	-0.392291428
cg21173239	ELK3	9.24E-05	-0.392539026
cg00532477	SMPD4;SMPD4;SMPD4;SMPD4;SMPD4;SMPD	0.000570759	-0.392595753
cg12629515	HIST1H3J;HIST1H2BO	1.96E-05	-0.392601302
cg26700919	C18orf1;C18orf1	0.001376172	-0.392613483
cg00183468		0.000187194	-0.39265245
cg08245249	SLC8A1;SLC8A1;SLC8A1;SLC8A1	0.000537905	-0.392689567
cg10203922	HHIP	0.000290372	-0.392742424
cg09267773		0.000955793	-0.392748602
cg05592114	C1R	0.000357885	-0.392760956
cg15514918		0.000117986	-0.392806853
cg01495332	GRB10;GRB10;GRB10	9.24E-05	-0.392809854
cg20159193	NUDT3	5.59E-05	-0.392834158
cg10296867	GAS7	9.24E-05	-0.392839461
cg15522953	ZMIZ1	0.000290372	-0.39304363
cg05154234	HDAC7;HDAC7	2.58E-05	-0.393061547
cg13619044	C7orf41	4.34E-05	-0.393146753
cg27012421		1.96E-05	-0.393151332
cg04096368	SLC37A1	0.001376172	-0.393195969
cg24326021	EPN1;EPN1;EPN1;EPN1	7.23E-05	-0.393206883
cg20681184	PIK3CD	0.000148457	-0.393271714
cg13974894		0.00043997	-0.393465927
cg19095143	TEX264;TEX264;TEX264	9.24E-05	-0.393466082
cg00920892	RAB11FIP1;RAB11FIP1;RAB11FIP1	2.58E-05	-0.3936084
cg06001786	CCNJL	0.001147425	-0.393610408
cg08364730	CREBBP;CREBBP	4.34E-05	-0.393678456
cg16012294	MAD1L1;MAD1L1;MAD1L1	5.59E-05	-0.393694411
cg21494075	PRDM10;PRDM10;PRDM10;PRDM10	0.001948248	-0.393819471
cg18076842	C11orf75	4.34E-05	-0.393840053
cg09936008	ZNF213;ZNF213	0.001639597	-0.39385719
cg09217898		0.000233422	-0.393910998
cg00223136		0.000791389	-0.393938455

cg20029153	MYO18A;MYO18A	0.000117986	-0.394046168
cg20414082		4.34E-05	-0.39424663
cg12338690	POM121	0.000117986	-0.394253669
cg03402235	ZFP106	0.000537905	-0.394289167
cg10687006	FAM188B	0.001948248	-0.394289224
cg02547426	RGS12;RGS12;RGS12	1.50E-05	-0.394488322
cg19919989	CD180	1.11E-05	-0.394498118
cg27480819	SOX5;SOX5;SOX5	4.34E-05	-0.394531241
cg06796885		0.000117986	-0.394534615
cg17783213	COL22A1	0.000791389	-0.39463045
cg04767507	ZMIZ1	7.23E-05	-0.394696484
cg21639922	PDE9A;PDE9A;PDE9A;PDE9A;PDE9A;PDE9A;I	0.009066563	-0.394817827
cg25635544		0.000233422	-0.394847838
cg03403168		0.000357885	-0.394909459
cg16560824	DENND3	0.001639597	-0.394924354
cg08289130		0.00043997	-0.394969
cg05450916	TGFBR2;TGFBR2	3.34E-05	-0.395005707
cg22413209	FBLN7;FBLN7	0.000233422	-0.395079651
cg04051927		0.000955793	-0.39511619
cg00976532	PDPK1;PDPK1	0.000187194	-0.395166988
cg22335223	FXYD2	1.50E-05	-0.395346162
cg19212779	FRYL	0.00043997	-0.395361828
cg21331947	CPNE5	3.34E-05	-0.395370109
cg03144619	GALNT2	0.007873393	-0.395377687
cg27090678		5.59E-05	-0.395453216
cg17904575	PPP2R5C;PPP2R5C;PPP2R5C;PPP2R5C;PPP2	1.96E-05	-0.395592355
cg10696539	LSM7	9.24E-05	-0.395598159
cg13351061	RPS6KA2	3.34E-05	-0.395624316
cg27569829	BCL2	7.23E-05	-0.395883231
cg13799081	RBM20	4.34E-05	-0.395928255
cg17837492	SH3RF3	5.59E-05	-0.395960798
cg15242630	MICAL3;MICAL3;MICAL3	0.001376172	-0.395967752
cg10903916	ASAP2;ASAP2	0.001948248	-0.395971651
cg24607603	KIAA2018	0.000334462	-0.396070423
cg05481243	BID;BID	0.000654035	-0.396085061
cg26736232	SCT	0.000537905	-0.396159553
cg21012788		0.005897668	-0.39622815
cg22040631		0.000233422	-0.396261333
cg23672659	KIAA1949;KIAA1949	2.58E-05	-0.396360086
cg20557159	SEPT9;SEPT9;SEPT9;SEPT9;SEPT9;SEPT9	0.001147425	-0.396638691
cg04854098	TSPAN5	0.000654035	-0.39666002
cg02642565	C16orf5	0.000187194	-0.396690755
cg24426483		2.58E-05	-0.396749965
cg06942637	SNAPIN	0.00043997	-0.396770116
cg07127883		0.002714607	-0.396814223
cg24143611	SNORD23;GLTSCR2	0.007873393	-0.396886085
cg15035590	LRIG1	0.000654035	-0.396919685
cg14073818		0.001639597	-0.396979711
cg21881330	SLC24A4;SLC24A4;SLC24A4	0.006823935	-0.396989835
cg14256511		0.003189589	-0.397057225
cg10959907	RGS12;RGS12;RGS12	0.001147425	-0.397075639
cg18714560	SCT	5.59E-05	-0.397089515
cg09286367	MIR589;FBXL18	0.00043997	-0.397147153
cg27210863	SH2D4B;SH2D4B	0.000117986	-0.397412152
cg21141089		4.34E-05	-0.397723244
cg02766770	YPEL5;YPEL5;YPEL5;YPEL5	0.00043997	-0.397896502
cg01735277	CSK;CSK	2.58E-05	-0.397907332
cg10321623	B4GALNT3	0.000290372	-0.397930944
cg03112433	CDK14	3.34E-05	-0.397993604
cg12547839	UBE2O	0.000537905	-0.398056693

cg05226180	KCNMA1;KCNMA1;KCNMA1;KCNMA1	0.000290372	-0.398102577
cg06285909	TMCO7	0.000791389	-0.398160964
cg23025244	APOM	0.005897668	-0.398177941
cg13152690		2.58E-05	-0.398192756
cg11683242	LCK	0.000290372	-0.398283904
cg04525441	ADARB2	0.000187194	-0.398501766
cg26384465	LDB1;LDB1	0.001147425	-0.39859329
cg04781796	GABRD	0.000654035	-0.398620394
cg13554018	SPIN1	3.34E-05	-0.398634573
cg01891260	VPREB1	0.000148457	-0.398661042
cg23019125	RPTOR;RPTOR	4.34E-05	-0.398672681
cg11351527	FFAR1	5.59E-05	-0.398691202
cg26391080	SH2D4B	0.00043997	-0.398773808
cg05688618	ARHGAP24;ARHGAP24;ARHGAP24	0.00043997	-0.398842377
cg00517080	VPS26B	0.000187194	-0.398843409
cg06789686		2.58E-05	-0.398853808
cg10422067	TP53I11;TP53I11	0.000117986	-0.3990199
cg19830983		0.002304201	-0.399083051
cg09152136		0.002714607	-0.399109177
cg10741308	SORBS1;SORBS1;SORBS1;SORBS1;SORBS1	0.000233422	-0.399152973
cg13763661		0.000117986	-0.399181797
cg05705140		0.000290372	-0.399291836
cg03377767	MSGN1	4.34E-05	-0.399369133
cg26163153	RHOH	0.000791389	-0.399422658
cg15142214		0.000357885	-0.399547815
cg07139495		0.000654035	-0.399579685
cg07546433		0.000187194	-0.399587525
cg02830351	FKBP1A;FKBP1A	0.000955793	-0.399591623
cg04837170	CCDC154	0.007873393	-0.399699395
cg17604985		0.000290372	-0.399800966
cg24655669	MAD1L1;MAD1L1;MAD1L1	5.59E-05	-0.39980737
cg05035470		0.005897668	-0.399887002
cg16194437	CASD1	0.001147425	-0.399888249
cg00815266		5.59E-05	-0.399967728
cg01644640		2.58E-05	-0.400081996
cg20812722		1.50E-05	-0.400110303
cg00629382	MSRA;MSRA;MSRA	0.001639597	-0.400112907
cg14646977	NRM;KIAA1949	2.58E-05	-0.400129121
cg21775279	XKR8;SMPDL3B	0.000233422	-0.400179115
cg08789697	TMPRSS11A;TMPRSS11A	0.000791389	-0.400197095
cg06603309	KCNQ1;KCNQ1	3.34E-05	-0.400269591
cg27248474	CBFA2T3;CBFA2T3	0.000357885	-0.400292574
cg14779735	BANK1;BANK1;BANK1	0.000955793	-0.400485154
cg05949181		0.000290372	-0.400494624
cg16703466	ZNF672	0.000187194	-0.400517026
cg11132534		9.24E-05	-0.400547877
cg24090911	AHRR	3.34E-05	-0.400551266
cg14528056	GBAP1	7.23E-05	-0.400620571
cg16172923	MAD1L1;MAD1L1;MAD1L1	0.000537905	-0.400742253
cg00559635	RGS12;RGS12;RGS12	2.58E-05	-0.400747413
cg11105610	LGALS3BP	0.000654035	-0.400839223
cg05163330	ADPRHL1;ADPRHL1	0.000357885	-0.400839284
cg22928606	SMURF1;SMURF1	0.004357816	-0.400861379
cg15706621	SPTBN1;SPTBN1	0.000117986	-0.4009766
cg03518390	PACS2;PACS2	1.96E-05	-0.401001416
cg19723715	GPER;C7orf50;C7orf50;GPER;C7orf50	0.000290372	-0.401081353
cg26632171	MSGN1	0.000233422	-0.401084168
cg20769856		3.34E-05	-0.4011622
cg04369957	ST5;ST5;ST5	0.000233422	-0.401166287
cg00697095	GRB7;GRB7	0.000233422	-0.401474386

cg07609372	ASB10;ASB10;ASB10	4.34E-05	-0.4014834
cg13474692	MGMT	4.34E-05	-0.40156431
cg24485696	AGPAT5	0.000148457	-0.401595998
cg06193766	DIP2C	5.59E-05	-0.401605924
cg11253913	CALD1;CALD1;CALD1;CALD1;CALD1	0.000233422	-0.401619268
cg23462129	PLEKHG4B	0.000233422	-0.401682425
cg10812439	ZMIZ1	9.24E-05	-0.401695643
cg16182685	TSSC1	0.000955793	-0.401697647
cg03228516	CSNK1E;CSNK1E	0.000117986	-0.401730969
cg04153882	WIPI2;WIPI2;WIPI2;WIPI2;WIPI2	0.000654035	-0.401745522
cg20772590	SEPT9;SEPT9;SEPT9;SEPT9;SEPT9;SEPT9	3.34E-05	-0.401786762
cg01353670		0.000955793	-0.401807585
cg24803346	SFXN5	0.000148457	-0.401830416
cg18181904	PCTP;PCTP	0.000148457	-0.401927748
cg26542567		0.003189589	-0.402166245
cg15464148	LPAR5	0.000187194	-0.402323151
cg06238667	KIF13B	0.000537905	-0.402360789
cg15817163	SFRS8	0.000791389	-0.402390128
cg08466256	COL5A2	9.24E-05	-0.402480071
cg23318523	ZC3H3	0.000117986	-0.402516434
cg07637440		0.000357885	-0.402726029
cg03900378		3.34E-05	-0.402738713
cg15936385	CD81	0.001639597	-0.402752265
cg19863456	GPR157	0.003734675	-0.402841516
cg14792008	SNRNP70	0.000290372	-0.402890133
cg22115465	MAD1L1;MAD1L1;MAD1L1	0.000187194	-0.402968486
cg23218957	DACT1;DACT1	0.00043997	-0.403020261
cg20657903	C10orf128	7.23E-05	-0.403200905
cg14855367	UTS2D;CCDC50;CCDC50;UTS2D	9.24E-05	-0.403203343
cg16306078		0.000117986	-0.403260778
cg25578949		1.50E-05	-0.403350713
cg16872613		0.000148457	-0.403424574
cg02611507	HLTF;HLTF	0.001639597	-0.403434891
cg27109748	MAD1L1;MAD1L1;MAD1L1	0.000117986	-0.403533916
cg01942816	MIR589;FBXL18	4.34E-05	-0.403555463
cg20456620	INPP5A	0.000791389	-0.403568408
cg20781981	C5orf56	0.00043997	-0.403615665
cg26271001		3.34E-05	-0.403625359
cg12708109		9.24E-05	-0.40370081
cg13200575		0.000357885	-0.403829785
cg20234640	FCER2	7.23E-05	-0.403864856
cg12810837	CLEC2D;CLEC2D	3.34E-05	-0.403887038
cg13406085	ABCD2	0.000290372	-0.404042301
cg17095147	WDR1;WDR1	0.001948248	-0.404057226
cg15119640	CAPN8	0.000187194	-0.404196143
cg06933796		0.000117986	-0.404228668
cg23240213		3.34E-05	-0.404332357
cg19384997	CRYL1	0.007873393	-0.40437166
cg01323777	KCNAB3	0.003734675	-0.404505326
cg08939052		0.00043997	-0.404600642
cg24154631		0.000537905	-0.404671414
cg08461451	LINGO3	0.000357885	-0.404748582
cg03243902	TRAPPC9;TRAPPC9	0.000233422	-0.404911182
cg15561778	GAB1;GAB1	0.00043997	-0.404918213
cg01433914	ADD3;ADD3;ADD3	0.000955793	-0.404958956
cg22466678		0.000148457	-0.405011762
cg17404289	MAD1L1;MAD1L1;MAD1L1	0.002304201	-0.405056025
cg14885762	SEPT9;SEPT9;SEPT9;SEPT9;SEPT9;SEPT9	2.58E-05	-0.405211172
cg14422093		9.24E-05	-0.405227065
cg26069615	SLC38A1;SLC38A1	0.001948248	-0.405310067

cg19730314		0.002714607	-0.405611958
cg25112590		3.34E-05	-0.405716867
cg15532942	NFATC1;NFATC1;NFATC1;NFATC1;NFATC1	0.000187194	-0.405768323
cg23259289	RICH2	0.000187194	-0.405816543
cg14014879	ABCA4	0.000357885	-0.405821327
cg06334737	SCGB2A1;SCGB2A1	0.001376172	-0.405880902
cg15181441		0.000187194	-0.405886227
cg23100428	SNAI1	0.000187194	-0.40595924
cg23807924		0.00043997	-0.406074626
cg19194405	DDC;DDC	0.000148457	-0.406163073
cg08122051	WWOX	0.007873393	-0.406171397
cg22106284	POM121C	7.23E-05	-0.406225302
cg26367031	KCNMB3;KCNMB3;KCNMB3;KCNMB3	0.000117986	-0.406268708
cg19260718		0.000148457	-0.4062794
cg06925389		0.000187194	-0.406418773
cg25738350		0.00043997	-0.406452524
cg10518873	WNT5B;WNT5B	0.000117986	-0.406561679
cg04074908	TMEM63B	2.58E-05	-0.406587861
cg22344254	LOC100133612	9.24E-05	-0.406750587
cg02253978	CMTM2	0.000233422	-0.406761402
cg20969923	NIN;NIN;NIN	7.23E-05	-0.406811179
cg16727006	ZCCHC14	0.00041966	-0.406901076
cg07337598	ANXA9	2.58E-05	-0.406940545
cg13667124		5.59E-05	-0.406983303
cg16658412	MAD1L1;MAD1L1;MAD1L1	2.80E-05	-0.406984288
cg27383859	PDIA5;PDIA5	9.24E-05	-0.407008488
cg24924051	CMTM8	0.00043997	-0.407350866
cg04083553	GP9	7.23E-05	-0.407369124
cg17353680	KCNK12	0.000233422	-0.40749564
cg06829157		0.000148457	-0.407506364
cg17988326	RIMS3	2.58E-05	-0.407568858
cg10154880	TRRAP	0.000537905	-0.407571382
cg21923959	POU2AF1	5.59E-05	-0.407586324
cg12200164	TNFAIP3	0.000955793	-0.407627758
cg18183671	ARPP-21;ARPP-21;ARPP-21;ARPP-21	0.005897668	-0.407677218
cg19310167		8.60E-06	-0.407689017
cg07456815	XKR4	8.60E-06	-0.407935511
cg15719903	TAPBPL	7.23E-05	-0.408091561
cg00324827	MAPK10;MAPK10;MAPK10;MAPK10	0.000187194	-0.408092134
cg14921884		5.59E-05	-0.408276111
cg13643509	RPTOR;RPTOR	0.00043997	-0.408594307
cg17820022	PLCH2	0.000537905	-0.408671859
cg04522498	TRAPPC9;TRAPPC9	0.000290372	-0.408691011
cg05367967		0.000791389	-0.408700646
cg06469570	ACOX3;ACOX3	0.000148457	-0.408750272
cg17679987	ZFPM2	5.59E-05	-0.408753968
cg27072813	SLC12A7	5.59E-05	-0.408849205
cg14381994		0.000233422	-0.408872166
cg17910564	VDAC3;VDAC3	0.000290372	-0.408928518
cg00255699	TRIT1	0.000357885	-0.408973804
cg02617418	GCET2;GCET2	0.000290372	-0.408976223
cg00969893	TLN2	0.000148457	-0.408995461
cg07720334	DNMT3A;DNMT3A;DNMT3A	7.23E-05	-0.408997607
cg02007534		0.000290372	-0.409042663
cg18912107	TBPL1	1.96E-05	-0.409102469
cg10694914	POU2AF1	3.34E-05	-0.409146981
cg18743793	IKZF1	0.000117986	-0.409197113
cg22664614	CPA5;CPA5;CPA5	0.000510765	-0.409211396
cg14905514		9.24E-05	-0.409222684
cg25844587		5.59E-05	-0.409305468

cg03075156	PRKCE	0.000357885	-0.409389298
cg13471712	PHF21A;PHF21A	4.34E-05	-0.409406978
cg04794465	APOM	0.004357816	-0.409545052
cg19422205	SEPT9;SEPT9;SEPT9;SEPT9;SEPT9	0.000955793	-0.409567394
cg05623815		0.000187194	-0.409607872
cg01389506	SSH1;SSH1;SSH1	0.000791389	-0.409659917
cg18676586	TNFRSF17	1.50E-05	-0.409830265
cg16875500		2.58E-05	-0.410000072
cg06431514	CUGBP2;CUGBP2;CUGBP2;CUGBP2	0.000117986	-0.410078093
cg14957089		3.34E-05	-0.410100226
cg26839652		0.001147425	-0.410163751
cg00042325	LOC728743	4.34E-05	-0.410205747
cg21171115	AKAP2;PALM2-AKAP2;PALM2-AKAP2;AKAP2	7.23E-05	-0.410241079
cg07633835	FBXO18;FBXO18	5.59E-05	-0.410302206
cg26889659	EXOC2	0.000233422	-0.410363541
cg17532753	HDAC4	7.23E-05	-0.410366189
cg22211917	LAIR1;LAIR1	3.34E-05	-0.410385121
cg01392180	LYRM4;LYRM4	0.001147425	-0.410405323
cg12391643		0.000148457	-0.41044208
cg09461851	LMF1	0.001147425	-0.410468066
cg27047494		4.34E-05	-0.410479034
cg03163759	ANKRD11	0.000233422	-0.410490682
cg23464041	ITPR3	0.000955793	-0.410503798
cg05584070	CARS2	0.004357816	-0.410504119
cg06529843	GZMK	0.006823935	-0.410510977
cg00256068	PREX1	0.00043997	-0.410555393
cg24045212	MAD1L1;MAD1L1;MAD1L1	5.59E-05	-0.410591688
cg13868473	MAD1L1;MAD1L1;MAD1L1	0.001639597	-0.410632363
cg20415945	HDAC4	0.003734675	-0.410676481
cg04681879	RPTOR;RPTOR	0.002714607	-0.410680461
cg16876219		4.34E-05	-0.410715009
cg15552491		0.001147425	-0.410811294
cg11846618	BRF1	0.000290372	-0.410914677
cg23558116		0.002714607	-0.410931613
cg08599437		9.24E-05	-0.411082331
cg18979491	LBP	2.58E-05	-0.411228817
cg13713821		5.59E-05	-0.411239021
cg01311102	PTGES2;PTGES2;PTGES2	1.96E-05	-0.411300748
cg18468844	PTAFR;PTAFR;PTAFR;PTAFR	2.58E-05	-0.411399059
cg11898646	EBF1	0.001948248	-0.411493923
cg01603073		0.000117986	-0.411502838
cg12771187		9.24E-05	-0.411522458
cg18148156	LIPF	0.000117986	-0.41155301
cg10784511	AGTR1;AGTR1;AGTR1;AGTR1	4.34E-05	-0.411564067
cg19091930	LRRC14B	0.000791389	-0.411575975
cg23669440	A4GNT	1.96E-05	-0.411633268
cg26776806	CBFA2T3;CBFA2T3	0.000148457	-0.411742408
cg26308704	PTPRN2;PTPRN2;MIR595;PTPRN2	0.000290372	-0.411749443
cg22156899	SECTM1	4.34E-05	-0.411756875
cg21356630	TTLL2	2.58E-05	-0.41176822
cg09552510	MPEG1	0.001147425	-0.411802195
cg19513987	MAD1L1;MAD1L1;MAD1L1	0.000537905	-0.411852744
cg04545079	CTCF	0.00043997	-0.411903838
cg17254229		3.34E-05	-0.411903935
cg18384277	LTB;LTB	0.000290372	-0.411938492
cg09201499	FAM38A	0.000357885	-0.412008808
cg01027405	IL7R	0.000357885	-0.412299771
cg23192749		0.000148457	-0.41248154
cg08140114	HECA	0.000537905	-0.412557469
cg13671536		0.000233422	-0.412694593

cg20415092		0.001376172	-0.412697491
cg15889594		0.001639597	-0.412705905
cg01502811		9.24E-05	-0.412705997
cg16286735	ITGB3	0.000117986	-0.412708854
cg09362796	HTT	0.000791389	-0.412709961
cg27566842	ADARB2	0.00043997	-0.412714449
cg26707052		0.000148457	-0.412768703
cg17579089	KIF9;KIF9;KIF9	5.59E-05	-0.412883513
cg01588379	PRKAG2;PRKAG2	0.000537905	-0.412913976
cg03800281	LRRC20;LRRC20;LRRC20	0.001376172	-0.412924266
cg10337290	ADAMTS7	0.000654035	-0.412956169
cg23910341		0.000654035	-0.413019124
cg08216099	PXDN	0.000233422	-0.413033205
cg01984854		0.001147425	-0.413119412
cg23222617	FOXN3;FOXN3	4.34E-05	-0.413176364
cg19988482		0.000654035	-0.413204574
cg10064525	FNDC7	3.34E-05	-0.413207515
cg16902863	SSPO	0.00043997	-0.413271498
cg24903893	EBF1	0.003189589	-0.413283545
cg02612971		0.000148457	-0.413293981
cg04949429	LHPP;LHPP	3.34E-05	-0.413388347
cg24823222		0.000148457	-0.413458428
cg25107522	DGKD;DGKD	9.24E-05	-0.413479481
cg08202743	ABTB2	7.23E-05	-0.41367141
cg02146383	C16orf52	0.009066563	-0.413685982
cg02835823		3.34E-05	-0.413697646
cg17128308	STX18	3.34E-05	-0.413840624
cg01296705	HRH2;HRH2	0.000187194	-0.413909878
cg03357721	ARHGEF7;ARHGEF7	0.001376172	-0.41408177
cg12424383		0.000187194	-0.414089122
cg17487741	RCBTB1	7.23E-05	-0.41411077
cg26714263	RPTOR;RPTOR	7.23E-05	-0.414216036
cg05036615	PHTF2;PHTF2;PHTF2;PHTF2;PHTF2	0.000148457	-0.414412273
cg23413954	SERINC5	7.23E-05	-0.414530514
cg12266861		0.000117986	-0.41453826
cg02088785	STK24	0.000117986	-0.414588913
cg10983484	LYSMD2;LYSMD2	7.23E-05	-0.414599947
cg27354893		0.00043997	-0.414606901
cg11354682	C19orf38	5.59E-05	-0.414633582
cg25821399	C3orf37;C3orf37	5.59E-05	-0.414711983
cg26946311	DECR2	7.23E-05	-0.414825429
cg02903589	KDM1B	2.58E-05	-0.414866179
cg06287775	FOXK1	0.000357885	-0.414872183
cg04207746		0.000187194	-0.414966271
cg00412094	SH3BP2	4.34E-05	-0.414987708
cg23493867	SYNPO2;SYNPO2;SYNPO2	0.000148457	-0.415161238
cg03134661	UHRF1;UHRF1	0.000654035	-0.415474981
cg10311020	ABI1;ABI1;ABI1;ABI1	0.000148457	-0.415494119
cg17022488		0.000117986	-0.415581865
cg07491702	CDAN1	0.003189589	-0.415659474
cg03540329	GK5	0.000117986	-0.415688398
cg13500819	MGC29506	2.58E-05	-0.415776203
cg12840818	NRXN2;NRXN2	1.50E-05	-0.415915117
cg25278786	GUK1;GUK1;GUK1	0.000187194	-0.416114683
cg27561099	MAP3K13	8.60E-06	-0.416143224
cg13521170	BCL2	0.000357885	-0.416389754
cg19983801	MUC4;MUC4;MUC4;MUC4;MUC4;MUC4	0.000187194	-0.416405245
cg24517738	RIMS3	0.000148457	-0.41653544
cg09817283	IQCE;IQCE	5.59E-05	-0.416642674
cg07319459		2.58E-05	-0.416835981

cg14763104	CD81	2.58E-05	-0.416863789
cg03302822	C10orf28	7.23E-05	-0.416885748
cg12150931	ZNF385A;ZNF385A	0.001639597	-0.416926357
cg24065451	ETS1	0.000233422	-0.416970601
cg26004235	KIAA1949;NRM;KIAA1949	2.58E-05	-0.4170599
cg04994970	COX8C;KIAA1409	0.006823935	-0.417140892
cg26328150	TRAPPC9;TRAPPC9	0.001147425	-0.417217988
cg11035730		7.23E-05	-0.417447968
cg15411840	CCDC162	5.59E-05	-0.417453439
cg26565476	RAB11FIP1;RAB11FIP1;RAB11FIP1;RAB11FIP	0.000187194	-0.417514579
cg22059211		0.002304201	-0.417517461
cg14364926	ZER1	0.000187194	-0.417538132
cg07061368	FKBP5;FKBP5;FKBP5;FKBP5	0.000654035	-0.417549369
cg08893087	FAM69A	4.34E-05	-0.417551223
cg01997884		0.000148457	-0.417577184
cg13888445	AK1	0.000233422	-0.417698265
cg07308257	EEPD1	0.000955793	-0.417728608
cg19573599		7.23E-05	-0.417753519
cg26598348		0.000148457	-0.417763805
cg15765353		0.001639597	-0.417803721
cg04338890		0.000233422	-0.417806113
cg18891604		9.24E-05	-0.417838123
cg14456143	GEN1;GEN1	0.001376172	-0.417839551
cg04336905	DIP2C	0.003734675	-0.417850598
cg21848117	SLC38A10;SLC38A10	4.34E-05	-0.417898869
cg16312609		0.000187194	-0.417912276
cg22013055	LOC100134259	4.34E-05	-0.417932994
cg13128443		0.000537905	-0.418029866
cg11105358		0.000791389	-0.418039011
cg04516672	FAM53B	9.24E-05	-0.418049695
cg19317600	ACOX3;ACOX3	0.007873393	-0.418126811
cg20580833	CRTAC1	0.000537905	-0.418168108
cg08428292		4.34E-05	-0.418329362
cg20793665		1.96E-05	-0.41839068
cg07611121	TRIO	0.000187194	-0.418425235
cg21329649	MAD1L1;MAD1L1;MAD1L1	5.59E-05	-0.418481227
cg10803218	N4BP3	0.000290372	-0.418572514
cg09145629		4.34E-05	-0.418777738
cg16784234		5.59E-05	-0.418793845
cg06861560		0.000148457	-0.418880865
cg07218880	UPF3A;UPF3A	2.58E-05	-0.418956299
cg27299033	RTN4RL1	0.000357885	-0.418995201
cg16555866	TBCD	2.58E-05	-0.41907908
cg24231380	C2orf85	0.000955793	-0.419139585
cg18647268	MACF1	0.000148457	-0.419246117
cg25556035	NFIX	0.001376172	-0.419424772
cg08875297		0.000148457	-0.419462162
cg12682972	MAP4K4;MAP4K4;MAP4K4	1.96E-05	-0.419588564
cg15287850	ST6GAL1;ST6GAL1;ST6GAL1	0.000117986	-0.419665162
cg21133433		7.23E-05	-0.419711311
cg21442730		0.00043997	-0.419727376
cg19149132		0.000233422	-0.419746682
cg21733794	BLCAP;BLCAP;BLCAP;BLCAP;BLCAP	0.000357885	-0.419941324
cg15833565	ERI3	0.000290372	-0.420008544
cg18048309	KIAA1688	0.000357885	-0.420299704
cg08621773		4.34E-05	-0.420319147
cg01368900	IQSEC1	1.96E-05	-0.420541605
cg01975786		0.000117986	-0.420635555
cg04855961		4.34E-05	-0.420781534
cg16722001	VCL;VCL	0.000117986	-0.420877105

cg24688143	PTPRN2;PTPRN2;PTPRN2	0.000117986	-0.421020126
cg26692749	RCAN3	7.23E-05	-0.421064511
cg01706991	BAALC;BAALC	1.96E-05	-0.421147974
cg11793977		0.000148457	-0.421227745
cg11898431		1.50E-05	-0.421254891
cg11307565	PXN;PXN	1.11E-05	-0.421519299
cg15348679		0.004878663	-0.421536888
cg26314399	PLEKHG4B	0.001639597	-0.421568315
cg03773862	ELMO1	0.000117986	-0.421782952
cg23079727	VGLL4;ATG7;VGLL4;ATG7;VGLL4;ATG7;VGLL	0.000290372	-0.421820607
cg21577598	CCDC57	3.34E-05	-0.421869803
cg18406792	C18orf1;C18orf1;C18orf1;C18orf1;C18orf1;C18o	4.34E-05	-0.421889519
cg00385956	NPHP4	0.002304201	-0.42190867
cg09305113	RPS6KA2	0.000117986	-0.421938063
cg07303805	NOMO1	5.59E-05	-0.421978625
cg16711597	STK24	0.000791389	-0.421978705
cg22175624	TBCD	7.23E-05	-0.422030968
cg06508085		9.24E-05	-0.422046034
cg03366285	FSD2;FSD2	0.000148457	-0.422070153
cg23531734	LOC100130691	0.000148457	-0.422153801
cg07394914	IRF2	0.000148457	-0.422258663
cg26513654	SNX10	0.001948248	-0.422283958
cg08075528	CSNK1G2	0.005742788	-0.422374863
cg07446753		9.24E-05	-0.42241427
cg16536740		4.34E-05	-0.422549628
cg02057796	CYGB	5.11E-05	-0.422781941
cg12453504	IRF2	0.000233422	-0.422788015
cg09693588		2.58E-05	-0.422855855
cg20438460		0.000148457	-0.423025449
cg08428188		0.000233422	-0.423078625
cg18069144		0.000955793	-0.42311073
cg16409368	IMPDH1;IMPDH1;IMPDH1;IMPDH1;IMPDH1;IM	0.000117986	-0.423144872
cg22082015	NR1D1	0.000117986	-0.423174702
cg15417287		4.34E-05	-0.423306361
cg00226923	FGD2	1.96E-05	-0.423369203
cg05433111	CD19	0.000117986	-0.423373891
cg16005592		2.58E-05	-0.423420768
cg25958098	SLC12A6;SLC12A6;SLC12A6;SLC12A6;SLC12/	0.000187194	-0.423534852
cg11256132	C1orf100	0.000654035	-0.423655011
cg20097219	TBC1D16	2.58E-05	-0.423727909
cg14613361		7.23E-05	-0.423788555
cg14129169	ZNF788	4.34E-05	-0.423797994
cg13947310	PLXNA4;PLXNA4	0.00043997	-0.423860146
cg22881435	RAB11FIP1;RAB11FIP1;RAB11FIP1	2.58E-05	-0.423863933
cg11908155	NEIL2;NEIL2;NEIL2;NEIL2	4.34E-05	-0.42391608
cg23882164		7.23E-05	-0.423936402
cg14631438	KCNJ1;KCNJ1;KCNJ1;KCNJ1	5.59E-05	-0.424006585
cg18393175		0.000357885	-0.424031496
cg17515767	ADPRHL1;ADPRHL1	0.000117986	-0.424134812
cg14557690	UAP1L1	9.24E-05	-0.424198189
cg08063160	LRRFIP1	4.34E-05	-0.424223903
cg21741081	ZP1	0.001147425	-0.424243303
cg20666585		9.24E-05	-0.424263519
cg00178728	GPR125	0.002304201	-0.424335419
cg18452449	TLR9	3.34E-05	-0.424341678
cg20387392	ZBTB20;ZBTB20;ZBTB20;ZBTB20	0.001639597	-0.424349057
cg15416352	MNT	0.000233422	-0.424354432
cg24804106	PSD3	0.000357885	-0.424393968
cg02142900		2.58E-05	-0.424446593
cg03145274	SLC45A1	9.24E-05	-0.424470522

cg19882315	BLK;BLK	0.000148457	-0.424758542
cg06960941	GPR144	3.34E-05	-0.424883864
cg11170796	TCF3;TCF3	0.000357885	-0.425061573
cg19048010	SSH2	0.000233422	-0.425101364
cg17479280	MEF2D	4.34E-05	-0.425129002
cg10869531	SH3BP2	7.23E-05	-0.425326063
cg16462433		4.34E-05	-0.425375726
cg09173565	RPTOR;RPTOR	0.001948248	-0.425388027
cg23204276	AP1B1;AP1B1;SNORD125;AP1B1	5.59E-05	-0.425423829
cg04505348	ADAM33;ADAM33	0.000233422	-0.425435545
cg01858089		0.001639597	-0.425498552
cg05707116	EIF2AK3	0.00043997	-0.425511998
cg02743256	MAD1L1;MAD1L1;MAD1L1	0.000187194	-0.425543314
cg26382697	CD81	5.59E-05	-0.425605817
cg19004971	PAG1	0.000187194	-0.425649887
cg14536906	NEK6;NEK6;NEK6;NEK6;NEK6;NEK6;NEK6	2.08E-05	-0.425669073
cg01977762	UHRF1;UHRF1	2.58E-05	-0.425977113
cg15997518		4.34E-05	-0.425985961
cg12961889		7.23E-05	-0.425996869
cg05180258	NUMB;NUMB;NUMB;NUMB	0.000117986	-0.426089144
cg13699650		2.58E-05	-0.426223062
cg18152830	TNFRSF13B	4.34E-05	-0.42622523
cg16576160		3.34E-05	-0.426290812
cg08203192	PRKCE	0.000117986	-0.426427506
cg01449469	KIAA1949;KIAA1949	0.000187194	-0.42666869
cg11630392	GPR171;MED12L;GPR171	1.50E-05	-0.4267588
cg01391022	WDR66	0.000290372	-0.426957912
cg04737759		5.59E-05	-0.426972277
cg00999904	ALLC	0.000290372	-0.427075165
cg03840351	PDCD6	0.000233422	-0.427123793
cg23736297	RPTOR;RPTOR	7.23E-05	-0.427134423
cg23367351	MAD1L1;MAD1L1;MAD1L1	2.58E-05	-0.427139798
cg10962223		1.96E-05	-0.427154321
cg17184161	TBCD	0.000187194	-0.427263486
cg12592387		9.24E-05	-0.427303778
cg01981433	TNFAIP3	0.000654035	-0.427353036
cg08002791	GPR133	0.000148457	-0.427450061
cg03161498		8.60E-06	-0.427569591
cg11341011		0.000117986	-0.427662626
cg15207662	HRH2;HRH2	0.000187194	-0.427776721
cg08497772		2.58E-05	-0.42785241
cg17477946	TBCD	0.000537905	-0.427869111
cg17845669		0.000955793	-0.427871683
cg19905757	CORO2B	3.34E-05	-0.428051721
cg21688152	B3GNTL1	0.000187194	-0.428177854
cg06522833	NAA15	0.000654035	-0.428224831
cg07546334		9.24E-05	-0.428295124
cg23174662	HIF1A;HIF1A	7.23E-05	-0.428301677
cg06639923	FBXL18	9.24E-05	-0.42840381
cg08876518	CMIP;CMIP	0.003189589	-0.428523881
cg18338046	TCF7;TCF7;TCF7;TCF7;TCF7;TCF7;TCF7	4.34E-05	-0.428529148
cg22110998		9.24E-05	-0.42856666
cg02482003		0.000791389	-0.428567478
cg24135923		0.005897668	-0.428599398
cg02393092	SET	0.001948248	-0.428641522
cg22702334	PLEKHG4B	0.000791389	-0.428658578
cg19602452	SPRR4	0.003486652	-0.428660757
cg01542019	TECR	0.003734675	-0.428673009
cg21261158	RASA3	0.000654035	-0.428791922
cg01192077	EBF1	0.002304201	-0.428947445

cg05524458	ANKRD33B	9.24E-05	-0.42911055
cg02744046	LIPC	0.000537905	-0.429253282
cg03282107		4.34E-05	-0.429258332
cg01829163	SLC7A5	0.000233422	-0.429280637
cg19089328	NADSYN1	4.34E-05	-0.429489818
cg10673192	TRAPPC9;TRAPPC9	0.000233422	-0.42953458
cg12774454	ABLIM2;ABLIM2;ABLIM2;ABLIM2;ABLIM2;ABLI	0.000233422	-0.429562179
cg01965380	LRRC8D;LRRC8D	3.34E-05	-0.429818968
cg24691891	PHC2	0.000233422	-0.429929898
cg08006309	IFT140;TMEM204	0.000537905	-0.430074466
cg03653585	DLC1;DLC1;DLC1	0.000187194	-0.430099948
cg05794411	COX8C;KIAA1409	0.006823935	-0.430205609
cg05991442	RECQL5;LOC643008;LOC643008	0.001291055	-0.430370709
cg21165812		3.34E-05	-0.430519397
cg13740598	LSM4	0.000187194	-0.43054095
cg23881099		0.003734675	-0.430557077
cg08559364	VGLL4;VGLL4	0.000955793	-0.430691979
cg27052418		0.004357816	-0.430856553
cg03736062	TIFA	9.24E-05	-0.431150104
cg17002328	CCDC88C	5.59E-05	-0.431210919
cg02811473	PPP1R16B	0.000117986	-0.431260085
cg08579962		5.59E-05	-0.431518908
cg03818572		7.23E-05	-0.43156247
cg05113898	RPTOR;RPTOR	0.000290372	-0.431600078
cg14102807	CD19	0.000148457	-0.431685785
cg11298144	PLEKHG4B	0.001376172	-0.431689959
cg02448743	KAZALD1	9.24E-05	-0.43170499
cg02505099	SEMA5B	0.000148457	-0.431742903
cg11680908		0.000290372	-0.431743352
cg01868869		0.004357816	-0.431827457
cg26035201	PSMG3;PSMG3	0.000148457	-0.431834277
cg10678215	HMHB1	0.000117986	-0.431907083
cg01824412		0.004357816	-0.432009032
cg15121276	CLPTM1L	0.009066563	-0.432038869
cg12926910	MACC1	0.000537905	-0.432083073
cg15914335	PXDN	9.24E-05	-0.432127086
cg05921699	CD79A;CD79A	0.000117986	-0.432164955
cg14606478	TRIM26	5.59E-05	-0.432169115
cg08009711	C2CD2;C2CD2	1.96E-05	-0.432247617
cg01554316	GALNT2	0.001639597	-0.432375724
cg01345354	SERPINB9	0.005897668	-0.432688678
cg16779976	BLNK;BLNK	0.00043997	-0.432699806
cg08766762		5.59E-05	-0.432782012
cg04820773		5.59E-05	-0.432797871
cg18169610	CD81	2.58E-05	-0.432837194
cg10068403		0.00043997	-0.432863147
cg26583584		5.59E-05	-0.43307066
cg01394349	CAPN8	0.001061731	-0.43329909
cg20841436	PACRG;PACRG;PACRG	1.96E-05	-0.433345925
cg24336808		0.000117986	-0.433458663
cg21811284	SEC14L1;SEC14L1;SEC14L1;SEC14L1;SEC14	3.34E-05	-0.433626095
cg13751548	PAX5	1.50E-05	-0.43366968
cg18072388	RAVER1	1.96E-05	-0.433699969
cg01203457	BRD1	9.24E-05	-0.433806499
cg21193975	WDR5;WDR5	0.001147425	-0.433850927
cg06454410	ZGLP1	1.11E-05	-0.433880237
cg01299332		7.23E-05	-0.433974738
cg16185947		0.000148457	-0.434022839
cg16410524	RCSD1	0.001948248	-0.434089693
cg26870057		9.24E-05	-0.434136653

cg03384915	SIN3B	0.000537905	-0.434299397
cg05302701	NFATC1;NFATC1;NFATC1;NFATC1;NFATC1	0.000233422	-0.434500106
cg26249168		0.000955793	-0.434564131
cg17200850	HRH2;HRH2	0.000654035	-0.434772183
cg08024174		0.000233422	-0.435063774
cg26657404	KIAA0182;KIAA0182	9.24E-05	-0.435079951
cg24981097	STK24	5.59E-05	-0.435128997
cg07053162	PPL	4.34E-05	-0.435148208
cg22747994	KCNK4	3.34E-05	-0.435162105
cg20931965	HIF1A;HIF1A	2.58E-05	-0.435255705
cg16071091	NFATC1;NFATC1;NFATC1;NFATC1;NFATC1	0.000537905	-0.435314328
cg00649632	ABCC1;ABCC1;ABCC1;ABCC1;ABCC1	0.000148457	-0.435341315
cg09139509	RPTOR;RPTOR	4.34E-05	-0.435346319
cg03914925		0.001147425	-0.435362939
cg00870269	LIPC	0.000654035	-0.435422339
cg21547557	C21orf33;C21orf33	3.34E-05	-0.435473193
cg19668724		0.000357885	-0.435676633
cg19004465	TBC1D16	0.000654035	-0.435844213
cg05615150	ARPP-21	0.000654035	-0.436263942
cg01098955		0.000290372	-0.43631863
cg17110675		0.000117986	-0.436397207
cg20943353	MAD1L1;MAD1L1;MAD1L1	0.001376172	-0.436409338
cg07929956	ST3GAL2	5.59E-05	-0.436460677
cg27043141	GCET2;GCET2	0.000187194	-0.436470445
cg23645046		4.34E-05	-0.436514937
cg19174554	DIAPH3	0.000357885	-0.436626233
cg10318744		4.34E-05	-0.436657445
cg08622666	RANBP9	7.23E-05	-0.436829309
cg17192381	BCL2	0.000117986	-0.437053947
cg19624425		0.000955793	-0.437436316
cg02156723		5.59E-05	-0.437465283
cg10863922	SERPINB9	0.007873393	-0.437499945
cg09375756	DEAF1	0.000654035	-0.437507605
cg13477178	DIP2C	7.23E-05	-0.437521821
cg05830220	KLHDC4	0.000148457	-0.437543716
cg03096954		2.58E-05	-0.437648349
cg21868798		0.000654035	-0.437838802
cg20604028		0.000233422	-0.437962737
cg23007665	C7orf44	1.11E-05	-0.437983526
cg10548805		0.000290372	-0.438275921
cg26574610	VPREB3	5.59E-05	-0.438394503
cg10557584	SLFNL1;SLFNL1	9.24E-05	-0.438520414
cg03540589	CSRNP1	7.23E-05	-0.438561023
cg06806158	RPS6KA2;RPS6KA2	0.001147425	-0.438769089
cg02452552		0.00041966	-0.438933186
cg02482730	SEPT9;SEPT9;SEPT9;SEPT9;SEPT9;SEPT9;S	0.00043997	-0.438958877
cg02563364		2.58E-05	-0.439097634
cg01516792	RPTOR;RPTOR	7.23E-05	-0.439099746
cg16695253	BANP;BANP	0.001147425	-0.439100122
cg21720802	PELI3;PELI3	3.34E-05	-0.439246237
cg24630825	GPM6A;GPM6A	1.11E-05	-0.439329623
cg21830221	HIVEP3;HIVEP3	7.23E-05	-0.439463395
cg09909478	ABR	4.34E-05	-0.439502215
cg17218041	ATP11A;ATP11A	0.000233422	-0.439532015
cg00411413	CDH13	0.003760629	-0.439532128
cg21546950	AK5;AK5	0.00043997	-0.439571098
cg24466100	CLIC4	0.003189589	-0.439695209
cg08897688	GSG1;GSG1	0.001948248	-0.439702968
cg08210507	MAD1L1;MAD1L1;MAD1L1	0.000290372	-0.439709178
cg19708055		9.24E-05	-0.439834067

cg00770754	ANKRD11	0.000654035	-0.440039065
cg23185774	FGD2	1.96E-05	-0.440192819
cg23275914	TTLL10;TTLL10	2.58E-05	-0.440211099
cg00516030	CTGF	0.000654035	-0.440236329
cg26527263		7.23E-05	-0.440294601
cg04499011	FGD2	2.58E-05	-0.440386792
cg22835851	WDFY4	0.000117986	-0.440483516
cg09299082	MYL4;MYL4	4.34E-05	-0.440678499
cg02988730	AMPD2;AMPD2;AMPD2	2.58E-05	-0.440697125
cg00416882	PIAS2;PIAS2	0.000148457	-0.440724333
cg07015784	SLC11A1	5.59E-05	-0.440791241
cg18495884	MAD1L1;MAD1L1;MAD1L1	0.000117986	-0.440826439
cg16522412		7.23E-05	-0.440843775
cg07466463	EIF2C2;EIF2C2	0.000117986	-0.440861972
cg14573810	MEGF11	0.001147425	-0.440956013
cg17920789	LRCH1;LRCH1;LRCH1	0.000290372	-0.441039664
cg17519749	MYT1L	3.34E-05	-0.441117201
cg02650080	C3orf21	4.34E-05	-0.441147439
cg23816537	ZP1	4.34E-05	-0.441671816
cg02928840	PLXNA2	2.58E-05	-0.441828544
cg01323104	AFF1;AFF1	5.59E-05	-0.441904365
cg08070491	SP2	0.001147425	-0.441914126
cg01988602		0.000537905	-0.441949558
cg04003327	ESPNL;SCLY	0.000955793	-0.441977723
cg07102001	LOC100129637	4.34E-05	-0.442038025
cg00390724		1.96E-05	-0.442054461
cg13076829		1.96E-05	-0.442141605
cg14875327		0.000290372	-0.442203188
cg02426739		0.00043997	-0.442242716
cg09276655	LOC100129637	0.000187194	-0.442308564
cg16323293	PACRG;PACRG;PACRG	0.000357885	-0.442309162
cg25817701	MAD1L1;MAD1L1;MAD1L1	4.34E-05	-0.442347668
cg14866547	EYA4;EYA4;EYA4	7.23E-05	-0.442422239
cg06469955	RPTOR;RPTOR	4.34E-05	-0.442543987
cg04541228	NEIL2;NEIL2;NEIL2;NEIL2	0.000526175	-0.442570409
cg17161520	TBC1D10C	1.50E-05	-0.44257066
cg10022248		2.58E-05	-0.442589351
cg21118486	RPS6KA2	0.000117986	-0.442709749
cg05856556	LOC728743	5.59E-05	-0.44277475
cg27168131	PDE9A;PDE9A;PDE9A;PDE9A;PDE9A;PDE9A;[illegible]	0.000187194	-0.442865228
cg26724841	RNF216;RNF216	4.34E-05	-0.442872122
cg04421348	SH2D4B;SH2D4B	0.000117986	-0.44313491
cg25072766	CARS;CARS;CARS;CARS	3.21E-05	-0.443259637
cg07145038		0.000290372	-0.443279022
cg10834480	TRAF3IP2;TRAF3IP2;TRAF3IP2	3.34E-05	-0.443366836
cg03138446	SLC27A3	0.000148457	-0.443407333
cg01702009	PTCRA;PTCRA	7.23E-05	-0.443659562
cg11937508	GPR1;GPR1	0.000357885	-0.443694303
cg06883279		0.000357885	-0.443794845
cg20278154	CHST11	0.000290372	-0.443923088
cg15860235	SH3BP2	7.23E-05	-0.444046286
cg11858499	A4GALT	5.59E-05	-0.444049338
cg19771748		5.59E-05	-0.444135977
cg11051295		0.000148457	-0.444188653
cg24011341	SOX5	0.000290372	-0.444301162
cg05707833	NFATC1;NFATC1;NFATC1;NFATC1;NFATC1	1.96E-05	-0.444342562
cg17333291	GPER;C7orf50;C7orf50;GPER;C7orf50;GPER	2.58E-05	-0.444395827
cg22335802	IFI16	0.000946184	-0.444599918
cg08428868		2.58E-05	-0.444737854
cg07768103	RNF44	0.000233422	-0.444936487

cg24869834	SLC7A11	0.000117986	-0.44494008
cg00293644	DCDC2B	7.23E-05	-0.444967073
cg08946854		0.001948248	-0.44498908
cg11699517	BAHCC1	2.58E-05	-0.44499983
cg09841898	MAP4K5;MAP4K5	0.000537905	-0.445146982
cg07078269	PLXND1	2.58E-05	-0.445379429
cg25589371		7.23E-05	-0.44556708
cg00424286	RPS6KA2;RPS6KA2	0.000148457	-0.445593913
cg15025569	F2RL3	0.000537905	-0.445846657
cg03353699	KIAA1949;KIAA1949	7.23E-05	-0.446049023
cg01692110	ATP11A;ATP11A	9.24E-05	-0.446075956
cg01588250		0.000117986	-0.44610345
cg00536718		0.000117986	-0.446267416
cg20805133	PDCD1	0.002714607	-0.446278721
cg21987515	FBXW7;FBXW7;FBXW7	4.34E-05	-0.44638968
cg01551441	EBF1	9.24E-05	-0.44645953
cg14276730	CHD6	0.000117986	-0.446484808
cg14841514	ZMIZ1	0.000290372	-0.446508723
cg25134567	PRKCA;MIR634	3.34E-05	-0.446525359
cg20951650	IRS1	0.000187194	-0.446536048
cg09459774	FOXP1;FOXP1	0.005897668	-0.446606668
cg19471856	LAMB4	0.000290372	-0.446678098
cg25709789	TAOK1	0.00043997	-0.446703005
cg10435235	ARHGEF7;ARHGEF7	0.000233422	-0.446744399
cg20618826	ZCCHC24	0.000187194	-0.446822306
cg13036546	HLA-E	7.23E-05	-0.44685681
cg11895835	C5orf13;C5orf13;C5orf13;C5orf13;C5orf13;C5or	4.34E-05	-0.446878084
cg15659527	TTC7A	0.000357885	-0.446882841
cg09009070	AUTS2;AUTS2	9.24E-05	-0.446935606
cg14085952		5.59E-05	-0.446983902
cg13482010	SEMA5B	4.34E-05	-0.44713263
cg14078070	RPS6KA2	4.34E-05	-0.447145983
cg26291600	ALOX5	0.000148457	-0.447172014
cg02980499	BLNK;BLNK	3.34E-05	-0.447227211
cg06458258		0.000117986	-0.4473097
cg21253742	ZNF592	3.34E-05	-0.447398424
cg01519464	RCAN3	2.58E-05	-0.447411914
cg11615509	SHROOM3	4.34E-05	-0.447593617
cg05134426	ZNF608	0.000290372	-0.447610037
cg08795515	RAB40B	5.59E-05	-0.447859663
cg19779166		0.000334462	-0.447910129
cg04722215	ARID5A	4.34E-05	-0.448203457
cg26503038	GNG7	0.000187194	-0.448213666
cg01785706		5.59E-05	-0.448220113
cg07813747	LOC100271836	0.00043997	-0.448434668
cg10113107	RRM1	0.000148457	-0.448535066
cg26826183	EIF3G	3.34E-05	-0.448654918
cg18465945	PEMT;PEMT;PEMT	1.96E-05	-0.449195954
cg08608952	CDC42;CDC42;CDC42	9.24E-05	-0.449285817
cg16066008	C6orf125	4.34E-05	-0.449312486
cg17843665	MIPEP	1.50E-05	-0.449521242
cg06288355	RAG1	5.59E-05	-0.449551847
cg05187549		9.24E-05	-0.449600419
cg08654262	DDAH1;DDAH1	0.001948248	-0.449626242
cg22762189	MSGN1	5.59E-05	-0.449634245
cg01719433	UCKL1AS;UCKL1	9.24E-05	-0.449659637
cg13553498	CLEC2D;CLEC2D	0.000187194	-0.449739406
cg10227830	GAB1;GAB1	3.34E-05	-0.449793631
cg12177944	PLXND1	5.59E-05	-0.450082336
cg24940706	TCF3;TCF3	1.96E-05	-0.450138175

cg08719515	SEMA6A	0.000187194	-0.450145232
cg06804344	GP9	5.59E-05	-0.450357284
cg14918359		4.34E-05	-0.450456338
cg15997393	MAD1L1;MAD1L1;MAD1L1	0.001639597	-0.450461806
cg06282596	SORBS1;SORBS1;SORBS1;SORBS1;SORBS1	0.000357885	-0.450504241
cg02095334	COL11A2;COL11A2;COL11A2	1.96E-05	-0.450568963
cg04277893		0.000791389	-0.450591842
cg17031475	GRASP	0.000148457	-0.450614332
cg05859441	ARHGAP17;ARHGAP17	0.000357885	-0.450623227
cg19024599	SH3RF3	0.002714607	-0.450761467
cg26795340	GPD1;C12orf62	3.34E-05	-0.450835258
cg09214551	NMT1;DCAKD	0.000187194	-0.450843189
cg02525637	CD81	4.34E-05	-0.450910613
cg20382047	C6orf25;C6orf25;C6orf25;C6orf25;C6orf25;C6orf	0.000791389	-0.450956598
cg08675117	RFX2;RFX2	0.000955793	-0.451171712
cg09132058		7.23E-05	-0.451328327
cg16725974	SYNE2;SYNE2	0.000537905	-0.451347596
cg09376583		0.000148457	-0.45134804
cg24727290	DDR1;DDR1	3.34E-05	-0.451633206
cg06069407	TMCO3	0.000233422	-0.451714163
cg16241062		0.005897668	-0.451788096
cg17717972	ARHGEF10	3.34E-05	-0.451806887
cg08570686		1.96E-05	-0.451941077
cg02585724	KIAA1199	3.34E-05	-0.451946138
cg20847766	ARHGEF7;ARHGEF7;ARHGEF7;ARHGEF7;AR	4.34E-05	-0.451972787
cg21818891	SLC1A2	0.000187194	-0.452024456
cg18722086	KIAA2018	9.24E-05	-0.452322054
cg13750802	LOC646982;LOC646982;LOC646982	4.34E-05	-0.452358236
cg26334358		0.000117986	-0.452533036
cg02939659		0.002304201	-0.452547054
cg01807748	ABCA4	0.000537905	-0.452642118
cg10601159	THAP4;THAP4	9.24E-05	-0.452660766
cg14316944		5.59E-05	-0.452772875
cg17633015	MAD1L1;MAD1L1;MAD1L1	0.000290372	-0.45296821
cg03238899	GALNT7	5.59E-05	-0.453080513
cg16626875	HK2	0.000290372	-0.453296687
cg16081096	IQSEC1;IQSEC1	2.58E-05	-0.453542302
cg20973476		7.23E-05	-0.453604257
cg24949488	DNTT;DNTT	4.34E-05	-0.453646335
cg00762029	IRF2	0.000357885	-0.453788414
cg21711862		0.000117986	-0.453833797
cg14950134	TSSC1	7.23E-05	-0.453947065
cg26864036		0.000148457	-0.45397653
cg11495377		0.000148457	-0.45402162
cg23464271	KIAA1409;COX8C	0.002304201	-0.45440529
cg06767314	ABR	1.96E-05	-0.454477413
cg01582937	SORBS3	4.34E-05	-0.454502391
cg03264729	PDE2A;PDE2A;PDE2A;PDE2A	0.000117986	-0.454538117
cg04911180	TBL1XR1	7.23E-05	-0.454731443
cg21074015	ENTPD4;ENTPD4	0.001639597	-0.454925898
cg19813135	TNFAIP3	0.007873393	-0.454967196
cg02947214		7.23E-05	-0.454974151
cg24181389	RPTOR;RPTOR	4.34E-05	-0.455027706
cg09217856	RGS12;RGS12;RGS12	7.23E-05	-0.45509972
cg08457158		7.23E-05	-0.455212454
cg19275008	CDC42EP3	0.000148457	-0.455264921
cg05128379		0.000117986	-0.455477684
cg07597976	CD19	0.000233422	-0.455572307
cg00077838		1.50E-05	-0.455578053
cg04248499	MYO15B	2.58E-05	-0.455753037

cg21211645	LARS2	4.34E-05	-0.455754161
cg17434577	RPTOR;RPTOR	3.34E-05	-0.455789404
cg12183032	TCF3;TCF3	0.000233422	-0.455818922
cg24007886	OBFC2B;SLC39A5	0.000357885	-0.45613507
cg19365697	TRIM26	0.000148457	-0.456184383
cg04450994	SLC22A23;SLC22A23	5.11E-05	-0.456285314
cg17574471	LETM1	0.000233422	-0.45642153
cg14685006	NCRNA00114;NCRNA00114	0.000187194	-0.456484871
cg00145915		0.000357885	-0.456588897
cg00509249	CCDC162	4.34E-05	-0.456665992
cg11435441		0.000290372	-0.456711104
cg07464408		5.59E-05	-0.456746078
cg15742700	BLK	1.11E-05	-0.456826757
cg10330187	ZC3H18	0.001376172	-0.456918822
cg11494699	RAG1	7.23E-05	-0.457076772
cg18590995	PARP1	5.59E-05	-0.457147076
cg19565738	ZAK;ZAK	0.00043997	-0.457278464
cg17397870	TRIM39;TRIM39	7.23E-05	-0.457302194
cg24244854	GRB2;GRB2	1.11E-05	-0.457387394
cg27107094	ELFN2	0.00043997	-0.457401077
cg12385032	BCAS3;BCAS3	0.002714607	-0.457433569
cg13853953	THAP4;THAP4	5.59E-05	-0.457478485
cg00259518		1.96E-05	-0.457579294
cg12756527	CUGBP2;CUGBP2;CUGBP2;CUGBP2	4.34E-05	-0.457756322
cg01922613	ABCA4	0.000187194	-0.457756769
cg03877829	C5orf13;C5orf13	0.00043997	-0.457818073
cg13855435	YPEL2	2.58E-05	-0.457855768
cg00793937		7.23E-05	-0.458602107
cg18664915	C7orf50;C7orf50;C7orf50	3.34E-05	-0.458699619
cg03425860		0.000537905	-0.458747115
cg08430680	TCF3;TCF3	3.34E-05	-0.458776266
cg08986727	TRIO	0.002714607	-0.458863559
cg12500731		0.000357885	-0.458873969
cg00643293		0.001147425	-0.45898771
cg16308790	NFATC1;NFATC1;NFATC1;NFATC1;NFATC1	3.34E-05	-0.459172769
cg11329449	SH3PXD2B	0.000654035	-0.45919634
cg24893378	PART1	2.58E-05	-0.459202412
cg00001793	ETV6	3.92E-05	-0.459275755
cg09832911	ALOX5	0.000187194	-0.459346535
cg02211519	CD81	1.11E-05	-0.459567755
cg10168330	PTK2B;PTK2B;PTK2B;PTK2B	2.58E-05	-0.459662923
cg02763540	UHRF1;UHRF1	7.23E-05	-0.459668681
cg21822146	ISM2;ISM2	0.000122184	-0.460016212
cg05373251		0.000148457	-0.460094139
cg15787636	TBCD	0.000148457	-0.460305495
cg11629955	CHCHD6	0.000187194	-0.460461258
cg05891759	ILDR1	4.34E-05	-0.460550416
cg14218435	SLC26A9;SLC26A9;SLC26A9	9.24E-05	-0.460598457
cg11922066	EBF1	0.005897668	-0.460599979
cg14037250	BRD3	0.00043997	-0.460610262
cg22855764	TANC1;TANC1	5.59E-05	-0.460678105
cg04005793	OBFC2B;SLC39A5	0.00043997	-0.460700623
cg21554588	SLC23A1;SLC23A1	0.000187194	-0.460843949
cg00863309	DTL	0.000654035	-0.461118056
cg18278519	DNTT;DNTT	0.000148457	-0.461269964
cg15220055		0.000290372	-0.461384235
cg23017002		3.34E-05	-0.461479777
cg09735627		3.34E-05	-0.461590462
cg17634401	APOM	0.006823935	-0.461611177
cg09822812	DDR1;DDR1;DDR1	3.34E-05	-0.461638544

cg01324963	GPR160	7.23E-05	-0.46169011
cg17980999	PNLDC1	1.96E-05	-0.461779971
cg25263238	BLNK;BLNK	2.58E-05	-0.46204097
cg23246886	TSPAN5	0.000187194	-0.462065083
cg11383802	DHCR7;DHCR7	0.001948248	-0.462132635
cg04396112	MAD1L1;MAD1L1;MAD1L1	0.000233422	-0.462324765
cg24130568	TBCD	0.000537905	-0.462507432
cg25456728	ARSG	5.59E-05	-0.462669703
cg14773365	HRH2;HRH2	0.000148457	-0.462811997
cg27022201	PRKAR1B;PRKAR1B;PRKAR1B;PRKAR1B;PRK	3.34E-05	-0.46283758
cg20826442		3.34E-05	-0.462855196
cg13172153	TNNI1	2.58E-05	-0.462909196
cg20603106	ARHGAP24;ARHGAP24	9.24E-05	-0.462926702
cg08285589	RAG1	0.000290372	-0.462929134
cg24570346		9.24E-05	-0.463019533
cg07660114	ADARB2	5.59E-05	-0.463242291
cg12778178	UCKL1AS;UCKL1	9.24E-05	-0.463243957
cg23531049	MAPKBP1;MAPKBP1	3.34E-05	-0.463260248
cg10691395		0.000537905	-0.46336233
cg13745692	FHIT;FHIT	9.24E-05	-0.463413704
cg18797923	AMT;AMT;AMT;NICN1;AMT;AMT	3.34E-05	-0.463456059
cg17078393	LCK	1.50E-05	-0.463550246
cg24092939	ADCY6	7.23E-05	-0.463636152
cg10466421	JAM2	0.000357885	-0.463643113
cg12840847	SEC14L1;SEC14L1;SEC14L1;SEC14L1;SEC14	2.58E-05	-0.463722651
cg20055861	MAP2K5;MAP2K5	1.96E-05	-0.464233603
cg12279968	LOC100133612	0.006823935	-0.464291675
cg27031754		4.34E-05	-0.464357857
cg10361659	RNASEH2B;RNASEH2B	5.59E-05	-0.464512087
cg19948549	D4S234E;D4S234E	0.000357885	-0.464573555
cg19742736	TBC1D20	9.24E-05	-0.464760685
cg19796955	TBK1	0.001639597	-0.464791027
cg01731500	SLC16A3;SLC16A3;SLC16A3	3.34E-05	-0.464992762
cg04715649	CDC42BPB	0.000148457	-0.465119267
cg09437283	IQSEC1;IQSEC1	9.24E-05	-0.465269766
cg03554394		0.000290372	-0.465354224
cg10443421		9.24E-05	-0.465530243
cg08845973	SPG7;SPG7	0.00043997	-0.465671786
cg04828493	CARS2	0.000357885	-0.465822021
cg04406620	CYB5B	0.000290372	-0.465934857
cg23754918	IQCE;IQCE	3.34E-05	-0.466072574
cg27562023	RPH3AL	9.24E-05	-0.466213072
cg04216051	HDAC4	0.001376172	-0.466277126
cg18313416	NUP210	0.000148457	-0.466296427
cg00258779		7.23E-05	-0.466302283
cg07018090	BANP;BANP	0.000148457	-0.466371481
cg12417466	ARPP-21	0.001147425	-0.466378835
cg07517739		0.000233422	-0.466385912
cg13823257		4.34E-05	-0.466396932
cg18067520	QRSL1	3.34E-05	-0.466770403
cg14374994	PDCD1LG2	0.001147425	-0.466828042
cg17601191		9.24E-05	-0.466850913
cg19215110	DDR1;DDR1	3.34E-05	-0.466876981
cg04324917	LOC100129637	2.58E-05	-0.46694177
cg22014112	TNFAIP3	0.006823935	-0.466954066
cg18184053		9.24E-05	-0.466954568
cg22889918		5.59E-05	-0.467151139
cg02574101	LOC91450	0.000148457	-0.467217921
cg01466491		7.23E-05	-0.467327947
cg12450872		0.000148457	-0.467509673

cg11316887	SVIL;SVIL	3.34E-05	-0.467540494
cg13103051	ST8SIA5	1.96E-05	-0.467629667
cg02286857	TTC7A	7.23E-05	-0.467649873
cg26635451		0.000148457	-0.467719819
cg25469923		7.23E-05	-0.467733021
cg13466700	NOX3;NOX3	0.000357885	-0.467803939
cg17804342	RGS10;RGS10	0.000955793	-0.46781666
cg15562346		0.000290372	-0.467870394
cg16675872		0.00043997	-0.467888829
cg13915752	CDK19	0.002714607	-0.467911897
cg10351692	POM121C	0.000117986	-0.46792426
cg12147994	SH3YL1;SH3YL1	0.000187194	-0.467950257
cg08412215		0.0050758	-0.468489842
cg17850055		3.34E-05	-0.468764969
cg06380725	TRAF3IP2;TRAF3IP2;TRAF3IP2;TRAF3IP2	7.23E-05	-0.469246275
cg27026786	SPATA13;SPATA13	5.59E-05	-0.46927405
cg01882471		0.0050758	-0.46933477
cg13544075	LCN6	9.24E-05	-0.469447552
cg26171271		4.34E-05	-0.469504305
cg25115537	ZHX2	7.23E-05	-0.469719472
cg15188939	ARIH1	1.11E-05	-0.469810887
cg19348272	PION	0.000117986	-0.470023592
cg24710951	KIAA1949;NRM;KIAA1949	3.34E-05	-0.470109287
cg01024458	RERE;RERE	0.000187194	-0.470396066
cg23463186	TSPAN14;TSPAN14	4.34E-05	-0.470470864
cg05487001	ARHGEF7;ARHGEF7;ARHGEF7;ARHGEF7;AR	4.34E-05	-0.470574141
cg26247508	SH2D4B;SH2D4B	0.000187194	-0.470789226
cg22510662	C5orf33;C5orf33	4.34E-05	-0.470908469
cg24749385		8.60E-06	-0.470998642
cg14029759	FARS2	2.58E-05	-0.471000566
cg20072792	RECQL5;LOC643008;LOC643008	0.000290372	-0.471077484
cg00874877	C6orf10	0.000148457	-0.471088557
cg00559992	PIK3R1	0.000148457	-0.471186879
cg02451443		7.23E-05	-0.471208402
cg08469255	DDR1;DDR1	9.24E-05	-0.471365511
cg07194798		0.000187194	-0.471405307
cg14741870	TRIM26	7.23E-05	-0.471471445
cg11697194		1.50E-05	-0.471507755
cg14360448		0.000654035	-0.47161855
cg02538169	GIGYF1	9.24E-05	-0.471780315
cg24375364	AMZ1	3.34E-05	-0.472381426
cg12738008	MYOM2	0.000117986	-0.472647904
cg10715426		0.000187194	-0.472759515
cg18967835	GPR18;GPR18;UBAC2;UBAC2;UBAC2	1.50E-05	-0.472901557
cg03801691	VGLL4;VGLL4;VGLL4;VGLL4	0.003189589	-0.472941546
cg19190593	LY86;LOC285780	4.34E-05	-0.473265593
cg12627726	KCNQ4;KCNQ4	3.34E-05	-0.473273805
cg27058221	PLA2G10	0.000537905	-0.473311006
cg16660220	AMBRA1	4.34E-05	-0.473410011
cg15930643	FLJ37543	0.000357885	-0.473588219
cg10145196	KIAA1949;KIAA1949	2.58E-05	-0.473716295
cg06429138		0.000233422	-0.473789346
cg13911959	ADARB2	0.000290372	-0.473828522
cg11814882	CYGB	1.96E-05	-0.474070369
cg04396303	PDE11A;PDE11A;PDE11A;PDE11A	0.000117986	-0.474151538
cg06323049	CD19	9.24E-05	-0.474719547
cg25599924	ACOX3;ACOX3	0.000233422	-0.474750628
cg13329862	DDR1;DDR1	3.34E-05	-0.475397086
cg07841371	TTLL10;TTLL10	5.59E-05	-0.475609945
cg21812313		2.58E-05	-0.475643103

cg02212339	TRPV1;TRPV1;TRPV1;TRPV1	0.000117986	-0.475688323
cg19477205	MIR154	0.000148457	-0.475801088
cg13323091		9.24E-05	-0.475822135
cg12400702	CAND2;CAND2	0.000357885	-0.475848598
cg06155414	MBNL2;MBNL2	7.23E-05	-0.475880834
cg18092363	NFATC1;NFATC1;NFATC1;NFATC1;NFATC1	3.34E-05	-0.475881834
cg07836226	RERE;RERE	0.000117986	-0.475956047
cg11257300	MOV10;MOV10	4.34E-05	-0.476150075
cg07435215	LOC729082	7.23E-05	-0.476219664
cg08845721	NR3C1;NR3C1;NR3C1;NR3C1;NR3C1;NR3C1;I	0.000537905	-0.476283349
cg17065712	ZMIZ1	9.24E-05	-0.476331916
cg23344706		0.000117986	-0.476534502
cg02615735	LY6G5C	4.34E-05	-0.47654373
cg03347287	DEAF1	2.58E-05	-0.47663544
cg00110171	NFATC1;NFATC1;NFATC1;NFATC1;NFATC1	4.34E-05	-0.476657497
cg25099065	ANXA4	5.59E-05	-0.476714283
cg20540372	BLNK;BLNK	2.58E-05	-0.476871061
cg24262870		0.000117986	-0.476889844
cg11018338	CCDC109A	0.000117986	-0.477057593
cg19644145	SP4	0.000537905	-0.47713454
cg09717923		5.59E-05	-0.477335766
cg00470817	LPAR5;LPAR5	0.000148457	-0.47750176
cg16026522	MAD1L1;MAD1L1;MAD1L1	4.34E-05	-0.477593426
cg25315312	MAD1L1;MAD1L1;MAD1L1	0.00043997	-0.477624542
cg03776082		0.000117986	-0.477628551
cg00227665	LOC100129637	4.34E-05	-0.478042943
cg12976793		0.000357885	-0.478242953
cg01986619	IQCE;IQCE	4.34E-05	-0.478446391
cg26569634	NOL10	0.000148457	-0.478458501
cg06936246	KCTD5	0.000187194	-0.478627081
cg26883434	C5orf13;C5orf13;C5orf13;C5orf13;C5orf13;C5orf	0.000290372	-0.47863404
cg23200357		0.001376172	-0.478856247
cg02111433	FTO	0.000148457	-0.478981447
cg01113988		0.000791389	-0.47902548
cg04555379	MAD1L1;MAD1L1;MAD1L1	5.59E-05	-0.47916358
cg10147394	BLK	7.23E-05	-0.479199248
cg16325984	HDAC4	4.34E-05	-0.479253589
cg09481537		0.000290372	-0.479336997
cg10658307		3.34E-05	-0.479425879
cg16531348	AGPAT3;AGPAT3	0.000117986	-0.479919454
cg07425109	RPS6KA4;RPS6KA4	2.58E-05	-0.480072996
cg17461214	CHML;OPN3	0.000117986	-0.480458382
cg27308982	NFATC1;NFATC1;NFATC1;NFATC1;NFATC1	0.000187194	-0.480790394
cg05058976	BTBD12	2.58E-05	-0.480848332
cg17342132	NR3C1;NR3C1;NR3C1;NR3C1;NR3C1;NR3C1;I	0.000791389	-0.480871792
cg18264210		3.34E-05	-0.480969125
cg15674899		0.000187194	-0.48114947
cg18098769	DNAH17	0.000233422	-0.48116877
cg07694806		7.23E-05	-0.481338535
cg22826986	BLK	9.24E-05	-0.481662475
cg21398111	SEC24C;SEC24C	0.000290372	-0.481691662
cg05292310	DSPP	0.000117986	-0.482465202
cg14784253	NOD1	1.50E-05	-0.482593567
cg03980250		7.23E-05	-0.482924643
cg18050634	BAHCC1	2.58E-05	-0.483063608
cg02052797		2.58E-05	-0.483144132
cg13396060	AKT3;AKT3	7.23E-05	-0.483325331
cg02642949	TSPAN17;TSPAN17;TSPAN17	0.000290372	-0.48332864
cg01074955	NPHP4	0.00043997	-0.483402277
cg01562813	NUP210	0.000187194	-0.48370742

cg07396047	LOC100129637	0.000117986	-0.483820052
cg14413262	SLC45A1	4.34E-05	-0.483968031
cg00582583	CBFA2T3;CBFA2T3	0.000233422	-0.484020695
cg24091162	CD79B;CD79B;CD79B	0.000187194	-0.484140808
cg16654610	ZNF721	0.000117986	-0.484306448
cg21656522		1.11E-05	-0.484563112
cg24971846	JAG2;JAG2	0.000357885	-0.484590441
cg12406992	MEX3A	9.24E-05	-0.484620005
cg19610383	DPYSL2	0.000233422	-0.484676205
cg14308466	VAV2;VAV2	0.000187194	-0.485199463
cg25256141	CUX1;CUX1;CUX1	0.000117986	-0.4852803
cg01093028		0.000290372	-0.485373463
cg27060355	SLC8A1;SLC8A1;SLC8A1;SLC8A1	7.23E-05	-0.485408901
cg08703857	IQCE;IQCE	3.34E-05	-0.48544017
cg13765961	MS4A1;MS4A1	1.96E-05	-0.485646557
cg05606799	KISS1	0.000290372	-0.485674626
cg02483101		9.24E-05	-0.485692923
cg19769715	CAMK2A;CAMK2A	7.23E-05	-0.485745045
cg05052463	FBXW7;FBXW7;FBXW7;FBXW7	2.58E-05	-0.485813729
cg18693141		5.59E-05	-0.486018175
cg00760938		3.34E-05	-0.486312635
cg01094114	ATG5	4.34E-05	-0.486334379
cg14283140	C9orf9	4.34E-05	-0.486542513
cg01157650	COL23A1	0.000290372	-0.486809389
cg16564828	MCF2L;MCF2L	0.000187194	-0.487065192
cg26811425		0.000148457	-0.487079936
cg13880868	GLE1;GLE1	9.24E-05	-0.487092972
cg17685004		3.34E-05	-0.487167603
cg04190888		0.000117986	-0.487325099
cg00449738		0.000187194	-0.487570796
cg23944920		0.00041966	-0.48758092
cg02889869		1.11E-05	-0.488200999
cg21221540		7.23E-05	-0.488638633
cg21765730	NOX3	0.000117986	-0.48872335
cg14535245	TTC7A	0.000290372	-0.488849046
cg02742418	SLC15A4	0.000233422	-0.488871601
cg08155625		7.23E-05	-0.489108075
cg14482811	LCN8	4.34E-05	-0.489109206
cg08971859	SPTBN2	0.000233422	-0.489342607
cg16329842	MAD1L1;MAD1L1;MAD1L1	7.23E-05	-0.489603928
cg05923736	ECHS1	0.000791389	-0.489685068
cg23093217	FAM49A	4.34E-05	-0.490001942
cg08036285		5.59E-05	-0.490136709
cg18560936		5.59E-05	-0.490369716
cg25140783	RCAN3	5.59E-05	-0.490515136
cg23093090	C10orf26;C10orf26	0.000148457	-0.490535447
cg23322523	TRIM55;TRIM55;TRIM55;TRIM55	0.000537905	-0.4905565
cg13364558	SEMA6A	0.000187194	-0.490693713
cg06895218		0.000290372	-0.490885556
cg20452738	ITPKB	4.34E-05	-0.491055259
cg14575739	LILRA2;LILRA2	3.34E-05	-0.491084235
cg19702194	RALB	0.000117986	-0.491114492
cg23842572	MPRIP;MPRIP	2.58E-05	-0.491142149
cg21606953	NRM	3.34E-05	-0.491162522
cg23001905		0.000955793	-0.491223755
cg17913306		0.000148457	-0.49131044
cg01160079	PRKCA;MIR634	5.59E-05	-0.491345481
cg03860768	BLK;BLK	5.59E-05	-0.491636139
cg02605292	GAS7;GAS7	4.34E-05	-0.491832781
cg10498814		0.000233422	-0.491959898

cg10105218	CD53;CD53	4.34E-05	-0.492228749
cg11767757	NCRNA00114	2.58E-05	-0.492327739
cg24361265	ELL3	0.000148457	-0.49237972
cg21110576		4.34E-05	-0.492583478
cg18422371	RAG1	9.24E-05	-0.492676381
cg11390978	SIN3B	0.001639597	-0.492773866
cg22498365	TBCD	2.58E-05	-0.492778638
cg04819180	TBCD	3.34E-05	-0.492887387
cg17055207	ACOX3;ACOX3	5.59E-05	-0.493308544
cg25683989	HVCN1;HVCN1	0.000148457	-0.493317847
cg24794322	PTPRN2;PTPRN2;PTPRN2	2.58E-05	-0.49357847
cg03689092	LIMS2;LIMS2	5.59E-05	-0.493690633
cg13577149	C5orf20	3.34E-05	-0.49376285
cg18612040	RPTOR;RPTOR	2.58E-05	-0.494404418
cg10443971	NR1H3;NR1H3;NR1H3	7.23E-05	-0.494662905
cg07554408		0.000148457	-0.494835307
cg14480046		9.24E-05	-0.494894635
cg03880987	CYTH3	5.59E-05	-0.495538761
cg11572080	TRAF3IP2;TRAF3IP2;TRAF3IP2	0.000187194	-0.495600126
cg08783514		3.34E-05	-0.49561381
cg00340349	CDK9	4.34E-05	-0.495752852
cg07993367	ATP6V0E2;LOC401431;ATP6V0E2	4.34E-05	-0.495772862
cg27527642	NRM	3.34E-05	-0.495785165
cg00270654	LOC100129637	0.000148457	-0.495872018
cg21027282	RAD51L1;RAD51L1;RAD51L1	0.000654035	-0.495997669
cg21239227	MYT1L	3.34E-05	-0.496145076
cg25393284		0.000290372	-0.496205143
cg06372779	OSBPL3;OSBPL3;OSBPL3;OSBPL3	7.23E-05	-0.496835046
cg08880524		0.000117986	-0.496845895
cg27565966	CD19	0.000117986	-0.497160775
cg00644351	RGS10;RGS10	0.001147425	-0.497173135
cg17983657		5.59E-05	-0.497218109
cg12427162	SFT2D2	7.23E-05	-0.497339752
cg19219423	PRKG1	0.000117986	-0.497570755
cg21570493		0.000187194	-0.497611628
cg22281206	INPP5J	1.96E-05	-0.498388718
cg22301418	ATP11A;ATP11A	9.24E-05	-0.498428826
cg09948419	MYO1G	8.60E-06	-0.498487734
cg01552275		0.001948248	-0.498497806
cg15939287	NRXN2;NRXN2;NRXN2	0.000148457	-0.498620611
cg04346861	HDAC4	4.34E-05	-0.498663333
cg12958813	ATP6V1G3;ATP6V1G3	0.000233422	-0.498841855
cg20539175	TMCO3	0.000187194	-0.49884858
cg05484949		7.23E-05	-0.498881077
cg01637125		5.59E-05	-0.499045595
cg05279137	AP2A2	0.000357885	-0.499184991
cg11972721	MED9	2.58E-05	-0.499327776
cg03678039	CYGB	0.000187194	-0.499379544
cg14522718	SLC25A25;SLC25A25;SLC25A25;SLC25A25	0.000290372	-0.499874143
cg15357156		0.000357885	-0.499914392
cg14764357	C7orf50;C7orf50;C7orf50	0.004357816	-0.500396381
cg02061967	C2orf48	4.34E-05	-0.500564874
cg03475687	TBCD	0.000187194	-0.500635932
cg00179026		4.34E-05	-0.50082234
cg04898039	MAD1L1;MAD1L1;MAD1L1	4.34E-05	-0.501049822
cg17894779	FHIT;FHIT	5.59E-05	-0.501050055
cg07467861	TUBB	9.24E-05	-0.501177492
cg14579184	TBCD	4.34E-05	-0.501387034
cg07710833		0.000290372	-0.501561188
cg18749990		0.000187194	-0.50174065

cg14907310		0.000187194	-0.501896441
cg26845390		0.000537905	-0.502483787
cg06008531		0.00043997	-0.502773078
cg02047211	RPTOR;RPTOR	4.34E-05	-0.502833378
cg11758283		2.58E-05	-0.50325775
cg10219816	PLCH2	9.24E-05	-0.503627746
cg18808929		3.34E-05	-0.503820885
cg18831134	ABR	1.96E-05	-0.503959285
cg16100486	RPS6KA2	4.34E-05	-0.503976945
cg19045002	BAHCC1	0.003189589	-0.50410173
cg11160673	BBX;BBX	1.96E-05	-0.504459591
cg07076751	KIAA1949;KIAA1949	4.34E-05	-0.504656489
cg25131632		0.002714607	-0.504786701
cg27618318	TPM1;TPM1;TPM1;TPM1;TPM1;TPM1;TPM1	0.000148457	-0.504915704
cg05579731	FADS3	9.24E-05	-0.504931954
cg12499316		1.96E-05	-0.505690412
cg03699749	OSBPL2;OSBPL2	0.001147425	-0.50601835
cg08763351	SPRR4	4.34E-05	-0.50636301
cg17634650		3.34E-05	-0.506504221
cg21679402	THAP4	5.59E-05	-0.506529538
cg26330304	ZNF710	7.23E-05	-0.506719955
cg20405697	NRM	2.58E-05	-0.506728028
cg04747693		9.24E-05	-0.507087876
cg00867406	TUBB3	0.000148457	-0.508056388
cg01320579	SEPT9;SEPT9;SEPT9;SEPT9;SEPT9	1.50E-05	-0.508492389
cg00162673	RNF11	0.000122184	-0.508674294
cg11472422	B3GNTL1	0.000117986	-0.508715439
cg16055052	NRM	2.58E-05	-0.508742631
cg00739471	ACTN1;ACTN1;ACTN1	2.08E-05	-0.508913389
cg21997986	BANK1;BANK1;BANK1	3.34E-05	-0.509257969
cg00294538		5.59E-05	-0.509556428
cg16596052	RGS10;RGS10	0.00043997	-0.509613984
cg20110257	NRM	0.000117986	-0.509764068
cg07083043	RASD1	3.34E-05	-0.50988611
cg03804400	SLC9A9	0.000117986	-0.509991925
cg00709979		4.34E-05	-0.510169121
cg21531416	DEAF1	0.000117986	-0.510362882
cg13777984	ENOX1;ENOX1	0.000148457	-0.510422931
cg22432760	TBCD	0.000955793	-0.510443956
cg08373904	HCCA2	5.59E-05	-0.510497253
cg06471487	HMHB1	0.000148457	-0.510548094
cg24777505	TBCD	4.34E-05	-0.510974188
cg24517978	ZDHHC19	4.34E-05	-0.511137468
cg16706613		7.23E-05	-0.511269091
cg18250453	TERF1;TERF1	0.000290372	-0.511455445
cg26160564	LOC100133612	3.34E-05	-0.511519802
cg16531664	TRAPPC9;TRAPPC9	0.000290372	-0.512281325
cg05760765		9.24E-05	-0.512535276
cg05119467	GNB1	5.59E-05	-0.512732719
cg10145533		4.34E-05	-0.512744703
cg10274108	TBC1D1	9.24E-05	-0.513065453
cg10402321	UBXN11;UBXN11;UBXN11	0.001948248	-0.513073794
cg01105418	ZNF238;ZNF238	7.23E-05	-0.513112001
cg15696968	KIAA1949;NRM;KIAA1949	4.34E-05	-0.513188628
cg23948703	EFTUD2;EFTUD2	3.34E-05	-0.513361725
cg05070250	C11orf74;RAG2	0.000233422	-0.513820097
cg22381248	HIVEP3;HIVEP3	3.34E-05	-0.513894856
cg16660971	RPTOR;RPTOR	0.000233422	-0.513901425
cg06555661	SFRS8	7.23E-05	-0.514576211
cg21156386	DEAF1	0.000290372	-0.514828925

cg06495310	APOM	0.003734675	-0.515114377
cg03504039	LRRFIP1	7.23E-05	-0.515247867
cg16165258	OSBPL8;OSBPL8	7.23E-05	-0.515345349
cg22212560	FRMD8	7.23E-05	-0.515434603
cg09469986		3.34E-05	-0.51553762
cg24163263		9.24E-05	-0.51559264
cg16625671		0.000187194	-0.51565968
cg14299931	ARHGEF7;ARHGEF7;ARHGEF7;ARHGEF7;AR	0.000290372	-0.515986043
cg01591579	INPP5D;INPP5D	2.58E-05	-0.516014153
cg20800391	NRM;KIAA1949	2.58E-05	-0.516130509
cg11704201		7.23E-05	-0.516151989
cg08499840	CCL11	2.58E-05	-0.516432487
cg11798406	NCRNA00114	7.23E-05	-0.516791873
cg02386957	ARHGEF7;ARHGEF7;ARHGEF7;ARHGEF7;AR	0.000187194	-0.516876698
cg27253454	USP43	2.58E-05	-0.516899907
cg15823954	HMHB1	5.59E-05	-0.516977063
cg23722274	DNTT;DNTT	0.000187194	-0.51711423
cg09511477	PILRB;PILRB;PILRB	7.23E-05	-0.517474607
cg10645314	ALLC	0.000117986	-0.518009151
cg13283094		3.34E-05	-0.518410831
cg03448915	TK2	7.23E-05	-0.518499469
cg01458510	SH3RF3	7.23E-05	-0.518746125
cg01744412		0.000148457	-0.518790085
cg18641463	MAD1L1;MAD1L1;MAD1L1	0.000357885	-0.518795407
cg19187110		0.000148457	-0.519082739
cg11661493	UBE2O	5.59E-05	-0.519421858
cg16580765	PRKD2;PRKD2;PRKD2;PRKD2	9.24E-05	-0.519523571
cg08057432	ANKRD11	0.000148457	-0.520090842
cg22256853	GCET2;GCET2	4.34E-05	-0.520502408
cg26514793	CHST3	4.34E-05	-0.520509944
cg10824972		7.23E-05	-0.520639455
cg12604490	FBXL4	0.000117986	-0.520786332
cg13356455	ATP10A	7.23E-05	-0.520844987
cg03980424		7.23E-05	-0.521225387
cg27377289		4.34E-05	-0.521543279
cg14279856	DDR1;DDR1	1.96E-05	-0.521750691
cg02696067	DDR1;DDR1	2.58E-05	-0.521958416
cg22621310	MAD1L1;MAD1L1;MAD1L1	0.000187194	-0.522182924
cg05204566	MAP1D	9.24E-05	-0.522195355
cg17930505	GALNT2	1.50E-05	-0.523047215
cg26089007		4.34E-05	-0.523062997
cg10471113	IFT140;TMEM204	0.00043997	-0.523249412
cg27183818		2.58E-05	-0.524524328
cg08444833	EIF2C2;EIF2C2	4.34E-05	-0.524847245
cg17811857		0.000357885	-0.525162206
cg06146665	USP36	4.34E-05	-0.525686948
cg19627093	ARHGEF10	1.50E-05	-0.525935966
cg19766988	EIF3G	4.34E-05	-0.52738663
cg17812123		5.59E-05	-0.527440556
cg20743720	HIVEP2	3.34E-05	-0.527466568
cg17158584	CTBP1;CTBP1	9.24E-05	-0.527578123
cg15730481	GPER;C7orf50;C7orf50;GPER;C7orf50;GPER	1.96E-05	-0.527954109
cg16219283	LTA;LTA;LTA	0.000117986	-0.528771081
cg06934654	ACSF3;ACSF3;ACSF3	5.59E-05	-0.529016572
cg26740109		2.58E-05	-0.529309564
cg14293575	USP18	1.96E-05	-0.530388011
cg18554866	C11orf74	5.59E-05	-0.530401343
cg21596498	POU2F2	1.50E-05	-0.531170344
cg03742250		9.24E-05	-0.531279795
cg14936008		9.24E-05	-0.5318446

cg23535322		0.000158823	-0.53239878
cg00128100	KIAA1949;NRM;KIAA1949	5.59E-05	-0.532401549
cg22612590	TMCO3	7.23E-05	-0.532852996
cg21999229	LTA;LTA;LTA	0.000233422	-0.533632292
cg22734058	LRRFIP1	9.04E-05	-0.533789255
cg05814100	RPTOR;RPTOR	7.23E-05	-0.534045449
cg08619712		1.50E-05	-0.534092416
cg08556541	SPTBN1;SPTBN1	0.000117986	-0.534614657
cg03917666		4.34E-05	-0.534804059
cg07142068	PFKFB3;PFKFB3	0.000205664	-0.536763043
cg22905350	NEK10	7.23E-05	-0.537408644
cg26348614	IQGAP2	5.59E-05	-0.538177024
cg09755397		4.34E-05	-0.538314605
cg16411445	NCRNA00114	0.00041966	-0.539113308
cg25020969	MAD1L1;MAD1L1;MAD1L1	9.24E-05	-0.539138089
cg05205074		0.000148457	-0.539222414
cg13429555		7.23E-05	-0.53960489
cg26337914	PTPRN2;PTPRN2;PTPRN2	2.58E-05	-0.540114651
cg17800669	BAALC;BAALC	0.000233422	-0.54026542
cg20071744	TRAPPC9;TRAPPC9	0.00043997	-0.540510172
cg15198193	KIAA1614	5.59E-05	-0.540534178
cg05909702		0.000187194	-0.540558183
cg21585707	FBXL18	3.34E-05	-0.54137578
cg20122488	MED9	3.34E-05	-0.541813088
cg03922146	KIAA1949;NRM;KIAA1949	3.34E-05	-0.541813905
cg10249814	MSI2;MSI2	9.24E-05	-0.542036025
cg12822816	INPP5A	7.23E-05	-0.542392852
cg23375948	MAD1L1;MAD1L1;MAD1L1	7.23E-05	-0.543000032
cg21786786	ATP11A;ATP11A	9.24E-05	-0.543830407
cg10987840		4.34E-05	-0.545335819
cg23424988		2.58E-05	-0.545569322
cg07665575	RAG1	0.000117986	-0.545603073
cg04947949	WDFY4	0.000187194	-0.547091866
cg11348106	SEC14L1;SEC14L1;SEC14L1;SEC14L1;SEC14	1.11E-05	-0.547533378
cg22633936	SH2D4B;SH2D4B;SH2D4B	0.000148457	-0.547912257
cg17995557	LHPP;LHPP	3.34E-05	-0.549278924
cg03930857	DNTT;DNTT	0.000187194	-0.551105158
cg10374402	SH2D4B;SH2D4B;SH2D4B	0.000117986	-0.551786677
cg23324787	C11orf74;RAG2;RAG2	4.34E-05	-0.551891911
cg26214026	ATXN2	0.000148457	-0.55194786
cg27157038	DNTT;DNTT	0.000148457	-0.553334672
cg10667102		0.000148457	-0.553640054
cg24035107	METT10D	4.34E-05	-0.554545078
cg08822897		1.96E-05	-0.554584366
cg06687504	RPS6KA2	4.34E-05	-0.555250468
cg07721872	LOC100129637	5.59E-05	-0.556199755
cg13299324	NCRNA00114	0.000148457	-0.557796011
cg18758433	RPTOR;RPTOR	1.96E-05	-0.559066172
cg06154633	RPTOR;RPTOR	7.23E-05	-0.559472979
cg16755833	TBCD	0.000148457	-0.559709335
cg14060402	ZCCHC7	0.000148457	-0.559788361
cg21820873	RPS6KA4;RPS6KA4	0.000117986	-0.560411447
cg25373630	BCL2	3.34E-05	-0.560759344
cg01012295	MYOM2	7.23E-05	-0.562816449
cg21507958	RPTOR;RPTOR	7.23E-05	-0.562996238
cg01598421	TG	7.23E-05	-0.563000673
cg18790741	NUP210	5.59E-05	-0.563293791
cg14182254	SFRS4	5.59E-05	-0.565756886
cg24667756	RPTOR;RPTOR	9.24E-05	-0.565935778
cg21885159	GNG7	4.34E-05	-0.568364926

cg27106643	NFATC1;NFATC1;NFATC1;NFATC1	1.96E-05	-0.568401175
cg26092233		2.58E-05	-0.570128819
cg23730277	BANK1;BANK1;BANK1	4.34E-05	-0.570879066
cg00861669	GALNT7	5.59E-05	-0.57359218
cg05776861	ARHGEF7;ARHGEF7;ARHGEF7;ARHGEF7;AR	4.34E-05	-0.57542523
cg06091647	RPTOR;RPTOR	2.58E-05	-0.576368555
cg19755815	TBCD	1.96E-05	-0.577610081
cg20311673	PXDN	7.23E-05	-0.580443578
cg20572915	C18orf1;C18orf1;C18orf1;C18orf1;C18orf1;C18o	2.58E-05	-0.580623172
cg26994885	CSRP2	7.23E-05	-0.58833587
cg07558704	SFXN5	0.000117986	-0.594780905
cg25588389		0.000148457	-0.595773371
cg19038462	ZEB1;ZEB1;ZEB1;ZEB1;ZEB1	2.58E-05	-0.60043753
cg17152850	PDE11A;PDE11A;PDE11A;PDE11A	5.59E-05	-0.60272097
cg07698804	TBCD	4.34E-05	-0.603601477
cg13241681	TMCO3	7.23E-05	-0.604081709
cg23474890	BCL7A;BCL7A	5.11E-05	-0.604157459
cg02243479	RPTOR;RPTOR	3.34E-05	-0.606111328
cg18675847	AFF1	4.34E-05	-0.607717294
cg23948811		2.58E-05	-0.609710677
cg25154959	RAG1	5.59E-05	-0.611653875
cg00225902	LRIG1	7.23E-05	-0.613813884
cg09969462	TBCD	1.50E-05	-0.618452812
cg02092514	TBCD	1.96E-05	-0.633820089
cg12583572	MAD1L1;MAD1L1;MAD1L1	4.34E-05	-0.649648641
cg12602405		4.34E-05	-0.660027414

Supplementary table 3: Gene ontology analysis of hypomethylated gene promoters in group ALL-2.

Gene ontology term	Fold Enrichment	Bonferroni	Benjamini	FDR	Fisher Exact
system development	1,7	1,2E-43	1,2E-43	1,9E-43	6,1E-47
cell-cell adhesion	2,9	1,1E-23	5,3E-24	1,7E-23	2,6E-27
organ development	1,6	1,7E-22	4,2E-23	2,7E-22	9,5E-26
anatomical structure morphogenesis	1,8	1,5E-22	5,1E-23	2,5E-22	7,6E-26
cell differentiation	1,6	8,0E-22	1,6E-22	1,3E-21	4,5E-25
cell-cell signaling	2,1	2,5E-20	4,1E-21	4,0E-20	9,8E-24
cell development	2,0	7,7E-19	1,1E-19	1,2E-18	3,3E-22
cell fate commitment	3,3	3,4E-15	4,3E-16	5,5E-15	7,0E-19
pattern specification process	2,5	5,6E-15	6,2E-16	9,0E-15	1,7E-18
organ morphogenesis	2,0	2,2E-13	2,2E-14	3,6E-13	4,0E-17
embryonic development	1,9	3,3E-13	3,0E-14	5,2E-13	1,9E-16
embryonic morphogenesis	2,3	5,4E-13	4,5E-14	8,8E-13	2,4E-16
tube development	2,5	7,8E-12	6,0E-13	1,3E-11	2,4E-15
embryonic organ development	2,8	8,9E-12	6,4E-13	1,4E-11	2,3E-15
regionalization	2,6	2,3E-11	1,5E-12	3,7E-11	6,8E-15
neuron projection development	2,3	5,7E-10	3,6E-11	9,2E-10	2,0E-13
regulation of system process	2,2	1,3E-9	7,6E-11	2,1E-9	5,0E-13
tissue development	1,7	1,4E-9	7,6E-11	2,2E-9	7,3E-13
embryonic development ending in birth or egg hatching	2,1	3,2E-9	1,7E-10	5,2E-9	1,3E-12
feeding behavior	3,7	4,4E-9	2,2E-10	7,1E-9	7,3E-13
regulation of multicellular organismal process	1,6	1,3E-8	6,3E-10	2,1E-8	8,0E-12
cell projection morphogenesis	2,2	3,0E-8	1,3E-9	4,8E-8	1,1E-11
cell part morphogenesis	2,2	1,0E-7	4,4E-9	1,6E-7	3,9E-11
cell fate specification	4,0	1,4E-7	5,7E-9	2,2E-7	2,0E-11
multicellular organismal response to stress	4,4	2,7E-7	1,1E-8	4,4E-7	3,2E-11
positive regulation of cellular process	1,4	4,5E-7	1,7E-8	7,3E-7	3,2E-10
positive regulation of biological process	1,3	7,3E-7	2,7E-8	1,2E-6	5,1E-10
forebrain development	2,5	1,4E-6	5,1E-8	2,3E-6	4,6E-10
regulation of nervous system development	2,3	2,2E-6	7,4E-8	3,6E-6	8,1E-10
positive regulation of neurological system process	4,2	2,2E-6	7,6E-8	3,6E-6	2,8E-10
positive regulation of biosynthetic process	1,6	4,3E-6	1,4E-7	6,9E-6	2,6E-9
cell morphogenesis	1,8	8,5E-6	2,7E-7	1,4E-5	4,2E-9
tube morphogenesis	2,5	9,0E-6	2,7E-7	1,4E-5	2,8E-9
cell migration	2,0	9,5E-6	2,8E-7	1,5E-5	4,1E-9
cell motion	1,7	1,0E-5	2,9E-7	1,7E-5	5,6E-9
positive regulation of nitrogen compound metabolic process	1,6	1,0E-5	2,9E-7	1,6E-5	6,0E-9
spinal cord development	4,0	1,6E-5	4,4E-7	2,6E-5	2,3E-9

appendage development	2,7	2,0E-5	5,3E-7	3,2E-5	5,7E-9
cellular component morphogenesis	1,8	2,4E-5	6,2E-7	3,9E-5	1,3E-8
Calcium signaling pathway	2,3	2,2E-6	1,1E-6	1,5E-5	4,4E-9
adult behavior	2,8	8,5E-5	2,1E-6	1,4E-4	2,3E-8
cell motility	1,8	9,3E-5	2,3E-6	1,5E-4	4,4E-8
positive regulation of metabolic process	1,5	1,0E-4	2,5E-6	1,7E-4	7,1E-8
positive regulation of cellular metabolic process	1,5	1,3E-4	3,0E-6	2,1E-4	8,8E-8
positive regulation of macromolecule metabolic process	1,5	1,5E-4	3,5E-6	2,5E-4	1,0E-7
regulation of developmental process	1,5	1,8E-4	4,0E-6	2,9E-4	1,1E-7
axon guidance	2,5	2,1E-4	4,5E-6	3,3E-4	6,4E-8
response to hormone stimulus	1,7	3,4E-4	7,2E-6	5,4E-4	1,8E-7
locomotory behavior	1,9	4,3E-4	9,0E-6	6,9E-4	2,0E-7
neurotransmitter transport	2,7	4,7E-4	9,6E-6	7,6E-4	1,3E-7
learning or memory	2,4	5,6E-4	1,1E-5	9,0E-4	1,8E-7
positive regulation of multicellular organismal process	1,9	6,3E-4	1,2E-5	1,0E-3	2,9E-7
appendage morphogenesis	2,5	9,1E-4	1,8E-5	1,5E-3	2,9E-7
regulation of localization	1,5	1,2E-3	2,3E-5	2,0E-3	7,7E-7
ion transport	1,4	1,6E-3	2,8E-5	2,5E-3	1,1E-6
secretion	1,8	1,5E-3	2,8E-5	2,5E-3	7,7E-7
morphogenesis of a branching structure	2,7	1,6E-3	2,8E-5	2,6E-3	4,4E-7
circulatory system process	2,0	1,6E-3	2,9E-5	2,5E-3	6,7E-7
regulation of biosynthetic process	1,2	2,1E-3	3,6E-5	3,4E-3	1,7E-6
adult locomotory behavior	3,0	2,5E-3	4,2E-5	4,0E-3	5,7E-7
anatomical structure formation involved in morphogenesis	1,7	3,3E-3	5,5E-5	5,3E-3	1,8E-6
regulation of transport	1,6	3,6E-3	6,0E-5	5,9E-3	2,2E-6
hindbrain development	2,9	4,5E-3	7,3E-5	7,3E-3	1,2E-6
synapse organization	2,8	6,1E-3	9,8E-5	9,9E-3	1,6E-6
fear response	4,7	6,3E-3	9,8E-5	1,0E-2	6,5E-7
regulation of nitrogen compound metabolic process	1,2	6,4E-3	9,9E-5	1,0E-2	5,1E-6
startle response	5,1	6,5E-3	9,9E-5	1,1E-2	5,5E-7
secretion by cell	1,9	7,9E-3	1,2E-4	1,3E-2	3,7E-6
regulation of cell differentiation	1,5	8,5E-3	1,2E-4	1,4E-2	5,3E-6
cellular chemical homeostasis	1,6	1,0E-2	1,5E-4	1,6E-2	5,9E-6
regulation of hormone levels	2,0	1,2E-2	1,7E-4	1,9E-2	5,0E-6
positive regulation of developmental process	1,7	1,3E-2	1,9E-4	2,2E-2	7,1E-6
tissue morphogenesis	1,9	1,8E-2	2,5E-4	2,9E-2	8,1E-6
negative regulation of biological process	1,2	1,8E-2	2,5E-4	2,9E-2	1,5E-5
inner ear morphogenesis	2,8	1,9E-2	2,6E-4	3,1E-2	5,0E-6
response to organic substance	1,4	2,0E-2	2,7E-4	3,3E-2	1,4E-5

Supplementary table 4: Gene ontology analysis of hypermethylated gene promoters in group ALL-2.

Gene ontology term	Fold Enrichment	Bonferroni	Benjamini	FDR	Fisher Exact
B cell activation	6,5	2,3E-5	2,3E-5	2,8E-5	2,2E-9
leukocyte differentiation	4,2	1,5E-3	5,1E-4	1,9E-3	2,4E-7
regulation of signal transduction	2,0	2,4E-3	6,1E-4	3,0E-3	8,8E-7
lymphocyte differentiation	4,8	1,5E-3	7,5E-4	1,8E-3	2,0E-7
hemopoietic or lymphoid organ development	2,8	1,8E-2	3,6E-3	2,2E-2	4,4E-6
B cell differentiation	6,4	2,7E-2	4,5E-3	3,3E-2	2,6E-6
